# A function-based typology for Earth’s ecosystems

**DOI:** 10.1038/s41586-022-05318-4

**Published:** 2022-10-12

**Authors:** David A. Keith, José R. Ferrer-Paris, Emily Nicholson, Melanie J. Bishop, Beth A. Polidoro, Eva Ramirez-Llodra, Mark G. Tozer, Jeanne L. Nel, Ralph Mac Nally, Edward J. Gregr, Kate E. Watermeyer, Franz Essl, Don Faber-Langendoen, Janet Franklin, Caroline E. R. Lehmann, Andrés Etter, Dirk J. Roux, Jonathan S. Stark, Jessica A. Rowland, Neil A. Brummitt, Ulla C. Fernandez-Arcaya, Iain M. Suthers, Susan K. Wiser, Ian Donohue, Leland J. Jackson, R. Toby Pennington, Thomas M. Iliffe, Vasilis Gerovasileiou, Paul Giller, Belinda J. Robson, Nathalie Pettorelli, Angela Andrade, Arild Lindgaard, Teemu Tahvanainen, Aleks Terauds, Michael A. Chadwick, Nicholas J. Murray, Justin Moat, Patricio Pliscoff, Irene Zager, Richard T. Kingsford

**Affiliations:** 1 Centre for Ecosystem Science, University of New South Wales, Sydney, New South Wales Australia; 2 New South Wales Department of Planning, Industry and Environment, Hurstville, New South Wales Australia; 3 IUCN Commission on Ecosystem Management, Gland, Switzerland; 4 Centre for Integrative Ecology, Deakin University, Burwood, Victoria Australia; 5 Department of Biological Sciences, Macquarie University, Sydney, New South Wales Australia; 6 School of Mathematics and Natural Sciences, Arizona State University, Glendale, AZ USA; 7 Norwegian Institute for Water Research, Oslo, Norway; 8 REV Ocean, Lysaker, Norway; 9 Sustainability Research Unit, Nelson Mandela University, Port Elizabeth, South Africa; 10 Wageningen Environmental Research, Wageningen University, Wageningen, The Netherlands; 11 School of BioSciences, The University of Melbourne, Melbourne, Victoria Australia; 12 Institute for Resources, Environment and Sustainability, University of British Columbia, Vancouver, British Columbia Canada; 13 SciTech Environmental Consulting, Vancouver, British Columbia Canada; 14 BioInvasions, Global Change, Macroecology-Group, Department of Botany and Biodiversity Research, University of Vienna, Vienna, Austria; 15 Centre for Invasion Biology, Stellenbosch University, Stellenbosch, South Africa; 16 NatureServe, Arlington, VA USA; 17 University of California, Riverside, CA USA; 18 Royal Botanic Garden Edinburgh, Edinburgh, UK; 19 School of GeoSciences, University of Edinburgh, Edinburgh, UK; 20 Departamento de Ecología y Territorio, Pontificia Universidad Javeriana, Bogotá, Colombia; 21 Scientific Services, South African National Parks, George, South Africa; 22 Australian Antarctic Division, Department of Climate Change, Energy, the Environment and Water, Hobart, Tasmania Australia; 23 Department of Life Sciences, Natural History Museum, London, UK; 24 Instituto Español de Oceanografía, Centro Oceanográfico de Baleares, Palma, Spain; 25 Manaaki Whenua—Landcare Research, Lincoln, New Zealand; 26 Department of Zoology, School of Natural Sciences, Trinity College Dublin, Dublin, Ireland; 27 University of Calgary, Calgary, Alberta Canada; 28 College of Life and Environmental Sciences Geography, University of Exeter, Exeter, UK; 29 Department of Marine Biology, Texas A&M University, Galveston, TX USA; 30 Hellenic Centre for Marine Research (HCMR), Institute of Marine Biology, Biotechnology and Aquaculture (IMBBC), Heraklion, Greece; 31 Department of Environment, Faculty of Environment, Ionian University, Zakynthos, Greece; 32 School of Biological Earth and Environmental Sciences, University College Cork, Cork, Ireland; 33 School of Life Sciences, South China Normal University, Guangzhou, China; 34 Centre for Sustainable Aquatic Ecosystems, Harry Butler Institute, Murdoch University, Perth, Western Australia Australia; 35 Institute of Zoology, Zoological Society of London, London, UK; 36 Conservation International Colombia, Bogota, Colombia; 37 Norwegian Biodiversity Information Centre, Trondheim, Norway; 38 Department of Environmental and Biological Sciences, University of Eastern Finland, Joensuu, Finland; 39 Department of Geography, King’s College London, London, UK; 40 College of Science and Engineering, James Cook University, Townsville, Queensland Australia; 41 Royal Botanic Gardens Kew, Richmond, UK; 42 Institute of Geography, Department of Ecology, Center of Applied Ecology and Sustainability (CAPES), Universidad Católica de Chile, Santiago, Chile; 43 Instituto de Ecología y Biodiversidad, Santiago, Chile; 44 Provita, Caracas, Venezuela

**Keywords:** Biodiversity, Conservation biology, Ecosystem ecology

## Abstract

As the United Nations develops a post-2020 global biodiversity framework for the Convention on Biological Diversity, attention is focusing on how new goals and targets for ecosystem conservation might serve its vision of ‘living in harmony with nature’^1,2^. Advancing dual imperatives to conserve biodiversity and sustain ecosystem services requires reliable and resilient generalizations and predictions about ecosystem responses to environmental change and management^3^. Ecosystems vary in their biota^4^, service provision^5^ and relative exposure to risks^6^, yet there is no globally consistent classification of ecosystems that reflects functional responses to change and management. This hampers progress on developing conservation targets and sustainability goals. Here we present the International Union for Conservation of Nature (IUCN) Global Ecosystem Typology, a conceptually robust, scalable, spatially explicit approach for generalizations and predictions about functions, biota, risks and management remedies across the entire biosphere. The outcome of a major cross-disciplinary collaboration, this novel framework places all of Earth’s ecosystems into a unifying theoretical context to guide the transformation of ecosystem policy and management from global to local scales. This new information infrastructure will support knowledge transfer for ecosystem-specific management and restoration, globally standardized ecosystem risk assessments, natural capital accounting and progress on the post-2020 global biodiversity framework.

## Abstract (web summary)

The International Union for Conservation of Nature’s Global Ecosystem Typology has been developed to provide a systematic framework for data on all of Earth’s ecosystems in a unified theoretical context to support biodiversity conservation and ecosystem services.

## Main

Sustaining ecosystem functions and services requires an understanding of ecological processes and mechanisms that drive ecosystem change^6^. Ecosystem functioning not only underpins biomass production, but also depends on and regulates the stocks and fluxes of resources, energy and biota^7^. These functions, together with ecological processes and species traits—collectively referred to as ‘ecosystem properties’ (see Supplementary Information, Glossary)—define and sustain ecosystem identity and shape ecosystem responses to environmental change, including anthropogenic changes^8^. Ecosystems with different species compositions may show functional convergence if their biota share similar traits and contribute to similar ecological processes (for example, in ref. ^9^). Together with ecosystem function, the identity of constituent biota is central to biodiversity concepts, conservation goals and human values^10^. Although ecosystem functions and ecological processes support both the diversity of biota and human well-being, global assessments of ecosystems^11,12^ continue to rely heavily on species metrics or simplistic land-cover proxies that convey limited information about ecosystems themselves. This limits our ability to diagnose trends and to design and resource on-ground management and policy solutions for slowing and reversing current declines in biodiversity and ecosystem services.

To serve the dual needs of sustaining ecosystem services and conserving biodiversity, ecosystem assessments require a global typology to frame comparisons and standardize data aggregation for analysing ecosystem trends and diagnosing their causes. To support applications throughout Earth’s diverse ecosystems, users and scales of analysis, this typology should encapsulate: (1) ecosystem functions and ecological processes; (2) their characteristic biota; (3) conceptual consistency throughout the whole biosphere; (4) a scalable structure; (5) spatially explicit units; and (6) descriptive detail and minimal complexity (see Supplementary Information, Appendix 1 and Supplementary Table 1.1 for rationale).

We used these 6 design criteria to review a sample of 23 global-scale ecological typologies, finding none that explicitly represented both ecosystem functions and biota (Supplementary Table 1.2). This limits the ability of ecosystem managers to learn from related ecosystems with similar operating mechanisms and drivers of change. Only three typologies encompassed the whole biosphere, but these lacked a clear theoretical basis, limiting their ability to generalize about properties of ecosystems grouped together. Ecological classifications based on tested and established theory are more likely to be robust to new information than classifications based only on observed patterns and correlations, which may prove unstable when new information emerges. Many typologies that we examined either did not describe their units in sufficient detail for reliable identification, or required diagnostic features that are difficult to observe. Others were based on biophysical attributes or biogeography, but approaches differed across terrestrial, freshwater and marine domains, precluding a truly global approach. In this study, we developed a Global Ecosystem Typology that meets all six design principles, thereby providing a stronger foundation for systematic ecosystem assessments, sustainable management and biodiversity conservation.

## Conceptual foundations

**Fig. 1:**
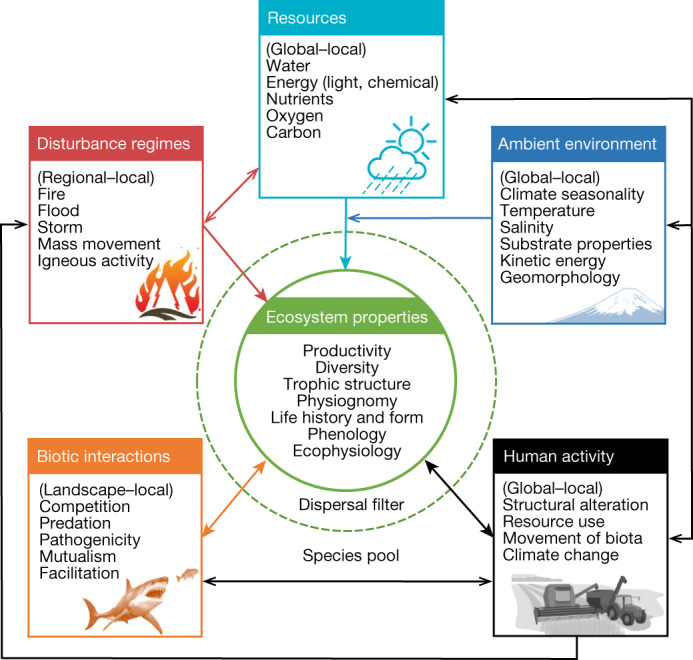
The generic model of ecosystem assembly underlying the Global Ecosystem Typology. Boxes represent abiotic (resources, the ambient environment and disturbance regimes) and biotic (biotic interactions and human activity) drivers that filter assemblages and form evolutionary pressures that in turn, shape ecosystem-level properties (inner green circle). The range of major organizational scales at which drivers operate are shown in parentheses, followed by a list of the major expressions of the drivers. The species pool is the set of ‘available’ traits on which the assembly filters and evolutionary pressures operate over short and longer time frames, respectively. Species pools are dynamic products of vicariance, dispersal and evolution that depend on biogeographic context and history. The outer green circle (dashed line) represents the contemporary dispersal filter that mediates the biota currently subjected to local selection by the abiotic and biotic filters and pressures. The inner green circle represents the properties (aggregate ecosystem functions and species-level traits) that characterize the ecosystem. Closed arrows show the influence of filtering processes on ecosystem properties. Feedbacks can occur whereby ecosystem properties modulate filtering processes (examples are indicated by bidirectional arrows). Interactions among drivers include indirect effects of human activity on assembly through other drivers (black open arrows) and the indirect effects of ambient environmental conditions on assembly by modulating resource availability or uptake (dark blue open arrow). Interactions among other drivers (omitted here for simplicity) are shown in ecosystem-specific adaptations of this generic model for each ecosystem functional group (level 3 of the typology) in Supplementary Information, Appendix 4. See Supplementary Information, Glossary, for explanation of terms. Details are in Supplementary Information, Appendix 2. Illustrations (wildfire icon; Japan mt. fuji; shark) DigitalVision Vectors via Getty Images.

We developed a conceptual model to inform the construction of the Global Ecosystem Typology, consistent with the six design principles, and to serve as a template for describing the units of classification. The model (Fig. 1) frames working hypotheses about the processes (or ‘drivers’) that shape ecosystem properties and the interactions among drivers and properties. Ecosystem properties are attributes of ecosystems and their component biota that result from assembly processes^13^. They include aggregate ecosystem functions (productivity, stocks and fluxes), ecological processes (for example, trophic networks), structural features (for example, 3D spatial structure and diversity) and species-level traits of characteristic organisms (for example, ecophysiology, life histories and morphology).

Our model postulates five groups of ecological drivers that may shape ecosystems by acting both as assembly filters and evolutionary pressures (Fig. 1 and Supplementary Information, Appendix 2, for details). Filters are biotic and abiotic processes that determine community assembly from a species pool, given initial occupancy or dispersal (based on community assembly theory^13,14^). Evolutionary pressures are agents of selection that influence ecosystem function and constituent species traits, typically over longer time scales, through evolution and extinction within a dynamic species pool^13,15^.

‘Resource drivers’ (Supplementary Information, Appendix 2, page 2) supply water, oxygen, nutrients, carbon and energy, the resources essential for life. The ‘ambient environment’ (Supplementary Information, Appendix 2, page 2) includes environmental features (for example, temperature, pH, salinity) that continually influence the availability of resources or the ability of organisms to acquire them. The model distinguishes these continuous factors from ‘disturbance regimes’ (Supplementary Information, Appendix 2, page 2), which are sequences of discrete events with different intensities and patterns of occurrence (for example, fires, floods, storms and earth mass movement) that destroy living biomass, liberate and redistribute resources, and regulate life-history processes. ‘Biotic interactions’ (Supplementary Information, Appendix 2, page 3) include competition, predation, pathogenicity, mutualisms and facilitation, which operate at local scales but may shape ecosystem properties at landscape and seascape scales (for example, reef-building symbioses). ‘Human activities’ (Supplementary Information, Appendix 2, page 3) are a special class of biotic interaction that influence ecosystem disassembly and reassembly through resource appropriation, physical restructuring, movement of biota, and climate change^16^. These anthropic processes operate largely, but not exclusively, through effects on other drivers. Although our model portrays humans as integral drivers of ecosystem assembly, we separated human activity from other biotic interactions to highlight connections between ecosystems and socio-economic systems that drive anthropogenic change^17^, and the need to assess and mitigate the human impacts on biodiversity and ecosystem functioning.

Interactions may exist among drivers, modulating their effects on ecosystem properties (Fig. 1 and Supplementary Information, Appendix 2, page 4). For example, resource levels may influence ecosystem assembly directly through niche partitioning or indirectly through alteration of biotic interactions^18^. Similarly, feedbacks exist between ecosystem properties and drivers. For example, human land-use intensification initiates changes in ecosystems that, in turn, influence human social structure, markets and consumption patterns, driving changes in resource appropriation and further change in ecosystem properties^17^. Variations on the model template applied to different groups of ecosystems in our typology (Supplementary Information, Appendices 3 and 4, pages 52–186) reflect our hypotheses about how drivers influence ecosystem properties directly, or indirectly through interactions with other drivers. The model posits that ecosystems share convergent ecological processes and functional properties if they are shaped by similar drivers—and conversely, major changes to these drivers (or their interactions) cause disassembly, transformation and ultimately ecosystem collapse, with consequent losses of biodiversity, ecosystem function and services^8^.

Convergences in ecosystem properties are axiomatic to a functionally based ecosystem typology because they underpin robust generalizations and predictions about ecosystem responses to environmental change and management. Convergences in species traits may arise from common evolutionary origins and niche conservatism^19,20^, but similarities in ecological drivers (selection pressures and assembly filters) may also produce functional convergences in independent lineages. These convergences are enablers of a functional classification framework represented in the upper three levels of our typology. Functional constraints may be imposed by the species pool, which is a dynamic outcome of vicariance, dispersal and evolution, depending on ecosystem location and biogeographic history^21^.

Only a few ecological drivers are likely to be important in shaping the key properties of any particular ecosystem^13^, despite the vast array of potential drivers on Earth and the complex interactions among them. This principle was critical to design of assembly models of each ecosystem functional group and for developing a parsimonious global typology (Supplementary Table 1, principle 6).

## Typology structure

Our ecosystem typology, adopted by the IUCN at the 2020 World Conservation Congress^22,23^ has six hierarchical levels, enabling applications at different thematic scales (Methods and Supplementary Fig. 3.1). Three upper levels (Supplementary Table 3.1) differentiate functional groupings and three lower levels (Methods and Supplementary Information, Appendix 3, pages 19 and 20) accommodate differences in biotic composition among functionally convergent ecosystems. The scalable hierarchical structure (Supplementary Table 1.1, principle 4) and the explicit description of properties and drivers enables units at any thematic level to be mapped at different spatial scales. These units may be tracked through different temporal scales according to needs of specific applications and constraints arising from the resolution of available data.

**Fig. 2:**
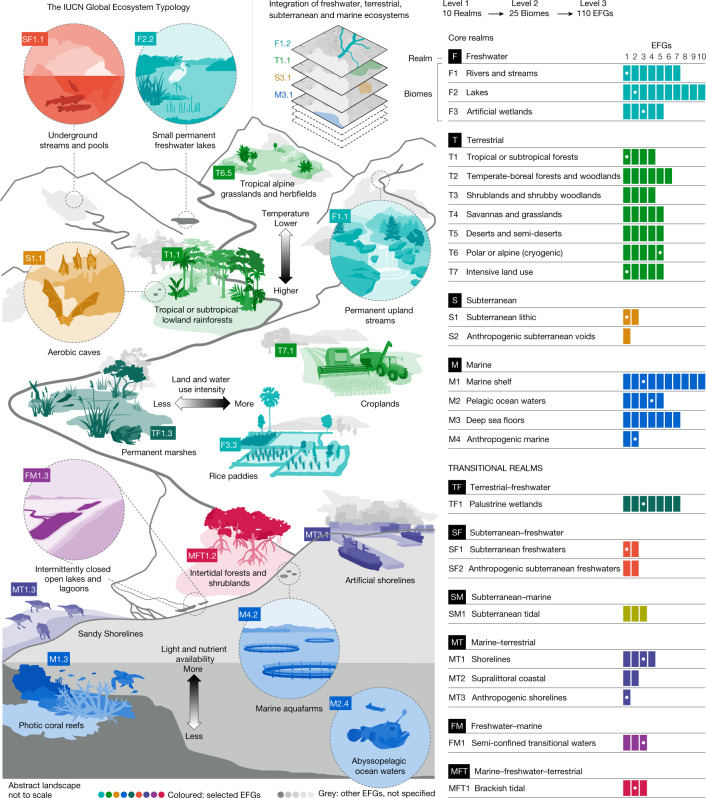
Landscape and seascape relationships of ecosystem functional groups. Left, a sample of ecosystem functional groups (EFGs) from the Global Ecosystem Typology distributed across a hypothetical tropical landscape and seascape. Right, the total number of ecosystem functional groups (coloured boxes) within each realm and functional biome listed (the ecosystem functional groups illustrated on the left are represented by white dots). Multidimensional environmental gradients—three examples are shown: temperature, intensity of human use and light and nutrient availability—influence the strength and spatial expression of ecological drivers (resources, ambient environment, disturbance regimes, biotic interactions and human activity) across landscapes and seascapes, and therefore the spatial relationships of ecosystem types.

**Fig. 3:**
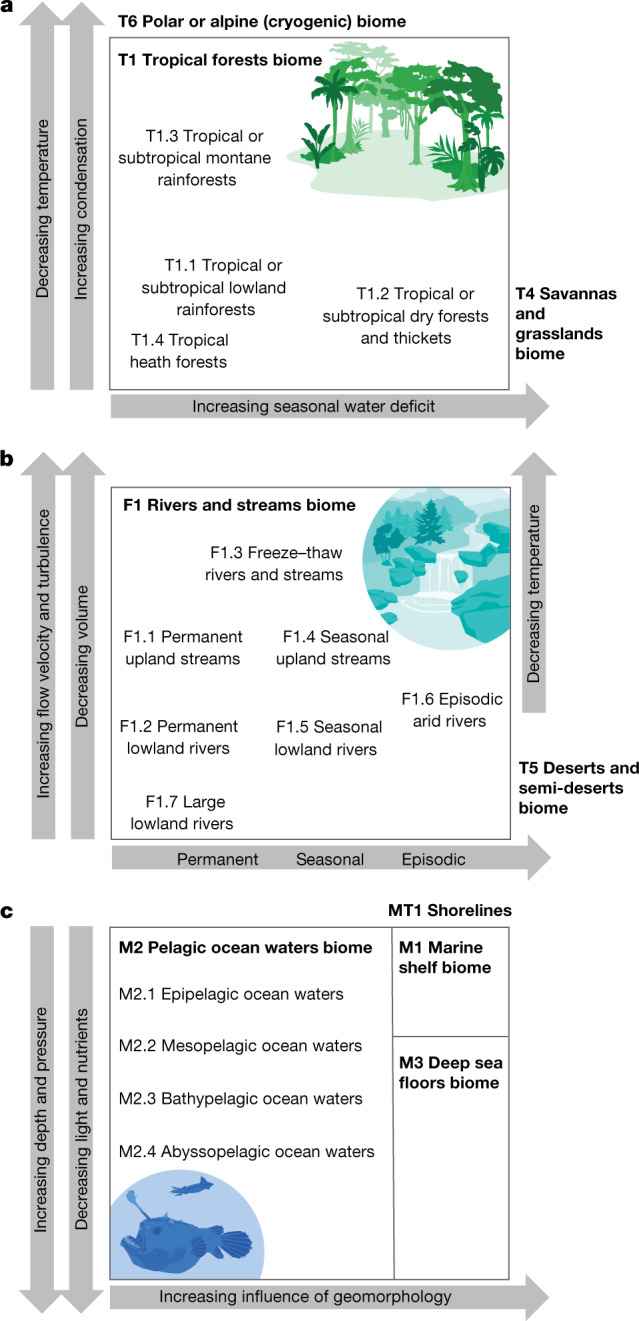
Hypothesized relationships of functional groups differentiated along gradients of selected assembly filters. **a**, The Tropical forests biome (T1), with temperature, elevation and water availability gradients. **b**, The Rivers and streams biome (F1), with stream gradient and temporal flow pattern. **c**, The Marine pelagic biome (M2), with depth and current gradients. In **a**, a third filter related to an edaphic environmental gradient differentiates group T1.4 from T1.1, but is not shown here (see Supplementary Information, Appendix 4, for details on the respective functional groups).

Level 3 units of the typology (ecosystem functional groups, described in Supplementary Information, Appendix 4, pages 52–186 and summarized in Extended Data Tables 1–4) are fundamental to generalizations and predictions about ecosystems with similar functional properties, and therefore have key roles in global synthesis and knowledge transfer for ecosystems. Their distribution across landscapes and seascapes (Fig. 2) is governed by the expression of ecological drivers along temporally variable multidimensional gradients^24,25^ (Fig. 3). Interactions between the drivers that operate at different spatial scales in this multidimensional space determine the dominant filters and evolutionary pressures that shape ecosystem properties in different parts of the biosphere (see Methods, ‘Hierarchical levels’ and Supplementary Information, Appendix 3 for key drivers that differentiate ecosystem functional groups along landscape and seascape gradients visualized in Figs. 2 and 3).

## Applications for ecosystem management

Decisions about effective action to conserve biodiversity and sustain ecosystem services require evidence of which ecosystems are most exposed to risks of collapse^6^ and which ecosystems contribute most to particular human benefits^5^. These analyses are conspicuously lacking in global ecosystem assessments^11,12,26^, but the IUCN Global Ecosystem Typology and a rapidly growing body of spatial data^27^ have established an ecologically robust and powerful capability, and signal a growing readiness for such syntheses.

The IUCN Global Ecosystem Typology facilitates integrated assessment of Earth’s ecosystems, enabling a more powerful and complete evaluation of progress towards biodiversity targets and sustainable development goals than previously possible. This fills a significant gap, exemplified by the limited range of ecosystems assessed in the Convention on Biological Diversity (CBD) Global Biodiversity Outlook 5^26^ and the IPBES Global Assessment^12^. It will also strengthen the evidence base for setting science- and knowledge-based specific, measurable, ambitious, realistic and time-bound (SMART) biodiversity targets in the forthcoming post-2020 CBD global biodiversity framework and for reviewing progress towards them^2^. The United Nations Statistical Commission recently adopted the IUCN typology as a reference classification for extending the System of Environmental Economic Accounting (SEEA) framework to Ecosystem Accounts^28^, meeting a long-recognized need for a spatially explicit, functionally based ecosystem typology to underpin natural capital accounting^29^.

**Fig. 4:**
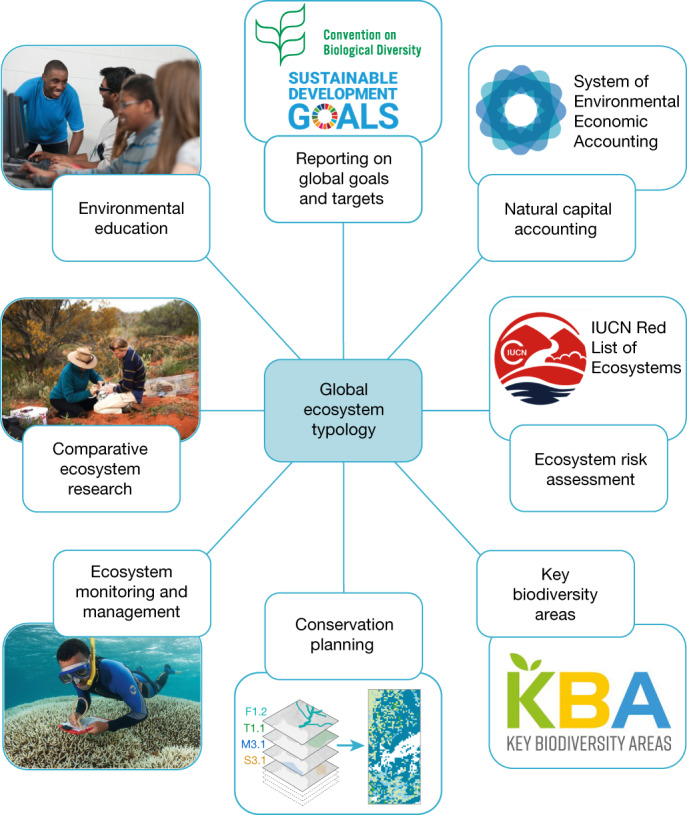
Current and potential applications of the Global Ecosystem Typology to conserve biodiversity and sustain ecosystem services. The typology provides a common ecosystem vocabulary and supports consistent treatment of ecosystems across applications where policy links exist between multiple initiatives. Details are presented in Supplementary Information, Appendix 4. Photo credit: Keith Ellenbogen (Ecosystem monitoring and management); Getty Images (Environmental education); KBA World Database of Key Biodiversity Areas at www.keybiodiversityareas.org; United Nations Sustainable Development Goals at: www.un.org/sustainabledevelopment.

Integrating both functions and biota into the hierarchical structure of the typology confers versatility for diverse applications in ecosystem management and conservation (Fig. 4 and Supplementary Information, Appendix 6). Our typology and developing archive of maps (see caveats in Supplementary Information, Appendix 4) provide a globally consistent framework for advancing the IUCN Red List of Ecosystems^6,30^ and Key Biodiversity Areas^31^, as well as broadly based nature education^32^.

Diagnostic models of ecosystem dynamics, as developed in Red List assessments^30^, with improved ecosystem and threat distribution data, will strengthen capacity to forecast state changes that result in loss of ecosystem function, services and biota. Ecosystem groupings based on convergent drivers, properties and environmental relationships will reveal similarities in threats and mechanisms of degradation, and therefore inform the development of ecosystem-specific management strategies for recovery. Embracing the dynamic nature of ecosystems and its dependency on ecological processes is a key feature that differentiates the IUCN Global Ecosystem Typology from other ecological typologies (Supplementary Table 1.2). This will enable policy and management actions to be targeted towards causes of ecosystem degradation, with knowledge transfer and adaptive learning^33^ about local ecosystems from functionally similar ecosystems elsewhere (Supplementary Fig. 6.1).

## Limitations and the way forward

We expect progressive improvements in future versions of the IUCN Global Ecosystem Typology as knowledge increases. Several aspects of the typology warrant further development to address uncertainties. In particular, models of assembly for each ecosystem functional group represent working hypotheses, for which available empirical evidence varies greatly (Methods, ‘Limitations’). Redressing research biases across different ecosystem types and among different assembly filters will help improve not only the assembly models, but also the distinctions between ecosystem functional groups and units within other levels of the typology.

By highlighting poorly known systems in the atmosphere, deep sea floors, subterranean freshwaters, lithosphere and beneath ice, and by prompting researchers and other users to ask where particular ecosystems belong in the scheme, we foresee the typology promoting research to fill significant knowledge gaps that will improve outcomes of its application and inform future amendments of its structure, as well as descriptions of its units.

Ecosystem mapping is another component of the information base that urgently requires further development, as the currently available indicative global maps for ecosystem functional groups vary substantially in accuracy and precision (Methods, ‘Limitations’). Many uses of the typology (Fig. 3) do not require a full set of comprehensive and globally consistent maps because they are non-spatial (that is, knowledge transfer and framing generalizations), national in scope, or specific to particular ecosystem groups (for example, forests, coral reefs and mangroves). Reliable global maps of suitable resolution, however, are pivotal to the global synthesis of ecosystems, as required for systematic reporting on CBD targets and some other applications^2^.

By decoupling the mapping process from prior development of the classification, our approach liberates the definition of ecosystem units from constraints imposed by the current availability of spatial data and allows for progressive improvement in maps (Supplementary Information, Appendix 4, page 13). New technologies in cloud computing and artificial intelligence, improved global environmental data and deepening time archives of satellite images are paving the way^34,35^. High-resolution maps, some with extended time series, that match the concepts of ecosystem functional groups have been produced for contrasting ecosystem groups such as tidal mudflats^36^ (TM1.2), glacial lakes^37^ (F2.4) and tropical cloud forests^38^ (T1.3) (Supplementary Table 4.1); whereas generic data cubes for forest cover^39^ and surface water^40^ suggest that global high-resolution time-series mapping should be possible for most ecosystem functional groups within the next decade. Future versions of the typology will progressively improve map standards to support applications that depend on spatial analysis. Improved mapping of threats and degradation is similarly required to support ecosystem assessments^41^, particularly in marine environments.

We acknowledge the limitations associated with discrete representation of continuous ecological patterns in nature (Supplementary Information, Appendix 3, page 23). Even though our descriptive framework recognizes core and transitional units, its discrete structure generates boundary and other uncertainties among ecosystems that are ultimately unavoidable, even with extensive description or splitting of classes^42^. However, this fallibility is outweighed by a classificatory approach founded in deep-seated cognitive processes that govern how humans understand and manage environmental, social, economic and cultural dimensions of their conscious universe by dividing it into parts^43^. This will facilitate the widespread uptake of the IUCN typology for effective storage, retrieval and transfer of ecosystem information.

The hierarchical structure of our typology should enable global imperatives to be linked directly with on-ground, nature-based solutions^44^, supporting international mandates for sustainable development and biodiversity conservation. Viewing Earth’s ecosystems through a dynamic functional lens, rather than through largely biogeographic or biophysical ones, will enable a more powerful and direct basis to address the dual goals of conserving biodiversity and sustaining ecosystem services.

## Methods

We developed the IUCN Global Ecosystem Typology in the following sequence of steps: design criteria; hierarchical structure and definition of levels; generic ecosystem assembly model; top-down classification of the upper hierarchical levels; iterative circumscription of the units and ecosystem-specific adaptations of the assembly model; full description of the units; and map compilation. Some iteration proved necessary, as the description and review process sometimes revealed a need for circumscribing additional units.

### Design criteria and other typologies

Under the auspices of the IUCN Commission on Ecosystem Management, we developed six design principles to guide the development of a typology that would meet the needs for global ecosystem reporting, risk assessment, natural capital accounting and ecosystem management: (1) representation of ecological processes and ecosystem functions; (2) representation of biota; (3) conceptual consistency throughout the biosphere; (4) scalable structure; (5) spatially explicit units; and (6) parsimony and utility (see Supplementary Table 1.1 and Supplementary Information, Appendix 1 for definitions and rationale).

We assessed 23 existing ecological classifications with global coverage of terrestrial, freshwater, and/or marine environments against these principles to determine their fitness for IUCN’s purpose (Supplementary Information, Appendix 1). These include general classifications of land, water or bioclimate, as well as classifications of units that conform with the definition of ecosystems adopted in the United Nations Convention on Biological Diversity^45^ or an equivalent definition in the IUCN Red List of Ecosystems^30^. We reviewed documentation on methods of derivation, descriptions of classification units and maps to assess each classification against the six design principles (Supplementary Table 1.2 for details).

### Typology structure and ecosystem assembly

We developed the structure of the Global Ecosystem Typology and the generic ecosystem assembly model at a workshop attended by 48 terrestrial, freshwater and marine ecosystem experts at Kings College London, UK, in May 2017. Participants agreed that a hierarchical structure would provide an effective framework for integrating ecological processes and functional properties (Supplementary Table 1.1, design principle 1), and biotic composition (principle 2) into the typology, while also meeting the requirement for scalability (principle 4). Although neither function nor composition were intended to take primacy within the typology, we reasoned that a hierarchy representing functional features in the upper levels is likely to support generalizations and predictions by leveraging evolutionary convergence^13^. By contrast, a typology reflecting compositional similarities in its upperlevels is less likely to be stable owing to dynamism of species assemblages and evolving knowledge on species taxonomy and distributions. Furthermore, representation of compositional relationships at a global scale would require many more units in upper levels, and possibly more hierarchical levels. Therefore, we concluded that a hierarchical structure recognizing compositional variants at lower levels within broad functionally based groupings at upper levels would be more parsimonious and robust (principle 6) than one representing composition at upper levels and functions at lower levels.

Workshop participants initially agreed that three hierarchical levels for ecosystem function and three levels for biotic composition could be sufficient to represent global variation across the whole biosphere. Participants developed the concepts of these levels into formal definitions (Supplementary Table 3.1), which were reviewed and refined during the development process.

To ensure conceptual consistency of the typology and its units throughout the biosphere (principle 3), we drew from community assembly theory to develop a generic model of ecosystem assembly. The traditional community assembly model incorporates three types of filters (dispersal, the abiotic environment and biotic interactions) that determine which biota from a larger pool of potential colonists can occupy and persist in an area^13^. We extended this model to ecosystems by: (1) defining three groups of abiotic filters (resources, ambient environment and disturbance regimes) and two groups of biotic filters (biotic interactions and human activity); (2) incorporating evolutionary processes that shape characteristic biotic properties of ecosystems over time; (3) defining the outcomes of filtering and evolution in terms of all ecosystem properties including both ecosystem-level functions and species-level traits, rather than only in terms of species traits and composition; and (4) incorporating interactions and feedbacks among filters and selection agents and ecosystem properties to elucidate hypotheses about processes that influence temporal and spatial variability in the properties of ecosystems and their component biota. In community assembly, only a small number of filters are likely to be important in any given habitat^13^. In keeping with this proposition, we used the generic model to identify biological and physical features that distinguish functionally different groups of ecosystems from one another by focusing on different ecological drivers that come to the fore in structuring their assembly and shaping their properties.

### Hierarchical levels

The top level of classification (Fig. 2 and Extended Data Tables 1–4) defines five core realms of the biosphere based on contrasting media that reflect ecological processes and functional properties: terrestrial; freshwaters and inland saline waters (hereafter freshwater); marine; subterranean; and atmospheric. Biome gradient concepts^25^ highlight continuous variation in ecosystem properties, which is represented in the typology by transitional realms that mark the interfaces between the five core realms (for example, floodplains (terrestrial–freshwater), estuaries (freshwater–marine), and so on). In Supplementary Information, Appendix 3 (pages 3–16) and Supplementary Table 3.1, we describe the five core realms and review the hypothesized assembly filters and ecosystem properties that distinguish different groups within them. The atmospheric realm is included for comprehensive coverage, but we deferred resolution of its lower levels because its biota is poorly understood, sparse, itinerant and represented mainly by dispersive life stages^46^.

Functional biomes (level 2) are components of the biosphere united by one or more major assembly processes that shape key ecosystem functions and ecological processes, irrespective of taxonomic identity (Supplementary Information, Appendix 3, page 17). Our interpretation aligns broadly with ‘functional biomes’ described elsewhere^24,25,47^, extended here to reflect dominant assembly filters and processes across all realms, rather than the more restricted basis of climate-vegetation relationships that traditionally underpin biome definition on land. Hence, the 25 functional biomes (Supplementary Information, Appendix 4, pages 52–186 and https://global-ecosystems.org/) include some ‘traditional’ terrestrial biomes^47^, as well as lentic and lotic freshwater systems, pelagic and benthic marine systems, and anthropogenic functional biomes assembled and usually maintained by human activity^48^.

Level 3 of the typology defines 110 ecosystem functional groups described with illustrated profiles in Supplementary Information, Appendix 4 (pages 52–186) and at https://global-ecosystems.org/. These are key units for generalization and prediction, because they include ecosystem types with convergent ecosystem properties shaped by the dominance of a common set of drivers (Supplementary Information, Appendix 3, pages 17–19). Ecosystem functional groups are differentiated along environmental gradients that define spatial and temporal variation in ecological drivers (Figs. 2 and 3 and Supplementary Figs. 3.2 and 3.4). For example, depth gradients of light and nutrients differentiate functional groups in pelagic ocean waters (Fig. 3c and Extended Data Table 4), influencing assembly directly and indirectly through predation. Resource gradients defined by flow regimes (influenced by catchment precipitation and evapotranspiration) and water chemistry, modulated by environmental gradients in temperature and geomorphology, differentiate functional groups of freshwater ecosystems^25^ (Fig. 3b and Extended Data Table 3). Terrestrial functional groups are distinguished primarily by gradients in water and nutrient availability and by temperature and seasonality (Fig. 3a and Extended Data Table 1), which mediate uptake of those resources and regulate competitive dominance and productivity of autotrophs. Disturbance regimes, notably fire, are important global drivers in assembly of some terrestrial ecosystem functional groups^49^.

Three lower levels of the typology distinguish functionally similar ecosystems based on biotic composition. Our focus in this paper is on global functional relationships of ecosystems represented in the upper three levels of the typology, but the lower levels (Supplementary Information, Appendix 3, pages 19 and 20) are crucial for representing the biota in the typology, and facilitate the scaling up of information from established local-scale typologies that support decisions where most conservation action takes place. These lower levels are being developed progressively through two contrasting approaches with different trade-offs, strengths and weaknesses. First, level 4 units (regional ecosystem subgroups) are ecoregional expressions of ecosystem functional groups developed from the top-down by subdivisions based on biogeographic boundaries (for example, in ref. ^50^) that serve as simple and accessible proxies for biodiversity patterns^51^. Second, level 5 units (global ecosystem types) are also regional expressions of ecosystem functional groups, but unlike level 4 units they are explicitly linked to local information sources by bottom-up aggregation^52^ and rationalization of level 6 units from established subglobal ecological classifications. Subglobal classifications, such as those for different countries (see examples for Chile and Myanmar in Supplementary Tables 3.3 and 3.4), are often developed independently of one another, and thus may involve inconsistencies in methods and thematic resolution of units (that is, broadly defined or finely split). Aggregation of level 6 units to broader units at level 5 based on compositional resemblance is necessary to address inconsistencies among different subglobal classifications and produce compositionally distinctive units suitable for global or regional synthesis.

Integrating local classifications into the global typology, rather than replacing them, exploits considerable efforts and investments to produce existing classifications, already developed with local expertise, accuracy and precision. By placing national and regional ecosystems into a global context, this integration also promotes local ownership of information to support local action and decisions, which are critical to ecosystem conservation and management outcomes (Supplementary Information, Appendix 3, page 20). These benefits of bottom-up approaches come at the cost of inevitable inconsistencies among independently developed classifications from different regions, a limitation avoided in the top-down approach applied to level 4.

### Circumscribing upper-level units

We formed specialist working groups (terrestrial/subterranean, freshwater and marine) to develop descriptions of the units within the upper levels of the hierarchy, subdividing realms into functional biomes, and biomes into ecosystem functional groups. We used definitions of the hierarchical levels (Supplementary Table 3.1) and the conceptual model of ecosystem assembly (Fig. 1) to maintain consistency in defining the units at each level during iterative discussions within and between the working groups.

Working groups agreed on preliminary lists of functional biomes and ecosystem functional groups by considering variation in major drivers along ecological gradients (Figs. 2 and 3 and Supplementary Figs. 3.2 and 3.4) based on published literature, direct experience and expertise of working group members, and consultation with colleagues in their respective research networks. After the workshop, working groups sought recent global reviews of the candidate units and recent case studies of exemplars to shape descriptions of the major groups of ecosystem drivers and properties for each unit. Circumscriptions and descriptions of the units were reviewed and revised iteratively to ensure clear distinctions among units, with a total of 206 reviews of descriptive profiles undertaken by 60 specialists, a mean of 2.4 reviews per profile (Supplementary Table 5.1). The working groups concurrently adapted the generic model of ecosystem assembly (Fig. 1) to represent working hypotheses on salient drivers and ecosystem properties for each ecosystem functional group.

### Incorporating human influence

Very few of the ecological typologies reviewed in Supplementary Information, Appendix 1 integrate anthropogenic ecosystems in their classificatory frameworks. Anthropogenic influences create challenges for ecosystem classification, as they may modify defining features of ecosystems to a degree that varies from negligible to major transformation across different locations and times. We addressed this problem by distinguishing transformative outcomes of human activity at levels 2 and 3 of the typology from lesser human influences that may be represented either at levels 5 and 6, or through measurements of ecosystem integrity or condition that reflect divergence from reference states arising from human activity.

Anthropogenic ecosystems grouped within levels 2 and 3 were thus defined as those created and sustained by intensive human activities, or arising from extensive modification of natural ecosystems such that they function very differently. These activities are ultimately driven by socio-economic and cultural-spiritual processes that operate across local to global scales of human organization. In many agricultural and aquacultural systems and some others, cessation of those activities may lead to transformation into ecosystem types with qualitatively different properties and organizational processes (see refs. ^53^^,^^54^ for cropland and urban examples, respectively). Indices such as human appropriation of net primary productivity^55^, combined with land-use maps^56^, offer useful insights into the distribution of some anthropogenic ecosystems, but further development of indices is needed to adequately represent others, particularly in marine, and freshwater environments. Beyond land-use classification and mapping approaches (Supplementary Information, Appendix 1, page 6), a more comprehensive elaboration of the intensity of human influence underpinning the diverse range of anthropogenic ecosystems requires a multidimensional framework incorporating land-use inputs, outputs, their interactions, legacies of earlier activity and changes in system properties^17^.

Where less intense human activities occur within non-anthropogenic ecosystem types, we focused descriptions on low-impact reference states. Therefore, human activities are not shown as drivers in the assembly models for non-anthropogenic ecosystem groups, even though they may have important influences on the contemporary ecosystem distribution. This approach enables the degree and nature of human influence to be described and measured against these reference states using assessment methods such as the Red List of Ecosystems protocol^30^, with appropriate data on ecosystem change.

### Indicative distribution maps

Finally, to produce spatially explicit representations of the units at level 3 of the typology (principle 5), we sought published global maps (sources in Supplementary Table 4.1) that were congruent with the concepts of respective ecosystem functional groups. Where several candidate maps were available, we selected maps with the closest conceptual alignment, finest spatial resolution, global coverage, most recent data and longest time series. The purpose of maps for our study was to visualize global distributions. Prior to applications of map data to spatial analysis, we recommend critical review of methods and validation outcomes reported in each data source to ensure fitness for purpose (Supplementary Information, Appendix 4).

Extensive searches of published literature and data archives identified high-quality datasets for some ecosystem functional groups (for example, T1.3 Tropical–subtropical montane rainforests; MT1.4 Muddy shorelines; M1.5 Sea ice) and datasets that met some of these requirements for a number of other ecosystem functional groups (see Supplementary Table 4.1 for details). Where evaluations by authors or reviewers identified limitations in available maps, we used global environmental data layers and biogeographic regionalizations as masks to adjust source maps and improve their congruence to the concept of the relevant functional group (for example, F1.2 Permanent lowland rivers). For ecosystem functional groups with no specific global mapping, we used ecoregions^50,57,58^ as biogeographic templates to identify broad areas of occurrence. We consulted ecoregion descriptions, global and regional reviews, national and regional ecosystem maps, and applied in situ knowledge of participating experts to identify ecoregions that contain occurrences of the relevant ecosystem functional group (for example, T4.4 Temperate woodlands) (see Supplementary Table 4.1 for details). We mapped ecosystem functional groups as major occurrences where they dominated a landscape or seascape matrix and minor occurrences where they were present, but not dominant in landscape–seascape mosaics, or where dominance was uncertain. Although these two categories in combination communicate more information about ecosystem distribution than binary maps, simple spatial overlays using minor occurrences are likely to inflate spatial statistics. The maps are progressively upgraded in new versions of the typology as explicit spatial models are developed and new data sources become available (see ref. ^27^ for a current archive of spatial data).

The classification and descriptive profiles, including maps, for each functional biome and ecosystem functional group underwent extensive consultation, and targeted peer review and revision through a series of four phases described in Supplementary Information, Appendix 5 (pages 2–4). The reviewer comments and revisions from targeted peer review are documented in Supplementary Table 5.1. In all, more than 100 ecosystem specialists have contributed to the development of v2.1 of the typology.

### Limitations

Uneven knowledge of Earth’s biosphere has constrained the delimitation and description of units within the typology. There is a considerable research bias across the full range of Earth’s ecosystems, with few formal research studies evaluating the relative influence of different ecosystem drivers in many of the functional groups, and abiotic assembly filters generally receiving more attention than biotic and dispersal filters. This poses challenges for developing standardized models of assembly for each ecosystem functional group. The models therefore represent working hypotheses, for which available evidence varies from large bodies of published empirical evidence to informal knowledge of ecosystem experts and their extensive research networks. Large numbers of empirical studies exist for some forest functional groups, savannas, temperate heathlands in Mediterranean-type climates, coral reefs, rocky shores, kelp forests, trophic webs in pelagic waters, small permanent freshwater lakes, and others (see references in the respective profiles (Supplementary Information, Appendix 4)). For example, Bond^49^ reviewed empirical and modelling evidence on the assembly and function of tropical savannas that make up three ecosystem functional groups, showing that they have a large global biophysical envelope that overlaps with tropical dry forests, and that their distribution and dynamics within that envelope is strongly influenced by top-down regulation via biotic filters (large herbivores and their predators) and recurrent disturbance regimes (fires). Despite the development of this critical knowledge base, savannas suffer from an awareness disparity that hinders effective conservation and management^59^. In other ecosystems, our assembly models rely more heavily on inferences and generalizations of experts drawn from related ecosystems, are more sensitive to interpretations of participating experts, and await empirical testing and adjustment as understanding improves. Empirical tests could examine hypothesized variation in ecosystem properties along gradients within and between ecosystem functional groups and should return incremental improvements on group delineation and description of assembly processes.

High-quality maps at suitable resolution are not yet available for the full set of ecosystem functional groups, which limits current readiness for global analysis. The maps most fit for global synthesis are based on remote sensing and environmental predictors that align closely to the concept of their ecosystem functional group, incorporate spatially explicit ground observations and have low rates of omission and commission errors, ‘high’ spatial resolution (that is, rasters of 1 km^2^ (30 arcsec) or better), and time series of changes. Sixty of the maps currently in our archive^27^ aligned directly or mostly with the concept of their corresponding ecosystem functional group, while the remainder were based on indirect spatial proxies, and most were derived from polygon data or rasters of 30 arcsec or finer (Supplementary Table 4.1). Maps for 81 functional groups were based either on known records, or on spatial data validated by quantitative assessments of accuracy or efficacy. Therefore, we suggest that maps currently available for 60–80 of the 110 functional groups are potentially suitable for global spatial analysis of ecosystem distributions. Although, a significant advance on broad proxies such as ecoregions, the maps currently available for ecosystem functional groups would benefit from expanded application of recent advances in remote sensing, environmental datasets, spatial modelling and cloud computing to redress inequalities in reliability and resolution. The most urgent priorities for this work are those identified in Supplementary Table 4.1 as relying on indirect proxies for alignment to concept, qualitative evaluation by experts and coarse resolution (>1 km^2^) spatial data.

### Reporting summary

Further information on research design is available in the Nature Research Reporting Summary linked to this article.

## Online content

Any methods, additional references, Nature Research reporting summaries, source data, extended data, supplementary information, acknowledgements, peer review information; details of author contributions and competing interests; and statements of data and code availability are available at 10.1038/s41586-022-05318-4.

### Supplementary information

Supplementary Information This file contains a glossary of technical terms. The glossary provides definitions for specialist terms used in descriptive profiles of ecosystems in Supplementary Information, Appendix 4. It also defines terms used in the main text.

Reporting Summary

Peer Review File

Appendix S1 Review of existing ecological typologies. Descriptions and justifications of six principles that a global ecosystem typology should meet to support planning and management decisions for biodiversity conservation and sustain ecosystem services. We use the principles to frame a tabular comparison of 23 existing ecological classifications of global extent and conclude that none meet all six principles.

Appendix S2 Conceptual foundations for the IUCN Global Ecosystem Typology: a generic model of ecosystem assembly. Development of a conceptual model of ecosystem assembly to frame descriptions of functionally contrasting ecosystems by adapting key elements of community assembly theory (species pools, assembly filters and species traits). We describe the roles of biotic and biotic drivers, dispersal processes and evolutionary legacies and their interactions in shaping ecosystem properties.

Appendix S3 Structure of the IUCN Global Ecosystem Typology. A description of the structure of the typology and its rationale. Three upper hierarchical levels distinguish functionally contrasting units and three lower levels distinguish compositionally different units with similar functional properties. We contrast postulated assembly filters and ecosystem properties from our assembly model (S2) across ecosystems within the five realms of the biosphere.

Appendix S4 The IUCN Global Ecosystem Typology v2.1: Descriptive profiles for functional biomes and ecosystem functional groups. Descriptions and explanatory notes for 25 functional biomes and 110 ecosystem functional groups in v2.1 of the typology. Descriptions include text, images, diagrammatic assembly models, indicative global maps and key references. Explanatory notes include keys to the diagrammatic models and map assembly methods and data sources.

Appendix S5 Development, review and revision process for the IUCN Global Ecosystem Typology. A full description of the review peer review processes undertaken during typology development. Tabulation of all reviewer comments and detailed responses of authors for all 110 ecosystem functional groups in level 3 of the typology.

Appendix S6 Applications of the IUCN Global Ecosystem Typology. A review of the role of the typology in applications including reporting on global conservation and sustainability goals and targets, natural capital accounting, ecosystem risk assessment, identification of key biodiversity areas, conservation planning ecosystem monitoring, comparative ecosystem research and environmental education.

## Figures and Tables

**Extended Data Table 1:**
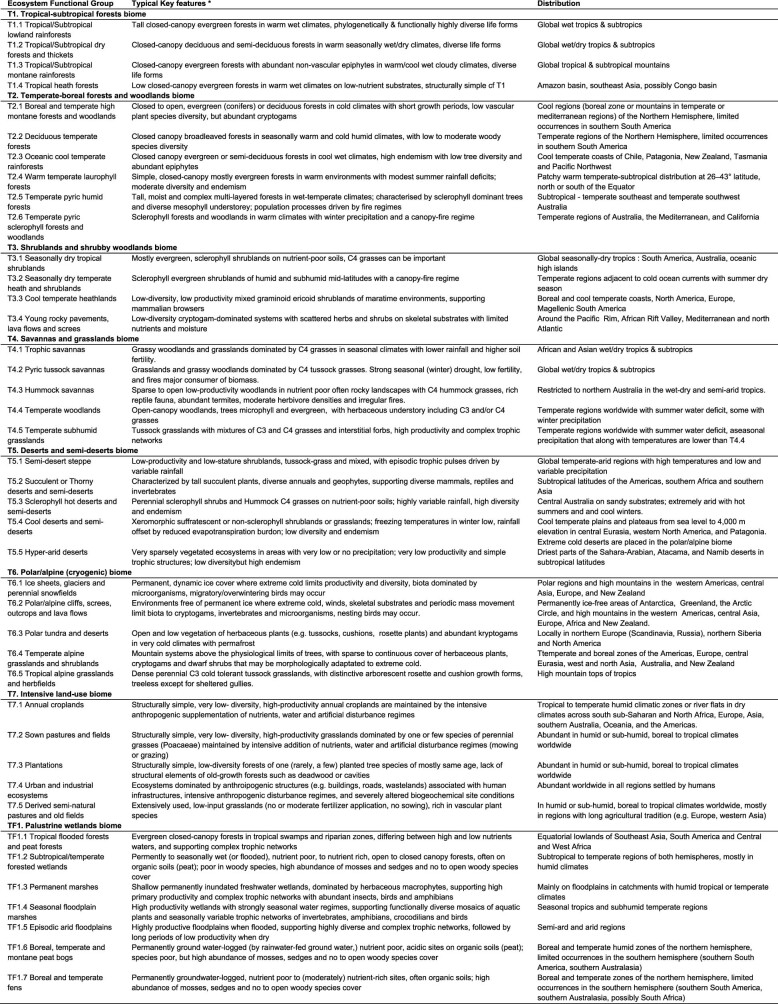
Key features of Ecosystem Functional Groups In The Terrestrial Realm And The Terrestrial-freshwater transitional realm of the IUCN Global Ecosystem Typology v2.1

See Appendix S3 for further details of typology structure and Appendix S4 for descriptions of functional biomes (Level 2) and Ecosystem Functional Groups (Level 3).

**Extended Data Table 2:**
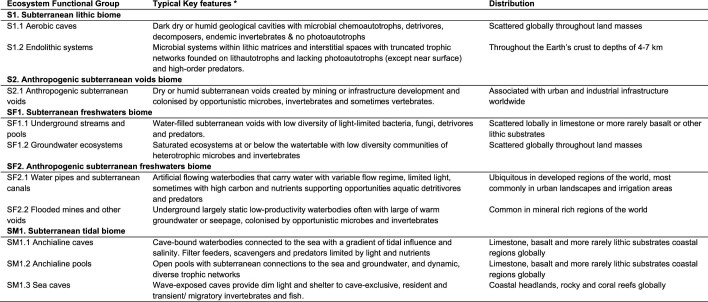
Key features of Ecosystem Functional Groups in the Subterranean realm, Subterranean-Freshwater transitional realm and Subterranean-Marine transitional realm of the IUCN Global Ecosystem Typology v2.1

See Appendix S3 for further details of typology structure and Appendix S4 for descriptions of functional biomes (Level 2) and Ecosystem Functional Groups (Level 3).

**Extended Data Table 3:**
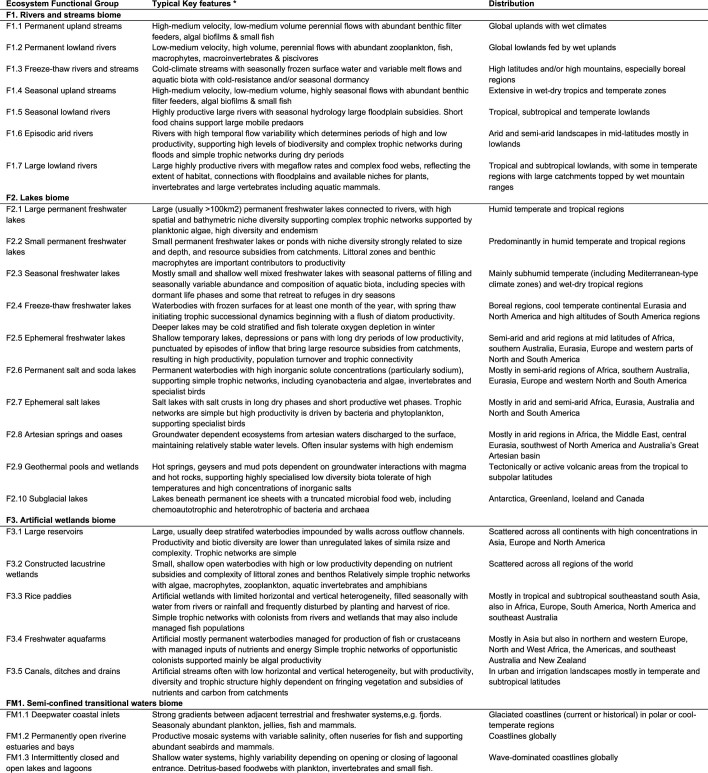
Key features of Ecosystem Functional Groups in the Freshwater realm and Freshwater-Marine transitional realm of the IUCN Global Ecosystem Typology v2.1

See Appendix S3 for further details of typology structure and Appendix S4 for descriptions of functional biomes (Level 2) and Ecosystem Functional Groups (Level 3).

**Extended Data Table 4:**
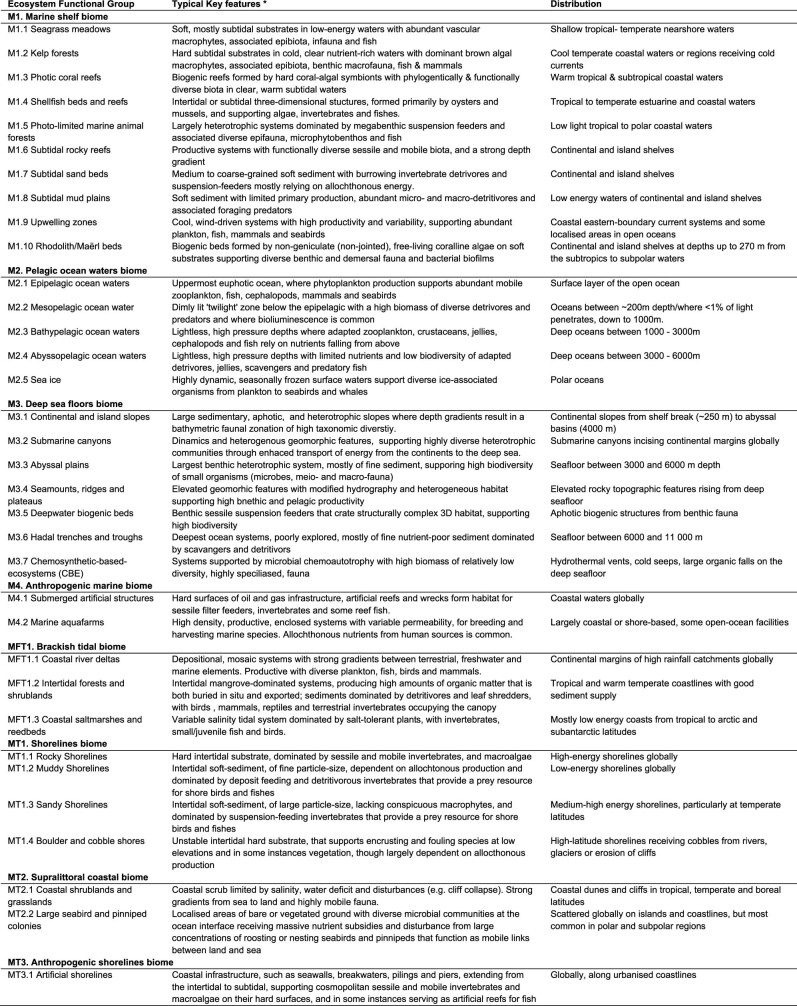
Key features of Ecosystem Functional Groups in the Marine realm, Marine-Terrestrial transitional realm and Marine-Freshwater-Terrestrial transitional realm of the IUCN Global Ecosystem Typology v2.1

See Appendix S3 for further details of typology structure and Appendix S4 for descriptions of functional biomes (Level 2) and Ecosystem Functional Groups (Level 3).

## Data Availability

Descriptions, images and interactive maps for the typology are updated periodically at https://global-ecosystems.org/. The spatial data for this study are available at Zenodo (10.5281/zenodo.3546513).
