# Supplementary material for: A function-based typology for Earth’s ecosystems
Source: Nature. 2022 Oct 12;610(7932):513–8. doi: 10.1038/s41586-022-05318-4 (PMC9581774; doi:10.1038/s41586-022-05318-4)
Supplement: Supplementary file 1 — This file contains a glossary of technical terms. The glossary provides definitions for specialist terms used in descriptive profiles of ecosystems in Supplementary Information, Appendix 4. It also defines terms used in the main text. [file 41586_2022_5318_MOESM1_ESM.pdf]

nature portfolio

https://doi.org/10.1038/s41586-022-05318-4

Supplementary information

A function-based typology for Earth's ecosystems

In the format provided by the
authors and unedited

Glossary of technical terms

'*A function-based typology for Earth's ecosystems*'

David A. Keith, Jose R. Ferrer-Paris, Emily Nicholson, Melanie J. Bishop, Beth A. Polidoro, Eva Ramirez-Llodra, Mark G. Tozer, Jeanne L. Nel, Ralph Mac Nally, Edward J. Gregr, Kate E. Watermeyer, Franz Essl, Don Faber-Langendoen, Janet Franklin, Caroline E. R. Lehmann, Andres Etter, Dirk J. Roux, Jonathan S. Stark, Jessica A. Rowland, Neil A. Brummitt, Ulla C. Fernandez-Arcaya, Iain M. Suthers, Susan K. Wiser, Ian Donohue, Leland J. Jackson, R. Toby Pennington, Thomas M. Iliffe, Vasilis Gerovasileiou, Paul Giller, Belinda J. Robson, Nathalie Pettorelli, Angela Andrade, Arild Lindgaard, Teemu Tahvanainen, Aleks Terauds, Michael A Chadwick, Nicholas J. Murray, Justin Moat, Patricio Pliscoff, Irene Zager, Richard T. Kingsford

Nature 2022

Accurate description of ecosystems sometimes requires specialist technical terminology. The following definitions are provided for specialist terms used in descriptive profiles presented in Appendix S4. The glossary also includes definitions for terms used in the main text.

**Term**	**Definition**
Allochthonous energy	Energy imported into an ecosystem from external sources in the form of organic material.
Ambient environment	Non-resource environmental factors that modify the availability of resources or the ability of organisms to acquire them.
Anthropogenic ecosystems	Ecosystem types created and sustained by intensive human activities. For some of these systems, cessation of those activities may lead to transformation into ecosystem types with different properties and organisational processes.
Aphotic	A zone where light intensity is too low to support photosynthesis.
Assembly processes	The combined action of the abiotic, biotic and dispersal filters (see Fig. 1).
Autochthonous energy	Energy captured from abiotic sources *in situ* by autotrophs living within an ecosystem.
Autotroph	An organism that fixes carbon from its surroundings, manufacturing complex energy-storing organic compounds, generally using energy from light (photosynthesis) or inorganic chemical reactions (chemosynthesis). Autotrophs are primary producers in trophic webs.

Term	Definition
Basin fill	Unconsolidated to moderately consolidated subterranean sediments that bear aquifers. They are composed of gravel, sand, silt, and clay deposited on antecedent alluvial fans, pediments, flood plains, and playas.
Biofilm	Periphyton. A complex layer composed of algae, cyanobacteria and heterotrophic microbes embedded in a mucopolysaccharide matrix cohering to submerged aquatic surfaces. Important food source for aquatic animals.
Biogenic	A structure created by living organisms (e.g. a coral reef, tunnels in soils or sediment).
Biophysical attributes	Spatial representations of environmental variables (e.g. maps of climate variables, topography, bathymetry, substrate, etc.) that are assumed to represent the dimensions of species' environmental niches. They are used to classify the earth's surface into spatial units that represent the distributions of different assemblages of biota (see Table S1.1 for examples of biophysical classifications). Some biophysical attributes may be correlated with ecosystem stocks and fluxes.
Bottom-up regulation	Describing a strong influence of primary producers and resource availability on ecosystem structure (also known as 'green world')
C_3	The most common photosynthetic pathway in plants based only on the Calvin cycle with associated energy loss to photorespiration and dependence on daytime CO_2 uptake. This pathway is dominant in environments with abundant moisture and cool temperatures.
Cauliflory	An arrangement of flowers and fruits in which they are borne directly on the main stems of a tree.
C_4	A photosynthetic pathway with a supplementary C-fixation pathway that minimises photorespiration, reduces CO_2 demand and increases water use efficiency, often dominating in warm and dry environments.
CAM	A specialised C_4 photosynthetic pathway in which CO_2 uptake and fixation occur during the night, followed by internal release in daytime when light-dependent photosynthesis can take place. Stomatal closure occurs during the day, reducing moisture loss and enabling survival in very hot and dry conditions.
Chemoautotroph	An organism that fixes carbon from its surroundings using energy from inorganic chemical reactions.
Convergent functional traits	Properties of species that perform similar functional roles in different ecosystems even though they are descendants from independent or semi-independent lineages (e.g. large consumers of biomass, conservation of water

Term	Definition
	in arid climates, tolerance of low temepratures, etc.); outcomes of converegent evolution, given similar selection pressures.
Cryogenic	Ecosystems that develop and function under low temperatures.
C:N ratio	Carbon-to-nitrogen ratio in biological tissues. Reflects differences in tissue composition related to nitrogen availability and capture as well as woodiness in plants.
Dimicitic lakes	Lakes with waters that mix from top to bottom twice per year, before and after surface freezing in winter.
Disassembly	A process in a trajectory towards ecosystem collapse, whereby defining features of an ecosystem decline, interact more weakly or in different ways with other ecosystem components, and may completely cease or disappear (see reassembly)
Disturbances	Sequences or 'regimes' of environmental events with different intensities and patterns of occurrence that destroy living biomass, liberate and redistribute resources and trigger life history processes in some organisms (e.g. fires, floods, storms, mass movement)
Dystrophic	Waters with low levels of dissolved nutrients, high acidity, brown colouration and low light penetration due to tannins, organic acids and undecayed plant matter, usually originating from peaty substrates
Ecological processes	Activities that result from interactions among organisms and between organisms and their environment (after Pettorelli et al. 2018).
Ecosystem properties	Defining attributes of ecosystems and their component biota that result from assembly processes (i.e. properties are outcomes of filtering). They include aggregate ecosystem functions (stocks and fluxes), ecological processes (e.g. trophic networks), structural features (e.g. 3-D spatial structure, diversity), and species-level traits of characteristic organisms that influence their performance (e.g. fitness).
Ecosystem functions	Attributes related to the performance of an ecosystem that are the consequence of one or multiple ecosystem processes, and that directly and/or indirectly benefit a range of species, including humans (after Pettorelli et al. 2018). Examples of ecosystem functions include biomass production, regulation of fluxes between stocks of resources, organisation of habitat structure, etc.
Emergent	A large tree, emerging above the height of a main forest canopy.

Term	Definition
Engineer	Ecological or ecosystem engineers are organisms that directly or indirectly alter the biotic or abiotic structure of ecosystems and resource availability, making it suitable for habitation by other organisms (Jones et al., 1994)
Epicormic resprouting	New shoots on trees emerging from meristematic tissues beneath the bark on large stems and trunks, usually after death of canopy foliage.
Ericoid leaves	Small, sclerophyllous leaves with thick cuticles and typically crowded on the branchlets; resembling those of heather.
Euphotic	A zone with abundant light that can support photosynthesis.
Filter (assembly filter)	A limiting process imposed by the physical environment (abiotic filter, niche constraint), or by interactions with organisms (biotic filter, e.g. competition, predation, disease), or by barriers to arrival (dispersal filter cf. prior occupancy), that organises and maintains the assembly of ecosystem components.
Functionally similar ecosystems	Ecosystems having similar operating mechanisms, drivers of change and sensitivities to environmental change and management. For example, grassy ecosystems (Biome T4) have similar mechanisms of responses to changes in their key drivers (herbivory, fire regimes), rivers and streams (Biome F1) have mechanistically similar responses to changes in catchment rainfall or pollution, cryogenic ecosystems (Biome T6) have mechanistically similar responses to temperature change, etc.
Heterotroph	An organism that derives its intake of nutrition from extant sources of organic carbon, mainly plant or animal matter. In the food chain, heterotrophs are secondary and tertiary consumers. Heterotrophs are consumers in trophic webs, including decomposers, detritivores, herbivores and predators.
Igneous activity	The extrusion of hot molten rock or magma onto the earth's surface or sea floor from volcanoes or vents, or its intrusion into other geological formations below ground.
LAI	Leaf Area Index, the projected area of leaves as a proportion of the area of land compared to which it is measured. Useful in remote sensing for describing vegetation density.
Leaf sizes	Terms describing leaf size follow Raunkiaer (1934) except 'Notophyll'.

Size class	Leaf area
Megaphyll	>164,025 mm^2
Macrophyll	18,225–164,025 mm^2
Mesophyll	2,025–18,225 mm^2
[Notophyll	2,025–4,500 mm^2]

Term	Definition
	Microphyll 225–2,025 mm^2<br>Nanophyll 25–225 mm^2<br>Leptophyll <25 mm^2
Kinetic energy	A property of aquatic ecosystems describing the motion of water in terms of velocity and mass. It influences the assembly of aquatic systems through movement and supply of resources (oxygen, nutrients, organic particles containing energy and carbon) and other components of the ambient environment (e.g. substrate stability), particularly for ecosystems in the marine shelf (M1), shorelines (MT1) and rivers and streams (F1) biomes.
Mass movement	Bulk movements of soil or rock debris down slope or vertically downwards in response to gravity.
Mesophotic	A zone of moderate light intensity that can support photosynthesis.
Meromictic lakes	Lakes with waters that rarely mix from top to bottom, and thus remaining semi-permanently stratified into stable layers with contrasting temperature and hydrochemistry and biota.
Monomictic lakes	Lakes with waters that mix from top to bottom once per year, regardless of whether the surface freezes in winter, although the seasonal timing of mixing depends on whether surface freezing occurs.
Peat	A deposit of partially decayed organic matter in the upper soil horizons.
Periphyton	Biofilm. A complex layer composed of algae, cyanobacteria and heterotrophic microbes embedded in a mucopolysaccharide matrix cohering to submerged aquatic surfaces. Important food source for aquatic animals.
Photoautotroph	An organism that fixes carbon from its surroundings using energy from light.
Phreatic	Related to groundwater or aquifers.
Physical restructuring	A transformational change to the vertical or horizontal arrangement of living (biogenic) or non-living ecosystem components.
Polymicitic lakes	Lakes with waters that mix continuously from top to bottom, and thus are never vertically stratified, usually due to their shallow depth.
Primary productivity*	The amount of chemical energy (expressed as carbon biomass) that autotrophs create in a given length of time.
Resources	Five fundamental resources in the environment that are essential to sustaining all life: water, nutrients, oxygen, carbon and energy

Term	Definition
Resource appropriation	The diversion or removal of resources from an ecosystem for human use (e.g. water diversion from rivers to irrigation systems, removal of biomass as in fishing or logging).
Reassembly	A process in which a new ecosystem replaces one that is collapsing or has collapsed. Typically this involves the loss of some ecosystems components, addition of others and alterations to processes that drive ecosystem dynamics and resilience (see disassembly)
Ruderal	Plants with a combination of life-history traits that enable colonisation of open post-disturbance environments. Traits and related trade-offs include rapid growth, high fecundity, wide propagule dispersal, short life-span, high demands for nutrients and intolerance of competition.
Sclerophyll	Plants or vegetation bearing leaves hardened by an abundance of woody tissue (sclerenchyma) and thick cuticles. Typically associated with environments that experience limited nutrients or water or cold stress.
Secondary productivity	Biomass of heterotrophic (consumer) organisms generated in a given length of time, driven by the transfer of organic material between trophic levels.
Serotinous	Refers to seedbanks that are held in woody fruits retained on the parent plant for later release, which may occur spontaneously or en masse in response to fire or adult mortality.
Semelparous	Plant life cycle with a single reproductive episode before death.
SLA	Specific Leaf Area, the ratio of area of a fresh leaf to its dry mass. Positively related to plant relative growth rate.
Succulent	Having tissues (usually leaves or stems of plats) engorged with water, as a mechanism for drought tolerance or salt dilution.
Species traits	Properties of species (e.g. ecophysiological, morphological, behavioural or life-history features) that influence their survival, growth or reproduction (after Violle et al. 2014).
Top-down regulation	Describing a strong influence of consumers, such as herbivores and predators or fire, on ecosystem structure (also known as ‘brown world’ and ‘black-world’)
Ultramafic	Rocks and derivative soils with low silica content, also low in Potassium, but with high concentrations of Magnesium and Iron.
Vicariance	The geographical segregation of biota promoting evolution in isolation.

Term	Definition
Xeromorphic	Plants and animals possessing traits that enable them to tolerate drought by storing water, enhancing uptake and reducing loss. Example traits include nocturnal activity, deep roots, etc.

*Descriptive profiles use ordinal descriptors (high, medium, and low) of productivity, which refers to Net Primary Productivity, unless otherwise stated. For terrestrial and transitional realms, these descriptors are based on estimates from an ensemble of global vegetation models (Cramer et al., 1999; Kicklighter et al., 1999; Huston and Wolverton, 2009). For marine surface systems, they are based on estimates of chlorophyll *a* concentration for the upper 30 m of the water column (Sarmiento et al., 2004; Huston and Wolverton, 2009):

High: >2,000 g dry mass $m^{-2}.yr^{-1}$ for terrestrial and transitional ecosystems; >8 $mg.m^{-3}$ chlorophyll *a* concentration for marine ecosystems.

Medium: 500–2,000 g dry mass $m^{-2}.yr^{-1}$ for terrestrial and transitional ecosystems; 0.1–8 $mg.m^{-3}$ chlorophyll *a* concentration for marine ecosystems.

Low: <500 g dry mass $m^{-2}.yr^{-1}$ for terrestrial and transitional ecosystems; <0.1 $mg.m^{-3}$ chlorophyll *a* concentration for marine ecosystems.
