# Supplementary material for: A function-based typology for Earth’s ecosystems
Source: Nature. 2022 Oct 12;610(7932):513–8. doi: 10.1038/s41586-022-05318-4 (PMC9581774; doi:10.1038/s41586-022-05318-4)
Supplement: Supplementary file 2 — Reporting Summary [file 41586_2022_5318_MOESM2_ESM.pdf]

nature portfolio

Corresponding author(s): David Keith

Last updated by author(s): Apr 14, 2022

Reporting Summary

Nature Portfolio wishes to improve the reproducibility of the work that we publish. This form provides structure for consistency and transparency in reporting. For further information on Nature Portfolio policies, see our Editorial Policies and the Editorial Policy Checklist.

Statistics

For all statistical analyses, confirm that the following items are present in the figure legend, table legend, main text, or Methods section.

n/a	Confirmed	
☒	☐	The exact sample size (*n*) for each experimental group/condition, given as a discrete number and unit of measurement
☒	☐	A statement on whether measurements were taken from distinct samples or whether the same sample was measured repeatedly
☒	☐	The statistical test(s) used AND whether they are one- or two-sided<br>*Only common tests should be described solely by name; describe more complex techniques in the Methods section.*
☒	☐	A description of all covariates tested
☒	☐	A description of any assumptions or corrections, such as tests of normality and adjustment for multiple comparisons
☒	☐	A full description of the statistical parameters including central tendency (e.g. means) or other basic estimates (e.g. regression coefficient) AND variation (e.g. standard deviation) or associated estimates of uncertainty (e.g. confidence intervals)
☒	☐	For null hypothesis testing, the test statistic (e.g. *F*, *t*, *r*) with confidence intervals, effect sizes, degrees of freedom and *P* value noted<br>*Give P values as exact values whenever suitable.*
☒	☐	For Bayesian analysis, information on the choice of priors and Markov chain Monte Carlo settings
☒	☐	For hierarchical and complex designs, identification of the appropriate level for tests and full reporting of outcomes
☒	☐	Estimates of effect sizes (e.g. Cohen's *d*, Pearson's *r*), indicating how they were calculated

Our web collection on statistics for biologists contains articles on many of the points above.

Software and code

Policy information about availability of computer code

Data collection	All data used in this study came from published sources as cited. Arc Map v 10.2.2, GRASS GIS v 7.4.0, PostGIS v 2.4.3 and Google Earth Engine were used to import, edit and curate input spatial data.
Data analysis	Table S4.1 details the assembly methods for thumbnail maps presented in descriptive profiles of Ecosystem Functional Groups in Appendix S4. GRASS GIS v 7.4.0, R statistical package v 3.6.1 and Python v 3.7.3 were used for data analysis. Detailed descriptions and code will also be available at: https://doi.org/10.5281/zenodo.6459843. Code for visualisation of the data in Earth Engine is available at: https://zenodo.org/record/6459698, users can also add the following repository to the Earth Engine Code Editor: https://code.earthengine.google.com/?accept_repo=users/jrferrerparis/IUCN-GET

For manuscripts utilizing custom algorithms or software that are central to the research but not yet described in published literature, software must be made available to editors and reviewers. We strongly encourage code deposition in a community repository (e.g. GitHub). See the Nature Portfolio guidelines for submitting code & software for further information.

Data

Policy information about availability of data

All manuscripts must include a data availability statement. This statement should provide the following information, where applicable:

- Accession codes, unique identifiers, or web links for publicly available datasets
- A description of any restrictions on data availability
- For clinical datasets or third party data, please ensure that the statement adheres to our policy

Profiles, diagrammatic assembly models and interactive maps are available at https://global-ecosystems.org. Permanent record of the current version of the profiles is available at https://doi.org/10.5281/zenodo.6459844 (All versions available at https://doi.org/10.5281/zenodo.6459843). Permanent record of the current

version of the spatial data for the indicative distribution maps of the Ecosystem Functional Groups is available at: https://zenodo.org/record/5090419 (all versions available at https://doi.org/10.5281/zenodo.3546513).

Field-specific reporting

Please select the one below that is the best fit for your research. If you are not sure, read the appropriate sections before making your selection.

☐ Life sciences ☐ Behavioural & social sciences ☒ Ecological, evolutionary & environmental sciences

For a reference copy of the document with all sections, see nature.com/documents/nr-reporting-summary-flat.pdf

Ecological, evolutionary & environmental sciences study design

All studies must disclose on these points even when the disclosure is negative.

Study description	Our study presents a new typology for Earth's ecosystems and reviews its strengths, weakness and recent and potential applications to conservation and sustainability from global to local scales.
Research sample	The typology was developed by consensus among the 41 authors and 55 reviewers, selected based on published expertise encompassing terrestrial, freshwater and marine ecosystems, as well as global synthesis.
Sampling strategy	Not applicable
Data collection	Spatial data were compiled from published sources (documented in Appendix S4) primarily by DAK, JRFP and NJM.
Timing and spatial scale	Most spatial data sets were published between years 2000 and 2021 (see Table S4.1 for full details of sources), spatial resolution was as published in original sources or else reclassified to 30 arc seconds to ensure clear representation in the thumbnail maps in Appendix S4.
Data exclusions	Not applicable
Reproducibility	Development, revision and update history for the typology and its units are fully documented. No experiments were undertaken.
Randomization	Not applicable
Blinding	Not applicable

Did the study involve field work? ☐ Yes ☒ No

Reporting for specific materials, systems and methods

We require information from authors about some types of materials, experimental systems and methods used in many studies. Here, indicate whether each material, system or method listed is relevant to your study. If you are not sure if a list item applies to your research, read the appropriate section before selecting a response.

Materials & experimental systems

n/a	Involved in the study	
☒	☐	Antibodies
☒	☐	Eukaryotic cell lines
☒	☐	Palaeontology and archaeology
☒	☐	Animals and other organisms
☒	☐	Human research participants
☒	☐	Clinical data
☒	☐	Dual use research of concern

Methods

n/a	Involved in the study	
☒	☐	ChIP-seq
☒	☐	Flow cytometry
☒	☐	MRI-based neuroimaging
