# Supplementary material for: A function-based typology for Earth’s ecosystems
Source: Nature. 2022 Oct 12;610(7932):513–8. doi: 10.1038/s41586-022-05318-4 (PMC9581774; doi:10.1038/s41586-022-05318-4)
Supplement: Supplementary file 4 — Review of existing ecological typologies. Descriptions and justifications of six principles that a global ecosystem typology should meet to support planning and management decisions for biodiversity conservation and sustain ecosystem services. We use the principles to frame a tabular comparison of 23 existing ecological classifications of global extent and conclude that none meet all six principles. [file 41586_2022_5318_MOESM4_ESM.pdf]

Appendix S1. Review of existing ecological typologies

'*A function-based typology for Earth's ecosystems*'

David A. Keith, Jose R. Ferrer-Paris, Emily Nicholson, Melanie J. Bishop, Beth A. Polidoro, Eva Ramirez-Llodra, Mark G. Tozer, Jeanne L. Nel, Ralph Mac Nally, Edward J. Gregr, Kate E. Watermeyer, Franz Essl, Don Faber-Langendoen, Janet Franklin, Caroline E. R. Lehmann, Andres Etter, Dirk J. Roux, Jonathan S. Stark, Jessica A. Rowland, Neil A. Brummitt, Ulla C. Fernandez-Arcaya, Iain M. Suthers, Susan K. Wiser, Ian Donohue, Leland J. Jackson, R. Toby Pennington, Thomas M. Iliffe, Vasilis Gerovasileiou, Paul Giller, Belinda J. Robson, Nathalie Pettorelli, Angela Andrade, Arild Lindgaard, Teemu Tahvanainen, Aleks Terauds, Michael A Chadwick, Nicholas J. Murray, Justin Moat, Patricio Pliscoff, Irene Zager, Richard T. Kingsford

Nature 2022

Introduction

A large number of ecological typologies have been designed for diverse applications at a range of global to local scales. We define an 'ecological' typology as a classification of land, water or bioclimate intended to represent variation in the expression of ecological features. Ecosystem typologies are therefore a subset of ecological typologies. As noted in the main text, to serve the dual needs for conservation and sustainability, an ecosystem typology must represent both ecosystem functions and the biota engaged in them. For application across diverse ecosystems, users and scales of analysis, a useful ecosystem typology must also be conceptually consistent throughout the biosphere, have a scalable structure, spatially explicit, well-described units, and avoid superfluous complexity.

Prior to developing the IUCN Global Ecosystem Typology, we developed six design principles representing these qualities and used them to review a large sample of existing ecological typologies to compare their features and evaluate their suitability for assessing the functions and biodiversity of ecosystems and for reporting on their status throughout the biosphere. We subsequently used the six design principles to guide the development of the IUCN Global Ecosystem Typology.

Methods

The diverse approaches employed in the development of ecological typologies include delineations based on patterns in diversity and endemism of particular taxonomic groups, traits of structurally dominant biota, and biophysical proxies, such as climate, hydrology, geomorphology or geochemistry. We identified seven groups of ecological typologies according to their approach and the types of features on which their units are based: i) biogeographic regionalisations; ii) compositional clusters; iii) biophysical classifications; iv) climatic biomes and vegetation; v) functional biomes; vi) ecosystems; and vii) species habitats. We identified a sample of 23 typologies that represent these seven groups of approaches and span the global range of terrestrial, freshwater and/or marine environments. Although most of the typologies were systematic classification frameworks, we also included

some that were not intended for systematic application because they had relevant properties that provide insights into functions and biota of ecosystems, including chapter structures for multi-volume treatises on the world's ecosystems (Goodall 1974-2005; Goldstein & DellaSala 2020) and habitat classifications for individual species (IUCN 2012; Jung et al. 2020). A much larger range of regional- and national-scale ecological typologies were beyond the scope of our review. We evaluated each typology against six design principles defined in Table S1.1.

Table S1.1. Design principles for a global ecosystem typology and review of existing ecological typologies (see Table S1.2 for more details).

Principle	Description	Rationale	Existing typologies
1 Representation of ecological processes & ecosystem functions	Groupings reflect major ecological processes and ecosystem functions that shape ecosystem assembly and maintain their defining characteristics and traits.	Enables generalisations and predictions about ecosystem functions and dynamics; responses to environmental change and management actions; and ecosystem services that flow from functions. Major ecosystem components, drivers, states and transitions should be consistent within a group.	Eight typologies represented ecological processes and/or ecosystem functions directly, including 5 explicitly through global ecosystem models. Fifteen represented processes only incidentally or indirectly through assumed relationships.
2 Representation of biota	Within groups, ecosystems are distinguishable through differences in the identity (composition) of their biota.	Enables generalisations and predictions about biodiversity for conservation and management applications. Units reflect not only functions, but the identity of engaged biota, and thus should serve as informative units for biodiversity conservation.	Four typologies represented biota explicitly, but none of the seven that addressed Principle 1 explicitly (above). The 19 remaining typologies represented biota only incidentally or indirectly through assumed relationships with traits or biophysical proxies or spatial autocorrelation.
3 Conceptual consistency throughout the biosphere	The typology should encompass all components of the biosphere within a single theoretical framework.	Promotes comprehensive coverage of ecosystems from all environments, conceptual consistency, robustness to new knowledge, consistent ecosystem identification and comparison between units.	Eight typologies were explicitly based on theory while others failed to articulate a clear conceptual basis; only three typologies spanned terrestrial, freshwater and marine environments (none with a clear conceptual basis); 12 were terrestrial (or part thereof); 2 were freshwater; 6 were marine (or part thereof).
4 Scalable structure	Groupings should be arranged in a hierarchical or nested structure to reflect	Enables representation of different features at particular hierarchical levels and facilitates applications across a range of spatial and organisational scales.	Fourteen typologies had a hierarchical structure, most of them with 2 or 3 levels and one with 8 levels. Nine typologies had no scalable structure.

Principle	Description	Rationale	Existing typologies
	the nature and magnitude of their similarities.		
5 Spatially explicit units	Distributions of units should be mappable through any practical combination of ground observation, remote sensing and spatial modelling.	Enables comparative spatial analyses and time series analyses of ecosystem extent and distribution for monitoring and reporting.	12 typologies were digitally mapped globally at high resolution and a further 4 had coarse maps and 5 were not spatially explicit.
6 Parsimony & utility	The typology should be no more complex than required to achieve other specifications and should use simple, accessible and clearly defined terminology.	Facilitates wide usage among people with varied objectives, skills and backgrounds.	15 typologies had moderate or low parsimony and utility due to either complex or vague diagnostic criteria, large numbers of units, limited descriptions or limited spatial information. The remainder supported users with data for spatial diagnosis or detailed descriptions of distinguishing features, mostly for manageable numbers of units.

Results and Discussion

None of the ecological typologies that we reviewed met all six evaluation criteria, highlighting key differences with the IUCN Global Ecosystem Typology, which was designed to meet all six criteria (Table S1.2). None of the other typologies were designed explicitly to represent both ecosystem functions (Principle 1, Table S1.1) and biota (Principle 2). The IUCN typology has explicit conceptual links to ecosystem functions through its underlying ecosystem assembly model (Appendix S2). Most other typologies are based on biogeography or biophysical attributes, with implied rather than explicit representation of assembly processes.

A second major distinction is the broad scope of the IUCN typology, which encompasses the entire biosphere within a single conceptual framework. The scope of most other global typologies is limited to either terrestrial, freshwater or marine environments, with different classificatory approaches applied to each. Only three of 23 classifications that we reviewed attempted full coverage of the biosphere, but those failed to meet other design criteria (Table S1.2). These and some other typologies lacked a clear theoretical basis (Table S2.1), in part because they were not designed or intended for systematic applications to ecosystems.

Finally, very few other global typologies provide detailed, consistent and systematic ecological descriptions of their units of classification (Table S1.2 cf. Appendix S4), which limits their diagnostic utility, especially in the field. Some typologies (e.g. most of the biophysical classifications) used thresholds or clusters based on quantitative global variables to define their units with explicit descriptions. However, these units may still be difficult to interpret and identify in the field because the variables require specialised in situ instruments or laboratory analyses to compare field observations to thresholds for identification, or because globally modelled estimates deviate from those on the ground. For some biophysical variables, such as temporal averages or minima, measurements are required over long time frames to estimate their values at locations on the ground.

Three ecoregional classifications, respectively, of terrestrial (Olson et al. 2001; Dinerstein et al. 2017); coastal marine (Spalding et al. 2007) and freshwater (Abell et al. 2008) environments are among the most widely used of global ecological classifications that we reviewed (Table S1.2). All three were developed by expert elicitation and have a primary basis in biogeography, with the aim of representing the distribution of biodiversity. Tests of terrestrial ecoregions indicate that they serve this purpose well (Smith et al. 2018). For this reason, in our future work, they will be used as spatial templates for developing maps of Level 4 units in the IUCN Global Ecosystem Typology by subdividing Ecosystem Functional Groups (Level 3). Collectively, the three ecoregion classifications cover most of the biosphere but, unlike the IUCN GET, they do not classify marine pelagic, deep sea floor or subterranean environments. The approaches to classification also differ slightly among the three ecoregion classifications, which were developed largely independently of one another. For example, terrestrial ecoregions were largely a synthesis of national and regional land classification, whereas freshwater ecoregions were framed around watersheds (catchments) as dispersal barriers based on occurrences of freshwater aquatic taxa for which suitable data were available (primarily fish).

Ecoregions are intentionally broad, often heterogeneous biogeographic regions, each of which contains multiple (and in some cases, highly contrasting) IUCN Ecosystem Functional

Groups. Although some the labels of ecoregions appear to represent specific ecosystem types, the detailed descriptions, where available, characterise the heterogeneity of functionally contrasting ecosystem types present within them. For example, the Cantabrian mixed forests ecoregion (https://www.worldwildlife.org/ecoregions/pa0406) is a relatively small ecoregion that is likely to encompass multiple Ecosystem Functional Groups (including T2.2, T3.2, T3.3, T4.4, T6.4, T7.1, T7.2, T7.3, T7.4 ,T7.5, TM2.1, as well as several transitional terrestrial-freshwater and terrestrial-marine groups). Similar heterogeneity is evident in numerous ecoregions. This is not a criticism, but rather a reflection of the intended character of ecoregions as mosaics of ecosystem types that share common biogeographic settings. Thus, the assessments based on the IUCN Global Ecosystem Typology will offer results that are more focussed on ecosystems (as defined in the CDB) and more granular than is possible from alternative ecological classifications.

We also draw a distinction between ecosystem typologies and land use classifications (e.g. Di Gregorio & Jansen 2000). Given fundamentally different purposes and trade-offs in design principles, the IUCN Global Ecosystem Typology will not be fit for all purposes that a land use classification can serve, and vice versa. We did not include land use classifications in our review of ecological typologies because they are classes of human economic, social and cultural activity that reflect the types and intensity of interactions between humans and their environment (Erb et al. 2017; Mayfroidt et al. 2018). Land use classifications typically group 'natural' and 'semi-natural' systems with low land use intensity into broad land cover categories based on plant life form, cover, height, and micropattern (Di Gregorio & Jansen 2000); attributes that are, at best, indirect proxies for ecosystem function. They classify higher intensity land uses on the basis of different criteria, for example, cultivated areas are partitioned by plant growth form, field size and spatial distribution, crop combination and cover-related cultural practices (Di Gregorio & Jansen 2000). Other land use mapping approaches estimate the intensity of different land use types (e.g. Erb et al. 2013).

In a broad sense, the anthropogenic components of the IUCN Global Ecosystem Typology address similar themes to some land use classifications. For example, intensity mapping of land uses by Erb et al. (2007) corresponds broadly to five anthropogenic ecosystem functional groups in the terrestrial biome of the IUCN typology: cropping (T7.1 Annual croplands); grazing (T7.2 Sown pastures and fields, T7.5 Derived semi-natural pastures and old fields); forestry (T7.3 Perennial crops and plantations); and infrastructure (T7.4 Urban and industrial systems). However, anthropogenic ecosystem types (defined as those that are created and sustained by intensive human activities, see Glossary, Appendix S4) should not be confused with land use activities. For example, grazing and forestry activities occur at varying intensities across natural systems, but in anthropogenic ecosystems (T7.2, T7.3), they occur at high intensities, that have transformative effects resulting in qualitatively different ecosystem properties and organisational processes that may not persist when the activity ceases (Erb 2013).

We provide a synopsis of reviews against the six design principles below. Although all of the ecological typologies that we reviewed had relevant characteristics, we conclude that none were entirely suitable for comprehensive, globally consistent assessments of ecosystem functions and biodiversity.

Principle 1. Representation of ecological processes and ecosystem functions

Among the eight groups of typologies, functional biome (trait-based) classifications represent ecosystem functions and assembly processes most explicitly, because they were linked to or derived from global models of ecosystem function (e.g. Woodward et al. 2004). While some of these models are mechanistic (e.g. Higgins et al. 2016), others are qualitative conceptual frameworks, including classical climatic biome typologies (e.g. Walter 1976). Some ecological processes are implicit in classifications of ecosystems and vegetation (e.g Faber-Langendoen et al. 2014), and indirectly represented in biophysical classifications (e.g. Sayr et al. 2017) through environmental proxies. In contrast, biogeographic regionalisations (e.g. Olson et al. 2001) and species' habitat classifications (IUCN 2012) were not designed explicitly to represent ecological processes, but may represent them incidentally because processes are not independent of the species engaged in them (Table S1.2).

Principle 2. Representation of biota

The typologies based on biogeographic patterns and composition-based vegetation classifications both perform well in representation of biota (Table S1.2). In contrast, typologies that represent ecological processes well (Principle 1) generally perform poorly on biota (Table S1.2), suggesting a trade-off between Principles 1 and 2. Biophysical classifications represent biota incidentally because species habitats are, in part, defined by biophysical variables. However, their efficacy in representing biota depends on how well the biophysical proxies describe the habitats of species, a point that was not explored in any of the biophysical classifications examined.

Principle 3. Conceptual consistency throughout the biosphere

Only Goodall (1974-2005), IUCN (2012) and Goldstein & DellaSala (2020) covered terrestrial, freshwater and marine environments of the biosphere (Table S1.2). These lacked a consistent rationale or conceptual basis for synthesis and classification across terrestrial and aquatic environments, although several contributions within Goodall (1974-2005) provide insights into concepts applied to develop typologies for different parts of the biosphere, and the IUCN (2012) species habitat classification borrowed parts of its structure from other schemes (Holdridge 1947; Ramsar Convention 1990). In combination, the ecoregion classifications (Olson et al. 2001; Spalding et al. 2007; Abell et al. 2008) covered most of the biosphere, except for oceanic pelagic and subterranean ecosystems, but each had slightly different conceptual foundations and methods of derivation (Table S1.2). The typologies with the strongest theoretical basis were those associated with models (e.g. Woodward et al. 2004) or ecosystem theory (Higgins et al. 2016).

Principle 4. Scalable structure

Roughly half of the typologies incorporate scalable hierarchies, the main exceptions being trait-based functional biomes (Table S1.2). Most of the hierarchical typologies incorporate three or fewer levels. The EcoVeg system (Faber-Langendoen's et al. 2014) has the most elaborate hierarchical structure with eight levels, allowing detailed compositional relationships to be represented at its finest level, and structural and physiognomic features to be represented in its upper levels.

Principle 5. Spatially explicit units

All but five of the 23 typologies have spatially explicit (i.e. mapped) units (Table S1.2). Four typologies had coarse-resolution maps that were not available in digital format or based on >1 degree grids. The remaining typologies were had maps available in digital format, either as global-scale polygons or as grids with cells usually between 30 arc-seconds and 0.5 degree

resolution, and were ranged as ‘fine resolution;. In some spatially explicit typologies, available spatial proxies defined the ecosystem units directly (e.g. Higgins et al. 2016, Sayre et al. 2017). In others, the units were defined independently of spatial data, which were later used to generate indicative maps of distribution (e.g. Costello et al. 2017).

Principle 6. Parsimony and utility

All typologies achieve a degree of parsimony for their stated purpose, while those with large hierarchical structures (e.g. Faber-Langendoen et al. 2014) or coupled to sophisticated mechanistic models (e.g. Woodward et al. 2004) are among the more complex (Table S1.2). None of the typologies explicitly address integration with established local or regional classification units in wide use. Crosswalks and integration can be done spatially for those typologies with high resolution maps, while typologies with detailed descriptions of the units (e.g. Faber-Langendoen et al. 2014) allow crosswalks to be constructed from expert opinion or automated attribute matching. However, very few of the typologies had sufficiently detailed descriptions that would enable users to readily interpret and diagnose the features of their units in the field, a key pre-requisite for effective ecosystem management.

Table S1.2. Review of existing typologies against design criteria (see Table S1.1). Classification units use nomenclature of respective sources.

Classification units	Approach	Source	1 Representation of ecological processes	2 Representation of biota	3 Conceptual consistency throughout biosphere	4 Scalable structure	5 Spatially explicit units	6 Parsimony & utility
**IUCN Global Ecosystem Typology**								
110 Ecosystem Functional Groups within 25 functional biomes and 5 realms with three lower levels of classification	Three upper levels developed deductively from the top-down based on ecosystem assembly models, with level 4 based on biogeographic subdivision of level 3, and levels 5 and 6 developed from the bottom-up based on aggregation of compositional patterns	This publication (v2.1)	Direct – upper 3 levels derived from explicit conceptual model of ecosystem assembly processes	Explicit in Levels 5 & 6 (via bottom-up aggregation of field-based data and expert input). Implicit in Level 4 (via biogeography & endemism patterns).	Explicit via conceptual model of ecosystem assembly	6-level hierarchy	fine, -digital	High: detailed text descriptions, assembly models, illustrations and global maps available for all units in top 3 levels, future development of descriptions for units in bottom 3 levels
**Biogeographic ecoregions**								
193 biogeographic provinces within 8 biogeographic realms	Subjective synthesis of earlier treatments of plant and animal biogeography	Udvardy (1975)	Incidental	Implicit via biogeography & endemism patterns	Implicit biogeographic concepts; Terrestrial coverage (Freshwater implicit)	2-level hierarchy	coarse, non-digital	Low-Moderate: unclear distinctions among 193 units with low spatial resolution & limited descriptions
846 terrestrial ecoregions within 14 biomes (8 biogeographic realms, non-hierarchical)	Assembled by stitching & rationalisation of regional maps with reference to vegetation, and plant and vertebrate species distributions	Olson et al. (2001); Dinerstein et al. (2017)	Incidental	Implicit via biogeography & endemism patterns	Implicit biogeographic concepts; Terrestrial coverage	2-3-level hierarchy	fine, digital	High-Moderate: 846 units identifiable with high spatial resolution & mostly detailed descriptions (some undescribed)

Classification units	Approach	Source	1 Representation of ecological processes	2 Representation of biota	3 Conceptual consistency throughout biosphere	4 Scalable structure	5 Spatially explicit units	6 Parsimony & utility
426 freshwater ecoregions within 8 biogeographic realms	Assembled by stitching & rationalising regional maps with reference to fish distributions in regional catchments, characterised by climate and physiography	Abell et al. (2008)	Indirect - catchment proxies	Implicit via biogeography & endemism patterns	Implicit biogeographic concepts; Freshwater coverage	2-level hierarchy	fine, digital	High: 426 units delimited by watersheds, identifiable with high spatial resolution & mostly detailed descriptions
232 marine ecoregions within 62 provinces within 12 realms	Assembled by stitching & rationalising regional maps with reference to marine species distributions	Spalding et al. (2007)	Incidental	Implicit via biogeography & endemism patterns	Implicit biogeographic concepts; Marine coast and shelf coverage	3-level hierarchy	fine, digital	Moderate: 232 units identifiable with high spatial resolution, but no descriptions available
**Compositional clusters**								
30 marine realms within 15 groups of seas and oceans and 8 major biogeographic groups	Numerical multivariate cluster analysis of grid cells attributed by species occurrence data from GBIF	Costello et al. (2017)	Incidental	Explicit via species occurrence records, but potentially sensitive to new data	Compositional resemblance & endemism concepts; Marine coverage	3-level hierarchy	fine, digital	Moderate: 30 units with high spatial resolution, quantitative compositional descriptions unavailable
**Biophysical classifications**								
~40 Life zones	Derived from factorial combinations of precipitation, evapotranspiration and temperature. 100 units mentioned, only 42 delineated. A second level (association) not developed	Holdridge (1947)	Indirect -climatic proxies	Incidental via biophysical proxies	Implicit niche model; Terrestrial coverage	no	not mapped	Low: 42(100?) conceptually simple but underspecified units with arbitrary climatic thresholds for some units, no spatial data

Classification units	Approach	Source	1 Representation of ecological processes	2 Representation of biota	3 Conceptual consistency throughout biosphere	4 Scalable structure	5 Spatially explicit units	6 Parsimony & utility
40 Wetland types within 11 Wetland subgroups and 3 Wetland groups	Intuitively derived categories based on geomorphology, water regime and chemistry, and vegetation	Ramsar Convention (1990); Scott & Jones (1995)	Indirect - geomorphic, water chemistry & inundation regime proxies	Incidental via biophysical proxies	Implicit concepts of hydrological form & function; Freshwater & coastal marine coverage	3-level hierarchy	not mapped	Moderate: 40 units with geomorphic/hydrological labels but no descriptions or spatial data
3923 Ecological Land Units	Derived from factorial combinations of categorical bioclimate, landform, lithology and landcover maps	Sayre et al. (2014)	Indirect -physical & climatic proxies	Incidental via biophysical proxies	Implicit biophysical niche concepts quantitatively mapped; Terrestrial coverage	no	fine, digital	Moderate: 3923 units with high spatial resolution, but no descriptions available
37 Ecological Marine Units	Derived from cluster analysis of five water chemistry variables and temperature	Sayre et al. (2017)	Indirect -physical & chemical proxies	Incidental via biophysical proxies	Explicit biophysical niche concepts quantitatively mapped; Marine coverage	no	fine, digital	Moderate-High: 37 units with high spatial resolution, but no descriptions available
431 'World ecosystems'	A reconfiguration of Sayre et al. (2014) derived from factorial combinations of climate, terrain and landcover units (lithology excluded cf. Sayre et al. 2014)	Sayre et al. (2020)	Indirect -physical & climatic proxies	Incidental via biophysical proxies	Implicit biophysical niche concepts quantitatively mapped; Terrestrial coverage	no	fine, digital	Moderate: 431 units with high spatial resolution, but no descriptions available
29 Geomorphic feature classes, 4 base layers	A priori benthic geomorphic features mapped quantitatively using digital bathymetric model	Harris et al. (2004)	Indirect - geomorphological proxies	Incidental via biophysical proxies	Implicit geomorphic habitat concept, quantitatively mapped bathymetric model; Marine benthic coverage	2-level hierarchy	fine, digital	High-Moderate:

Classification units	Approach	Source	1 Representation of ecological processes	2 Representation of biota	3 Conceptual consistency throughout biosphere	4 Scalable structure	5 Spatially explicit units	6 Parsimony & utility
**Climatic biomes & vegetation**								
9 Zonobiomes and 10 zono-ecotones	Derived from subjective evaluation of climatic theory & biogeographic relationships, only top-level units explicitly enumerated & described	Walter (1973); Walter & Box (1976)	Direct -explicit rationale (climatic primacy with edaphic & topographic modifiers)	Explicit at lower hierarchical levels	Explicit bioclimatic/pedological niche model; Terrestrial coverage	7-level hierarchy	coarse, non-digital	Low-Moderate: units not explicitly delineated within 9 zonobiomes that are thoroughly described but mapped at low spatial resolution
35 Biomes	Derived from subjective evaluation of climatic & biogeographic relationships based on environmental gradients: climatic moisture, temperature, tidal inundation	Whittaker (1975)	Indirect - physiognomic proxies & environmental gradients	Implicit via biota associated with vegetation structural forms	Implicit bioclimatic niche model; Terrestrial vegetation	no	not mapped	Moderate: 35 units with qualitative descriptions of climate & vegetation, no spatial data
50 formations within 17 subclasses within 7 classes. Lower levels not yet fully delineated	EcoVeg Approach: combination of subjective evaluation and numerical analyses with upper levels defined by plant growth forms and physiognomy in relation to climate, soils, topography, and human drivers, with lower levels defined by compositional and growth form similarity.	Faber-Langendoen et al. (2014)	Indirect - physiognomic & growth form proxies assumed correlative with ecological drivers	Explicit at lower hierarchical levels	Taxonomic framework based on plant growth form, physiognomy & composition; Terrestrial & aquatic vegetation	8-level hierarchy (incl. lower levels in progress)	partially mapped (regions & case studies)	Moderate: detailed descriptions complete for 50 L1-L3 units; L4-L5 largely complete; many more to be defined at L6-L8, requiring structural and compositional observations for diagnosis and detailed description

Classification units	Approach	Source	1 Representation of ecological processes	2 Representation of biota	3 Conceptual consistency throughout biosphere	4 Scalable structure	5 Spatially explicit units	6 Parsimony & utility
**Functional biomes (trait-based)**								
15 Pheno-physiognomic biome types	Derived from co-occurrences of plant functional types	Box (1996)	Direct -explicit productivity model based on tree traits	Implicit via plant traits (leaf type, phenology)	Explicit physiognomic & phenological trait model; Terrestrial woody vegetation	2-level hierarchy	coarse digital	Moderate-High: 42 units with coarse spatial resolution and semi-quantitative descriptions of bioclimate and dominant plant functional traits
14 Functional Types	Based on dominance by 13 woody plant types characterised by environmental constraints on growth defined by indices for cold tolerance, chilling requirements, heat requirements, moisture requirements and a dominance hierarchy based on growth form	Prentice et al. (1992)	Direct -explicit model of ecophysiological niches of woody plants	Incidental	Explicit ecophysiological niche model; Terrestrial vegetation	no	fine, digital	Low: 13 units with low spatial resolution delineated by ecophysiological traits requiring empirical diagnosis
10 Land cover types	Based on dominance by plant types characterised by growth form, leaf breadth, phenology and photosynthetic pathway	Woodward et al. (2004)	Direct -explicit productivity model based on plant traits	Incidental	Explicit ecophysiological trait model; Terrestrial vegetation	no	fine, digital	Low-Moderate: Low spatial resolution units requiring diagnosis by observation of plant traits

Classification units	Approach	Source	1 Representation of ecological processes	2 Representation of biota	3 Conceptual consistency throughout biosphere	4 Scalable structure	5 Spatially explicit units	6 Parsimony & utility
24 Functional biomes	Derived from factorial combinations of a vegetation productivity index, moisture- and temperature-limited seasonality indices & vegetation height	Higgins et al. (2016)	Direct -explicit model based on productivity, seasonal limiters, and vegetation height	Incidental	Explicit quantitative ecosystem function model; Terrestrial	no	fine, digital	High-Moderate: 24 units with high spatial resolution & quantitative descriptions of modelled functional traits
4 Primary biomes, 56 Biogeochemical Provinces	Subjective partitioning of ocean waters based on 8 mechanisms that limit primary productivity: polar irradiance, seasonal nutrient availability, seasonal winds and monsoons, benthic and coastal topography, and upwelling. Provinces were subsequently quantified and modelled based on bathymetry, chlorophyll concentration, sea surface temperature, and sea surface salinity	Longhurst (2007), Reygondeau et al. (2013)	Direct -explicit rationale based on productivity and mixing	Incidental	Explicit semi-quantitative biogeochemical niche model; Marine coverage	2-level hierarchy	fine, digital	High-Moderate: 56 units with high spatial resolution & quantitative descriptions of modelled biogeochemical traits
**Ecosystems**								
30 ecosystem types within 6 subgroups and 3 major groups	Approach undocumented; subjective appraisal of ecosystem processes to define thematic ecosystem types for review in a multi-volume series	Goodall (1974-2005)	Direct -implicit rationale embedded in analytical chapters	Explicit through descriptive chapters	Implicit concepts of ecosystem function; Freshwater, Marine & Terrestrial coverage	3-level hierarchy	coarse, non-digital	Moderate: 30 units with low spatial resolution but detailed text descriptions of ecological features

Classification units	Approach	Source	1 Representation of ecological processes	2 Representation of biota	3 Conceptual consistency throughout biosphere	4 Scalable structure	5 Spatially explicit units	6 Parsimony & utility
9 major sections, >100 informal biomes and exemplary areas	Approach undocumented; subjective arrangement of diverse contributions on biome ecology in a multi-volume series	Goldstein & DellaSala (2020)	Incidental and variable between sections	Implicit in descriptions of units	No clear conceptual base; Terrestrial, Freshwater & Marine coverage	2-level hierarchy implied, lower leve not fully populated	not mapped	Low: unsystematic approach to defining informal units makes them unsuitable for unintended applications
66 Large marine ecosystems	Ecosystem units distinguished from one another by (i) bathymetry, (ii) hydrography, (iii) productivity, and (iv) trophic relationships	Sherman & Alexander (1986), Sherman (1991), http://www.lme.noaa.gov/index.php	Direct -implicit rationale and criteria for defining functionally distinctive units	Implicit via regional scale of units	Explicit concepts of ecosystem function; Marine coast and shelf	no	fine, digital	High: 66 units with high spatial resolution and detailed descriptions of biophysical and trophic properties
**Species habitats**								
105 habitat types (level 2), some subdivided at level 3, and arranged in 18 groups	A subjective amalgam of existing schemes including Holdridge (1947), Ramsar Convention (1990) and others	IUCN (2012)	Incidental	Implicit via species habitat fidelity	No clear conceptual base; Freshwater, Marine & Terrestrial coverage	3-level hierarchy	Now part mapped (Jung et al. 2020)	Moderate: 105 units with intuitive labels, limited descriptions and no spatial data

References

Abell R, Thieme ML, Revenga C, Bryer M, Kottelat M, Bogutskaya N, Coad B, Mandrak N, Contreras Balderas S, Bussing W, Stiassny MLJ, Skelton P, Allen GR, Unmack P, Naseka A, Ng R, Sindorf N, Robertson J, Armijo E, Higgins JV, Heibel TJ, Wikramanayake E, Olson D, López HL, Reis RE, Lundberg JG, Sabaj Pérez MH, Petry P (2008) Freshwater ecoregions of the world: A new map of biogeographic units for freshwater biodiversity conservation. BioScience 58: 403–414. [https://doi.org/10.1641/B580507]

Box EO (1996) Plant functional types and climate at the global scale. Journal of Vegetation Science 7: 309-320.

Costello MJ, Tsai P, Wong PS, Cheung AKL, Basher Z, Chaudhary C (2017) Marine biogeographic realms and species endemicity. Nature Communications 8: 1057. [doi: 10.1038/s41467-017-01121-2]

Di Gregorio A, Jansen LJM (2000) Land cover classification system (LCCS): Classification concepts and user manual. United Nations Food and Agriculture Organization (FAO), Rome.

Dinerstein E, Olson D, Joshi A, Vynne C, Burgess ND, Wikramanayake E, Hahn N, Palminteri S, Hedao P, Noss R, Hansen M, Locke H, Ellis EC, Jones B, Barber CV, Hayes R, Kormos C, Martin V, Crist E, Sechrest W, Price L, Baillie JEM, Weeden D, Suckling K, Davis C, Sizer N, Moore R, Thau D, Birch T, Potapov P, Turubanova S, Tyukavina A, de Souza N, Pintea L, Brito JC, Llewellyn OA, Miller AG, Patzelt A, Ghazanfar SA, Timberlake J, Klöser H, Shennan-Farpón Y, Kindt R, Lillesø JB, van Breugel P, Graudal L, Voge M, Al-Shammari KF, Saleem M (2017) An ecoregion-based approach to protecting half the terrestrial realm. Bioscience 67:534-545. [doi: 10.1093/biosci/bix014]

Erb KH, Gaube V, Krausmann F, Plutzar C, Bondeau A, Haberl H (2007) A comprehensive global 5 min resolution land-use data set for the year 2000 consistent with national census data. Journal of Land Use Science 2:3, 191-224. [doi: 10.1080/17474230701622981]

Erb KH, Haberl H, Jepsen MR, Kuemmerle T, Lindner M, Müller D, Verburg PH, Reenberg A (2013) A conceptual framework for analysing and measuring land-use intensity. Current Opinion in Environmental Sustainability 5: 464–470. [doi: 10.1016/j.cosust.2013.07.010]

Erb K, Luyssaert S, Meyfroidt P, Pongratz J, Don A, Kloster S, Kuemmerle T,Fetzel T, Fuchs R, Herold ,M Haberl H, Jones CD, Marin-Spiotta, E (Marin-Spiotta, Erika), McCallum I, Robertson E, Seufert V, Fritz S, Valade A, Wiltshire A, Dolman AJ (2017). Land management: data availability and process understanding for global change studies. Global Change Biology, 23: 512-533. [doi:10.1111/gcb.13443.]

Faber-Langendoen D, Keeler-Wolf T, Meidinger D, Tart D, Hoagland B, Josse C, Navarro G, Ponomarenko S, Saucier JP, Weakley A, Comer P (2014) EcoVeg: A new approach to vegetation description and classification. Ecological Monographs 84: 533-561.

Goldstein MJ, DellaSala D (2020) *Encyclopedia of the world's biomes*. Elsevier, Amsterdam.

Goodall DW (1974-2005) *Ecosystems of the World.* 36 vol. Elsevier, Amsterdam.

Harris PT, Macmillan-Lawler M, Rupp J, Baker EK (2004) Geomorphology of the oceans. Marine Geology 352: 4–24.

Higgins SI, Buitenwerf R, Moncrieff GR (2016) Defining functional biomes and monitoring their change globally. Global Change Biology 22: 3583–3593. [doi: 10.1111/gcb.13367]

Holdridge LR (1947) Determination of world plant formations from simple climatic data. Science 105: 367-368.

IUCN (2012) Habitats classification scheme v3.1. [http://www.iucnredlist.org/technical-documents/classification-schemes/habitats-classification-scheme-ver3, downloaded 9 Apr 2018]

Jung M, Raj Dahal P, Butchart SHM, Donald PF, De Lamo X, Lesiv M, Kapos V, Rondinini C, Visconti P (2020) A global map of terrestrial habitat types. Scientific Data 7:256. [https://doi.org/10.1038/s41597-020-00599-8]

Longhurst AR (2007) Ecological Geography of the Sea. Academic Press, London.

Meyfroidt P, Roy Chowdhury R, de Bremond A, Ellis EC, Erb KH, Filatova T, Garrett RD, Grove JM, Heinimann A, Kuemmerle T, Kull CA, Lambin EF, Landon Y, le Polain de Waroux Y, Messerli P, Müller D, Nielsen J, Peterson GD, Rodriguez García V, Schlüter M, Turner II BL, Verburg PH (2018) Middle-range theories of land system change. Global Environmental Change 53: 52-67. [doi: 10.1016/j.gloenvcha.2018.08.006]

Olson, DM, Dinerstein E, Wikramanayake ED, Burgess ND, Powell GVN, et al. (2001) Terrestrial Ecoregions of the World: A New Map of Life on Earth. Bioscience 51: 933-938.

Ramsar (1990) Information Sheet on Ramsar Wetlands (RIS)–2009-2014version. Ramsar Convention Secretariat, Gland. [http://www.ramsar.org/doc/ris/key_ris_e.doc]

Reygondeau G, Longhurst A, Martinez E, Beaugrand G, Antoine D, Maury O (2013), Dynamic biogeochemical provinces in the global ocean. Global Biogeochemical Cycles 27: 1046–1058. [doi:10.1002/gbc.20089]

Sayre RG, Dangermond J, Frye C, Vaughan R, Aniello P, Breyer S, Cribbs D, Hopkins D, Nauman R, Derrenbacher W, Wright D, Brown C, Convis C, Smith J, Benson L, Paco VanSistine D, Warner H, Cress J, Danielson J, Hamann S, Cecere T, Reddy A, Burton D, Grosse A, True D, Metzger M, Hartmann J, Moosdorf N, Dürr H, Paganini M, DeFourny P, Arino O, Maynard S, Anderson M, Comer P (2014) A new map of global ecological land units — An ecophysiographic stratification approach. Association of American Geographers, Washington DC.

Sayre RG, WrightDJ, Breyer SP, Butler KA, van Graafeiland K, Costello MJ, Harris PT, Goodin KL, Guinotte JM, Basher Z, Kavanaugh MT, Halpin PN, Monaco ME, Cressie N, Aniello P, Frye CE, Stephens D (2017) A three-dimensional mapping of the ocean based on environmental data. Oceanography 30: 90–103. [https://doi.org/10.5670/oceanog.2017.116]

Sayre R, Karagulle D, Frye C, Boucher T, Wolff NH, Breyer S, Wright D, Martin M, Butler K, Van Graafeiland K, Touval J, Sotomayor L, McGowan J, Game ET, Possingham H (2020) An assessment of the representation of ecosystems in global protected areas using new maps of World Climate Regions and World Ecosystems. Global Ecology and Conservation 21: e00860.

Scott DA, Jones TA (1995) Classification and inventory of wetlands: A global overview. Vegetatio 118: 3-16.

Sherman K (1991) The Large Marine Ecosystem concept: Research and management strategy for living marine resources. Ecological Applications 1: 350-360.

Sherman K, Alexander LM (1986) Variability and management of Large Marine Ecosystems. AAAS Selected Symposium 99. Westview Press, Boulder.

Spalding MD, Fox HE, Allen GR, Davidson N, Ferdaña ZA, Finlayson M, Halpern BS, Jorge MA, Lombana A, Lourie SA, Martin KD, McManus E, Molnar J, Recchia CA, Robertson J (2007) Marine ecoregions of the world: A bioregionalization of coastal and shelf areas. BioScience 57: 573–583. [https://doi.org/10.1641/B570707]

Udvardy MDF (1975) A classification of the biogeographical provinces of the world. IUCN Occasional Paper No. 18. International Union of Conservation of Nature and Natural Resources, Morges.

Walter H (1973) Vegetation of the earth. Springer, London-New York.

Walter H, Box E (1976) Global classification of natural terrestrial ecosystems. Vegetatio 32: 75-81.

Whittaker RH (1970) Communities and ecosystems. Macmillan, New York.

Woodward FI, Lomas MR, Kelly CK (2004) Global climate and the distribution of plant biomes. Phil. Trans. R. Soc. Lond. B 359: 1465-1476. [DOI: 10.1098/rstb.2004.1525]
