# Supplementary material for: A function-based typology for Earth’s ecosystems
Source: Nature. 2022 Oct 12;610(7932):513–8. doi: 10.1038/s41586-022-05318-4 (PMC9581774; doi:10.1038/s41586-022-05318-4)
Supplement: Supplementary file 5 — Conceptual foundations for the IUCN Global Ecosystem Typology: a generic model of ecosystem assembly. Development of a conceptual model of ecosystem assembly to frame descriptions of functionally contrasting ecosystems by adapting key elements of community assembly theory (species pools, assembly filters and species traits). We describe the roles of biotic and biotic drivers, dispersal processes and evolutionary legacies and their interactions in shaping ecosystem properties. [file 41586_2022_5318_MOESM5_ESM.docx]

Appendix S2. Conceptual foundations for the IUCN Global Ecosystem Typology: a generic model of ecosystem assembly

‘*Earth’s ecosystems: a function-based typology for conservation and sustainability*’

David A. Keith, Jose R. Ferrer-Paris, Emily Nicholson, Melanie J. Bishop, Beth A. Polidoro, Eva Ramirez-Llodra, Mark G. Tozer, Jeanne L. Nel, Ralph Mac Nally, Edward J. Gregr, Kate E. Watermeyer, Franz Essl, Don Faber-Langendoen, Janet Franklin, Caroline E. R. Lehmann, Andres Etter, Dirk Roux, Jonathan S. Stark, Jessica A. Rowland, Neil A. Brummitt, Ulla C. Fernandez-Arcaya, Iain M. Suthers, Susan K. Wiser, Ian Donohue, Leland J. Jackson, R. Toby Pennington, Thomas M. Iliffe, Vasilis Gerovasileiou, Paul Giller, Belinda J. Robson, Nathalie Pettorelli, Angela Andrade, Arild Lindgaard, Teemu Tahvanainen, Aleks Terauds, Michael A Chadwick, Nicholas J. Murray, Justin Moat, Patricio Pliscoff, Irene Zager, Richard T. Kingsford

*Nature* 2022

**Rationale**

The only three existing ecological typologies that attempt to cover the entire biosphere (Goodall 1974-2005; IUCN 2012; Goldstein & DellaSala 2020) lack a consistent conceptual rationale for synthesis and classification across terrestrial and aquatic environments (Appendix S1). A strong foundation in ecological theory should enable an ecosystem typology to support generalisations and predictions about ecosystem properties and responses to environmental change that are robust to developing knowledge and data availability (Moncrieff et al. 2016). Ecological classifications based on tested and established theory are more likely to support generalisations that are robust to new information because differences between groups are founded on causal relationships, compared to classifications in which groups are based only on observed patterns and correlations.

Here we elaborate on the rationale for a conceptual model of ecosystem assembly as a basis for grouping ecosystems according to functional similarities in the upper levels of the IUCN Global Ecosystem Typology (Appendix S3) to meet the design principles listed in Table S1.1 (for definitions of terms, see Glossary in Appendix S4).

Our conceptual model draws on community assembly theory (HilleRisLambers et al. 2012; Keddy & Laughlin 2022) to elucidate potential relationships among different ecological processes that shape and interact with characteristic properties of ecosystems over time. Our purpose was to identify biological and physical properties that distinguish functionally different groups of ecosystems from one another by highlighting different ecological drivers that come to the fore in structuring their assembly. More broadly, assembly theory is used widely to test coexistence mechanisms, phylogenetic convergence, predict community composition, or isolate the roles of environmental filtering and neutral processes in shaping community structure (Ackerley & Cornwell 2007; Cadotte & Tucker 2010; HilleRisLambers et al. 2012; Keddy & McLachlin 2022), but this was not our purpose here.

Traditional assembly theory recognises three groups of filters that determine which biota from a large pool of potential colonists can occupy an area: dispersal, the abiotic environment and biotic interactions (Keddy & Laughlin 2022). In reviewing these key components of the model, we divided the abiotic and biotic filters into five groups of inter-related ecological drivers, recognising that may they act simultaneously and interact. For example, outcomes of species’ interactions typically depend on aspects of the physical environment (Menge et al. 1987; Maestre et al. 2009).

# Abiotic drivers

We segregated abiotic filters into three groups.

## Resource filters

These drivers of ecosystem assembly include the availability of five *fundamental resources* essential to sustaining all life: water, nutrients, oxygen, carbon and energy (Fig. 1). Their availability may be expressed in environmental gradients that vary across global and local scales. For example, global-scale climatic gradients shape a sequence spanning water surplus to water deficit that encompasses humid forest ecosystems in the wet tropics through subhumid grassy ecosystems to xeric ecosystems in the subtropics and warm temperate latitudes that define Walter’s (1973) zonal biomes. Local gradients shaped by topography and the depth and texture of substrates also profoundly influence water availability, resulting in juxtaposition of functionally contrasting ecosystems within landscapes (see Fig. 2 in main text). In the oceans, currents that operate on global and regional scales mediate dynamic gradients in the availability of nutrients and oxygen, while extended local depth gradients structure the availability of light energy and nutrients that assemble a sequence from eutrophic near-surface ecosystems to oligotrophic ecosystems of the deep seas (Ramirez-Llodra et al. 2010). In some cases, such as arid ecosystems, it is not only the level of resource availability, but the temporal and spatial patterns of variability, that profoundly influences ecosystem assembly and function (Morton et al. 2011).

## Ambient environmental factors

The second group of abiotic drivers includes *ambient environmental factors* that modify the availability of resources or the ability of organisms to acquire them. Ambient temperature, for example, can regulate the length of the growing season in plants, with the cumulative number of growing degree-days (0°C base) a good predictor of the distribution of trees in cold environments (Prentice et al. 1992). Temperature also defines physiological limits to cell metabolic function, with cell death occurring beyond upper and lower thresholds (Clarke et al. 2013; Oerllana et al. 2018). Similarly, salinity filters ecosystem composition by limiting the ability of some organisms to acquire water and nutrients more than others (Waterkeyn et al. 2008) and pH does so by influencing nutrient availability and uptake functions (e.g. Guerinot & Yi 1994; Orr 2005). Substrate properties, kinetic energy of aquatic media and geomorphology all influence the supply and retention of nutrients and water, and the ability of organisms to extract them by absorption, filter feeding, mobile foraging or hunting. Spatial variation in these ambient environmental factors may be expressed in global-scale climatic gradients, landscape- or seascape scale gradients in altitude, depth or geomorphology, local-scale geomorphic gradients (e.g. on sea shores) or micro-scale spatial gradients (e.g. geothermal vents and chimneys).

## Disturbance regimes

The third group of abiotic filters includes a range of *disturbances* that destroy living biomass, liberate and redistribute resources and trigger life history processes in some organisms. Consequently, they are engaged in top-down mechanisms of ecosystem assembly, in contrast to bottom-up mechanisms associated with the first two groups of abiotic processes (e.g. Bond & Keeley 2005). Typically, disturbance regimes operate at landscape or seascape scales, but may also influence ecosystem processes at regional or local scales (Archibald et al. 2013). Contrasting fire regimes, for example, influence the distribution and character of savannas, forests and Mediterranean-type shrublands (Bond & Keeley 2005; He et al. 2019), while flood regimes are integral to structuring fluvial and wetland ecosystems (Leigh et al. 2010). In general, ecosystem responses to regimes of recurring disturbances depend on features of the events (intensity, depth, duration, season) and the intervals between them (frequency).

# Biotic drivers

## Biotic interactions

These *interactions among organisms* include a wide range of ecological processes that may have positive, negative or mixed effects on the survival, growth and reproduction of the organisms that make up the biotic assemblages of ecosystems. They include competition, predation, pathogenicity, parasitism, mutualism, facilitation and symbiosis. These biotic interactions influence ecosystem assembly from landscape to local scales, most often through top-down mechanisms (Fig. 1). Trophic interactions, for example, have major influences on the assembly of large lake and pelagic ocean ecosystems (e.g. Estes et al 2016), as well as some savannas (Archibald & Hempson 2016). In contrast, mutualistic interactions between corals and unicellular algae, for example, contribute collectively as ecosystem engineers to the structure and assembly of coral reef ecosystems in tropical neritic waters (Baker 2003; Sheppard et al. 2018). When the mutualism breaks down (e.g. via repeated bleaching or disease outbreaks), the ecosystem collapses (Hughes et al. 2018). Competition is unique among ecosystems in limiting the number of species that share similar traits, whereas other biological interactions promote trait convergence rather than divergence (Keddy & Laughlin 2022).

## Human activity

Humans have had varied and changing roles in different ecosystems through the Quaternary era. In the Anthropocene, *effects of human activity* became pervasive, affecting all biophysical processes (Steffen et al. 2015) and influencing ecosystem assembly from global to local scales. While the assembly model (Fig. 1) portrays humans as integral drivers of ecosystem assembly, we segregated human activity from other biotic interactions to highlight interactions and feedbacks between ecosystems and socio-economic systems (Erb et al. 2013; Meyfroidt et al. 2016) and the need to assess their consequences for biodiversity and ecosystem functioning in designing policies that change human activities or mitigate their effects. Almost all contemporary threats to ecosystems derive directly or indirectly from human activity, with the underlying mechanisms differing from those of other drivers.

Some human activities are large transformative processes, such as change in land use and land cover, where natural or semi-natural vegetation is replaced by agricultural, urban or industrial systems (Watson et al. 2016) or where infrastructure is installed on land, watercourses or seabeds to create new systems such as large water reservoirs (dams) or artificial reefs. Others involve resource exploitation, notably water, affecting flow regimes and variation in water body levels (Vorosmarty et al. 2010). Others include direct harvesting of trophic dominants (e.g. large fish) or structural dominants (e.g. trees, corals) or engineers (e.g. large herbivores) at levels that disrupt ecosystem functions or ecological processes (e.g. Scheffer et al. 2005). Human activity also influences ecosystem assembly indirectly, for example through movement of organisms that become invasive outside their natural range, and through a diversity of mechanisms related to climate change caused by greenhouse gas emissions (Scheffers et al. 2016). These activities modify existing abiotic and biotic filters, and also create new ones. They also alter dispersal processes, breaking down some barriers and creating new ones, resulting in transformation of ecosystem functions, ecological processes and prevalent species traits (Hobbs et al. 2006).

The strength of these effects varies from negligible to transformative, resulting in changes between ecosystems types and variation within ecosystem types spatially and temporally. For example, global models of Human Appropriation of Net Primary Productivity (HANPP) estimate variation from 0 to ~100%, as well as some areas where land use increases NPP above potential natural levels (Haberl et al. 2007). In the typology, we recognise major transformative outcomes of human activity by defining anthropogenic ecosystems as those created and sustained by human activity. We distinguish these types of ecosystems from lesser human influences where the underlying identity of a ‘natural’ system (i.e. its characteristic biota and ecological processes) is retained, albeit modified to some degree. Cessation of human activities or removal of anthropogenic infrastructure may lead to transformation into ecosystem types with different attributes and organisational processes. Examples include abandonment of croplands (Foster & O’Keefe 2000), abandonment of cities (Islebe et al. 1996), decommissioning of large dams or oil platforms, etc.

Characterising the intensity of human activity requires analytical frameworks that consider the multidimensional nature of land-use, water-use or sea-use intensity (Erb et al. 2013). These frameworks are most advanced for production systems (agriculture, forestry, fisheries), with conceptual models encompassing land use inputs, outputs, their interactions, and changes in system properties (Erb et al. 2013). Spatial modelling of indices such as HANPP (Haberl et al. 2007), offer opportunities to map global distributions of certain anthropogenic ecosystem types, and to assess human impacts on natural and semi-natural types, particularly where there is continuous variation in the drivers of human activity.

For non-anthropogenic ecosystem types, we focussed descriptions on reference states with negligible influence of human activity on characteristic components and processes. Hence, human activities are not shown in the assembly models of non-anthropogenic ecosystems, even though they may exert important influences over some or all of their contemporary distributions. This approach enables the degree and nature of human influence to be described and measured against these reference states using appropriate assessment methods such as land use intensity metrics (Erb et al. 2017) and risk assessment protocols (Keith et al. 2013).

# Dispersal processes and evolutionary legacies

Dispersal processes are an important part of assembly theory that have received less attention than other filters. Occupancy outcomes are mediated by a regional species pool that defines the set of biota exposed to local selection processes associated with abiotic and biotic filters.

At scales of biomes and large ecosystems, species’ pools (more generally biotic pools) include local evolutionary legacies whose progenitors had long histories of prior occupancy (Chase 2003), as well as taxa that evolved elsewhere and arrived more recently by dispersal. Although contemporary biomes comprise various mixtures of these occupancy types (e.g. Crisp & Cook 2003), the apparent niche conservatism within many evolutionary lineages can have profound effects on ecosystem assembly and function (Wiens et al. 2010). Even rare dispersal events (Clark et al. 1998), are critical in mediating functional differences between ecosystems that occupy broadly similar climatic niches in different places. Savanna ecosystems on different continents, for example, include trees with markedly differentiated responses to fire, indicating how evolutionary legacies and limitations on intercontinental tree dispersal may affect ecosystem form and function (Lehmann & Parr 2016). Other examples include alpine and Mediterranean-type ecosystems, insular water bodies and even tropical rainforests (Corlett & Primack 2016).

Dispersal filters are especially important for assembly of ecosystems that develop functional or composition properties as a consequence of insularity. Examples on a global scale include include systems in Mediterranean-type climate zone with similar climatic drivers, but lon-isolated evolutionary lineages that retain some legacies of vicariance, but with very infrequent subsequent interchange of biota through rare dispersal events (Keeley et al. 2012).

# Ecosystem properties

Ecosystem properties are attributes of ecosystems and their component biota that result from assembly processes (see Glossary, Appendix S4). These properties include three types: i) aggregate ecosystem functions (stocks and fluxes); ii) ecological processes (e.g. trophic networks), structural features (e.g. 3-D spatial structure, physiognomy, diversity); and iii) species-level traits that influence the performance (e.g. fitness) of characteristic organisms (Violle et al. 2014). Species-level traits encompass life-histories, life-forms, morphology, phenology, behavioural and ecophysiological features (Fig. 1). The assembly processes include the combined action of the abiotic, biotic and dispersal filters described above (Fig. 1).

Many terrestrial ecosystems are dominated by vascular plants (sessile photoautotrophs) that contribute to energy capture, account for most of the world’s biomass, engage in diverse biotic interactions and define the physical structure and niche diversity of the system. Consequently, leaf, wood, root and phenological traits of plants are critical in differentiating vegetation physiognomy and structure across many different types of terrestrial ecosystems (Pérez-Harguindeguy et al. 2013), and thus provide an important means of differentiating functionally contrasting terrestrial ecosystems (Appendix S4).

In contrast, subterranean ecosystems have truncated trophic structure, generally lacking photoautotrophs, vertebrate herbivores and large predators. Heterotrophs dominate these systems, supported by chemoautotrophs and allochthonous energy, and more complex organisms display traits that reflect low metabolic rates and aphotic sensory mechanisms enabling foraging, reproduction and predator avoidance in darkness (Gibert & Deharveng 2002). Similar traits are expressed in deep marine ecosystems (Sutton 2013).

In most freshwater and marine aquatic ecosystems, primary productivity is contributed largely by mobile photoautotrophs that live in the water column (planktonic algae). Some of these systems also have benthic biofilms or macrophytes. Trophic webs are often complex and dominated by heterotrophs. Consequently, the traits of dominant heterotrophs including diets, feeding modes, morphological and behavioural traits related to movement and predation or predator avoidance are often key biotic features that differentiate functionally contrasting ecosystems in freshwater and marine environments (e.g. Rossi et al. 2017 cf. Byers & Grabowski 2014). This is especially so in the deep seas where photoautotrophs are absent. Ecophysiological traits that confer tolerance to salinity are also prominent among dominant organisms of some aquatic ecosystems (Cloern et al. 2017).

# Interactions, dependencies and feedbacks

Different assembly processes (Fig. 1) do not act independently in shaping the properties of ecosystems, but rather covary and interact through space and time (Cadotte & Tucker 2010). Resource levels, for example, may influence ecosystem assembly directly through niche partitioning or indirectly by altering biotic interactions. Variations on the model template applied to different groups of ecosystems (Fig. 1 & Appendix S4) reflect our hypotheses about drivers that operate on ecosystem attributes directly or indirectly through effects on other drivers.

Although existing knowledge is patchy and still developing, there is strong evidence of the importance of interactions, dependencies and feedbacks in ecosystem assembly across all components of the biosphere. In lotic ecosystems, for example, geomorphology and kinetic energy (turbulence) affect both the distribution of resources and flooding and drying regimes, reflecting a 3-way interaction between the ambient environment, disturbance regimes and resource availability that profoundly shape assembly and function (Lytle & Poff. 2004).

Similar interactions involving waves, tides and currents profoundly influence ecosystem structure and function on marine shorelines and shelves, while feedbacks from biotic components may regulate physical processes to maintain stability and resilience to environmental change (De Boer 2007).

On land, recurring fires (disturbance regimes, Fig. 1) produce pulses of increased light, nutrient and water availability (resources, Fig. 1) and interrupt competitive interactions (biotic interactions, Fig. 1), allowing coexistence of species with various traits promoting fire evasion, resistance, colonisation and *in situ* regeneration in fire-prone ecosystems (Bond and van Wilgen 1995; Keith 2012). Feedbacks between vegetation and fire mediate stability or regime shifts in contrasting systems that may occupy climatically and edaphically similar environments (Tepley et al. 2018).

In some cases, these interactions and feedbacks among different assembly filters and ecosystem properties are defining features that discriminate functionally contrasting ecosystem types. Understanding interactions among ecological processes, while often complex, may be critical to evidence-based inferences and generalisations identifying salient drivers for particular groups of ecosystems, such as savannas (Lehmann & Parr 2016); those dominated by megafauna (Estes et al. 2016) and many other types.

Terrestrial anthropogenic ecosystems (biome T7 in Appendix S4) provide instructive examples of interactions between human activity and other assembly filters. Assembly processes may involve complex interactions and feedbacks between ecosystems and socio-economic systems with varied settings for labour and capital inputs, technology, market dynamics, cultural beliefs, economic decision making and geopolitics (Meyfroidt et al. 2016).

We posited that addition or removal of ecosystem components by humans may directly or indirectly alter ecosystem attributes such as diversity, productivity, dominance, characteristic life-histories, etc. Direct effects may occur independently of any modifications of other drivers. For example in T7.1 Annual croplands, the introduction of new crop strains with faster growth rates is an anthropogenic assembly process that directly influences ecosystem productivity, without operating through other drivers, because the new strains are inherently more efficient in resource extraction even when supply levels are unchanged (e.g. Pan et al. 2011). These direct effects contrast with interactive assembly processes, which operate on ecosystem properties through indirect effects on other drivers. For example, introduction of disease-resistant crop strains or control agents act indirectly on ecosystem assembly by altering interactions with pathogens (Kohl et a. 2019), while addition of fertilisers operates indirectly by altering resource levels (Berzsenyi et al. 2000). Tillage can be viewed as having direct effects (e.g. by altering soil structure and function, and removing indigenous biota) and indirect interactive effects (e.g. by increasing aeration, water permeability and oxygen availability, promoting nutrient release, etc.) (Hamza & Anderson 2003).

# Synthesis

Uncertainties exist in distinguishing direct and indirect effects of all drivers, and the key drivers and their interactions remain poorly understood in many ecosystems. However, assembly models that provide for both direct and indirect interactive effects of drivers offer more complete and parsimonious hypotheses about assembly processes than models that exclude all possibility of interactions, dependencies and feedbacks. The conceptual relationships posited in the assembly models offer hypotheses to advance our understanding of these underlying mechanisms of ecosystem dynamics and assembly.

Keddy & Laughlin (2022) posited that only a few of the possible range of filters will be important for the assembly any given community. By extension, we suggest that only a few key drivers shape the properties of particular ecosystems. We use this principle to help define and describe the distinguishing features of major biomes and ecosystem functional groups in a global typology.


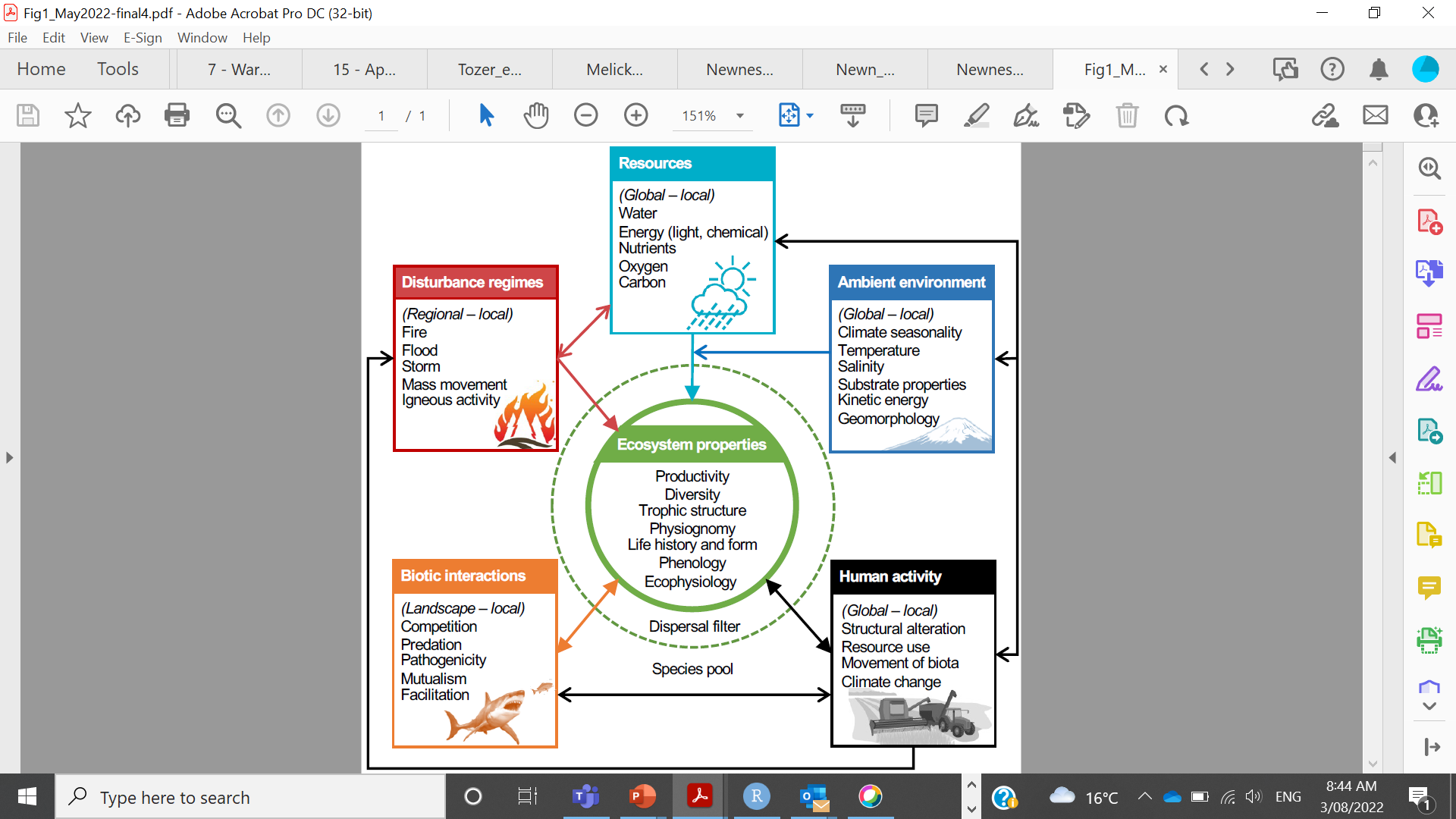


**Figure 1 [reproduced from main text]**. The generic model of ecosystem assembly underlying the global ecosystem typology (Appendix S2 for details). Boxes represent abiotic (resources, the ambient environment, disturbance regimes) and biotic (biotic interactions, human activity) drivers that filter assemblages and form evolutionary pressures, in turn, shaping ecosystem-level properties (filled green ellipse). The range of major organisational scales at which drivers operate are shown in italics in the boxes, followed by a list of the major expressions of the drivers. The species pool is the set of ‘available’ traits on which the assembly filters and evolutionary pressures operate over short and longer time frames, respectively. Species pools are dynamic products of vicariance, dispersal and evolution, that depend on biogeographic context and history. The outer green ellipse represents the contemporary dispersal filter that mediates the biota currently subjected to local selection by the abiotic and biotic filters/pressures. The inner filled green ellipse represents the properties (aggregate ecosystem functions and species-level traits) that characterise the ecosystem. Closed arrows show the influence of filtering processes on ecosystem properties. Feedbacks can occur whereby ecosystem properties modulate filtering processes (examples shown with bidirectional arrows). Interactions among drivers, include indirect effects of human activity on assembly through other drivers (black open arrows) and the indirect effects of ambient environmental conditions on assembly by modulating resource availability or uptake (dark blue open arrow). Interactions among other drivers (omitted here for simplicity) are shown in ecosystem-specific adaptations of this generic model for each ecosystem functional group (Level 3 of the typology) in Appendix S4. See Glossary (Supplementary information) for explanation of terms.

**References**

Ackerly DD, Cornwell WK (2007) A trait-based approach to community assembly: partitioning of species trait values into within-and among-community components. Ecology Letters 10: 135–145.

Archibald S, Hempson GP (2016) Competing consumers: contrasting the patterns and impacts of fire and mammalian herbivory in Africa. Philosophical Transactions of the Royal Society B 371: 20150309.

Archibald S, Lehmann CER, Gómez-Dans JL, Bradstock RA (2013) Defining pyromes and global syndromes of fire regimes. Proceedings of the National Academy of Science of the United States of America 110: 6442-6447.

Baker AC (2003) Flexibility and specificity in coral-algal symbiosis: Diversity, ecology, and biogeography of *Symbiodinium.* Annual Review of Ecology, Evolution, and Systematics 34: 661-689. [https://doi.org/10.1146/annurev.ecolsys.34.011802.132417]

Bond WJ, Keeley JE (2005) Fire as a global 'herbivore': The ecology and evolution of flammable ecosystems. Trends in Ecology & Evolution 20: 387-394 · [DOI: 10.1016/j.tree.2005.04.025]

Bond WJ, van Wilgen B (1996) Fire and plants. Chapman and Hall, New York)>

De Boer WF (2007) Seagrass–sediment interactions, positive feedbacks and critical thresholds for occurrence: a review. Hydrobiologia 591: 5-24.

Byers JE, Grabowski JH (2014) Soft-sediment communities. In: Marine community ecology and conservation (Eds. MD Bertness, JF Bruno, BR Silliman, JJ Stachowicz), pp227-249. Sinauer, Sunderland.

Cadotte MW, Tucker CM (2010) Should environmental filtering be abandoned? Trends in Ecology & Evolution 32:429-437.

Chase JM (2003) Community assembly: When should history matter? Oecologia 136: 489–498.

Clark JS, Fastie C, Hurtt G et al. (1998) Reid’s paradox of rapid plant migration. BioScience. 48: 13-24.

Clarke A, Morris GJ, Fonseca F, Murray BJ, Acton E, Price HC (2013) A low temperature limit for life on Earth. PLoS One 8:e66207. [doi: 10.1371/journal.pone.0066207]

Cloern JE, Jassby AD, Schraga TS, Nejad E, Martin C (2017) Ecosystem variability along the estuarine salinity gradient: Examples from long-term study of San Francisco Bay. Limnology and Oceanography.62: S272–S291.

Corlett RT, Primack RB (2011) Tropical Rain Forests: An ecological and biogeographical comparison. Second edition. Wiley-Blackwell, Chichester.

Crisp, MD, Cook LG (2013) How was the Australian flora assembled over the last 65 million years? A molecular phylogenetic perspective. Annual Review of Ecology, Evolution and Systematics 44: 303-324.

Estes JA, Heithaus M, McCauley DJ, Rasher DB, Worm, B (2016) Megafaunal impacts on structure and function of ocean ecosystems. Annual Review of Environment and Resources 41: 83-116. [doi: 10.1146/annurev-environ-110615-085622]

Foster DR, O'Keefe JF (2000) New England forests through time: Insights from the Harvard Forest dioramas. Harvard University Press, Cambridge.

Gibert J, Deharveng L (2002) Subterranean ecosystems: a truncated functional biodiversity. BioScience 52: 473-481.

Goodall DW (1974-2005) *Ecosystems of the World.* 36 vol. Elsevier, Amsterdam.

Green EP, Short FT (2003) World atlas of seagrasses. UNEP World Conservation Monitoring Centre. University of California Press, Berkeley.

Guerinot ML, Yi Y (1994) Iron: Nutritious, Noxious and Not Readily Available. Plant Physiology 104: 815-820.

Hamza MA, Anderson WK (2003) Responses of soil properties and grain yields to deep ripping and gypsum application in a compacted loamy sand soil contrasted with a sandy clay loam soil in Western Australia. Australian Journal of Agriculture Research 54: 273–282.

He T., Lamont BB., Pausas JG (2019) Fire as a key driver of Earth's biodiversity. Biological Reviews, in press [doi: 10.1111/brv.12544]

HilleRisLambers J, Adler PB, Harpole WS, Levine JM, Mayfield MM (2012) Rethinking community assembly through the lens of coexistence theory. Annual Review of Ecology and Evolution and Sysematics 43:227–428.

Hobbs RJ, Arico S, Aronson J, Baron JS, Bridgewater P, et al. (2006) Novel ecosystems: theoretical and management aspects of the new ecological world order. Global Ecology and Biogeography 15: 1-7.

Hughes TP, Anderson KD, Connolly SR, Heron SF, Kerry JT et al. (2018) Spatial and temporal patterns of mass bleaching of corals in the Anthropocene. Science 359: 80-83. [DOI: 10.1126/science.aan8048]

Islebe GA, Hooghiemstra H, Brenner M, Curtis J, Hodell D (1996) A Holocene vegetation history from lowland Guatemala. The Holocene 6: 265-271.

IUCN (2012) Habitats classification scheme v3.1. [http://www.iucnredlist.org/technical-documents/classification-schemes/habitats-classification-scheme-ver3, downloaded 9 Apr 2018]

Keddy PA, Laughlin DC (2022). A framework for community ecology: species pools, filters and traits. Cambridge University Press, Cambridge.

Keeley JE, Bond WJ, Bradstock RA, Pausas JG, Rundel PW (2012) Fire in Mediterranean ecosystems: ecology, evolution and management. Cambridge University Press, Cambridge.

Keith DA (2012). Functional traits: their roles in understanding and predicting biotic responses to fire regimes. In: ‘Flammable Australia: fire regimes, biodiversity and ecosystems in a changing world.’ second edition (Eds. RA Bradstock, RJ Williams, AM Gill), pp97-125. CSIRO, Melbourne.

Lehmann CE, Parr CL (2016) Tropical grassy biomes: linking ecology, human use and conservation. Philosophical Transactions of the Royal Society B. 371: 20160329. [doi: 10.1098/rstb.2016.0329]

Leigh C, Sheldon F, Kingsford RT, Arthington AH (2010) Sequential floods drive 'booms' and wetland persistence in dryland rivers: a synthesis. Marine and Freshwater Research 61: 896-908.

Lytle DA, Poff NL (2004) Adaptation to natural flow regimes. Trends in Ecology & Evolution 19: 94-100.

Maestre FT, Callaway RM, Valladares F, Lortie CJ (2009) Refining the stress‐gradient hypothesis for competition and facilitation in plant communities. Journal of Ecology 97: 199-205. [doi.org/10.1111/j.1365-2745.2008.01476.x]

Menge BA, Sutherland JP (1987) Community regulation: Variation in disturbance, and predation in relation to environmental stress and recruitment. American Zoologist 130: 730-757.

Moncrieff GR, Bond WJ, Higgin SI (2016) Revising the biome concept for understanding and predicting global change impacts. Journal of Biogeography 43, 863–873.

Morton SR, Smith DM, Stafford-Smith M, Dickman CR et al. (2011) A fresh framework for the ecology of arid Australia. Journal of Arid Environments 75: 313-329.

Orellana R, Macaya C, Bravo G, Dorochesi F, Cumsille A, Valencia R, Rojas C, Seeger M (2018) Living at the frontiers of life: Extremophiles in Chile and their potential for bioremediation. Frontiers in. Microbiology 9:2309. [doi: 10.3389/fmicb.2018.02309]

Orr JC, Fabry VJ, Aumont O, Bopp L, Doney SC, Feely RA, Gnanadesikan A, Gruber N, Ishida A, Joos F, Key RM, Lindsay K, Maier-Reimer E, Matear R, Monfray P, Mouchet A, Najjar, RG, Plattner GK, Rodgers KB, Sabine CL, Sarmiento JL, Schlitzer R, Slater RD, Totterdell IJ, Weirig MF, Yamanaka Y, Yool A (2005) Anthropogenic ocean acidification over the twenty-first century and its impact on calcifying organisms. Nature 437(7059): 681-686. [doi: 10.1038/nature04095]

Pan X, Lada RR, Caldwell CD, Falk KC (2011) Water-stress and N-nutrition effects on photosynthesis and growth of *Brassica carinata*. Photosynthetica 49: 309-315. [doi: 10.1007/s11099-011-0031-1]

Pérez-Harguindeguy N, Díaz S, Garnier E *et al.* (2013) New handbook for standardised measurement of plant functional traits worldwide. *Australian Journal of Botany* **61**, 167–234.

Prentice IC, Cramer W, Harrison SP, Leemans R, Monserud RA, Solomon AM (1992) A global biome model based on plant physiology and dominance, soil properties and climate. Journal of Biogeography 19:117–134.

Ramirez-Llodra E, Brandt A, Danovaro R, De Mol B, Escobar E, et al. (2010) Deep, diverse and definitely different: unique attributes of the world’s largest ecosystem. Biogeosciences 7: 2851–2899.

Rossi S, Bramanti L, Gori, A, Orejas C (2017) Marine Animal Forests: The ecology of benthic biodiversity Hotspots. Springer, Berlin.

Scheffer M, Carpenter S, de Young B (2005) Cascading effects of overfishing marine systems. Trends in Ecology & Evolution 20: 579-581.

Scheffers BR, De Meester L, Bridge TCL, Hoffmann AA, Pandolfi JM Corlett RT, Butchart SHM, Pearce-Kelly P, Kovacs KM, Dudgeon D, Pacifici M, Rondinini C, Foden WB, Martin TG, Mora C, Bickford D, Watson JEM (2016) The broad footprint of climate change from genes to biomes to people. Science 354(6313): aaf7671 [doi: 10.1126/science.aaf7671]

Sheppard C, Davy S, Pilling G, Graham N (2018) The biology of coral reefs. 2^nd^ Edition. Oxford University Press, Oxford

Steffen W, Broadgate W, Deutsch L, Gaffney O, Ludwig C (2015) The trajectory of the Anthropocene: The great acceleration. The Anthropocene Review 2: 81–98.

Sutton TT (2013) Vertical ecology of the pelagic ocean: Classical patterns and new perspectives. Journal of Fish Biology 83: 1508‐1527.

Tepley AJ, Thomann E, Veblen TT, Perry GLW, Holz A, Paritsis J, Kitzberger T, Anderson‐Teixeira KJ (2018) Influences of fire–vegetation feedbacks and post‐fire recovery rates on forest landscape vulnerability to altered fire regimes. Journal of Ecology 106: 1925-1940.

Violle C, Reich PB, Pacalae SW, Enquistf BJ, Kattge J (2014) The emergence and promise of functional biogeography. Proceedings of the National Academy of Sciences of the United States of America 111: 13690-13696.[doi:10.1073/pnas.31415442111]

Vorosmarty CJ, McIntyre PB. Gessner MO, Dudgeon D, Prusevich A et al. (2010) Global threats to human water security and river biodiversity. Nature 467: 555-561.

Walker B, Kinzig A, Langridge J (1999) Plant attribute diversity, resilience, and ecosystem function: The nature and significance of dominant and minor species. Ecosystems 2: 95–113.

Walter H (1973) Vegetation of the earth. Springer, London-New York.

Waterkeyn A, Grillas P, Vanschoenwinkel B, Brendonck L (2008) Invertebrate community patterns in Mediterranean temporary wetlands along hydroperiod and salinity gradients. Freshwater Biology **53**:1808-1822.

Watson JEM, Jones KR, Fuller RA, Di Marco M, Segan DB et al. (2016). Persistent disparities between recent rates of habitat conversion and protection and implications for future global conservation targets. Conservation Letters 9: 413-421. [DOI: 10.1111/conl.12295]

Wiens JJ, Ackerly DD, Allen AP, Anacker BL, Buckley LB et al. (2010) Niche conservatism as an emerging principle in ecology and conservation biology. Ecology Letters 13: 1310–1324. [doi: 10.1111/j.1461-0248.2010.01515.x]
