# Supplementary material for: A function-based typology for Earth’s ecosystems
Source: Nature. 2022 Oct 12;610(7932):513–8. doi: 10.1038/s41586-022-05318-4 (PMC9581774; doi:10.1038/s41586-022-05318-4)
Supplement: Supplementary file 6 — Structure of the IUCN Global Ecosystem Typology. A description of the structure of the typology and its rationale. Three upper hierarchical levels distinguish functionally contrasting units and three lower levels distinguish compositionally different units with similar functional properties. We contrast postulated assembly filters and ecosystem properties from our assembly model (S2) across ecosystems within the five realms of the biosphere. [file 41586_2022_5318_MOESM6_ESM.pdf]

Appendix S3. Structure of the IUCN Global Ecosystem Typology

'*A function-based typology for Earth's ecosystems*'

David A. Keith, Jose R. Ferrer-Paris, Emily Nicholson, Melanie J. Bishop, Beth A. Polidoro, Eva Ramirez-Llodra, Mark G. Tozer, Jeanne L. Nel, Ralph Mac Nally, Edward J. Gregr, Kate E. Watermeyer, Franz Essl, Don Faber-Langendoen, Janet Franklin, Caroline E. R. Lehmann, Andres Etter, Dirk Roux, Jonanthan S. Stark, Jessica A. Rowland, Neil A. Brummitt, Ulla C. Fernandez-Arcaya, Iain M. Suthers, Susan K. Wiser, Ian Donohue, Leland J. Jackson, R. Toby Pennington, Thomas M. Iliffe, Vasilis Gerovasileiou, Paul Giller, Belinda J. Robson, Nathalie Pettorelli, Angela Andrade, Arild Lindgaard, Teemu Tahvanainen, Aleks Terauds, Michael A Chadwick, Nicholas J. Murray, Justin Moat, Patricio Pliscoff, Irene Zager, Richard T. Kingsford

Nature 2022

Introduction

The IUCN Global Ecosystem Typology was developed under the auspices of the IUCN Commission on Ecosystem Management and was endorsed in Resolution 7.061 of the World Conservation Congress 2020 - Partnerships and further development of a Global Ecosystem Typology (https://portals.iucn.org/library/fr/node/49200). The typology is periodically updated as new information emerges. The structure of the current version (v2.1) is described here based on information from Keith et al. (2020), with future updates published at https://global-ecosystems.org/. The Glossary (Appendix S4) defines the terms used to describe the structure of the typology and the units within each hierarchical level.

Levels of classification within the typology

The typology comprises a nested hierarchy of units (Fig. S3.1; Table S3.1) to facilitate application at different organisational scales and enable integration of existing classifications where possible (Principle 4, see Table S1.1). Groupings in three upper levels of the typology (Table S3.1) represent ecosystems that share functional properties, irrespective of the biota engaged in the functions (Principle 1). The units of these upper levels were developed from the top-down (Fig. S3.1), with successive division to ensure global consistency and comprehensive coverage (Principle 3). We use codes F, M, T and S for systematic labelling of ecosystem units within the Freshwater, Marine, Terrestrial and Subterranean realms, respectively, and combinations of these for labelling ecosystems transitional between the realms. A fifth realm serves as a placeholder for ecosystems in the Atmosphere when knowledge is sufficient to develop their classification.

Three lower levels of the typology (Table S3.1) were designated to represent units with contrasting biotic composition (Principle 2). While neither function, nor biotic composition are intended to take primacy within the typology, functional units are represented in the upper levels of the hierarchy because representation of compositional relationships at global scale requires many more units and is more likely to change with developing knowledge than broad functional relationships among major ecosystem types. A structure that recognises

compositional variants within broad functional groupings is therefore more parsimonious and robust (Principle 6) than one that attempts to represent compositional resemblance at the upper levels. As the focus of this publication is on functional relationships, the units of the finer levels (4-6) will be described in future publications. The units of level 4 will be developed top-down by division of level 3 units (Fig. S3.1). In contrast, levels 5 and 6 will be developed from the bottom-up (Fig. S3.1) to exploit local data, detailed knowledge and established local typologies.

The overall structure of the typology is a simple hierarchy, with groups of units at each level nested within a broader unit at the level above. An important variation to this simple structure involves the units at levels 4 and 5, which are both nested within level 3 units (Fig. S3.1; Table S3.1). Level 4 (derived from the top-down) and level 5 (derived from the bottom-up) therefore represent alternative pathways below level 3. Thus, units at level 5 and 6 are nested directly within level 3, not within level 4.

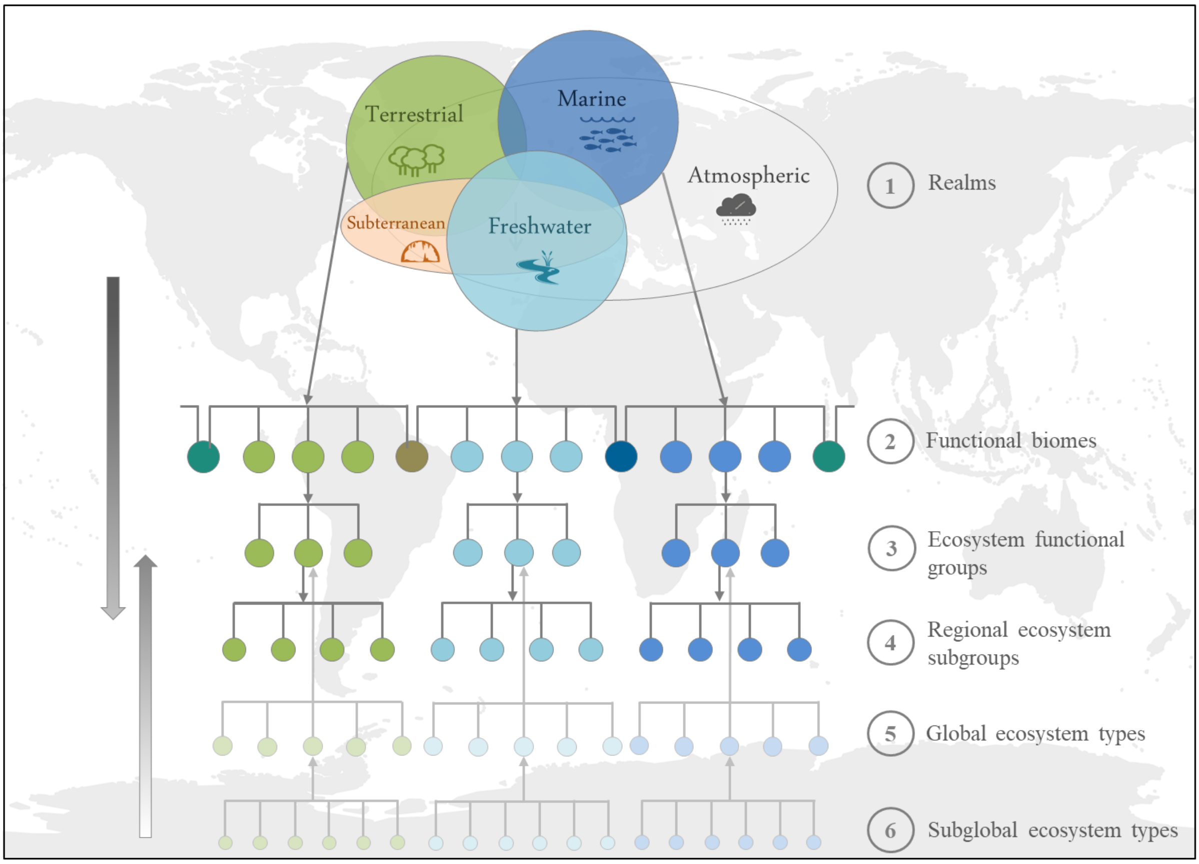

Figure S3.1. Hierarchical structure of IUCN Global Ecosystem Typology. Top cluster shows thematic overlaps of all five realms, recognising the continuous nature of variation. The expanded structure of three of the realms is elaborated (Atmospheric and subterranean realms not shown for diagrammatic simplicity). Units in three upper levels (realms, biomes and Ecosystem Functional Groups) are distinguished based on ecosystem function and ecological processes, irrespective of compositional differences in biota (Table S3.1). The bottom three levels (regional ecosystem subgroups, global ecosystem types and subglobal ecosystem types) represent units with increasing compositional homogeneity. Arrows show top-down delineation of four upper levels by successive splitting, in contrast to levels 5 and 6 aggregated from the bottom-up from local data and assigned to Level 3 units. Hence, note that Levels 4 and 5 represent alternative classificatory pathways beneath Level 3, such that Level 5 is not nested within Level 4. See Appendix S4 (pp36-168) for descriptions of functional biomes (Level 2) and Ecosystem Functional Groups (Level 3).

Table S3.1. Definitions of hierarchical levels within the global ecosystem typology.

Level	Definition
1 Realm	One of five major components of the biosphere that differ fundamentally in ecosystem organisation and function: terrestrial, freshwater, marine, subterranean, atmospheric.
2 Functional biome	A component of a realm united by one or a few common major ecological drivers that regulate major ecosystem functions and ecological processes, derived from the top-down by subdivision of realms (level 1).
3 Ecosystem Functional Group	A group of related ecosystems within a biome that share common ecological drivers promoting convergence of ecosystem properties that characterise the group. Derived from the top-down by subdivision of biomes.
4 Regional ecosystem subgroups	An ecoregional expression of an Ecosystem Functional Group derived from the top-down by subdivision of Ecosystem Functional Groups (level 3). They are proxies for compositionally distinctive geographic variants that occupy different areas within the distribution of a functional group.
5 Global ecosystem type	A complex of organisms and their associated physical environment within an area occupied by an Ecosystem Functional Group. Global ecosystem types grouped into the same Ecosystem Functional Group share similar ecological processes, but exhibit substantial difference in biotic composition. They are derived from the bottom-up, either directly from ground observations or by aggregation of subglobal types (level 6).
6 Subglobal ecosystem type	A subunit or nested group of subunits within a global ecosystem type, which therefore exhibit a greater degree of compositional homogeneity and resemblance to one another than Global ecosystem types (level 5). These represent units of established classifications (e.g. at national level), in some cases arranged in a sub-hierarchy of multiple levels, derived directly from ground observations.

Realms

Collectively, the five realms encompass the entire biosphere. We used the conceptual model of ecosystem assembly (Fig. 1 in main text) to describe and compare the ecosystem properties and postulated ecological drivers that characterise ecosystems within the five realms. This comparative review of realms helped to identify major gradients of variation (see Fig. S3.2 for examples) and informed the definition of functional biomes (Level 2) and Ecosystem Functional Groups (Level 3) within each realm. We refined the comparative review of realms iteratively as we developed levels 2 and 3 of the typology, enabling a comprehensive overview of ecosystem properties and postulated drivers across the biosphere and a synopsis of each realm (Table S3.2).

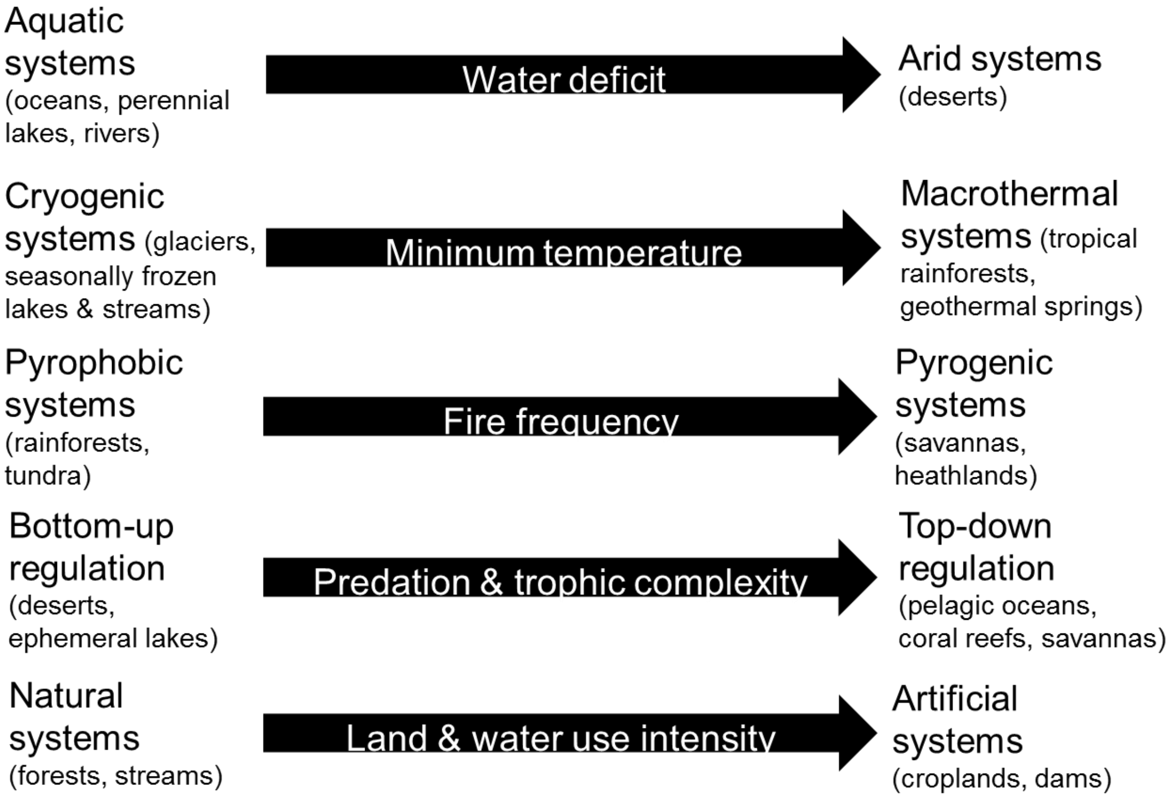

Figure S3.2. Examples of major ecosystem assembly filters represented as gradients, along which functionally contrasting ecosystems are segregated.

The terrestrial realm includes all dry land, its vegetation cover, proximate atmosphere and substrate (soils, rocks) to the rooting depth of plants, and associated animals and microbes. Water and nutrients are the principal resource drivers in terrestrial ecosystems (Fig. 1 in main text; Polis 1999; Seneviratne et al. 2010; Vitousek et al. 2010), with energy, oxygen and carbon rarely limiting (Table S3.2). Temperature and its variability on interannual, seasonal and diurnal time scales, is a major ambient environmental driver (Prentice et al. 1992), with ecosystem function and structure responding to global latitudinal and altitudinal climatic gradients. Fire is a major ecosystem driver (Fig. 1; Bond et al. 2005), essentially unique to the terrestrial realm, although it may occur rarely in the subterranean realm. Gradients in these key resources, ambient environmental factors and disturbance regimes influence biotic interactions, with the strength of competition, predation and pathogenicity varying greatly across the realm (Polis 1999). Human activity is a key driver through structural manipulations and resource appropriation and subsidies associated with land use (Erb et al. 2013, 2016), but also through movement of biota and anthropogenic climate change (Fig. 1;). The complexity of trophic webs is similarly variable, with vegetation a key feature in the expression of different ecosystem types within the realm.

The subterranean realm includes the earth's crust and subsurface voids characterised by an absence or (at void entrances) very low intensity of sunlight. Energy is therefore a major resource driver in these ecosystems (Fig. 1; Gibert & Deharveng 2002), although other resources such as water and carbon, may sometimes be limiting (Table S3.2). The ambient environment is relatively stable, although extreme temperatures occur in local areas influenced by vulcanism, and substrate type influences availability of nutrients and seepage

of moisture. Disturbances are rare and limited to mass movement or subterranean fires restricted to coal, oil or gas strata, with combustion rates limited by oxygen availability. Subterranean trophic webs are truncated, lacking photoautotrophs, herbivores and large predators (Fig. 1). The microbial biota is a key feature in the expression of different subterranean ecosystems (Engel 2010).

The freshwater realm includes all permanent and temporary freshwater bodies, as well as saline water bodies that are not directly connected to the oceans. Although some authorities describe this same unit as ‘inland water bodies’, we follow a conventional interpretation that includes saline water bodies as ‘freshwater’ for nomenclatural brevity and to avoid implications that freshwaters are always distant from the coast. Water regimes, defined by frequency, duration, flow velocity, depth and extent of inundation, are critical to the structure and function of all freshwater ecosystems (Fig. 1; Bunn & Arthington 2002). They regulate allochthonous inputs of water and nutrients from catchments (Cross et al. 2005), as well as inundation and drying regimes and turbulence (Table S3.2). Catchment geomorphology, substrates and climate (especially minimum temperatures and seasonality and interannual variability of precipitation) are key components of the ambient environment that regulate resource inputs and water regimes (Fig. 1; Thorp et al. 2006; Cassie 2006). Trophic complexity increases with the size and connectivity of the water body (Jeppersen et al. 1997). Biota of the benthos and the water column are closely associated with flow regimes, drying regimes and catchments in the diverse expressions of freshwater ecosystems.

The marine realm includes all connected saline ocean waters characterised by waves, tides and currents (Table S3.2; Todd et al. 2019). These processes transport resources and biota over short temporal and local spatial scales (waves) to global circulation over centuries (deep ocean currents). Salinity is a universal, and relatively constant ambient environmental filter throughout the realm (Fig. 1; Millero et al. 2008). The availability of light and nutrients diminishes along depth gradients (Carney 2005). Oxygen may be locally limited in zones of high heterotrophic activity (Rabalais et al. 2010). Substrate type (hard vs soft) is a salient ambient environmental factor that influences the traits of benthic fauna (Schlacher et al. 1998), while geomorphology, influences the movement of deep currents and local or regional upwelling. Upwelling and terrestrial runoff are critical to supply of nutrients to euphotic waters, and hence productivity and trophic complexity (Fig. 1; Cury et al. 2000). Primary productivity is contributed mainly by planktonic algae (Maranon et al. 2001), but benthic macrophytes can make important contributions on shallow parts of the marine shelf. Trophic interactions are critical to the structure of many marine ecosystems through both bottom-up and top-down regulatory mechanisms (Frank et al. 2007).

The atmospheric realm includes the gaseous medium and its suspended particulate liquids and solids, including bioaerosols (bacteria, fungal spores, pollen, and other bioparticles). It extends above the terrestrial realm to the altitudinal limits of life (Fröhlich-Nowoisky et al. 2016). Altitudinal gradients strongly structure the diminishing availability of water and nutrients, coincident with declines in ambient temperatures and barometric pressure, and increasing UV-B radiation (Table S3.2, Fig. 1). Nonetheless, atmospheric ecosystems are among the most dynamic over time scales of days to decades driven by planetary oscillations, lateral air movement, cloud dynamics and interaction with surface systems that drive bioaerosol cycling (Pöschl et al. 2010; Fröhlich-Nowoisky et al. 2016). We deferred resolution of lower levels of the typology within the atmospheric realm, due to the currently poor knowledge base on biotic components of these ecosystems (Williams & Smith 2021). Additional challenges to classifying atmospheric ecosystems are posed by their sparse and

itinerant biota, represented mainly by dispersive life stages. Meteorological classifications based on patterns of weather and air masses suggest a way forward to identify distinguishing drivers and properties of atmospheric ecosystems.

Transitional realms accommodate continuous variation among the core units (Fig. S3.3). The interface between terrestrial and freshwater realms is occupied by palustrine wetlands, some of which accumulate peat due to impeded substrate drainage. The freshwater-marine transitional realm is occupied by brackish aquatic ecosystems on marine coasts. The marine-terrestrial transitional realm is occupied by contrasting shoreline ecosystems, which are characterised by steep environmental gradients in desiccation, salinity and wave and tide disturbance (Fig. 1). The interface of marine and terrestrial ecosystems is sometimes influenced by outflow or seepage of freshwater, supporting intertidal wetlands and deltaic systems. Unique ecosystems also occur where the subterranean realm meets the interface of freshwater and marine realms, respectively.

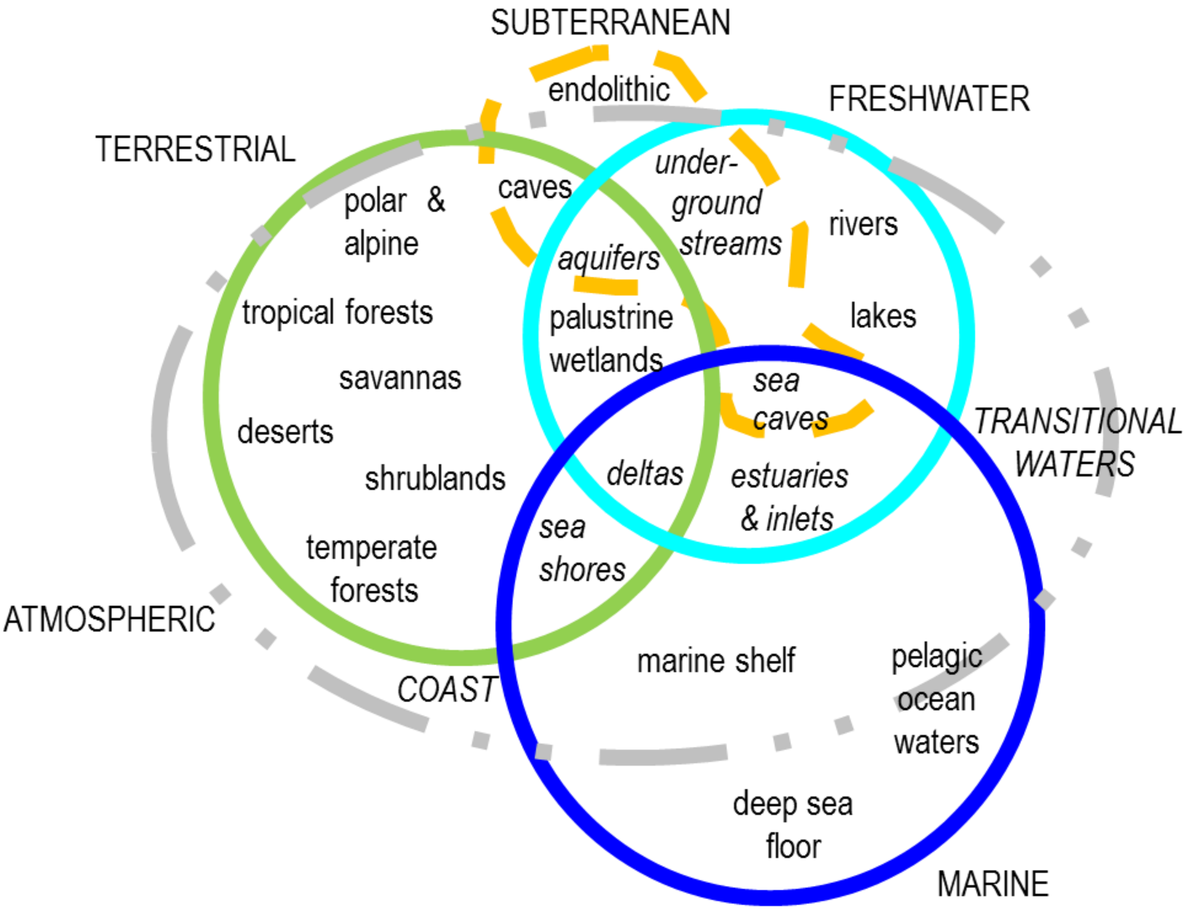

Figure S3.3 Continuous variation and transitional states among realms. Broken lines represent overlaps of Subterranean (orange) and Atmospheric realms (grey) in a fourth dimension. Transitional realms and biomes shown in italics.

Table S3.2. Synopsis of postulated assembly filters and ecosystem properties distinguishing ecosystems within the five realms of the biosphere (Fig. S3.1). Refer to Appendix S4 for glossary of selected terms and to text commentary (above) and respective descriptions of Ecosystem Functional Groups within each realm (Appendix S4).

Realm:	**Terrestrial**	**Subterranean**	**Freshwater**	**Marine**	**Atmospheric**
Substrate	Soil/Rock	Rock	Separate fresh & saline waters & benthos	Connected saline waters & benthos	Atmospheric gases
**Resource filters**					
Water	climatic & topographic gradients, sometimes limiting	diffusion gradients, sometimes limiting	climatic & topographic gradients, sometimes limiting	not limiting	convection & turbulence, limited to vapour & condensation
Nutrients	topographic & substrate gradients, sometimes limiting, climatic leaching	substrate & seepage gradients, sometimes limiting	catchment substrates & stratification gradients, sometimes limiting	sometimes limiting along depth & mixing gradients; deviations from the Redfield ratio (C:N:P)	limited to aerosols
Energy	euphotic, rarely limiting except at high latitude or by autotrophic competition	aphotic, principally chemical sources, limiting	mostly euphotic-mesophotic (rarely aphotic), depth & turbidity gradients, sometimes limiting	euphotic-aphotic, depth, turbidity & benthic geomorphology (influencing lateral & vertical flux of organic carbon) gradients, often limiting	not limiting
Oxygen	rarely limiting	diffusion & depth gradients, sometimes limiting	turbulence, diffusion, depth & consumption gradients, sometimes limiting	depth, mixing & consumption gradients, sometimes limiting (oxygen minimum zones)	not limiting

Realm:	**Terrestrial**	**Subterranean**	**Freshwater**	**Marine**	**Atmospheric**
Carbon	not limiting	diffusion gradients, often limiting	inflow & mixing gradients, sometimes limiting	depth & nutrient gradients, often limiting	allochthonous sources, limiting
**Ambient environmental filters**					
Temperature	extended hot-cold climatic gradients altered locally by topography & altitude, limiting metabolic function & growing season	geothermal heat gradients, sometimes heat-limited but typically not cold-limited	limited climatic & depth gradients, rarely heat-limited and rarely below freezing	latitudinal & depth gradients influence metabolism, productivity & growth, some systems heat-limited but rarely below freezing	extended altitudinal and regional gradients
Geomorphology	landforms influence water, nutrients, light (high latitudes)	landform influences surface connectivity, hence water, nutrients & carbon	topography defines catchment extent & form, water flow direction & velocity, influencing water & nutrient supply & flood regimes	bathymetry influences currents & habitat structure, hence nutrients, carbon, oxygen & biotic processes	topography regulate orographic uplift, hence water & atmospheric instability
Solid substrate	soil chemistry, texture & depth gradients influence nutrients & water percolation	lithology influences nutrients & structure	catchment & benthic substrates influence nutrients & water percolation	hard/soft sediment gradients define habitat structure, influence nutrients & mobility of benthic life forms	no solid substrates

Realm:	**Terrestrial**	**Subterranean**	**Freshwater**	**Marine**	**Atmospheric**
Fluid circulation	surface flow influences fine-scale nutrient & water patterns	fluid connectivity to surface influences water, nutrients, carbon & dispersal	directional flows & mixing influence oxygen, nutrients & biotic dispersal	tidal regimes & currents influence nutrients, oxygen, carbon sediment transport, biotic reproduction & dispersal	convection, wind influence water & biotic dispersal
Seasonality	influences water, energy, temperature & phenology in many systems	influences water in surface-connected systems	influences flow & filling/drying regimes, water, nutrients, temperature in many systems	seasonal productivity of surface layers influences vertical flux of nutrients & carbon through water column & to benthos	seasonal weather patterns influence water, temperature & wind
Interannual variability	very high interannual variability drives boom/bust supply of water & nutrients at extremes	low variability except in connected streams	very high interannual variability drives boom/bust supply of water & nutrients at extremes	low variability in most systems, but interannual climate cycles (e.g El Niño, Indian Ocean Dipole) & forage fish may drive trophic fluctuations	regional scale cycles such as El Niño drive large fluctuations
UV-B radiation	may limit function at extremes of altitudinal and latitudinal gradients	not applicable	rarely limiting	rarely limiting on function, diminishes with depth and turbidity	may limit function in some biota
Salinity	groundwater salinity may limit water & nutrient uptake	rarely influential	high groundwater salinity limits uptake of water & nutrients	relatively stable except on shorelines, rarely limiting	not limiting

Realm:	**Terrestrial**	**Subterranean**	**Freshwater**	**Marine**	**Atmospheric**
Geothermal flux	strong influence at local scales on nutrients and temperature	rarely influential	local gradients influence nutrients, toxins & temperature	local gradients influence nutrients, toxins & temperature	not applicable
**Disturbance regime filters**					
Fires	strong climatic and flammability gradients & feedbacks consume biomass & influence life histories, water & nutrients	surface fires may influence flux of nutrients & carbon, subterranean fires rare	catchment fires influence water and nutrient fluxes, turbidity, sedimentation rates	rarely influential except on some shorelines	smoke plume gradients influence convection, nutrients, carbon & other particulates
Floods	rare events & biomass destruction may initiate regime shifts	rare events may alter structure, remove biomass	flood regime gradients influence life histories, lateral connectivity & flux of water, nutrients & carbon	not influential except at river outflows	not applicable
Storms	climatic storm gradients influence biomass destruction, biotic dispersal, may initiate succession & regime shifts	not applicable	climatic storm gradients influence biomass destruction, turbidity & biotic dispersal, may initiate succession & regime shifts	climatic storm gradients influence surface & shoreline systems, nutrient mixing, turbidity, biotic dispersal	climatic storm gradients influence distribution of water, nutrients & biotic dispersal

Realm:	**Terrestrial**	**Subterranean**	**Freshwater**	**Marine**	**Atmospheric**
Volcanism	strong influence in local areas on nutrients & regime shifts	structures lava tube systems; rare events my alter structure, remove biomass	strong influence in local areas on nutrients, chemical energy & regime shifts	strong influence in local areas on nutrients, chemical energy & regime shifts	transient influence on particulate matter and greenhouse effects
Mass movement	strong influence in locally steep areas, biomass destruction, succession, regime shifts	infrequent but strong influence on ecosystem structure, connectivity	localised but strong influence ecosystem structure, connectivity	strong influence in tectonically active areas on nutrients, chemical energy, biomass destruction, shoreline & benthos structure	
**Biotic interactions & filters**					
Autotrophic competition	strongly vertically structured in many systems, related to resource gradients	weak or absent	may be strong in eutrophic systems & shorelines	limited except in a few shelf systems dominated by autotrophs (also some sessile heterotrophs)	mostly absent
Herbivory & predation	strong influence in many systems, related to resource gradients	weak in most systems except the most productive	influential on structure in some systems	strongly influential on structure in most systems	herbivores mostly absent, predators itinerant but potentially influential on trophic structure

Realm:	**Terrestrial**	**Subterranean**	**Freshwater**	**Marine**	**Atmospheric**
Ecosystem engineers	sessile autotrophs engineer biogenic structure & influence light, water & nutrients in most systems, animals manipulate structure in some systems	rarely influential	sessile plants and fish engineer structure and resources influencing oxygen & light (via turbidity)	benthic autotrophs and consumers influence biogenic structure and sediment structure	unlikely to be influential
Mutualisms & symbioses	host-dependent interactions influential on survival & reproduction in most systems	rarely influential	host-dependent interactions influential on survival & reproduction in some systems	host-dependent interactions influential on survival & reproduction in many systems	unlikely to be influential
Detritivory	invertebrate detritivores prominent in the soils of most systems, nutrient and carbon cycling	invertebrate detritivores prominent in resource-rich hotspots, nutrient & carbon cycling	vertebrate and invertebrate detritivores mainly on benthos, nutrient & carbon cycling	vertebrate and invertebrate detritivores mainly on benthos, major component in some deep and transitional systems, nutrient & carbon cycling	mostly absent

Realm:	**Terrestrial**	**Subterranean**	**Freshwater**	**Marine**	**Atmospheric**
Decomposition	fungal, archaean and bacterial decomposers prominent in soils of most systems, nutrient & carbon cycling	archaean and bacterial decomposers are major ecosystem components, nutrient & carbon cycling	archaean and bacterial decomposers, nutrient & carbon cycling	archaean and bacterial decomposers, abundant in some deep systems, nutrient & carbon cycling	unlikely to be influential cf. chemical decomposition
**Anthropogenic filters**					
Structural transformation	vegetation & landform transformation via land use, excavation, ploughing, construction	structural transformation via tunnels & mines	bank, channel & benthos transformation via canalisation, dredging & earthworks	transformation of biogenic, shoreline & benthic structure via engineering, bottom trawling, dredging, marine mining & dumping	not applicable
Water extraction, diversion & impoundment	sediment redistribution; desiccation or flooding	desiccation or flooding	altered flow & filling regimes	reduced freshwater inflow to coastal systems	not applicable
Pollution	nutrient & toxin release, night lights	artificial light, nutrient & toxin seepage	eutrophication, toxin release, increased turbidity, reduced light penetration	eutrophication & toxin release, micro- & macroplastics, increased turbidity	release of greenhouse gases, particulates, toxins, CFCs

Realm:	**Terrestrial**	**Subterranean**	**Freshwater**	**Marine**	**Atmospheric**
Resource extraction	mineral extraction	mineral extraction	see water extraction; sand/rock extraction	solute extraction, seafloor mining	not applicable
Appropriation of biomass and productivity	hunting, timber extraction, plant harvesting	negligible	Fishing, hunting, plant harvesting	fishing, gathering, hunting, seaweed harvest	Not applicable
Assisted biotic migration	managed & incidental translocations of organisms & propagules	incidental introductions of microbes & invertebrates	managed & incidental translocations of organisms & propagules	mostly incidental translocations via shipping or aquaculture	incidental movement mostly of propagules, e.g. via convection of urban heat
Climate change	warming and alteration of precipitation patterns, increased variability and extreme events	limited influence	altered flow & filling regimes, warming, marine incursions	warming sea surface, reduced oxygen, ocean acidification, altered circulation, upwelling processes & nutrient cycling, sea-level rise, increased storm frequency	warming & altered air circulation, increased frequency & intensity of extreme weather
**Ecosystem properties**					
Energy Sources	mostly autochthonous	allochthonous	mixed	mixed, but allochthonous at depth	allochthonous
Trophic structure	autotrophs support multiple heterotrophic levels; complexity varies with resources	truncated, no autotrophs, few predators, weak interactions	complexity varies with water body size	complexity varies along depth & resource gradients	simple & truncated, weak trophic interactions

Realm:	**Terrestrial**	**Subterranean**	**Freshwater**	**Marine**	**Atmospheric**
Productivity	varies greatly with resource gradients and temporally in some systems	low and stable, but varies with connectivity	varies greatly with resource gradients and temporally with flow & filling regimes	varies greatly with resource gradients and ocean circulation	low and variable with weather
Autotroph traits	complex differentiated organs and phenology	microbial or absent	mostly simple organisms or unicellular, specialised organisms in some systems	mostly simple organisms or unicellular	microbial or absent
Biogenic structure	complex & vertically stratified in most systems, but complexity varies along resource gradients	simple biofilms	simple vertical structure mostly with one, sometimes more strata: biofilm, submerged, floating, emergent	complex plant or animal foundations in some benthic systems, soft sediments mostly with simple structure	absent
Heterotroph diets	heterotrophs with diverse diets	detritivores & decomposers dominant, few predators	heterotrophs with diverse diets	heterotrophs with diverse diets	few, but specialised predators
Body sizes	small-large	small only	small-medium	small-very large	small only
Phenology	seasonal growth, mortality & reproduction in many systems, deciduous organs in some systems	limited seasonality influenced by connectivity to surface	life histories cued to seasonality of flows and filling regimes	seasonality of currents on surface drives variation in productivity throughout	seasonal winds & precipitation influence dispersal

Realm:	**Terrestrial**	**Subterranean**	**Freshwater**	**Marine**	**Atmospheric**
Salinity tolerance & regulation	osmotic regulation in rare cases	osmotic regulation in some species	salt exclusion & excretion, osmotic regulation increasing along salinity gradients	osmotic regulation pervasive across all taxa	not applicable
Water conservation	diverse morphological, dietary, digestive, life-history, physiological & behavioural traits	physiological traits in some taxa	diverse morphological, dietary, digestive, life-history, physiological & behavioural traits in systems with intermittent water	few taxa with traits except on shorelines	morphological traits to promote water retention
Buoyancy	few traits except in propagules of some species	few species with traits except aquatic vertebrates	many species with morphological & physiological water-buoyancy traits	many species with morphological & physiological water-buoyancy traits	many species with morphological air-buoyancy traits

Functional biomes

The biome concept evolved from its original application to continental-scale vegetation units associated with major climate types, to units that reflect functional and evolutionary processes, albeit still with an emphasis on terrestrial vegetation (Mucina 2018). Here, we adopt elements of the functional biome concept proposed by Moncrieff et al. (2016) and generalised by Mucina (2018). We took a deductive approach to definition of units, using our conceptual model to focus *a priori* on convergent ecosystem functions, ecological processes, structural properties and species traits that are shaped by one or more dominant assembly processes (Fig. 1 in main text; Table S3.2).

The focus on ecosystem properties and the ecological drivers that shape them, albeit qualitative, enabled us to extend the traditional scope of the biome concept from vegetation-climate relationships on land (Ricklefs & Relye 2018) to the entire biosphere. For example, functional distinctions justify recognition of different biomes on marine shelves and the deep sea floor. Marine shelves host diverse photoautotroph communities of benthic macrophytes, epiphytic algae, planktonic algae and symbiotic associations of algae and coral, compared to the deep sea floor, where a lack of sunlight precludes the existence of all photoautotrophs. The pelagic ocean waters, with exclusively planktonic autotrophs that diminish with depth and sunlight penetration define a third functionally distinctive biome in the oceans. Conceptually, such distinctions among ecosystems are analogous to those between traditionally recognised terrestrial biomes, such as tropical forests and deserts that show marked contrasts in structural features of their autotroph communities shaped by major differences in the availability of water. In both cases, the functional differences between biomes is founded on a diagnosis of the major assembly filters, expressed in landscapes and seascapes as gradients, that come to the fore in shaping functional properties of the ecosystems. Although the ultimate outcomes in structuring the typology involve subjective judgements, the common conceptual model provides a clear framework for justifying the recognition of separate biomes.

The 25 biomes recognised in v2.1 of the IUCN Global Ecosystem Typology are described in Appendix S4 (pp36-168), with updates published at https://global-ecosystems.org/. These are assigned to respective core and transitional realms (Fig. S3.3). As noted above, many conform to ‘traditional’ terrestrial biomes (e.g. Whittaker 1975; Ricklefs & Relye 2018) because of the close interrelations between functional traits and plant dominant growth forms. Other functionally distinctive groupings fall outside the traditional scope of the biome concept, including lentic and lotic freshwater biomes, pelagic and benthic marine biomes, and several anthropogenic biomes.

Ecosystem Functional Groups

Delineation of Ecosystem Functional Groups (Level 3) was based on gradients in key assembly filters for each biome and major ecosystem properties that vary along them (Table S3.2). Initial identification of key gradients and candidate functional groups was done by expert working groups for each realm, and later refined by input from an extensive network of ecosystem specialists (see Appendix S5 for development and review process). In terrestrial environments, key assembly gradients include water deficit, seasonality, temperature, nutrient availability, fire activity and herbivory (Table S3.2). In subterranean environments, substrate structure and connectivity to surface systems are major factors. In freshwater environments, gradients in flow continuity and velocity, water body size, seasonal freezing and salinity are

key assembly filters. In marine environments, depth gradients in light, vertical and horizontal movement of nutrients and substrate stability and particle size are major assembly filters.

An example for tropical forests (Fig. S3.4) shows how candidate functional groups were delineated by identifying distinctive sets of traits associated with particular segments of the assembly filter space defined by the gradients. All Ecosystem Functional Groups were delineated using reasoning based on our conceptual model of ecosystem assembly (Fig. 1 in main text).

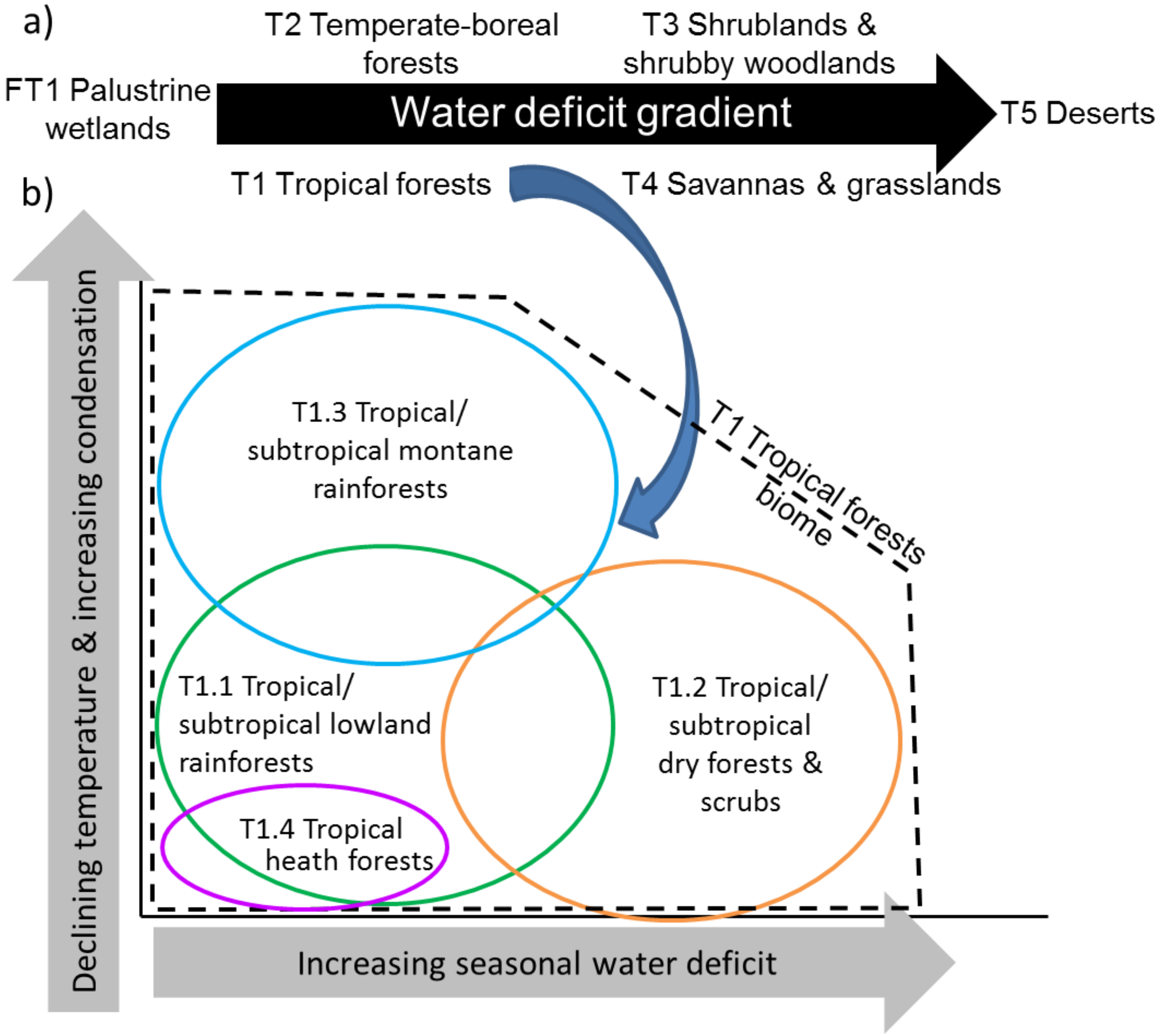

Figure S3.4. a) Relationships of terrestrial biomes to a major assembly filter represented by a water deficit gradient (five of seven terrestrial biomes and one freshwater-terrestrial biome shown). b) Elaborated relationships of four Ecosystem Functional Groups within the Tropical forests biome (T1) to assembly filters represented by two environmental gradients. A third filter related to an edaphic environmental gradient differentiates group T1.4 from T1.1, but is not shown here (see Appendix S4 for details on respective functional groups). See also Fig. 3 in main text.

In the Terrestrial realm, variation in ecosystem properties is closely related to a water deficit gradient (Fig S3.4a). Water surplus occurs at the interface with the Freshwater realm, an environmental space occupied by the Palustrine Wetlands biome. Several biomes replace one another along the gradient of increasing water deficit, with the Tropical/subtropical forests biome occupying a mesic segment of this gradient. Within that biome, we identified seasonal water deficit and temperature as two specific assembly filters that enable recognition of three functional groups of forest characterised by distinctive combinations of properties (Fig. S3.4b). Tropical/subtropical lowland rainforests (Ecosystem Functional Group T1.1) develop under year-round water surplus and consistently warm temperatures (limited frost incidence). They are characterised by high diversity and endemism, a dense evergreen tree canopy, high structural complexity and several other properties (Appendix S4 p37 for details). Tropical heath forests (EFG T1.4) occupy a similar, but more restricted hydrological and thermal niche with respect to the environmental gradients in Fig. S3.4b, but are differentiated by a third assembly filter related to a gradient in soil acidity and nutrient status (not shown in Fig. S3.4b). As the seasonal water deficit increases, these humid forests are replaced by Tropical/subtropical dry forests (EGF T1.2). The distinctive traits of these dry forests include seasonal phenology (including deciduousness), reduced leaf area, and scarcity of certain growth forms such as ferns and bryophytes, etc. (Appendix S4 for details). A fourth Ecosystem Functional Group (T1.3) occurs in tropical montane environments that experience cooler temperatures and regular fogs and frosts. These forests are distinguished by their simpler evergreen tree canopies with a smaller range of leaf sizes, high epiphyte loading, an abundance of ferns and bryophytes, high local endemism and lower plant and animal diversity (Appendix S4 pp41-44 for details).

For each of the 110 Ecosystem Functional Groups recognised in v2.1 of the typology, we represented the key drivers and traits in simplified conceptual models derived from Fig. 1 as a basis for review and description. We adjusted and augmented descriptions based on published reviews (see cited references in the descriptive profiles, Appendix S4) and in consultation with extensive networks of specialists (Appendix S5). Finally, we prepared indicative global distribution maps from available spatial data (see Appendix S4 pp15-16 and Table S4.1 for full details of map compilation for each functional group).

Lower levels of classification

The three lower levels of the typology were designed to represent different compositional expressions of functionally convergent groups of ecosystems. They are being developed progressively through two contrasting approaches that involve different trade-offs, strengths and weaknesses. Firstly, Level 4 units (Regional ecosystem subgroups) are being developed from the top-down, by subdividing Ecosystem Functional Groups using an ecoregional template. Ecoregionalisations (e.g. Spalding et al. 2007; Abell et al. 2008; Dinerstein et al. 2017) serve as simple and accessible proxies for biotic composition based on biogeographic boundaries and have recently been shown to delineate biodiversity patterns effectively, at least on land (Smith et al. 2018). The efficacy of spatial proxies for biodiversity is underpinned by niche differentiation and variation in species' interactions along regional gradients, insularity and historical legacies of vicariance and dispersal (Chase 2003; Maestre et al. 2009; Nekola & White 2009). Assuming 2 to 5 functional groups may be represented in each ecoregion, we estimate 3,000-7,500 units could be identified across the biosphere at Level 4. An important limitation of this approach is that ecoregional proxies impose the same fixed spatial template across all Ecosystem Functional Groups. This lack of flexibility limits the scope for adjustment of units even where a more evidence-based delineation of units is possible.

The second approach addresses this problem by developing Levels 5 and 6 of the typology (global and subglobal ecosystem types, respectively) from the bottom-up. In practice, subglobal ecosystem types will be based on established local classifications (e.g. Mucina & Rutherford 2006). These benefit from the abundance of direct observational data and expertise available at local scales. Incorporating these classifications into a global framework acknowledges the value of substantial investments in data acquisition and development, as well as the integration of these classifications into policy instruments and management plans. Improved local accuracy and precision, however, are traded off against inconsistencies that inevitably exist among local classifications based on different data sets and methods of construction. Subglobal ecosystem types will be aggregated into Global ecosystem types (Level 5) based on compositional similarities. This will help resolve some of the inconsistencies between different classifications. In turn, Global ecosystem types (Level 5) will be assigned to Ecosystem Functional Groups (Level 3) based on their key ecological traits and drivers. In Tables S3.3 and S3.4, we give two examples where experts assigned Level 6 units from national classifications to global Level 3 Ecosystem Functional Groups by matching descriptions of respective units.

As noted previously, Level 5 units are not nested within Level 4 units because they are alternative pathways for representation of variation in biotic composition within Ecosystem Functional Groups (Level 3). The more complex, non-nested relationship between units of Levels 4 and 5, including one to many, many to one and one to one, could ultimately be mapped in a cross-walk based on methods discussed below.

Reflections on the approach to typology development

Theoretical foundations

The biome concept has terrestrial foundations, with a focus on global climatic relationships, vegetation structure and physiognomy (Walter 1973; Mucina 2018). Broadening its scope to encompass a more diverse suite of properties and selection filters in the context of assembly theory (Fig. 1) allows a logical extension of this powerful concept to freshwater and marine realms (Dodds et al. 2019). Our model of ecosystem assembly offers a deductive framework to identify the key ecological processes that sustain the identity of ecosystems characterised by different properties.

Some biomes and Ecosystem Functional Groups that we identified through our approach are difficult to reconcile with traditional climate-centric biome concepts (Moncrieff et al. 2016). For example, Bond et al. (2005) were unable to predict the global savanna distribution from climate in the absence of fire, while Orians & Milewski (2007) highlighted the profound influence of nutrient poverty, despite climatic variation, in structuring traits and functions of some shrub-dominated and temperate forest systems recognised at Level 3 of our typology.

Strong theoretical foundations of our ecosystem typology should also make the overall framework robust to advances in data quality and availability. Nonetheless, adjustments to the circumscriptions and descriptions of units in version 2.1 (Appendix S4) will be needed as our knowledge of assembly processes improves for particular types of ecosystems. Notably, ecosystem processes and variation in traits within the atmospheric realm need further research before that component of the typology can be resolved.

Top-down and bottom-up construction

Our framework combines top-down and bottom-up approaches to classification in a manner that seeks to balance consistency and realism (Fig. S3.1). A top-down approach, subdividing functionally contrasting biomes within realms and functional groups within biomes, is guided by assembly theory. The dual approach to building the typology from the top-down and bottom-up is critical to consistent and comprehensive global coverage (Principle 3; Table S1.1), to integrating both ecosystem function and composition (Principles 1 and 2) into a single classificatory framework, and for scalability to different applications (Principle 4). A bottom-up approach to grouping compositionally similar units at lower levels and assigning them to functional groups promotes realism by incorporating local data and expertise. This flexibility to define compositional relationships from the bottom-up is critical to utility, local ownership and wide use of the typology (Principle 6) because: i) expertise and data on compositional relationships reside primarily at national and subnational levels; and ii) ecosystem management and biodiversity conservation is implemented through locally-based on-ground action.

The combination of top-down and bottom-up approaches to construction of the typology has two advantages. First, it enables explicit linkages with finer scale classifications that catalogue local expressions of global biomes and functional groups. Second, it provides two alternative options for global analysis based on Levels 4 and 5, which have complementary strengths and weaknesses. Concordance between these options allows inferences to be drawn that are robust to uncertainties in ecosystem classification, while discordance highlights a need for additional data to resolve uncertainties.

Effective coupling of top-down and bottom-up units of classification requires Global ecosystem types (Level 5) to be assigned to Ecosystem Functional Groups (Level 3), either quantitatively or qualitatively, using methods designed to handle inherent uncertainty. Quantitative methods such as fuzzy sets (De Caceras et al. 2010) and model-based approaches (Lyons et al. 2016) can be applied if suitable attribute data are available. Where formal data are lacking, structured expert elicitation provides a repeatable method to reduce subjective biases in cross-walking classifications (Hemming et al. 2018).

Dealing with anthropogenic influences on ecosystems

Human activity influences assembly of almost all ecosystems. Erb et al. (2017), for example, defined 10 different land management activities, quantified their variation in intensity, and estimated global-scale biophysical and biogeochemical effects on terrestrial ecosystems. The effects play out through complex interactions and feedbacks between ecosystems and socio-economic systems with varied settings for labour and capital inputs, technology, market dynamics, cultural beliefs, business or subsistence decision making and geopolitics (Meyfroidt et al. 2016). Erb et al. (2017) estimated that intensive land management activities occur on about 10% of Earth's ice-free terrestrial surface based on subjectively thresholded metrics for each activity. Haberl et al. (2007) estimated that humans appropriate >40% of Net Primary Productivity over ~20% of Earth's ice-free terrestrial surface.

These anthropogenic influences create challenges for ecosystem classification, as they may influence defining features of the units to a degree that varies from negligible to transformative across different locations and times. We addressed this problem by distinguishing transformative outcomes of human activity (i.e. due to qualitative changes in land/sea use type) from other influences that relate to land use intensification (increases in multidimensional inputs or outputs of socio-economic systems, or both) within broadly

defined ecosystem units (Erb et al. 2013). We thus distinguished anthropogenic ecosystems conceptually as those that are created and sustained by intensive human activities.

Examples of ecosystems that depend on ongoing intensive human activity for their maintenance include annual cropfields, which when abandoned have transformed to semi-natural grasslands or secondary forests (Foster & O'Keefe 2000), and cities, which when abandoned were transformed to secondary forests (Islebe et al. 1996). Both transformations occurred through socio-ecological feedbacks. Within the structure of the typology we thus defined broad groups of anthropogenic ecosystems at Levels 2 (biomes) and 3 (Ecosystem Functional Groups). In contrast, some anthropogenic ecosystems are sustained by legacies that persist long after their establishment. For example, many small water reservoirs established by humans such as mill ponds no longer retain their intensive use, but nonetheless persist, albeit with some changes to their properties. Artificial reefs may similarly persist long after their initial structure is established by humans.

By focussing descriptions of non-anthropogenic ecosystem types on reference states with negligible influence of human activity, our approach enables the degree and nature of human influence to be described and assessed against these reference states using appropriate methods such as the Red List of Ecosystems protocol (Keith et al. 2013), productivity appropriation (Haberl et al. 2007), restoration standards (McDonald et al. 2016), etc.

Discrete representation of continuous patterns in nature

Like any ecological classification, our global ecosystem typology artificially compartmentalises natural continua (Keith 2015; Riesch et al. 2018). While we recognise continua between units of classification (Figs. S3.3, S3.4), they generate uncertainties related to 'vagueness', a form of linguistic uncertainty pertaining to boundary cases in which there is no objective, determinate way to resolve whether objects belong to one category or another (Regan et al. 2002). Vagueness, which also occurs in taxonomic classifications, is reducible through lucid descriptions, but cannot be eliminated. Constraints related to vagueness are especially relevant to representation of spatial patterns in maps (Ferrier et al. 2007), as there is no ultimate truth in precise location of boundaries in a fundamentally continuous world.

Practitioners nevertheless find the abstraction of nature in a classification cognitively attractive for interpretation and communication of patterns and relationships. While gradient-based methods of representing nature are available (Legendre & Legendre 2012) and have advantages for particular applications (Ferrier et al. 2007), they have not gained traction across the full range of applications that classificatory frameworks have, especially among non-scientific audiences. Classification is deep-seated in human nature and cognition (Pirsig 1974, p85). Hence, classifications are used widely across environmental, social, economic and cultural dimensions of human activity, despite the underlying continuity of variation in the properties of interest. We therefore considered that the benefits of wide uptake outweighed the limitations of adopting a discrete model of ecosystem variation.

References

Abell R, Thieme ML, Revenga C, Bryer M, Kottelat M, Bogutskaya N, Coad B, Mandrak N, Contreras Balderas S, Bussing W, Stiassny MLJ, Skelton P, Allen GR, Unmack P, Naseka A, Ng R, Sindorf N, Robertson J, Armijo E, Higgins JV, Heibel TJ, Wikramanayake E, Olson D, López HL, Reis RE, Lundberg JG, Sabaj Pérez MH, Petry P (2008) Freshwater ecoregions of

the world: A new map of biogeographic units for freshwater biodiversity conservation. BioScience 58: 403–414. [https://doi.org/10.1641/B580507]

Bond WJ, Woodward FI, Midgley GF (2005) The global distribution of ecosystems in a world without fire. New Phytologist 165: 525-538. [doi: 10.1111/j.1469-8137.2004.01252.x]

Bunn SE, Arthington AH (2002) Basic principles and ecological consequences of altered flow regimes for aquatic biodiversity. Environmental Management 30: 492–507. [doi: 10.1007/s00267-002-2737-0]

Carney RS (2005) Zonation of deep biota on continental margins. In: Oceanography and Marine Biology - An annual review vol 43 (Eds. R Gibson, J Gordon, R Atkinson), pp211-278. Taylor & Francis, Boca Raton. [doi: 10.1201/9781420037449.ch6]

Chase JM (2003) Community assembly: When should history matter? Oecologia 136: 489–498.

Cross WF, Benstead JP, Frost PC, Thomas SA (2005) Ecological stoichiometry in freshwater benthic systems: recent progress and perspectives. Freshwater Biology 50: 1895-1912. [doi: 10.1111/j.1365-2427.2005.01458.x]

Cury P, Bakun Andrew, Crawford RJM, Jarre A, Quiñones RA, Shannon LJ, Verheye HM (2000) Small pelagics in upwelling systems: Patterns of interaction and structural changes in “wasp-waist” ecosystems. ICES Journal of Marine Science 57: 603–618. [doi: 10.1006/jmsc.2000.0712]

De Cáceres M, Font X, Oliva F (2010) The management of vegetation classifications with fuzzy clustering. Journal of Vegetation Science 21: 1138-1151.

Dinerstein E, Olson D, Joshi A, Vynne C, Burgess ND, Wikramanayake E, Hahn N, Palminteri S, Hedao P, Noss R, Hansen M, Locke H, Ellis EC, Jones B, Barber CV, Hayes R, Kormos C, Martin V, Crist E, Sechrest W, Price L, Baillie JEM, Weeden D, Suckling K, Davis C, Sizer N, Moore R, Thau D, Birch T, Potapov P, Turubanova S, Tyukavina A, de Souza N, Pintea L, Brito JC, Llewellyn OA, Miller AG, Patzelt A, Ghazanfar SA, Timberlake J, Klöser H, Shennan-Farpón Y, Kindt R, Lillesø JB, van Breugel P, Graudal L, Voge M, Al-Shammari KF, Saleem M (2017) An ecoregion-based approach to protecting half the terrestrial realm. Bioscience 67:534-545. [doi: 10.1093/biosci/bix014]

Ellis EC, Goldewijk KK, Siebert S, Lightman D, Ramankutty N (2010) Anthropogenic transformation of the biomes, 1700 to 2000. Global Ecology and Biogeography 19: 589–606.

Engel AS (2010) Microbial diversity of cave ecosystems. In ‘Geomicrobiology: Molecular and environmental perspective.’ (Eds LL Barton, M Mandl, A Loy) pp. 219–238. Springer, Dordrecht.

Erb KH, Haberl H, Jepsen MR, Kuemmerle T, Lindner M, Müller D, Verburg PH, Reenberg A (2013) A conceptual framework for analysing and measuring land-use intensity. Current Opinion in Environmental Sustainability 5: 464–470. [doi: 10.1016/j.cosust.2013.07.010]

Erb K, Luyssaert S, Meyfroidt P, Pongratz J, Don A, Kloster S, Kuemmerle T,Fetzel T, Fuchs R, Herold M, Haberl H, Jones CD, Marin-Spiotta, E, McCallum I, Robertson E, Seufert V, Fritz S, Valade A, Wiltshire A, Dolman AJ (2017) Land management: data availability and process understanding for global change studies. Global Change Biology 23: 512-533. [doi:10.1111/gcb.13443.]

Ferrier S, Manion G, Elith J, Richardson K (2007) Using generalized dissimilarity modelling to analyse and predict patterns of beta diversity in regional biodiversity assessment. Diversity and Distributions 13: 252-264.

Foster DR, O'Keefe JF (2000) New England forests through time: Insights from the Harvard Forest dioramas. Harvard University Press, Cambridge.

Frank KT, Petrie B, Shackell NL (2007) The ups and downs of trophic control in continental shelf ecosystems. Trends In Ecology & Evolution 22, 236-242 [doi: 10.1016/j.tree.2007.03.002]

Fröhlich-Nowoisky J, Kampf CJ, BettinaWeber, Huffman JA, Pöhlker C, Andreae MO, Lang-Yona N, Burrows SM, Gunthe SS, Elbert W, Su H, Hoor P, Thines E, Hoffmann T, Després VR, Pöschl U (2016) Bioaerosols in the Earth system: climate, health and ecosystem interactions. Atmospheric Research 182: 346– 376. [doi: 10.1016/j.atmosres.2016.07.018]

Gibert J, Deharveng L (2002) Subterranean ecosystems: A truncated functional biodiversity. BioScience 52: 473–481.

Guisan A, Zimmermann NE (2000) Predictive habitat distribution models in ecology. Ecological Modelling 135: 147-186. https://doi.org/10.1016/S0304-3800(00)00354-9

Haberl H, Erb KH, Krausmann F, Gaube V, Bondeau A, Plutzar C, Gingrich S, Lucht W, Fischer-Kowalski, M (2007) Quantifying and mapping the human appropriation of net primary production in earth's terrestrial ecosystems. Proceedings of the National Academy of Sciences of the United States of America 104: 12942-12945. [doi: 10.1073/pnas.0704243104]

Harris PT, Macmillan-Lawler M, Rupp J, Baker EK (2014) Geomorphology of the oceans. Marine Geology 352: 4-24.

Hemming V, Burgman MA, Hanea AM, McBride MF, Wintle BC (2018) A practical guide to structured expert elicitation using the IDEA protocol. Methods in Ecology and Evolution 9: 169-180.

Islebe, G A; Hooghiemstra, H; Brenner, M; Curtis, JH; Hodell, DA (1996) A Holocene history from lowland Guatamala. The Holocene 6: 265-271.

Jeppesen E, Jensen JP, Søndergaard M, Lauridsen T, Pedersen LJ, Jensen L (1997) Top-down control in freshwater lakes: The role of nutrient state, submerged macrophytes and water depth. Hydrobiologia 342–343: 151–164.

Keith DA (2015) Assessing and managing risks to ecosystem biodiversity. Austral Ecology 40: 337-346. [doi:10.1111/aec.12249]

Legendre P, Legendre L (2012) Numerical ecology. Developments in environmental modelling vol. 24. Elsevier, Amsterdam.

Lyons M, Keith DA, Warton D, Somerville M, Kingsford RT, De Cáceres M (2016). Model-based assessment of ecological community classifications. *Journal of Vegetation Science* **27**, 704-715.

Maestre FT, Callaway RM, Valladares F, Lortie CJ (2009) Refining the stress-gradient hypothesis for competition and facilitation in plant communities. Journal of Ecology 97: 199-205. [doi.org/10.1111/j.1365-2745.2008.01476.x]

Maranon E, Holligan PM, Barciela R, Gonzalez N, Mourino B, Pazo MJ, Varela, M (2001) Patterns of phytoplankton size structure and productivity in contrasting open-ocean environments. Marine Ecology Progress Series 216: 43-56. [doi: 10.3354/meps216043]

McDonald T, Jonson J, Dixon KW (2016) National standards for the practice of ecological restoration in Australia. Restoration Ecology 24: S6-S32. [doi: 10.1111/rec.12359]

Meyfroidt P, Roy Chowdhury R, de Bremond A, Ellis EC, Erb KH, Filatova T, Garrett RD, Grove JM, Heinimann A, Kuemmerle T, Kull CA, Lambin EF, Landon Y, le Polain de Waroux Y, Messerli P, Müller D, Nielsen J, Peterson GD, Rodriguez García V, Schlüter M, Turner II BL, Verburg PH (2018) Middle-range theories of land system change. Global Environmental Change 53: 52-67. [doi: 10.1016/j.gloenvcha.2018.08.006]

Millero FJ, Feistel R, Wright DG, McDougall TJ (2008) The composition of standard seawater and the definition of the reference-composition salinity scale. Deep-sea Research Part I-oceanographic Research Papers 55: 50-72. [doi: 10.1016/j.dsr.2007.10.001]

Moncrieff GR, Bond WJ, Higgin SI (2016) Revising the biome concept for understanding and predicting global change impacts. Journal of Biogeography 43, 863–873.

Mucina L (2018) Biome: evolution of a crucial ecological and biogeographical concept. New Phytologist 222: 97-114, doi:10.1111/nph.15609.

Mucina L, Rutherford MC (2006) The Vegetation of South Africa, Lesotho and Swaziland. Strelitzia 19, South African National Biodiversity Institute, Pretoria.

Murray NJ, Phinn SR, DeWitt M, Ferrari R, Johnston R, Lyons MB, Clinton N, Thau D, Fuller RA (2019) The global distribution and trajectory of tidal flats. Nature 565: 222–225.

Nekola JC, White PS (1999) The distance decay of similarity in biogeography and ecology. Journal of Biogeography 26: 867-878.

Orians GH, Milewski AV (2007) Ecology of Australia: the effects of nutrient-poor soils and intense fires. Biological Reviews 82: 393-423.

Pirsig RM (1974) Zen and the art of motorcycle maintenance: an inquiry into values. Vintage, London.

Polis GA (1999) Why Are Parts of the World Green? Multiple Factors Control Productivity and the Distribution of Biomass. Oikos 86: 3-15. [doi: 10.2307/3546565]

Pöschl U, Martin ST, Sinha B, Chen Q, Gunthe SS, Huffman JA, Borrmann S, Farmer DK, Garland RM, Helas G, Jimenez JL, King SM, Manzi A, Mikhailov E, Pauliquevis T, Petters MD, Prenni AJ, Roldin P, Rose D, Schneider J, Su H, Zorn SR, Artaxo P, Andreae MO (2010) Rainforest aerosols as biogenic nuclei of clouds and precipitation in the Amazon. Science 329, 1513–1516. [doi: 10.1126/science.1191056]

Prentice IC, Cramer W, Harrison SP, Leemans R, Monserud RA, Solomon AM (1992) A Global Biome Model Based on Plant Physiology and Dominance, Soil Properties and Climate. Journal of Biogeography 19: 117-134. [doi: 10.2307/2845499]

Rabalais NN, Diaz, RJ, Levin LA, Turner RE, Gilbert, D Zhang J (2010) Dynamics and distribution of natural and human-caused hypoxia. Biogeosciences 7: 585-619. [doi: 10.5194/bg-7-585-2010]

Regan HM, Colyvan M, Burgman MA (2002) A taxonomy and treatment of uncertainty for ecology and conservation biology. Ecological Applications 12: 618-628.

Reisch R, Plath M, Bierbach D (2018) Ecology and evolution along environmental gradients. Current Zoology 64: 193–196.

Ricklefs RE, Relye R (2018) Ecology: The economy of nature. WH.Freeman & Co, New York.

Smith JR, Letten AD, Ke PJ, Anderson CB, J. Hendershot N, Dhami MK, Dlott GA, Grainger TN, Howard ME, Morrison BML, Routh D, San Juan PA, Mooney HA, Mordecai EA, Crowther TW, Daily GC (2018) A global test of ecoregions. Nature Ecology & Evolution 2: 1889–1896.

Seneviratne SI, Corti T, Davin EL, Hirschi M, Jaeger EB, Lehner I, Orlowsky B, Teuling AJ (2010) Investigating soil moisture-climate interactions in a changing climate: A review. Earth Science Reviews 99: 125-161. [doi: 10.1016/j.earscirev.2010.02.004]

Schlacher TA, Newell P, Clavier J, Schlacher-Hoenlinger MA, Chevillon C, Britton J (1998) Soft-sediment benthic community structure in a coral reef lagoon - the prominence of spatial heterogeneity and 'spot endemism'. Marine Ecology Progress Series 174: 159-174. [doi: 10.3354/meps174159]

Spalding MD, Fox HE, Allen GR, Davidson N, Ferdaña ZA, Finlayson M, Halpern BS, Jorge MA, Lombana A, Lourie SA, Martin KD, McManus E, Molnar J, Recchia CA, Robertson J (2007) Marine ecoregions of the world: A bioregionalization of coastal and shelf areas, BioScience 57: 573–583. [doi: 10.1641/B570707]

Thorp JH, Thoms MC, Delong MD (2006) The riverine ecosystem synthesis: Biocomplexity in river networks across space and time. River Research and Applications 22: 123-147. [doi: DOI: 10.1002/rra.901]

Todd RE, Chavez FP, Sophie C et al. (2019) Global perspectives on observing ocean boundary current systems. Frontiers in Marine 6: 423. [doi: 10.3389/fmars.2019.00423]

Vitousek PM, Porder S, Houlton BZ, Chadwick OA (2010) Terrestrial phosphorus limitation: mechanisms, implications, and nitrogen–phosphorus interactions. Ecological Applications 20: 5-15. [doi: 10.1890/08-0127.1]

Walter H (1973) Vegetation of the earth. Elsevier, London.

Whittaker RH (1975) Communities and ecosystems. 2nd Revised edition. MacMillan, New York.

Williams CG, Smith DJ (2021) Unifying atmospheric biology research for the U.S. scientific community. Ecological Applications 31: e02275. [doi: 10.1002/eap.2275]

Table S3.3. Cross-walk of the national vegetation classification of Chile (Luebert & Pliscoff 2017) with global Ecosystem Functional Groups.

Biome	Ecosystem Functional Group	map unit #	Vegetation belt (Luebert & Pliscoff 2017)
T1 Tropical & subtropical forests			
	T1.2 Tropical/Subtropical dry forests and scrubs		
		27	Inland mediterranean thorny scrub of Trevoa quinquinervia - Colliguaja odorifera
		28	Inland mediterranean thorny scrub of Puya coerulea - Colliguaja odorifera
		29	Inland tropical thorny forest of Prosopis tamarugo / Tessaria absinthiodes
		30	Inland tropical thorny forest of Geoffroea decorticans - Prosopis alba
		31	Andean tropical thorny forest of Browningia candelaris - Corryocactus brevistylus
		32	Inland mediterranean thorny forest of Acacia caven - Prosopis chilensis
		33	Andean mediterranean thorny forest of Acacia caven / Baccharis paniculata
		34	Coastal mediterranean thorny forest of Acacia caven - Maytenus boaria
		35	Inland mediterranean thorny forest of Acacia caven - Lithraea caustica
T2 Temperate-boreal forests & woodlands			
	T2.3 Oceanic temperate rainforests		
		46	Coastal mediterranean deciduous forest of Nothofagus macrocarpa / Ribes punctatum
		47	Inland mediterranean deciduous forest of Nothofagus obliqua - Cryptocarya alba
		48	Andean mediterranean deciduous forest of Nothofagus obliqua - Austrocedrus chilensis
		49	Coastal mediterranean deciduous forest of Nothofagus glauca - Azara petiolaris
		50	Coastal mediterranean deciduous forest of Nothofagus glauca - Persea lingue
		51	Andean mediterranean deciduous forest of Nothofagus glauca - N. obliqua
		52	Coastal mediterranean-temperate deciduous forest of Nothofagus obliqua - Gomortega keule
		53	Mediterranean deciduous forest of Nothofagus obliqua - Persea lingue
		54	Temperate deciduous forest of Nothofagus obliqua - Laurelia sempervirens
		55	Coastal mixed mediterranean-temperate forest of Nothofagus dombeyi - N. obliqua
		56	Coastal temperate deciduous forest of Nothofagus alpina - Persea lingue
		57	Andean mediterranean-temperate deciduous forest of Nothofagus alpina - N. obliqua
		58	Andean temperate deciduous forest of Nothofagus alpina - Dasyphyllum diacanthoides
		59	Andean temperate deciduous forest of Nothofagus alpina - N. dombeyi
		60	Andean mediterranean-temperate deciduous forest of Nothofagus pumilio - N. obliqua
		61	Andean temperate deciduous forest of Nothofagus pumilio - Araucaria araucana
		62	Andean temperate deciduous forest of Nothofagus pumilio / Drimys andina
		63	Andean temperate deciduous forest of Nothofagus pumilio / Berberis ilicifolia
		64	Andean temperate deciduous forest of Nothofagus pumilio / Azara alpina
		65	Andean temperate deciduous forest of Nothofagus pumilio / Ribes cucullatum
		66	Andean temperate-antiboreal deciduous forest of Nothofagus pumilio / Maytenus disticha
		67	Andean temperate deciduous scrub of Nothofagus antarctica
		part 68	Andean temperate deciduous scrub of Nothofagus antarctica / Empetrum rubrum
		69	Mediterranean-temperate deciduous arborescent shrubland of Nothofagus antarctica / Berberis microphylla
		70	Andean temperate-antiboreal deciduous arborescent shrubland of Nothofagus antarctica / Chiliotrichum diffusum
		part 71	Coastal temperate broad-leaved forest of Aextoxicon punctatum - Laurelia sempervirens
		72	Coastal temperate broad-leaved forest of Weinmannia trichosperma - Laureliopsis philippiana
		73	Inland temperate broad-leaved forest of Nothofagus dombeyi - Eucryphia cordifolia
		74	Coastal temperate coniferous forest of Araucaria araucana
		75	Andean temperate coniferous forest of Araucaria araucana - Nothofagus dombeyi

Biome	Ecosystem Functional Group	map unit #	Vegetation belt (Luebert & Pliscoff 2017)
		76	Andean mediterranean-temperate coniferous forest of Araucaria araucana / Festuca scabriuscula
		77	Coastal temperate coniferous forest of Fitzroya cupressoides
		78	Andean temperate coniferous forest of Fitzroya cupressoides
		79	Coastal temperate coniferous forest of Pilgerodendron uviferum - Tepualia stipularis
		80	Coastal temperate coniferous forest of Pilgerodendron uviferum / Astelia pumila
		81	Andean temperate evergreen forest of Nothofagus dombeyi / Gaultheria phillyreifolia
		82	Andean temperate evergreen forest of Nothofagus dombeyi - Saxegothaea conspicua
		83	Andean temperate evergreen forest of Austrocedrus chilensis - Nothofagus dombeyi
		84	Inland temperate evergreen forest of Nothofagus nitida - Podocarpus nubigenus
		85	Inland temperate evergreen forest of Nothofagus betuloides / Desfontainia fulgens
		86	Andean temperate evergreen forest of Nothofagus betuloides - Laureliopsis philippiana
		87	Andean temperate evergreen forest of Nothofagus betuloides / Chusquea macrostachya
		88	Andean mixed temperate forest of Nothofagus betuloides / Berberis ilicifolia
		89	Andean mixed temperate-antiboreal forest of Nothofagus betuloides - Nothofagus pumilio
		90	Coastal antiboreal evergreen forest of Nothofagus betuloides - Embothrium coccineum
		91	Coastal temperate-antiboreal evergreen forest of Nothofagus betuloides - Drimys winteri
		92	Coastal temperate evergreen shrubland of Pilgerodendron uviferum - Nothofagus nitida
	T2.4 Warm temperate rainforests		
		part 71	Coastal temperate broad-leaved forest of Aextoxicon punctatum - Laurelia sempervirens
T3 Shrublands and shrubby woodlands biome			
	T3.2 Seasonally dry temperate heaths and shrublands		
		36	Coastal mediterranean sclerophyllous arborescent shrubland of Peumus boldus - Schinus latifolia
		37	Inland mediterranean sclerophyllous arborescent shrubland of Quillaja saponaria / Porlieria chilensis
		38	Andean mediterranean sclerophyllous forest of Kageneckia angustifolia / Guindilia trinervis
		39	Coastal mediterranean sclerophyllous forest of Cryptocarya alba - Peumus boldus
		40	Coastal mediterranean sclerophyllous forest of Lithraea caustica - Cryptocarya alba
		41	Andean mediterranean sclerophyllous forest of Quillaja saponaria - Lithraea caustica
		42	Coastal mediterranean sclerophyllous forest of Lithraea caustica - Azara integrifolia
		43	Inland mediterranean sclerophyllous forest of Lithraea caustica - Peumus boldus
		44	Andean mediterranean sclerophyllous forest of Lithraea caustica - Lomatia hirsuta
		45	Inland mediterranean sclerophyllous psamophil forest of Quillaja saponaria / Fabiana imbricata
	T3.3 Cool temperate heathlands		
		part 68	Andean temperate deciduous scrub of Nothofagus antarctica / Empetrum rubrum
T4 Savannas and grasslands			
	T4.5 Temperate grasslands		
		122	Western mediterranean-temperate steppe of Festuca pallescens / Mulinum spinosum
		123	Western mediterranean-temperate steppe of Festuca gracillima
		124	Western mediterranean-temperate steppe of Festuca gracillima / Mulinum spinosum
		125	Western temperate steppe of Festuca gracillima / Chiliotrichum diffusum
T5 Deserts and semi-deserts			
	T5.1 Semi-desert steppes		
		5	Inland tropical desert scrub of Atriplex atacamensis - Tessaria absinthioides
		23	Andean tropical desert dwarf scrub of Atriplex imbricata - Acantholippia deserticola
	T5.2 Thorny deserts and semi-deserts		
		6	Coastal tropical desert scrub of Nolana sedifolia / Eulychnia iquiquensis

Biome	Ecosystem Functional Group	map unit #	Vegetation belt (Luebert & Pliscoff 2017)
		7	Coastal tropical-mediterranean desert scrub of Ephedra breana / Eulychnia iquiquensis
		8	Coastal tropical-mediterranean desert scrub of Copiapoa boliviana - Heliotropium pycnophyllum
		9	Coastal mediterranean desert scrub of Gypothamnium pinifolium - Heliotropium pycnophyllum
		10	Coastal mediterranean desert scrub of Euphorbia lactiflua / Eulychnia iquiquensis
		11	Coastal mediterranean desert scrub of Euphorbia lactiflua / Eulychnia saint-pieana
		12	Inland mediterranean desert scrub of Oxyphyllum ulicinum - Gymnophyton foliosum
		13	Coastal mediterranean desert scrub of Heliotropium floridum - Atriplex clivicola
		14	Coastal mediterranean desert scrub of Oxalis virgosa / Eulychnia breviflora
		15	Inland tropical-mediterranean desert scrub of Skytanthus acutus - Atriplex deserticola
		16	Inland tropical desert scrub of Huidobria chilensis - Nolana leptophylla
		17	Coastal mediterranean desert scrub of Oxalis virgosa - Heliotropium stenophyllum
		18	Inland mediterranean desert scrub of Adesmia argentea - Bulnesia chilensis
		19	Inland mediterranean desert scrub of Heliotropium stenophyllum - Flourensia thurifera
		20	Inland mediterranean desert scrub of Flourensia thurifera - Colliguaja odorifera
		21	Coastal mediterranean desert scrub of Bahia ambrosioides / Puya chilensis
		22	Inland tropical desert dwarf scrub of Adesmia atacamensis - Cistanthe salsoloides
		24	Inland tropical desert dwarf scrub of Nolana leptophylla - Cistanthe salsoloides
		25	Andean tropical-mediterranean desert dwarf scrub of Atriplex imbricata
		26	Andean mediterranean desert dwarf scrub of Senecio proteus - Haplopappus baylahuen
	T5.5 Hyper-arid deserts		
		1	Inland tropical desert with scarce vegetation
		2	Coastal tropical dunes of Tillandsia landbeckii - T. marconae
		3	Coastal tropical desert scrub of Nolana adansonii - N. lycioides
		4	Inland tropical desert scrub of Malesherbia auristipulata - Tarasa operculata
T6 Polar-alpine (cryogenic)			
	T6.1 Ice sheets, glaciers and perennial snowfields		
		SV	Sin vegetacion
	T6.2 Polar/alpine rocky outcrops		
		SV	Sin vegetacion
	T6.4 Temperate alpine meadows and shrublands		
		108	Andean mediterranean-tropical dwarf scrub of Adesmia hystrix - Ephedra breana
		109	Andean mediterranean-tropical dwarf scrub of Adesmia subterranea - Adesmia echinus
		110	Coastal mediterranean dwarf scrub of Chuquiraga oppositifolia - Mulinum spinosum
		111	Andean mediterranean dwarf scrub of Chuquiraga oppositifolia - Nardophyllum lanatum
		112	Andean mediterranean dwarf scrub of Laretia acaulis - Berberis empetrifolia
		113	Andean mediterranean dwarf scrub of Chuquiraga oppositifolia - Discaria articulata
		114	Andean temperate dwarf scrub of Discaria chacaye / Berberis empetrifolia
		115	Andean temperate dwarf scrub of Adesmia longipes - Senecio bipontinii
		116	Andean antiboreal dwarf scrub of Bolax gummifera - Azorella selago
		117	Andean mediterranean-tropical alpine herbaceous vegetation of Chaetanthera sphaeroidalis
		118	Andean mediterranean alpine herbaceous vegetation of Nastanthus spathulatus - Menonvillea spathulata
		119	Andean mediterranean alpine herbaceous vegetation of Oxalis adenophylla - Pozoa coriacea
		120	Andean temperate alpine herbaceous vegetation of Nassauvia dentata - Senecio portalesianus
		121	Andean antiboreal alpine herbaceous vegetation of Nassauvia pygmaea - N. lagascae
	T6.5 Tropical alpine meadows and shrublands		
		97	Andean tropical dwarf scrub of Fabiana ramulosa - Diplostephium meyenii

Biome	Ecosystem Functional Group	map unit #	Vegetation belt (Luebert & Pliscoff 2017)
		98	Andean tropical dwarf scrub of Parastrephia lucida - Azorella compacta
		99	Andean tropical dwarf scrub of Parastrephia lucida / Festuca orthophylla
		100	Andean tropical dwarf scrub of Parastrephia lepidophylla - P. qudrangularis
		101	Andean tropical dwarf scrub of Azorella compacta - Pycnophyllum molle
		102	Andean tropical dwarf scrub of Fabiana denudata - Chuquiraga atacamensis
		103	Andean tropical dwarf scrub of Fabiana squamata / Festuca chrysophylla
		104	Andean tropical dwarf scrub of Fabiana bryoides - Parastrephia quadrangularis
		105	Andean tropical dwarf scrub of Mulinum crassifolium - Urbania pappigera
		106	Andean tropical dwarf scrub of Artemisia copa / Jarava frigida
		107	Andean tropical dwarf scrub of Adesmia frigida / Jarava frigida
	TF1.6 Boreal, temperate and montane peat bogs		
		93	Coastal temperate moorland of Donatia fascicularis - Oreobolus obtusangulus
		94	Coastal antiboreal moorland of Astelia pumila - Donatia fascicularis
		95	Coastal temperate-antiboreal moorland of Bolax caespitosus - Phyllachne uliginosa
		96	Inland temperate-antiboreal moorland of Sphagnum magellanicum / Schoenus antarcticus

Table S3.4. Cross-walk of the Maynmar national ecosystem classification (Murray et al. 2020) with global ecosystem Functional Groups.

Biome	Ecosystem Functional Group	map unit #	Myanmar ecosystem (Murray et al. 2020)
T1 Tropical & subtropical forests (T1)			
	T1.1 Tropical-Subtropical lowland rainforests		
		MMR-T1.1.1	Tanintharyi island rainforests
		MMR-T1.1.2	Tanintharyi Sundaic lowland evergreen rainforest
		MMR-T1.1.3	Tanintharyi limestone tropical evergreen forest
		MMR-T1.1.4	Tanintharyi upland evergreen rainforest
		MMR-T1.1.5	Karin evergreen tropical rainforest
		MMR-T1.1.6	Southern Rakhine evergreen rainforest
		MMR-T1.1.7	Western Shan Plateau subtropical evergreen rainforest
		MMR-T1.1.8	Kachin - Sagaing low elevation subtropical rainforest
		MMR-T1.1.9	Kachin – Sagaing mid elevation subtropical rainforest
		MMR-T1.1.10	Kachin Hills subtropical rainforest
	T1.2 Tropical-Subtropical dry forests and scrubs		
		MMR-T1.2.1	Tanintharyi semi-evergreen forest
		MMR-T1.2.2	Rocky Taninthayri karst
		MMR-T1.2.3	Mixed cane break
		MMR-T1.2.4	Bago semi-evergreen forest
		MMR-T1.2.5	Dry zone foothills spiny scrub
		MMR-T1.2.6	Rakhine Elephant Range bamboo brake
		MMR-T1.2.7	Elephant range semi-evergreen dry forest
		MMR-T1.2.8	Magway dry cycad forest
		MMR-T1.2.9	Magway semi-evergreen dry gully forest
		MMR-T1.2.10	East Myanmar dry valley forest
		MMR-T1.2.11	Eastern Shan semi-evergreen forest
		MMR-T1.2.12	Western Shan semi-evergreen forest
		MMR-T1.2.13	Indaing forest
		MMR-T1.2.14	Northern semi-evergreen forest
		MMR-T1.3.1	Tanintharyi cloud forest
T2 Temperate-boreal forests & woodlands			
		MMR-T2.1.1	Kachin mountain conifer forest
		MMR-T2.4.1	Shan Warm Temperate Rainforest
		MMR-T2.4.2	Chin Hills warm temperate rainforest
		MMR-T2.4.3	Chin-Naga Warm Temperate Rainforest
		MMR-T2.4.4	Kachin Warm Temperate Rainforest
		MMR-T2.4.5	Mountain bamboo brake
		MMR-T2.4.6	Kachin Montane Temperate Broadleaf Forest
T4 Savannas and grasslands			
		MMR-T4.2.1	Rakhine coastal savanna
		MMR-T4.2.2	Central Ayeyarwady Than-Dahat grassy forest
		MMR-T4.2.3	Central Ayerawady Palm Savanna
		MMR-T4.2.4	Shwe Settaw Sha-Bamboo thicket
		MMR-T4.2.5	Magway Than-Dahat dry grassy forest
		MMR-T4.2.6	Sha Thorny Scrub
		MMR-T4.2.7	Shan foothills Than-Dahat grassy forest
		MMR-T4.2.8	Shan hills pine savanna
		MMR-T4.2.9	Chin hills pine savanna
		MMR-T4.2.10	Sagaing hills pine savanna
		MMR-T4.2.11	Kachin pine savanna

Biome	Ecosystem Functional Group	map unit #	Myanmar ecosystem (Murray et al. 2020)
		MMR-T4.5.1	Shan limestone grassland
T6 Polar/alpine (cryogenic)			
		MMR-T6.1.1	Kachin snowfields
		MMR-T6.2.1	Alpine cliffs and screes
		MMR-T6.4.1	High mountain scrub
		MMR-T6.4.2	Alpine herbfield
S1 Dry subterranean			
		MMR-S1.1.1	Aerobic karst caves
SF1 Subterranean streams			
		MMR-SF1.1.1	Subterranean karst streams
TF1 Palustrine wetlands			
		MMR-TF1.1.1	Ayeyarwady kanazo swamp forest
		MMR-TF1.1.2	Central dry evergreen riparian forest
		MMR-TF1.1.3	Mixed delta scrub
		MMR-TF1.4.1	Ayeyarwady floodplain wetlands
		MMR-TF1.4.2	Central Ayeyarwady floodplain grasslands
F2 Lakes			
		MMR-F2.4.1	Glacial Lakes
MT1 Shoreline systems			
		MMR-MT1.2.1	Coastal mudflats
		MMR-MT1.3.1	Sandy shoreline
MT2 Supralittoral coastal systems			
		MMR-MT2.1.1	Tanintharyi coastal dune forest
		MMR-MT2.1.2	Rakhine coastal dune forest
MFT1 Brackish tidal systems			
		MMR-MFT1.2.1	Tanintharyi mangrove forest
		MMR-MFT1.2.2	Ayeyarwady delta mangrove forest
		MMR-MFT1.2.3	Dwarf mangrove (shrubland) on shingle
		MMR-MFT1.2.4	Rakhine mangrove forest on mud
		MMR-MFT1.3.1	Grassy saltmarsh
