# Supplementary material for: A function-based typology for Earth’s ecosystems
Source: Nature. 2022 Oct 12;610(7932):513–8. doi: 10.1038/s41586-022-05318-4 (PMC9581774; doi:10.1038/s41586-022-05318-4)
Supplement: Supplementary file 7 — The IUCN Global Ecosystem Typology v2.1: Descriptive profiles for functional biomes and ecosystem functional groups. Descriptions and explanatory notes for 25 functional biomes and 110 ecosystem functional groups in v2.1 of the typology. Descriptions include text, images, diagrammatic assembly models, indicative global maps and key references. Explanatory notes include keys to the diagrammatic models and map assembly methods and data sources. [file 41586_2022_5318_MOESM7_ESM.pdf]

Appendix S4. The IUCN Global Ecosystem Typology v2.1: Descriptive profiles for Functional Biomes and Ecosystem Functional Groups

'A function-based typology for Earth's ecosystems'

David A. Keith, Jose R. Ferrer-Paris, Emily Nicholson, Melanie J. Bishop, Beth A. Polidoro, Eva Ramirez-Llodra, Mark G. Tozer, Jeanne L. Nel, Ralph Mac Nally, Edward J. Gregr, Kate E. Watermeyer, Franz Essl, Don Faber-Langendoen, Janet Franklin, Caroline E. R. Lehmann, Andres Etter, Dirk Roux, Jonanthan S. Stark, Jessica A. Rowland, Neil A. Brummitt, Ulla C. Fernandez-Arcaya, Iain M. Suthers, Susan K. Wiser, Ian Donohue, Leland J. Jackson, R. Toby Pennington, Thomas M. Iliffe, Vasilis Gerovasileiou, Paul Giller, Belinda J. Robson, Nathalie Pettorelli, Angela Andrade, Arild Lindgaard, Teemu Tahvanainen, Aleks Terauds, Michael A Chadwick, Nicholas J. Murray, Justin Moat, Patricio Pliscoff, Irene Zager, Richard T. Kingsford

Nature 2022 https://doi.org/10.1038/s41586-022-05318-4

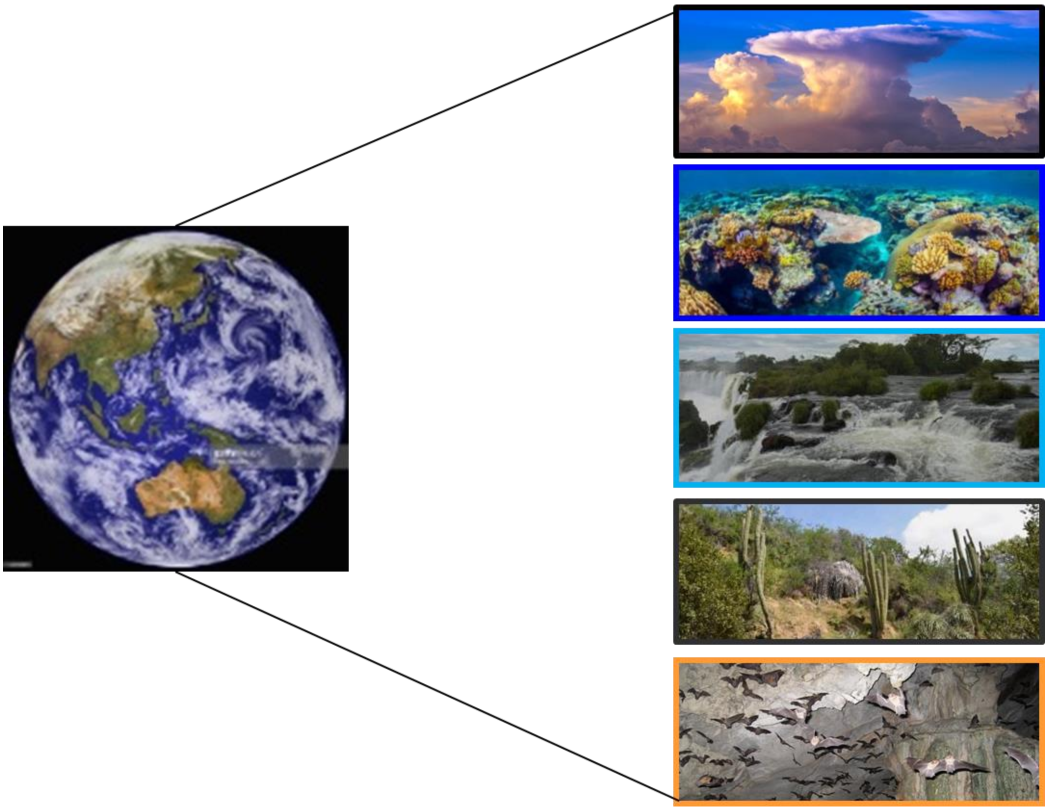

Contributors to descriptive profiles

Professor David A. Keith, *Lead author and editor*	Centre for Ecosystem Science, University of NSW, Sydney, Australia<br>NSW Department of Planning, Industry and Environment, Sydney, Australia<br>IUCN Commission on Ecosystem Management, Gland, Switzerland
Dr Jose R. Ferrer-Paris, *Map development*	Centre for Ecosystem Science, University of NSW, Sydney, Australia<br>IUCN Commission on Ecosystem Management, Gland, Switzerland
Associate Professor Emily Nicholson, *Peer-review editor*	Centre for Integrative Ecology, Deakin University, Burwood, Victoria, Australia<br>IUCN Commission on Ecosystem Management, Gland, Switzerland
Professor Richard T. Kingsford, *Governance, author and editor*	Centre for Ecosystem Science, University of NSW, Sydney, Australia
Professor Alicia T.R. Acosta	Department of Environmental Biology, Università Roma, Italy
Dr Andrew H. Altieri	Department of Environmental Engineering Sciences, University of Florida, Gainesville, USA
Dr Angela Andrade	Conservation International Colombia, Bogota, Colombia<br>IUCN Commission on Ecosystem Management, Gland, Switzerland
Professor Alexandre M.B. Anesio	Department of Environmental Science, Aarhus University, Denmark
Professor Angela H. Arthington	Australian Rivers Institute, Griffith University, Australia
Dr Jorge Assis	Centro de Ciências do Mar (CCMAR), Universidade do Algarve, Faro, Portugal
Professor Stefan Bertilsson	Department of Aquatic Sciences and Assessment, Swedish University of Agricultural Sciences, Uppsala, Sweden
Professor Malcom Beveridge	University of Stirling, Stirling, Scotland
Dr P. Bhanumati	National Statistical Office, Ministry of Statistics and Programme Implementation, New Delhi, India
Professor Thomas S. Bianchi	Department of Geological Sciences, University of Florida, Miami, USA
Associate Professor Melanie J. Bishop	Department of Biological Sciences, Macquarie University, Sydney, Australia
Dr Lucie M. Bland	Lucie Bland Book Coach 7 Editor, Melbourne, Australia
Dr Patrick Bogaart	United Nations Statistics Division, The Hague, Netherlands
Dr Stef F. Bokhorst	Department of Ecological Science, Vrije Universiteit, Amsterdam, The Netherlands
Dr David Brankovits	Marine Biology Department, Texas A&M University, Galveston, USA
Dr Neil A. Brummitt	Department of Life Sciences, Natural History Museum, London, England
Dr Paul E. Carnell	Centre for Integrative Ecology, Deakin University, Burwood, Australia
Dr Jane Catford	Kings College London, England
Dr Michael A Chadwick	Department of Geography, King's College London, England
Dr Alan Channing	School of Earth & Ocean Sciences, Cardiff University, Wales
Associate Professor Jeremy T Claisse	Biological Sciences, California State Polytechnic University, Pomona, USA
Professor Richard T. Corlett	Xishuangbanna Tropical Botanical Garden, China
Professor Tasman P. Crowe	School of Biology and Environmental Science, University College Dublin, Ireland

Dr Nick A. Cutler	School of Geography, Politics & Sociology, Newcastle University, England
Dr Katherine A. Dafforn	Department of Earth and Environmental Sciences, Macquarie University,Sydney, Australia
Associate Professor Ian Donohue	School of Natural Sciences, Department of Zoology, Trinity College Dublin, Dublin, Ireland
Professor David Eldridge	Centre for Ecosystem Science, University of NSW, Sydney, Australia
Dr Julie C. Ellis	School of Veterinary Medicine, University of Pennsylvania, Philadelphia, USA
Associate Professor Franz Essl	Department of Botany and Biodiversity Research, Unversity of Vienna, Vienna, Austria<br>Centre for Invasion Biology, Department of Botany and Zoology, Stellenbosch University, Stellenbosch, 7602, South Africa
Professor Andrés Etter	Departamento de Ecología y Territorio, Pontificia Universidad Javeriana, Bogotá, Colombia
Dr Don Faber-Langendoen	Conservation Science Division, NatureServe, Arlington, USA<br>SUNY College of Environmental Science and Forestry, Syracuse, USA
Dr Ulla Fernandez-Arcaya	Instituto Español de Oceanografía, Centro Oceanográfico de Baleares, Palma, Spain
Dr Louise B. Firth	School of Biological and Marine Sciences, University of Plymouth, England
Dr Eliza Fragkopoulou	Centro de Ciências do Mar (CCMAR), Universidade do Algarve, Faro, Portugal
Professor Janet Franklin	Department of Botany, University of California, Riverside, United States of America
Dr Lila García	Provita, Caracas, Venezuela
Dr Vasilis Gerovasileiou	Hellenic Centre for Marine Research, Anavyssos, Greece
Professor David Gibson	Department of Plant Biology, Southern Illinois University, Carbondale, USA
Professor Paul S. Giller	University College Cork, Ireland
Dr Brett C. Gonzalez	Smithsonian National Museum of Natural History, Washington DC, USA
Simon Gorta	Centre fr Ecosystem Science, University of NSW, Sydney, Australia
Dr Edward J. Gregr	Institute for Resources, Environment, and Sustainability, University of British Columbia, Vancouver, British Columbia, Canada<br>SciTech Environmental Consulting, Vancouver, British Columbia, Canada
Professor Richard Harper	School of Veterinary and Life Sciences, Murdoch University, Perth, Australia
Dr Justin C. Havird	Department of Biology, Colorado State University, Fort Collins, USA
Dr Sylvia E. Hay	Centre for Ecosystem Science, University of NSW, Sydney, Australia
Professor J. Tim Hollibaugh	Department of Marine Sciences, University of Georgia, Athens, USA
Professor Grant C. Hose	Department of Biological Sciences, Macquarie University, Australia
Professor Thomas M. Iliffe	Marine Biology Department, Texas A&M University, Galveston, USA
Professor Kenneth Irvine	IHE Delft Institute for Water Education, Delft, Netherlands
Professor Leland J. Jackson	Department of Biological Sciences, University of Calgary, Calgary, Canada.

Associate Professor Mary Kelly-Quinn	School of Biology and Environmental Science, University College Dublin, Ireland
Dr Tytti Kontula	Finnish Environment Institute, Helsinki, Finland
Professor Christian H. Körner	Department of Environmental Sciences, University of Basel, Basel, Switzerland
Megan Lamson	University of Hawaii, Hilo, USA
Dr Caroline E. R. Lehmann	Royal Botanic Garden Edinburgh, Edinburgh Scotland<br>School of GeoSciences, University of Edinburgh, Edinburgh, Scotland
Dr Christy Linardich	Arizona State University, Glendale, USA
Dr Arild Lindgaard	Norwegian Biodiversity Information Centre, Trondheim, Norway
Professor Javier Loidi	Department of Plant Biology and Ecology, University of the Basque Country, Spain
Professor Ralph Mac Nally	Institute for Applied Ecology, University of Canberra, Canberra, Australia<br>School of BioSciences, The University of Melbourne, Melbourne, Australia
Dr Alejandro Martínez García	Institute for Ecosystem Studies, Italian National Research Council, Italy
Dr Matt McGlone	Manaaki Whenua -- Landcare Research, Lincoln, New Zealand
Dr Sarah E. McSweeney	Department of Geography, The University of Melbourne, Melbourne, Australia
Dr Justin Moat	Royal Botanic Gardens Kew, England
Dr Nicholas J. Murray	College of Science & Engineering, James Cook University, Townsville, Australia
Dr Jeanne L. Nel	Sustainability Research Unit, Nelson Mandela University, Port Elizabeth, South Africa<br>Wageningen Environmental Research, Wageningen University, Wageningen, The Netherlands
Professor Wendy A. Nelson	Department of Biological Sciences, University of Auckland, Auckland, New Zealand
Professor Robert J. Orth	Biological Sciences Department, Virginia Institute of Marine Science, Gloucester Point, USA
Professor R. Toby Pennington	Geography, University of Exeter, Exeter, England<br>Royal Botanic Garden Edinburgh, Edinburgh, Scotland
Dr Nathalie Pettorelli	Zoological Society of London, London, England
Dr Patricio Pliscoff	Department of Ecology, Pontifica Universidad Católitica de Chile, Santiago, Chile
Associate Professor Beth A. Polidoro	School of Mathematical and Natural Sciences, Arizona State University, Glendale, USA
Professor Imants G. Priede	Institute of Biological and Environmental Sciences, University of Aberdeen, Scotland
Professor Mark C. Rains	School of Geosciences, University of South Florida, USA
Dr Eva Ramirez-Llodra	Norwegian Institute for Water Research, Gaustadalléen, Oslo, Norway<br>REV Ocean, Lysaker, Norway
Dr Catherine A. Reidy Liermann	Huxley College of the Environment, Western Washington University, USA
Associate Professor Belinda J Robson	School of Environmental Science, Murdoch University, Perth, Australia
Associate Professor Sergio Rossi	Department of Biological and Environmental Sciences and Technologies, Università del Salento, Lecce, Italy
Dr Dirk J. Roux	Scientific Services, South African National Parks, George, South Africa<br>Sustainability Research Unit, Nelson Mandela University, George, South Africa

Dr Jessica A. Rowland	Centre for Integrative Ecology, Deakin University, Burwood, Victoria, Australia
Professor Jeremy Russell-Smith	Bushfire and Natural Hazards CRC, Charles Darwin University, Darwin Australia
Dr Ryan R. Rykaczewski	Ecosystem Sciences Division, NOAA Pacific Islands Fisheries Science Center, Honolulu, USA
Dr Troy S. Sakihara	Department of Land and Natural Resources, Hawaii, USA
Dr Scott R. Santos	Department of Biological Sciences, Auburn University, Auburn, Alabama, USA
Dr Nadine Schubert	Centre of Marine Sciences (CCMAR), Universidade do Algarve, Faro, Portugal
Dena M. Sedar	Hawaii State Parks, Honolulu, USA
Barbara Seidel	The Nature Conservancy, Honolulu, USA
Professor Lynne Shannon	Department of Biological Sciences, University of Cape Town, South Africa
Dr Monica Sharma	Consultant to the United Nations, New Delhi, India
Professor Charles R.C. Sheppard	Department of Life Sciences, University of Warwick, Coventry, England
Professor Martin J. Siegert	Grantham Institute for Climate Change & Environment, Imperial College London, London, England
Dr Jonathan S. Stark	Australian Antarctic Division, Hobart, Australia
Professor Iain M. Suthers	Centre for Marine Science & Innovation, University of New South Wales, Sydney, Australia
Professor Tracey T. Sutton	Department of Marine and Environmental Sciences, Nova Southeastern University, Florida, USA
Professor Stephen E. Swearer	School of BioSciences, The University of Melbourne, Australia
Dr Teemu Tahvaneinan	Department of Environmental and Biological Sciences, University of Eastern Finland, Joensuu, Finland
Dr Aleks Terauds	Australian Antarctic Division, Hobart, Australia
Mark G. Tozer	NSW Department of Planning, Industry and Environment, Sydney, Australia
Dr Kate E. Watermeyer	Centre for Integrative Ecology, Deakin University, Burwood, Victoria, Australia
Dr Richard Williams	CSIRO, Melbourne, Australia
Dr Susan K. Wiser	Manaaki Whenua -- Landcare Research, Lincoln, New Zealand
Dr Ryan Woodland	Center for Environmental Science, University of Maryland, Solomons, USA
Professor Kenneth R. Young	Department of Geography and the Environment, University of Texas, Austin, USA
Dr Irene Zager	Provita, Caracas, Venezuela<br>IUCN Commission on Ecosystem Management, Gland, Switzerland

Contents

Contributors to descriptive profiles 2

Descriptive profiles for Functional Biomes and Ecosystem Functional Groups 11

Nomenclature 11

Text descriptions 11

Exemplary photographs 11

Key ecological drivers 12

Diagrammatic assembly models 12

Indicative distribution maps 13

Use of references 40

Updates 40

References cited 42

List of Ecosystem Functional Groups by realms and biomes 48

T1. Tropical-subtropical forests biome 52

T1.1 Tropical/Subtropical lowland rainforests 53

T1.2 Tropical/Subtropical dry forests and thickets 54

T1.3 Tropical/Subtropical montane rainforests 55

T1.4 Tropical heath forests 56

T2. Temperate-boreal forests and woodlands biome 57

T2.1 Boreal and temperate high montane forests and woodlands 58

T2.2 Deciduous temperate forests 59

T2.3 Oceanic cool temperate rainforests 60

T2.4 Warm temperate laurophyll forests 61

T2.5 Temperate pyric humid forests 62

T2.6 Temperate pyric sclerophyll forests and woodlands 63

T3. Shrublands and shrubby woodlands biome 64

T3.1 Seasonally dry tropical shrublands 65

T3.2 Seasonally dry temperate heath and shrublands 66

T3.3 Cool temperate heathlands 67

T3.4 Young rocky pavements, lava flows and screes 68

T4. Savannas and grasslands biome 69

T4.1 Trophic savannas 70

T4.2 Pyric tussock savannas 71

T4.3 Hummock savannas 72

T4.4 Temperate woodlands 73

T4.5 Temperate tussock grasslands 74
T5. Deserts and semi-deserts biome 75
T5.1 Semi-desert steppe 76
T5.2 Succulent or Thorny deserts and semi-deserts 77
T5.3 Sclerophyll hot deserts and semi-deserts 78
T5.4 Cool deserts and semi-deserts 79
T5.5 Hyper-arid deserts 80
T6. Polar/alpine (cryogenic) biome 81
T6.1 Ice sheets, glaciers and perennial snowfields 82
T6.2 Polar/alpine cliffs, screes, outcrops and lava flows 83
T6.3 Polar tundra and deserts 84
T6.4 Temperate alpine grasslands and shrublands 85
T6.5 Tropical alpine grasslands and herbfields 86
T7. Intensive land-use biome 87
T7.1 Short-rotation croplands 88
T7.2 Intensive livestock pastures 89
T7.3 Plantations 90
T7.4 Cities, villages and infrastructure 91
T7.5 Derived semi-natural pastures and old fields 92
S1. Subterranean lithic biome 93
S1.1 Aerobic caves 94
S1.2 Endolithic systems 95
S2. Anthropogenic subterranean voids biome 96
S2.1 Anthropogenic subterranean voids 97
SF1. Subterranean freshwaters biome 98
SF1.1 Underground streams and pools 99
SF1.2 Groundwater ecosystems 100
SF2. Anthropogenic subterranean freshwaters biome 101
SF2.1 Water pipes and subterranean canals 102
SF2.2 Flooded mines and other voids 103
SM1. Subterranean tidal biome 104
SM1.1 Anchialine caves 105
SM1.2 Anchialine pools 106
SM1.3 Sea caves 107
TF1. Palustrine wetlands biome 108

TF1.1 Tropical flooded forests and peat forests 109
TF1.2 Subtropical/temperate forested wetlands 110
TF1.3 Permanent marshes 111
TF1.4 Seasonal floodplain marshes 112
TF1.5 Episodic arid floodplains 113
TF1.6 Boreal, temperate and montane peat bogs 114
TF1.7 Boreal and temperate fens 115
F1. Rivers and streams biome 116
F1.1 Permanent upland streams 117
F1.2 Permanent lowland rivers 118
F1.3 Freeze-thaw rivers and streams 119
F1.4 Seasonal upland streams 120
F1.5 Seasonal lowland rivers 121
F1.6 Episodic arid rivers 122
F1.7 Large lowland rivers 123
F2. Lakes biome 124
F2.1 Large permanent freshwater lakes 125
F2.2 Small permanent freshwater lakes 126
F2.3 Seasonal freshwater lakes 127
F2.4 Freeze-thaw freshwater lakes 128
F2.5 Ephemeral freshwater lakes 129
F2.6 Permanent salt and soda lakes 130
F2.7 Ephemeral salt lakes 131
F2.8 Artesian springs and oases 132
F2.9 Geothermal pools and wetlands 133
F2.10 Subglacial lakes 134
F3. Artificial wetlands biome 135
F3.1 Large reservoirs 136
F3.2 Constructed lacustrine wetlands 137
F3.3 Rice paddies 138
F3.4 Freshwater aquafarms 139
F3.5 Canals, ditches and drains 140
FM1. Semi-confined transitional waters biome 141
FM1.1 Deepwater coastal inlets 142
FM1.2 Permanently open riverine estuaries and bays 143

FM1.3 Intermittently closed and open lakes and lagoons 144
M1. Marine shelf biome 145
M1.1 Seagrass meadows 146
M1.2 Kelp forests 147
M1.3 Photic coral reefs 148
M1.4 Shellfish beds and reefs 149
M1.5 Photo-limited marine animal forests 150
M1.6 Subtidal rocky reefs 151
M1.7 Subtidal sand beds 152
M1.8 Subtidal mud plains 153
M1.9 Upwelling zones 154
M1.10 Rhodolith/Maërl beds 155
M2. Pelagic ocean waters biome 156
M2.1 Epipelagic ocean waters 157
M2.2 Mesopelagic ocean water 158
M2.3 Bathypelagic ocean waters 159
M2.4 Abyssopelagic ocean waters 160
M2.5 Sea ice 161
M3. Deep sea floors biome 162
M3.1 Continental and island slopes 163
M3.2 Submarine canyons 164
M3.3 Abyssal plains 165
M3.4 Seamounts, ridges and plateaus 166
M3.5 Deepwater biogenic beds 167
M3.6 Hadal trenches and troughs 168
M3.7 Chemosynthetic-based-ecosystems (CBE) 169
M4. Anthropogenic marine biome 170
M4.1 Submerged artificial structures 171
M4.2 Marine aquafarms 172
MT1. Shorelines biome 173
MT1.1 Rocky Shorelines 174
MT1.2 Muddy Shorelines 175
MT1.3 Sandy Shorelines 176
MT1.4 Boulder and cobble shores 177
MT2. Supralittoral coastal biome 178

MT2.1 Coastal shrublands and grasslands ... 179
MT2.2 Large seabird and pinniped colonies ... 180
MT3. Anthropogenic shorelines biome ... 181
MT3.1 Artificial shorelines ... 182
MFT1. Brackish tidal biome ... 183
MFT1.1 Coastal river deltas ... 184
MFT1.2 Intertidal forests and shrublands ... 185
MFT1.3 Coastal saltmarshes and reedbeds ... 186

Descriptive profiles for Functional Biomes and Ecosystem Functional Groups

The supplementary material in this appendix includes descriptive profiles for functional biomes (Level 2) and Ecosystem Functional Groups (EFGs) of the IUCN Global Ecosystem Typology v2.1 (superseding Keith et al. 2020). Updates of the typology are published at https://global-ecosystems.org/. The descriptive profiles provide brief summaries of key ecological traits and processes, particularly features that distinguish different functional groups from one another to inform diagnosis and to enable any ecosystem type to be assigned to a group.

Inevitably, there are inherent uncertainties in assigning ecosystem types to unique EFGs because ecological classifications, in general, simplify complex multidimensional variation in nature by segmenting and categorising continuous gradients in multiple features (see Appendix S3; Regan et al. 2002). Thus, any given ecosystem type may possess a suite of features that are typical of different functional groups, and a single feature can rarely be definitive for ecosystem identification (e.g. Erb et al. 2013). For this reason we avoid prescriptive approaches to description of the units that seek to identify strictly exclusive or diagnostic ecosystem characteristics, and instead use appropriate qualifiers and caveats in descriptions where important exceptions apply to generalisations about ecosystem properties and postulated drivers. Users should assess and weigh evidence on all features to identify the most likely functional group and report the nature of uncertainties in group membership.

Nomenclature

Terminology follows the Glossary (see Supplementary Information). Names of functional biomes and EFGs are vernacular — we adopt names and descriptors frequently applied in the literature that reflect key ecosystem properties. A vernacular (rather than systematic) approach to nomenclature is intended to exploit terms (e.g. rainforest, lake, or reef) that are familiar to a wide range of users, recognising regional variations and conventions in terminology, and hence more likely to facilitate wide uptake than an approach seeking to impose rigid naming conventions. Although a vernacular approach seems appropriate at this stage of development of the global typology, further work on the development a more systematic approach, or at least guidance on how salient features of classifications units can be represented in the names of units. Senterre et al (2021), for example, propose several principles for the development of ecosystem nomenclature.

Text descriptions

The text describes key ecosystem properties that characterise each EFG and help distinguish it from other groups. The descriptions include ecosystem-level properties and ecological processes (e.g. productivity, energy sources, trophic structure, physical structure, bottom-up and top-down organisational processes) as well as species-level traits that are represented among the component biota of ecosystem types within the group (e.g. life forms, life-history traits, specialised organs, and characteristic behaviours and mobility). Where possible, variability in traits is noted. The Glossary (Supplementary Information) defines selected technical terms used in the profiles.

Exemplary photographs

Each profile is illustrated with a photograph that shows some of the ecological features mentioned in the text. Although representative examples were chosen for illustration, they may

not represent the range of variability in features expressed within each EFG, some of which have extensive global distributions. In future work, we will buid a library of reference images to illustrate variability more fully (https://global-ecosystems.org/) .

Key ecological drivers

The text identifies key ecological drivers that are positied to shape the ecological character of ecosystem types within a functional group. The processes identified for all EFGs were deduced from a consistent conceptual framework defined by a diagnostic model of ecosystem assembly (Fig. 1), the underpinnings of which are drawn from ecological theory, as described in Appendix S2. The inferences made about major drivers for each EFG represent hypotheses based on the consensus of specialist contributors and peer reviewers of the profiles (Appendix S5). Contributors drew from a substantial body of evidence, including their knowledge of scientific literature and direct research experience in the field. The most important literature is cited in respective descriptions, subject to limitations on space (see 'Use of references' below). We acknowledge that experts may put forward diverging but legitimate interpretations of available evidence on the nature of ecological drivers that influence salient ecosystem properties. The consensus interpretations in the descriptive profiles therefore may not represent all possible interpretations of available evidence.

Abiotic drivers and processes include ambient environmental features and disturbance regimes that directly or indirectly influence resource availability. Biotic drivers include a range of interactions and dependencies that arise from the biotic properties of the ecosystem. Hence there are inherent feedbacks between biotic drivers and ecosystem properties. Human activities are explicitly addressed as ecological drivers in anthropogenic EFGs. The descriptions of non-anthropogenic EFGs, however, focus on reference states with negligible human influence, even though humans affect most ecosystems on earth. These effects vary greatly in type, intensity and spatially in a manner that reflects social, cultural and economic norms and opportunities, technology and access, as well as ecosystem characteristics. Specific influences of anthropogenic processes vary with ecosystem state. A focus on reference states in this treatement will therefore enable the wide range of human influences to be addressed with appropriate assessment tools such as the IUCN Red List of Ecosystems protocol (Keith et al. 2013).

Diagrammatic assembly models

To illustrate characteristic ecosystem properties and assembly filters (i.e. drivers that shape ecosystem properties). an ecosystem assembly model was developed for each EFG, by adapting the generic model described in Fig. 1 (main text) and Appendix S2. As noted above, the inferences made about major processes and traits for each EFG represent hypothesese based on the consensus of specialist contributors and peer reviewers, who drew from a substantial body of evidence, including their knowledge of scientific literature and direct research experience in the field. Nonetheless, we acknowledge uncertainties and that there may be legitimate alternative diagrammatic representations of evidence on these relationships.

Only the major features are shown in the diagrammatic models and anthropogenic processes are only shown for anthropogenic EFGs. In the diagrammatic representations of the models, ecosystem properties are listed in green circles at the centre, while drivers are identified in peripheral boxes using the following colours:

- pale blue: resources

- dark blue: ambient environmental factors that influence resource availability or uptake
- red: environmental disturbance regimes
- orange: biotic interactions
- black: human activities

Connecting arrows show hypothesised influences of, and interactions among drivers as well as feedbacks (bidirectional arrows). Only major connections are shown and feedbacks are generally not shown except for those involving biotic components.

Indicative distribution maps

We separated the task of mapping spatial distributions from constructing the typology and defining its units. This liberates the definition of units from constraints imposed by current availability of spatial data and allows for progressive improvement of maps representing spatial expression of conceptually stable ecosystem types. Maps are, however, essential to many applications (Appendix S6) including ecosystem risk assessment and management (Design Principle 5, Table S.1). Classification units at all levels of the typology have spatial distributions and are therefore mappable, aided by recent advances in global spatial data and cloud computing (Murray et al. 2018). Mapping at any level of the typology requires spatially explicit ground observations, interpretive expertise, spatial predictors (including remote sensing data and environmental variables) and appropriate methods for spatial interpolation (Guisan & Zimmermann, 2000).

Separate distribution maps were developed for each EFG, largely independently of one another. This multi-layer format to the spatial data enabled us to incorporate more spatial information on EFG distributions than is possible in a single composite map. It also enabled us to accommodate different mapping approaches appropriate to particular ecosystem types, different levels of data quality and uncertainty, different degrees of spatial dynamism over relatively short time scales, and spatial juxtapositions with other EFGs. The multi-layered format allows occurrences of two or more EFGs to be represented within the same spatial unit (i.e. grid cells). Interactive versions of the maps are available at https://global-ecosystems.org/.

The maps show areas of the world containing major (in red) or minor occurrences (in yellow) of each EFG. Major occurrences indicate areas where an EFG occupies a large portion (generally >20%) of a landscape or seascape. Minor occurrences are areas where an EFG is scattered in patches within matrices of other EFGs or where they occur in large patches but only within a segment of a larger region. Distributions that are uncertain were mapped as minor occurrences across large geographic envelopes. Small but important occurrences are identified with black ellipses. In landscapes or seascapes occupied by mosaics of ecosystems, EFGs comprising the matrix of the mosaic are mapped as major occurrences, and those distributed in patches are mapped as minor occurrences.

The maps were designed to be indicative of global distribution patterns and are not intended to represent fine-scale patterns. The spatial grain of map rasters varies from 10 minutes to 1 degree of latitude and longitude, depending on the resolution of available base layers (Table S4.1). For most EFGs, the spatial resolution is 30 arc seconds, approximately 1 km^2 at the equator. Given bounds of resolution and accuracy of source data, the maps should be used to query which EFGs are likely to occur within areas, rather than which occur at particular point locations.

To compile distribution maps, we first searched for existing spatial data on map units that aligned with the concept of individual EFGs by comparing descriptions in metadata or associated publications to the EFG descriptive profiles. We found matching spatial data sets that directly matched the concepts of 38 EFGs, comprising either polygons or rasters (e.g. MT1.2, T7.4, M1.3; Table S4.1) or point records (e.g. F3.1). For eight of those EFGs, we supplemented direct maps with biogeographic regions likely to contain minor occurrences (e.g. TF1.1). For a further 21 EFGs, we found maps that aligned with key features of EFGs, but applied them over a broader range of environments or locations (ie. a 'semi-driect' match). In those cases, we used environmental spatial data or biogeographic regions to clip the broader mapped extent to achieve closer alignment with the EFG concept (e.g. F1.1, T1.1). The remaining EFGs had no suitable direct mapping, we assembled maps from simple combinations of remote sensing and/or environmental proxies, clipped by biogeographic regions where necessary to obtain an approximate match to the concept. Base data for all maps were published in peer-reviewed scientific literature and/or available in global repositories administered by major agencies such as NASA or USGS. Source maps for 81 of the EFGs were based on known records, or had undergone a quantitative accuracy assessment or similar thematic evaluation. One EFG (S1.2) was not mapped due to its distribution throughout the Earth's crust.

Ecoregions (Spalding et al., 2007; Abell et al., 2008; Dinerstein et al., 2017) are one of numerous spatial data sets used in the construction of some of the maps. We use them as templates to either to constrain the extent mapped from remote sensing and environmental proxies or to provide indicative distributions of minor occurrences that occur outside the core distributions of some EFGs. We emphasise that the conceptual differences between Ecosystem Functional Groups (founded on ecological processes and ecosystem functions, irrespective of biotic composition; see definition in Table S3.1 and associated text) and ecoregions (founded on biogeographic patterns and processes and as proxies for species distrbutions; reviewed in Appendix S1). Similar distinctions apply to functional biomes (Table S3.1 and associated text) and biogeographic biomes as mapped and described in respective ecoregion classifications (provinces of Spalding et al., 2007; major habitat types of Abell et al., 2008; biomes of Olson et al., 2001 and Dinerstein et al., 2017), reflecting functional and biogeographic interpretations of the term "biome" (Mucina, 2019). Hence when EFGs are aggregated into functional biomes (Level 2 of the Global Ecosystem Typology), spatial patterns may differ from those of ecoregions aggregated into biomes (e.g. Olson et al., 2001).

Table S4.1. Methods and source data for indicative maps of each Ecosystem Functional Group (EFG). Concept alignment: Direct – Source map is consistent with EFG concept without adjustment; Semi-direct – Source map is consistent with EFG concept after clipping or supplementation; Indirect – Proxy variables used to construct map approximating EFG concept.

Code	Description	Concept alignment	Base data evaluation	Spatial resolution
T1.1	Lowland rainforests were initially identified using consensus land-cover maps (Tuanmu et al., 2014) and then cropped to selected terrestrial ecoregions (Dinerstein et al., 2017) at 30 arc seconds spatial resolution. Ecoregions were selected if: i) their descriptions mentioned features consistent with those identified in the profile of the Ecosystem Functional Group; and ii) if their location was consistent with the ecological drivers described in the profile. Ecoregions were designated as containing major occurrences if ecoregion descriptions mentioned extensive areas of lowland rainforests or as minor occurences if lowland rainforests were described as patches within a matrix dominated by other ecosystems.	Indirect	Quantitative accuracy assessment	30 arc-second (ca. 1km2) or better
T1.2	Tropical dry rainforests were initially identified using consensus land-cover maps (Tuanmu et al., 2014) and then cropped to selected terrestrial ecoregions (Dinerstein et al., 2017) at 30 arc seconds spatial resolution. Ecoregions were selected if: i) their descriptions mentioned features consistent with those identified in the profile of the Ecosystem Functional Group; and ii) if their location was consistent with the ecological drivers described in the profile. Ecoregions were designated as containing major occurrences if ecoregion descriptions mentioned extensive areas of dry forests, or as minor occurences if dry forests were described as patches within a matrix dominated by other ecosystems.	Indirect	Quantitative accuracy assessment	30 arc-second (ca. 1km2) or better
T1.3	The distribution of tropical montane rainforest was approximated from a model of environmental suitability based on climatic variables and cloud cover (Wilson and Jetz, 2016, Karger et al. 2021). Occurrences were aggregated to half degree spatial resolution and cells reclassified as major occurrences (>25% of cell area) and minor occurrences (< 25% of cell area).	Direct	Quantitative accuracy assessment	30 arc-second (ca. 1km2) or better
T1.4	Terrestrial ecoregions containing occurrences of heath forests were identified by consulting available ecoregion descriptions (Dinerstein et al., 2017), global and regional reviews, national and regional ecosystem maps, locations of relevant examples, and author expertise. Consequently, they are coarse-scale indicative representations of distribution, except where they occupy small ecoregions. Ecoregions were mapped at 30 arc second spatial resolution. Ecoregions were designated as containing major occurrences if ecoregion descriptions mentioned extensive areas of heath forests, or as minor occurences if heath forests were described as patches within a matrix dominated by other ecosystems.	Indirect	Quantitative thematic assessment	larger than 1km2/30 arc-second

Code	Description	Concept alignment	Base data evaluation	Spatial resolution
T2.1	Boreal and temperate montane forests were initially identified using consensus land-cover maps (Tuanmu et al., 2014; generalised landcover class 1 Evergreen/deciduous needleleaf trees) and then cropped to selected terrestrial ecoregions (Dinerstein et al., 2017) at 30 arc seconds spatial resolution. Ecoregions were selected if: i) their descriptions mentioned features consistent with those identified in the profile of the Ecosystem Functional Group; and ii) if their location was consistent with the ecological drivers described in the profile. Ecoregions were designated as containing major occurrences if ecoregion descriptions mentioned extensive areas of boreal and temperate montane forests, or as minor occurences if these forests were described as patches within a matrix dominated by other ecosystems.	Semi-direct	Quantitative accuracy assessment	30 arc-second (ca. 1km2) or better
T2.2	Temperate deciduous forests were initially identified using consensus land-cover maps (Tuanmu et al., 2014; generalised landcover class 3 Deciduous broadleaf trees) and then cropped to selected terrestrial ecoregions (Dinerstein et al., 2017) at 30 arc seconds spatial resolution. Ecoregions were selected if: i) their descriptions mentioned features consistent with those identified in the profile of the Ecosystem Functional Group; and ii) if their location was consistent with the ecological drivers described in the profile. Ecoregions were designated as containing major occurrences if ecoregion descriptions mentioned extensive areas of temperate deciduous forests, or as minor occurences if these forests were described as patches within a matrix dominated by other ecosystems.	Semi-direct	Quantitative accuracy assessment	30 arc-second (ca. 1km2) or better
T2.3	Cool temperate and boreal rainforest regions identified by DellaSala et al. (2011) were matched with ecoregion descriptions (Dinerstein et al., 2017) and proofed by author's expertise. Ecosregions were designated as major occurrences where rainforests dominated the landscape matrix, and minor occurrences where rainforests were present as patches within a matrix of other ecosystems. Ecoregions were mapped at 30 arc second spatial resolution.	Semi-direct	Quantitative thematic assessment	larger than 1km2/30 arc-second

Code	Description	Concept alignment	Base data evaluation	Spatial resolution
T2.4	Terrestrial ecoregions containing occurrences of warm temperate laurophyll forests were identified by consulting available ecoregion descriptions (Dinerstein et al., 2017), global and regional reviews, national and regional ecosystem maps, locations of relevant examples, and author expertise. Consequently, they are coarse-scale indicative representations of distribution, except where they occupy small ecoregions. Ecoregions were mapped at 30 arc second spatial resolution. Ecoregions were designated as containing major occurrences if ecoregion descriptions mentioned extensive areas of laurophyll forests, or as minor occurences if laurophyll forests were described as patches within a matrix dominated by other ecosystems.	Indirect	Quantitative thematic assessment	larger than 1km2/30 arc-second
T2.5	Remote sensing estimates of canopy height were used as a direct indicator of the distribution of this group of tall forest ecosystems (Armston et al., 2015, Tang et al., 2019). All areas with tree canopies taller than 40m were selected and clipped to the spatial extent of temperate climate types (Beck et al., 2018). Mapped occurrences were then aggregated to half degree spatial resolution and reclassified as major occurrences (>20% of cell area) and minor occurrences (< 20% of cell area).	Semi-direct	Quantitative accuracy assessment	30 arc-second (ca. 1km2) or better
T2.6	Sclerophyll forests were initially identified using consensus land-cover maps (Tuanmu et al., 2014) and then cropped to selected terrestrial ecoregions (Dinerstein et al., 2017) at 30 arc seconds spatial resolution. Ecoregions were selected if: i) their descriptions mentioned features consistent with those identified in the profile of the Ecosystem Functional Group; and ii) if their location was consistent with the ecological drivers described in the profile. Ecoregions were designated as containing major occurrences if ecoregion descriptions mentioned extensive areas of sclerophyll forests or as minor occurences if sclerophyll forests were described as patches within a matrix dominated by other ecosystems.	Indirect	Quantitative accuracy assessment	30 arc-second (ca. 1km2) or better
T3.1	Terrestrial ecoregions containing occurrences of tropical heathlands were identified by consulting available ecoregion descriptions (Dinerstein et al., 2017), global and regional reviews, national and regional ecosystem maps, locations of relevant examples, and author expertise. Consequently, they are coarse-scale indicative representations of distribution, except where they occupy small ecoregions. Ecoregions were mapped at 30 arc second spatial resolution. Ecoregions were designated as containing major occurrences if ecoregion descriptions mentioned extensive areas of tropical heathlands, or as minor occurences if these heathlands were described as patches within a matrix dominated by other ecosystems.	Indirect	Quantitative thematic assessment	larger than 1km2/30 arc-second

Code	Description	Concept alignment	Base data evaluation	Spatial resolution
T3.2	Terrestrial ecoregions containing occurrences of seasonal temperate heathlands were identified by consulting available ecoregion descriptions (Dinerstein et al., 2017), global and regional reviews, national and regional ecosystem maps, locations of relevant examples, and author expertise. Consequently, they are coarse-scale indicative representations of distribution, except where they occupy small ecoregions. Ecoregions were mapped at 30 arc second spatial resolution. Ecoregions were designated as containing major occurrences if ecoregion descriptions mentioned extensive areas of seasonal temperate heathlands, or as minor occurences if these heathlands were described as patches within a matrix dominated by other ecosystems.	Indirect	Quantitative thematic assessment	larger than 1km2/30 arc-second
T3.3	Cool temperate heathlands were mapped using consensus land-cover maps (Tuanmu et al., 2014; Latifovic et al., 2016), cropped to selected terrestrial ecoregions at 30 arc seconds spatial resolution (Dinerstein et al., 2017; CEC 1997). Ecoregions were selected if they contained areas mentioned or mapped in published regional studies (Loidi et al., 2015; Luebert & Pliscoff, 2017), or if: i) their descriptions mentioned features consistent with those identified in the profile of the Ecosystem Functional Group; and ii) if their location was consistent with the ecological drivers described in the profile. Ecoregions were designated as containing major occurrences if ecoregion descriptions mentioned extensive areas of lowland rainforests or as minor occurences if lowland rainforests were described as patches within a matrix dominated by other ecosystems.	Semi-direct	Quantitative accuracy assessment	30 arc-second (ca. 1km2) or better
T3.4	Rocky pavements and screes were initially identified using consensus land-cover maps (Tuanmu et al., 2014) and then cropped to selected terrestrial ecoregions (Dinerstein et al., 2017) at 30 arc seconds spatial resolution. Ecoregions were selected if: i) their descriptions mentioned features consistent with those identified in the profile of the Ecosystem Functional Group; and ii) if their location was consistent with the ecological drivers described in the profile. Ecoregions were designated as containing major occurrences if ecoregion descriptions mentioned extensive areas of rocky pavements or screes or as minor occurences if these ecosystems were described as patches within a matrix dominated by other ecosystems.	Semi-direct	Quantitative accuracy assessment	30 arc-second (ca. 1km2) or better

Code	Description	Concept alignment	Base data evaluation	Spatial resolution
T4.1	Trophic savannas were initially identified using consensus land-cover maps (Tuanmu et al., 2014) and then cropped to selected terrestrial ecoregions (Dinerstein et al., 2017) at 30 arc seconds spatial resolution. Ecoregions were selected if: i) their descriptions mentioned features consistent with those identified in the profile of the Ecosystem Functional Group; and ii) if their location was consistent with the ecological drivers described in the profile. Ecoregions were designated as containing major occurrences if ecoregion descriptions mentioned extensive areas of savannas or as minor occurences if these ecosystems were described as patches within a matrix dominated by other ecosystems.	Indirect	Quantitative accuracy assessment	30 arc-second (ca. 1km2) or better
T4.2	Terrestrial ecoregions containing occurrences of pyric tussock savannas were identified by consulting available ecoregion descriptions (Dinerstein et al., 2017), global and regional reviews, national and regional ecosystem maps, locations of relevant examples, and author expertise. Consequently, they are coarse-scale indicative representations of distribution, except where they occupy small ecoregions. Ecoregions were mapped at 30 arc second spatial resolution. Ecoregions were designated as containing major occurrences if ecoregion descriptions mentioned extensive areas of pyric savannas, or as minor occurences if these ecosystems were described as patches within a matrix dominated by other ecosystems.	Indirect	Quantitative thematic assessment	larger than 1km2/30 arc-second
T4.3	The distribution of Hummock savannas in Australia was mapped from data sets compiled by Keith and Tozer (2017) from the Australian National Vegetation Information System (NVIS 2016). The original mapping was done by remote sensing with field reconnaissance. It was mapped at 30 arc second spatial resolution.	Direct	Qualitative expert assessment	30 arc-second (ca. 1km2) or better
T4.4	Terrestrial ecoregions containing occurrences of temperate woodlands were identified by consulting available ecoregion descriptions (Dinerstein et al., 2017), global and regional reviews, national and regional ecosystem maps, locations of relevant examples, and author expertise. Consequently, they are coarse-scale indicative representations of distribution, except where they occupy small ecoregions. Ecoregions were mapped at 30 arc second spatial resolution. Ecoregions were designated as containing major occurrences if ecoregion descriptions mentioned extensive areas of temperate woodlands, or as minor occurences if these ecosystems were described as patches within a matrix dominated by other ecosystems.	Indirect	Quantitative thematic assessment	larger than 1km2/30 arc-second

Code	Description	Concept alignment	Base data evaluation	Spatial resolution
T4.5	Terrestrial ecoregions containing occurrences of temperate grasslands were identified by consulting available ecoregion descriptions (Dinerstein et al., 2017), global and regional reviews, national and regional ecosystem maps, locations of relevant examples, and author expertise. Consequently, they are coarse-scale indicative representations of distribution, except where they occupy small ecoregions. Ecoregions were mapped at 30 arc second spatial resolution. Ecoregions were designated as containing major occurrences if ecoregion descriptions mentioned extensive areas of temperate grasslands, or as minor occurences if these ecosystems were described as patches within a matrix dominated by other ecosystems.	Indirect	Quantitative thematic assessment	larger than 1km2/30 arc-second
T5.1	Semi-desert steppes were initially identified using consensus land-cover maps (Tuanmu et al., 2014) and then cropped to selected terrestrial ecoregions (Dinerstein et al., 2017) at 30 arc seconds spatial resolution. Ecoregions were selected if: i) their descriptions mentioned features consistent with those identified in the profile of the Ecosystem Functional Group; and ii) if their location was consistent with the ecological drivers described in the profile. Ecoregions were designated as containing major occurrences if ecoregion descriptions mentioned extensive areas of semi-desert steppes or as minor occurences if these ecosystems were described as patches within a matrix dominated by other ecosystems.	Indirect	Quantitative accuracy assessment	30 arc-second (ca. 1km2) or better
T5.2	Succulent or thorny deserts were initially identified using consensus land-cover maps (Tuanmu et al., 2014) and then cropped to selected terrestrial ecoregions (Dinerstein et al., 2017) at 30 arc seconds spatial resolution. Ecoregions were selected if: i) their descriptions mentioned features consistent with those identified in the profile of the Ecosystem Functional Group; and ii) if their location was consistent with the ecological drivers described in the profile. Ecoregions were designated as containing major occurrences if ecoregion descriptions mentioned extensive areas of succulent deserts or as minor occurences if these ecosystems were described as patches within a matrix dominated by other ecosystems.	Indirect	Quantitative accuracy assessment	30 arc-second (ca. 1km2) or better
T5.3	The distribution of Sclerophyll deserts was mapped from data sets for arid sclerophyll shrublands and hummock grasslands compiled by Keith and Tozer (2017) from the Australian National Vegetation Information System (NVIS 2016). The original mapping was done by remote sensing with field reconnaissance. It was mapped at 30 arc second spatial resolution.	Direct	Qualitative expert assessment	30 arc-second (ca. 1km2) or better

Code	Description	Concept alignment	Base data evaluation	Spatial resolution
T5.4	Cold deserts were initially identified using consensus land-cover maps (Tuanmu et al., 2014) and then cropped to selected terrestrial ecoregions (Dinerstein et al., 2017) at 30 arc seconds spatial resolution. Ecoregions were selected if: i) their descriptions mentioned features consistent with those identified in the profile of the Ecosystem Functional Group; and ii) if their location was consistent with the ecological drivers described in the profile. Ecoregions were designated as containing major occurrences if ecoregion descriptions mentioned extensive areas of cold deserts or as minor occurences if these ecosystems were described as patches within a matrix dominated by other ecosystems.	Indirect	Quantitative accuracy assessment	30 arc-second (ca. 1km2) or better
T5.5	Hyper-arid deserts were initially identified using consensus land-cover maps (Tuanmu et al., 2014) and then cropped to selected terrestrial ecoregions (Dinerstein et al., 2017) at 30 arc seconds spatial resolution. Ecoregions were selected if: i) their descriptions mentioned features consistent with those identified in the profile of the Ecosystem Functional Group; and ii) if their location was consistent with the ecological drivers described in the profile. Ecoregions were designated as containing major occurrences if ecoregion descriptions mentioned extensive areas of hyper-arid deserts or as minor occurences if these ecosystems were described as patches within a matrix dominated by other ecosystems.	Semi-direct	Quantitative accuracy assessment	30 arc-second (ca. 1km2) or better
T6.1	Areas of permanent snow were identified from consensus land-cover maps (Tuanmu et al., 2014), glacier inventories (Raup et al., 2007; GLIMS and NSIDC, 2005-2018) and the Antarctic Land Cover map for 2000 (Hui et al., 2017). A composite map was created at 30 arc seconds spatial resolution in geographic projection, occurrences were then aggregated to half degree spatial resolution and reclassified as major occurrences (cells with > 22% snow coverage) and minor occurrences (cells with at least one occurrence).	Direct	Quantitative accuracy assessment	30 arc-second (ca. 1km2) or better

Code	Description	Concept alignment	Base data evaluation	Spatial resolution
T6.2	Known locations of prominent ice-free rock in glacial and alpine environments were selected from global geographical gazeteers (GeoNames, 2020), glacier inventories (Raup et al 2007; GLIMS and NSIDC, 2005-2018) and the Antarctic Land Cover map for 2000 (Hui et al., 2017). Further areas with mixed occurrence of barren and snow/ice cover were identified from the Circumpolar Arctic Vegetation Map (Raynolds et al., 2019), the USGS EROS LandCover GLCCDB, version 2 (Loveland et al., 2000) and a 1km consensus land-cover map (Tuanmu et al., 2014). A composite map was created at 30 arc seconds spatial resolution in geographic projection, occurrences were then aggregated to half degree cells. Cells containing at least one known location were designated major occurrences, while those mapped as mixed barren and snow/ice cover were designated as minor occurrences if snow/ice covered at least 2.5% of the cell area.	Direct	Quantitative accuracy assessment	30 arc-second (ca. 1km2) or better
T6.3	Areas corresponding to the tundra climatic zone according to the Köppen-Geiger classification system (Beck et al., 2018) were first identified. Additional areas were then selected in high latitudes corresponding with low annual solar radiation (values <1800 in Beckmann et al., 2014). A union of these maps was created at 30 arc seconds spatial resolution in geographic projection, occurrences were then aggregated to half degree spatial resolution and reclassified cells as major occurrences (>80% of cell area) and minor occurrences (30-80% of cell area).	Semi-direct	Quantitative accuracy assessment	30 arc-second (ca. 1km2) or better
T6.4	Terrestrial ecoregions containing occurrences of temperate alpine ecosystems were identified by consulting available ecoregion descriptions (Dinerstein et al., 2017), global and regional reviews, national and regional ecosystem maps, locations of relevant examples, and author expertise. Consequently, they are coarse-scale indicative representations of distribution, except where they occupy small ecoregions. Ecoregions were mapped at 30 arc second spatial resolution. Ecoregions were designated as containing major occurrences if ecoregion descriptions mentioned extensive areas of temperate alpine ecosystems, or as minor occurences if these ecosystems were described as patches within a matrix dominated by other ecosystems.	Indirect	Quantitative thematic assessment	larger than 1km2/30 arc-second

Code	Description	Concept alignment	Base data evaluation	Spatial resolution
T6.5	Tropical alpine grasslands were initially identified using consensus land-cover maps (Tuanmu et al., 2014) and then cropped to selected terrestrial ecoregions (Dinerstein et al., 2017) at 30 arc seconds spatial resolution. Ecoregions were selected if: i) their descriptions mentioned features consistent with those identified in the profile of the Ecosystem Functional Group; and ii) if their location was consistent with the ecological drivers described in the profile. Ecoregions were designated as containing major occurrences if ecoregion descriptions mentioned extensive areas of tropical alpine grasslands or as minor occurences if these ecosystems were described as patches within a matrix dominated by other ecosystems.	Semi-direct	Quantitative accuracy assessment	30 arc-second (ca. 1km2) or better
T7.1	Major occurrences of croplands were taken from the map of Habitat type 14.1 by Jung et al. (2020) based on the IUCN Habitats Classification Scheme v3.1 (IUCN 2012). We compared this to cropping areas in consensus land-cover maps (Tuanmu et al., 2014) and found that maps of Jung et al. (2020) more closely matched the concept of T7.1. Occurrences were extracted from fractional aggregated 1 km resolution base data (Jung et al. 2020), approximating 30 arc second spatial resolution.	Direct	Quantitative accuracy assessment	30 arc-second (ca. 1km2) or better

Code	Description	Concept alignment	Base data evaluation	Spatial resolution
T7.2	Mapping of intensive livestock pastures was based on fractional land use mapping (Ramankutty et al. 2008), dasymetric estimates of ruminant livestock density for cattle and sheep (Gilbert et al. 2010), and Human Appropriation of Net Primary Production (HANPP, Haberl et al. 2007). Fractional land use cover indicated firstly where pastures occur and secondly where they occupy a large portion of area relative to croplands. This helped to exclude intensive croplands that are also used to graze livestock, either through temporal rotation or on the margins of cropped paddocks (e.g. in south Asia). Livestock densities indicated where ruminants were important components of pasture systems, and helped exclude some rangelands with low livestock densities. Finally, HANPP helped exclude low productivity rangelands with high stocking rates and additional areas of cropland. Mapped outputs of different combinations and thresholds for the input data layers were visually inspected in South Asia, Australia, West Africa, and North and South America. Major occurrences were mapped where pasture area fraction greater than zero (PAF>0) and greater than cropland area fraction (PAF-CAF>0), densities of cattle or sheep were greater than 500 per cell, and 100 < HANPP < 700 gC/m²/yr. Examination of the sensitivity of mapped area to variation in these thresholds indicated no appreciatble change in the global mapped area when livestock density was varied by ±20% and marginal change in mapped area with variation in the other thresholds by the same amount. To represent this uncertainty, we mapped minor occurrences as the additional area where PAF>0, PAF-CAP>-0.2 and 80 < HANPP < 840 gC/m²/yr. We acknowledge significant untested assumptions and limitations on spatial predictors that challenge the global-scale delineation of pasture ecosystems with varied levels of human influence. We therefore advise appropriate caution in use of the spatial data for quantitative analysis.	Indirect	Quantitative accuracy assessment	larger than 1km2/30 arc-second
T7.3	Major occurrences of plantations were taken from the map of Habitat type 14.3 by Jung et al. (2020) based on the IUCN Habitats Classification Scheme v3.1 (IUCN 2012). We compared this to cropping areas in consensus land-cover maps (Tuanmu et al., 2014) and found that maps of Jung et al. (2020) more closely matched the concept of T7.3. Occurrences were extracted from fractional aggregated 1 km resolution base data (Jung et al. 2020), approximating 30 arc second spatial resolution.	Direct	Quantitative accuracy assessment	30 arc-second (ca. 1km2) or better
T7.4	The distribution of urban and industrial infrastructure lands was taken from a global land use/land cover map (LULC class 7 'built areas') for the year 2020 at 10 metre resolution (Karra et al. 2021). Class 7 includes major road and rail networks, large homogenous impervious surfaces including parking structures, office buildings and residential housing, dense. Sparse villages may not be represented. We calculated the proportion of built area per square kilometre and applied a threshold of 1 to 5 % for minor occurrences and >5% for major occurrences.	Direct	Quantitative accuracy assessment	30 arc-second (ca. 1km2) or better

Code	Description	Concept alignment	Base data evaluation	Spatial resolution
T7.5	The distribution of semi-natural pastures is poorly documented at global scales. Areas where these ecosystems are most likely to occur were estimated from land use and land suitability datasets for the year 2000 (Erb et al. 2007) and Human Appropriation of Net Primary Productivity (Haberl et al. 2007). Areas were included on the map if they met the following conditions based on the available spatial data if they: a) were mapped as suitable for livestock grazing; b) had a greater proportion of grazing lands than croplands or forestry lands (grazing> forestry and grazing>cropland); c) had intermediate to high cover of grazing lands (>30%); and intermediate to high estimates of Human Appropriation of Net Primary Productivity (20%<HANPP <90%). In combination, these conditions were assumed to exclude intensive pastures and wild rangelands with low productivity for livestock grazing. We classified areas with the highest grazing suitability (class 1 in Erb et al. 2007) as major occurrences, and areas with the second highest grazing suitability (class 2) as minor occurrences. We acknowledge significant untested assumptions and limitations on spatial predictors that challenge the global-scale delineation of pasture ecosystems with varied levels of human influence. We therefore advise appropriate caution in use of the spatial data for quantitative analysis.	Indirect	Quantitative accuracy assessment	larger than 1km2/30 arc-second
S1.1	Distributions of aerobic caves were based on mapped area of carbonate rock outcrop (Williams & Ting Fong 2016). This provides an bound limit for the area of exposed karst terrain, as not all carbonate rocks are karstified. Lava tubes and other rocks that may contain these ecosystem functional groups are not shown on this indicative map, but are less extensive than those in carbonate rock.	Indirect	Qualitative expert assessment	larger than 1km2/30 arc-second
S1.2	Global distribution throughout the earth's crust.	.	.	.
S2.1	Freshwater ecoregions (Abell et al., 2008) containing urban and industrialised areas with water transfer infrastructure were identified by consulting available ecoregion descriptions (http://www.feow.org/), maps of irrigation and other water infrastructure, and expertise of authors. Due to uncertainty and limited verification and likely limited spatial extent within mapped areas, all inferred occurrences were shown as minor at 30 arc seconds spatial resolution	Indirect	Quantitative thematic assessment	larger than 1km2/30 arc-second
SF1.1	Distributions of Underground streams and pools were based on mapped area of carbonate rock outcrop (Williams & Ting Fong 2016). This. provides an upper bound for the area of exposed karst terrain, as not all carbonate rocks are karstified. Lava tubes and other rocks that may contain these ecosystem functional groups are not shown on this indicative map, but are less extensive than those in carbonate rock.	Indirect	Qualitative expert assessment	larger than 1km2/30 arc-second

Code	Description	Concept alignment	Base data evaluation	Spatial resolution
SF1.2	Indicative global maps of Groundwater aquifers were based on Bundesanstalt für Geowissenschaften und Rohstoffe & UNESCO (2012) in major groundwater basins with types 13-15 designated as major occurrences and types 11-12 designated as minor occurrences.	Direct	Obtained from facility with high data standards	larger than 1km2/30 arc-second
SF2.1	Freshwater ecoregions (Abell et al., 2008) containing urban and industrialised areas with water transfer infrastructure were identified by consulting available ecoregion descriptions (http://www.feow.org/), maps of irrigation and other water infrastructure, and expertise of authors. Due to uncertainty and limited verification and likely limited spatial extent within mapped areas, all inferred occurrences were shown as minor at 30 arc seconds spatial resolution.	Indirect	Qualitative expert assessment	larger than 1km2/30 arc-second
SF2.2	Point records of flooded mines were compiled from public databases (https://www.unexmin.eu/the-european-inventory-of-flooded-mines-is-now-online/), an internet search for "flooded mines" and locations of deep mines inferred from world mineral resources spatial data (USGS: https://mrdata.usgs.gov/). Terrestrial ecoregions (Dinerstein et al., 2017) with concentrations of these records were selected to represent an indicative global distribution of flooded mines at 30 arc seconds spatial resolution.	Semi-direct	Known records	variable (points)
SM1.1	Indicative distributions of anchialine caves and pools were based on mapped areas of carbonate rock outcrop (Williams & Ting Fong, 2016) and lava flows intersecting the coast, which were aggregated within a template of 1-degree grid cells.	Indirect	Qualitative expert assessment	variable (polygons)
SM1.2	Indicative distributions of anchialine caves and pools were based on mapped areas of carbonate rock outcrop (Williams & Ting Fong, 2016) and lava flows intersecting the coast, which were aggregated within a template of 1-degree grid cells.	Indirect	Qualitative expert assessment	ca. 1km2
SM1.3	Marine ecoregions (Spalding et al., 2008) containing occurrences of rocky coastline (see MT1.1) were verified by inspection of imagery available in Google Earth to identify an envelope of potential distribution for sea caves. The coastlines within these ecoregions were summarised using a template of 1-degree grid cell intersected with the coast. As caves represent a small portion of such coastlines, all mapped areas were designated as minor occurrences.	Indirect	Qualitative expert assessment	larger than 1km2/30 arc-second

Code	Description	Concept alignment	Base data evaluation	Spatial resolution
TF1.1	Major occurrences of tropical swamp forest and flooded forest were taken from the map of Habitat type 1.8 by Jung et al. (2020) based on the IUCN Habitats Classification Scheme v3.1 (IUCN 2012). We compared this to areas of of tropical swamp forest and flooded forest mapped Global Lakes and Wetlands Database (Lehner and Döll, 2004) as well as ecoregions with such forests mentioned in their description (Dinerstein et al., 2017), and found that maps of Jung et al. (2020) more closely matched the concept of TF1.1. Occurrences were extracted from fractional aggregated 1 km resolution base data (Jung et al. 2020), approximating 30 arc second spatial resolution.	Direct	Quantitative accuracy assessment	30 arc-second (ca. 1km2) or better
TF1.2	Terrestrial ecoregions containing occurrences of temperate forested wetlands were identified by consulting available ecoregion descriptions (Dinerstein et al., 2017), global and regional reviews, national and regional ecosystem maps, locations of relevant examples, and author expertise. Consequently, they are coarse-scale indicative representations of distribution, and all ecoregions were mapped as minor occurrences. Ecoregions were mapped at 30 arc second spatial resolution.	Indirect	Quantitative thematic assessment	larger than 1km2/30 arc-second
TF1.3	Terrestrial ecoregions containing occurrences of permanent floodplain marshes were identified by consulting available ecoregion descriptions (Dinerstein et al., 2017), global and regional reviews, national and regional ecosystem maps, locations of relevant examples, and author expertise. Consequently, they are coarse-scale indicative representations of distribution. Mapped ecoregions were designated as major occurrences where the ecoregions were small and marshes dominat most of their area based on the text descriptions and inspection of Google Earth imagery. The remaining ecoregions were designated as minor occurrences. Ecoregions were mapped at 30 arc second spatial resolution.	Indirect	Quantitative thematic assessment	larger than 1km2/30 arc-second
TF1.4	Major occurrences of freshwater marshes and floodplains were taken from the Global Lakes and Wetlands Database (Lehner and Döll, 2004). Occurrences in boreal and polar climates were excluded by removing KoeppnGeiger_classes>26 in Beck et al., (2018). Additional areas with minor occurrences were identified in selected freshwater ecoregions (Abell et al., 2008). Ecoregions were selected if: i) their descriptions mentioned features consistent with those identified in the profile of the Ecosystem Functional Group; and ii) if their location was consistent with the ecological drivers described in the profile. Occurrences were aggregated to half degree spatial resolution.	Direct	Quantitative accuracy assessment	30 arc-second (ca. 1km2) or better, variable (polygons)

Code	Description	Concept alignment	Base data evaluation	Spatial resolution
TF1.5	Major occurrences of ephemeral floodplains were mapped from remote sensing estimates of ephemeral surface water (classes 4, 5 and 8 from Pekel et al., 2016) within selected ecoregions. Freshwater ecoregions (Abell et al., 2008) were identified as containing occurrences of this functional group if: i) their descriptions mentioned features consistent with those identified in the profile of the Ecosystem Functional Group; and ii) if their location was consistent with the ecological drivers described in the profile. The remaining areas of selected ecoregions were mapped as containing minor occurrences. Map data were presented at 0.5 minute spatial resolution	Indirect	Quantitative accuracy assessment	30 arc-second (ca. 1km2) or better
TF1.6	Terrestrial ecoregions containing major or minor occurrences of this ecosystem functional group were identified by consulting available ecoregion descriptions (Dinerstein et al., 2017), global and regional reviews, national and regional ecosystem maps, locations of relevant examples, and proofed by expert reviewers. Consequently, they are coarse-scale indicative representations of distribution, except where they occupy small ecoregions. Ecoregions were mapped at 30 arc second spatial resolution.	Indirect	Quantitative thematic assessment	larger than 1km2/30 arc-second
TF1.7	Terrestrial ecoregions containing major or minor occurrences of this ecosystem functional group were identified by consulting available ecoregion descriptions (Dinerstein et al., 2017), global and regional reviews, national and regional ecosystem maps, locations of relevant examples, and proofed by expert reviewers. Consequently, they are coarse-scale indicative representations of distribution, except where they occupy small ecoregions. Ecoregions were mapped at 30 arc second spatial resolution.	Indirect	Quantitative thematic assessment	larger than 1km2/30 arc-second
F1.1	Major occurrences were mapped by intersecting selected ecoregions with the distribution of 1st-3rd order streams taken from the RiverATLAS (v1.0) database (Linke et al., 2019) clipped to exclude cold or dry climates (i.e. excluding areas with mean temperature of coldest quarter <0°C, mean annual precipitation <300mm) based on data from Karger et al. (2017). Freshwater ecoregions (Abell et al., 2008) were identified as containing permanent upland streams if: i) their descriptions mentioned features consistent with those identified in the profile of the Ecosystem Functional Group; and ii) if their location was consistent with the ecological drivers described in the profile. The remaining areas within selected ecoregions, clipped as above to exclude cold or dry climates, were designated as minor occurrences. Maps data were presented at 30 arc seconds spatial resolution.	Semi-direct	Obtained from facility with high data standards	30 arc-second (ca. 1km2) or better

Code	Description	Concept alignment	Base data evaluation	Spatial resolution
F1.2	Major occurrences were mapped within selected freshwater ecoregions using stream orders 4-9 taken from the RiverATLAS (v1.0) database (Linke et al. 2019) combined with global estimates of surface water phenology (classes 1, 2 and 7 from Pekel et al., 2016). Freshwater ecoregions (Abell et al., 2008) were identified as containing occurrences of this functional group if: i) their descriptions mentioned features consistent with those identified in the profile of the Ecosystem Functional Group; and ii) if their location was consistent with the ecological drivers described in the profile. The remaining area of selected ecoregions was designated as minor occurrences. Occurrences were aggregated to ten minute spatial resolution.	Semi-direct	Obtained from facility with high data standards	30 arc-second (ca. 1km2) or better
F1.3	Major occurrences of freeze-thaw rivers and streams was mapped from the Global River Classification database (Ouellet Dallaire et al., 2018), including all reaches with minimum temperature below 0°C. Freshwater ecoregions (Abell et al., 2008) were identified as containing minor occurrences of this functional group if: i) their descriptions mentioned features consistent with those identified in the profile of the Ecosystem Functional Group; and ii) if their location was consistent with the ecological drivers described in the profile. Occurrences were aggregated to ten minute spatial resolution.	Direct	Obtained from facility with high data standards	30 arc-second (ca. 1km2) or better
F1.4	Within selected ecoregions, major occurrences were mapped using 1st-4th order streams (3km buffer) taken from the RiverATLAS (v1.0) database (Linke et al., 2019). Freshwater ecoregions (Abell et al., 2008) were identified as containing occurrences of this functional group if: i) their descriptions mentioned features consistent with those identified in the profile of the Ecosystem Functional Group; and ii) if their location was consistent with the ecological drivers described in the profile. The remaining areas of selected ecoregions were mapped as minor occurrences. Occurrences were aggregated to ten minute spatial resolution.	Semi-direct	Obtained from facility with high data standards	30 arc-second (ca. 1km2) or better
F1.5	Major occurrences were mapped within selected freshwater ecoregions using stream orders 4-9 taken from the RiverATLAS (v1.0) database (Linke et al. 2019) combined with global estimates of surface water phenology (classes 1, 2 and 7 from Pekel et al., 2016). Freshwater ecoregions (Abell et al., 2008) were identified as containing occurrences of this functional group if: i) their descriptions mentioned features consistent with those identified in the profile of the Ecosystem Functional Group; and ii) if their location was consistent with the ecological drivers described in the profile. The remaining area of selected ecoregions was designated as minor occurrences. Occurrences were aggregated to ten minute spatial resolution.	Semi-direct	Obtained from facility with high data standards	30 arc-second (ca. 1km2) or better

Code	Description	Concept alignment	Base data evaluation	Spatial resolution
F1.6	Major occurrences of ephemeral streams were mapped by intersecting streams of all orders taken from the MERIT Hydro river channels dataset (Yamazaki et al. 2019) with remote sensing estimates of ephemeral surface water (classes 4, 5 and 8 from Pekel et al., 2016) within selected ecoregions. Data were aggregated at 30 arc-second resolution. Freshwater ecoregions (Abell et al., 2008) were identified as containing occurrences of this functional group if: i) their descriptions mentioned features consistent with those identified in the profile of the Ecosystem Functional Group; and ii) if their location was consistent with the ecological drivers described in the profile. The remaining river channels within the selected ecoregions were mapped as containing minor occurrences.	Indirect	Quantitative accuracy assessment	30 arc-second (ca. 1km2) or better
F1.7	The distribution of large lowland rivers was taken from the Global River Classification database (Ouellet Dallaire et al., 2018). Reaches with flow > 10,000 m3/s were mapped with a 20 km buffer (added for display) as major occurrences, clipped to exclude those with minimum temperature <0°C.	Direct	Obtained from facility with high data standards	variable (points)
F2.1	Locations of large lakes (>100km2) were taken from the HydroLAKES database (Messager et al., 2016) and combined with global estimates of permanent surface water surfaces (classes 1, 2 and 7 from Pekel et al., 2016). Freeze/thaw lakes (F2.3) in cold climates (approximated by mean temperature of coldest quarter < -10°C, Beck et al., 2018) were excluded. Occurrences were aggregated to 30 arc second spatial resolution. Grid cells with at least one lake >1000km2 were designated major occurrences, those only with lakes 100-1000km2 were designated minor occurrences.	Direct	Quantitative accuracy assessment	30 arc-second (ca. 1km2) or better
F2.10	Major occurrences of subglacial lakes were mapped as 0.5 degree cells containing the point records of Wright and Siegert (2012), Bowling et al., (2019), Thór Marteinsson et al. (2013) and Livingstone et al. (2016). Unmapped lakes are likely to occur within areas with permanent snow and ice cover and were mapped as minor occurrences based on permanent snow and ice from Dinerstein et al. (2017) and Tuanmu et al. (2014).	Direct	Known records	variable (points)

Code	Description	Concept alignment	Base data evaluation	Spatial resolution
F2.2	Within selected freshwater ecoregions (2008), major occurrences of small permanent lakes (<100km2), were mapped by taking water body types 1 and 3 (which exclude artificial lakes), from the HydroLAKES database (Messager et al., 2016) and combining them with global estimates of surface water phenology (classes 1, 2 and 7 from Pekel et al., 2016). Freshwater ecoregions (Abell et al., 2008) were identified as containing occurrences of these functional groups if: i) their descriptions mentioned features consistent with those identified in the profile of the Ecosystem Functional Group; and ii) if their location was consistent with the ecological drivers described in the profile. Occurrences were aggregated to 10 minutes spatial resolution. Grid cells occupied by ≥6km2 of lakes were designated as major occurrences, while those with <6km2 and >1km2 of lakes were designated minor occurrences.	Semi-direct	Quantitative accuracy assessment	30 arc-second (ca. 1km2) or better
F2.3	Within selected freshwater ecoregions (2008), major occurrences of small permanent lakes (<100km2), were mapped by taking water body types 1 and 3 (which exclude artificial lakes), from the HydroLAKES database (Messager et al., 2016) and combining them with global estimates of surface water phenology (classes 1, 2 and 7 from Pekel et al., 2016). Freshwater ecoregions (Abell et al., 2008) were identified as containing occurrences of these functional groups if: i) their descriptions mentioned features consistent with those identified in the profile of the Ecosystem Functional Group; and ii) if their location was consistent with the ecological drivers described in the profile. Occurrences were aggregated to 10 minutes spatial resolution. Grid cells occupied by ≥6km2 of lakes were designated as major occurrences, while those with <6km2 and >1km2 of lakes were designated minor occurrences.	Semi-direct	Quantitative accuracy assessment	30 arc-second (ca. 1km2) or better
F2.4	Major occurrences of freeze-thaw lakes were mapped by taking water body types 1 and 3 of all sizes from the HydroLAKES database with minimum temperature below 0°C (Linke et al., 2019). Occurrences were aggregated to ten minute spatial resolution. Grid cells occupied by ≥6km2 of lakes were designated as major occurrences, while those with <6km2 and >1km2 of lakes were designated minor occurrences.	Direct	Obtained from facility with high data standards	30 arc-second (ca. 1km2) or better
F2.5	Major occurrences of natural ephemeral freshwater lakes were mapping by intersecting the estimated distribution of ephemeral surface water (classes 9 and 10 from Pekel et al., 2016) with global lake databases (Lehner and Döll, 2004; types 1 an 3 from Messager et al., 2016), excluding those from endorheic basins cf. F2.7 (Linke et al., 2019). Occurrences were aggregated to 10 minutes spatial resolution. Grid cells occupied by ≥6km2 of lakes were designated as major occurrences, while those with <6km2 and >1km2 of lakes were designated minor occurrences.	Semi-direct	Obtained from facility with high data standards	30 arc-second (ca. 1km2) or better

Code	Description	Concept alignment	Base data evaluation	Spatial resolution
F2.6	Major occurrences of permanent salt and soda lakes were compiled from a list of known salt lakes in Wurtsbaugh et al., (2017) and augmented by the authors, then matched with names in the HydroLAKES database to identify natural lakes (types 1 and 3 of Messager et al., 2016). Minor occurrences were mapped within arid and semi-arid parts of selected freshwater ecoregions (Abell et al., 2008) by clipping ecoregions to exclude areas with mean annual rainfall >250mm (Harris et al., 2014a). Freshwater ecoregions (Abell et al., 2008) were selected if they contained occurrences of permanent salt or soda lakes if: i) their descriptions mentioned features consistent with those identified in the profile of the Ecosystem Functional Group; and ii) if their location was consistent with the ecological drivers described in the profile. Occurrences were aggregated to 10 minutes spatial resolution.	Semi-direct	Quantitative accuracy assessment	30 arc-second (ca. 1km2) or better
F2.7	Ephemeral salt lakes were mapped by intersecting water bodies taken from global lake databases (Lehner and Döll, 2004; types 1 and 3 from Messager et al., 2016) with estimated ephemeral surface water (classes 9 and 10 from Pekel et al., 2016) and the distribution of arid and semi-arid, endorheic basins (Linke et al., 2019). Occurrences were aggregated to 10 minutes spatial resolution. Grid cells occupied by ≥6km2 of lakes were designated as major occurrences, while those with <6km2 and >1km2 of lakes were designated minor occurrences.	Semi-direct	Quantitative accuracy assessment	30 arc-second (ca. 1km2) or better
F2.8	Within selected freshwater ecoregions (2008), major occurrences of small permanent lakes (<100km2) were mapped by taking water body types 1 and 3 (which exclude artificial lakes), from the HydroLAKES database (Messager et al., 2016) and combining them with global estimates of surface water phenology (classes 1, 2 and 7 from Pekel et al., 2016). Freshwater ecoregions (Abell et al., 2008) were identified as containing occurrences of these functional groups if: i) their descriptions mentioned features consistent with those identified in the profile of the Ecosystem Functional Group; and ii) if their location was consistent with the ecological drivers described in the profile. The remaining area of selected ecoregions was designated as minor occurrences. Occurrences were aggregated to 10 minutes spatial resolution.	Indirect	Quantitative accuracy assessment	variable (polygons)

Code	Description	Concept alignment	Base data evaluation	Spatial resolution
F2.9	Within selected freshwater ecoregions (2008), major occurrences of small permanent lakes (<100km2), were mapped by taking water body types 1 and 3 (which exclude artificial lakes), from the HydroLAKES database (Messager et al., 2016) and combining them with global estimates of surface water phenology (classes 1, 2 and 7 from Pekel et al., 2016). Freshwater ecoregions (Abell et al., 2008) were identified as containing occurrences of these functional groups if: i) their descriptions mentioned features consistent with those identified in the profile of the Ecosystem Functional Group; and ii) if their location was consistent with the ecological drivers described in the profile. The remaining area of selected ecoregions was designated as minor occurrences. Occurrences were aggregated to 10 minutes spatial resolution.	Indirect	Qualitative expert assessment	30 arc-second (ca. 1km2) or better
F3.1	Polygons of reservoirs were obtained from water bodies in classes 2 and 3 in the HydroLakes database, except for those larger than 100 km2, as checking showed that these included a number of semi-regulated natural lakes (Messager et al. 2016). Additional point locations were taken from the Global Georeferenced Database of Dams (Mulligan et al. 2020), adding a spatial buffer of 15 arc-minutes to represent uncertainty in their exact location and extent. Major and minor occurrences were not distinguished .	Direct	Quantitative accuracy assessment	30 arc-second (ca. 1km2) or better
F3.2	A spatial buffer of 10 km was applied to generalise point locations of over 1 million small water bodies (area: 0.1 - 1 km2) obtained from the HydroLakes database (Messager et al. 2016). These areas were intersected with estimated the intensity of agricultural use using the mapped area fractions of pasture (PAF) and cropland (CAF) from Ramankutty et al. (2008), assuming that the majority of small water bodies within these intensive use areas are likely to be artificial. We classified the intersection of the lakes-buffer with PAF+CAF>0.5 as major occurrence and the intersection of the lakes-buffer with 0.05<(PAF+CAF)<0.5 as minor occurrences. The resulting map should show the main concentrations of constructed lacustrine wetlands but will underestimate occurrences in non-agricultural areas.	Indirect	Quantitative accuracy assessment	larger than 1km2/30 arc-second
F3.3	The distribution of rice paddies was estimated from the percentage of rice cover at a 5 arc minute resolution based on Monfreda et al. (2008). Cells with > 10% rice cover were designated as major occurrences, and those with 1-10% rice cover were designated as minor occurrences.	Direct	Quantitative accuracy assessment	larger than 1km2/30 arc-second

Code	Description	Concept alignment	Base data evaluation	Spatial resolution
F3.4	Direct data on the distribution of freshwater aquafarms are currently unavailable. To approximate the global distribution, freshwater ecoregions (Abell et al., 2008) were identified as containing minor occurrences of freshwater aquafarms if: i) their descriptions mentioned features consistent with those identified in the profile of the Ecosystem Functional Group; and ii) if their location was consistent with the ecological drivers described in the profile. The selections were checked by expert reviewers. Occurrences were mapped at 30 arc seconds spatial resolution.	Indirect	Qualitative expert assessment	larger than 1km2/30 arc-second
F3.5	A global map of irrigation for the year 2005 (Siebert et al. 2013) was used to map areas containing irrigation canals and a global land use/land cover (LULC) map for the year 2020 (Karra et al. 2021) to map built areas likely to contain drains and canals. Both maps were resampled and aggregated to a 30 arc-second (ca. 1km) resolution. The mapped areas were designated as major occurrences of canals, ditches and drains were designated where the percentage of area equipped for irrigation was >20% (Siebert et al. 2013) or the proportion of built area was >5% (Karra et al. 2021). Minor occurrences were designated where irrigation-equipped area was 10-20%) or where there was low cover of built area (1-5%). The irrigation map compiled by Siebert et al. (2013) was selected instead of a more recent one prepared by Nagaraj et al. (2021) because a comparison revealed more mapping artefacts in the latter data set.	Indirect	Quantitative accuracy assessment	30 arc-second (ca. 1km2) or better
FM1.1	Known locations of fjords were selected from a global geographical gazetteer (GeoNames, 2020) and the composite gazetteer of Antarctica (SCAR, 1992-2020). We further selected related coastal areas from a global coastal typology (Type IV in Dürr et al., 2011) and the adjacent marine shelves to 2000 metre depth (Becker et al., 2009). A composite map was created at 30 arc seconds spatial resolution in geographic projection, occurrences were then aggregated to half degree spatial resolution and reclassified as major occurrences (cells with at least one known occurrence) and minor occurrences (cells with > 5% occurrence of coastal/marine shelf areas). Minor occurrences were clipped to a 50km buffer along the coast to remove inland and oceanic areas.	Direct	Known records	30 arc-second (ca. 1km2) or better
FM1.2	Approximate distributions of permanently open coastal inlets were identified in marine ecoregions (Spalding et al., 2008) likely to contain occurrences based on inspection of coastal maps, imagery available in Google Earth and expertise of authors. Occurrences were converted to 30 arc second spatial resolution and clipped to a 50 km buffer along the coastline to exclude inland and offshore areas of the ecoregions.	Indirect	Qualitative expert assessment	larger than 1km2/30 arc-second

Code	Description	Concept alignment	Base data evaluation	Spatial resolution
FM1.3	Marine ecoregions (Spalding et al., 2008) containing major occurrences of intermittently closed and open coastal lagoons identified by McSweeney et al. (2017) were mapped and supplemented with minor occurrences identified by apprasial of imagery available in Google Earth and expertise of authors. Occurrences were converted to 30 arc second spatial resolution and clipped to a 50 km buffer along the coastline to exclude inland and offshore areas of the ecoregions.	Indirect	Qualitative expert assessment	larger than 1km2/30 arc-second
M1.1	Indicative maps of Seagrass meadows were obtained from UNEP-WCMC & Short (2017) based on Green & Short (2003). Occurrences were converted to 30 arc second spatial resolution.	Direct	Qualitative expert assessment	variable (polygons)
M1.2	Ecoregions with major and minor occurrences of Kelp forests were identified by overlaying a global map of kelp systems (Wernberg and Filbee-Dexter, 2019) on marine ecoregions (Spalding et al., 2008), and then clipping to bathymetry with <80m depth (Becker et al., 2009). Clipped ecoregions were assigned to major and minor occurrences based on information in Wernberg and Filbee-Dexter (2019) and author expertise, and proofed by specialist reviewers. Occurrences were converted to 30 arc second spatial resolution.	Direct	Known records	30 arc-second (ca. 1km2) or better
M1.3	Indicative maps of Photic coral reefs were obtained from Institute for Marine Remote Sensing et al. (2011). Occurrences were converted to 30 arc second spatial resolution.	Direct	Qualitative expert assessment	30 arc-second (ca. 1km2) or better
M1.4	Major and minor occurrences of shellfish beds and reefs were identified by overlaying a global map of oyster reefs (Beck et al., 2011) on marine ecoregions (Spalding et al., 2008), and then clipping to the extent of the marine 'shelf' base layer as mapped by Harris et al. (2014b). Occurrences were converted to 30 arc second spatial resolution.	Semi-direct	Known records	larger than 1km2/30 arc-second
M1.5	Photo-limited marine animal forests are widespread through the global extent of the marine shelf biome. Reliable data on their precise distribution are limited. To represent regional uncertainty, their indicative distributions were mapped through the full extent of the marine 'shelf' base layer as mapped by Harris et al. (2014b). Occurrences were converted to 30 arc second spatial resolution.	Indirect	Quantitative accuracy assessment	larger than 1km2/30 arc-second
M1.6	Subtidal rocky reefs are widespread through the global extent of the marine shelf biome. Reliable data on their precise distribution are limited. To represent regional uncertainty, their indicative distributions were mapped through the full extent of the marine 'shelf' base layer as mapped by Harris et al. (2014b). Occurrences were converted to 30 arc second spatial resolution.	Indirect	Quantitative accuracy assessment	larger than 1km2/30 arc-second

Code	Description	Concept alignment	Base data evaluation	Spatial resolution
M1.7	Subtidal sand beds are widespread through the global extent of the marine shelf biome. Reliable data on their precise distribution are limited. To represent regional uncertainty, their indicative distributions were mapped through the full extent of the marine 'shelf' base layer as mapped by Harris et al. (2014b). Occurrences were converted to 30 arc second spatial resolution.	Indirect	Quantitative accuracy assessment	larger than 1km2/30 arc-second
M1.8	Subtidal mudplains are widespread through the global extent of the marine shelf biome. Reliable data on their precise distribution are limited. To represent regional uncertainty, their indicative distributions were mapped through the full extent of the marine 'shelf' base layer as mapped by Harris et al. (2014b). Occurrences were converted to 30 arc second spatial resolution.	Indirect	Quantitative accuracy assessment	larger than 1km2/30 arc-second
M1.9	Marine ecoregions (Spalding et al., 2008) with major and minor occurrences of Upwelling zones were identified by consulting global and regional reviews (Hutchings et al. 1995; Cury et al. 2003), maps of relevant ecosystems and expertise of authors. The identified ecoregions were then clipped to the extent of the marine 'shelf' base layer as mapped by Harris et al. (2014b). Occurrences were converted to 30 arc second spatial resolution.	Indirect	Qualitative expert assessment	larger than 1km2/30 arc-second
M1.10	The distribution of Rhodolith/Maërl beds was mapped directly from distribution models of species that primarily form rhodoliths (Fragkopoulou et al. 2021). Models were evaluated by cross-validation and performed well (AUC ~0.9). Models for polar-cold temperate and tropicalwarm temperate affiliated species were combined (as in Fig 2 of Fragkopoulou et al. 2021). The data has a spatial resolution of 5 arc-minute.	Direct	Quantitative accuracy assessment	30 arc-second (ca. 1km2) or better
M2.1	Indicative distributions of these epipelagic ocean waters were based on bathymetric spatial data (Becker et al. 2009) using a depth range of 0-200m. Occurrences were mapped at 30 arc second spatial resolution.	Direct	Quantitative accuracy assessment	30 arc-second (ca. 1km2) or better
M2.2	Indicative distributions of these mesopelagic ocean waters were based on bathymetric spatial data (Becker et al. 2009) using a depth range of 200-1000m. Occurrences were mapped at 30 arc second spatial resolution.	Direct	Quantitative accuracy assessment	30 arc-second (ca. 1km2) or better
M2.3	Indicative distributions of these bathypelagic ocean waters were based on bathymetric spatial data (Becker et al. 2009) using a depth range of 1000-3000m. Occurrences were mapped at 30 arc second spatial resolution.	Direct	Quantitative accuracy assessment	30 arc-second (ca. 1km2) or better
M2.4	Indicative distributions of these epipelagic ocean waters were based on bathymetric spatial data (Becker et al. 2009) using a depth range of >3000m. Occurrences were mapped at 30 arc second spatial resolution.	Direct	Quantitative accuracy assessment	30 arc-second (ca. 1km2) or better

Code	Description	Concept alignment	Base data evaluation	Spatial resolution
M2.5	Indicative distributions of sea ice were obtained from Fetterer et al. (2017). To approximate the maximum annual global extent, we used the monthly extent for March 2019 for the northern hemisphere, and the monthly extent for September 2018 for the southern hemisphere. Occurrences were mapped at 30 arc second spatial resolution.	Direct	Quantitative accuracy assessment	larger than 1km2/30 arc-second
M3.1	Major occurrences of continental and island slopes was based on the 'slope' geomorphic unit of Harris et al. (2014b). Occurrences were converted to 30 arc second spatial resolution.	Direct	Quantitative accuracy assessment	variable (polygons)
M3.2	Major occurrences of submarine canyons was based on the 'canyons' geomorphic unit of Harris et al. (2014b). Occurrences were converted to 30 arc second spatial resolution.	Direct	Quantitative accuracy assessment	variable (polygons)
M3.3	Major occurrences of Abyssal plains was based on the 'plains' and 'hills' classes within the abyssal geomorphic unit of Harris et al. (2014b). Occurrences were converted to 30 arc second spatial resolution.	Direct	Quantitative accuracy assessment	variable (polygons)
M3.4	Major occurrences of seamounts, ridges and plateaus was based on the 'mountains' classes within the abyssal geomorphic unit of Harris et al. (2014b). Occurrences were converted to 30 arc second spatial resolution.	Direct	Quantitative accuracy assessment	variable (polygons)
M3.5	The distribution of deepwater biogenic beds was based on the 'mountains' and 'hills' classes within the abyssal geomorphic unit of Harris et al. (2014b). These were mapped as minor occurrences to acknowledge considerable uncertainties in the distribution of biogenic beds within these geomorphic units. Occurrences were converted to 30 arc second spatial resolution.	Indirect	Quantitative accuracy assessment	larger than 1km2/30 arc-second
M3.6	Major occurrences of Hadal trenches and troughs were based on the 'hadal' and 'trenches' geomorphic units of Harris et al. (2014b). Occurrences were converted to 30 arc second spatial resolution.	Direct	Quantitative accuracy assessment	variable (polygons)
M3.7	Major occurrences of Chemosynthetic-based ecosystems were based on the distribution of hydrothermal vents on spreading plate boundaries mapped in 'Plate lines and polygons' data by USGS/ESRI (undated). Occurrences were converted to 30 arc second spatial resolution. The distribution of cold seeps is poorly known and was not mapped.	Indirect	Undocumented	larger than 1km2/30 arc-second
M4.1	Marine ecoregions that include occurrences of submerged artificial structures were identified by overlaying a mapped distribution of shipwrecks (Monfils, 2004) on marine ecoregions (Spalding et al., 2008). Occurrences were converted to 30 arc second spatial resolution. In many cases these ecoregions encompassed other submerged structures such as energy infrastructure. To represent uncertainty, indicative distributions were mapped as minor occurrences.	Direct	Known records	larger than 1km2/30 arc-second

Code	Description	Concept alignment	Base data evaluation	Spatial resolution
M4.2	Marine ecoregions (Spalding et al., 2008) containing marine aquafarms were identified by consulting global and regional reviews, suitability maps (Gentry et al., 2017) and expertise of authors. These were clipped to the extent of the marine 'shelf' base layer as mapped by Harris et al. (2014b) and converted to 30 arc second spatial resolution.	Indirect	Qualitative expert assessment	larger than 1km2/30 arc-second
MT1.1	Marine ecoregions (Spalding et al., 2008) containing rocky shorelines were identified by consulting regional substrate maps, imagery available in Google Earth (to exclude ecoregions with extensive sandy or muddy shores) and expertise of authors. Occurrences were aggregated to 1 degree spatial resolution.	Indirect	Qualitative expert assessment	larger than 1km2/30 arc-second
MT1.2	Tidal flats were mapped directly from remote sensing time series and aggregated to 1 degree spatial resolution by Murray et al. (2019). Major occurrences were mapped in 1-degree cells with >200km2 mudflat extent, and minor occurrences were mapped in cells with 5-200km2 mudflat extent.	Direct	Quantitative accuracy assessment	30 arc-second (ca. 1km2) or better
MT1.3	The indicative map of Sandy shorelines was based on point records of sandy coastlines mapped by Vousdoukas et al. (2020) aggregated to 1 degree spatial resolution. Cells with >50 points were reclassified as major occurrences, and those with 1-50 points were reclassified as minor occurrences.	Direct	Quantitative accuracy assessment	30 arc-second (ca. 1km2) or better
MT1.4	Marine ecoregions (Spalding et al., 2008) containing boulder and cobble shorelines were identified by consulting regional substrate maps, imagery available in Google Earth (to exclude ecoregions with extensive sandy or muddy shores) and expertise of authors. Occurrences were aggregated to 1 degree spatial resolution.	Indirect	Qualitative expert assessment	larger than 1km2/30 arc-second
MT2.1	Coastlines were mapped between 60°S and 60°N with a 20 km buffer applied.	Indirect	Quantitative accuracy assessment	30 arc-second (ca. 1km2) or better
MT2.2	Spatial data on Nitrogen (N) and Phosphorus (P) deposition from seabird colonies (Otero et al. 2018) were used as indicators of the distribution of seabird and pinniped colonies. Original point data were in decimal degrees rounded to 6 arc-min resolution, which were aggregated data to square grid cells of 250 km. We used a threshold of >1000 and <100000 kg/yr N to identify minor occurrences and a threshold of >100000 kg/yr N for major occurrences.	Direct	Known records	larger than 1km2/30 arc-second

Code	Description	Concept alignment	Base data evaluation	Spatial resolution
MT3.1	Marine ecoregions (Spalding et al., 2008) containing major and minor occurrences of urbanised shorelines were identified from the map of night lights (Cinzano et al. 2019), imagery available on Google Earth and expertise of authors. Occurrences were aggregated to 1 degree spatial resolution and intersected with the coastline to exclude areas inland and in the open ocean.	Indirect	Quantitative accuracy assessment	variable (polygons)
MFT1.1	The extent of major coastal deltas was taken directly from Nienhuis et al. (2020). The data are based on polygons that encompass the lowest reaches of deltaic floodplains and a marine buffer approximating the extent of subtidal deltaic sediments. We checked the data for completeness against point locations shown in Fig. 1 of Goodbred & Saito (2012) and maps of Tessler et al. (2015) and found them to be inclusive of major occurrences. Tessler et al. (2015) included fewer deltas and polygons that extended some distance up freshwater floodplains into the Freshwater-Terrestrial (FT) transition biome and therefore was not used.	Direct	Qualitative expert assessment	30 arc-second (ca. 1km2) or better
MFT1.2	The indicative map for Intertidal forests and shrublands was was developed by resampling the known global distribution of mangrove forests for the year 2016 mapped by Global Mangrove Watch (Bunting et al. 2018). We used a buffer of 1km around the distribution data and a 30 arc second grid, thus large aggregations (> 1km2) are depicted as major occurrences, and the buffer areas with small occurrences are shown as minor occurrences.	Direct	Quantitative accuracy assessment	30 arc-second (ca. 1km2) or better
MFT1.3	The indicative map for Coastal saltmarshes was based on mapping by McOwen et al. (2017) summarised within a template of 1-degree grid cells. Cells with >5% cover of marsh vegetation were reclassified as major occurrences, and those with non-zero cover up to 5% were reclassified as minor occurrences.	Direct	Qualitative expert assessment	30 arc-second (ca. 1km2) or better

Use of references

Key references are listed in the descriptive profiles as sources of further information for each EFG. Preference has been given to recent global reviews and where these are not available, regional reviews or publications addressing characteristic ecological processes are provided for respective ecosystem groups. Older literature was cited where it addressed key features more directly than recent literature.

Updates

The Global Ecosystem Typology will be updated periodically as new information comes to light. Updates to version 1.0 incorporated in version 1.01 include:

- an expanded glossary of terms (see Supplementary Information)
- a full copy edit of descriptive profiles
- inclusion of a new Ecosystem Functional Group, F2.10 Subglacial Lakes

Version 2.0 is the outcome of further major review and revision of the typology by 48 additional ecosystem specialists in 2020. Updates to version 1.01 incorporated in version 2.0 include:

- addition of five new Ecosystem Functional Groups to Level 3 of the typology in response to reviewers' recommendations (one freshwater group F1.7, one anthropogenic terrestrial group T7.5, two subterranean freshwater groups SM1.2 and SM1.3; and one artificial subterranean-freshwater group SF2.2)
- major revisions to four existing profiles for freshwater EFGs (F1.2, F1.4, F1.5, F3.2)
- amendments to diagrammatic models for 28 EFGs in response to recommendations from specialist reviewers,
- thematic adjustments to distribution maps for 12 EFGs
- addition or replacement of references in 12 EFGs.
- minor edits to text in profiles for all EFGs to improve clarity and detail
- substantial expansion of the glossary (see Supplemenatry Information)
- comprehensive upgrade of broad-scale indicative maps to higher resolution maps based directly on remote sensing, or point locations, or indirectly on environmental proxies.

Version 2.01 was the outcome of additional reviews completed in 2021 and revision. Updates to version 2.0 incorporated in version 2.01 include:

- minor adjustment to names of four EFGs to improve clarity and distinction from related EFGs (T4.5, T7.1, T7.2, T7.4)
- minor revisions to text for eight EFGs (T1.1, T1.2, T1.3, T1.4, T7.1, T7.2, T7.5, M1.5)
- updates to maps for eight EFGs (T7.2, T7.4, T7.5, F3.1, F3.2, F3.5, MFT1.1, MFT1.2)
- refinements to the format of diagrammatic models for all EFGs (changing labels from 'Ecological traits' to 'Ecosystem Properties', adding feedbacks to biotic interactions)
- further additions to the glossary (see Supplemenatry Information)

Version 2.1 expanded the set set of Level 3 units, updated maps and incorporated text revisions from additional reviews completed in early 2022. Updates to version 2.01 incorporated in version 2.1 include:

- two additional EFGs in level 3, M1.10 Rhodolith/Maërl beds and MT2.2 Large seabird and pinniped colonies
- updates to maps for six EFGs (T1.3, T4.3, T5.3, TF1.2, TF1.5, F1.6)
- Minor revisions to text for four EFGs.

An interactive interface to the IUCN Global Ecosystem Typology, its hierarchical structure, descriptive profiles and maps is available at https://global-ecosystems.org/. Future updates will also be available at that site.

References cited

Abell, R., Thieme, M.L., Revenga, C., Bryer, M., Kottelat, M., Bogutskaya, N., Coad, B., Mandrak, N., Contreras Balderas, S., Bussing, W., Stiassny, M.L.J., Skelton, P., Allen, G.R., Unmack, P., Naseka, A., Ng, R., Sindorf, N., Robertson, J., Armijo, E., Higgins, J.V., Heibel, T.J., Wikramanayake, E., Olson, D., López, H.L., Reis, R.E., Lundberg, J.G., Sabaj Pérez, M.H. and Petry, P. (2008). 'Freshwater ecoregions of the world: A new map of biogeographic units for freshwater biodiversity conservation'. *BioScience* 58: 403–414. https://doi.org/10.1641/B580507

Armston, J., Scarth, P., Lucas, R., Lewis, P., Disney, M. and Phinn, S. (2015). 'Validation of continental scale vertical plant profile mapping using waveform lidar airborne laser scanning'. Paper delivered at Silvilaser 2015, La Grande Motte, France, 28-30 September.

Beck, M.W., Brumbaugh, R.D., Airoldi, L., Carranza, A., Coen, L.D., Crawford, C., Defeo, O., Edgar, G.J., Handcock, B., Kay, M.C., Lenihan, H.S., Luckenbach, M., Toropova, C.L., Zhang, G. and Guo X. (2011). 'Oyster reefs at risk and recommendations for conservation, restoration, and management'. *BioScience* 61: 107–116. https://doi.org/10.1525/bio.2011.61.2.5

Beck, H., Zimmermann, N., McVicar, T., Vergopolan N., Berg, A. and Wood, E.F. (2018). 'Present and future Köppen-Geiger climate classification maps at 1-km resolution'. *Scientific Data* 5: 180214. https://doi.org/10.1038/sdata.2018.214

Becker, J.J., Sandwell, D.T., Smith, W.H.F., Braud, J., Binder, B., Depner, J., Fabre, D., Factor, J., Ingalls, S., Kim, S-H., Ladner, R., Marks, K., Nelson, S., Pharaoh, A., Trimmer, R., Von Rosenberg, J., Wallace, G. and Weatherall, P. (2009). 'Global bathymetry and elevation data at 30 arc seconds resolution: SRTM30_PLUS'. *Marine Geodesy* 32: 355–371. https://doi.org/10.1080/01490410903297766

Beckmann M, Václavík T, Manceur AM, Šprtová L, von Wehrden H, Welk E, Cord AF (2014) glUV: A global UV-B radiation dataset for macroecological studies, Methods in Ecology and Evolution 5: 372–383. doi: 10.1111/2041-210X.12168

Bowling, J.S., Livingstone, S.J., Sole, A.J. and Chu, W. (2019). 'Distribution and dynamics of Greenland subglacial Lakes'. *Nature Communications* 10: 2810. https://doi.org/10.1038/s41467-019-10821-w

Bundesanstalt für Geowissenschaften und Rohstoffe (BGR) & UNESCO (2012). *River and groundwater basins of the world 1: 50 000 000*. Paris: Hannover

Bunting P., Rosenqvist A., Lucas R., Rebelo L-M., Hilarides L., Thomas N., Hardy A., Itoh T., Shimada M. and Finlayson C.M. (2018). The Global Mangrove Watch – a New 2010 Global Baseline of Mangrove Extent. *Remote Sensing* 10: 1669. doi: 10.3390/rs1010669

CEC Commission for Environmental Cooperation (1997). *Ecological regions of North America - Toward a common perspective*. Montréal, Canada: Commission for Environmental Cooperation.

Cinzano, P., Falchi, F. and Elvidge, C.D. (2019). 'The world atlas of the artificial night sky brightness'. [Online Dataset]. Copyright Royal Astronomical Society, Reproduced from the Monthly Notices of the RAS by permission of Blackwell Science. Boulder, Colorado: University of Padova & NOAA National Geophysical Data Center. Available at http://www.inquinamentoluminoso.it/worldatlas/pages/fig1.htm (accessed 11 November 2019).

Cramer, W., Kicklighter, D.W., Bondeau, A., Moore Iii, B., Churkina, G., Nemry, B., Ruimy, A., Schloss, A.L. and Participants of the Potsdam NPP Model Intercomparison (1999). 'Comparing global models of terrestrial net primary productivity (NPP): overview and key results'. *Global Change Biology* 5: 1–15. https://doi.org/10.1046/j.1365-2486.1999.00009.x

Cury P, Shannon L, Shin YJ (2003) The functioning of marine ecosystems: a fisheries perspective. *Responsible fisheries in the marine ecosystem* (Eds. M Sinclair, G Valdimarsson), p103–123. FAO, Rome, & CABI Publishing, Wallingford.

DellaSala, D.A., Alaback, P., Spribille, T., von Wehrden, H., Nauman, R.S. (2011). Just what are temperate and boreal rainforests?. Temperate and boreal rainforests of the world: ecology and conservation (Ed. D.A. DellaSala), pp1-41. Island Press, Washington DC

Dinerstein, E., Olson, D., Joshi, A., Vynne, C., Burgess, N.D., Wikramanayake, E., Hahn, N., Palminteri, S., Hedao, P., Noss, R., Hansen, M., Locke, H., Ellis, E.E., Jones, B., Barber, C.V., Hayes, R., Kormos, C., Martin, V., Crist, E., Sechrest, W., Price, L., Baillie, J.E.M., Weeden, D., Suckling, K., Davis, C., Sizer, N., Moore, R., Thau, D., Birch, T., Potapov, P., Turubanova, S., Tyukavina, A., de Souza, N., Pintea, L., Brito, J.C., Llewellyn Barnekow Lillesø, J.P., van Breugel, P., Graudal, L., Voge, M., Al-Shammari, K.F., and Saleem, M. (2017). 'An ecoregion-based approach to protecting half the terrestrial realm'. *BioScience* 67: 534–545. https://doi.org/10.1093/biosci/bix014

Dürr, H.H., Laruelle, G.G., van Kempen, C.M., Slomp, C.P., Meybeck, M. and Middelkoop, H. (2011). 'Worldwide typology of nearshore coastal systems: Defining the estuarine filter of river inputs to the oceans'. *Estuaries and Coasts* 34: 441-458. https://doi.org/10.1007/s12237-011-9381-y

Fetterer, F., Knowles, K., Meier, W.N., Savoie, M. and Windnagel, A.K. (2017). 'Sea ice index, version 3, Monthly sea ice extent'. [Online Dataset]. Boulder, Colorado: National Snow and Ice Data Center (NSIDC). Available at https://doi.org/10.7265/N5K072F8 (accessed 11 November 2019).

Gentry, R.R., Froehlich, H.E., Grimm, D., Kareiva, P., Parke, M., Rust, M., Gaines, S.D. and Halpern, B.S. (2017). 'Mapping the global potential for marine aquaculture'. *Nature Ecology & Evolution* 1: 1317–1324. https://doi.org/10.1038/s41559-017-0257-9

GeoNames (2020). 'The GeoNames geographical database'. [Online Database]. Available at https://www.geonames.org (Accessed Feb 2020).

Gilbert, M., Nicolas, G., Cinardi, G., Van Boeckel, T.P., Vanwambeke, S.O., William Wint, G.R. and Robinson, T.P. (2018). 'Global distribution data for cattle, buffaloes, horses, sheep, goats, pigs, chickens and ducks in 2010'. *Scientific Data* 5: 180227. https://doi.org/10.1038/sdata.2018.227

GLIMS and NSIDC (2005-2018). 'Global Land Ice Measurements from Space glacier database'. [Online Database]. Boulder, Colorado: Compiled and made available by the international GLIMS community and the National Snow and Ice Data Center (NSIDC). Available at http://dx.doi.org/10.7265/N5V98602

Goodbred, S.L. and Saito, Y. (2012). 'Tide-dominated deltas'. In: R.A. Davis and R.W. Dalrymple (Eds.) *Principles of tidal sedimentology*, pp. 129–149. Berlin: Springer. https://doi.org/10.1007/978-94-007-0123-6_7

Green, E.P., Short, F.T. (2003). 'World atlas of seagrasses'. UNEP World Conservation Monitoring Centre. Berkeley: University of California Press. Available at https://gis.unep-wcmc.org/arcgis/rest/services/marine/WCMC_013_014_Seagrass_WMS/MapServer (Accessed 11 November 2019)

Haberl, H., K.-H. Erb, F. Krausmann, V. Gaube, A. Bondeau, C. Plutzar, S. Gingrich, W. Lucht and M. Fischer-Kowalski, (2007). Quantifying and mapping the global human appropriation of net primary production in Earth's terrestrial ecosystems. Proceedings of the National Acadademy of Sciences of the United States of America 104, 12942-12947.

Harris, I., Jones, P.D., Osborn, T.J. and Lister, D.H. (2014a). 'Updated high-resolution grids of monthly climatic observations - the CRU TS3.10 Dataset'. *International Journal of Climatology* 34: 623-642. [Revised appendix] (https://crudata.uea.ac.uk/cru/data/hrg/Revised_Appendix_3_CLD.pdf)

Harris, P.T., Macmillan-Lawler, M., Rupp, J. and Baker, E.K. (2014b). 'Geomorphology of the oceans'. *Marine Geology* 352: 4–24. https://doi.org/10.1002/joc.3711

Hui, F., Kang, J., Liu, Y., Cheng, X., Gong, P., Wang, F., Li, Z., Ye, Y. and Guo, Z. (2017). 'AntarcticaLC2000: The new Antarctic land cover database for the year 2000'. *SCIENCE CHINA Earth Sciences* 60: 686-696. https://doi.org/10.1007/s11430-016-0029-2

Huston, M.A. and Wolverton, S. (2009). 'The global distribution of net primary production: resolving the paradox'. *Ecological Monographs* 79: 343–377. https://doi.org/10.1890/08-0588.1

Hutchings L, Pitcher GC, Probyn TA, Bailey GW (1995) The chemical and biological consequences of coastal upwelling. *Upwelling in the oceans: Modern Processes and Ancient Records* (Eds. CP Summerhayes, KC Emeis, MV Angel, RL Smith, B Zeitzschel), pp. 65–81. John Wiley, New York.

Institute for Marine Remote Sensing, University of South Florida (IMaRS/USF), Institut de Recherche pour le Développement (IRD), UNEP-WCMC, The WorldFish Center, and WRI (2011). 'Global Coral Reefs composite dataset compiled from multiple sources for use in the Reefs at Risk Revisited project incorporating products from the Millennium Coral Reef Mapping Project prepared by IMaRS/USF and IRD'. [Online Dataset]. Available at http://datasets.wri.org/dataset/tropical-coral-reefs-of-the-world-500-m-resolution-grid (accessed 11 November 2019)

IUCN (2012) Habitats classification scheme v3.1. http://www.iucnredlist.org/technical-documents/classification-schemes/habitats-classification-scheme-ver3

Jones, C.G., Lawton, J.H. and Shachak, M. (1994). 'Organisms as ecosystem engineers'. *Oikos* 69: 373-386. https://doi.org/10.2307/3545850

Jung M, Raj Dahal P, Butchart SHM, Donald PF, De Lamo X, Lesiv M, Kapos V, Rondinini C, Visconti P (2020) A global map of terrestrial habitat types. Scientific Data 7:256. [https://doi.org/10.1038/s41597-020-00599-8]

Karger, D.N., Conrad, O., Böhner, J., Kawohl, T., Kreft, H., Soria-Auza, R.W., Zimmermann, N.E., Linder, H.P. and Kessler, M. (2017). 'Climatologies at high resolution for the earth's land surface areas'. *Scientific Data* 4(1): 170122. https://doi.org/10.1038/sdata.2017.122

Karger, D.N., Kessler, M., Lehnert, M. and Jetz W (2021) Limited protection and ongoing loss of tropical cloud forest biodiversity and ecosystems worldwide. Nature Ecology & Evolution 5, 854–862. https://doi.org/10.1038/s41559-021-01450-y

Karra, K., Kontgis, C., Statman-Weil, Z., Mazzariello, J., Mathis, M. and Brumby, S. (2021). Global land use/land cover with Sentinel-2 and deep learning. IEEE International Geoscience and Remote Sensing Symposium. Institute of Electrical and Electronics Engineers, Brussels. Map available at: https://www.arcgis.com/home/item.html?id=d6642f8a4f6d4685a24ae2dc0c73d4ac

Keith, D.A., Ferrer-Paris, J.R., Nicholson, E., Bishop, M.J., Polidoro, B.A., Ramirez-Llodra, E., Tozer, M.G., Nel, J.L., Mac Nally, R., Gregr, E.J., Watermeyer, K.E., Essl, F., Faber-Langendoen, D., Giller, P.S., Robson, B., Franklin, J., Lehmann, C.E.R., Etter, A., Roux, D.J., Stark, J.S., Rowland, J.A., Brummitt, N.A., Fernandez-Arcaya, U.C., Suthers, I.M., Iliffe, T.M., Gerovasileiou, V., Sakihara, T.S., Wiser, S.K, Donohue, I., Jackson, L.J., Pennington, R.T., Linardich, C., Pettorelli, N., Andrade, A., Kontula, T., Lindgaard, A., Tahvanainan, T., Terauds, A., Venter, O., Watson, J.E.M., Chadwick, M.A., Murray, N.J., Moat, J., Pliscoff, P., Corlett, R.T., Young, K.R., McGlone, M.S., Williams, R., Loidi, J., Russell-Smith, J., Gibson, D., Eldridge, D.J., Anesio, A.M.B., Körner, C.H., Harper, R., Bogaart, P.W., Bhanumati, P., Sharma, M., Hose, G.C., Gonzalez, B.C., Brankovits, D., Martínez García, A., Lamson, M., Seidel, B., Sedar, D.M., Santos, S., Havird, J., Catford, J.A., Rains, M.C., Irvine, K., Arthington, A.H., Kelly-Quinn, M., Bertilsson, S., Hollibaugh, J.T., Channing, A., Siegert, M.J., Reidy Liermann, C., Beveridge, M., Bianchi, T.S., Woodland, R.J., Dafforn, K.A., McSweeney, S.L., Cutler, N.A., Orth, R.J., Altieri, A.H., Rossi, S., Sheppard, C.R.C., Swearer. S.E., Rykaczewski, R.R., Shannon, L.J., Priede, I.G., Sutton, T.T., Claisse, J.T., Acosta, A.T.R., Carnell, P.E, Crowe, T.P., Firth, L.B., Hay, S.E., García, L., Zager, I., Bland, L.M., Kingsford, R.T. (2020) *The IUCN Global Ecosystem Typology v2.0: Descriptive profiles for Biomes and Ecosystem Functional Groups.* IUCN, Gland, Switzerland.

Keith, D. A. and Tozer, M. G. (2017). Girt: a continental synthesis of Australian vegetation. In: Australian vegetation. D. A. Keith. Editor, pp3-39. Cambridge University Press, Cambridge

Kicklighter, D.W., Bondeau, A., Schloss, A.L., Kaduk, J., McGuire, A.D. and the Participants of the Potsdam NPP Model Intercomparison (1999). 'Comparing global models of terrestrial net primary productivity (NPP): global pattern and differentiation by major biomes'. *Global Change Biology* 5: 16–24. https://doi.org/10.1046/j.1365-2486.1999.00003.x

Latifovic R, Homer C, Ressl R, Pouliot D, Hossain SN, Colditz RR, Olthof I, Giri C, Victoria A (2016) North American land change monitoring system. In: Giri C (Ed), Remote Sensing of Land Use and Land Cover: Principles and Applications. CRC-Press, pp. 303-324. DOI: 10.1201/b11964

Lehner, B. and Döll, P. (2004). 'Development and validation of a global database of lakes, reservoirs and wetlands'. *Journal of Hydrology* 296: 1–22. https://doi.org/10.1016/j.jhydrol.2004.03.028

Livingstone, S.J., Utting, D.J., Ruffell, A., Clark, C.D., Pawley, S., Atkinson, N. and Fowler, A.C. (2016). 'Discovery of relict subglacial lakes and their geometry and mechanism of drainage'. *Nature Communications* 10: 2810. https://doi.org/10.1038/ncomms11767

Linke, S., Lehner, B., Ouellet Dallaire, C., Ariwi, J., Grill, G., Anand, M., Beames, P., Burchard-Levine, V., Maxwell, S., Moidu, H., Tan, F. and Thieme, M. (2019). 'Global hydro-environmental sub-basin and river reach characteristics at high spatial resolution'. *Scientific Data* 6: 283. https://doi.org/10.1038/s41597-019-0300-6

Livingstone, S., Utting, D., Ruffell, A., Clark, C.D., Pawley, S., Atkinson, N. and Fowler, A.C. (2016). 'Discovery of relict subglacial lakes and their geometry and mechanism of drainage'. *Nature Communications* 7: 11767. https://doi.org/10.1038/ncomms11767

Loidi, J., Biurrun, I., Juan Antonio Campos, J.A., García-Mijangos, I. and Herrera, M. (2010). 'A biogeographical analysis of the European Atlantic lowland heathlands'. *Journal of Vegetation Science* 21: 832–842. https://doi.org/10.1111/j.1654-1103.2010.01204.x

Loveland, T.R., Reed, B.C., Brown, J.F., Ohlen, D.O., Zhu, J., Yang, L. and Merchant, J.W. (2000). 'Development of a Global Land Cover Characteristics Database and IGBP DISCover from 1-km AVHRR Data'. *International Journal of Remote Sensing* 21: 1303-1330. https://doi.org/10.1111/j.1654-1103.2010.01204.x

Luebert, F. and Pliscoff, P. (2017). *Sinopsis bioclimatíca y vegetactional de Chile*. Segundo edicíon. Santiago: Editorial Universitaria.

McOwen, C.J., Weatherdon, L.V., van Bochove, J.W., Sullivan, E., Blyth, S., Zockler, C., Stanwell-Smith, D., Kingston, N., Martin, C.S., Spalding, M. and Fletcher, S. (2017). 'A global map of saltmarshes'. *Biodiversity Data Journal* 5: e11764. https://doi.org/10.3897/BDJ.5.e11764

McSweeney, S.L., Kennedy, D.M., Rutherfurd, I.D. and Stout, J.C. (2017). Intermittently Closed/Open Lakes and Lagoons: Their global distribution and boundary conditions. *Geomorphology* 292:142-52.

Messager, M.L., Lehner, B., Grill, G., Nedeva, I. and Schmitt, O. (2016). 'Estimating the volume and age of water stored in global lakes using a geo-statistical approach'. *Nature Communications* 7: 13603. https://doi.org/10.1038/ncomms13603

Monfils, R. (2004). 'The global risk of marine pollution from WWII shipwrecks: Examples from the Seven Seas'. Canberra, Australia: Sea Australia. Available at http://www.seaaustralia.com/documents/The%20Global%20Risk%20of%20Marine%20Pollution%20from%20WWII%20Shipwrecks-final.pdf (Accessed 11 November 2019)

Monfreda, C.N., Ramankutty, N. and Foley, J.A. (2008). 'Farming the planet: 2. Geographic distribution of crop areas, yields, physiological types, and net primary production in the year 2000'. *Global Biogeochemical Cycles* 22: GB1022. https://doi.org/10.1029/2007GB002947

Mucina, L. (2019). 'Biome: evolution of a crucial ecological and biogeographical concept'. *New Phytologist* 222: 97–114. https://doi.org/10.1111/nph.15609

Mulligan, M., van Soesbergen, A. and Sáenz, L. (2020). GOODD, a global dataset of more than 38,000 georeferenced dams. *Scientific Data* 7: 31. https://doi.org/10.1038/s41597-020-0362-5

Murray, N.J., Phinn, S.R., DeWitt, M., Ferrari, R., Johnston, R., Lyons, M.B., Clinton, N., Thau, D. and Fuller, R.A. (2019). 'The global distribution and trajectory of tidal flats'. *Nature* 565: 222–225. https://doi.org/10.1038/s41586-018-0805-8

Nagaraj, D., Proust, E., Todeschini, A., Rulli, M.C., D'Odorico, P. (2021). A new dataset of global irrigation areas from 2001 to 2015, *Advances in Water Resources* 152: 103910. https://doi.org/10.1016/j.advwatres.2021.103910.

Nienhuis, J.H., Ashton, A.D., Edmonds, D.A. et al. (2020). Global-scale human impact on delta morphology has led to net land area gain. *Nature* 577: 514–518. https://doi.org/10.1038/s41586-019-1905-9

NOAA / National Centers for Environmental Information (NOAA/NCEI) (2019). 'VIIRS Day/Night Band Nighttime Lights'. [Online Dataset]. Earth Observation Group, NOAA National Centers for Environmental Information (NCEI) (Version 1). Available at: https://eogdata.mines.edu/download_dnb_composites.html

NVIS (2016) National Vegetation Information System. Version 4.2. Australian Government, Canberra. https://www.awe.gov.au/agriculture-land/land/native-vegetation/national-vegetation-information-system/data-products [Downloaded December 2017].

Olson, D.M., Dinerstein, E., Wikramanayake, E.D., Burgess, N.D., Powell, G.V., Underwood, E.C., D'amico, J.A., Itoua, I., Strand, H.E., Morrison, J.C. and Loucks, C.J. (2001). 'Terrestrial ecoregions of the world: A new map of life on earth'. *BioScience* 51: 933–938. https://doi.org/10.1641/0006-3568(2001)051[0933:TEOTWA]2.0.CO;2

Ouellet Dallaire, C., Lehner, B., Sayre, R. and Thieme, M. (2018). 'A multidisciplinary framework to derive global river reach classifications at high spatial resolution'. *Environmental Research Letters* 14: 024003 https://doi.org/10.1088/1748-9326/aad8e9

Pekel, J.F., Cottam, A., Gorelick, N., Belward, A.S. (2016). 'High-resolution mapping of global surface water and its long-term changes'. *Nature* 540: 418-422. https://doi.org/10.1038/nature20584

Pettorelli N., Schulte to Buehne H., Tulloch A., Dubois G., Macinnis-Ng C., Queiros A. M., Keith D. A., Wegmann M., Schrodt F., Stellmes M., Sonnenschein R., Geller G. N., Roy S., Somers B., Murray N., Bland L., Geijzendorffer I., Kerr J. T., Broszeit S., Leitao P. J., Duncan C., El Serafy G., He K. S., Blanchard J. L., Lucas R., Mairota P., Webb T. J. and Nicholson E. (2018) Satellite remote sensing of ecosystem functions: opportunities, challenges and way forward, *Remote Semnsing in Ecology and Conservation* 4: 71-93. https://doi.org/10.1002/rse2.59

Ramankutty, N., Evan, A.T., Monfreda, C., Foley, J.A. (2008), Farming the planet: 1. Geographic distribution of global agricultural lands in the year 2000. Global Biogeochemical Cycles 22, GB1003, doi:10.1029/2007GB002952.

Raunkiaer, C. (1934). 'The use of leaf size in biological plant geography'. In: A.G.T.H. Gilbert-Carter, A. Fausbøll (Eds.) *The life forms of plants and statistical biogeography* pp. 368–378. Clarendon: Oxford.

Raup, B.H., Racoviteanu, A., Khalsa, S.J.S., Helm, C., Armstrong, R. and Arnaud, Y. (2007) 'The GLIMS Geospatial Glacier Database: a New Tool for Studying Glacier Change'. *Global and Planetary Change* 56: 101-110. https://doi.org/10.1016/j.gloplacha.2006.07.018

Raynolds, M.K., Walker, D.A., Balser, A., Bay, C., Campbell, M., Cherosov, M.M., Daniëls, F.J.A., Bronken Eidesen, P., Ermokhina, K.A., Frost, G.V., Jedrzejek, B., Jorgenson, M.T., Kennedy, B.E., Kholod, S.S., Lavrinenko, I.A., Lavrinenko, O.V., Magnússon, B., Matveyeva, N.V., Metúsalemsson, S., Nilsen, L., Olthof, I., Pospelov, I.N., Pospelova, E.B., Pouliot, D., Razzhivin, V., Schaepman-Strub, G., Šibík, J., Telyatnikov, M.Y., Troeva, E. (2019). 'A raster version of the Circumpolar Arctic Vegetation Map (CAVM)'. *Remote Sensing of Environment* 232: 111297. https://doi.org/10.1016/j.rse.2019.111297

Sarmiento, J.L., Gruber, N., Brzezinski, M.A. and Dunne, J.P. (2004). 'High-latitude controls of thermocline nutrients and low latitude biological productivity'. *Nature* 427: 56–60. https://doi.org/10.1038/nature02127

SCAR (1992-2020). 'Composite Gazetteer of Antarctica Scientific Committee on Antarctic Research' [GCMD Metadata]. Available at http://gcmd.nasa.gov/records/SCAR_Gazetteer.html

Senterre B, Lowry II PP, Bidault E, Stévart T (2021) Ecosystemology: A new approach toward a taxonomy of ecosystems. Ecological Complexity 47: 100945. https://doi.org/10.1016/j.ecocom.2021.100945

Siebert, S., Döll, P., Hoogeveen, J., Faures, J.-M., Frenken, K. and Feick, S. (2005). 'Development and validation of the global map of irrigation areas'. *Hydrology and Earth System Sciences* 9: 535-547. https://doi.org/10.5194/hess-9-535-2005

Siebert, S., Henrich, V., Frenken, K. and Burke, J. (2013). 'Update of the Global Map of Irrigation Areas to version 5 Project report'. DOI: 10.13140/2.1.2660.6728

Spalding, M.D., Fox, H.E., Allen, G.R., Davidson, N., Ferdaña, Z.A., Finlayson, M.A., Halpern, B.S., Jorge, M.A., Lombana, A.L., Lourie, S.A. and Martin, K.D. (2007). 'Marine ecoregions of the world: a bioregionalization of coastal and shelf areas'. *BioScience* 57: 573–583. https://doi.org/10.1641/B570707

Tang, H., Armston, J., Hancock, S., Marselis, S., Goetz, S. and Dubayah, R. (2019). 'Characterizing global forest canopy cover distribution using spaceborne lidar'. *Remote Sensing of Environment* 231: 111262. https://doi.org/10.1016/j.rse.2019.111262

Tessler, Z.D., Vörösmarty, C.J., Grossberg, M., Gladkova, I., Aizenman, H., Syvitski, J.P.M. and Foufoula-Georgiou, E. (2015) Profiling Risk and Sustainability in Coastal Deltas of the World. *Science* 349(6248): 638-643. DOI: 10.1126/science.aab3574

Thór Marteinsson, V., Rúnarsson, Á., Stefánsson, A., Thorsteinsson, T., Jóhannesson, T., Magnússon, S.H., Reynisson, E., Einarsson, B., Wade, N., Morrison, H.G. and Gaidos, E. (2013). 'Microbial communities in the subglacial waters of the Vatnajökull ice cap, Iceland'. ISME Journal 7: 427–437. https://doi.org/10.1038/ismej.2012.97

Tuanmu, M-N. and Jetz, W. (2014). 'A global 1-km consensus land-cover product for biodiversity and ecosystem modelling'. *Global Ecology and Biogeography* 23: 1031—1045. https://doi.org/10.1111/geb.12182

UNEP-WCMC and Short, F.T. (2017). 'Global distribution of seagrasses (version 6.0)'. Sixth update to the data layer used in Green and Short (2003). Cambridge, UK: UN Environment World Conservation Monitoring Centre. Available at http://data.unep-wcmc.org/datasets/7

USGS/ESRI (undated) Plate lines and polygons [Dataset]. Available at https://www.arcgis.com/home/item.html?id=f155b76c13c84f62864446847f1ae652. Accessed 5/5/2019.

Violle C, Reich PB, Pacalae SW, Enquistf BJ, Kattge J (2014) The emergence and promise of functional biogeography. *Proceedings of the National Academy of Sciences of the United States of America* 111: 13690-13696. https://doi.org/10.1073/pnas.31415442111

Vousdoukas, M., Ranasinghe, R., Mentaschi, L., Plomaritis, T., Athanasiou, P., Luijendijk, A. and Feyen, L. (2020) 'Sandy coastlines under threat of erosion'. *Nature Climate Change* 10: 260–263. https://doi.org/10.1038/s41558-020-0697-0

Wernberg, T., Filbee-Dexter, K. (2019). 'Missing the marine forest for the trees'. *Marine Ecology Progress Series* 612: 209-215. https://doi.org/10.3354/meps12867

Williams, P. and Ting Fong, Y. (2016). 'World map of carbonate rock outcrops v3.0'. [Online Resource]. Auckland: The University of Auckland. Available at https://www.fos.auckland.ac.nz/our_research/karst/ (Accessed 11 November 2019)

Wilson, A.M. and Jetz, W. (2016). 'Remotely Sensed High-Resolution Global Cloud Dynamics for Predicting Ecosystem and Biodiversity Distributions'. *PLoS Biology* 14(3): e1002415. [Dataset available on-line]. Available at http://www.earthenv.org/) https://doi.org/10.1371/journal.pbio.1002415

Wright, A. and Siegert, M. (2012). 'A fourth inventory of Antarctic subglacial lakes'. *Antarctic Science* 24: 659–664. https://doi.org/10.1017/S095410201200048X

Wurtsbaugh, W.A., Miller, C., Null, S.E., DeRose, R.J., Wilcock, P., Hahnenberger, P., Howe, F. and Moore, J. (2017). 'Decline of the world's saline lakes'. *Nature Geoscience* 10: 816–821. https://doi.org/10.1038/ngeo3052

List of Ecosystem Functional Groups by realms and biomes

Realm	Biome	Ecosystem Functional Group (EFG)
Terrestrial	T1 Tropical-subtropical forests	T1.1Tropical-subtropical lowland rainforests
Terrestrial	T1 Tropical-subtropical forests	T1.2 Tropical-subtropical dry forests and thickets
Terrestrial	T1 Tropical-subtropical forests	T1.3 Tropical-subtropical montane rainforests
Terrestrial	T1 Tropical-subtropical forests	T1.4 Tropical heath forests
Terrestrial	T2 Temperate-boreal forests & woodlands	T2.1 Boreal and temperate montane forests and woodlands
Terrestrial	T2 Temperate-boreal forests & woodlands	T2.2 Deciduous temperate forests
Terrestrial	T2 Temperate-boreal forests & woodlands	T2.3 Oceanic cool temperate rainforests
Terrestrial	T2 Temperate-boreal forests & woodlands	T2.4 Warm temperate laurophyll forests
Terrestrial	T2 Temperate-boreal forests & woodlands	T2.5 Temperate pyric humid forests
Terrestrial	T2 Temperate-boreal forests & woodlands	T2.6 Temperate pyric sclerophyll forests and woodlands
Terrestrial	T3 Shrublands & shrubby woodlands	T3.1 Seasonally dry tropical shrublands
Terrestrial	T3 Shrublands & shrubby woodlands	T3.2 Seasonally dry temperate heaths and shrublands
Terrestrial	T3 Shrublands & shrubby woodlands	T3.3 Cool temperate heathlands
Terrestrial	T3 Shrublands & shrubby woodlands	T3.4 Rocky pavements, screes and lava flows
Terrestrial	T4 Savannas and grasslands	T4.1 Trophic savannas
Terrestrial	T4 Savannas and grasslands	T4.2 Pyric tussock savannas
Terrestrial	T4 Savannas and grasslands	T4.3 Hummock savannas
Terrestrial	T4 Savannas and grasslands	T4.4 Temperate woodlands
Terrestrial	T4 Savannas and grasslands	T4.5 Temperate tussock grasslands
Terrestrial	T5 Deserts and semi-deserts	T5.1 Semi-desert steppes
Terrestrial	T5 Deserts and semi-deserts	T5.2 Thorny deserts and semi-deserts
Terrestrial	T5 Deserts and semi-deserts	T5.3 Sclerophyll hot deserts and semi-deserts
Terrestrial	T5 Deserts and semi-deserts	T5.4 Cool deserts and semi-deserts
Terrestrial	T5 Deserts and semi-deserts	T5.5 Hyper-arid deserts
Terrestrial	T6 Polar-alpine	T6.1 Ice sheets, glaciers and perennial snowfields

Realm	Biome	Ecosystem Functional Group (EFG)
Terrestrial	T6 Polar-alpine	T6.2 Polar-alpine rocky outcrops
Terrestrial	T6 Polar-alpine	T6.3 Polar tundra and deserts
Terrestrial	T6 Polar-alpine	T6.4 Temperate alpine grasslands and shrublands
Terrestrial	T6 Polar-alpine	T6.5 Tropical alpine grasslands and shrublands
Terrestrial	T7 Intensive land-use systems	T7.1 Croplands
Terrestrial	T7 Intensive land-use systems	T7.2 Intensive livestock pastures
Terrestrial	T7 Intensive land-use systems	T7.3 Plantations
Terrestrial	T7 Intensive land-use systems	T7.4 Cities, villages and infrastructure
Terrestrial	T7 Intensive land-use systems	T7.5 Derived semi-natural pastures and oldfields
Subterranean	S1 Subterranean lithic systems	S1.1 Aerobic caves
Subterranean	S1 Subterranean lithic systems	S1.2 Endolithic systems
Subterranean	S2 Anthropogenic subterranean voids	S2.1 Anthropogenic subterranean voids
Subterranean-Freshwater	SF1 Subterranean freshwaters	SF1.1 Underground streams and pools
Subterranean-Freshwater	SF1 Subterranean freshwaters	SF1.2 Groundwater ecosystems
Subterranean-Freshwater	SF2 Anthropogenic subterranean freshwaters	SF2.1 Water pipes and subterranean canals
Subterranean-Freshwater	SF2 Anthropogenic subterranean freshwaters	SF2.2 Flooded mines and other voids
Subterranean-Marine	SM1 Tidal subterranean systems	SM3.1 Anchialine caves
Subterranean-Marine	SM1 Tidal subterranean systems	SM3.2 Anchialine pools
Subterranean-Marine	SM1 Tidal subterranean systems	SM3.1 Sea caves
Freshwater-Terrestrial	TF1 Palustrine wetlands	TF1.1 Tropical flooded forests and peat forests
Freshwater-Terrestrial	TF1 Palustrine wetlands	TF1.2 Subtropical/temperate forested wetlands
Freshwater-Terrestrial	TF1 Palustrine wetlands	TF1.3 Permanent marshes
Freshwater-Terrestrial	TF1 Palustrine wetlands	TF1.4 Seasonal floodplain marshes
Freshwater-Terrestrial	TF1 Palustrine wetlands	TF1.5 Episodic arid floodplains
Freshwater-Terrestrial	TF1 Palustrine wetlands	TF1.6 Boreal, temperate and montane peat bogs
Freshwater-Terrestrial	TF1 Palustrine wetlands	TF1.7 Boreal and temperate fens

Realm	Biome	Ecosystem Functional Group (EFG)
Freshwater	F1 Rivers and streams	F 1.1 Permanent upland streams
Freshwater	F1 Rivers and streams	F 1.2 Permanent lowland rivers
Freshwater	F1 Rivers and streams	F1.3 Freeze-thaw rivers and streams
Freshwater	F1 Rivers and streams	F 1.4 Seasonal upland streams
Freshwater	F1 Rivers and streams	F 1.5 Seasonal lowland rivers
Freshwater	F1 Rivers and streams	F 1.6 Episodic arid rivers
Freshwater	F1 Rivers and streams	F 1.7 Large lowland rivers
Freshwater	F2 Lakes	F2.1 Large permanent freshwater lakes
Freshwater	F2 Lakes	F2.2 Small permanent freshwater lakes
Freshwater	F2 Lakes	F2.3 Seasonal freshwater lakes
Freshwater	F2 Lakes	F2.4 Freeze-thaw freshwater lakes
Freshwater	F2 Lakes	F2.5 Ephemeral freshwater lakes
Freshwater	F2 Lakes	F2.6 Permanent salt and soda lakes
Freshwater	F2 Lakes	F2.7 Ephemeral salt lakes
Freshwater	F2 Lakes	F2.8 Artesian springs and oases
Freshwater	F2 Lakes	F2.9 Geothermal pools and wetlands
Freshwater	F2 Lakes	F2.10 Subglacial lakes
Freshwater	F3 Artificial fresh waters	F3.1 Large reservoirs
Freshwater	F3 Artificial fresh waters	F3.2 Constructed lacustrine wetlands
Freshwater	F3 Artificial fresh waters	F3.3 Rice paddies
Freshwater	F3 Artificial fresh waters	F3.4 Freshwater aquafarms
Freshwater	F3 Artificial fresh waters	F3.5 Canals, ditches and drains
Freshwater-Marine	FM1 Semi-confined transitional waters	FM1.1 Deepwater coastal inlets
Freshwater-Marine	FM1 Semi-confined transitional waters	FM 1.2 Permanently open riverine estuaries and bays
Freshwater-Marine	FM1 Semi-confined transitional waters	FM 1.3 Intermittently closed and open lakes and lagoons
Marine	M1 Marine shelves	M1.1 Seagrass meadows
Marine	M1 Marine shelves	M1.2 Kelp forests
Marine	M1 Marine shelves	M1.3 Photic coral reefs
Marine	M1 Marine shelves	M1.4 Shellfish beds and reefs
Marine	M1 Marine shelves	M1.5 Photo-limited marine animal forests
Marine	M1 Marine shelves	M1.6 Subtidal rocky reefs
Marine	M1 Marine shelves	M1.7 Subtidal sand beds
Marine	M1 Marine shelves	M1.8 Subtidal mud plains
Marine	M1 Marine shelves	M1.9 Upwelling zones
Marine	M1 Marine shelves	M1.10 Rhodolith/Maërl beds
Marine	M2 Pelagic ocean waters	M2.1 Epipelagic ocean waters
Marine	M2 Pelagic ocean waters	M2.2 Mesopelagic ocean waters
Marine	M2 Pelagic ocean waters	M2.3 Bathypelagic ocean waters
Marine	M2 Pelagic ocean waters	M2.4 Abyssopelagic ocean waters
Marine	M2 Pelagic ocean waters	M2.5 Sea ice
Marine	M3 Deep sea floors	M3.1 Continental and island slopes

**Realm**	**Biome**	**Ecosystem Functional Group (EFG)**
Marine	M3 Deep sea floors	M3.2 Marine canyons
Marine	M3 Deep sea floors	M3.3 Abyssal plains
Marine	M3 Deep sea floors	M3.4 Seamounts, ridges and plateaus
Marine	M3 Deep sea floors	M3.5 Deepwater biogenic beds
Marine	M3 Deep sea floors	M3.6 Hadal trenches and troughs
Marine	M3 Deep sea floors	M3.7 Chemosynthetically-based ecosystems
Marine	M4 Anthropogenic marine systems	M4.1 Submerged artificial structures
Marine	M4 Anthropogenic marine systems	M4.2 Marine aquafarms
Marine-Terrestrial	MT1 Shoreline systems	MT1.1 Rocky shores
Marine-Terrestrial	MT1 Shoreline systems	MT1.2 Muddy shores
Marine-Terrestrial	MT1 Shoreline systems	MT1.3 Sandy shores
Marine-Terrestrial	MT1 Shoreline systems	MT1.4 Boulder and cobble shores
Marine-Terrestrial	MT2 Supralittoral coastal systems	MT2.1 Coastal shrublands and grasslands
Marine-Terrestrial	MT2 Supralittoral coastal systems	MT2.2 Large seabird and pinniped colonies
Marine-Terrestrial	MT3 Anthropogenic shorelines	MT3.1 Artificial shores
Marine-Freshwater-Terrestrial	MFT1 Brackish tidal systems	MFT 1.1 Coastal river deltas
Marine-Freshwater-Terrestrial	MFT1 Brackish tidal systems	MFT1.2 Intertidal forests and shrublands
Marine-Freshwater-Terrestrial	MFT1 Brackish tidal systems	MFT 1.3 Coastal saltmarshes and reedbeds

T1. Tropical-subtropical forests biome

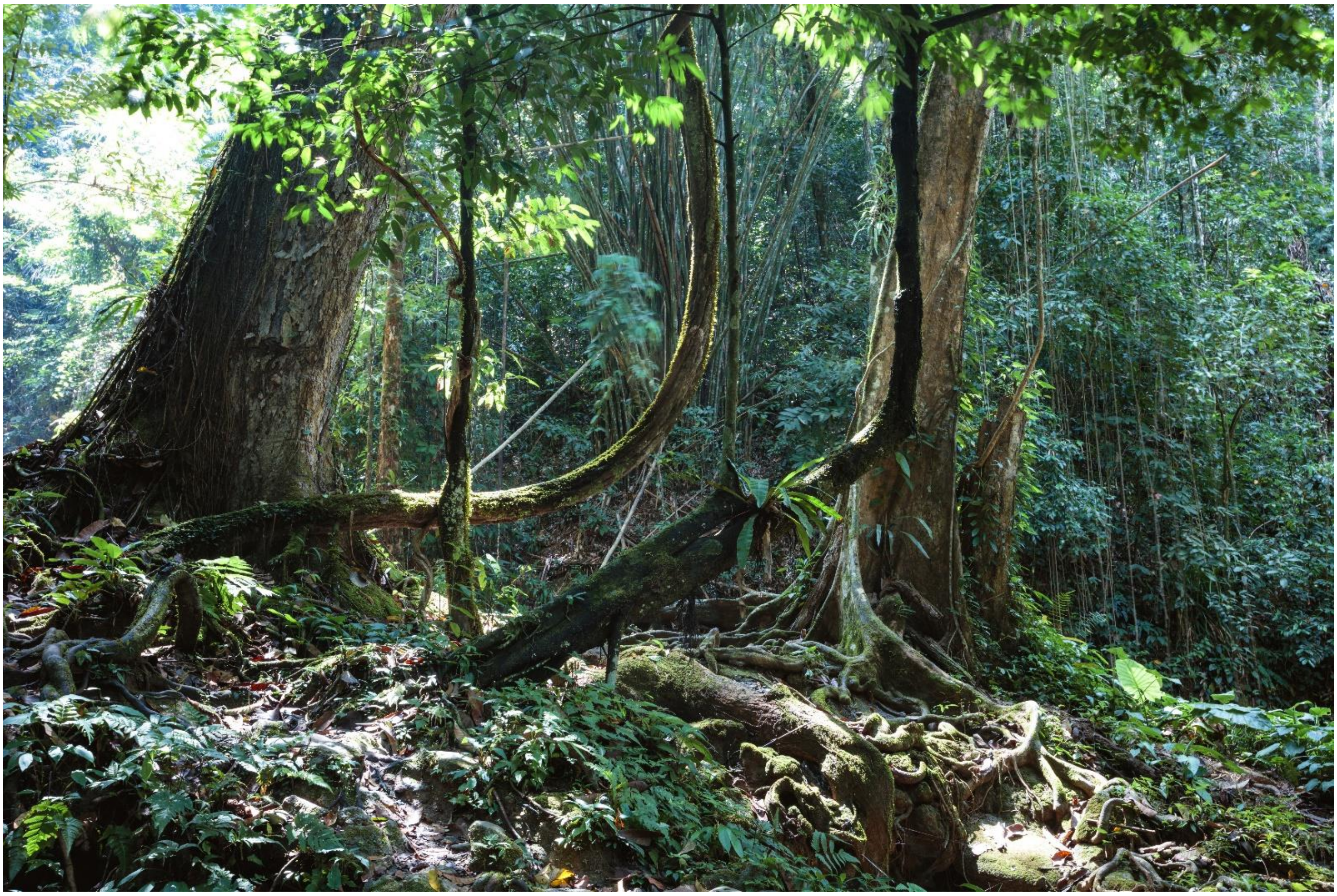

Tropical rainforest, Phang Nga bay, Thailand.

Credit: Matteo Colombo / Getty Images

The Tropical-subtropical forests biome includes moderate to highly productive ecosystems with closed tree canopies occurring at lower latitudes north and south of the equator. Fragmented occurrences extend to the subtropics in suitable mesoclimates.

High primary productivity is underpinned by high insolation, warm temperatures, relatively low seasonal variation in day length and temperature (increasing to the subtropics), and strong water surpluses associated with the intertropical convergence zone extending to wetter parts of the seasonal tropics and subtropics. Productivity and biomass vary in response to: i) strong rainfall gradients associated with seasonal migration of the intertropical convergence zone, ii) altitudinal gradients in precipitation, cloud cover, and temperatures, and iii) edaphic gradients that influence the availability of soil nutrients.

Species diversity and the complexity of both vegetation and trophic structures are positively correlated with standing biomass and primary productivity, however, trophic webs and other ecosystem processes are strongly regulated from the bottom-up by the dominant photoautotrophs (trees), which fix abundant energy and carbon, engineer habitats for many other organisms, and underpin feedbacks related to nutrient and water cycling and regional climate.

Complex nutrient cycling and/or sequestering mechanisms are common, countering the high potential for soil nutrient leaching due to high rainfall. Plant species exhibit leaf plasticity, shade tolerance, and gap-phase dynamics in response to the periodic opening of canopy gaps initiated by tree death, storm damage, and lightning strikes. Fires may occur in ecotonal areas between these forests and savannas.

Biogeographic legacies result in strong compositional distinctions and consequently some functional differences among land masses within the biome.

T1.1 Tropical/Subtropical lowland rainforests

Ecosystem properties: These closed-canopy forests are renowned for their complex structure and high primary productivity, which support high functional and taxonomic diversity. At subtropical latitudes they transition to warm temperate forests (T2.4). Bottom-up regulatory processes are fuelled by large autochthonous energy sources that support very high primary productivity, biomass and LAI. The structurally complex, multi-layered, evergreen tree canopy has a large range of leaf sizes (typically macrophyll-notophyll) and high SLA, reflecting rapid growth and turnover. Diverse plant life forms include buttressed trees, bamboos (sometimes abundant), palms, epiphytes, lianas and ferns, but grasses and hydrophytes are absent or rare. Trophic networks are complex and vertically stratified with low exclusivity and diverse representation of herbivorous, frugivorous, and carnivorous vertebrates. Tree canopies support a vast diversity of invertebrate herbivores and their predators. Mammals and birds play critical roles in plant diaspore dispersal and pollination. Growth and reproductive phenology may be seasonal or unseasonal, and reproductive masting is common in trees and regulates diaspore predation. Fungal, microbial, and diverse invertebrate decomposers and detritivores dominate the forest floor and the subsoil. Diversity is high across taxa, especially at the upper taxonomic levels of trees, vertebrates, fungi, and invertebrate fauna. Neutral processes, as well as micro-niche partitioning, may have a role in sustaining high diversity, but evidence is limited. Many plants are in the shade, forming seedling banks that exploit gap-phase dynamics initiated by individual tree-fall or stand-level canopy disruption by tropical storms (e.g. in near-coastal forests). Seed banks regulated by dormancy are uncommon. Many trees exhibit leaf plasticity enabling photosynthetic function and survival in deep shade, dappled light or full sun, even on a single individual. A few species germinate on tree trunks, gaining quicker access to canopy light, while roots absorb microclimatic moisture until they reach the soil.

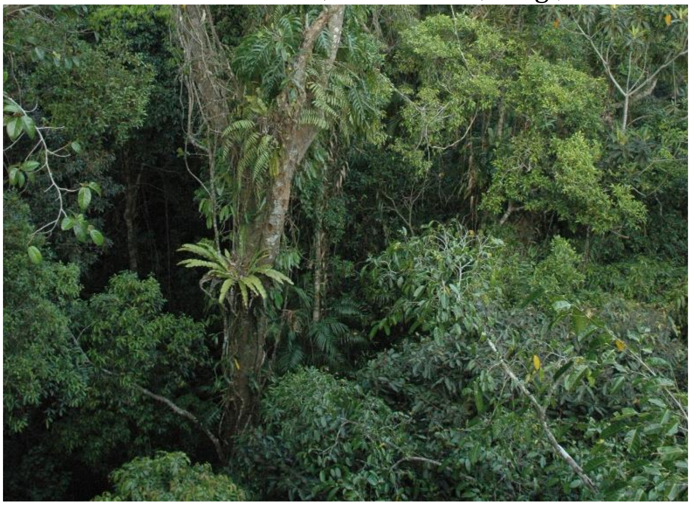

Tropical rainforest, Daintree, northeast Australia.
Credit: David Keith

Ecological drivers: Precipitation exceeds evapo-transpiration with low intra- and inter-annual variability, creating a reliable year-round surplus, while closed tree canopies maintain humid microclimate and shade. Temperatures are warm with low-moderate diurnal and seasonal variation (mean winter minima rarely <10°C except in subtropical transitional zones). Soils are moist but not regularly inundated or peaty (see TF1.1) and vary widely in nutrient status. Most nutrient capital is sequestered in vegetation or cycled through the dynamic litter layer, critical for retaining nutrients that would otherwise be leached or lost to runoff. In some coastal regions outside equatorial latitudes (mostly >10° and excluding extensive forests in continental America and Africa), decadal regimes of tropical storms drive cycles of canopy destruction and renewal.

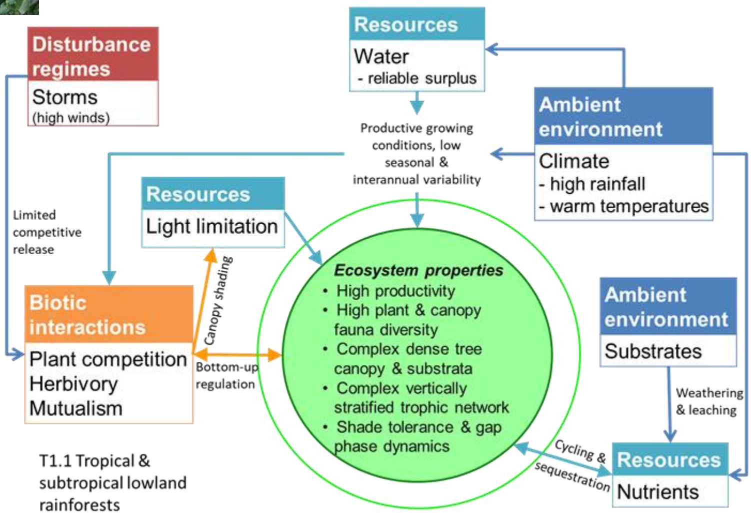

Distribution: Humid tropical and subtropical regions in Central and West Africa, Southeast Asia, Oceania, northeast Australia, Central and tropical South America and the Caribbean.

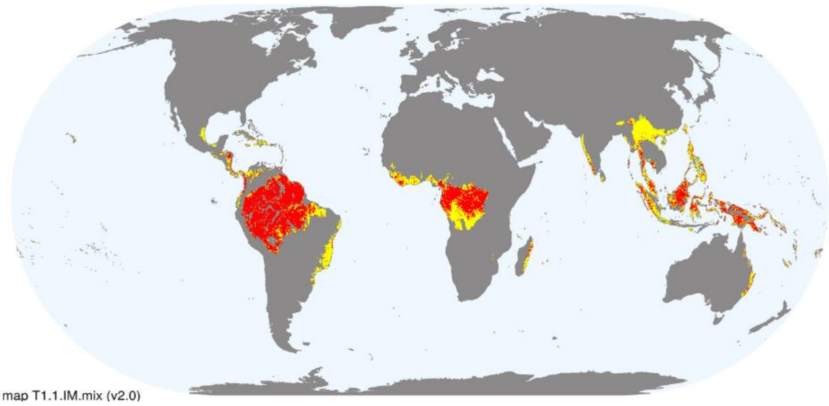

map T1.1.IM.mix (v2.0)

Map is for illustrative purposes only and does not support spatial analyses unless formally validated.

References:

Ashton PS, Seidler R (2014) *On the forests of tropical Asia: lest the memory fade.* Kew Publishing: Kew.

Corlett RT, Primack RB (2011) *Tropical Rain Forests: An ecological and biogeographical comparison.* Wiley-Blackwell.

Contributors: RT Pennington, J Franklin, NA Brummitt, A Etter, KR Young, RT Corlett, DA Keith

T1.2 Tropical/Subtropical dry forests and thickets

Ecosystem properties: These closed-canopy forests and thickets have drought-deciduous or semi-deciduous phenology in at least some woody plants (rarely fully evergreen), and thus seasonally high LAI. Strongly seasonal photoautotrophic productivity is limited by a regular annual water deficit/surplus cycle. Diversity is lower across most taxa than T1.1, but tree and vertebrate diversity is high relative to most other forest systems. Plant growth forms and leaf sizes are less diverse than in T1.1. Grasses are rare or absent, except on savanna ecotones, due to canopy shading and/or water competition, while epiphytes, ferns, bryophytes, and forbs are present but limited by seasonal drought. Trophic networks are complex with low exclusivity and diverse representation of herbivorous, frugivorous, and carnivorous vertebrates. Fungi and other microbes are important decomposers of abundant leaf litter and N-fixing plants can be abundant. Many woody plants are dispersed by wind and some by vertebrates. Most nutrient capital is sequestered in vegetation or cycled through the litter layer. Trees typically have thin bark and low fire tolerance and can recruit in shaded microsites, unlike many in savannas. Plants are tolerant of seasonal drought but can exploit moisture when it is seasonally available through high SLA and plastic productivity. Gap-phase dynamics are driven primarily by individual tree-fall and exploited by seedling banks and vines (seedbanks are uncommon). These forests may be involved in fire-regulated stable-state dynamics with savannas.

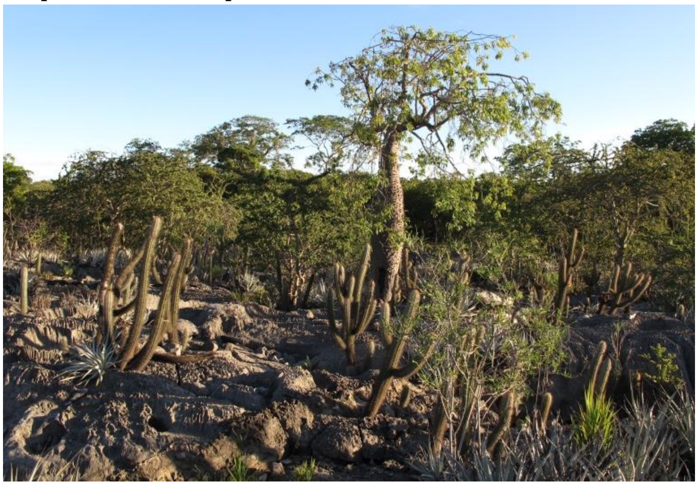

Tropical dry forest, northern Minas Gerais, Brazil.
Credit: Toby Pennington

Ecological drivers: Overall water surplus (or small deficit <100 mm), but a substantial seasonal deficit in winter in which little or no rain falls within a 4–7-month period. Warm temperatures (minima rarely <10°C) with low-moderate diurnal and seasonal variability in the tropics, but greater seasonal variability in subtropical continental areas. Diverse substrates generally produce high levels of nutrients. Tropical storms may be important disturbances in some areas but flammability is low due to limited ground fuels except on savanna ecotones.

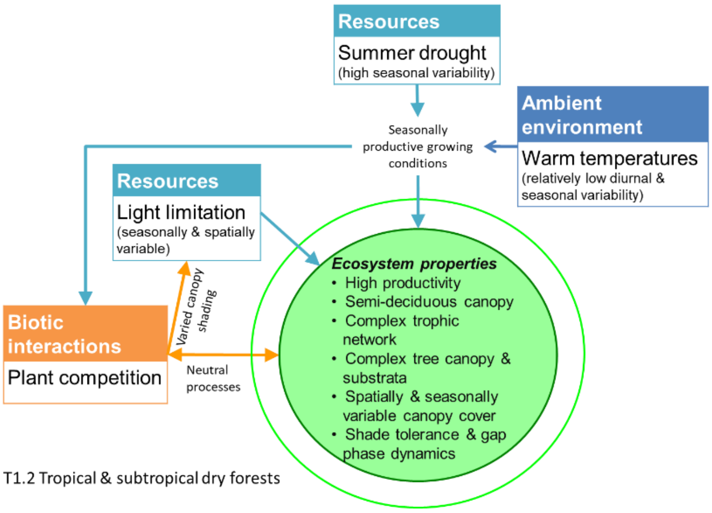

Distribution: Seasonally dry tropical and subtropical regions in Central and West Africa, Madagascar, southern Asia, north and northern and eastern Australia, the Pacific, Central and South America and the Caribbean.

References:

Bunyavejchewin SC, Baker P, Davis SJ (2011) Seasonally dry tropical forests in continental southeast Asia - structure, composition, and dynamics. *The ecology and conservation of seasonally dry forests in Asia* WJ McShea, SJ Davis, N Bhumpakphan, (Eds), pp. 9-35. Smithsonian Institution Scholarly Press, Washington DC. ISBN 9781935623021.

DRYFLOR, Banda-R K, Delgado-Salinas A, Dexter KG et al. (2016) Plant diversity patterns in neotropical dry forests and their conservation implications. *Science* 353:1383-1387.

Murphy PG, Lugo AE (1986) Ecology of tropical dry forest. *Annual Review of Ecology and Systematics* 17:67–88.

Pennington RT, Lewis G, Ratter J (2006) *Neotropical savannas and dry forests: plant diversity, biogeography and conservation* CRC Press, Florida.

Sanchez-Azofeifa A, Powers JS, Fernandes GW, Quesada M (2019) 'Tropical Dry Forests in the Americas: ecology, conservation, and management.' (CRC Press: Florida)

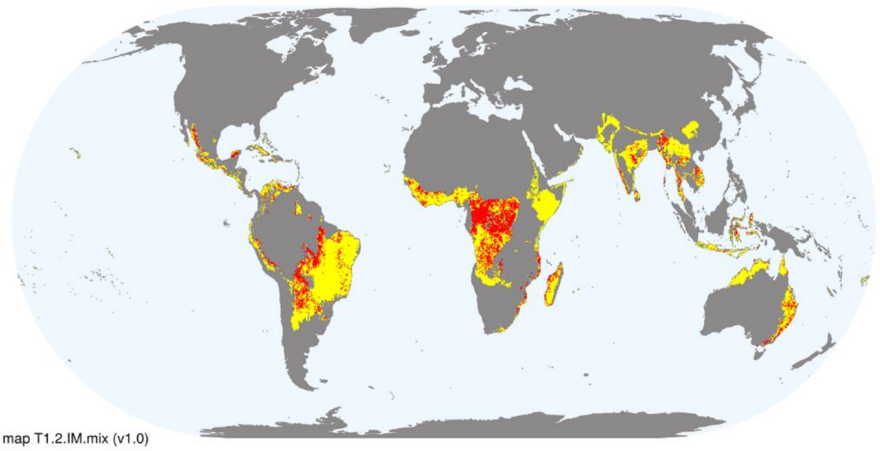

map T1.2.IM.mix (v1.0)

Map is for illustrative purposes only and does not support spatial analyses unless formally validated.

Contributors: DA Keith, NA Brummitt, KR Young, RT Corlett, A Etter

T1.3 Tropical/Subtropical montane rainforests

Ecosystem properties: Closed-canopy evergreen forests on tropical mountains usually have a single-layer low tree canopy (~5–20m tall) with small leaf sizes (microphyll-notophyll) and moderate-high SLA. They transition to lowland rainforests (T1.1) with decreasing altitude and to warm temperate forests (T2.4) at higher latitudes. Structure and taxonomic diversity become more diminutive and simpler with altitude, culminating in elfinwood forms. Conspicuous epiphytic ferns, bryophytes, lichens, orchids, and bromeliads drape tree branches and exploit atmospheric moisture (cloud stripping), but grasses are rare or absent, except for bamboos in some areas. Moderate productivity fuelled by autochthonous energy is limited by high exposure to UV-B radiation, cool temperatures, and sometimes by shallow soil or wind exposure. Limited energy and sequestration in humic soils may limit N and P uptake. Growth and reproductive phenology is usually seasonal. Plant propagules are dispersed mostly by wind and territorial birds and mammals. Tree diversity is moderate to low, while epiphytes are diverse, but there is often high local endemism at higher altitudes in most groups, especially amphibians, birds, plants, and invertebrates. Gap-phase dynamics are driven by tree-fall, landslides, lightning strikes, or in some areas more rarely by extreme wind storms. Seedling banks are common (seedbanks are uncommon) and most plants are shade tolerant and can recruit in the shade.

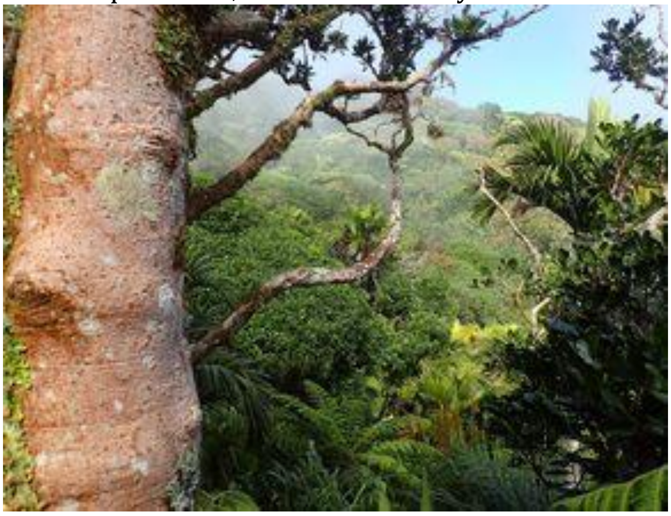

Cloud forest, Mt Gower, Lord Howe Island, Oceania.
Credit: David Keith

Ecological drivers: Substantial cloud moisture and high humidity underpin a reliable year-round rainfall surplus over evapotranspiration. Altitudinal gradients in temperature, precipitation, and exposure are pivotal in ecosystem structure and function. Frequent cloud cover from orographic uplift and closed tree canopies maintain a moist microclimate and shady conditions. Temperatures are mild-cool with occasional frost. Seasonal variability is low-moderate but diurnal variability is moderate-high. Winter monthly mean minima may be around 0°C in some areas. Landslides are a significant form of disturbance that drives successional dynamics on steep slopes and is exacerbated by extreme rainfall events. Mountains experience elevated UV-B radiation with altitude and, in some regions, are exposed to local or regional storms.

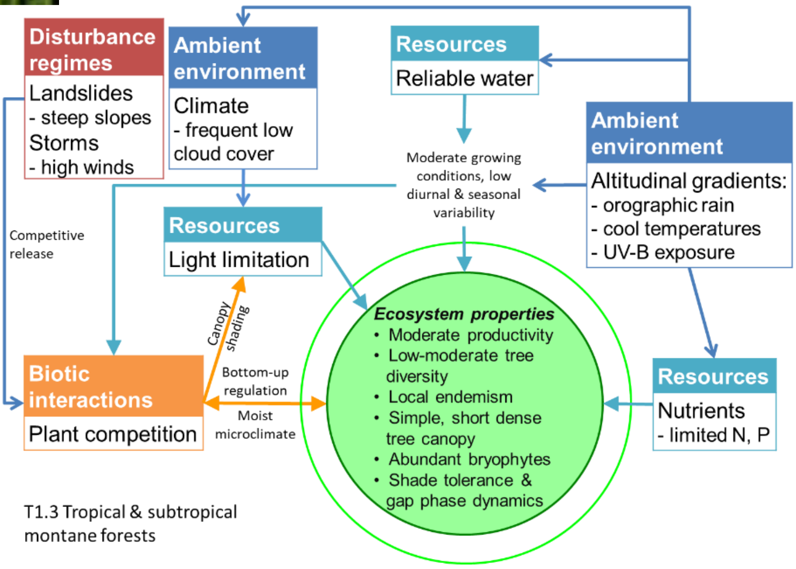

Distribution: Humid tropical and subtropical regions in East Africa, East Madagascar, Southeast Asia, west Oceania, northeast Australia, Central and tropical South America.

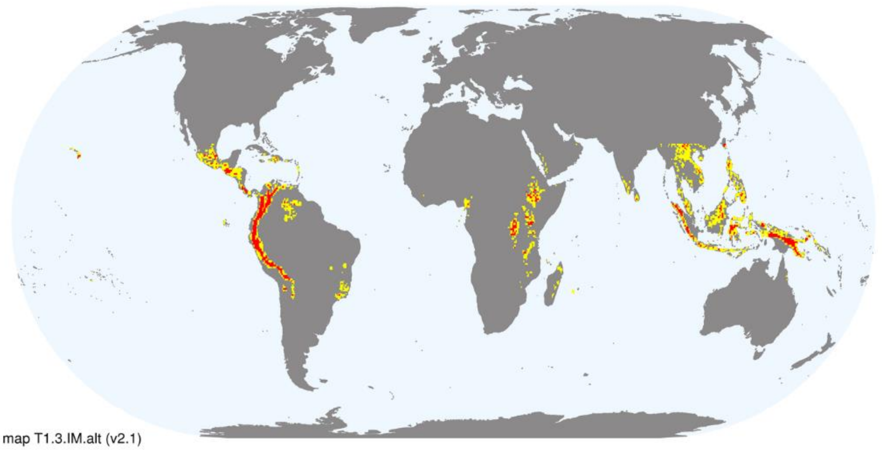

Map is for illustrative purposes only and does not support spatial analyses unless formally validated.

References:

Ashton PS, Seidler R (2014) *On the forests of tropical Asia: lest the memory fade* Kew Publishing: Kew.

Gradstein SR, Homeier J, Gansert D (2008) *The tropical mountain forest – patterns and processes in a biodiversity hotspot* Biodiversity and Ecology Series 2. Göttingen, Centre for Biodiversity and Ecology.

Grubb PJ (1977) Control of forest growth and distribution on wet tropical mountains. *Ann. Rev. Ecol. Syst.* **8**, 83-107.

Hamilton LS, Juvik JO, Scatena FN (1995) Tropical Montane Cloud Forests. *Ecological Studies* 110. Springer-Verlag, Berlin.

T1.4 Tropical heath forests

Ecosystem properties: Structurally simple evergreen forests with high densities of thin stems, closed to open uniform canopies, typically 5–20 m tall and uniform with a moderate to high LAI. Productivity is lower than in other tropical forests, weakly seasonal and limited by nutrient availability and in some cases by soil anoxia, but decomposition is rapid. Plant traits such as insectivory, N-fixing microbial associations and ant mutualisms are well represented, suggesting adaptive responses to nitrogen deficiency. Plant insectivory aside, trophic networks are simple compared to other tropical forests. Diversity of plant and animal taxa is also relatively low, but dominance and endemism are proportionately high. Tree foliage is characterised by small (microphyll-notophyll) leaves with lower SLA than other tropical forests. Leaves are leathery and often ascending vertically, enabling more light penetration to ground level than in other tropical forests. Tree stems are slender (generally <20 cm in diameter), sometimes twisted, and often densely packed and without buttresses. Epiphytes are usually abundant but lianas are rare and ground vegetation is sparse, with the forest floor dominated by insectivorous vascular plants and bryophytes.

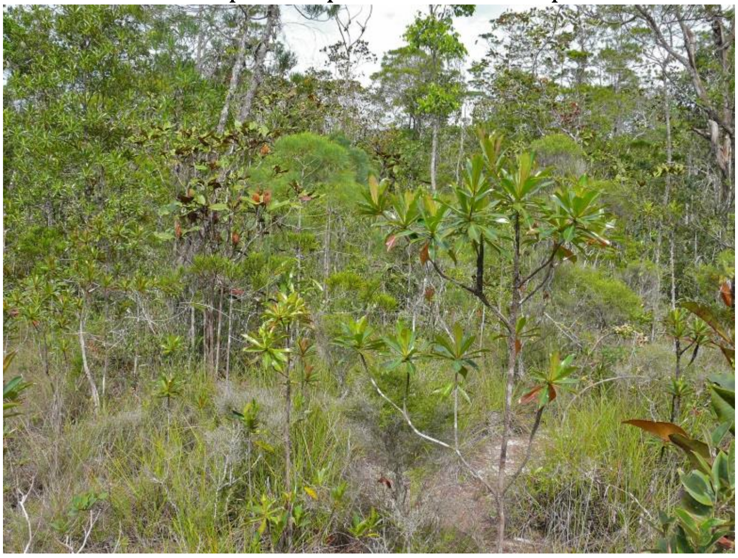

Kerangas Sundaland Heath Forest, Bako National Park, Malaysia.
Credit: Bernard Dupont

Ecological drivers: These forests experience an overall water surplus, but productivity is limited by deep sandy low-nutrient acidic substrates, which are leached by high rainfall. Acidity promotes high Al levels that inhibit root growth. Most nutrients are retained in vegetation. Downward movement of clay and organic particles through the soil profile results in a deep, white sandy horizon capped by a thin grey surface horizon (typical of podzols), limiting the capacity of the soil to retain nutrients (especially nitrogen) and moisture within the shallow rooting zone. Hence they are prone to inter-annual droughts, but waterlogging may occur where the water table is close to the surface, resulting in periodic anoxia within the root zone. Landscape water-table gradients result in surface mosaics in which heath forests may be juxtaposed with more waterlogged peat forests (TF1.1) and palustrine wetland systems (TF1.2).

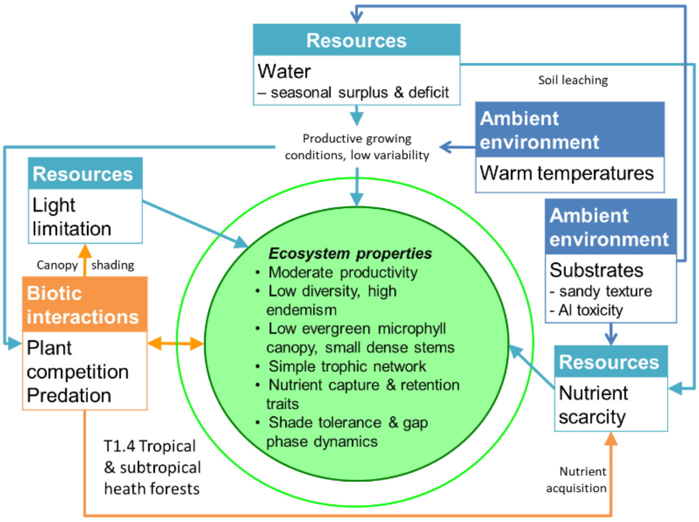

Distribution: Scattered through northwest and west Amazonia, possibly Guiana, and Southeast Asia, notably in the Rio Negro catchment and southern Kalimantan. Poorly known in Africa, but possibly in the Gabon region.

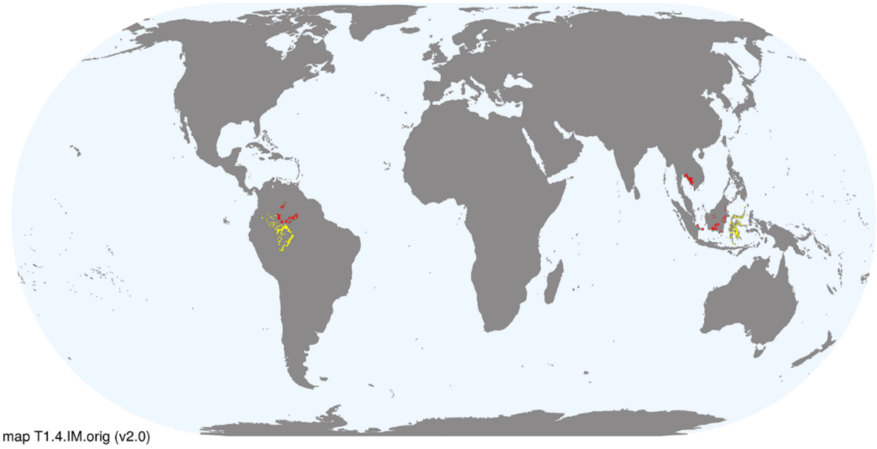

map T1.4.IM.orig (v2.0)

References:

Adeney JM, Christensen NL, Vicentini A, Cohn-Haft M (2016) White-sand ecosystems in Amazonia. *Biotropica* 48: 7–23.

Ashton PS, Seidler R (2014) *On the forests of tropical Asia: lest the memory fade* Kew Publishing: Kew.

Fortunel C, Paine CET; Fine PVA, J. B. Kraft NJB, Baraloto C (2014) Environmental factors predict community functional composition in Amazonian forests. *Journal of Ecology* 102: 145–155.

Miyamoto K, Rahajoe JS, Kohyama T (2007) Forest structure and primary productivity in a Bornean heath forest. *Biotropica* 39: 35–42.

Miyamoto K, Wagai R, Aiba S, Nilus R (2016) Variation in aboveground stand structure and fine-root biomass of Bornean heath (kerangas) forests in relation to altitude and soil nitrogen availability. *Trees-Structure and Function* 30: 385-394.

T2. Temperate-boreal forests and woodlands biome

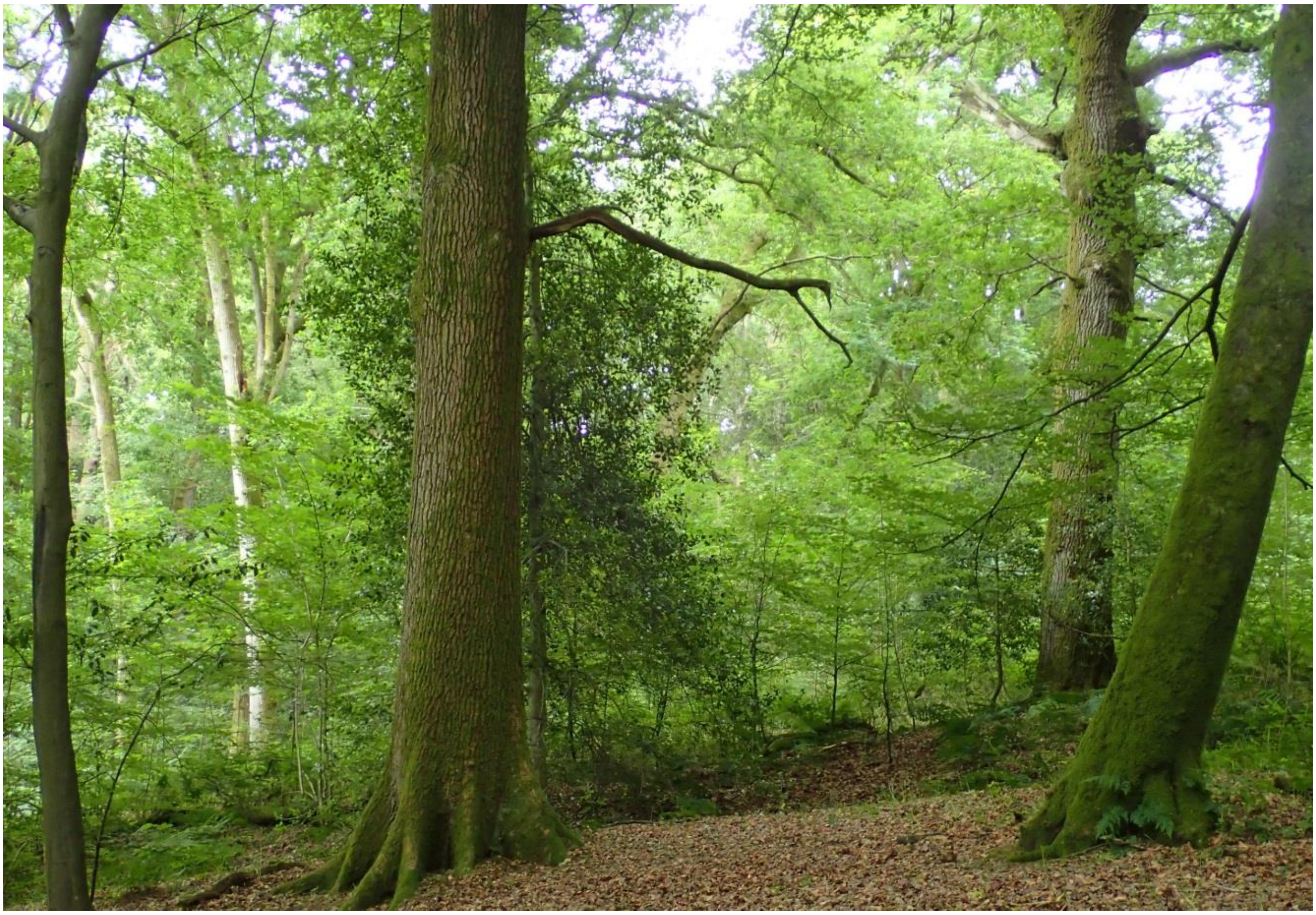

Petworth, Sussex, England.

Credit: David Keith

Temperate-boreal forests and woodlands biome include moderate to highly productive tree-dominated systems with a wide range of physiognomic and structural expressions distributed from warm-temperate to boreal latitudes.

Although generally less diverse than Tropical-subtropical forests (T1) in taxa such as flowering plants, primates, and birds, these Temperate-boreal forests exhibit greater temporal and spatial variability in productivity, biomass, phenology, and leaf traits of trees. Temporal variability is expressed primarily through seasonal variation in water balance and/or temperature, which regulate the length and timing of growing and breeding seasons. Inter-annual variation is usually less important than in some other biomes (e.g. T5), but nonetheless may play significant roles in resource availability and disturbance regimes (e.g. fire and storms).

Gradients in minimum temperatures, soil nutrients, and fire regimes differentiate ecosystem functional groups within this biome. These influence traits such as leaf form (broadleaf vs. needleleaf), leaf phenology (evergreen vs. deciduous), ecophysiological and morphological traits promoting nutrient acquisition and conservation, and morphological traits related to flammability, fire resistance, and recovery.

The dominant photoautotrophs (trees) engineer habitats and underpin trophic webs. Resource gradients exert strong bottom-up controls on trophic processes, but in some temperate forests, fires are significant top-down consumers of biomass, as well as influencing flammability feedbacks and timing of life-history processes, such as reproduction and recruitment

Contributors: DA Keith, D Faber-Langendoen, T Kontula, J Franklin, NA Brummitt

T2.1 Boreal and temperate high montane forests and woodlands

Ecosystem properties: Evergreen, structurally simple forests and woodlands in cold climates are dominated by needle-leaf conifers and may include a subdominant component of deciduous trees, especially in disturbed sites, accounting for up to two-thirds of stand-level leaf biomass. Boreal forests are generally less diverse, more cold-tolerant and support a more migratory fauna than temperate montane forests. Structure varies from dense forest up to 30 m tall to stunted open woodlands <5 m tall. Large trees engineer habitats of many fungi, non-vascular plants, invertebrates, and vertebrates that depend on rugose bark, coarse woody debris, or large tree canopies. Energy is mainly from autochthonous sources but may include allochthonous subsidies from migratory vertebrates. Primary productivity is limited by seasonal cold and may also be limited by water deficit on coarse textured soils. Forested bogs occupy peaty soils (TF1.6). Seasonal primary productivity may sustain a trophic web with high densities of small and large herbivores (e.g. hare, bear, deer, and insects), with feline, canine, and raptor predators. Browsers are top-down regulators of plant biomass and cyclers of nitrogen, carbon, and nutrients. Forest structure may be disrupted by insect defoliation or fires on multi-decadal cycles. Tree recruitment occurs semi-continuously in gaps or episodically after canopy fires and may be limited by spring frost, desiccation, permafrost fluctuations, herbivory, and surface fires. Plants and animals have strongly seasonal growth and reproductive phenology and possess morphological, behavioural, and ecophysiological traits enabling cold-tolerance and the exploitation of short growing seasons. Plant traits include bud protection, extra-cellular freezing tolerance, hardened evergreen needle leaves with low SLA or deciduous leaves with high SLA, cold-stratification seed dormancy, seasonal geophytic growth forms, and vegetative storage organs. Tracheids in conifers confer resistance to cavitation in drought by compartmentalising water transport tissues. Some large herbivores and most birds migrate to winter habitats from the boreal zone, and thus function as mobile links, dispersing other biota and bringing allochthonous subsidies of energy and nutrients into the system. Hibernation is common among sedentary vertebrates, while insect life cycles have adult phases cued to spring emergence.

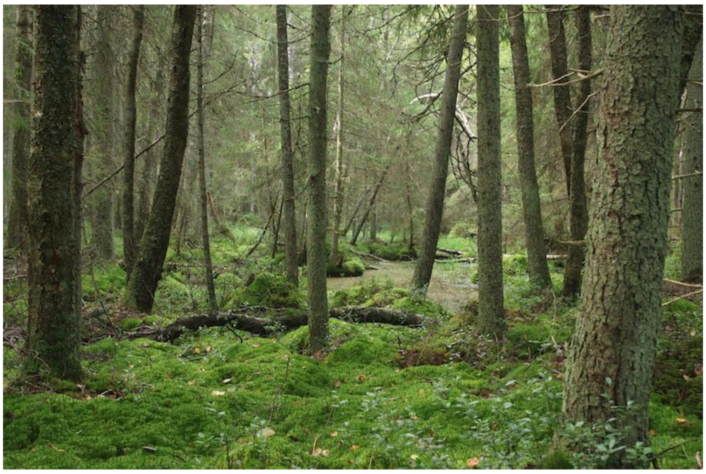

Boreal forest with old growth spuce, near Sideby, Finland.
Credit: Staffan Storteir

Ecological drivers: These systems are driven by large seasonal temperature ranges, cold winters with prolonged winter snow, low light, short growing seasons (1–3 months averaging >10°C) and severe post-thaw frosts. There is an overall water surplus, but annual precipitation can be <200 mm. Soil moisture recharged by winter snow sustains the system through evapotranspiration peaks in summer, but moisture can be limiting where these systems extend to mountains in warm semi-arid latitudes. The acid soils usually accumulate peat and upper horizons may be frozen in winter. Forests may be prone to lightning-induced canopy fires on century time scales and surface fires on multi-decadal scales.

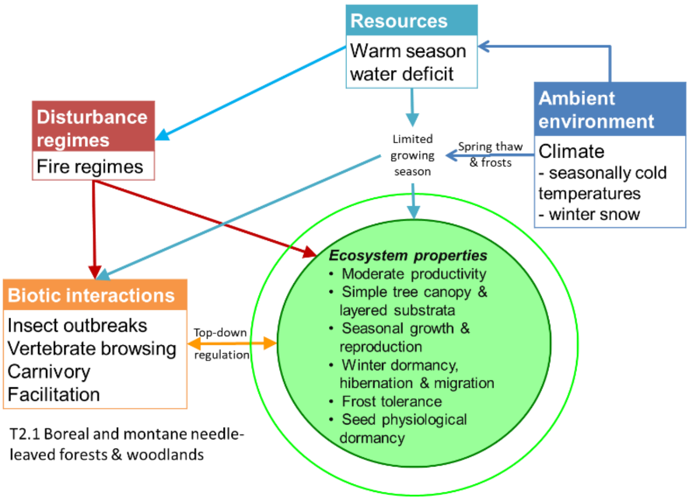

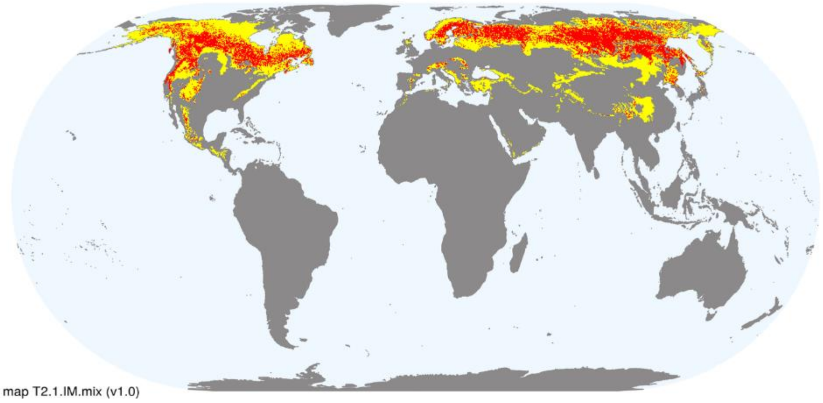

Distribution: Boreal distribution across Eurasia and North America, extending to temperate (rarely subtropical) latitudes on mountains.

References:

Bonan GB, Shugart HH (1989) Environmental Factors and Ecological Processes in Boreal Forests. *Annual Review of Ecology and Systematics* 20: 1–28.

Crawford RMM (2013) *Tundra-taiga biology* Ch 5. Oxford University Press, Oxford.

Larsen JA (1980) *The boreal ecosystem* (Ed. T. T. Kozlowski) Academic Press, New York.

Map is for illustrative purposes only and does not support spatial analyses unless formally validated.

Contributors: DA Keith, NA Brummitt, F Essl, D Faber-Langendoen

T2.2 Deciduous temperate forests

Ecosystem properties: These structurally simple, winter deciduous forests have high productivity and LAI in summer. Winter dormancy, hibernation and migration are common life histories among plants and animals enabling cold avoidance. Local endemism is comparatively low and there are modest levels of diversity across major taxa. The forest canopy comprises at least two-thirds deciduous broad-leaf foliage (notophylll-mesophyll) with high SLA and up to one-third evergreen (typically needleleaf) cover. As well as deciduous woody forms, annual turnover of above-ground biomass also occurs some in non-woody geophytic and other ground flora, which are insulated from the cold beneath winter snow and flower soon after snowmelt before tree canopy closure. Annual leaf turnover is sustained by fertile substrates and water surplus, with nutrient withdrawal from foliage and storage of starch prior to fall. Tissues are protected from cold by supercooling rather than extra-cellular freeze-tolerance. Dormant buds are insulated from frost by bracts or by burial below the soil in some non-woody plants. Fungal and microbial decomposers play vital roles in cycling carbon and nutrients in the soil surface horizon. Despite highly seasonal primary productivity, the trophic network includes large browsing herbivores (deer), smaller granivores and herbivores (rodents and hares), and mammalian predators (canids and felines). Most invertebrates are seasonally active. Behavioural and life-history traits allow animals to persist through cold winters, including through dense winter fur, food caching, winter foraging, hibernation, dormant life phases, and migration. Migratory animals provide allochthonous subsidies of energy and nutrients and promote incidental dispersal of other biota. Browsing mammals and insects are major consumers of plant biomass and cyclers of nitrogen, carbon, and nutrients. Deciduous trees may be early colonisers of disturbed areas (later replaced by evergreens) but are also stable occupants across large temperate regions. Tree recruitment is limited by spring frost, allelopathy, and herbivory, and occurs semi-continuously in gaps. Herbivores may influence densities of deciduous forest canopies by regulating tree regeneration. Deciduous leaf fall may exert allelopathic control over tree seedlings and seasonal ground flora.

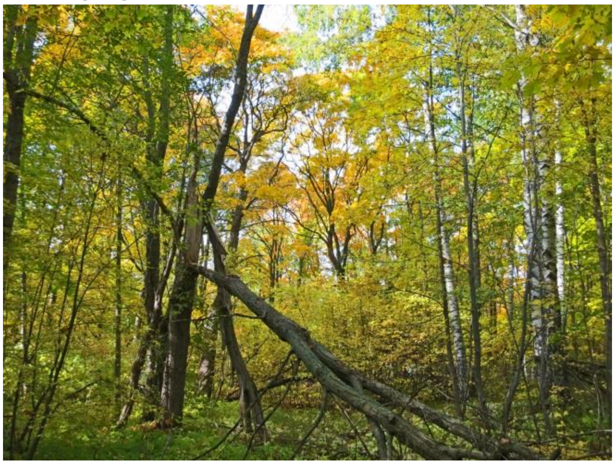

Deciduous forest during autumn leaf fall, Inkoo, Finland.
Credit: Anne Raunio

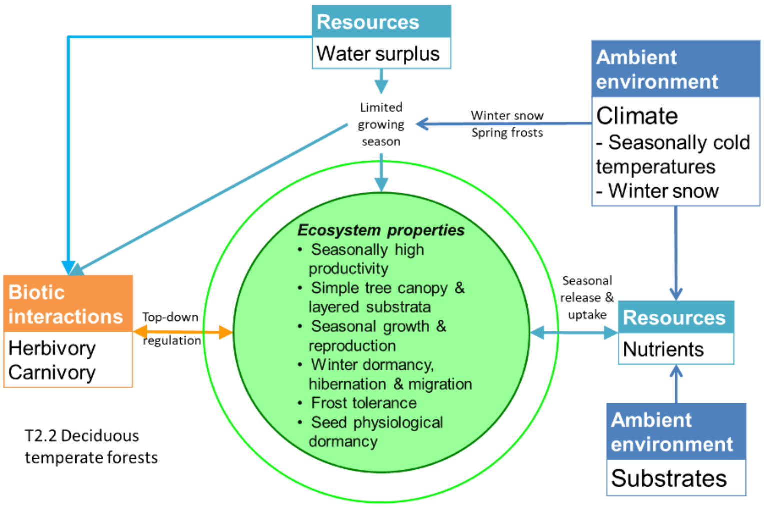

Ecological drivers: Phenological processes in these forests are driven by large seasonal temperature ranges, (mean winter temperatures <–1°C, summer means up to 22°C), typically with substantial winter snow and limited growing season, with 4–6 months >10°C, and severe post-thaw frosts. Fertile soils with high N levels and an overall water surplus support deciduous leaf turnover. Fires are uncommon.

Distribution: Cool temperate Europe (southwest Russia to British Isles), northeast Asia (northeast China, southern Siberia Korea, and Japan), and northeast America. Limited occurrences in warm-temperate zones of south Europe and Asia and the Midwest USA.

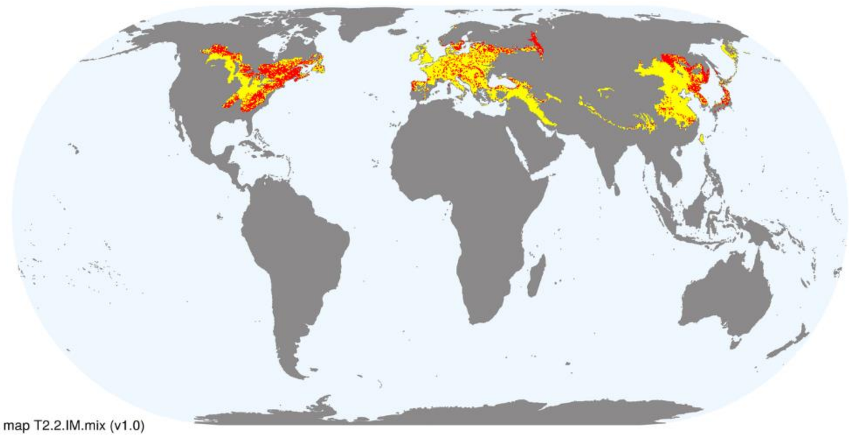

References:

Box EO, Fujiwara K (2015) *Warm temperate deciduous forests* Springer, Cham.

Röhrig E, Ulrich B (1991) Temperate deciduous forests. *Ecosystems of the world* Vol. 7. Elsevier, Amsterdam.

Map is for illustrative purposes only and does not support spatial analyses unless formally validated.

T2.3 Oceanic cool temperate rainforests

Ecosystem properties: Broadleaf and needleleaf rainforests in cool temperate climates have evergreen or semi-deciduous tree canopies with high LAI and mostly nanophyll-microphyll foliage. Productivity is moderate to high and constrained by strongly seasonal growth and reproductive phenology and moderate levels of frost tolerance. SLA may be high but lower than in T2.2. Evergreen trees typically dominate, but deciduous species become more abundant in sites prone to severe frost and/or with high soil fertility and moisture surplus. The smaller range of leaf sizes and SLA, varied phenology, frost tolerance, broader edaphic association, and wetter, cooler climate distinguish these forests from warm temperate forests (T2.4). Local or regional endemism is significant in many taxa. Nonetheless, energy sources are primarily autochthonous. Trophic networks are less complex than in other cool-temperate or boreal forests (T2.1 and T2.2), with weaker top-down regulation due to the lower diversity and abundance of large herbivores and predators. Tree diversity is low (usually <8–10 spp./ha), with abundant epiphytic and terrestrial bryophytes, pteridophytes, lichens, a modest range of herbs, and conspicuous fungi, which are important decomposers. The vertebrate fauna is mostly sedentary and of low-moderate diversity. Most plants recruit in the shade and some remain in seedling banks until gap-phase dynamics are driven by individual tree-fall, lightning strikes, or by extreme wind storms in some areas. Tree recruitment varies with tree masting events, which strongly influence trophic dynamics, especially of rodents and their predators.

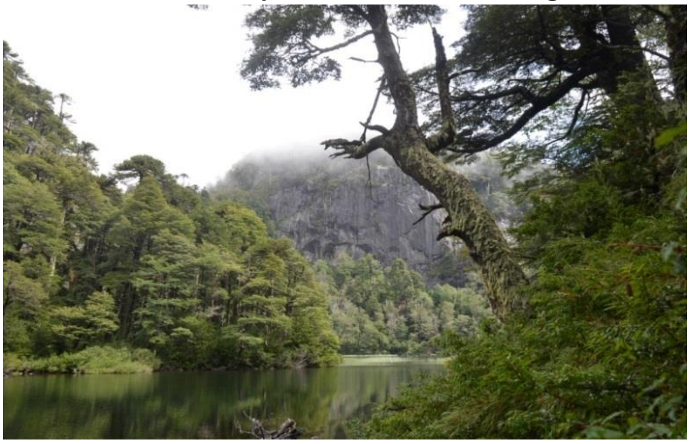

Cool temperate evergreen forest, Hwequehwe, Chile.
Credit: David Keith

Ecological drivers: There is a large water surplus, rarely with summer deficits. Rainfall is seasonal, borne on westerly winds peaking in winter months and inter-annual variability is relatively low. Cool winters (minima typically <0–5°C for 3 months) limit the duration of the growing season. Maritime air masses are the major supply of climatic moisture and moderate winters and summer temperatures. Light may be limited in winter by frequent cloud cover and high latitude. Intermittent winter snow does not persist for more than a few days or weeks. Soils are moderately fertile to infertile and may accumulate peat. Exposure to winter storms and landslides leaves imprints on forest structure in some regions. Fires are rare, occurring on century time scales when lightning (or human) ignitions follow extended droughts.

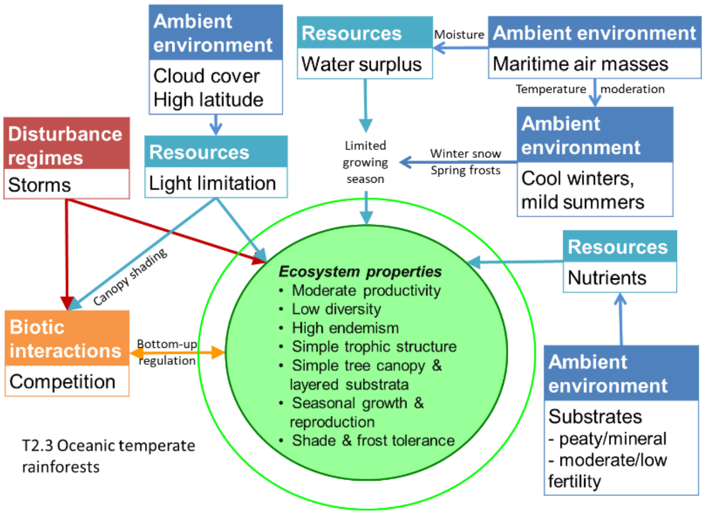

Distribution: Cool temperate coasts of Chile and Patagonia, New Zealand, Tasmania and the Pacific Northwest, rarely extending to warm-temperate latitudes on mountains in Chile, southeast Australia, and outliers above 2,500-m elevation in the New Guinea highlands. Some authors extend the concept to wet boreal forests on the coasts of northwest Europe, Japan, and northeast Canada.

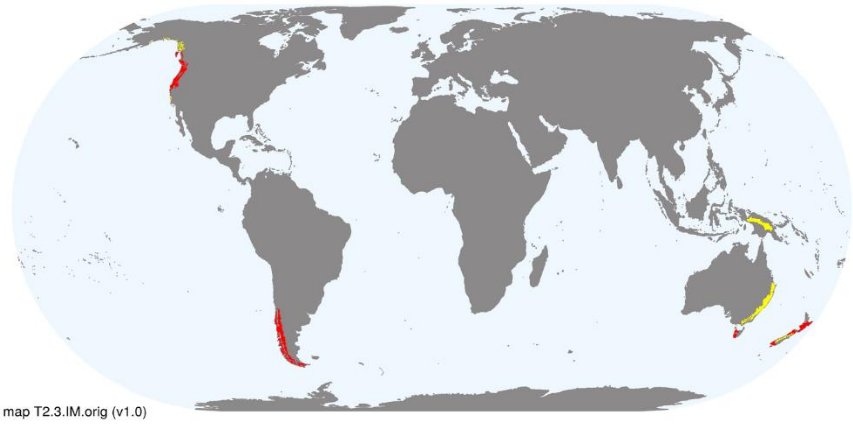

map T2.3.IM.orig (v1.0)

References:

DallaSala DA, Alaback P, Spribille T, von Wehrden H, Nauman RS (2011) Just what are temperate and boreal rainforests?. Temperate and boreal rainforests of the world: ecology and conservation (Ed. DA DellaSala), pp1-41. Island Press, Washington DC.

McGlone MS, Buitenwerf R, Richardson SJ (2016) The formation of the oceanic temperate forests of New Zealand. *New Zealand Journal of Botany* 54:128-155.

McGlone MS, Lusk CH, Armesto JJ (2016) Biogeography and ecology of south-temperate forests. *New Zealand Journal of Botany* 54: 94-99.

Map is for illustrative purposes only and does not support spatial analyses unless formally validated.

Contributors: DA Keith, NA Brummitt, D Faber-Langendoen, RT Corlett, MS McGlone

T2.4 Warm temperate laurophyll forests

Ecosystem properties: Relatively productive but structurally simple closed-canopy forests with high LAI occur in humid warm-temperate to subtropical climates. The tree canopies are more uniform than most tropical forests (T1.1 and T1.2) and usually lack large emergents. Their foliage is often leathery and glossy (laurophyll) with intermediate SLA values, notophyll-microphyll sizes, and prodigiously evergreen. Deciduous species are rarely scattered within the forest canopies. These features, and drier, warmer climates and often more acid soils distinguish them from oceanic cool temperate forests (T2.3), while in the subtropics they transition to T1 forests. Autochthonous energy supports relatively high primary productivity, weakly limited by summer drought and sometimes by acid substrates. Forest function is regulated mainly by bottom-up processes related to resource competition rather than top-down trophic processes or disturbance regimes. Trophic structure is simpler than in tropical forests, with moderate levels of diversity and endemism among major taxa (e.g. typically <20 tree spp./ha), but local assemblages of birds, bats, and canopy invertebrates may be abundant and species-rich and play important roles in pollination and seed dispersal. Canopy insects are the major consumers of primary production and a major food source for birds. Decomposers and detritivores such as invertebrates, fungi, and microbes on the forest floor are critical to nutrient cycling. Vertebrate herbivores are relatively uncommon, with low-moderate mammalian diversity. Although epiphytes and lianas are present, plant life-form traits that are typical of tropical forests (T1.1 and T1.2) such as buttress roots, compound leaves, monopodial growth, and cauliflory are uncommon or absent in warm-temperate rainforests. Some trees have ecophysiological tolerance of acid soils (e.g. through aluminium accumulation). Gap-phase dynamics are driven by individual tree-fall and lightning strikes, but many trees are shade-tolerant and recruit slowly in the absence of disturbance. Ground vegetation includes varied growth forms but few grasses.

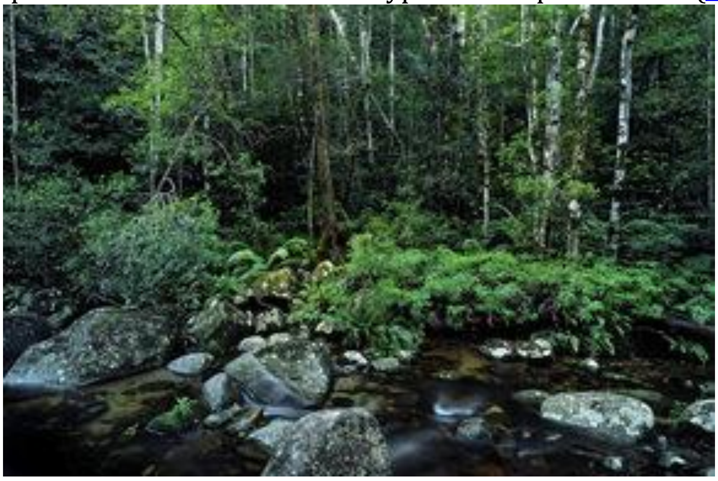

Warm temperate rainforest with Coachwood (Ceratopetalum apetalum), Washpool National Park, New South Wales, Australia.
Credit: Jaime Plaza van Roon / AUSCAPE

Ecological drivers: The environmental niche of these forests is defined by a modest overall water surplus with no distinct dry season, albeit moderate summer water deficits in some years. Mean annual rainfall is typically 1,200–2,500 mm, but topographic mesoclimates (e.g. sheltered gullies and orographic processes) sustain reliable moisture at some sites. Temperatures are mild with moderate seasonality and a growing season of 6–8 months, and mild frosts occur. Substrates may be acidic with high levels of Al and Fe that limit the uptake of nutrients. These forests may be embedded in fire-prone landscapes but are typically not flammable due to their moist microclimates .

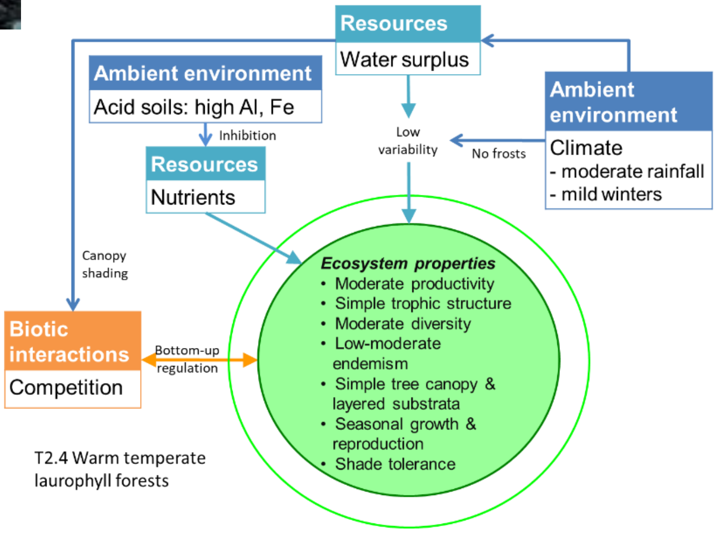

Distribution: Patchy warm temperate-subtropical distribution at 26–43° latitude, north or south of the Equator.

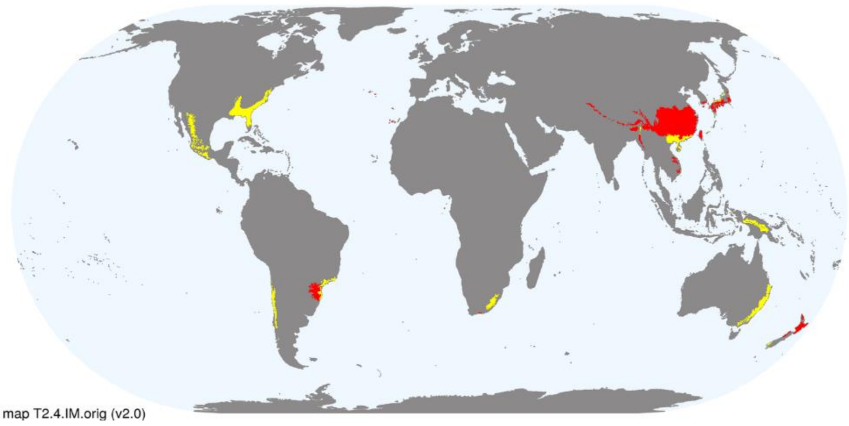

References:

Corlett RT Hughes AC (2015) Subtropical Forests. *Handbook of Forest Ecology* pp. 46-55. Routledge.

Grubb PJ, Bellingham PJ, Kohyama TS, Piper FS Valido A (2013) Disturbance regimes, gap-demanding trees and seed mass related to tree height in warm temperate rain forests worldwide. *Biological Reviews* 88: 701–744.

Mucina L (2018) *Vegetation survey and classification of subtropical forests of southern Africa* Springer International Publishing.

Map is for illustrative purposes only and does not support spatial analyses unless formally validated.

T2.5 Temperate pyric humid forests

Ecosystem properties: This group includes the tallest forests on earth. They are moist, multi-layered forests in wet-temperate climates with complex spatial structure and very high biomass and LAI. The upper layer is an open canopy of sclerophyllous trees 40–90-m tall with long, usually unbranched trunks. The open canopy structure allows light transmission sufficient for the development of up to three subcanopy layers, consisting mostly of non-sclerophyllous trees and shrubs with higher SLA than the upper canopy species. These forests are highly productive, grow rapidly, draw energy from autochthonous sources and store very large quantities of carbon, both above and below ground. They have complex trophic networks with a diverse invertebrate, reptile, bird, and mammal fauna with assemblages that live primarily in the tree canopy or the forest floor, and some that move regularly between vertical strata. Some species are endemic and have traits associated with large trees, including the use of wood cavities, thick or loose bark, large canopies, woody debris, and deep, moist leaf litter. There is significant diversification of avian foraging methods and hence a high functional and taxonomic diversity of birds. High deposition rates of leaf litter and woody debris sustain diverse fungal decomposers and invertebrate detritivores and provide nesting substrates and refuges for ground mammals and avian insectivores. The shade-tolerant ground flora may include a diversity of ferns forbs, grasses (mostly C3), and bryophytes. The dominant trees are shade-intolerant and depend on tree-fall gaps or periodic fires for regeneration. In cooler climates, trees are killed by canopy fires but may survive surface fires, and canopy seedbanks are crucial to persistence. Epicormic resprouting (i.e. from aerial stems) is more common in warmer climates. Subcanopy and ground layers include both shade-tolerant and shade-intolerant plants, the latter with physically and physiologically dormant seedbanks that cue episodes of mass regeneration to fire. Multi-decadal or century-scale canopy fires consume biomass, liberate resources, and trigger life-history processes in a range of biota. Seedbanks sustain plant diversity through storage effects.

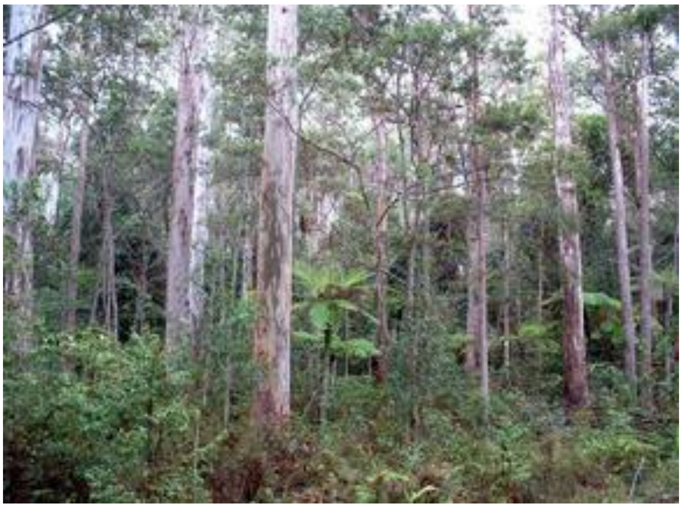

Structurally complex pyric wet forest, Guy Fawkes National Park, Australia.
Credit: Monica Campbell

Ecological drivers: There is an annual water surplus with seasonal variation (peak surplus in winter) and rare major summer deficits associated with inter-annual drought cycles. Multiple tree layers produce a light diminution gradient and moist micro-climates at ground level. Winters are cool and summers are warm with occasional heatwaves that dry out the moist micro-climate and enable periodic fires, which may be extremely intense and consume the canopy. The growing season is 6–8 months. Snow is uncommon and short-lived. Soils are relatively fertile, but often limited in Nitrogen.

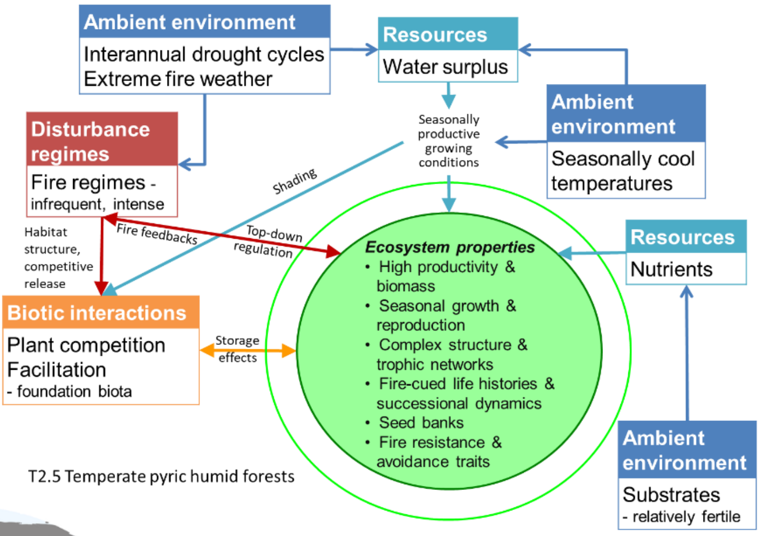

Distribution: Subtropical - temperate southeast and temperate southwest Australia.

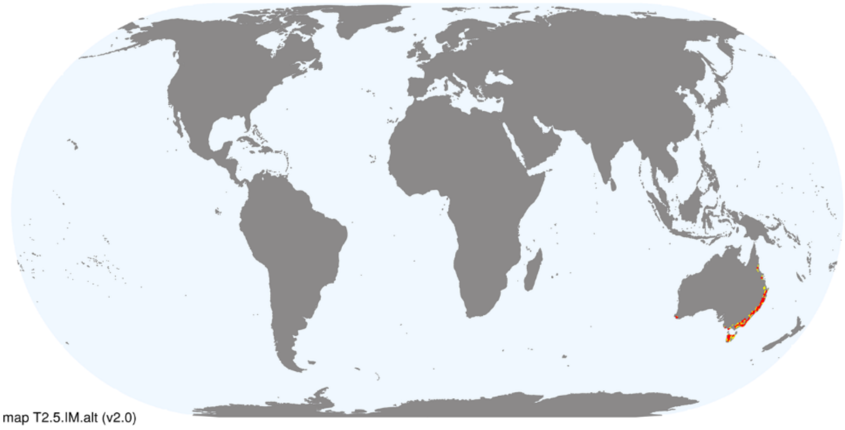

Map is for illustrative purposes only and does not support spatial analyses unless formally validated.

References:

Mac Nally RC (1995) On large-scale dynamics and community structure in forest birds: Lessons from some eucalypt forests of southeastern Australia. *Philosophical Transactions of the Royal Society B* 350B: 369-79.

Wardell-Johnson G, Neldner J, Balmer J (2017) Wet sclerophyll forests. *Australian vegetation* (Ed. DA Keith), pp 281-313. Cambridge University Press, Cambridge.

Contributors: DA Keith, F Essl, J Franklin, R Mac Nally

T2.6 Temperate pyric sclerophyll forests and woodlands

Ecosystem properties: Forests and woodlands, typically 10–30-m tall with an open evergreen sclerophyllous tree canopy and low-moderate LAI grow in fire-prone temperate landscapes. Productivity is lower than other temperate and tropical forest systems, limited by low nutrient availability and summer water deficits. Abundant light and water (except in peak summer) enable the development of substantial biomass with high C:N ratios. Trees have microphyll foliage with low to very low SLA. Sclerophyll or subsclerophyll shrubs with low to very low SLA foliage form a prominent layer between the trees. A sparse ground layer of C3 and C4 tussock grasses and forbs becomes more prominent on soils of loamy texture. Diversity and local endemism may be high among some taxa including plants, birds, and some invertebrates such as dipterans and hemipterans. Low nutrients and summer droughts limit the diversity and abundance of higher trophic levels. Plant traits (e.g. sclerophylly, stomatal invagination, tubers, and seedbanks) confer tolerance to pronounced but variable summer water deficits. Plants possess traits that promote the efficient capture and retention of nutrients, including specialised root structures, N-fixing bacterial associations, slow leaf turnover, and high allocation of photosynthates to structural tissues and exudates. Consumers have traits that enable the consumption of high-fibre biomass. Mammalian herbivores (e.g. the folivorous koala) can exploit high-fibre content and phenolics. Plants and animals have morphological and behavioural traits that allow tolerance or avoidance of fire and the life-history processes of many taxa are cued to fire (especially plant recruitment). Key fire traits in plants include recovery organs protected by thick bark or burial, serotinous seedbanks (i.e. held in plant canopies), physical and physiological seed dormancy and pyrogenic reproduction. Almost all plants are shade-intolerant and fire is a critical top-down regulator of diversity through storage effects and the periodic disruption of plant competition.

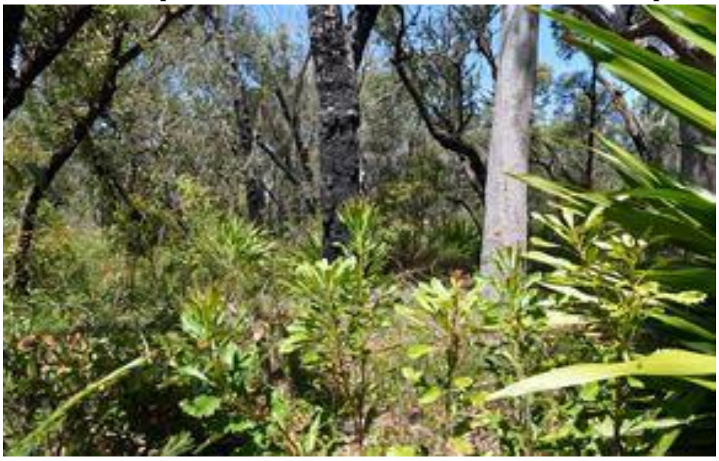

Sclerophyll Forest regenerating after fire, Royal National Park, Australia.
Credit: David Keith

Ecological drivers: Hot summers generate a marked but variable summer water deficit, usually with a modest winter surplus, irrespective of whether rainfall is highly seasonal with winter maximum, aseasonal, or weakly seasonal with inter-annually variable summer maxima. Soils are acidic, sandy, or loamy in texture, and low to very impoverished in P and N. Hot summers define a marked season for canopy or surface fires at decadal to multi-decadal intervals. Light frost occurs periodically in some areas but snow is rare.

Distribution: Temperate regions of Australia, the Mediterranean, and central California.

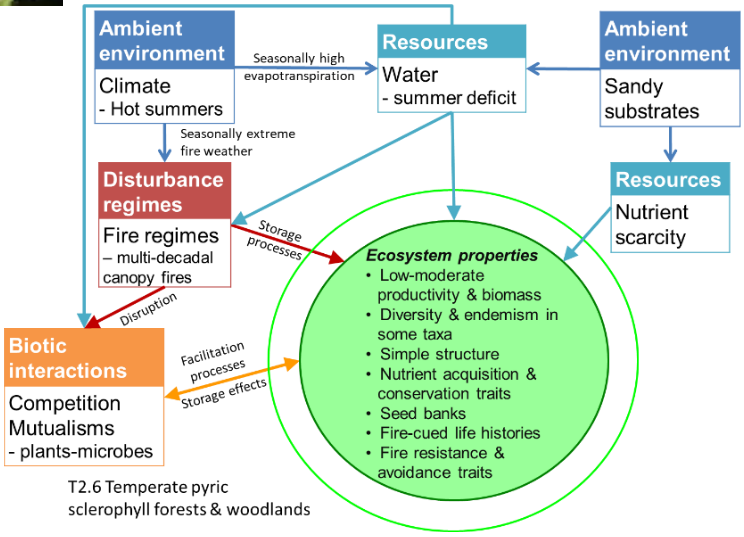

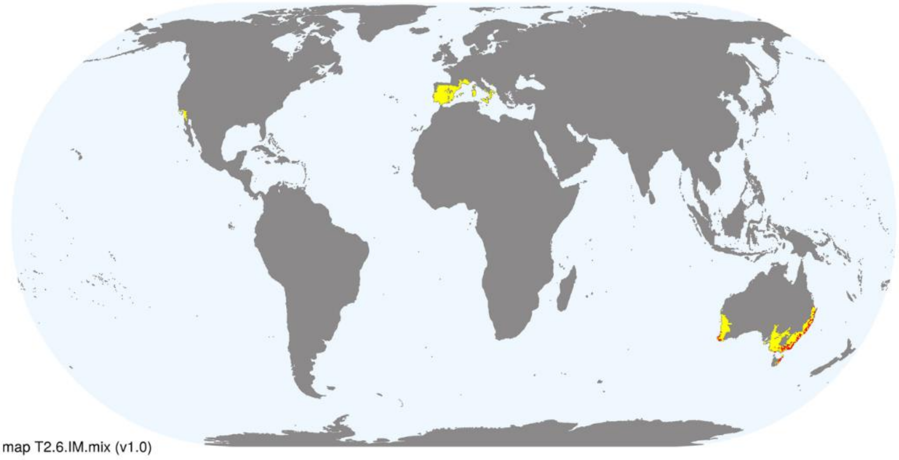

References:

Barbour M, Keeler-Wolf T, Schoenherr AA (2007) *Terrestrial vegetation of California* 3rd edition. University of California Press, Berkeley.

Tozer MG, Simpson CC, Jansens IB, Keith DA (2017) Biogeography of Australia's dry sclerophyll forests: drought, nutrients and fire. *Australian vegetation* (Ed. DA Keith), pp 314-338. Cambridge University Press, Cambridge. ISBN 978-1-107-11843-0.

Map is for illustrative purposes only and does not support spatial analyses unless formally validated.

T3. Shrublands and shrubby woodlands biome

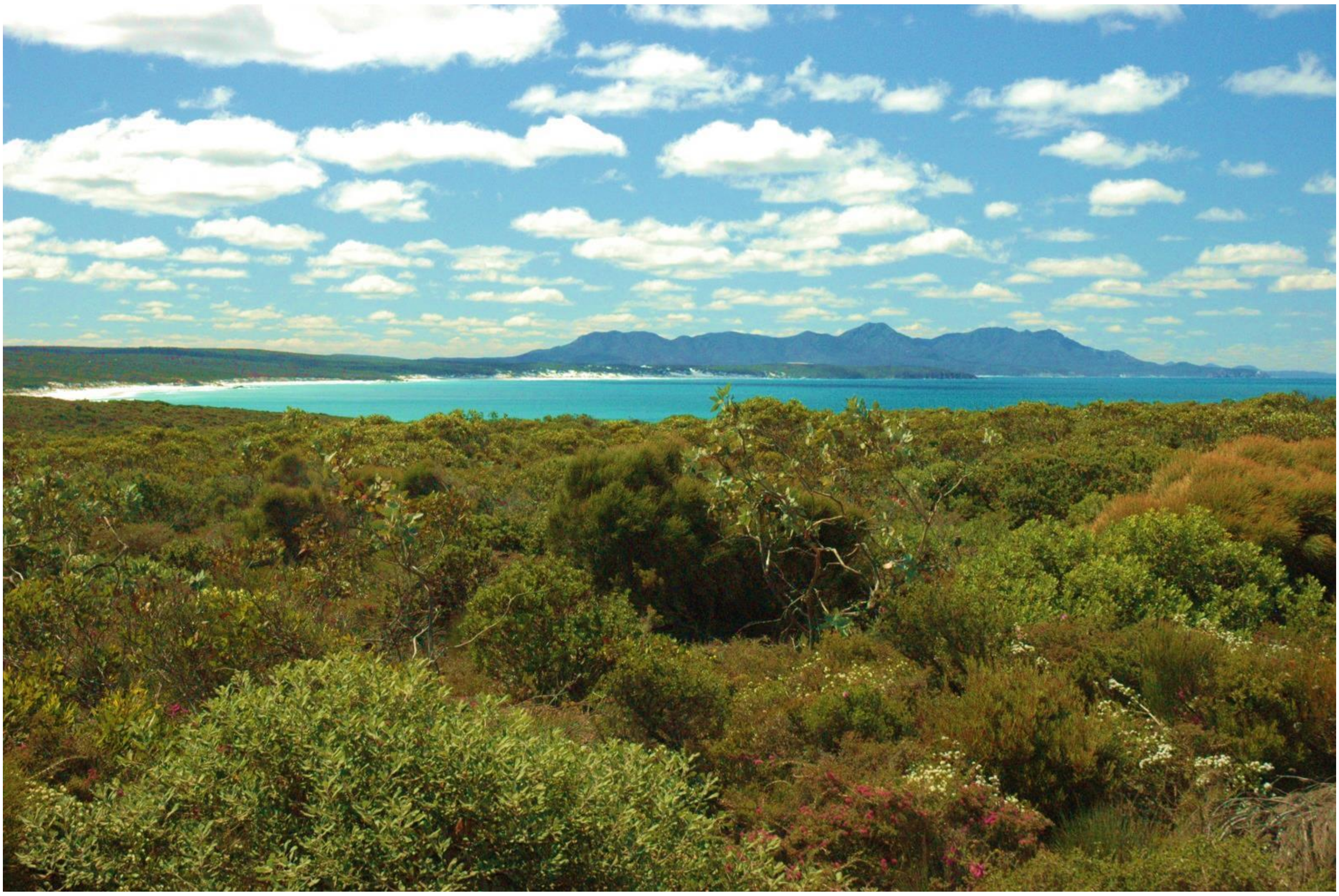

Anna Bay, Western Australia.

Credit: David Keith

The Shrublands and shrub-dominated woodlands biome includes oligotrophic systems occurring on acidic, sandy soils that are often shallow or skeletal. Classically regarded as 'azonal' biomes or 'pedobiomes' (i.e. biomes determined by soils), they are scattered across all landmasses outside the polar regions, generally (but not always) closer to continental margins than to interior regions and absent from central Asia.

Productivity and biomass are low to moderate and limited by soil fertility. The effect of nutrient poverty on productivity is exacerbated in tropical to mid-latitudes by water deficits occurring during either winter (tropics) or summer (temperate humid and Mediterranean climates) and by low insolation and cold temperatures at higher latitudes. Trophic networks are simple but the major functional components (photoautotrophic plants, decomposers, detritivores, herbivores, and predators) are all represented and fuelled by autochthonous energy sources.

Shrubs are the dominant primary producers and possess a diversity of leaf and root traits as well as mutualistic relationships with soil microbes that promote the capture and conservation of nutrients. Recurrent disturbance events exert top-down regulation by consuming biomass, releasing resources, and triggering life-history processes (including recruitment and dispersal) in a range of organisms.

Fire is the most widespread mechanism, with storms or mass movement of substrate less frequently implicated. Storage effects related to re-sprouting organs and seed banks appear to be important for maintaining plant diversity and hence structure and function in shrublands exposed to recurring fires and water deficits.

Contributors: DA Keith, J Russell-Smith

T3.1 Seasonally dry tropical shrublands

Ecosystem properties: These moderate-productivity, mostly evergreen shrublands, shrubby grasslands and low, open forests (generally <6-m tall) are limited by nutritional poverty and strong seasonal drought in the tropical winter months. Taxonomic and functional diversity is moderate in most groups but with high local endemism in plants, invertebrates, birds, and other taxa. Vegetation is spatially heterogeneous in a matrix of savannas (T4.2) or tropical dry forests (T1.2) and dominated by sclerophyllous shrubs with small leaf sizes (nanophyll-microphyll) and low SLA. C4 grasses may be conspicuous or co-dominant (unlike in most temperate heathlands, T3.2) but generally do not form a continuous stratum as in savannas (T4). These systems have relatively simple trophic networks fuelled by autochthonous energy sources. Productivity is low to moderate and constrained by seasonal drought and nutritional poverty. Shrubs are the dominant primary producers and show traits promoting the capture and conservation of nutrients (e.g. sclerophylly, cluster roots, carnivorous structures, and microbial and fungal root mutualisms) and tolerance to severe seasonal droughts (e.g. stomatal invagination). Nectarivorous and/or insectivorous birds and reptiles and granivorous small mammals dominate the vertebrate fauna, but vertebrate herbivores are sparse. Recurring fires play a role in the top-down regulation of ecosystem structure and composition.

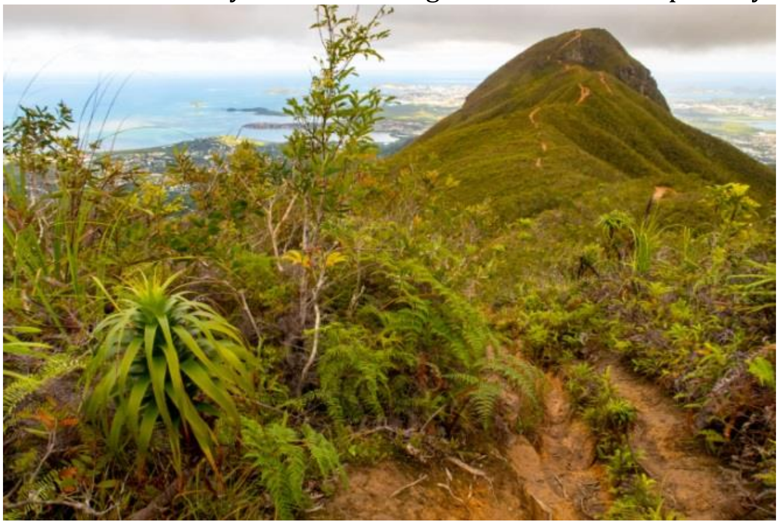

Tropical maquis on serpentinite, Pic Maloui, New Caledonia.
Credit: Oliver Descoeudres

Ecological drivers: A severe seasonal climatic water deficit during tropical winter months is exacerbated by sandy or shallow rocky substrates with low moisture retention. Nutritional poverty (especially N and P) stems from oligotrophic, typically acid substrates such as sandstones, ironstones, leached sand deposits, or rocky volcanic or ultramafic substrates. Vegetation holds the largest pool of nutrients. Temperatures are warm, rarely <10°C, with low diurnal and seasonal variation. Dry-season fires recur on decadal or longer time scales, but they are rare in table-top mountains (tepui).

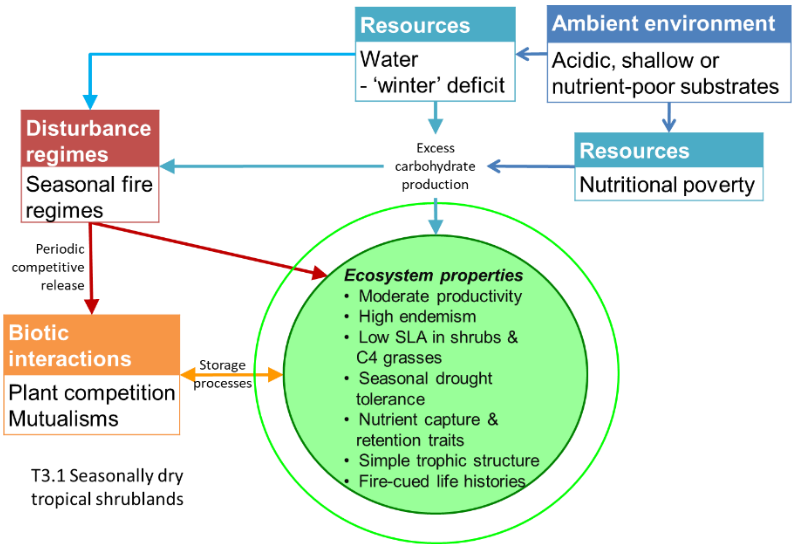

Distribution: Brazilian campos rupestres (where grasses are important), Venezuelan tepui, Peruvian tabletops, Florida sands, and scattered in northern Australia and montane oceanic islands.

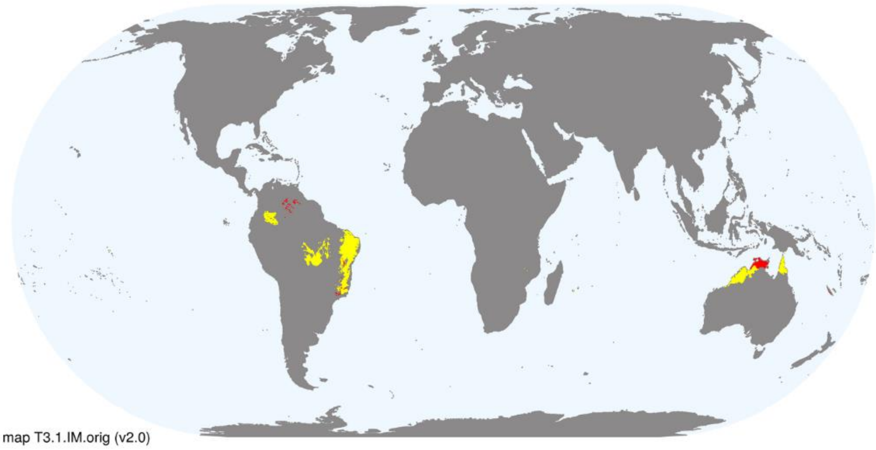

map T3.1.IM.orig (v2.0)

References:

Fernandes GW (2016) *Ecology and conservation of mountaintop grasslands in Brazil* Springer: Berlin.

Morellato LPC, Silveira.FAO (2018) Plant life in campo rupestre: New lessons from an ancient biodiversity hotspot. *Flora* 238: 1-10.

Russell-Smith J, Ryan PG, Klessa D, Waight G, Harwood R (1998) Fire regimes, fire-sensitive vegetation, and fire management of the sandstone Arnhem Plateau, monsoonal northern Australia. *Journal of Applied Ecology* 35, 829-846.

Map is for illustrative purposes only and does not support spatial analyses unless formally validated.

T3.2 Seasonally dry temperate heath and shrublands

Ecosystem properties: Sclerophyllous, evergreen shrublands are distinctive ecosystems of humid and subhumid climates in mid-latitudes. Their low-moderate productivity is fuelled by autochthonous energy sources and is limited by resource constraints and/or recurring disturbance. Vegetation is dominated by shrubs with very low SLA, high C:N ratios, shade-intolerance, and long-lived, small, often ericoid leaves, sometimes with a low, open canopy of sclerophyll trees. The ground layer may include geophytes and sclerophyll graminoids, though less commonly true grasses. Trophic webs are simple, with large mammalian predators scarce or absent, and low densities of vertebrate herbivores. Native browsers may have local effects on vegetation. Diversity and local endemism may be high among vascular plants and invertebrate consumers. Plants and animals have morphological, ecophysiological, and life-history traits that promote persistence under summer droughts, nutrient poverty, and recurring fires, which play a role in top-down regulation. Stomatal regulation and root architecture promote drought tolerance in plants. Cluster roots and acid exudates, mycorrhizae, and insectivory promote nutrient capture, while cellulose, lignin, exudate production, and leaf longevity promote nutrient conservation in plants. Vertebrate herbivores and granivores possess specialised dietary and digestive traits enabling consumption of foliage with low nutrient content and secondary compounds. Slow decomposition rates are slow, allowing litter-fuel accumulation to add to well-aerated fine fuels in shrub canopies. Life-history traits such as recovery organs, serotiny, post-fire seedling recruitment, pyrogenic flowering, and fire-related germination cues promote plant survival, growth, and reproduction under recurring canopy fires. Animals evade fires in burrows or through mobility. Animal pollination syndromes are common (notably dipterans, lepidopterans, birds, and sometimes mammals) and ants may be prominent in seed dispersal.

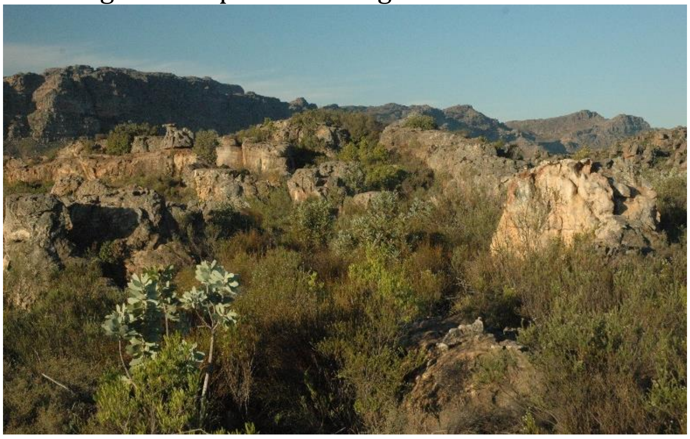

Fynbos, Pakhuispas, Cederberg Mountains, South Africa.
Credit: David Keith

Ecological drivers: A marked summer water deficit and a modest winter surplus is driven by high summer temperatures and evapotranspiration with winter-maximum or aseasonal rainfall patterns. Winters are mild, or cool at high elevations. Sandy soil textures or reverse-texture effects of clay-loams exacerbate an overall water deficit. Soils are typically acid, derived from siliceous sand deposits, sandstones, or acid intrusives or volcanics, and are low to very low in P, N, and mineral cations (though this varies between regions, e.g, base-rich limestones, marl and dolomites in southern Europe). The climate, soils, and vegetation promote summer canopy fires at decadal to multi-decadal intervals. Positive feedbacks between fire and vegetation may be important in maintaining flammability.

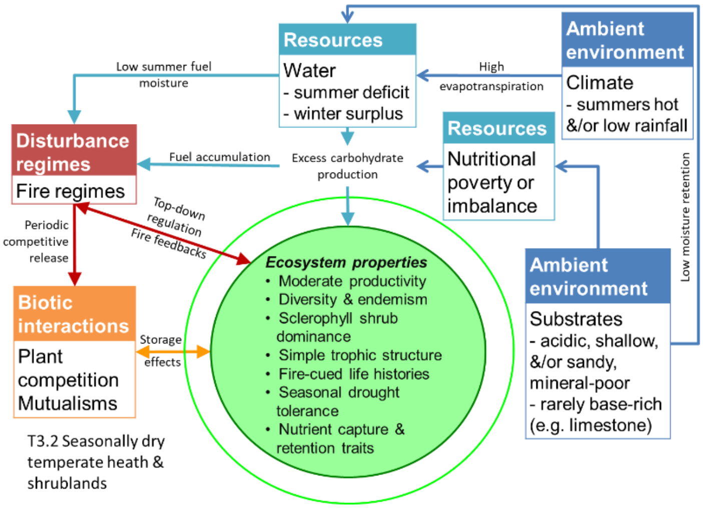

Distribution: Mediterranean-type climate regions of Europe, north and south Africa, southern Australia, western North and South America, and occurrences in non-Mediterranean climates in eastern Australia, the USA, and Argentina.

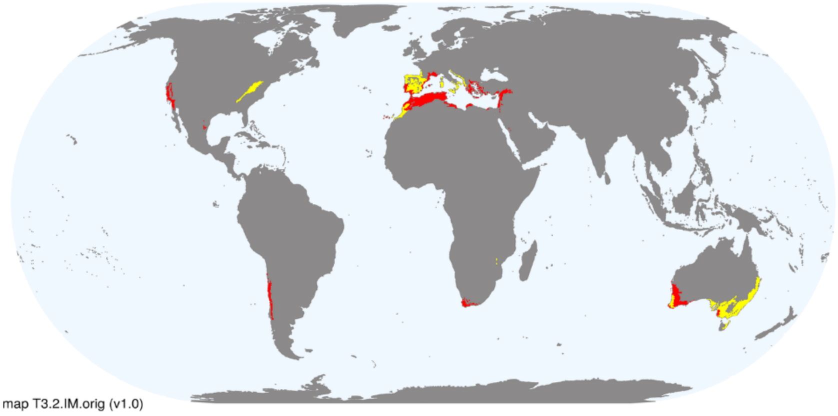

References:

Keeley JE, Bond WJ, Bradstock RA, Pausas, JG, Rundel PW (2012) *Fire in Mediterranean Ecosystems: ecology, evolution and management* Cambridge University Press, Cambridge.

Lamont BB, Keith DA (2017) Heathlands and associated shrublands. *Australian vegetation* (Ed. DA Keith), pp 339-368. Cambridge University Press, Cambridge. ISBN [978-1-107-11843-0](978-1-107-11843-0).

Map is for illustrative purposes only and does not support spatial analyses unless formally validated.

T3.3 Cool temperate heathlands

Ecosystem properties: These mixed graminoid shrublands are restricted to cool-temperate maritime environments. Typically, the vegetation cover is >70% and mostly less than 1-m tall, dominated by low, semi-sclerophyllous shrubs with ferns and C3 graminoids. Shrub foliage is mostly evergreen and ericoid, with low SLA or reduced to spiny stems. Modular growth forms are common among shrubs and grasses. Diversity and local endemism are low across taxa and the trophic network is relatively simple. Primary productivity is low, based on autochthonous energy sources and limited by cold temperatures and low-fertility acid soils rather than by water deficit (as in other heathlands, T3). Seasonally low light may limit productivity at the highest latitudes. Cool temperatures and low soil oxygen due to periodically wet subsoil limit decomposition by microbes and fungi so that soils accumulate organic matter despite low productivity. Mammalian browsers including cervids, lagomorphs, and camelids (South America) consume local plant biomass but subsidise autochthonous energy with carbon and nutrients consumed in more productive forest or anthropogenic ecosystems adjacent to the heathlands. Browsers and recurring low-intensity fires appear to be important in top-down regulatory processes that prevent the transition to forest, as is anthropogenic fire, grazing, and tree removal. Canids and raptors are the main vertebrate predators. Other characteristic vertebrate fauna include ground-nesting birds and rodents. At least some communities exhibit autogenic cyclical patch dynamics in which shrubs and grasses are alternately dominant, senescent, and regenerating.

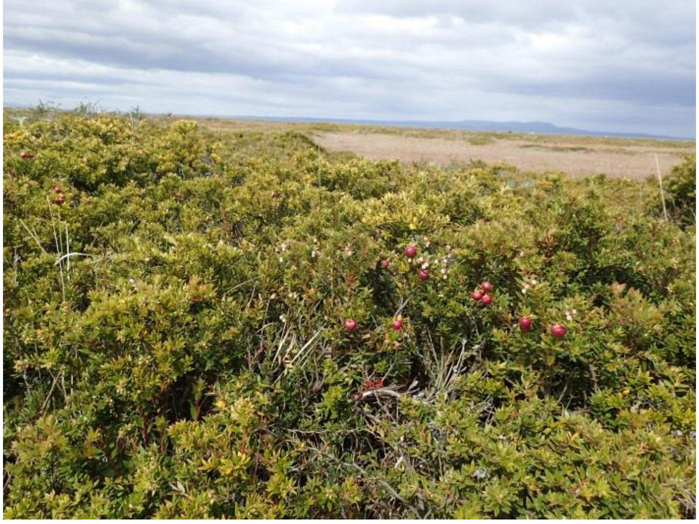

Magellanic heath, Patagonia, Chile.
Credit: David Keith

Ecological drivers: Unlike most other heathlands, these ecosystems have an overall water surplus, though sometimes with small summer deficits. Mild summers and cold winters with periodic snow are tempered by maritime climatic influences. A short day length and low solar angle limits energy influx at the highest latitudes. Severe coastal storms with high winds occur periodically. Acid soils, typically with high humic content in upper horizons, are often limited in N and P. Low-intensity fires recur at decadal time scales or rarely. Some northern European heaths were derived from forest and return to forest when burning and grazing ceases.

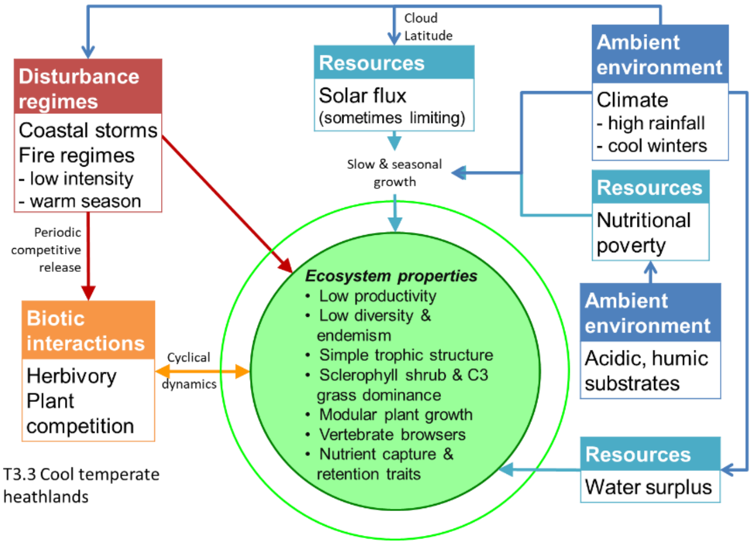

Distribution: Boreal and cool temperate coasts of western Europe and America, the Azores, and the Magellanic region of South America, mostly at >40° latitude, except where transitional with warm-temperate heaths (e.g. France and Spain).

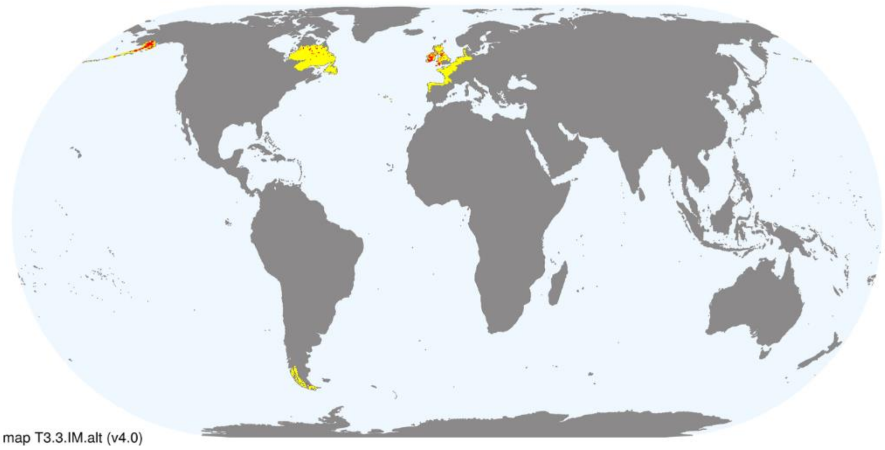

map T3.3.IM.alt (v4.0)

References:

Aerts R, Heil GW (1993) *Heathlands: patterns and processes in a changing environment* Kluwer Academic Publishing.

Loidi J, Biurrun I, Juan Antonio Campos JA, García-Mijangos I, Herrera M (2010) A biogeographical analysis of the European Atlantic lowland heathlands. *Journal of Vegetation Science* 21(5): 832–842.

Watt AS (1947) Pattern and process in the plant community. *Journal of Ecology* 35, 1-22.

Map is for illustrative purposes only and does not support spatial analyses unless formally validated.

Contributors: DA Keith, NA Cutler

T3.4 Young rocky pavements, lava flows and screes

Ecosystem properties: Vegetation dominated by cryptogams (lichens, bryophytes) develops on skeletal rocky substrates and may have scattered shrubs with very low LAI. These low-productivity systems are limited by moisture and nutrient scarcity, temperature extremes, and periodic disturbance through mass movement. Diversity and endemism is low across taxa and the trophic structure is simple. Reptiles and ground-nesting birds are among the few resident vertebrates. Lichens and bryophytes may be abundant and perform critical roles in moisture retention, nutrient acquisition, energy capture, surface stabilisation, and proto-soil development, especially through carbon accumulation. N-fixing lichens and cyanobacteria, nurse plants, and other mutualisms are critical to ecosystem development. Rates of ecosystem development are linked to substrate weathering, decomposition, and soil development, which mediate nutrient supply, moisture retention, and temperature amelioration. Vascular plants have nanophyll-microphyll leaves and low SLA. Their cover is sparse and comprises ruderal pioneer species (shrubs, grasses, and forbs) that colonise exposed surfaces and extract moisture from rock crevices. Species composition and vegetation structure are dynamic in response to surface instability and show limited differentiation across environmental gradients and microsites due to successional development, episodes of desiccation, and periodic disturbances that destroy biomass. Rates of vegetation development, soil accumulation, and compositional change display amplified temperature-dependence due to resource-concentration effects. Older rocky systems have greater micro-habitat diversity, more insular biota, and higher endemism and are classified in other functional groups.

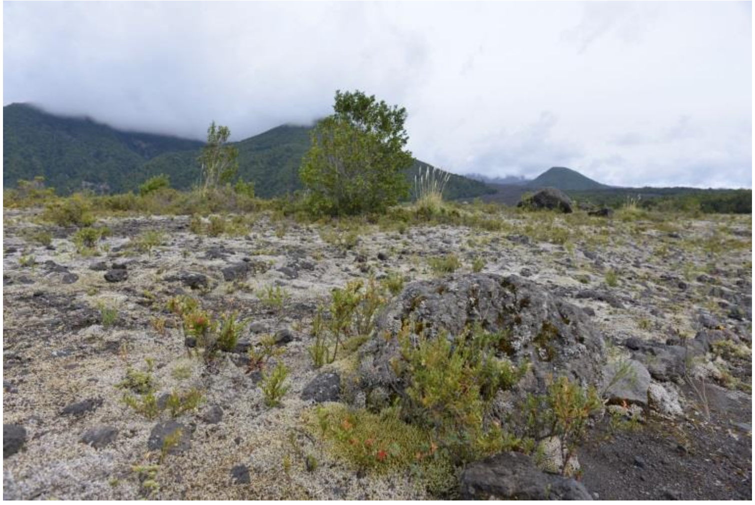

Lava flow, Conguillo National Park, Chile.
Credit: David Keith

Ecological drivers: Skeletal substrates (e.g. lava pavements, scree slopes, and rock outcrops) limit water retention and nutrient capital and increase heat absorption, leading to periodically extreme temperatures. High summer temperatures and solar exposure concentrate resources and increase the temperature-sensitivity of biogeochemical processes. Winter temperatures may be cold at high elevations (see T6.2). Recurring geophysical disturbances such as lava flow, mass movement, and geothermal activity as well as desiccation episodes periodically destroy biomass and reset successional pathways.

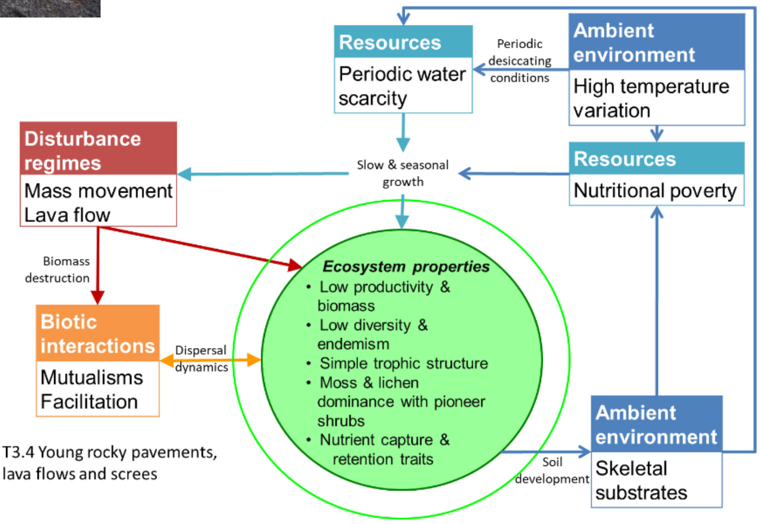

Distribution: Localised areas scattered around the Pacific Rim, African Rift Valley, Mediterranean and north Atlantic.

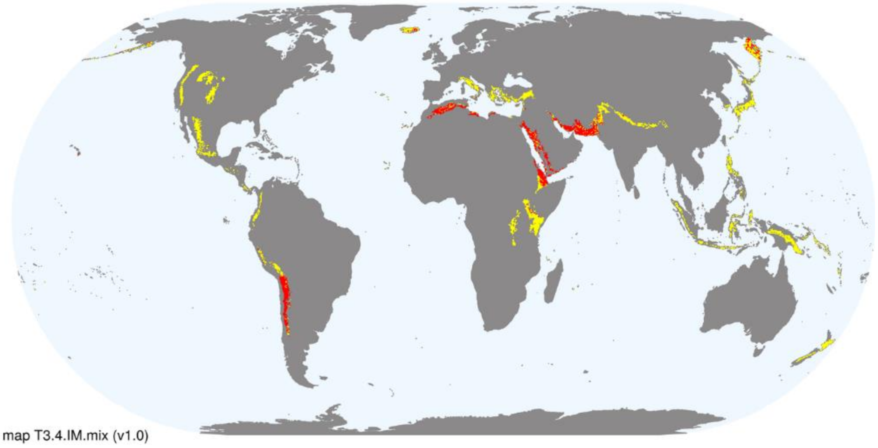

map T3.4.IM.mix (v1.0)

Map is for illustrative purposes only and does not support spatial analyses unless formally validated.

References:

Anderson-Teixeira KJ, Vitousek PM, Brown JH (2007) Amplified temperature dependence in ecosystems developing on the lava flows of Mauna Loa, Hawai'i. *PNAS* 105: 228-233.

Crews TE, Kurina, LM, Vitousek PM (2001) Organic matter and nitrogen accumulation and nitrogen fixation during early ecosystem development in Hawaii. *Biogeochemistry* 52: 259-279.

Cutler NA, Belyea LR, Dugmore AJ (2008) The spatiotemporal dynamics of a primary succession. *Journal of Ecology* 96: 231-246.

T4. Savannas and grasslands biome

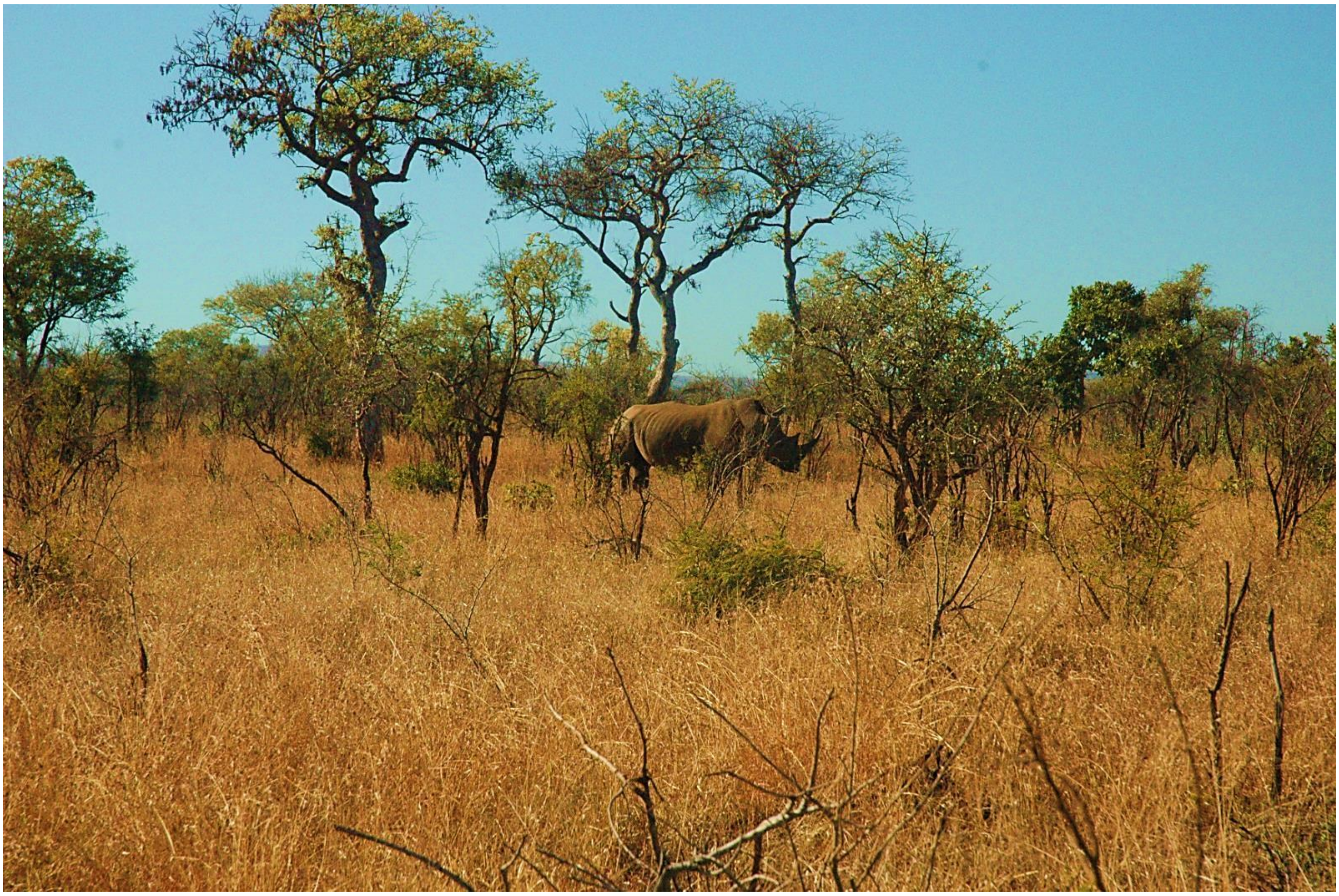

Letaba, Kruger National Park, South Africa.

Credit: David Keith

Ecological functions within the Savannas and grasslands biome are closely linked to a mostly continuous ground layer of grasses that contribute moderate to very high levels of primary productivity driven by strongly seasonal water surplus and deficit cycles.

The timing of the seasonal cycle of productivity varies with latitude and becomes more variable inter-annually as total rainfall declines. The woody component of the vegetation may be completely absent or may vary to a height and stature that resembles that of a forest. In the tropics and subtropics, productivity peaks in the summer when high rainfall coincides with warm temperatures. At temperate latitudes, summer growth is suppressed by water deficits associated with high evapotranspiration, sometimes exacerbated by weakly seasonal (winter-maximum) rainfall, so that productivity peaks in spring when warming temperatures coincide with high soil moisture accumulated over winter.

Co-existence between trees and grasses and between grasses and interstitial forbs is mediated by herbivory and/or fire. These agents are critical in the top-down regulation of grassy ecosystems and in some cases are involved in feedback mechanisms that mediate regime shifts between alternative stable states. Herbivory is the primary driver in highly fertile and productive systems, whereas fire is the primary driver in less fertile and lower productivity systems. Nutrient gradients are exacerbated volatilisation during fire and the loss of nutrients in smoke.

The representation of grass species with C3 and C4 photosynthetic pathways varies with water availability and temperature over regional and continental climatic gradients. Grasses are rapid responders to seasonal pulses of elevated soil moisture and sustain a complex trophic web with large-bodied mammalian herbivores and their predators. The seasonal drying of grasses is critical to flammability. Mammal diversity, trophic complexity, and the expression of physical and chemical defences against herbivory also vary with soil fertility.

T4.1 Trophic savannas

Ecosystem properties: These grassy woodlands and grasslands are dominated by C4 grasses with stoloniferous, rhizomatous and tussock growth forms that are kept short by vertebrate grazers. Trophic savannas (relative to pyric savannas, T4.2) have unique plant and animal diversity within a complex trophic structure dominated by abundant mammalian herbivores and predators. These animals are functionally differentiated in body size, mouth morphology, diet, and behaviour. They promote fine-scale vegetation heterogeneity and dominance of short grass species, sustaining the system through positive feedbacks and limiting fire fuels. Trees and grasses possess functional traits that promote tolerance to chronic herbivory as well as seasonal drought. Seasonal high productivity coincides with summer rains. The dry season induces grass drying and leaf fall in deciduous and semi-deciduous woody plants. Trees are shade-intolerant during their establishment and most develop chemical (e.g. phenolics) or physical (e.g. spinescence) herbivory defence traits and an ability to re-sprout as they enter the juvenile phase. Their soft microphyll-notophyll foliage has relatively high SLA and low C:N ratios, as do grasses. Robust root systems and stolons/rhizomes enable characteristic grasses to survive and spread under heavy grazing. As well as vertebrate herbivores and predators, vertebrate scavengers and invertebrate detritivores are key components of the trophic network and carbon cycle. Nitrogen fixation, recycling, and deposition by animals exceeds volatilisation.

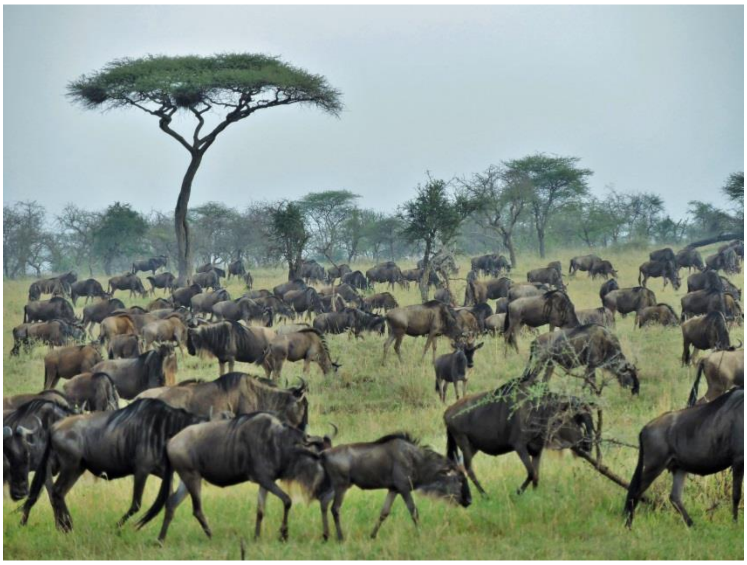

Wildebeest savanna migration, Seronera, Tanzania.
Credit: Lennart Van Den Berg / EyeEm / Getty Images

Ecological drivers: Trophic savannas like pyric savannas are driven by seasonal climates but generally occupy environmental niches with lower rainfall and higher soil fertility. High annual rainfall deficit of 400 mm to >1,800 mm. Annual rainfall generally varies from 300 mm to 700 mm, always with strong seasonal (winter) drought, but these savanna types are restricted to landscapes with sufficient water bodies (rivers and lakes) to sustain high densities of large mammals. Temperatures are warm-hot with low-moderate variability through the year. Low intensity fires have return intervals of 5–50 years, depending on animal densities and inter-annual rainfall variation, usually after the growing season, removing much of the remaining biomass not consumed by herbivores. Soils are moderately fertile and often have a significant clay component.

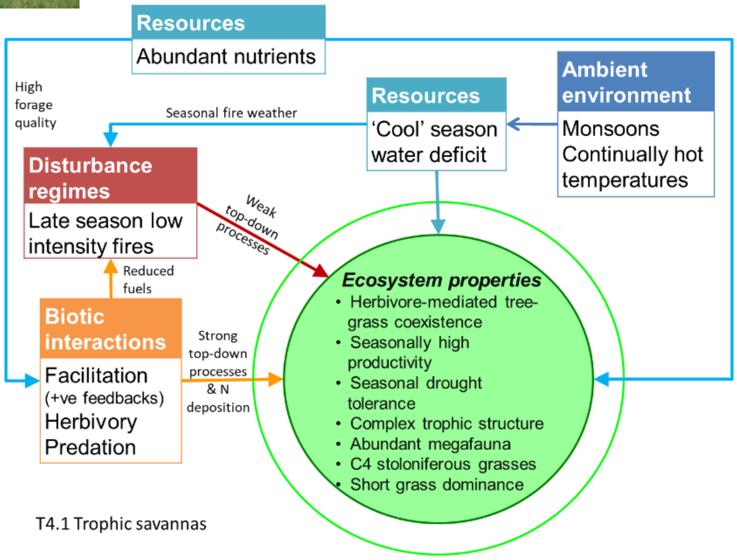

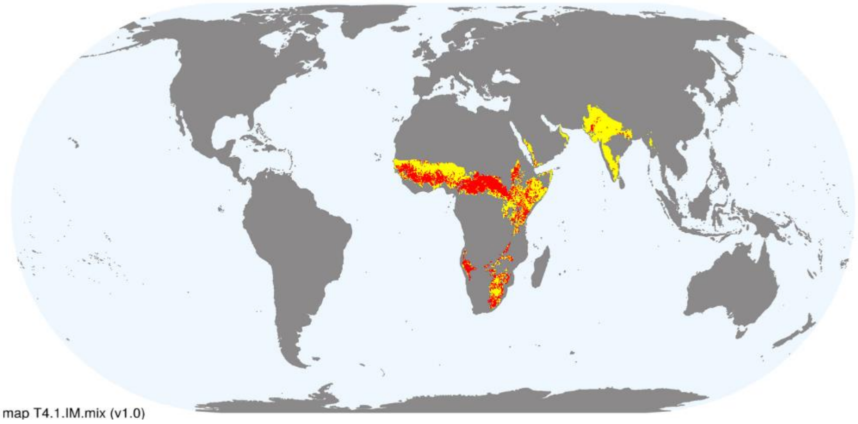

map T4.1.IM.mix (v1.0)

Distribution: Seasonal tropics and subtropics of Africa and Asia.

References:

Archibald S, Hempson GP (2016) Competing consumers: contrasting the patterns and impacts of fire and mammalian herbivory in Africa. *Phil Trans R Soc B* 371: 20150309.

Hempson GP, Archibald S, Bond WJ, Ellis RP, Grant CC, Kruger FJ, et al. (2015) Ecology of grazing lawns in Africa. *Biological Reviews* 90: 979–994.

Map is for illustrative purposes only and does not support spatial analyses unless formally validated.

T4.2 Pyric tussock savannas

Ecosystem properties: Grassy woodlands and grasslands are dominated by C4 tussock grasses, with some C3 grasses in the Americas and variable tree cover. In the tropics, seasonally high productivity coincides with the timing of summer rains and grasses cure in dry winters, promoting flammability. This pattern also occurs in the subtropics but transitions occur with temperate woodlands (T4.4), which have different seasonal phenology, tree and grass dominance, and fire regimes. Tree basal area, abundance of plants with annual semelparous life cycles and abundant grasses with tall tussock growth forms are strongly dependent on mean annual rainfall (i.e. limited by seasonal drought). Local endemism is low across all taxa but regional endemism is high, especially in the Americas and Australasia. Plant traits such as deciduous leaf phenology or deep roots promote tolerance to seasonal drought and rapid resource exploitation. Woody plants have microphyll-notophyll foliage with moderate-high SLA and mostly high C:N ratios. Some C4 grasses nonetheless accumulate high levels of rubisco, which may push down C:N ratios. Nitrogen volatilisation exceeds deposition because fire is the major consumer of biomass. Woody plant species are shade-intolerant during their establishment and develop fire-resistant organs (e.g. thick bark and below-ground bud banks). The contiguous ground layer of erect tussock grasses creates an aerated flammable fuel bed, while grass architecture with tightly clustered culms vent heat away from meristems. Patchy fires promote landscape-scale vegetation heterogeneity (e.g. in tree cover) and maintain the dominance of flammable tussock grasses over shrubs, especially in wetter climates, and hence sustain the system through positive feedbacks. Fires also enhance efficiency of predators. Vertebrate scavengers and invertebrate detritivores are key components of the trophic network and carbon cycle. Mammalian herbivores and predators are present but exert less top-down influence on the diverse trophic structure than fire. Consequently, plant physical defences against herbivores, such as spinescence are less prominent than in T4.1.

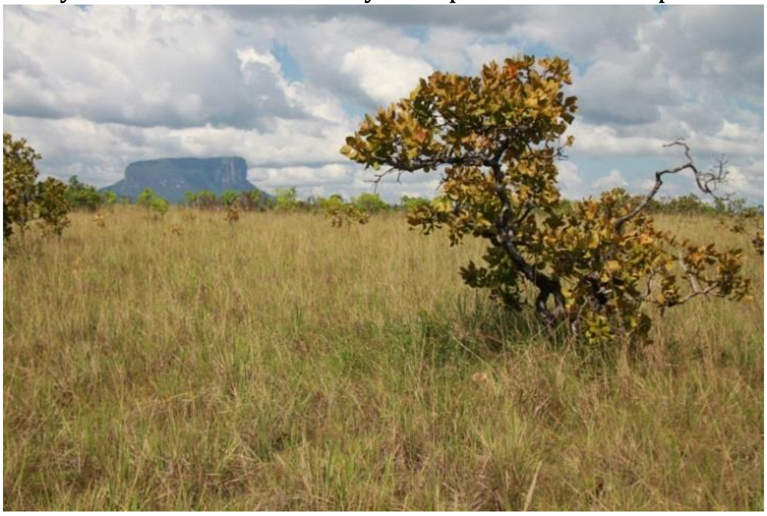

Gran Sabana near Rio Carrao, Venezuela.
Credit: David Keith

Ecological drivers: An overall rainfall deficit up to ~1,200 mm or a modest surplus of up to 500 mm, always with strong seasonal (winter) drought with continuously warm-hot temperatures through the year, even though rainfall becomes less seasonal in the subtropics. Mean annual rainfall varies from 650 mm to 1,500 mm. Sub-decadal fire regimes of surface fires occur throughout the dry season, while canopy fires occur rarely, late in the dry season. Soils are of low-moderate fertility, often with high Fe and Al.

Distribution: Seasonally dry tropics and subtropics of the Americas, Australia, Asia, and Africa.

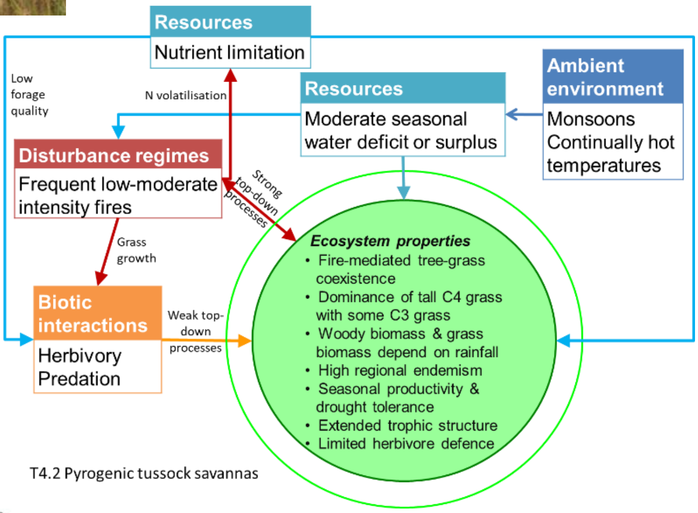

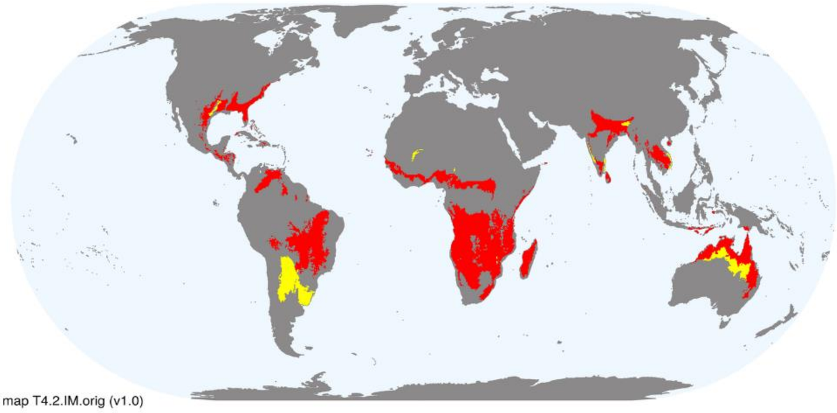

map T4.2.IM.orig (v1.0)

References:

Furley PA (1999) The nature and diversity of neotropical savanna vegetation with particular reference to the Brazilian cerrados. *Global Ecology and Biogeography* 8, 223-241.

Lehmann CER, Anderson TM, Sankaran M, Lehmann CE, Anderson TM, Sankaran M, Higgins SI, Archibald S, Hoffmann WA, Hanan NP, Williams RJ, Fensham RJ, Felfili J, Hutley LB (2014) Savanna vegetation-fire-climate relationships differ among continents. *Science* 343, 548–552.

Map is for illustrative purposes only and does not support spatial analyses unless formally validated.

Contributors: DA Keith, RJ Williams

T4.3 Hummock savannas

Ecosystem properties: These open woodlands are dominated by C4 hummock grasses (C3 and stoloniferous grasses are absent) with sclerophyllous trees and shrubs. Their primary productivity is lower and less regularly seasonal than in other savannas of the subtropics (T4.1 and T4.2), but the seasonal peak nonetheless coincides with summer monsoonal rains. Plant traits promote tolerance to seasonal drought, including reduced leaf surfaces, thick cuticles, sunken stomata, and deep root architecture to access subsoil moisture. Deciduous leaf phenology is less common than in other savannas, likely due to selection pressure for nutrient conservation associated with oligotrophic substrates. A major feature distinguishing this group of savannas from others is its ground layer of slow-growing sclerophyllous, spiny, domed hummock grasses interspersed with bare ground. Woody biomass and LAI decline along rainfall gradients. Sclerophyll shrubs and trees are shade-intolerant during establishment and most possess fire-resistant organs (e.g. thick bark, epicormic meristematic tissues, and below-ground bud banks). Their notophyll foliage and that of hummock grasses have low SLA and mostly high C:N ratios, although N may be elevated in rubisco-enriched C4 grasses. Trophic structure is therefore simpler than in other savannas. Mammalian herbivores and their predators are present in low densities, but fire and invertebrates are the major biomass consumers. Fire promotes landscape-scale vegetation heterogeneity but occurs less frequently than in other savannas due to slow recovery of perennial hummock grass fuels. Nitrogen volatilisation exceeds deposition due to recurring fires.

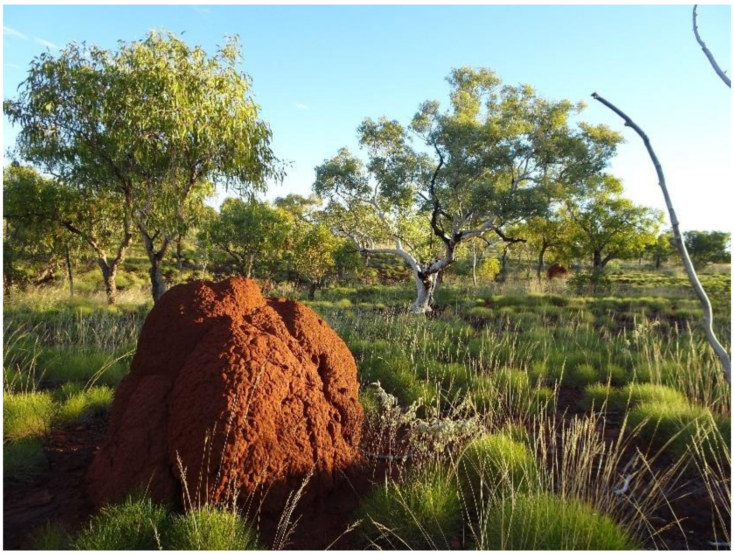

Savanna with eucalypts, hummock grass and termite mounds, Karatjini NP, Australia.
Credit: David Keith

Ecological drivers: Large overall rainfall deficit up to ~2,000 mm, always with a seasonal (winter) drought, but in drier areas seasonality is weaker than in other savanna groups. Mean annual rainfall is generally 400–1,000 mm. Climatic water deficit is exacerbated by coarse-textured, usually shallow, rocky soils. These are characteristically infertile. Temperatures are warm-hot with moderate seasonal and diurnal variability. Fires promoted by flammable hummocks may consume the low tree canopies and occur at variable decadal intervals any time when it is dry, but fire spread depends on ground fuel continuity which is limited by rainfall and rocky terrain.

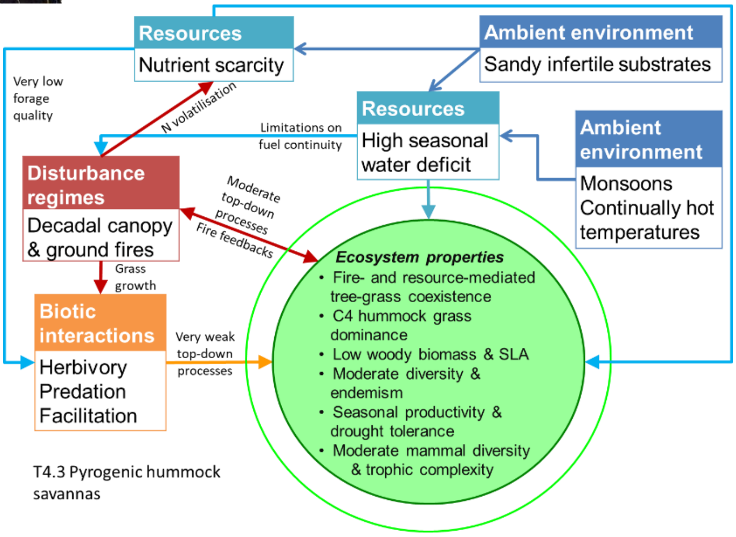

Distribution: Rocky areas of the seasonal Australian tropics, extending to the semi-arid zone.

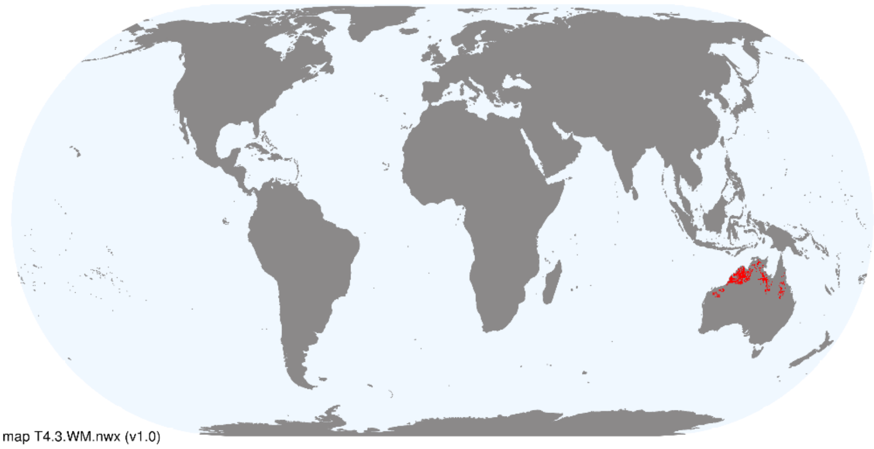

References:

Lehmann CER, Anderson TM, Sankaran M, Lehmann CE, Anderson TM, Sankaran M, Higgins SI, Archibald S, Hoffmann WA, Hanan NP, Williams RJ, Fensham RJ, Felfili J, Hutley LB (2014) Savanna vegetation-fire-climate relationships differ among continents. *Science* 343, 548–552.

Williams RJ, Cook GD, Liedloff AC, Bond WJ (2017) Australia's tropical savannas: vast ancient and rich landscapes. *Australian vegetation* (Ed. DA Keith), pp 368-388. Cambridge University Press, Cambridge.

Map is for illustrative purposes only and does not support spatial analyses unless formally validated.

T4.4 Temperate woodlands

Ecosystem properties: These structurally simple woodlands are characterised by space between open tree crowns and a ground layer with tussock grasses, interstitial forbs, and a variable shrub component. Grasses with C3 and C4 photosynthetic pathways are common, but C4 grasses may be absent from the coldest and wettest sites or where rain rarely falls in the summer. In any given area, C4 grasses are most abundant in summer or on dry sites or areas with summer-dominant rainfall, while C3 grasses predominate in winter, locally moist sites, cold sites, or areas without summer rainfall. The ground flora also varies inter-annually depending on rainfall. Trees generate spatial heterogeneity in light, water, and nutrients, which underpin a diversity of microhabitats and mediate competitive interactions among plants in the ground layer. Foliage is mostly microphyll and evergreen (but transmitting abundant light) or deciduous in colder climates. Diversity of plant and invertebrate groups may therefore be relatively high at local scales, but local endemism is limited due to long-distance dispersal. Productivity is relatively high as grasses rapidly produce biomass rich in N and other nutrients after rains. This sustains a relatively complex trophic network of invertebrate and vertebrate consumers. Large herbivores and their predators are important top-down regulators. Bioturbation by fossorial mammals influences soil structure, water infiltration, and nutrient cycling. The fauna is less functionally and taxonomically diverse than in most tropical savannas (T4.1 and T4.2), but includes large and small mammals, reptiles, and a high diversity of birds and macro-invertebrates, including grasshoppers, which are major consumers of biomass. Plants and animals tolerate and persist through periodic ground fires that consume cured-grass fuels, but few have specialised traits cued to fire (cf. pyric ecosystems such as T2.6).

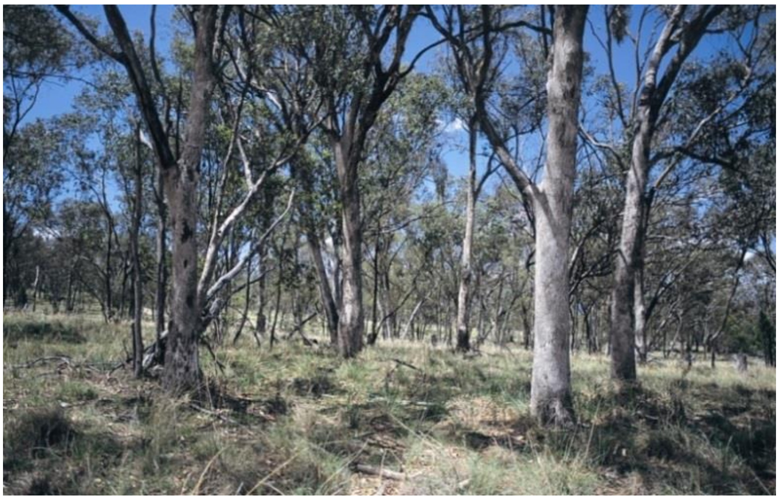

Temperate grassy woodland, Tamworth, Australia.
Credit: David Keith

Ecological drivers: A water deficit occurs seasonally in summer, driven primarily by peak evapotranspiration under warm-hot temperatures and, in some regions, seasonal (winter-maximum) rainfall patterns. Mean annual rainfall is 350–1,000 mm. Low winter temperatures and occasional frost and snow may limit the growing season to 6–9 months. Soils are usually fine-textured and fertile, but N may be limiting in some areas. Fires burn mostly in the ground layers during the drier summer months at decadal intervals.

Distribution: Temperate southeast and southwest Australia, Patagonia and Pampas of South America, western and eastern North America, the Mediterranean region, and temperate Eurasia.

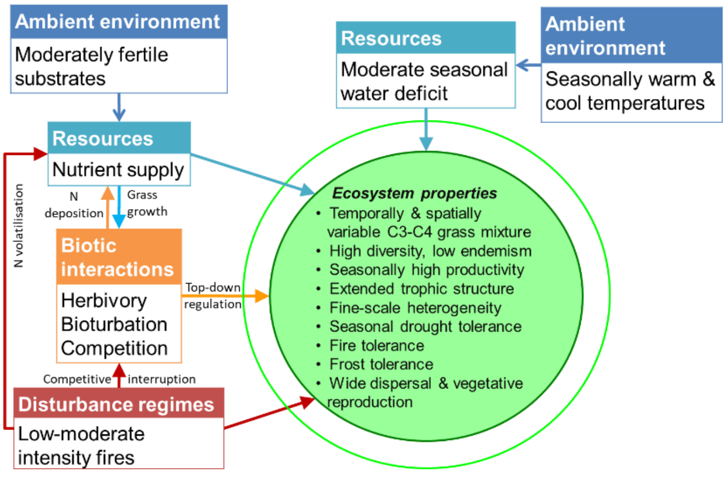

T4.4 Temperate woodlands

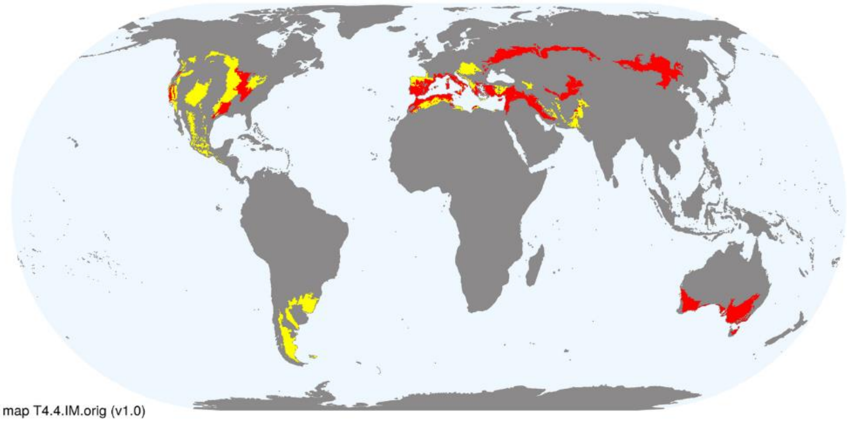

map T4.4.IM.orig (v1.0)

References:

Davis FW, Baldocchi DD, Tyler CM (2019) Oak woodlands. *Ecosystems of California* (Eds. HA Mooney, E Zavaleta). Chapter 25 pp 509-534. University of California Press, Berkeley.

Gibson GJ (2009) *Grasses and grassland ecology* Oxford University Press, Oxford.

Prober SM, Gosper CR, Gilfedder L, Harwood TD, Thiele KR, Williams KJ, Yates CJ (2017) Temperate eucalypt woodlands. *Australian vegetation* (Ed. DA Keith), pp 410-437. Cambridge University Press, Cambridge.

Map is for illustrative purposes only and does not support spatial analyses unless formally validated.

T4.5 Temperate tussock grasslands

Ecosystem properties: Structurally simple tussock grasslands with interstitial forbs occur in subhumid temperate climates. Isolated trees or shrubs may be present in very low densities, but are generally excluded by heavy soil texture, summer drought, winter frost, or recurring summer fires. Unlike tropical savannas (T4.1–T4.3), these systems are characterised by a mixture of both C3 and C4 grasses, with C4 grasses most abundant in summer or on dry sites and C3 grasses predominating in winter or locally moist sites. There are also strong latitudinal gradients, with C3 grasses more dominant towards the poles. Diversity of plant and invertebrate groups may be high at small spatial scales, but local endemism is limited due to long-distance dispersal. Productivity is high as grasses rapidly produce biomass rich in N and other nutrients after rains. This sustains a complex trophic network in which large herbivores and their predators are important top-down regulators. Fossorial mammals are important in bioturbation and nutrient cycling. Mammals are less functionally and taxonomically diverse than in most savannas. Taxonomic affinities vary among regions (e.g. ungulates, cervids, macropods, and camelids), but their life history and dietary traits are convergent. Where grazing is not intense and fire occurs infrequently, leaf litter accumulates from the tussocks, creating a thatch that is important habitat for ground-nesting birds, small mammals, reptiles, and macro-invertebrates, including grasshoppers, which are major consumers of plant biomass. Dense thatch limits productivity. Plant competition plays a major role in structuring the ecosystem and its dynamics, with evidence that it is mediated by resource ratios and stress gradients, herbivory, and fire regimes. Large herbivores and fires both interrupt competition and promote coexistence of tussocks and interstitial forbs.

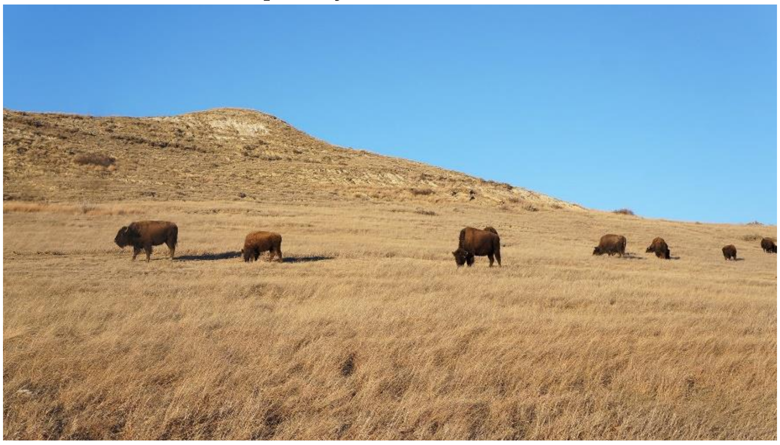

Herd of bison, Badlands Overlook, North Dakota USA.
Credit: indielista / Shutterstock

Ecological drivers: A strong seasonal water deficit in summer driven by peak evapotranspiration under warm-hot temperatures, despite an unseasonal or weakly seasonal rainfall pattern. Mean annual rainfall varies from 250 mm to 750 mm. Cold winter temperatures limit the growing season to 5–7 months, with frost and snow frequent in continental locations. Summers are warm. Soils are deep, fertile and organic and usually fine-textured. Fires ignited by lightning occur in the drier summer months at sub-decadal or decadal intervals.

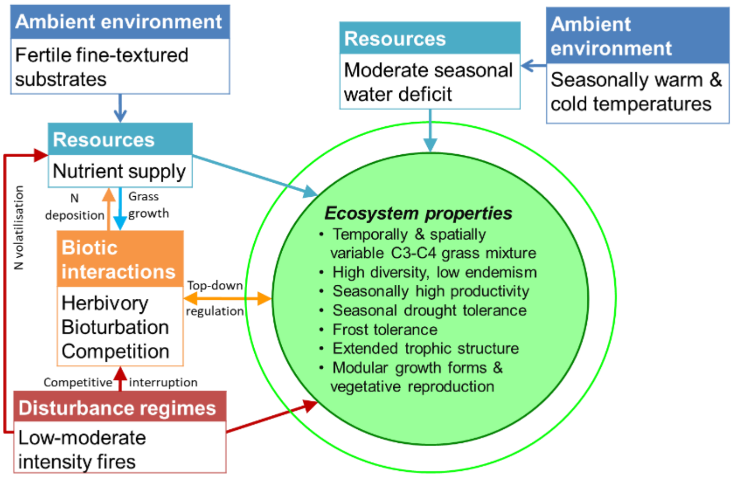

T4.5 Temperate subhumid semi-arid grasslands

Distribution: Subhumid and semi-arid regions of western Eurasia, northeast Asia, Midwest North America, Patagonia and Pampas regions of South America, southeast Africa, southeast Australia, and southern New Zealand.

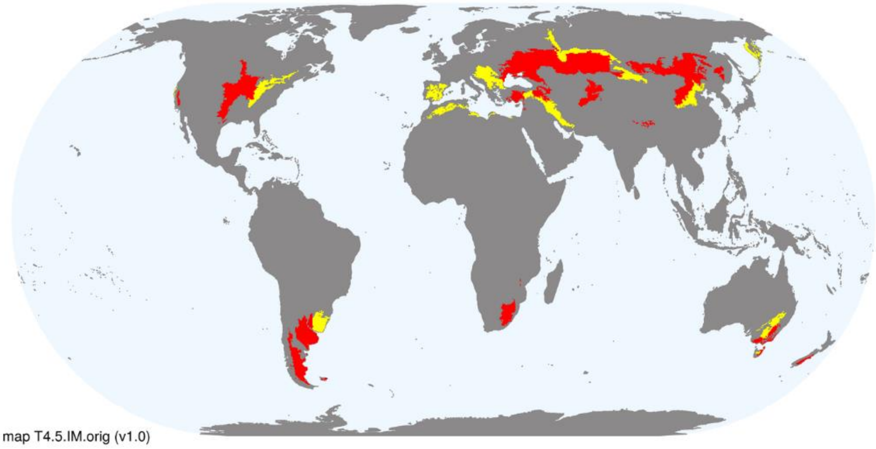

Map is for illustrative purposes only and does not support spatial analyses unless formally validated.

References:

Gibson GJ (2009) *Grasses and grassland ecology* Oxford University Press, Oxford.

T5. Deserts and semi-deserts biome

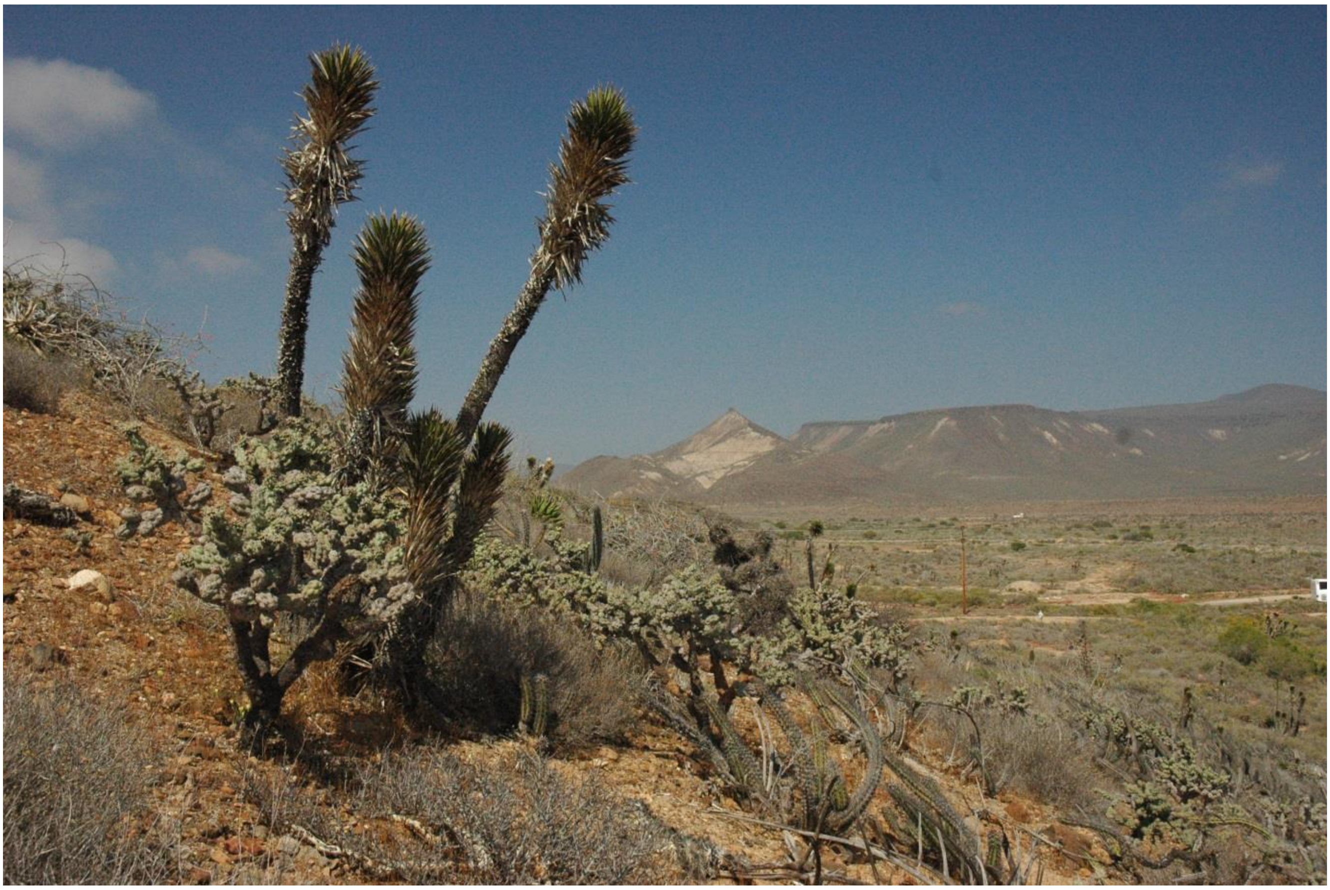

Bahía de los Ángeles, Mexico.

Credit: David Keith

The Deserts and semi-deserts biome includes low to very low biomass ecosystems occurring in arid or semi-arid climates, principally associated with the subtropical high-pressure belts and major continental rain shadows.

Primary productivity is low or very low and dependent on low densities of low-stature photoautotrophs that sustain a complete but sparse trophic web of consumers and predators. Productivity is limited by severe water deficits caused by very low rainfall. Rainfall deficits are exacerbated by extremes of temperature and desiccating winds.

Resources, productivity, and biomass are highly variable in space and time in response to the amount of annual rainfall, the size of individual rainfall events, and the lateral movement of resources from sources to sinks. Landscape heterogeneity and resource gradients are therefore critical to the persistence of desert biota in the context of highly stochastic, unseasonal temporal patterns of rainfall events that drive 'pulse and reserve' or 'boom-bust' ecosystem dynamics. There may be high rates of erosion and sedimentation due to the lack of surface stability provided by the sparse vegetation cover and this can be amplified by the activities of large mammals and people.

Extreme and prolonged water deficits, punctuated by short episodes of surplus, impose severe physiological constraints on plants and animals, which exhibit a variety of physiological, morphological, behavioural, and life-history traits enabling water acquisition and conservation. The life-history spectra of desert systems are polarised between long-lived drought tolerators with low metabolic rates and opportunistic drought evaders with either high mobility or short-lived active phases and long dormant phases. Mobility enables organisms to track transient resources over large distances. Competitive interactions are generally weak relative to most other terrestrial biomes (T1-T4), although herbivory and predation are more evident in the most productive ecosystems and during the decline in resource availability that follows rainfall events.

Contributors: MG Tozer, DJ Eldridge, DA Keith

T5.1 Semi-desert steppe

Ecosystem properties: These mixed semi-deserts are dominated by suffrutescent (i.e. with a woody base) or subsucculent (semi-fleshy) perennial shrubs and tussock grasses. Productivity and biomass are limited by low average precipitation, extreme temperatures and, to a lesser extent, soil nutrients, but vary temporally in response to water availability. Vegetation takes a range of structural forms including open shrublands, mixed shrublands with a tussock grass matrix, prairie-like tall forb grasslands, and very low dwarf shrubs interspersed with forbs or grasses. Total cover varies from 10% to 30% and the balance between shrubs and grasses is mediated by rainfall, herbivory, and soil fertility. Stress-tolerator and ruderal life-history types are strongly represented in flora and fauna. Trait plasticity and nomadism are also common. Traits promoting water capture and conservation in plants include xeromorphy, deep roots, and C4 photosynthesis. Shrubs have small (less than nanophyll), non-sclerophyll, often hairy leaves with moderate SLA. Shrubs act as resource-accumulation sites, promoting heterogeneity over local scales. C3 photosynthesis is represented in short-lived shrubs, forbs, and grasses, enabling them to exploit pulses of winter rain. Consumers include small mammalian and avian granivores, medium-sized mammalian herbivores, and wide-ranging large mammalian and avian predators and scavengers. Abundant detritivores consume dead matter and structure resource availability and habitat characteristics over small scales. Episodic rainfall initiates trophic pulses with rapid responses by granivores and their predators, but less so by herbivores, which show multiple traits promoting water conservation.

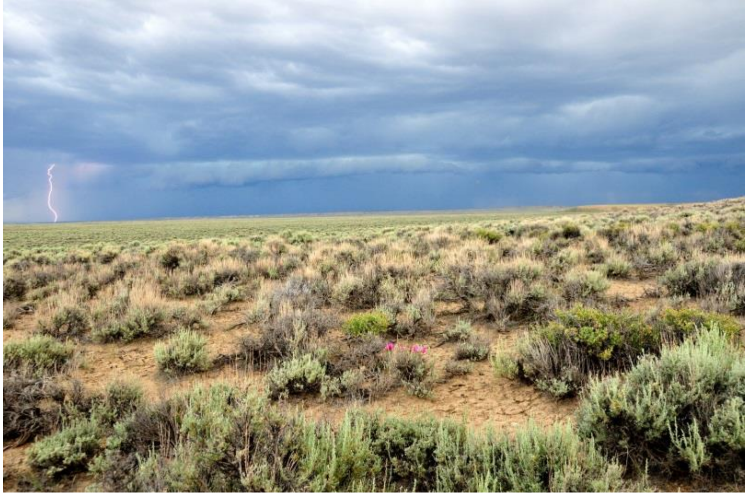

Sagebrush steppe in Seedskadee National Wildlife Refuge, southwest Wyoming.
Credit: Tom Koerner / U.S. Fish and Wildlife Service

Ecological drivers: Semi-desert steppes are associated with fine-textured, calcareous soils of low-moderate fertility, and may contain appreciable levels of magnesium or sodium. Clay particles exchange mineral ions with plant roots and have 'reverse texture effects', limiting moisture extraction as soils dry. Indurated subsoils influence infiltration/runoff relationships and vegetation patterns. Semi-desert steppes are not typically fire-prone and occur in temperate-arid climates. Mean annual rainfall (~150–300 mm), with and has a winter maximum. Evapotranspiration is 2-20 times greater than precipitation, but large rain events bring inter-annual pulses of water surplus. Temperatures are highly variable diurnally and seasonally, often exceeding 40°C in summer and reaching 0°C in winters but rarely with snow.

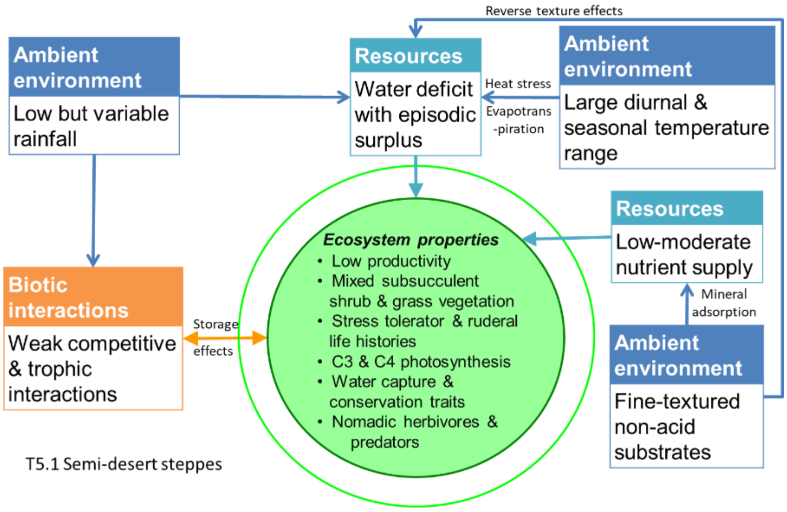

Distribution: Extensive areas across the Sahara, the Arabian Peninsula, west Asia, southwest Africa, southern Australia, Argentina, and the Midwest USA.

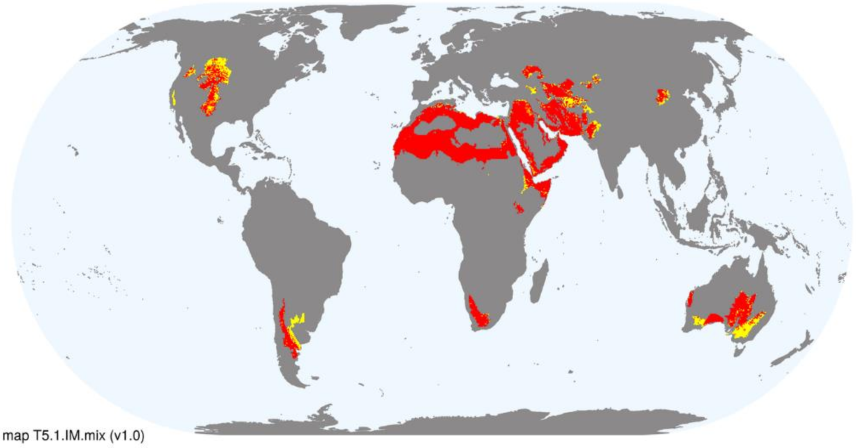

map T5.1.IM.mix (v1.0)

References:

Eldridge DJ, Travers SK, Facelli AF, Facelli JM, Keith DA (2017) The Chenopod shrublands. *Australian vegetation* (Ed. DA Keith), pp 599-625. Cambridge University Press, Cambridge.

West NE (1983) Comparisons and contrasts between the temperate deserts and semi-deserts of three continents. *Ecosystems of the World* Vol. 5. (Ed. NE West) pp. 461–472. (Elsevier: Amsterdam).

Map is for illustrative purposes only and does not support spatial analyses unless formally validated.

Contributors: MG Tozer, DA Keith

T5.2 Succulent or Thorny deserts and semi-deserts

Ecosystem properties: These deserts are characterised by long-lived perennial plants, many with spines and/or succulent stem tissues or leaves. Local endemism is prominent among plants and animals. Productivity is low but relatively consistent through time and limited by precipitation and extreme summer temperatures. Vegetation cover is sparse to moderate (10–30%) and up to several metres tall. Dominant plants are stress-tolerators with slow growth and reproduction, many exhibiting CAM physiology and traits that promote water capture, conservation, and storage. These include deep root systems, suffrutescence, plastic growth and reproduction, succulent stems and/or foliage, thickened cuticles, sunken stomata, and deciduous or reduced foliage. Spinescence in many species is likely a physical defence to protect moist tissues from herbivores. Annuals and geophytes constitute a variable proportion of the flora exhibiting rapid population growth or flowering responses to semi-irregular rainfall events, which stimulate germination of soil seed banks or growth from dormant subterranean organs. Mammalian, reptilian, and invertebrate faunas are diverse, with avian fauna less well represented. Faunal traits adaptive to drought and heat tolerance include physiological mechanisms (e.g. specialised kidney function and reduced metabolic rates) and behavioural characters (e.g. nocturnal habit and burrow dwelling). Many reptiles and invertebrates have ruderal life histories, but fewer mammals and birds do. Larger ungulate fauna exhibit flexible diets and forage over large areas. Predators are present in low densities due to the low productivity of prey populations.

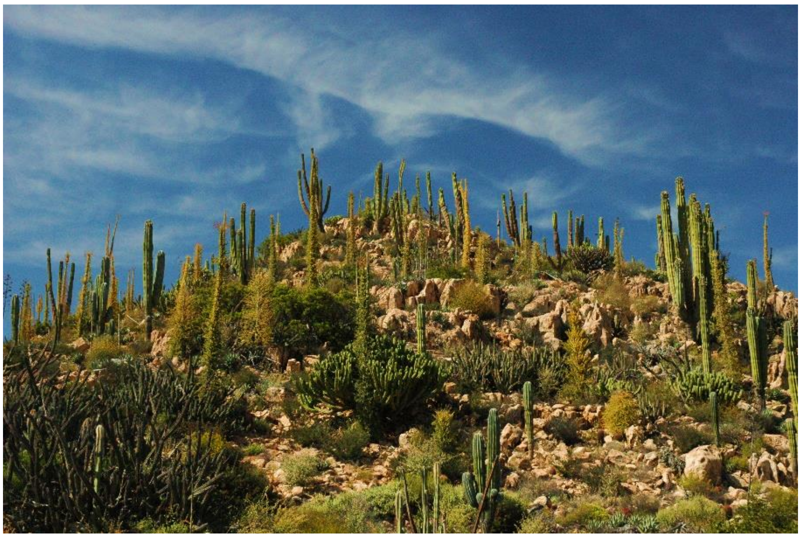

Thorny Desert, Cataviña, Mexico.
Credit: David Keith

Ecological drivers: These systems occur in subtropical arid climates with large overall water deficits. Precipitation is 5–20% of potential evapotranspiration, but exhibits low inter-annual variability relative to other desert systems. Inter-annual pulses of surplus are infrequent and atmospheric moisture from fogs may contribute significantly to available water. Temperatures are hot with relatively large diurnal ranges, but seasonal variation is less than in other deserts, with very hot summers and mild winters. Substrates are stony and produce soils of moderate to low fertility. Thorny deserts are generally not fire-prone.

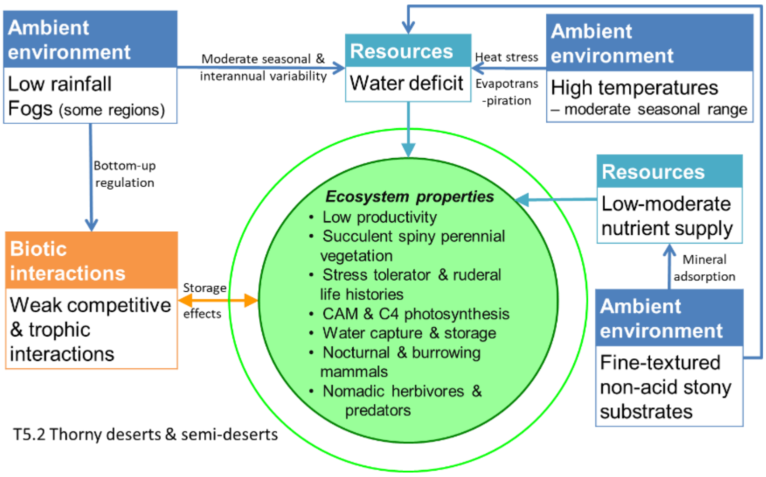

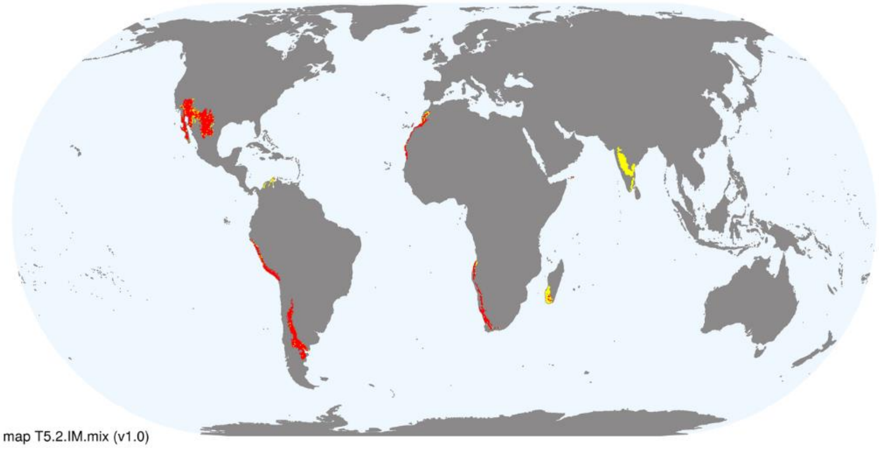

Distribution: Mostly subtropical latitudes in the Americas, southern Africa, and southern Asia.

References:

Shmida A, Evenari M, Noy-Meir I (1986) Hot desert ecosystems: an integrated view. *Ecosystems of the World, Vol. 12b: Hot deserts and arid shrublands* (Eds. M Evenari, I Noy Meir, DW Goodall), pp. 379-387. . Elsevier, Amsterdam.

Map is for illustrative purposes only and does not support spatial analyses unless formally validated.

Contributors: MG Tozer, DA Keith

T5.3 Sclerophyll hot deserts and semi-deserts

Ecosystem properties: Arid systems dominated by hard-leaved (sclerophyll) vegetation have relatively high diversity and local endemism, notably among plants, reptiles, and small mammals. Large moisture deficits and extremely low levels of soil nutrients limit productivity, however, infrequent episodes of high rainfall drive spikes of productivity and boom-bust ecology. Spatial heterogeneity is also critical in sustaining diversity by promoting niche diversity and resource-rich refuges during 'bust' intervals. Stress-tolerator and ruderal life-history types are strongly represented in both flora and fauna. Perennial, long-lived, slow-growing, drought-tolerant, sclerophyll shrubs and hummock (C4) grasses structure the ecosystem by stabilising soils, acting as nutrient-accumulation sites and providing continuously available habitat, shade, and food for fauna. Strong filtering by both nutritional poverty and water deficit promote distinctive scleromorphic and xeromorphic plant traits. They include low SLA, high C:N ratios, reduced foliage, stomatal regulation and encryption, slow growth and reproduction rates, deep root systems, and trait plasticity. Perennial succulents are absent. Episodic rains initiate emergence of a prominent ephemeral flora, with summer and winter rains favouring grasses and forbs, respectively. This productivity 'boom' triggers rapid responses by granivores and their predators. Herbivore populations also fluctuate but less so due to ecophysiological traits that promote water conservation. Abundant detritivores support a diverse and abundant resident reptilian and small-mammal fauna. Small mammals and some macro-invertebrates are nocturnal and fossorial, with digging activity contributing to nutrient and carbon cycling, as well as plant recruitment. The abundance and diversity of top predators is low. Nomadism and ground-nesting are well represented in birds. Periodic fires reduce biomass, promote recovery traits in plants (e.g. re-sprouting and fire-cued recruitment) and initiate successional processes in both flora and fauna.

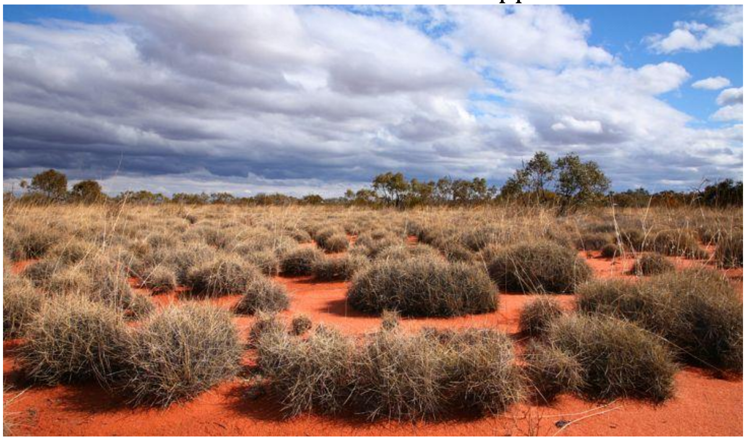

Hummock grasses, Great Victoria Desert, Australia.
Credit: S.D. Hopper

Ecological drivers: Resource availability is limited by a large overall water deficit (rainfall <250 mm p.a., 5–50% of potential evapotranspiration) and acid sandy soils with very low P and N, together with high diurnal and seasonal variation in temperatures. Summers have runs of extremely hot days (>40°C) and winters have cool nights (0°C), rarely with snow. Long dry spells are punctuated by infrequent inter-annual pulses of water surplus, driving ecological booms and transient periods of fuel continuity. Fires occur at decadal- or century-scale return intervals when lightning or human ignitions coincide with fuel continuity.

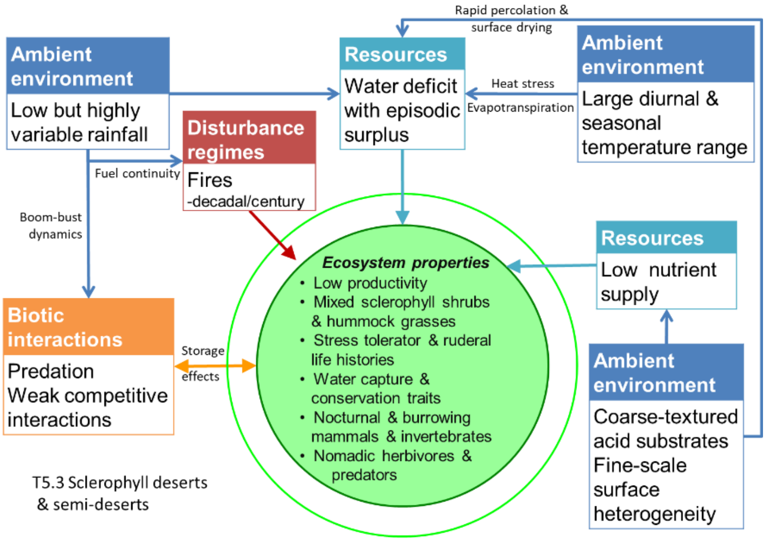

Distribution: Mid-latitudes on sandy substrates of central and northwestern Australia.

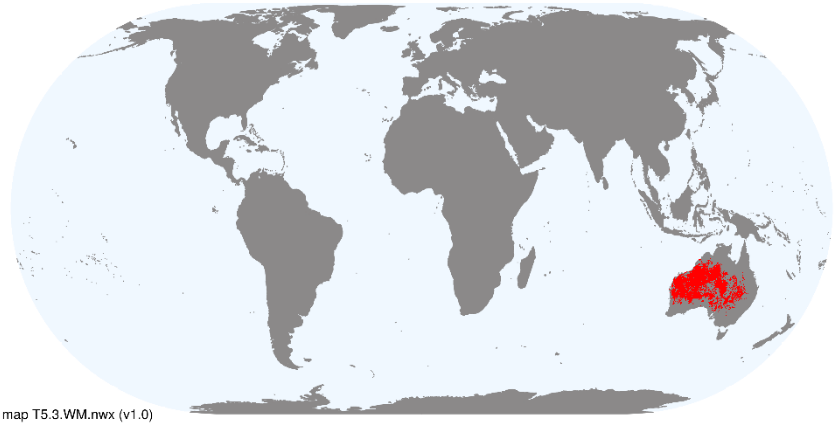

map T5.3.WM.nwx (v1.0)

References:

Keith DA (2004) *Ocean shores to desert dunes: the native vegetation of NSW and the ACT* NSW Department of Environment and Conservation, Sydney.

Morton S, Stafford Smith DM, Dickman CR, Dunkerley DL, Friedel MH, McAllister RRJ, Reid RW, Roshier DA, Smith MA, Walsh FJ, Wardle GM, Watson IW, Westoby M (2011) A fresh framework for the ecology of arid Australia. *Journal of Arid Environments* 75: 313-329.

Map is for illustrative purposes only and does not support spatial analyses unless formally validated.

T5.4 Cool deserts and semi-deserts

Ecosystem properties: In these arid systems, productivity is limited by both low precipitation and cold temperatures but varies spatially in response to soil texture, salinity, and water table depth. Vegetation cover varies with soil conditions from near zero (on extensive areas of heavily salinized soils or mobile dunes) to >50% in upland grasslands and shrublands, but is generally low in stature (<1 m tall). The dominant plants are perennial C3 grasses and xeromorphic suffrutescent or non-sclerophyllous perennial shrubs. Dwarf shrubs, tending to prostrate or cushion forms occur in areas exposed to strong, cold winds. Plant growth occurs mainly during warming spring temperatures after winter soil moisture recharges. Eurasian winter annuals grow rapidly in this period after developing extensive root systems over winter. Diversity and local endemism are low across all taxa relative to other arid ecosystems. Trophic networks are characterised by large nomadic mammalian herbivores. Vertebrate herbivores including antelopes, equines, camelids, and lagomorphs are important mediators of shrub-grass dynamics, with heavy grazing promoting replacement of grasses by N-fixing shrubs. Grasses become dominant with increasing soil fertility or moisture but may be replaced by shrubs as grazing pressure increases. Fossorial lagomorphs and omnivorous rodents contribute to soil perturbation. Predator populations are sparse but taxonomically diverse. They include raptors, snakes, bears, and cats. Bio-crusts with mosses, lichens and cyanobacteria are prominent on fine-textured substrates and become dominant where it is too cold for vascular plants. They play critical roles in soil stability and water and nutrient availability.

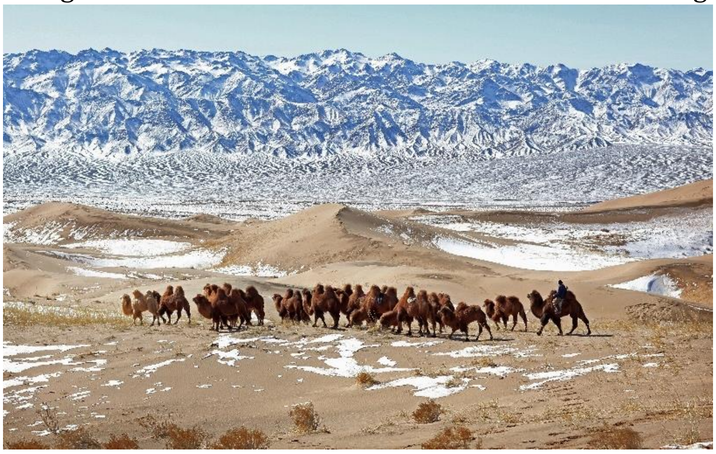

Bactrian camels over the sand dunes of the Gobi desert beneath the Altai Mountains, southern Mongolia.
Credit: Timothy Allen / Getty Images

Ecological drivers: Mean annual precipitation is similar to most warm deserts (<250 mm) due to rain shadows and continentality, however, in cool deserts this falls mainly as snow or sleet in winter rather than rain. Evapotranspiration is less severe than in hot deserts, but a substantial water deficit exists due to low precipitation (mostly 10–50% of evapotranspiration) and strong desiccating winds that may occasionally propagate fires. Mean monthly temperatures may fall below −20°C in winter (freezing the soil surface) and exceed 15°C in summer. Substrates vary from stony plains and uplands to extensive dune fields, with mosaics of clay and sandy regolith underpinning landscape-scale heterogeneity. Large regions were submerged below seas or lakes in past geological eras with internal drainage systems leaving significant legacies of salinity in some lowland areas, especially in clay substrates.

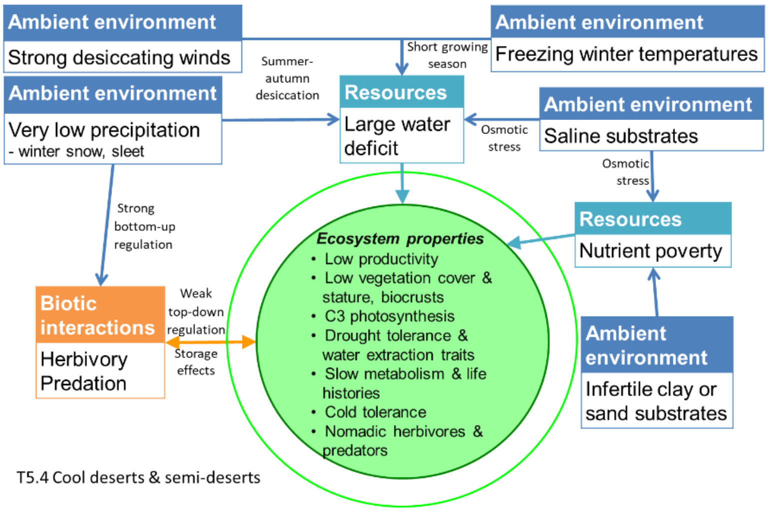

Distribution: Cool temperate plains and plateaus from sea level to 4,000 m elevation in central Eurasia, western North America, and Patagonia. Extreme cold deserts are placed in the polar/alpine biome.

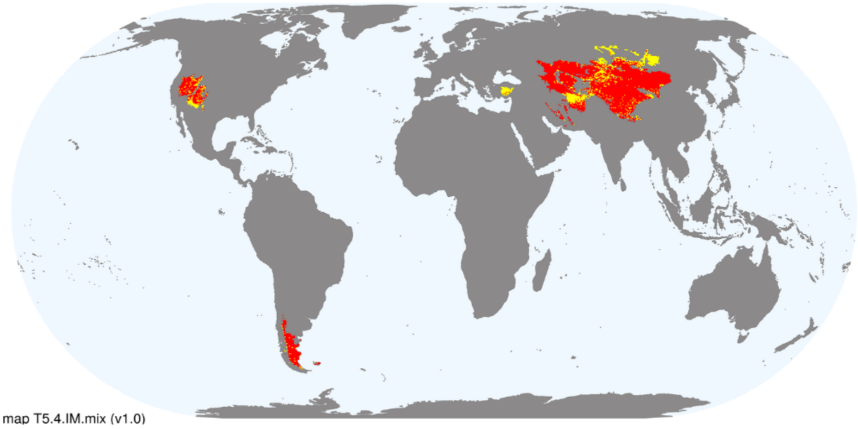

Map is for illustrative purposes only and does not support spatial analyses unless formally validated.

References:

Johnson SL, Kuske CR, Carney TD, Housman DC, Gallegos-Graves LV, Belnap J (2012) Increased temperature and altered summer precipitation have differential effects on biological soil crusts in a dryland ecosystem. *Global Change Biology* 18: 2583-2593.

West NE (1983) Comparisons and contrasts between the temperate deserts and semi-deserts of three continents. *Ecosystems of the World vol. 5: Temperate deserts and semi-deserts* (Ed. NE West). Elsevier, Amsterdam.

Contributors: MG Tozer, D Faber-Langendoen, DA Keith

T5.5 Hyper-arid deserts

Ecosystem properties: Hyper-arid deserts show extremely low productivity and biomass and are limited by low precipitation and extreme temperatures. Vegetation cover is very sparse (<1%) and low in stature (typically a few centimetres tall), but productivity and biomass may be marginally greater in topographically complex landscapes within patches of rising ground-water or where runoff accumulates or cloud cover intersects. Trophic networks are simple because autochthonous productivity and allochthonous resources are very limited. Rates of decomposition are slow and driven by microbial activity and UV-B photodegradation, both of which decline with precipitation. Microbial biofilms play important decomposition roles in soils and contain virus lineages that are putatively distinct from other ecosystems. Although diversity is low, endemism may be high because of strong selection pressures and insularity resulting from the large extent of these arid regions and limited dispersal abilities of most organisms. Low densities of drought-tolerant perennial plants (xerophytes) characterise these systems. The few perennials present have very slow growth and tissue turnover rates, low fecundity, generally long life spans, and water acquisition and conservation traits (e.g. extensive root systems, thick cuticles, stomatal regulation, and succulent organs). Ephemeral plants with long-lived soil seed banks are well represented in hyper-arid deserts characterised by episodic rainfall, but they are less common in those that are largely reliant on fog or groundwater. Fauna include both ruderal and drought-tolerant species. Thermoregulation is strongly represented in reptiles and invertebrates. Birds and large mammals are sparse and nomadic, except in areas with reliable standing water. Herbivores and granivores have boom-bust population dynamics coincident with episodic rains.

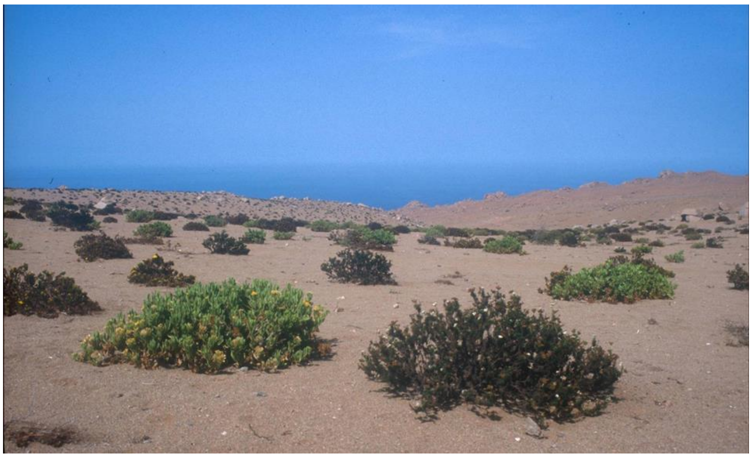

Sparsely vegetated Atacama desert, Peru, where sea fog is the main source of moisture.
Credit: Toby Pennington

Ecological drivers: Extreme rainfall deficit arising from very low rainfall (150 mm to almost zero and <5% of potential evapotranspiration), exacerbated by extremely hot temperatures and desiccating winds. Principal sources of moisture may include moisture-laden fog, irregular inter-annual or decadal rainfall events, and capillary rise from deep water tables. UV-B radiation is extreme except where moderated by fogs. Temperatures exhibit high diurnal and seasonal variability with extreme summer maxima and sub-zero winter night temperatures. Hyper-arid deserts occur on extensive low-relief plains (peneplains) and mountainous terrain. Substrates may be extensive sheets of unstable, shifting sand or stony gibber with no soil profile development and low levels of nutrients.

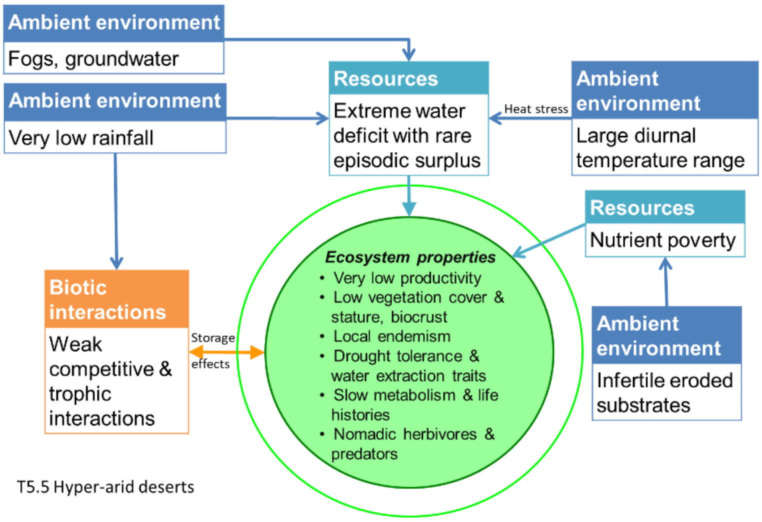

T5.5 Hyper-arid deserts

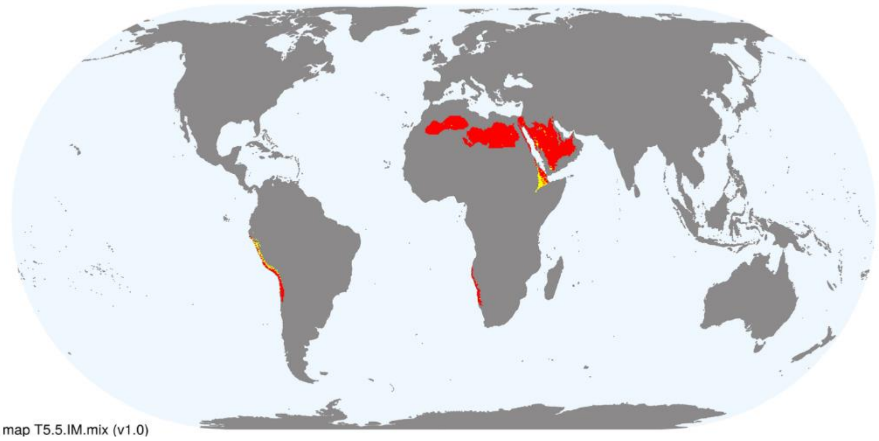

map T5.5.IM.mix (v1.0)

Distribution: Driest parts of the Sahara-Arabian, Atacama, and Namib deserts in subtropical latitudes.

References:

Rundel P W, Dillon MO, Palma B, Mooney HA, Gulmon SL, Ehleringer JR (1991) The phytogeography and ecology of the coastal Atacama and Peruvian deserts. *Aliso* 13(1): 2.

Zablocki O, Adriaenssens EM, Cowan D (2016) Diversity and ecology of viruses in hyperarid desert soils. *Applied and Environmental Microbioly* 82: 770 –777.

Map is for illustrative purposes only and does not support spatial analyses unless formally validated.

T6. Polar/alpine (cryogenic) biome

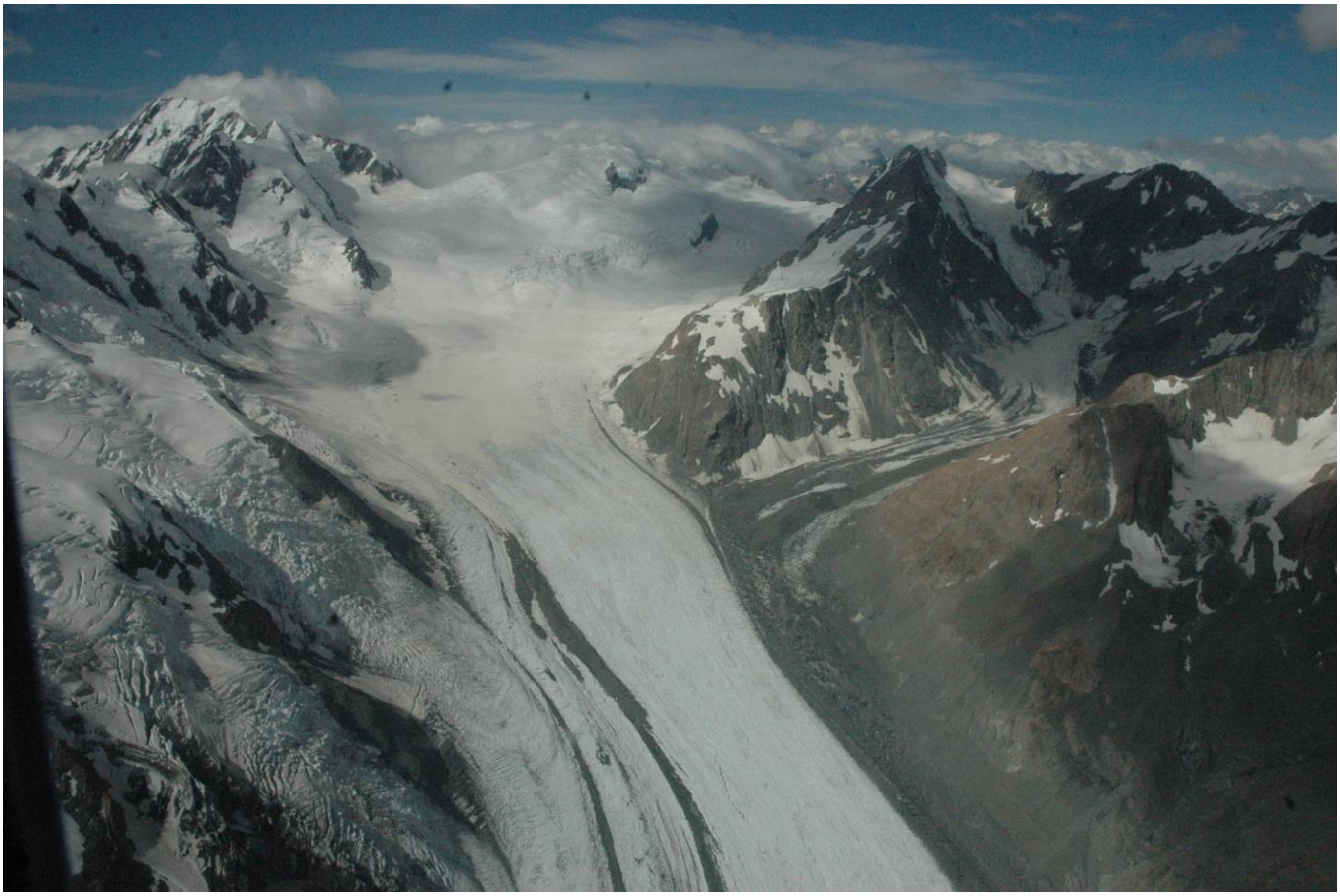

Mt Cook area, South Island, New Zealand.

Credit: David Keith

The Polar-alpine biome encompasses the extensive Arctic and Antarctic regions as well as high mountainous areas across all continental land masses.

Primary productivity is low or very low, strictly seasonal and limited by conditions of extreme cold associated with low insolation and/or high elevation, further exacerbated by desiccating conditions and high-velocity winds. Low temperatures limit metabolic activity and define the length of growing seasons. Microbial decomposition is slow, leading to peat accumulation in the most productive ecosystems. Regional and local temperature gradients shape ecosystems within the biome. Standing biomass, for example, is low or very low and varies with the severity of cold and insolation.

Microbial lifeforms dominate in the coldest systems with perennial snow or ice cover, augmented with crustose lichens, bryophytes, and algae on periodically exposed lithic substrates. Forbs, grasses and dwarf shrubs with slow growth rates and long lifespans become increasingly prominent and may develop continuous cover with increasing insolation and warmer conditions. This vegetation cover provides habitat structure and food for vertebrate and invertebrate consumers and their predators.

Trophic webs are simple or truncated and populations of larger vertebrates are generally migratory or itinerant. In these warmer cryogenic systems, snow cover is seasonal (except at equatorial latitudes) and insulates plants and animals that lie dormant beneath it during winter and during their emergence from dormancy prior to spring thaw. While dormancy is a common trait, a diverse range of other physiological, behavioural, and morphological traits that facilitate cold tolerance are also well represented among the biota.

Contributors: DA Keith, A Terauds, AMB Anesio

T6.1 Ice sheets, glaciers and perennial snowfields

Ecosystem properties: In these icy systems, extreme cold and periodic blizzards limit productivity and diversity to very low levels, and trophic networks are truncated. Wherever surface or interstitial water is available, life is dominated by micro-organisms including viruses, bacteria, protozoa, and algae, which may arrive by Aeolian processes. Bacterial densities vary from 107 to 1011 cells.L-1. On the surface, the main primary producers are snow (mainly Chlamydomonadales) and ice algae (mainly Zygnematales) with contrasting traits. Metabolic activity is generally restricted to summer months at temperatures close to zero and is enabled by exopolymeric substances, cold-adapted enzymes, cold-shock proteins, and other physiological traits. N-fixing cyanobacteria are critical in the N-cycle, especially in late summer. Surface heterogeneity and dynamism create cryoconite holes, rich oases for microbial life (especially cyanobacteria, prokaryotic heterotrophs and viruses) and active biogeochemical cycling. Most vertebrates are migratory birds with only the emperor penguin over-wintering on Antarctic ice. Mass movement and snow burial also places severe constraints on establishment and persistence of life. Snow and ice algae and cyanobacteria on the surface are ecosystem engineers. Their accumulation of organic matter leads to positive feedbacks between melting and microbial activity that discolours snow and reduces albedo. Organic matter produced at the surface can also be transported through the ice to dark subglacial environments, fuelling microbial processes involving heterotrophic and chemoautotrophic prokaryotes and fungi.

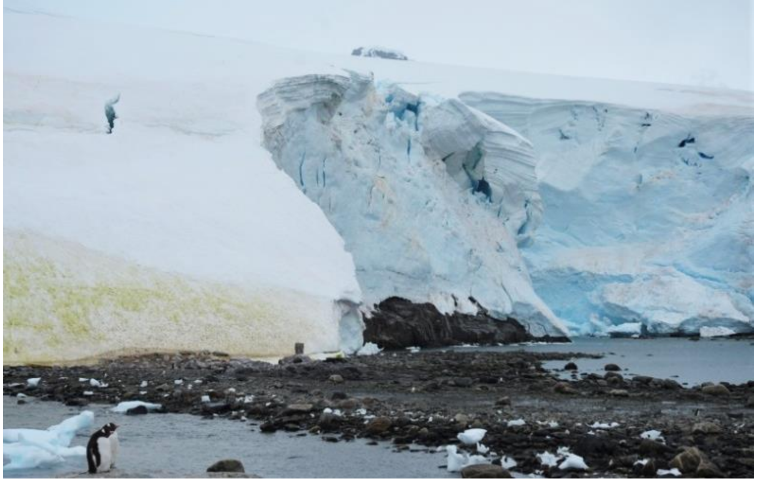

Edge of the Antarctic ice sheet, Paradise Bay.
Credit: David Keith

Ecological drivers: Permanent but dynamic ice cover accumulates by periodic snow fall and is reduced in summer by melting, sublimation, and calving (i.e. blocks of ice breaking free) in the ablation zone. Slow lateral movement occurs downslope or outwards from ice cap centres with associated cracking. Precipitation may average several metres per year on montane glaciers or less than a few hundred millimetres on extensive ice sheets. Surface temperatures are extremely cold in winter (commonly −60°C in Antarctica) but may rise above 0°C in summer. Desiccating conditions occur during high winds or when water is present almost entirely in solid form. Nutrients, especially N and P, are extremely scarce, the main inputs being glacial moraines, aerosols, and seawater (in sea ice), which may be supplemented locally by guano. Below the ice, temperatures are less extreme, there is greater contact between ice, water, and rock (enhancing nutrient supply), a diminished light intensity, and redox potential tends towards anoxic conditions, depending on hydraulic residence times.

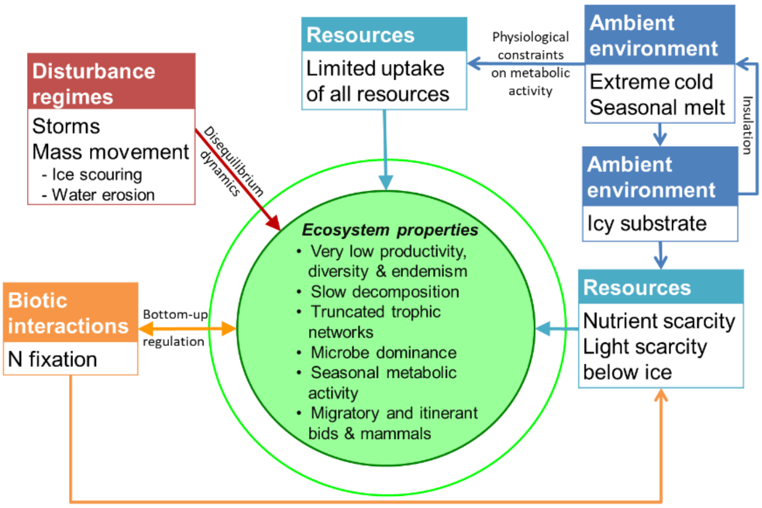

T6.1 Ice sheets, glaciers and perennial snowfields

Distribution: Polar regions and high mountains in the western Americas, central Asia, Europe, and New Zealand, covering ~10% of the earth's surface.

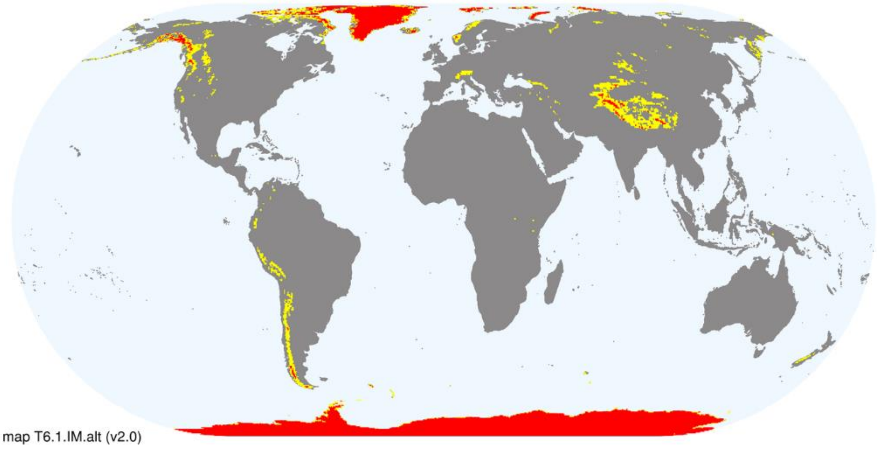

map T6.1.IM.alt (v2.0)

Map is for illustrative purposes only and does not support spatial analyses unless formally validated.

References:

Anesio AM, Laybourn-Parry J (2012) Glaciers and ice sheets as a biome. *Trends in Ecology and Evolution* .
Anesio AM, Lutz S, Chrismas NAM. Benning LG (2017) The microbiome of glaciers and ice sheets. *npj Biofilms Microbiomes* 3, 10.

Contributors: DA Keith, KR Young, A Terauds

T6.2 Polar/alpine cliffs, screes, outcrops and lava flows

Ecosystem properties: Low biomass systems with very low productivity constrained by extreme cold, desiccating winds, skeletal substrates, periodic mass movement, and, in polar regions, by seasonally low light intensity. The dominant lifeforms are freeze-tolerant crustose lichens, mosses, and algae that also tolerate periodic desiccation, invertebrates such as tardigrades, nematodes, and mites, micro-organisms including bacteria and protozoa, and nesting birds that forage primarily in other (mostly marine) ecosystems. Diversity and endemism are low, likely due to intense selection pressures and wide dispersal. Trophic networks are simple and truncated. Physiological traits such as cold-adapted enzymes and cold-shock proteins enable metabolic activity, which is restricted to summer months when temperatures are close to or above zero. Nutrient input occurs primarily through substrate weathering supplemented by guano, which along with cyanobacteria is a major source of N. Mass movement of snow and rock, with accumulation of snow and ice during the intervals between collapse events, promotes disequilibrium ecosystem dynamics.

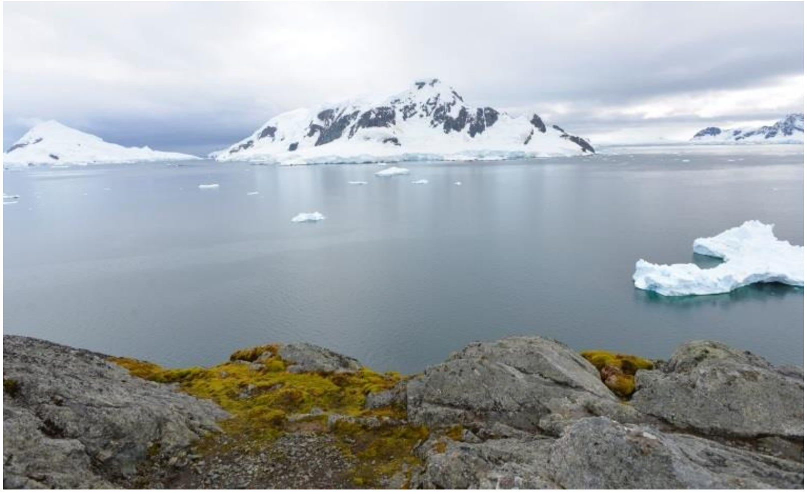

Rocky mountains around Paradise Bay, Antarctica.

Credit: David Keith

Ecological drivers: Extremely cold winters with wind-chill that may reduce temperatures below −80°C in Antarctica. In contrast, insolation and heat absorption on rocky substrates may increase summer temperatures well above 0°C. Together with the impermeable substrate and intermittently high winds, exposure to summer insolation may produce periods of extreme water deficit punctuated by saturated conditions associated with meltwater and seepage. Periodic burial by snow reduces light availability, while mass movement through landslides, avalanches, or volcanic eruptions maintain substrate instability and destroy biomass, limiting the persistence of biota.

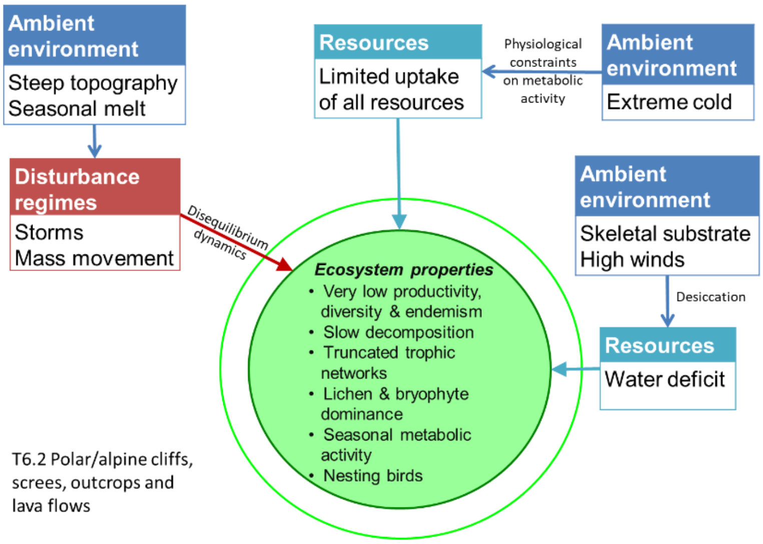

Distribution: Permanently ice-free areas of Antarctica, Greenland, the Arctic Circle, and high mountains in the western Americas, central Asia, Europe, Africa, and New Zealand.

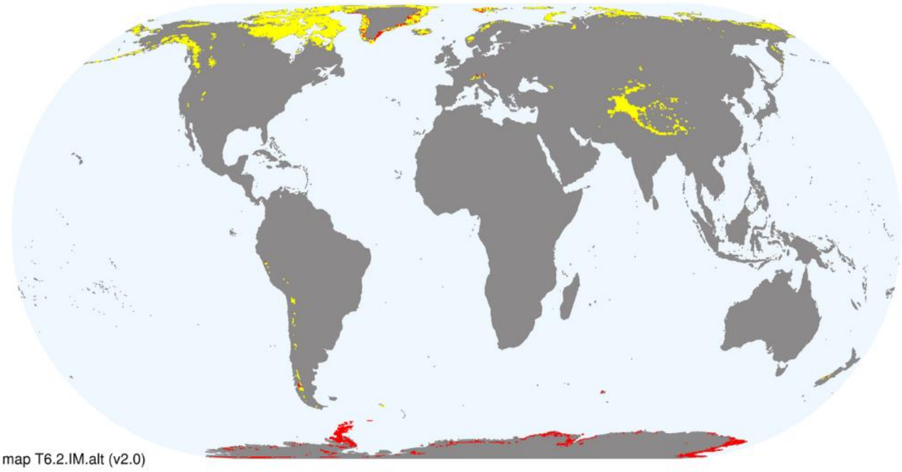

map T6.2.IM.alt (v2.0)

References:

Chown SL, Clarke A, Fraser CI, Cary SC, Moon KL, McGeoch MA (2015) The changing form of Antarctic Biodiversity. *Nature* 522: 431-438.

Convey P, Stevens PI (2007) Antarctic Biodiversity. *Science* 317: 1877-1878.

Map is for illustrative purposes only and does not support spatial analyses unless formally validated.

Contributors: DA Keith, F Essl, KR Young, CH Körner

T6.3 Polar tundra and deserts

Ecosystem properties: These low productivity autotrophic ecosystems are limited by winter dormancy during deep winter snow cover, extreme cold temperatures and frost during spring thaw, short growing seasons, desiccating winds, and seasonally low light intensity. Microbial decomposition rates are slow, promoting accumulation of peaty permafrost substrates in which only the surface horizon thaws seasonally. Vegetation is treeless and dominated by a largely continuous cover of cold-tolerant bryophytes, lichens, C3 grasses, sedges, forbs, and dwarf and prostrate shrubs. Tundra around the world, is delimited by the physiological temperature limits of trees, which are excluded where the growing season (i.e. days >0.9°C) is less than 90-94 days duration, with mean temperatures less than 6.5°C across the growing season. In the coldest and/or driest locations, vascular plants are absent and productivity relies on bryophytes, lichens, cyanobacteria, and allochthonous energy sources such as guano. Aestivating insects (i.e. those that lay dormant in hot or dry seasons) dominate the invertebrate fauna. Vertebrate fauna is dominated by migratory birds, some of which travel seasonal routes exceeding several thousand kilometres. Many of these feed in distant wetlands or open oceans. These are critical mobile links that transfer nutrients and organic matter and disperse the propagules of other organisms, both externally on plumage or feet and endogenously. A few mammals in the Northern Hemisphere are hibernating residents or migratory herbivores. Pinnipeds occur in near-coast tundras and may be locally important marine subsidies of nutrients and energy. Predatory canids and polar bears are nomadic or have large home ranges.

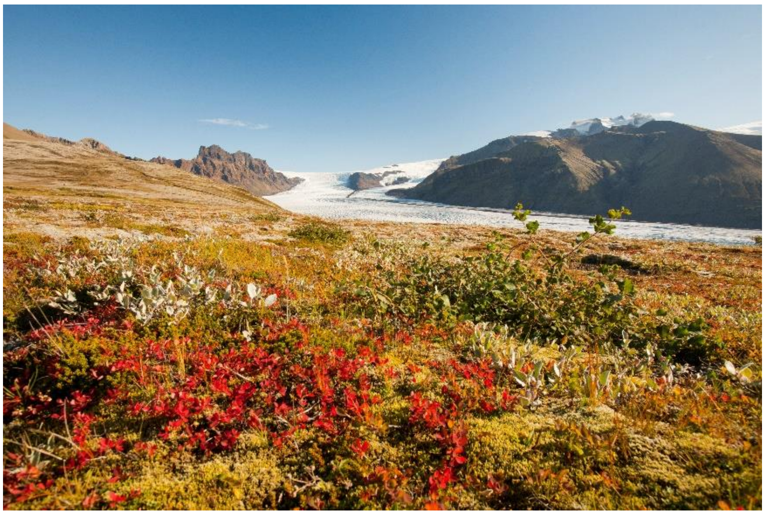

Tundra vegetation at Skaftafellsjokull in Skaftafell National Park, Iceland.
Credit: Ashely Cooper / Getty Images

Ecological drivers: Winters are very cold and dark and summers define short, cool growing seasons with long hours of low daylight. Precipitation falls as snow that persists through winter months. In most areas, there is an overall water surplus, occasionally with small summer deficit, but some areas are ice-free, extremely dry (annual precipitation <150mm p.a.) polar deserts with desiccating winds. Substrates are peaty or gravelly permafrost, which may partially thaw on the surface in summer, causing cryoturbation.

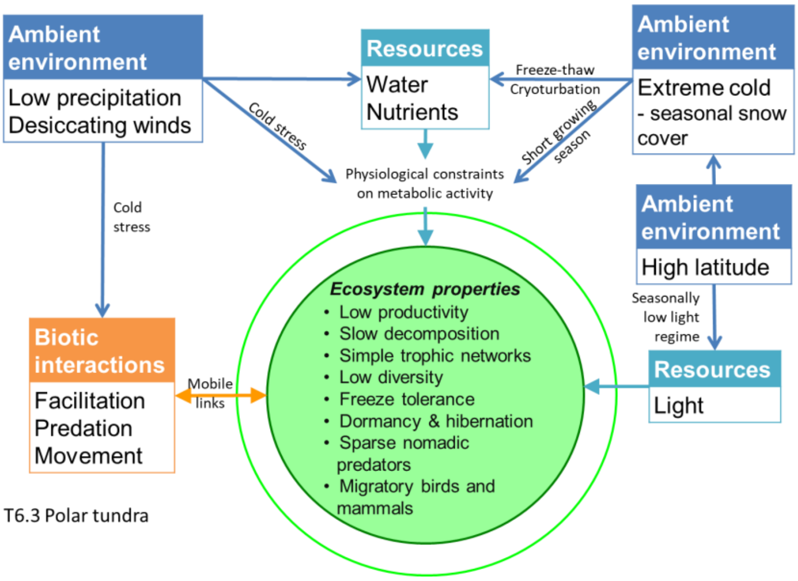

Distribution: Primarily within the Arctic Circle and adjacent subarctic regions, with smaller occurrences on subantarctic islands and the Antarctic coast.

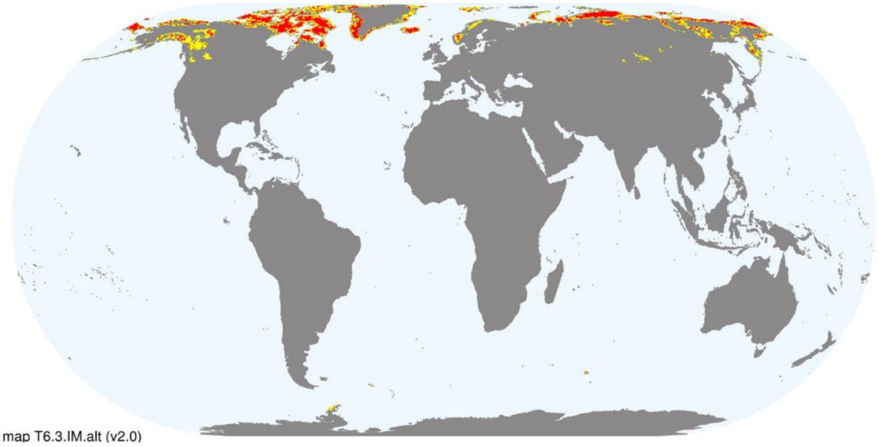

map T6.3.IM.alt (v2.0)

References:

Crawford RMM (2013) *Tundra-taiga biology* Ch 5. Oxford University Press, Oxford.

Paulsen J, Körner C (2014) A climate-based model to predict potential treeline position around the globe. *Alpine Botany* 124, 1-12.

Map is for illustrative purposes only and does not support spatial analyses unless formally validated.

Contributors: DA Keith, F Essl, KR Young, CH Körner

T6.4 Temperate alpine grasslands and shrublands

Ecosystem properties: Mountain systems beyond the cold climatic treeline are dominated by grasses, herbs, or low shrubs (typically <1 m tall). Moderate-low and strictly seasonal productivity is limited by deep winter snow cover, extreme cold and frost during spring thaw, short growing seasons, desiccating winds, and, in some cases, by mass movement. Vegetation comprises a typically continuous cover of plants including bryophytes, lichens, C3 grasses, sedges, forbs, and dwarf shrubs including cushion growth forms. However, the cover of vascular plants may be much lower in low-rainfall regions or in sites exposed to strong desiccating winds and often characterised by dwarf shrubs and lichens that grow on rocks (e.g. fjaeldmark). Throughout the world, alpine ecosystems are defined by the physiological temperature limits of trees, which are excluded where the growing season (i.e. days >0.9°C) is less than 90-94 days, with mean temperatures less than 6.5°C across the growing season. Other plants have morphological and ecophysiological traits to protect buds, leaves, and reproductive tissues from extreme cold, including growth forms with many branches, diminutive leaf sizes, sclerophylly, vegetative propagation, and cold-stratification dormancy. The vertebrate fauna includes a few hibernating residents and migratory herbivores and predators that are nomadic or have large home ranges. Aestivating insects include katydids, dipterans, and hemipterans. Local endemism and beta-diversity may be high due to steep elevational gradients, microhabitat heterogeneity, and topographic barriers to dispersal between mountain ranges, with evidence of both facilitation and competition.

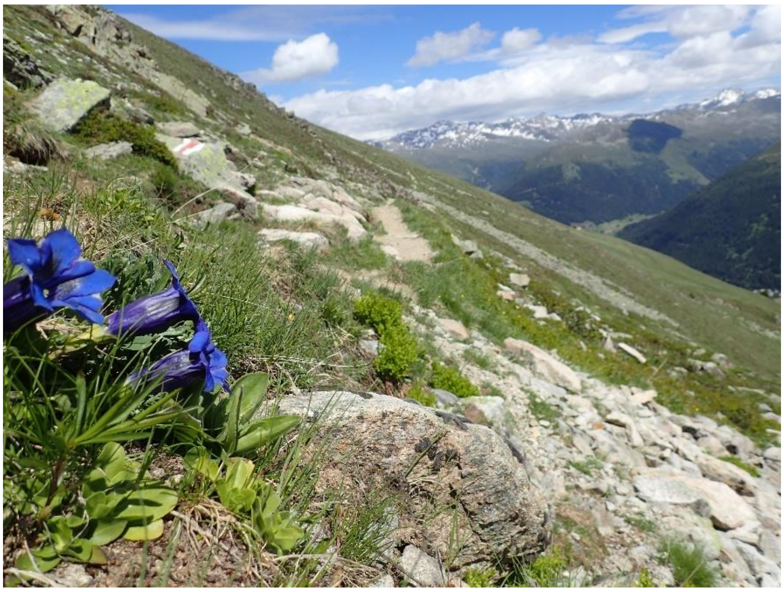

Alpine grassland with diverse herbs, Davos Klosters, Switzerland.
Credit: David Keith

Ecological drivers: Winters are long and cold, while summers are short and mild. Seasonal snow up to several metres deep provides insulation to over-wintering plants and animals. Severe frosts and desiccating winds characterise the spring thaw and exposed ridges and slopes. Severe storms may result from orographic-atmospheric instability. Typically there is a large precipitation surplus, but deficits occur in some regions. Steep elevational gradients and variation in micro-topography and aspect promote microclimatic heterogeneity. Steep slopes are subjected to periodic mass movements, which destroy surface vegetation.

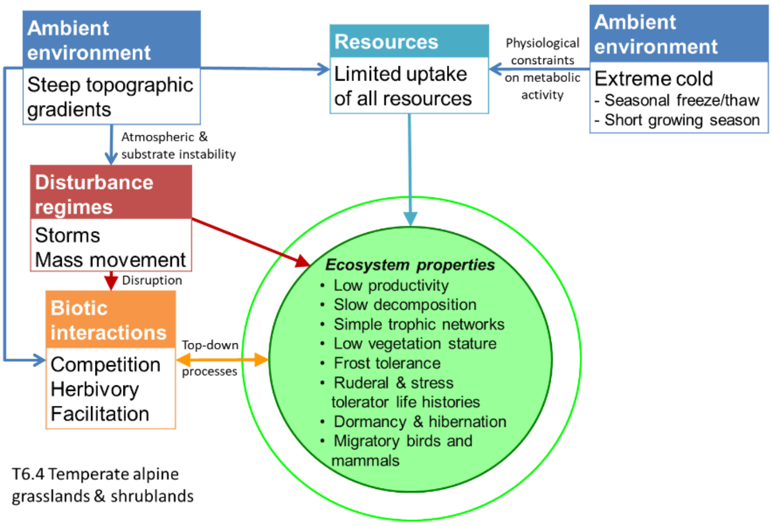

Distribution: Mountains in the temperate and boreal zones of the Americas, Europe, central Eurasia, west and north Asia, Australia, and New Zealand.

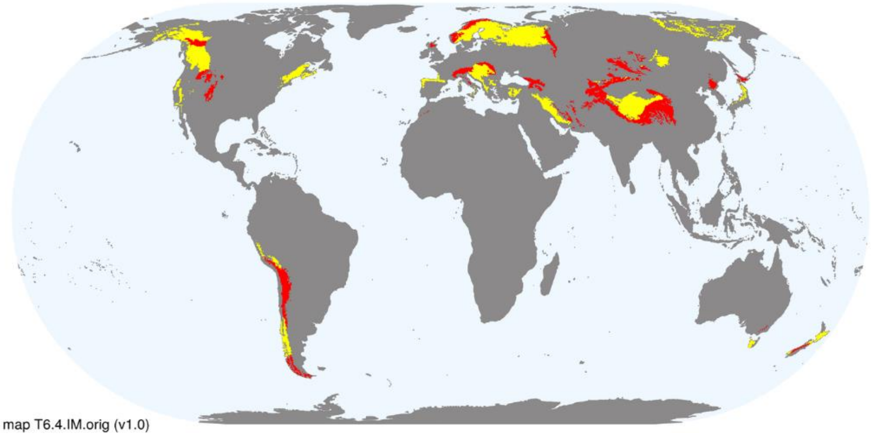

Map is for illustrative purposes only and does not support spatial analyses unless formally validated.

References:

Körner C (2004) Mountain biodiversity: its causes and function. *Ambio* Special Report Number 13. The Royal Colloquium: Mountain Areas: A Global Resource, pp. 11-17. Springer, Berlin.
Körner C (2012) *Alpine treelines* Springer, Basel. ISBN 978-3-0348-0396-0.
Paulsen J, Körner C (2014) A climate-based model to predict potential treeline position around the globe. *Alpine Botany* 124, 1-12.

T6.5 Tropical alpine grasslands and herbfields

Ecosystem properties: Treeless mountain systems dominated by an open to dense cover of cold-tolerant C3 perennial tussock grasses, herbs, small shrubs, and distinctive arborescent rosette or cushion growth forms. Lichens and bryophytes are also common. Productivity is low, dependent on autochthonous energy, and limited by cold temperatures, diurnal freeze-thaw cycles, and desiccating conditions, but not by a short growing season (as in T6.4). Elfin forms of tropical montane forests (T1.3) occupy sheltered gullies and lower elevations. Diversity is low to moderate but endemism is high among some taxa, reflecting steep elevational gradients, microhabitat heterogeneity, and topographic insularity, which restricts dispersal. Solifluction (i.e. the slow flow of saturated soil downslope) restricts seedling establishment to stable microsites. Plants have traits to protect buds, leaves, and reproductive tissues from diurnal cold and transient desiccation stress, including ramulose (i.e. many-branched), cushion, and rosette growth forms, insulation from marcescent (i.e. dead) leaves or pectin fluids, diminutive leaf sizes, leaf pubescence, water storage in stem-pith, and vegetative propagation. Most plants are long-lived and some rosette forms are semelparous. Cuticle and epidermal layers reduce UV-B transmission to photosynthetic tissues. Plant coexistence is mediated by competition, facilitation, herbivory (vertebrate and invertebrate), and fire regimes. Simple trophic networks include itinerant large herbivores and predators from adjacent lowland savannas as well as resident reptiles, small mammals, and macro-invertebrates.

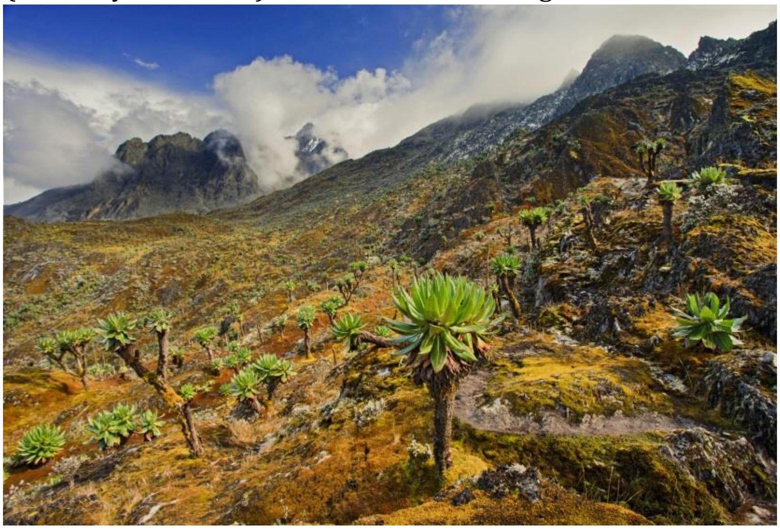

Giant rosettes of Lobelia and Dendrosenecio in alpine herbfields, Rwenzori Mountains, Uganda.
Credit: Rowan Donovan / National Geographic Image Collection / Alamy Stock Photo

Ecological drivers: Cold nights (as low as −10°C) and mild days (up to 15°C) produce low mean temperatures and diurnal freeze-thaw cycles, but seasonal temperature range is small and freezing temperatures are short-lived. Cloud cover and precipitation are unseasonal in equatorial latitudes or seasonal in the monsoonal tropics. Strong orographic effects result in an overall precipitation surplus and snow and fog are common, but desiccating conditions may occur during intervals between precipitation events, with morning insolation also increasing moisture stress when roots are cold. Exposure to UV-B radiation is very high. Substrates are typically rocky and shallow (with low moisture retention capacity) and exposed to solifluction. Micro-topographic heterogeneity influences fine-scale spatial variation in moisture availability. Steep slopes are subjected to periodic mass movements, which destroy surface vegetation. Low-intensity fires may be ignited by lightning or spread upslope from lowland savannas, but these occur infrequently at multi-decadal intervals.

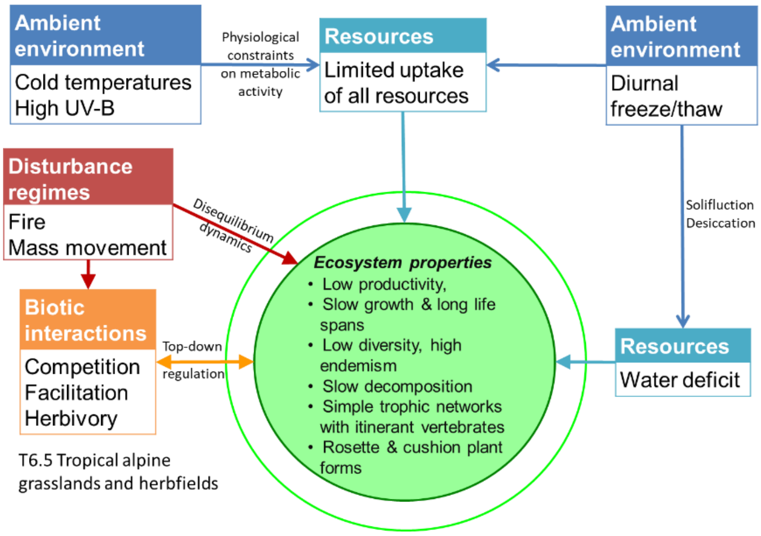

Distribution: Restricted mountainous areas of tropical Central and South America, East and West Africa, and Southeast Asia.

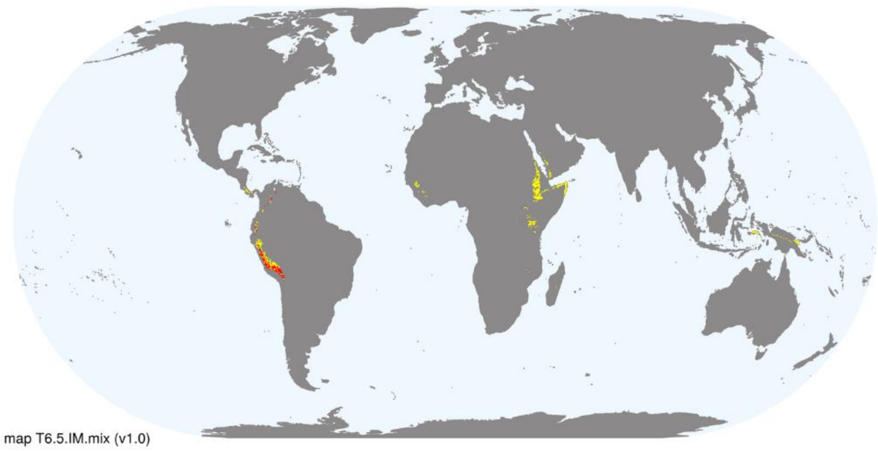

References:

Smith AP, Young TP (1987) Tropical alpine plant ecology. *Annual Review of Ecology and Systematics* 18: 137-58.

Map is for illustrative purposes only and does not support spatial analyses unless formally validated.

T7. Intensive land-use biome

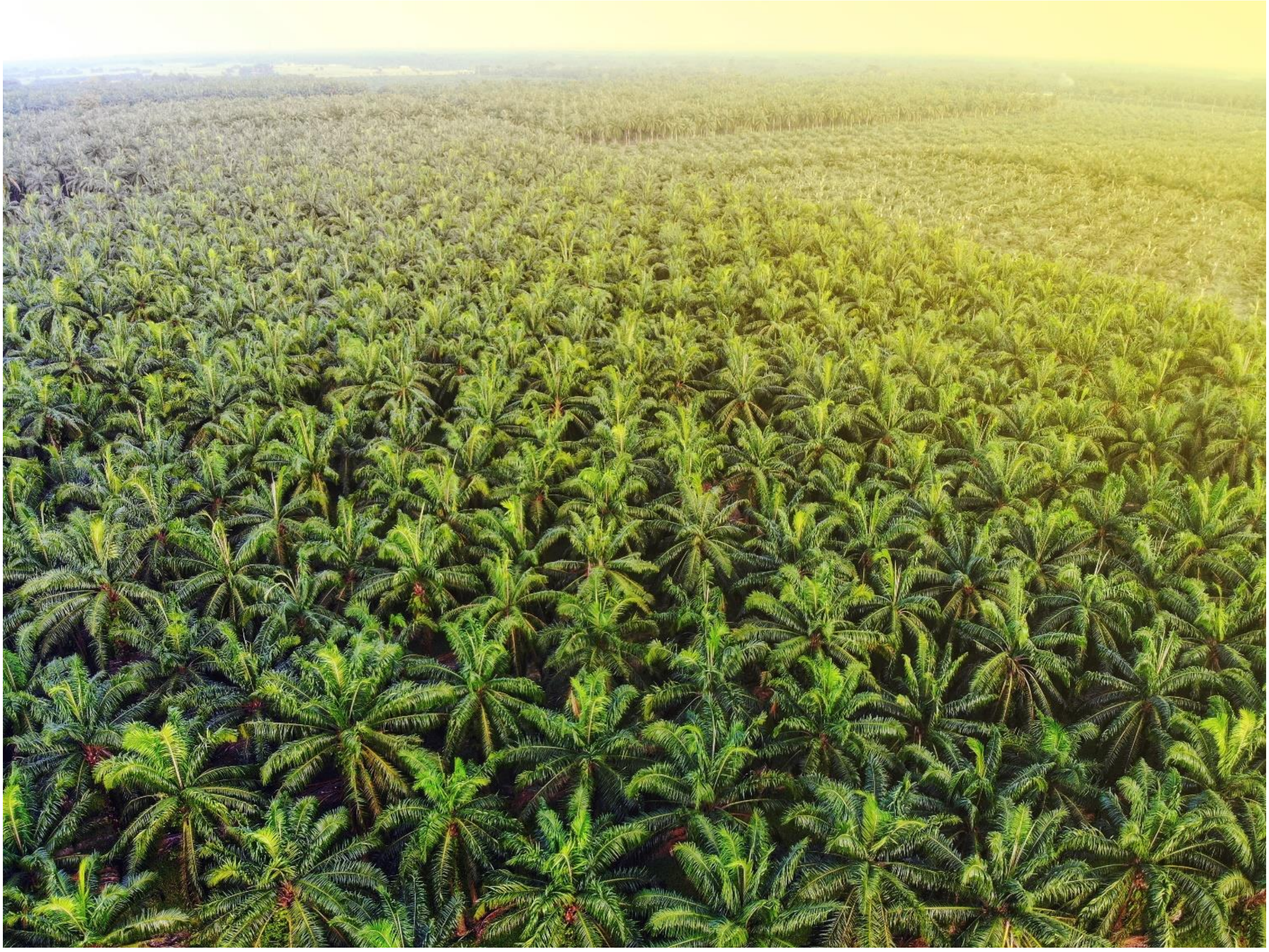

Palm oil plantation, Teluk Intan, Malaysia.

Credit: Hafizal Talib / Eye Em / Getty Images

Intensive land-use systems include major anthropogenic enterprises of cropping, high-density grazing of domesticated livestock, plantation farming, and urbanisation. Human intervention is a dominating influence on this biome, also known as the "anthrome". The intensity of human influence on ecosystems forms a continuum that is best assessed by multidimensional analysis of inputs, outputs, their interactions and alterations to system properties. However, most intensive harvest-based land use systems exhibit a high (> c. 40%) Human Appropriation of Net Primary Productivity (HANPP), an aggregate measure of alteration to ecosystem properties.

Maintenance of production systems is contingent on continuing human interventions, including alterations to the physical structure of vegetation and substrates (e.g. clearing, earthworks, and drainage), the supplementation of resources (e.g. with irrigation and fertilisers), and the introduction and control of biota.

These interventions may maintain disequilibrium community structure and composition, low endemism, and typically low functional and taxonomic diversity relative to comparable systems under low-intensity use, although taxonomic diversity can be higher in some groups in some systems. Target biota are genetically manipulated (by selective breeding or molecular engineering) to promote rapid growth rates, efficient resource capture, enhanced resource allocation to production tissues, and tolerance to harsh environmental conditions, predators, and diseases. Non-target biota include widely dispersed, cosmopolitan opportunists with short lifecycles.

Many intensive land use systems are maintained as artificial mosaics of contrasting patch types at scales of metres to hundreds of metres. Typically, but not exclusively, they are associated with temperate or subtropical climates and the natural availability of freshwater and nutrients from fertile soils on flat to undulating terrain accessible by machinery. The antecedent ecosystems that they replaced include forests, shrublands, grasslands palustrine wetlands and more rarely transitional marine systems (T1, T2, T3, T4, TF1, MT1 and MFT1).

On global and regional scales, intensive land-use systems are engaged in climate feedback processes via alterations to the water cycle and the release of greenhouse gases from vegetation, soils, livestock, and fossil fuels. On local scales, temperatures may be modified by human-built structures (e.g. heat-island effects) or may be artificially controlled

Contributors: DA Keith, R Harper

T7.1 Short-rotation croplands

Ecosystem properties: High-productivity croplands are maintained by the intensive anthropogenic supplementation of nutrients, water, and artificial disturbance regimes (e.g. annual cultivation), translocation (e.g. sowing), and harvesting of annual plants. These systems are typically dominated by one or few shallow-rooted short-lived plant species such as grains (mostly C3 grasses), vegetables, 'flowers', legumes, or fibre species harvested annually by humans for the commercial or subsistence production of food, materials, or ornamental displays. Disequilibrium community structure and composition is maintained by translocations and/or managed reproduction of target species and usually by periodic application of herbicides and pesticides and/or culling to exclude competitors, predators, herbivores, and/or pathogens. Consequently, compared to antecedent 'natural' systems, croplands are structurally simple, have low functional, genetic, and taxonomic diversity and no local endemism. Subsistence croplands, including Swidden rotation systems, are typically more diverse than industrial croplands. Productivity is highly sensitive to variations in resource availability. Target biota are genetically manipulated by selective breeding or molecular engineering to promote rapid growth rates, efficient resource capture, enhanced resource allocation to production tissues, and tolerance to harsh environmental conditions, insect predators, and diseases. Typically, at least 40% of net primary productivity is appropriated by humans. Croplands may be rotated inter-annually with livestock pastures or fallow fields (T7.2) or may be integrated into mixed cropping-livestock systems. Target biota coexists with a cosmopolitan ruderal biota (e.g. weedy plants, mice, and starlings) that exploits production landscapes opportunistically through efficient dispersal, itinerant foraging, rapid establishment, high fecundity, and rapid population turnover. Native biota from adjoining non-anthropogenic systems may also interact with croplands. When actively managed systems are abandoned or managed less intensively, these non-target biota, especially non-woody plants, become dominant and may form a steady, self-maintaining state or a transitional phase to novel ecosystems.

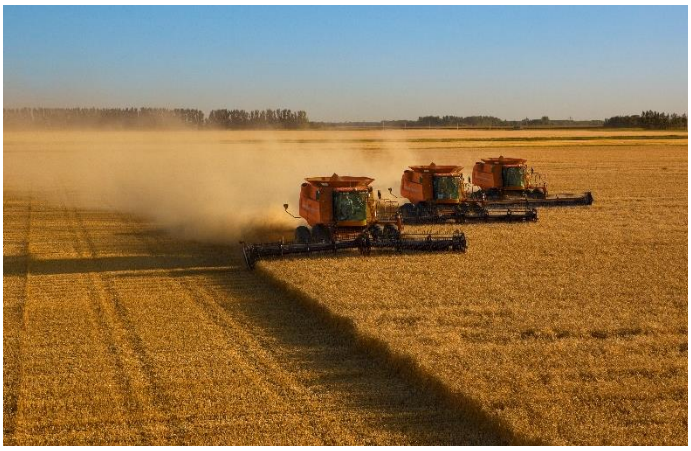

Wheat crop post-harvest, Crookston, Minnesota, USA.
Credit: Andy Sacks / Getty Images

Ecological drivers: The high to moderate natural availability of water (from at least seasonally high rainfall) and nutrients (from fertile soils) is often supplemented by human inputs via irrigation, landscape drainage modifications (e.g. surface earthworks), and/or fertiliser application by humans. Intermittent flooding may occur where croplands replace palustrine wetlands. Temperatures are mild to warm, at least seasonally. These systems are typically associated with flat to moderate terrain accessible by machinery. Artificial disturbance regimes (e.g. annual ploughing) maintain soil turnover, aeration, nutrient release, and relatively low soil organic carbon content.

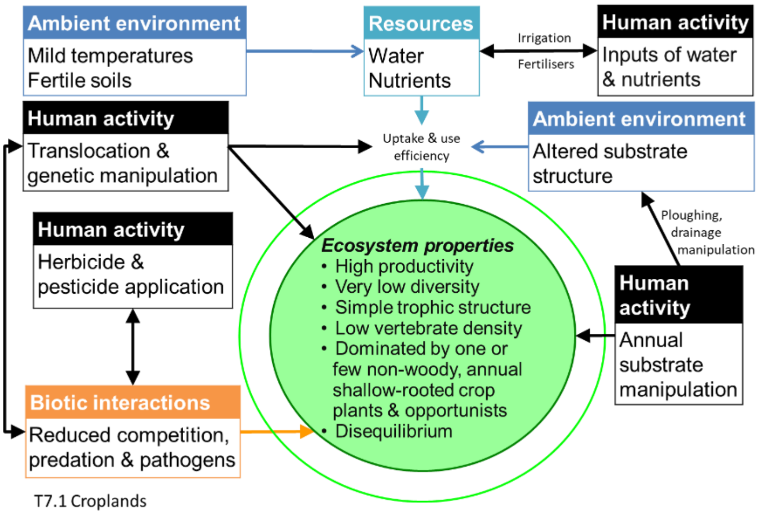

T7.1 Croplands

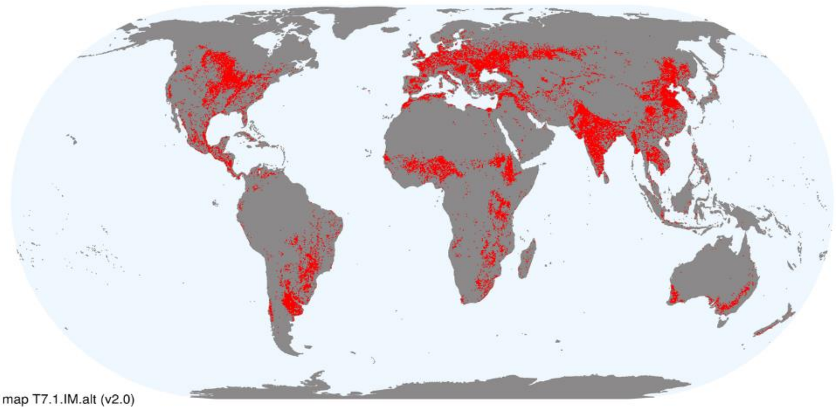

Distribution: Tropical to temperate humid climatic zones or river flats in dry climates across south sub-Saharan and North Africa, Europe, Asia, southern Australia, Oceania, and the Americas.

References:

Leff B, Ramankutty N, Foley JA (2004) Geographic distribution of major crops across the world. *Global Biogeochemical Cycles* 18(1), GB1009.

Ray DK., Foley JA (2013) Increasing global crop harvest frequency: recent trends and future directions. *Environmental Research Letters* 8(4): 044041.

Map is for illustrative purposes only and does not support spatial analyses unless formally validated.

Contributors: DA Keith, R Harper

T7.2 Intensive livestock pastures

Ecosystem properties: Structurally simple, high-productivity pastures are maintained by the intensive anthropogenic supplementation of nutrients (more rarely water) and artificial disturbance regimes (e.g. periodic ploughing,), translocation (e.g. livestock movement and sowing), and harvesting of animals or plants. The magnitude of these inputs distinguish these systems from semi-natural pastures and rangelands in biomes T4 and T5 used for less intense livestock production. They are dominated by one or few selected plant species (C3 and C4 perennial pasture grasses and/or herbaceous legumes) and animal species (usually large mammalian herbivores) for commercial production of food or materials, ornamental displays, or sometimes subsistence. Their composition and structure is maintained by the translocation and/or managed reproduction of target species and the periodic application of herbicides and pesticides and/or culling to exclude competitors, predators, herbivores, or pathogens. Consequently, compared to 'natural' rangeland systems and semi-natural pastures, these systems have low functional and taxonomic diversity and little or no local endemism. Target biota are genetically manipulated to promote rapid growth rates, efficient resource capture, enhanced resource allocation to production tissues, and tolerance to harsh environmental conditions, diseases, and predators, . They are harvested by humans continuously or periodically for consumption or maintenance. Typically, at least 40% of net primary productivity is appropriated by humans. Major examples include intensively managed production pastures for livestock or forage (e.g. hay). Livestock pastures may be rotated inter-annually with non-woody crops (T7.1), or they may be managed as mixed silvo-pastoral systems (T7.3). Target biota coexist with native and cosmopolitan ruderal biota that exploits production landscapes through efficient dispersal, rapid establishment, high fecundity, and rapid population turnover. When the ecosystem is abandoned or managed less intensively, non-target biota become dominant and may form a steady, self-maintaining state or a transitional phase to novel ecosystems.

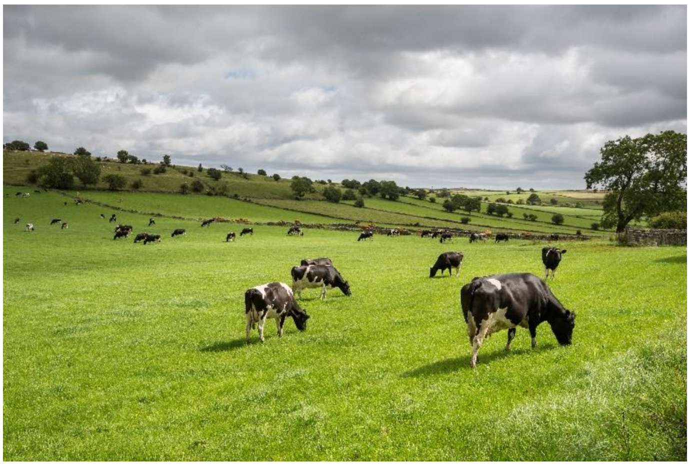

Dairy cattle grazing in sown pastures Buxton, England.
Credit: R A Kearton /Getty Images

Ecological drivers: High R A Kearton / Getty Images to moderate natural availability of water and nutrients is typically supplemented by human inputs via water management, landscape drainage modifications (e.g. surface earthworks), and/or fertiliser application at varied rates. Intermittent flooding may occur where pastures replace palustrine wetlands. Temperatures are mild to warm, at least seasonally. Typically associated with moderately fertile substrates and flat to undulating terrain accessible by machinery. Artificial disturbance regimes (e.g. ploughing for up to 5 years/decade) maintain soil turnover, aeration, and nutrient release.

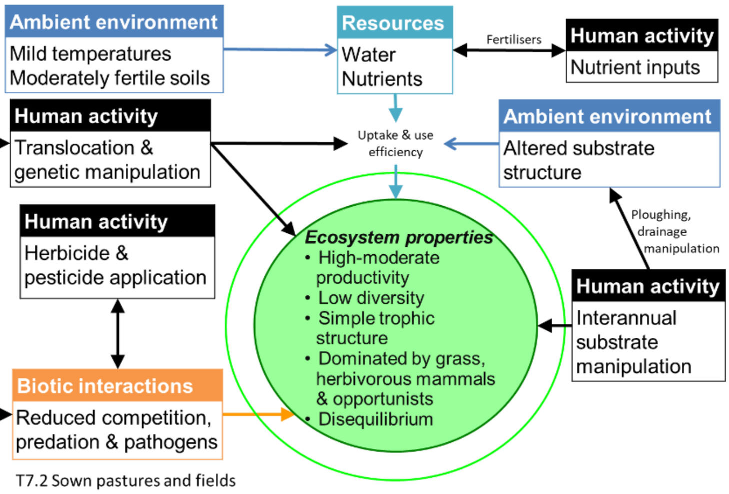

T7.2 Sown pastures and fields

Distribution: Mostly in tropical to temperate climatic zones and developed countries across Europe, east and south Asia, subtropical and temperate Africa, southern Australasia, north and central America, and temperate south America. See map caveats (Table S4.1)

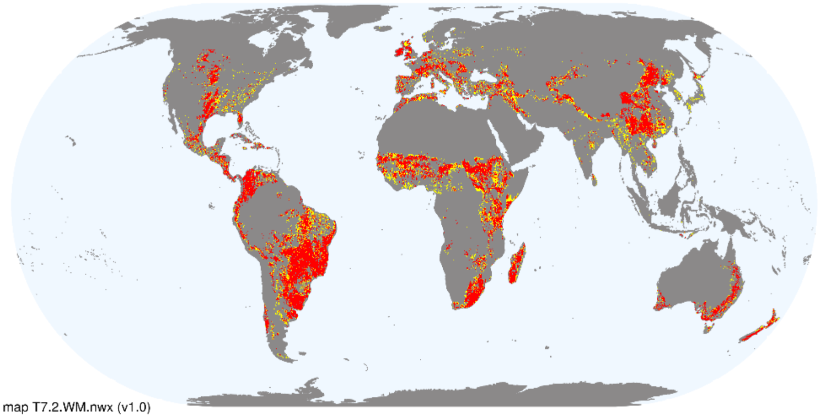

map T7.2.WM.nwx (v1.0)

References:

Bernués A, Ruiz R, Olaizola A, Villalba D, Casasús I (2011) Sustainability of pasture-based livestock farming systems in the European Mediterranean context: Synergies and trade-offs. *Livestock Science* 139: 44–57.

Spedding CRW (1986) Animal production from grass: A systems approach. *Bioindustrial ecosystems* (Eds. DAJ Cole, GC Brander), pp 107-120. Ecosystems of the world vol. 21. Elsevier, Amsterdam.

Map is for illustrative purposes only and does not support spatial analyses unless formally validated.

Contributors: DA Keith, KR Young

T7.3 Plantations

Ecosystem properties: These moderate to high productivity autotrophic systems are established by the translocation (i.e. planting or seeding) of woody perennial plants. Target biota may be genetically manipulated by selective breeding or molecular engineering to promote rapid growth rates, efficient resource capture, enhanced resource allocation to production tissues, and tolerance of harsh environmental conditions, insect predators, and diseases. The diversity, structure, composition, function, and successional trajectory of the ecosystem depends on the identity, developmental stage, density, and traits (e.g. phenology, physiognomy, and growth rates) of planted species, as well as the subsequent management of plantation development. Most plantations comprise at least two vertical strata (the managed woody species and a ruderal ground layer). Mixed forest plantings may be more complex and host a relatively diverse flora and fauna if managed to promote habitat features. Cyclical harvest may render the habitat periodically unsuitable for some biota. Mixed cropping systems may comprise two vertical strata of woody crops or a woody and herbaceous layer. Secondary successional processes involve colonisation and regeneration, initially of opportunistic biota. Successional feedbacks occur as structural complexity increases, promoting visits or colonisation by vertebrates and the associated dispersal of plants and other organisms. Crop replacement (which may occur on inter-annual or decadal cycles), the intensive management of plantation structure, or the control of non-target species may reset, arrest, or redirect successional processes. Examples with increasing management intervention include: environmental plantations established for wildlife or ecosystem services; agroforestry plantings for subsistence products or livestock benefits; forestry plantations for timber, pulp, fibre, bio-energy, rubber, or oils; and vineyards, orchards, and other perennial food crops (e.g. cassava, coffee, tea, palm oil, and nuts). Secondary (regrowth) forests and shrublands are not included as plantations even where management includes supplementary translocations.

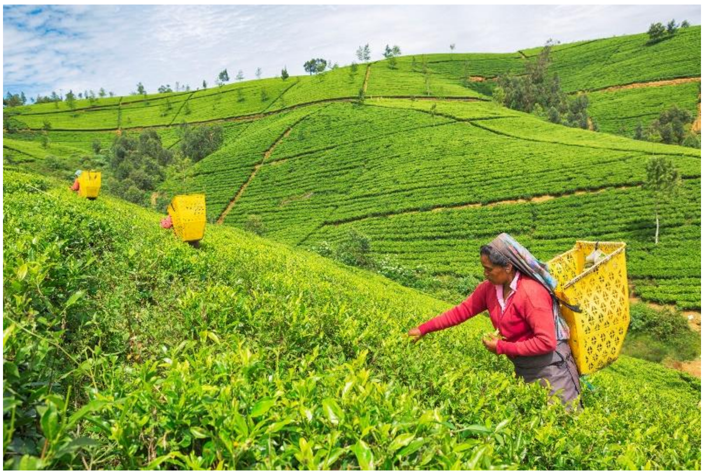

Harvesting in tea plantations, Nuwara Eliya, Sri Lanka.
Credit: Tunart /Getty Images

Ecological drivers: High to moderate natural availability of water and nutrients is supplemented by human inputs of fertiliser or mulch, landscape drainage modifications (e.g. surface earthworks), and, in intensively managed systems, irrigation. Rainfall is at least seasonally high. Temperatures are mild to warm, at least seasonally. Artificial disturbance regimes involving the complete or partial removal of biomass and soil turnover are implemented at sub-decadal to multi-decadal frequencies.

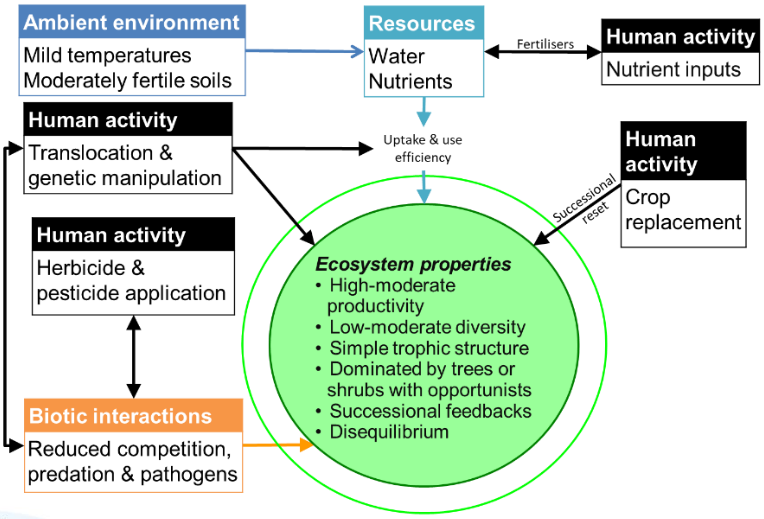

Distribution: Tropical to cool temperate humid climatic zones or river flats in dry climates across south sub-Saharan and Mediterranean Africa, Europe, Asia, southern Australia, Oceania, and the Americas.

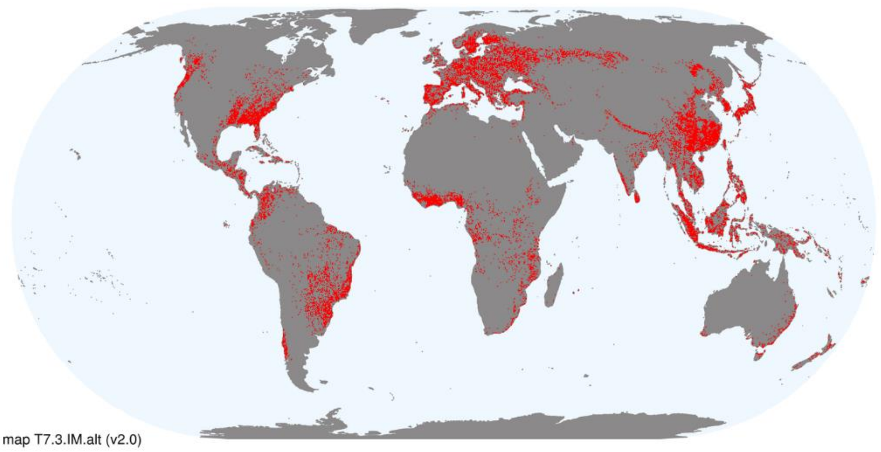

References:

Kanninen M (2010) Plantation forests: global perspectives. *Ecosystem goods and services from plantation forests* (Eds. J Bauhus, PJ van der Meer, M Kanninen), pp1-15. Earthscan, London.

Monfreda CN, Ramankutty N, Foley JA (2008) Farming the planet: 2. Geographic distribution of crop areas, yields, physiological types, and net primary production in the year 2000. Global Biogeochemical Cycles 22: GB1022.

Map is for illustrative purposes only and does not support spatial analyses unless formally validated.

T7.4 Cities, villages and infrastructure

Ecosystem properties: These systems are structurally complex and highly heterogeneous fine-scale spatial mosaics of diverse patch types that may be recognised in fine-scale land use classifications. These include: a) buildings; b) paved surfaces; c) transport infrastructure: d) treed areas; e) grassed areas; f) gardens; g) mines or quarries; h) bare ground; and i) refuse areas. Patch mosaics are dynamic over decadal time scales and driven by socio-ecological feedbacks and a human population that is highly stratified, functionally, socially and economically. Interactions among patch types and human social behaviours produce emergent properties and complex feedbacks among components within each system and interactions with other ecosystem types. Unlike most other terrestrial ecosystems, the energy, water and nutrient sources of urban/industrial village systems are highly allochthonous and processes within urban systems drive profound and extensive global changes in land use, land cover, biodiversity, hydrology, and climate through both resource consumption and waste discharge. Biotic community structure is characterised by low functional and taxonomic diversity, highly skewed rank-abundance relationships and relict local endemism. Trophic networks are simplified and sparse and each node is dominated by few taxa. Urban/village biota include humans, dependents (e.g. companion animals and cultivars), opportunists and vagrants, and legacy biota whose establishment pre-dates settlement. Many biota have highly plastic realised niches, traits enabling wide dispersal, high fecundity, and short generation times. The persistence of dependent biota is maintained by human-assisted migration, managed reproduction, genetic manipulation, amelioration of temperatures, and intensive supplementation of nutrients, food, and water. Pest biota are controlled by the application of herbicides and pesticides or culling with collateral impacts on non-target biota.

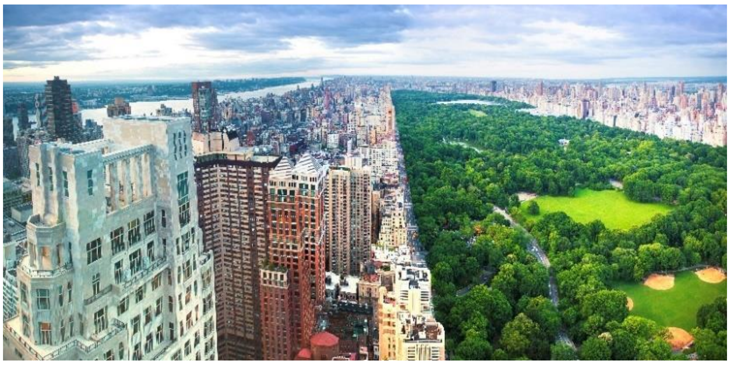

Manhattan and Central Park, New York, USA.
Credit: Alexander Spatari /Getty Images

Ecological drivers: Humans influence the availability of water, nutrients, and energy through governance systems for resource importation and indirectly through interactions and feedbacks. Light is enhanced artificially at night. Urban temperature regimes are elevated by the anthropogenic conversion of chemical energy to heat and the absorption of solar energy by buildings and paved surfaces. However, temperatures may be locally ameliorated within buildings. Surface water runoff is enhanced and percolation is reduced by sealed surfaces. Chemical and particulate air pollution, as well as light and noise pollution may affect biota. Infrastructure development and renewal, driven by socio-economic processes, as well as natural disasters (e.g. storms, floods, earthquakes, and tsunami) create recurring disturbances. There is frequent movement of humans and associated biota and matter between cities.

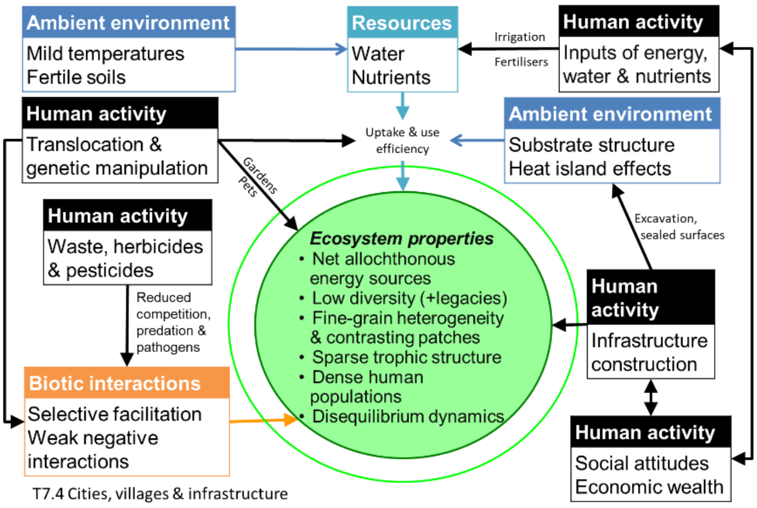

Distribution: Extensively scattered through equatorial to subpolar latitudes from sea-level to submontane altitudes, mostly in proximity to the coast, rivers or lakes, especially in North America, Western Europe and Japan, as well as India, China, and Brazil. Land use maps depict fine-scale patch types listed above.

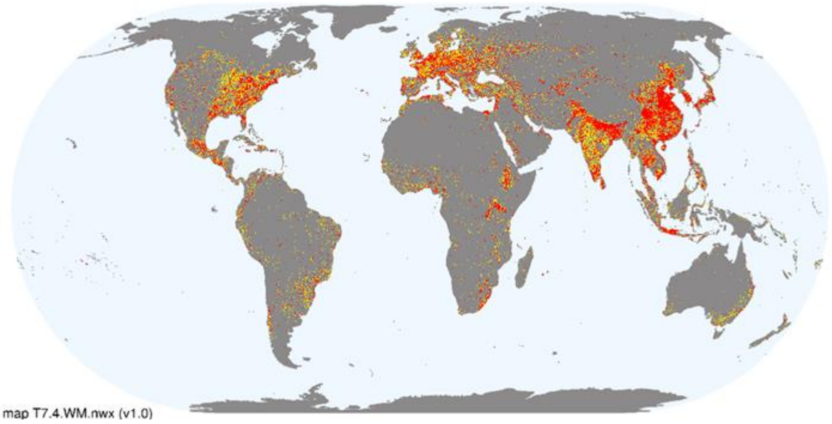

map T7.4.WM.nwx (v1.0)

Map is for illustrative purposes only and does not support spatial analyses unless formally validated.

References:

Alberti M, Marzluff JM, Shulenberger E, Bradley G, Ryan C, Zumbrunnen C (2003) Integrating humans into ecology: Opportunities and challenges for studying urban ecosystems. *Urban Ecology* (Eds. Marzluff JM et al.) Springer, Boston, MA.

Grimm NB, Faeth SH, Golubiewski NE, Redman CL, Wu J, Bai X, Briggs JM (2008) Global change and the ecology of cities. *Science* 319: 756-760.

T7.5 Derived semi-natural pastures and old fields

Ecosystem properties: Extensive 'semi-natural' grasslands and open shrublands exist where woody components of vegetation have been removed or greatly modified for agricultural land uses. Hence they have been 'derived' from a range of other ecosystems (mostly from biomes T1, T2, T3, T4, a few from T5). Remaining vegetation includes a substantial component of local indigenous species, as well as an introduced exotic element, providing habitat for a mixed indigenous and non-indigenous fauna. Although structurally simpler at site scales than the systems from which they were derived, spatial complexity may be greater in fragmented landscapes and they often harbour appreciable diversity of native organisms, including some no longer present in 'natural' ecosystems. Dominant plant growth forms include tussock or stoloniferous grasses and forbs, with or without non-vascular plants, shrubs and scattered trees. These support microbial decomposers and diverse invertebrate groups that function as detritivores, herbivores and predators, as well as vertebrate herbivores and predators characteristic of open habitats. Energy sources are primarily autochthonous, with varying levels of indirect allochthonous subsidies (e.g. via surface water sheet flows), but few managed inputs (cf. T7.2). Productivity can be low or high, depending on climate and substrate, but is generally lower and more stable than more intensive anthropogenic systems (T7.1-T7.3). Trophic networks include all levels, but complexity and diversity depends on the species pool, legacies from antecedent ecosystems, successional stage, and management regimes. These novel ecosystems may persist in a steady self-maintaining state, or undergo passive transformation (e.g. oldfield succession) unless actively maintained in disequilibrium. For example, removal of domestic herbivores may initiate transition to tree-dominated ecosystems.

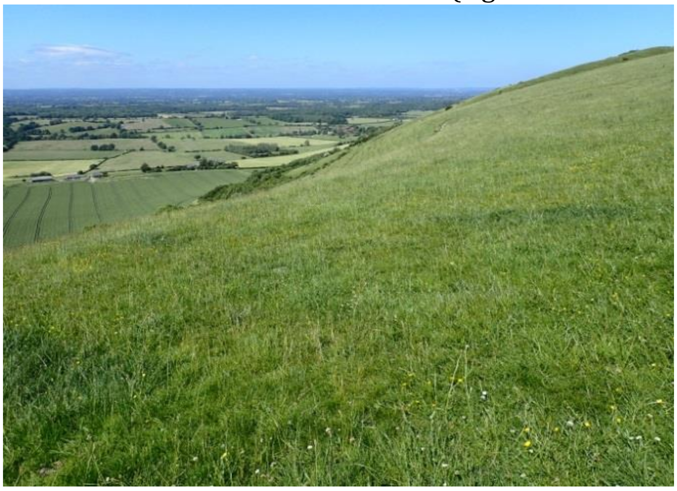

Semi-natural grassland, South Downs, England.
Credit: David Keith

Ecological drivers: Availability of water and nutrients varies depending on local climate, substrate and terrain (hence surface water movement and infiltration). The structure, function and composition of these ecosystems are shaped by legacy features of antecedent systems from which they were derived, as well as ongoing and past human activities. These activities may reflect production and/or conservation goals, or abandonment. They include active removal of woody vegetation, management of vertebrate herbivores, introductions of biota, control of 'pest' biota, manipulation of disturbance regimes, drainage and earthworks, etc. Fertilisers and pesticides are not commonly applied.

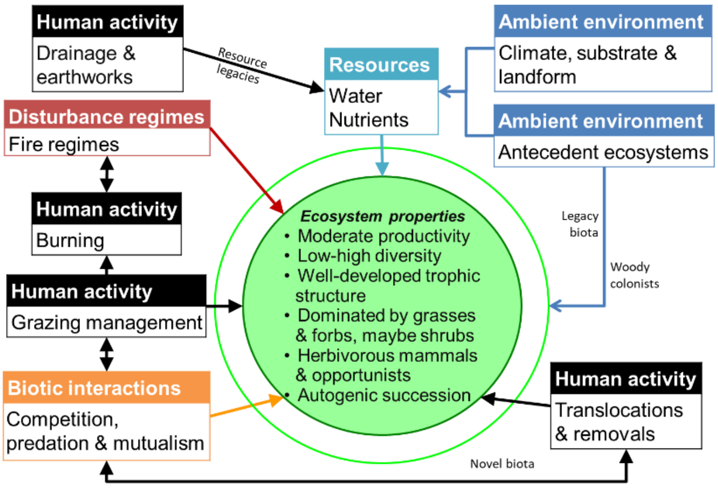

T7.5 Derived semi-natural pastures and old fields

Distribution: Mostly in temperate to tropical climates across all land masses. See map caveats (Table S4.1).

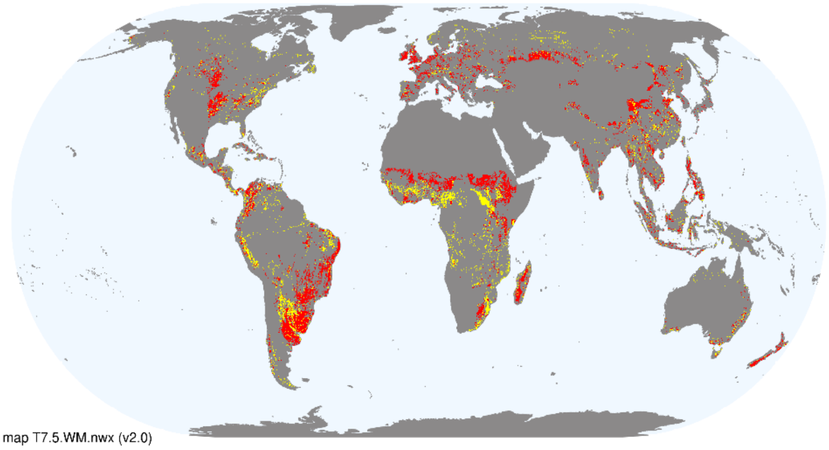

map T7.5.WM.nwx (v2.0)

References:

Cramer VA, Hobbs RJ, Standish RJ (2008) What's new about old fields? Land abandonment and ecosystem assembly. *Trends in Ecology & Evolution* 23: 104-112.

García-Feced, Weissteiner CJ, Baraldi A, Paracchini MA, Maes J, Zulian G, Kempen M, Elbersen B, Pérez-Sob M (2015) Semi-natural vegetation in agricultural land: European map and links to ecosystem service supply. *Agronomy for Sustainable Development* 35: 273–283.

Krauss J, Bommarco R, Guardiola M, Heikkinen RK, Helm A, Kuussaari M, Lindborg R, Öckinger E, Pärtel M, Pino J, Pöyry J, Raatikainen KM, Sang A, Stefanescu C, Teder T, Zobel M, Steffan-Dewenter I (2010) Habitat fragmentation causes immediate and time delayed biodiversity loss at different trophic levels. *Ecology Letters* 13, 597–605.

S1. Subterranean lithic biome

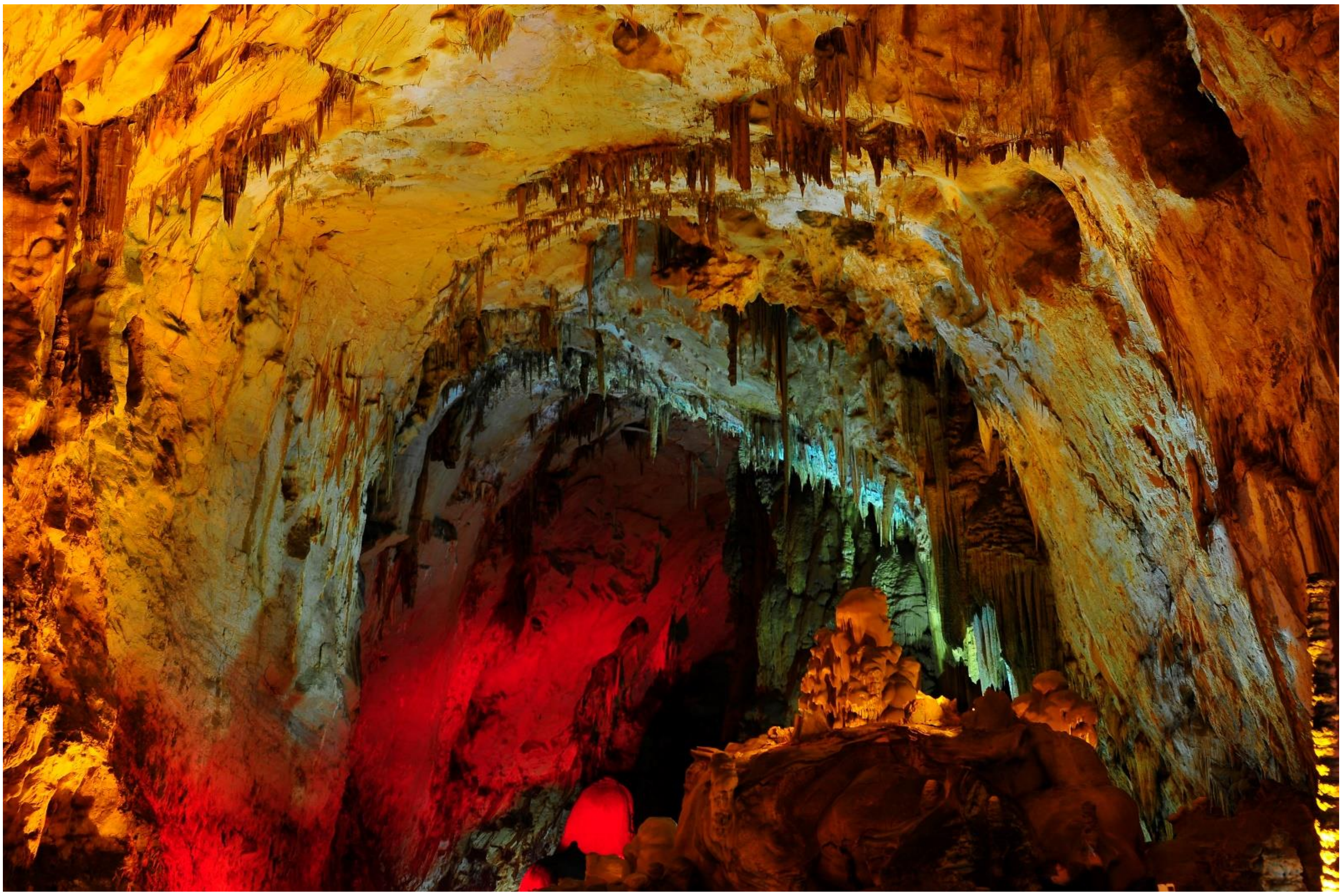

Zhijin cave, China.

Credit: Dong Ji / Getty Images

The subterranean lithic biome includes non-aquatic lithic systems beneath the earth's surface.

Sunlight is absent or of insufficient intensity to sustain photosynthesis. There is no standing water and moisture is supplied primarily by seepage through the substrate and may be lost by slow diffusion through the atmosphere to cave openings or by vertical or lateral seepage through the substrate. These physically stable systems exhibit low levels of environmental variability. Rarely, mass movements, for example rock falls, may re-organise the physical structure of subterranean ecosystems.

Subterranean ecosystems have truncated trophic structures with no photoautotrophs and few obligate predators. Heterotrophic microbes and invertebrates dominate the biota, while chemoautotrophs are the primary energy assimilators. Most have low metabolic rates and prolonged life histories in response to resource limitations, resulting in low overall productivity.

The subterranean biome includes dry caves and endolithic systems distributed throughout the earth's crust. Incursions of fresh or marine waters generate transitional biomes (SF1, SM1).

S1.1 Aerobic caves

Ecosystem properties: Dark subterranean air-filled voids support simple, low productivity systems. The trophic network is truncated and dominated by heterotrophs, with no representation of photosynthetic primary producers or herbivores. Diversity is low, comprising detritivores and their pathogens and predators, although there may be a few specialist predators confined to resource-rich hotspots, such as bat latrines or seeps. Biota include invertebrates (notably beetles, springtails, and arachnids), fungi, bacteria, and transient vertebrates, notably bats, which use surface-connected caves as roosts and breeding sites. Bacteria and fungi form biofilms on rock surfaces. Fungi are more abundant in humid microsites. Some are parasites and many are critical food sources for invertebrates and protozoans. Allochthonous energy and nutrients are imported via seepage moisture, tree roots, bats, and other winged animals. This leads to fine-scale spatial heterogeneity in resource distribution, reflected in patterns of biotic diversity and abundance. Autochthonous energy can be produced by chemoautotrophs. For example, chemoautotrophic Proteobacteria are prominent in subterranean caves formed by sulphide springs. They fix carbon through sulphide oxidation, producing sulphuric acid and gypsum residue in snottite draperies (i.e. microbial mats), accelerating chemical corrosion. The majority of biota are obligate subterranean organisms that complete their life cycles below ground. These are generalist detritivores and some are also opportunistic predators, reflecting the selection pressure of food scarcity. Distinctive traits include specialised non-visual sensory organs, reduced eyes, pigmentation and wings, elongated appendages, long lifespans, slow metabolism and growth, and low fecundity. Other cave taxa are temporary below-ground inhabitants, have populations living entirely above- or below-ground, or life cycles necessitating use of both environments. The relative abundance and diversity of temporary inhabitants decline rapidly with distance from the cave entrance. The specialist subterranean taxa belong to relatively few evolutionary lineages that either persisted as relics in caves after the extinction of above-ground relatives or diversified after colonisation by above-ground ancestors. Although diversity is low, local endemism is high, reflecting insularity and limited connectivity between cave systems.

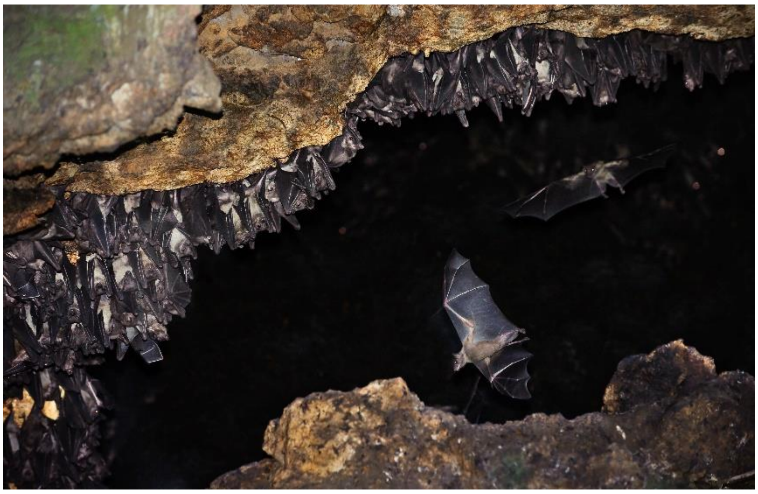

A cave colony of Egyptian fruit bats, Maramagambo Queen Elizabeth National Park, Uganda.
Credit: Marc Guitard / Getty Images

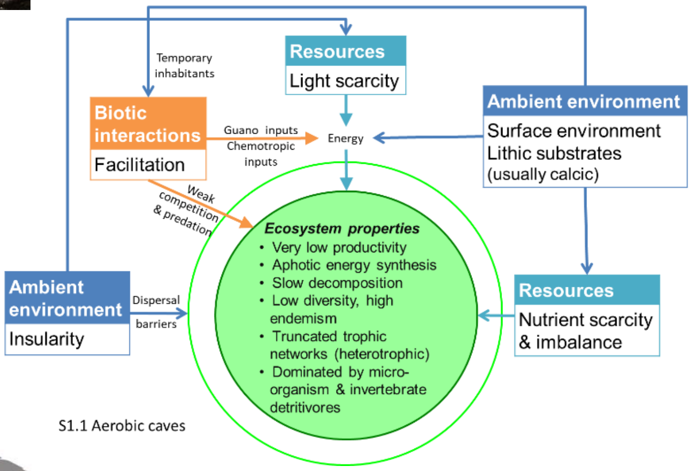

Ecological drivers: Most caves form from the chemical weathering of limestone, dolomite or gypsum, either from surface waters or from phreatic waters. Caves also derive from lava tubes and other substrates. Characteristics include the absence of light except at openings, low variability in temperature and humidity, and scarcity of nutrients. The high physical fragmentation of cave voids limits biotic connectivity and promotes insular evolution in stable conditions.

Distribution: Scattered worldwide, but mostly in the Northern Hemisphere, in limestone (map), basalt flows, and rarely in other lithic substrates.

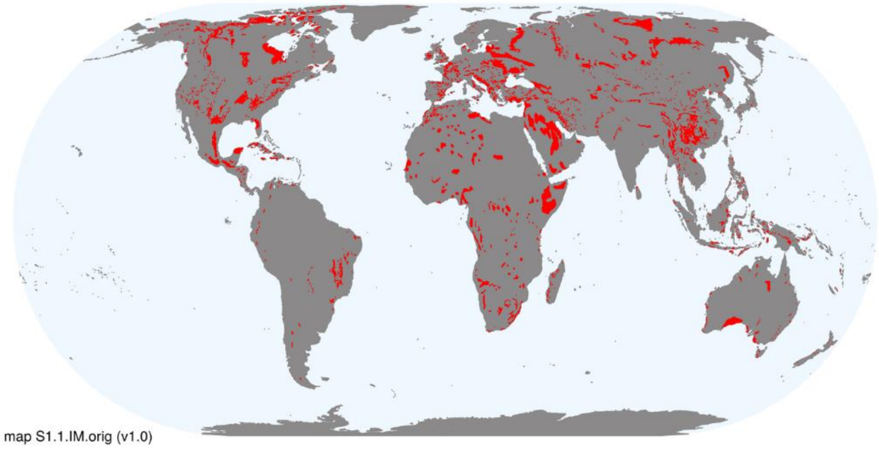

References:

Engel AS (2010) Microbial diversity of cave ecosystems. *Geomicrobiology: Molecular and environmental perspective* (Eds. LL Barton, M Mandl, A Loy), pp219-238. Springer, Dordrecht.

Gibert J, Deharveng L (2002) Subterranean ecosystems: a truncated functional biodiversity. *BioScience* 52, 473-481.

Map is for illustrative purposes only and does not support spatial analyses unless formally validated.

S1.2 Endolithic systems

Ecosystem properties: Lithic matrices and their microscopic cracks and cavities host microbial communities. Their very low productivity is constrained by the scarcity of light, nutrients, and water, and sometimes also by high temperatures. Diversity is low and the trophic network is truncated, supporting microscopic bacteria, archaea, viruses, and unicellular eukaryotes. Most are detritivores or lithoautotrophs, which derive energy, oxidants, carbohydrates, and simple organic acids from carbon dioxide, geological sources of hydrogen, and mineral compounds of potassium, iron and sulphur. Some fissures are large enough to support small eukaryotic predators such as nematodes. Photoautotrophs (i.e. cyanobacteria) are present only in the surface layers of exposed rocks. Sampling suggests that these systems harbour 95% of the world's prokaryote life (bacteria and archaea), with rocks below the deep oceans and continents containing similar densities of cells and potentially accounting for a significant proportion of sequestered carbon. Endolithic microbes are characterised by extremely slow reproductive rates, especially in deep sedimentary rocks, which are the most oligotrophic substrates. At some depth within both terrestrial and marine substrates, microbes are sustained by energy from organic matter that percolates through fissures from surface systems. In deeper or less permeable parts of the crust, however, lithoautotrophic microbes are the primary energy synthesisers that sustain heterotrophs in the food web. Methanogenic archaea and iron-reducing bacteria appear to be important autotrophs in sub-oceanic basalts. All endolithic microbes are characterised by slow metabolism and reproduction rates. At some locations they tolerate extreme pressures, temperatures (up to 125°C) and acidity (pH<2), notably in crustal fluids. Little is currently known of endemism, but it may be expected to be high based on the insularity of these ecosystems.

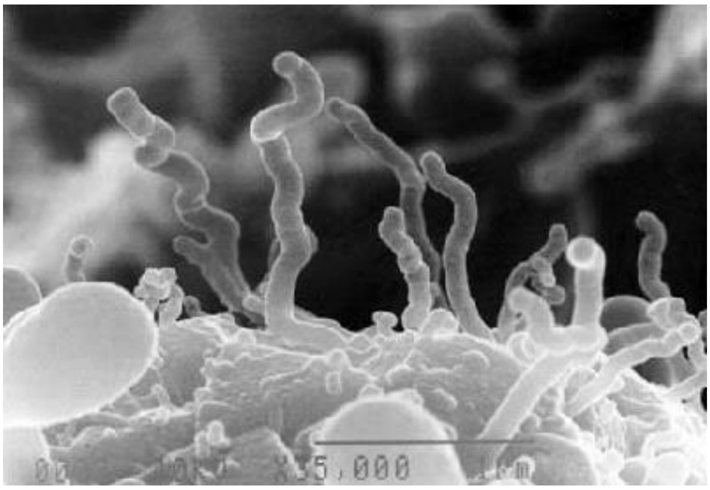

Endolithic nanobacteria in sandstone, Australia.
Credit: Philippa Uwins

Ecological drivers: Endolithic systems are characterised by a lack of light, a scarcity of nutrients, and high pressures at depth. Temperatures vary within the crust from <20°C up to 125°C, but show little temporal variation. The chemical properties and physical structure of lithic matrices influence the supply of resources and the movement of biota. Stable cratonic massifs have minimal pore space for microbial occupation, which is limited to occasional cracks and fissures. Sedimentary substrates offer more space, but nutrients may be scarce, while fluids in basic volcanic and crustal rocks have more abundant nutrients. Chemical and biogenic weathering occurs through biogenic acids and other corrosive agents. The matrix is mostly stable, but disturbances include infrequent and spatially variable earthquakes and volcanic intrusions.

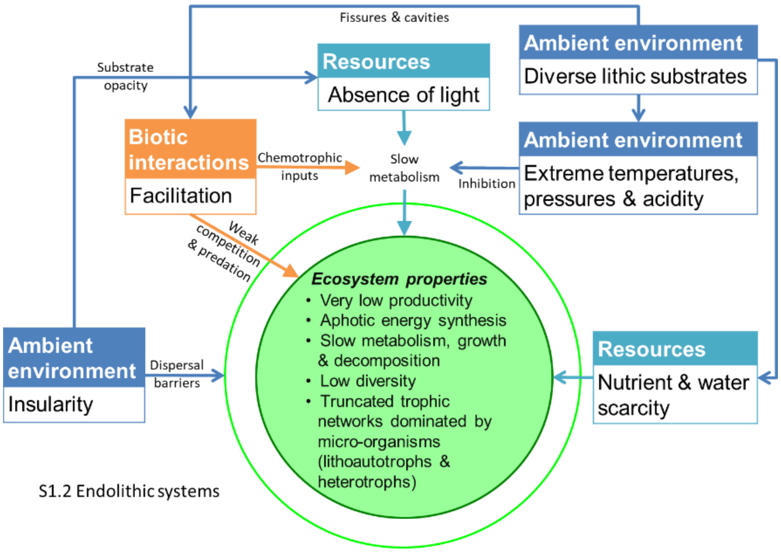

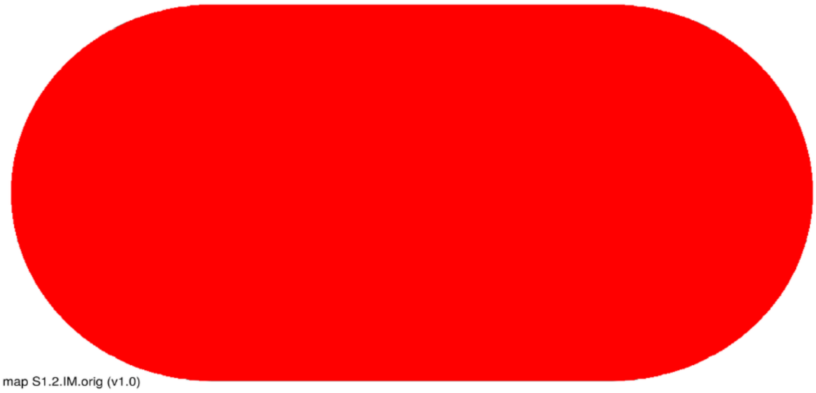
map S1.2.IM.orig (v1.0)

Distribution: Throughout the earth's crust, from surface rocks to a predicted depth of up to 4–4.5 km below the land surface and 7–7.5 km below ocean floors.

References:

Edwards KJ, Becker K, Colwell F (2012) The deep, dark energy biosphere: intraterrestrial life on earth. *Annual Review of Earth and Planetary Sciences* 40, 551–568.

Map is for illustrative purposes only and does not support spatial analyses unless formally validated.

S2. Anthropogenic subterranean voids biome

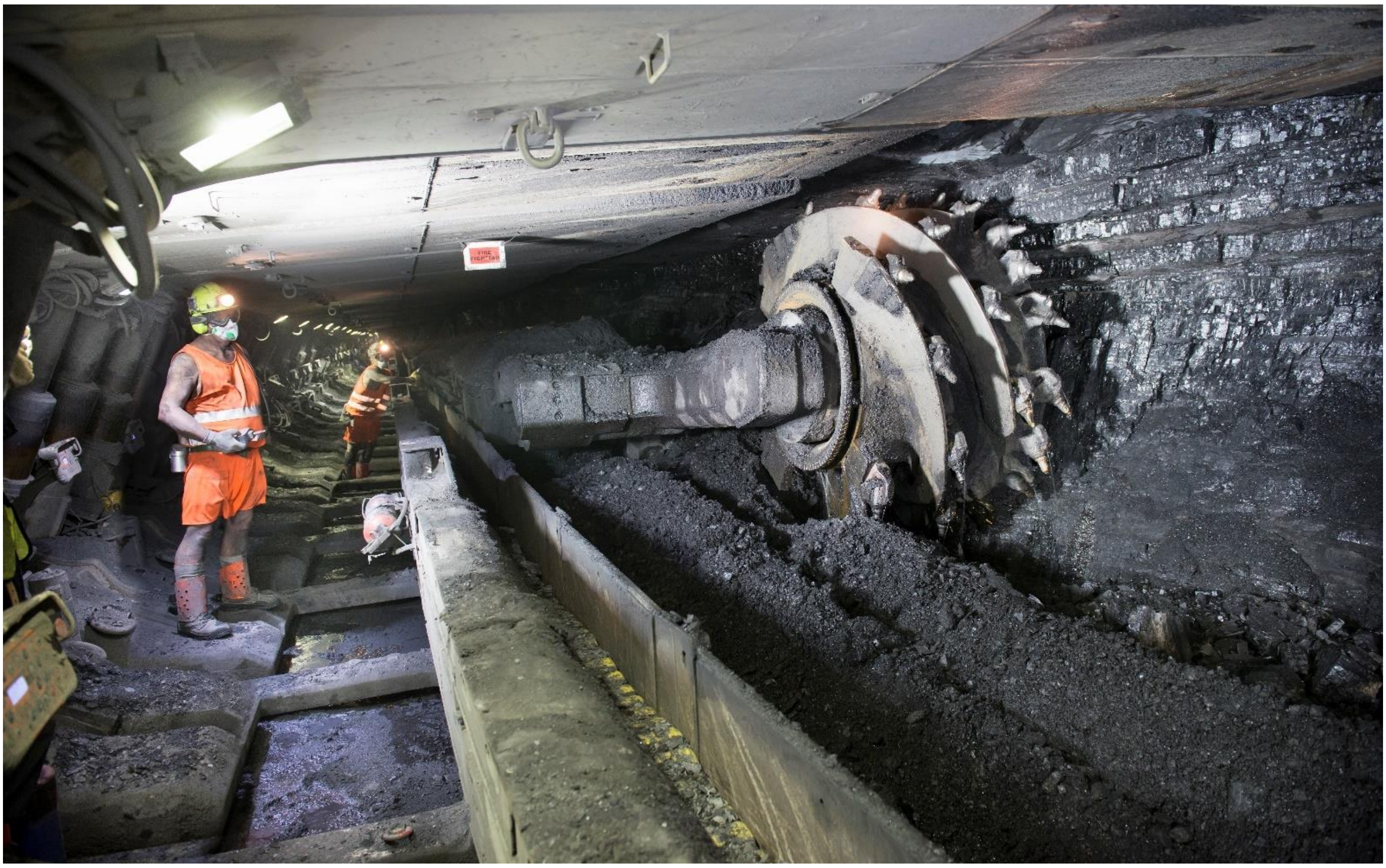

Underground coal mine.

Credit: Monty Rakusen / Getty Images

The Anthropogenic subterranean voids biome includes a single functional group of ecosystems that owe their genesis to excavation by humans. They include underground mines, transport tunnels, tombs, defence and energy installations, and other infrastructure. Most are very recent ecosystems constructed with earth-moving machinery during the industrial era, but some were constructed manually up to several millennia ago.

Productivity is low and energy generally comes from allochthonous sources via connections to the surface, either by atmospheric diffusion or seepage, but some energy is contributed by chemoautotrophic microbes. While sunlight is absent or highly diffuse, some active voids are artificially lit and this may provide sufficient energy to sustain algal autotrophs.

Trophic webs are simple and dominated by opportunistic microbes and invertebrates introduced by machinery or directly by humans, or else colonising spontaneously through openings to the surface. The latter may include small mammals that use the voids as refuges or breeding sites. Microbes from external and endolithic sources rapidly colonise newly exposed lithic surfaces and create biofilms that support detritivores and enhance substrate weathering.

The stability of artificial subterranean voids varies depending on their substrate and management, with some prone to collapse and structural change after active use ceases.

Contributors: DA Keith, TM Iliffe, V Gerovasileiou, B Gonzalez, D Brankovits, A Martínez García

S2.1 Anthropogenic subterranean voids

Ecosystem properties: These low-productivity systems in subterranean air-filled voids are created by excavation. Although similar to Aerobic caves (S1.1), these systems are structurally simpler, younger, more geologically varied, and much less biologically diverse with few evolutionary lineages and no local endemism. Low diversity, low endemism, and opportunistic biotic traits stem from founder effects related to their recent anthropogenic origin (hence few colonisation events and little time for evolutionary divergence), as well as low microhabitat niche diversity due to the simple structure of void walls compared to natural caves. The trophic network is truncated and dominated by heterotrophs, usually with no representation of photosynthetic primary producers or herbivores. Generalist detritivores and their pathogens and predators dominate, although some specialists may be associated with bat dung deposits. Biota include invertebrates (notably beetles, springtails, and arachnids), fungi, bacteria, and transient vertebrates, notably bats, which use the voids as roosts and breeding sites. Bacteria and fungi form biofilms on void surfaces. Many are colonists of human inoculations, with some microbes identified as "human-indicator bacteria" (e.g. E. coli, Staphylococcus aureus, and high-temperature Bacillus spp.). Fungi are most abundant in humid microsites. Some are parasites and many are critical food sources for invertebrates and protozoans. Sources of energy and nutrients are allochthonous, imported by humans, bats, winged invertebrates, other animals, and seepage moisture. Many taxa have long life pans, slow metabolism and growth, and low fecundity, but lack distinctive traits found in the biota of natural caves. Some are temporary below-ground inhabitants, have populations that live entirely above- or below-ground, or have life cycles necessitating the use of both environments.

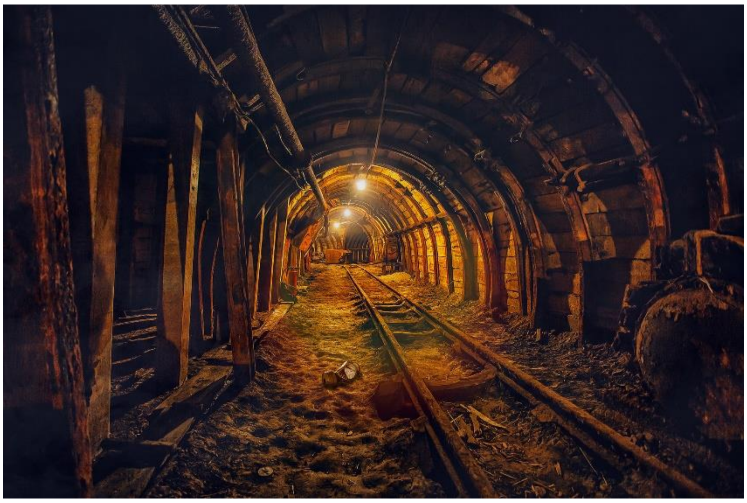

Underground mining tunnel.
Credit: Maxim / Adobe Stock

Ecological drivers: Excavations associated with tunnels, vaults and mines. While some are abandoned, others are continuously accessed by humans, enhancing connectivity with the surface, resource importation, and biotic dispersal. Substrates include a range of rock types as well as artificial surfaces on linings and debris piles. Air movement varies from still to turbulent (e.g. active train tunnels). Light is absent except at openings and where artificial sources are maintained by humans, sometimes supporting algae (i.e. lampenflora). Humidity and temperature are relatively constant, and nutrients are scarce except where enriched by human sources.

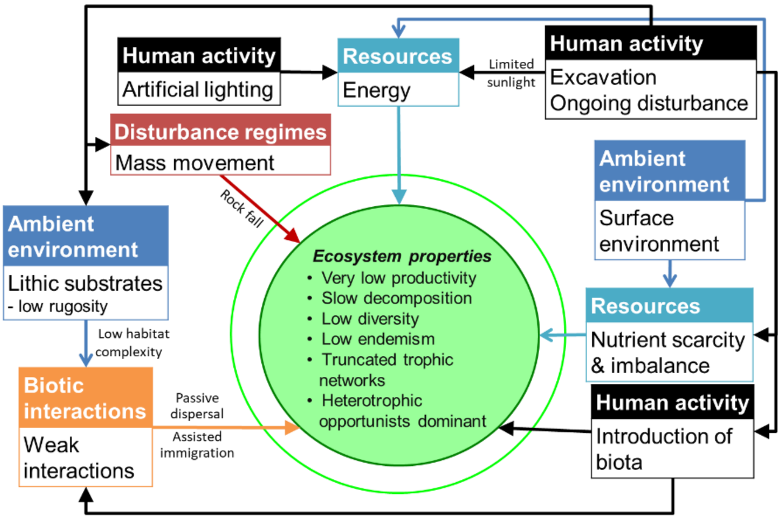

S2.1 Anthropogenic subterranean voids

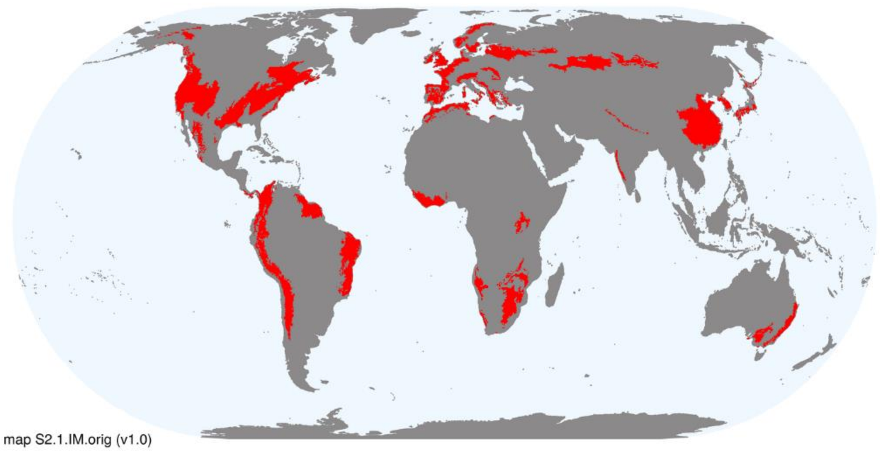

Distribution: Scattered worldwide, but mostly associated with urban centres, transit corridors, and industrial mines.

References:

Engel AS (2010) Microbial diversity of cave ecosystems. In: *Geomicrobiology: Molecular and environmental perspective* (Eds. LL Barton, M Mandl, A Loy), pp219-238. Springer, Dordrecht.

Gibert J, Deharveng L (2002) Subterranean ecosystems: a truncated functional biodiversity. *BioScience* 52, 473-481.

Map is for illustrative purposes only and does not support spatial analyses unless formally validated.

SF1. Subterranean freshwaters biome

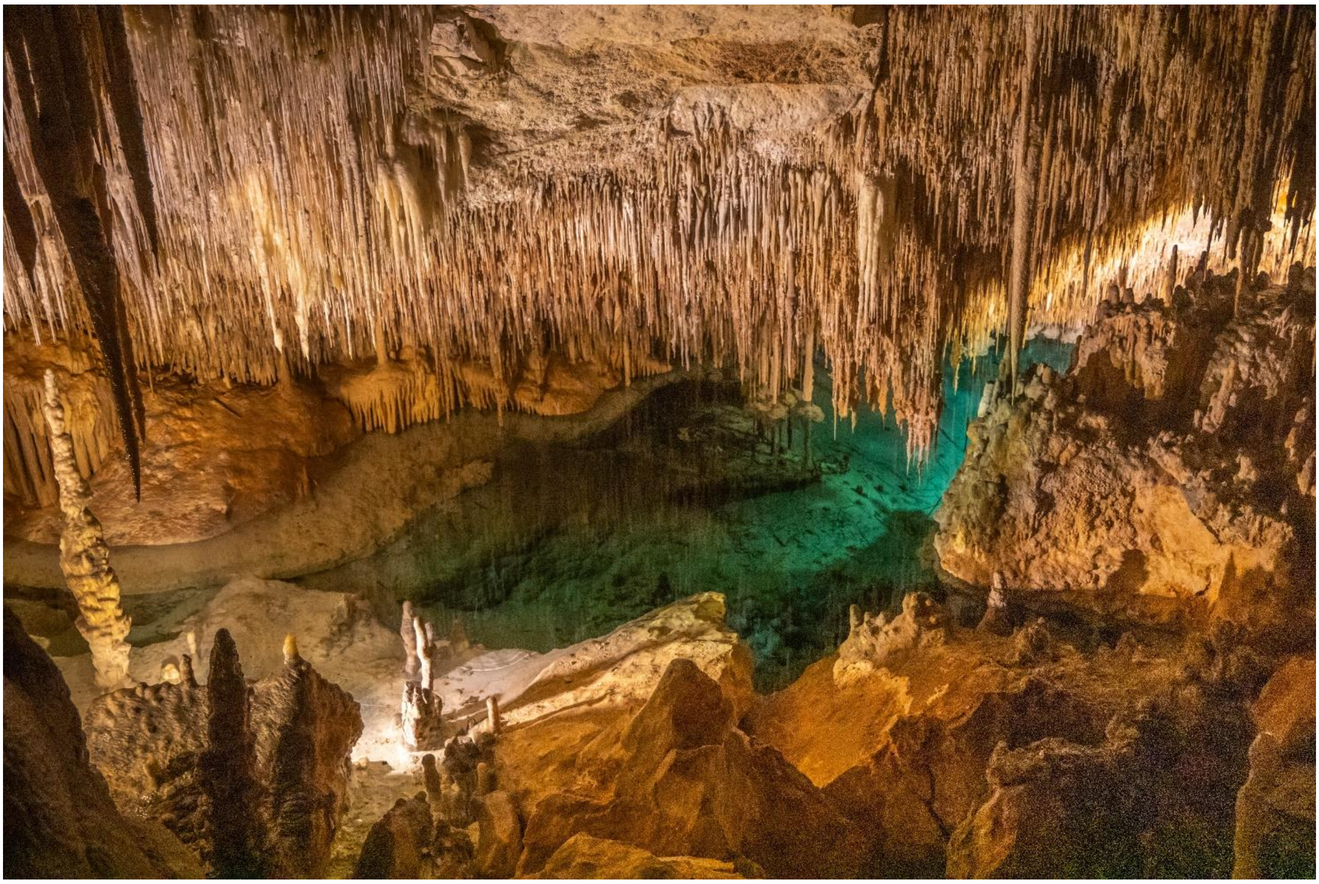

Cave lake, Mallorca Island, Spain.

Credit: Artur Debat / Getty Images

The Subterranean freshwaters biome includes streams, small lakes and aquifers beneath the earth's surface and potentially has the largest volume of water of all the freshwater biomes.

In the absence of sunlight, these ecosystems rely on allochthonous energy sourced from surface ecosystems via connected waters and in situ chemoautotrophs. Depending on the mode of connectivity to the surface, water flow-through varies from extremely rapid to slow. Highly connected subterranean streams in monsoonal climates undergo seasonal flooding and drying cycles. In contrast, paleo-aquifers are characterised by slow, low-variability seepage over millennial time scales.

Inflowing water is the principal source of dissolved oxygen and mineral nutrients, although some nutrients are liberated by in situ weathering of lithic substrates. The water regime largely determines environmental variability in subterranean freshwaters, but these systems may occasionally be influenced by mass movements.

The trophic structure of subterranean waters is typically truncated, although photosynthetically inactive algae and higher-plant propagules may be transient occupants in systems that are connected to the surface. Chemoautotrophic and heterotrophic microbes in biofilms and the water column dominate the trophic web, supporting small invertebrate detritivores and predators. Small predatory fish may occur in streams and lakes, where voids in the subsurface are of sufficient size. Productivity, metabolic rates, life histories and the diversity of the biota all reflect resource scarcity but may vary depending on water source. Insular systems exhibit high levels of endemism.

Contributors: DA Keith, GC Hose

SF1.1 Underground streams and pools

Ecosystem properties: Subterranean streams, pools, and aquatic voids (flooded caves) are low-productivity systems devoid of light. The taxonomic and functional diversity of these water bodies is low, but they may host local endemics, depending on connectivity with surface waters and between cave systems. The truncated trophic network is entirely heterotrophic, with no photosynthetic primary producers or herbivores. Detritivores and their predators are dominant, although a few specialist predators may be associated with resource-rich hotspots. Microbial mats composed of bacteria and aquatic fungi covering submerged rock surfaces are major food sources for protozoans and invertebrates. Other biota include planktonic bacteria, crustaceans, annelids, molluscs, arachnids, and fish in larger voids. Chemoautotrophic proteobacteria are locally abundant in sulphur-rich waters fed by springs but not widespread. Obligate denizens of subterranean waters complete their life cycles entirely below ground and derive from relatively few evolutionary lineages. These make up a variable portion of the biota, depending on connectivity to surface waters. Most species are generalist detritivores coexisting under weak competitive interactions. Some are also opportunistic predators, reflecting selection pressures of food scarcity. Distinctive traits include the absence of eyes and pigmentation, long lifespans, slow metabolism and growth rates, and low fecundity. Less-specialised biota include taxa that spend part of their life cycles below ground and part above, as well as temporary below-ground inhabitants. Transient vertebrates occur only in waters of larger subterranean voids that are well connected to surface streams with abundant food.

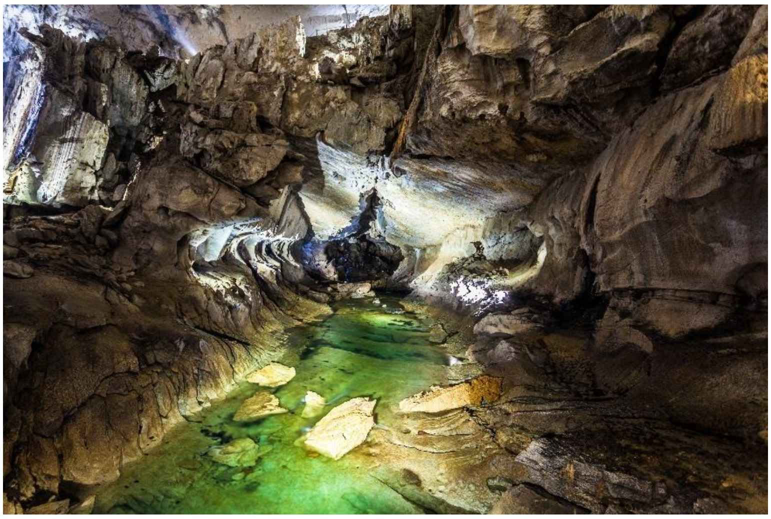

Underground stream, Gunung Mulu National Park, Borneo, Malaysia. Inset: Eyeless cave fish, Mammoth Cave National Park, Kentucky USA.
Credit: Zodebala / Getty Images

Ecological drivers: Most caves form from chemical weathering of soluble rocks such as limestone or dolomite, and others include lava tunnels. Cave waters are devoid of light, typically low in dissolved oxygen nutrients, and food, and exhibit low variability in temperature. Water chemistry reflects substrate properties (e.g. high Calcium levels in limestone voids). Resource supply and biotic dispersal depend on connectivity with surface waters, flow velocity and turbulence. In the absence of light, surface-connected streams are major allochthonous sources of energy and nutrients. Disconnected systems are the most biologically insular and oligotrophic, and may also be limited by nutrient imbalance. These features promote insular evolution in stable conditions.

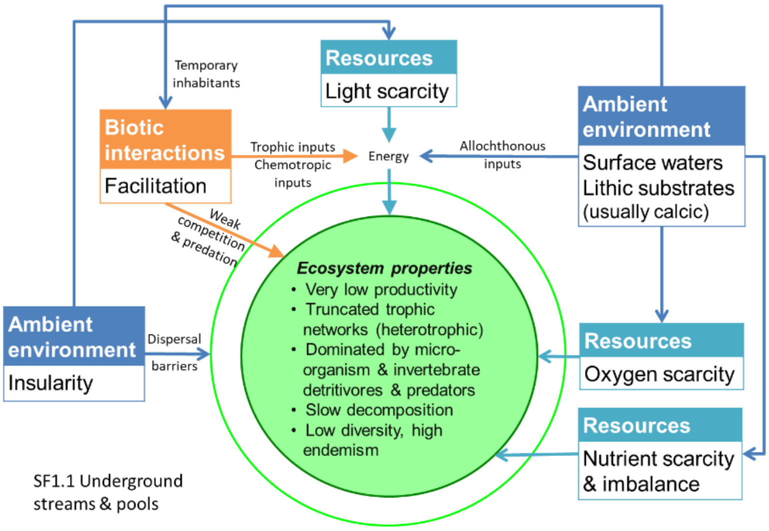

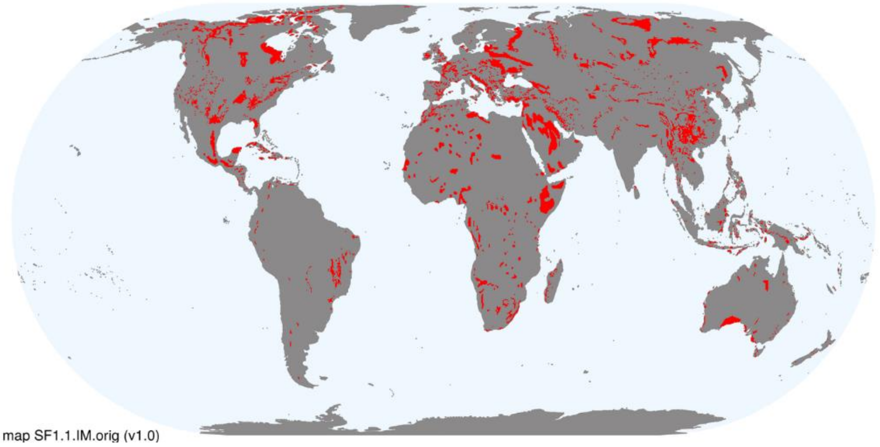

Distribution: Scattered worldwide, mostly in the Northern Hemisphere in limestone and more rarely in basalt flows and other lithic substrates.

References:

Gibert J, Deharveng L (2002) Subterranean ecosystems: a truncated functional biodiversity. *BioScience* 52, 473-481.

Map is for illustrative purposes only and does not support spatial analyses unless formally validated.

SF1.2 Groundwater ecosystems

Ecosystem properties: These low-productivity ecosystems are found within or below groundwater (phreatic) zones. They include aquifers (underground layers of water-saturated permeable rock or unconsolidated gravel, sand, or silt) and hyporheic zones beneath rivers and lakes (i.e. where shallow groundwater and surface water mix). Diversity and abundance of biota decline with depth and connectivity to surface waters, as do nutrients (e.g. most meiofauna is limited to 100m depth). Microbial communities are functionally diverse and invertebrate taxa exhibit high local endemism where aquifers are poorly connected. Trophic networks are truncated and comprised almost exclusively of heterotrophic microbes and invertebrates. Chemoautotrophic bacteria are the only source of autochthonous energy. Herbivores only occur where plant material enters groundwater systems (e.g. in well-connected hyporheic zones). Microbes and their protozoan predators dwell on particle surfaces rather than in pore water. They play key roles in weathering and mineral formation, engineer chemically distinctive microhabitats through redox reactions, and are repositories of Carbon, Nitrogen and Phosphorus within the ecosystem. Meio-faunal detritivores and predators transfer Carbon and nutrients from biofilms to larger invertebrate predators such as crustaceans, annelids, nematodes, water mites, and beetles. These larger trophic generalists live in interstitial waters, either browsing on particle biofilms or ingesting sediment grains, digesting their surface microbes, and excreting 'cleaned' grains. They have morphological and behavioural traits that equip them for life in dark, resource-scarce groundwater where space is limited. These include slow metabolism and growth, long lifespans without resting stages, low fecundity, lack of pigmentation, reduced eyes, enhanced non-optic sensory organs, and elongated body shapes with enhanced segmentation. Much of the biota belongs to ancient subterranean lineages that have diverged sympatrically within aquifers or allopatrically from repeated colonisations or aquifer fragmentation.

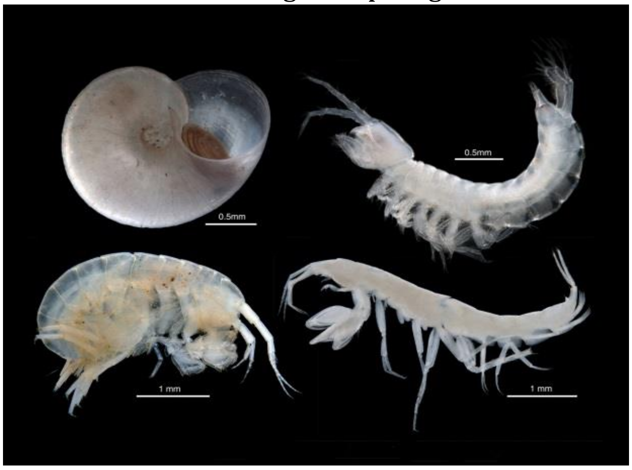

Stygofauna from the Pilbara, Western Australia.
Credit: Jane McRae / Western Australian Museum

Ecological drivers: Groundwater ecosystems are characterised by a scarcity of nutrients, Carbon, dissolved oxygen and free space, and an absence of light. They occur within basin fill or other porous geological strata. Groundwater flow, pore size, interstitial biogeochemistry, and hydrological conductivity to adjacent aquifers and surface waters determine ecosystem properties. Subsurface water residence times vary from days in shallow, well-connected, coarse-grained hyporheic systems to thousands of years in deep, poorly connected aquifers confined between impermeable rock strata. Lack of connectivity promotes insularity and endemism as well as reductive biogeochemical processes that influence the availability of food and nutrients.

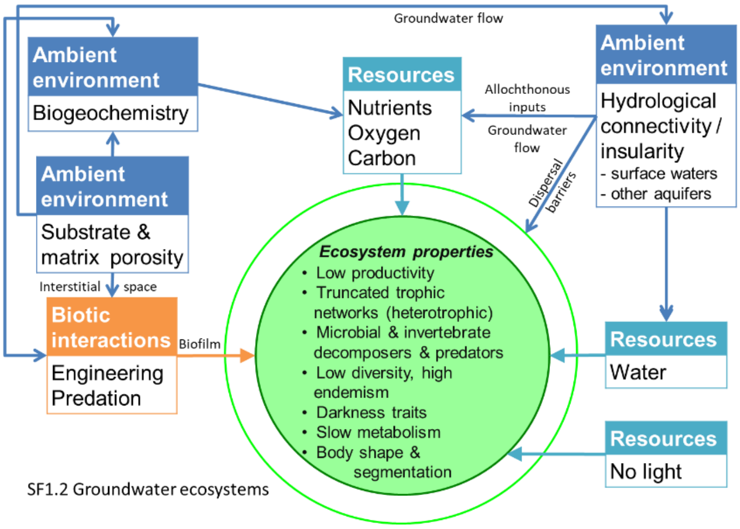

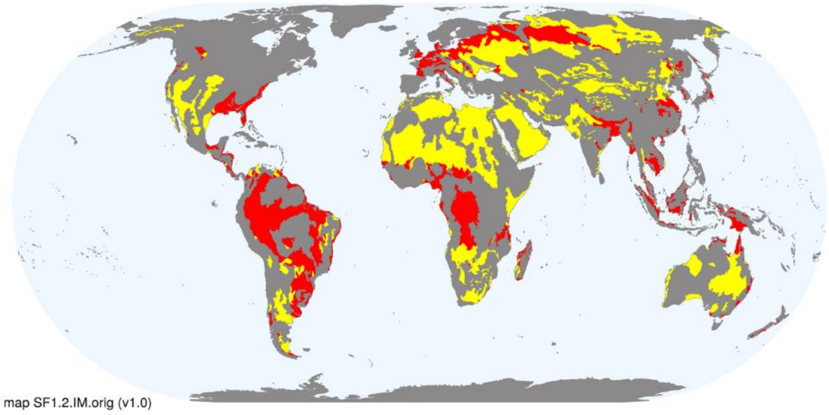

Distribution: Globally distributed. Map shows only the major groundwater basins by recharge rates.

References:

Danielopol DL, Griebler C, Gunatilaka A, Notenboom J (2003) Present state and future prospects for groundwater ecosystems. *Environmental Conservation* 30: 104-130.

Hancock PJ Boulton AJ, Humphreys WF (2005) Aquifers and hyporheic zones: towards an understanding of groundwater. *Hydrogeology Journal* 13: 98–111.

Struckmeier W, Richts A (2008) *Groundwater resources of the world* BGR Hannover / UNESCO Paris.

Map is for illustrative purposes only and does not support spatial analyses unless formally validated.

SF2. Anthropogenic subterranean freshwaters biome

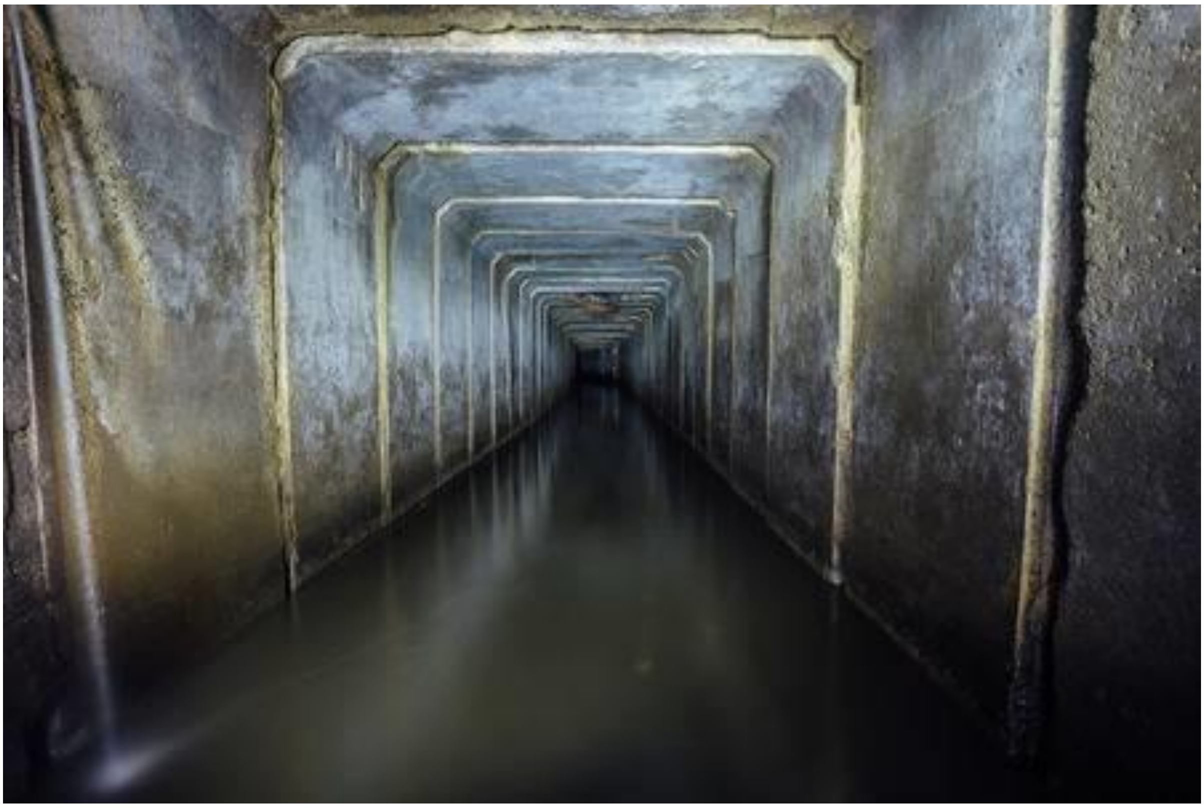

Underground river in sewer tunnel under Voronezh, Russia.

Credit: mulderphoto / 123RF.com

The Artificial subterranean freshwaters biome includes aquatic systems in underground canals, drains, sewers, water pipes, and flooded mines constructed by humans. These are usually well connected to surface waters.

The availability of resources is largely a function of source waters and the water regime, which varies from permanent to intermittent with low to high flow velocity or, in the case of flooded mines, negligible flow. Sunlight is absent or, if it diffuses through vents and portals (as in some canals), it is generally too dim to support photosynthesis. Algae may nonetheless be transported through these systems depending on the water of source.

Although primary productivity is low and energy is supplied from allochthonous sources, secondary productivity by heterotrophic microbes in biofilms and in the water column may be high in sewers and drains where organic Carbon, nutrients, and dissolved oxygen are abundant. This may support several tiers of detritivores and predators including microscopic invertebrates, macro-invertebrates, and small vertebrates including rodents and fish. Anaerobic bacteria may be important components of the trophic network where organic Carbon and nutrients are abundant but dissolved oxygen is scarce due to either low aeration or high microbial activity.

In water supply pipes, low levels of organic carbon and nutrients exacerbate constraints on productivity imposed by the absence of light. Trophic webs within pipes are truncated and simple, and the mostly transitory biota reflects that of source waters.

Contributors: DA Keith, TM Iliffe, V Gerovasileiou, B Gonzalez, D Brankovits, A Martínez García

SF2.1 Water pipes and subterranean canals

Ecosystem properties: Constructed subterranean canals and water pipes are dark, low-productivity systems acting as conduits for water, nutrients, and biota between artificial or natural freshwater ecosystems. Energy sources are therefore entirely or almost entirely allochthonous from surface systems. Although similar to underground streams (S2.1), these systems are structurally simpler, younger, and less biologically diverse with few evolutionary lineages and no local endemism. Diversity and abundance are low, often resulting from the accidental transport of biota from source to sink ecosystems. Trophic networks are truncated, with very few or no primary producers and no vertebrate predators except incidental transients. The majority of the resident heterotrophic biota are bacteria, aquatic fungi, and protists living in biofilms covering mostly smooth artificial surfaces or cut rock faces. Biofilms constitute food sources for detritivores and predators, including protozoans and planktonic invertebrates as well as filter feeders such as molluscs. The structure of the biofilm community varies considerably with hydraulic regime, as does the biota in the water column. Transient vertebrates, notably fish, occupy well-connected ecosystems with abundant food and predominantly depend on transported nutrients and prey. A range of organisms may survive in these environments but only some maintain reproductive populations. All biota are capable of surviving under no or low light conditions, at least temporarily while in transit. Other traits vary with hydraulic regimes and hydrochemistry, with physiological tolerance to toxins important in highly eutrophic, slow-flowing drains and tolerance to low nutrients and turbulence typical in high-velocity minerotrophic water pipes.

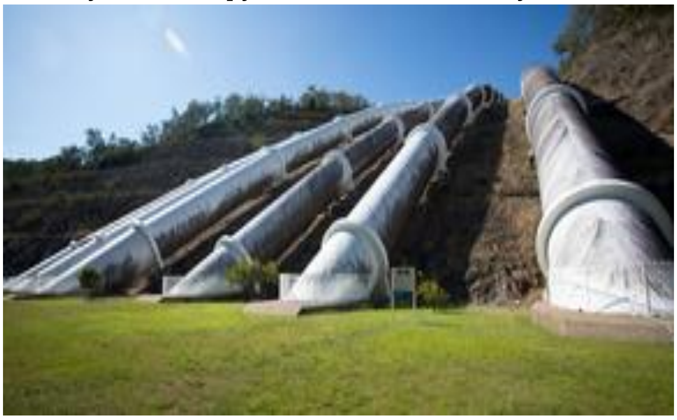

Water pipes in the Snowy Mountains, Australia.
Credit: Neale Cousland / Shutterstock

Ecological drivers: Subterranean canals and water pipes are engineered structures designed to connect and move waters between artificial (or more rarely natural) sources. They are united by an absence of light and usually low oxygen levels and low variability in temperatures, but hydraulic regimes, nutrient levels, water chemistry, flow and turbulence vary greatly among ecosystems. Water supply pipes are extreme oligotrophic systems with rapid flow, high turbulence, low nutrients and low connectivity to the atmosphere, often sourced from de-oxygenated water at depth within large reservoirs (F3.1). In contrast, subterranean wastewater or stormwater canals have slower, more intermittent flows, low turbulence, and very high nutrient levels and chemical pollutants including toxins. Many of these eutrophic systems have an *in situ* atmosphere, but dissolved oxygen levels are very low in connection with high levels of dissolved organic Carbon and microbial activity.

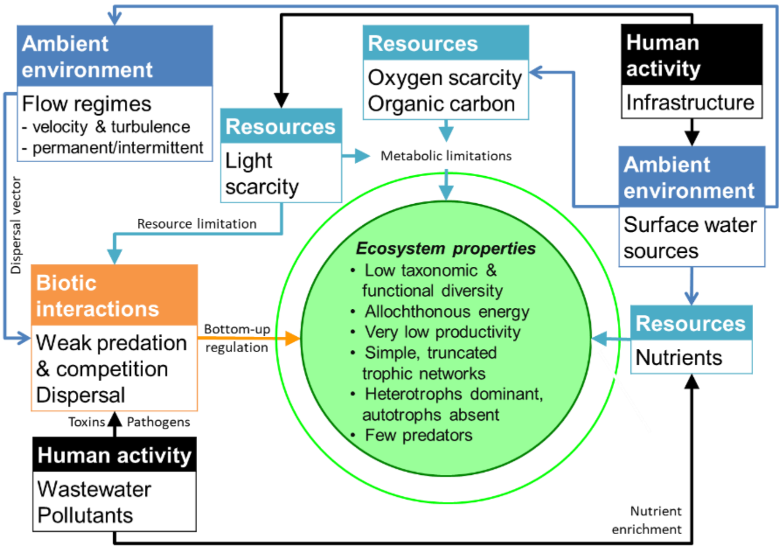

SF2.1 Water pipes & subterranean canals

Distribution: Common in landscapes with urban or industrial infrastructure including water supply and sewerage reticulation systems, hydroelectricity, irrigation, and other intensive agricultural industries.

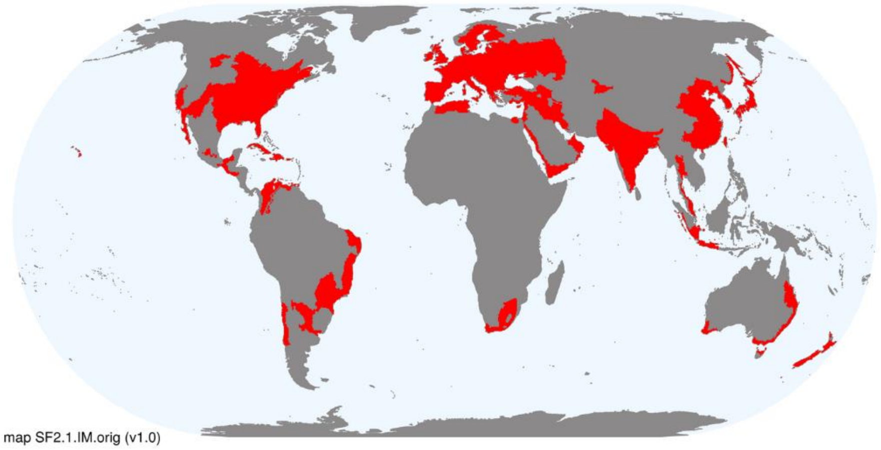

References:

Douterelo I, Sharpe RL, Boxall JB (2013) Influence of hydraulic regimes on bacterial community structure and composition in an experimental drinking water distribution system. *Water Research* 47, 503-516.

Map is for illustrative purposes only and does not support spatial analyses unless formally validated.

SF2.2 Flooded mines and other voids

Ecosystem properties: Abandoned and now flooded underground mines frequently contain extensive reservoirs of geothermally warmed groundwater, colonized by stygobitic invertebrates from nearby natural subterranean habitats. A fraction of the biota is likely to have been introduced by mining activities. A lack of light excludes photoautotrophs from these systems and low connectivity limits inputs from allochthonous energy sources. Consequently, overall productivity is low, and is likely to depend on chemoautrophic microbes (e.g. sulfate-reducing bacteria) as sources of energy. Few studies have investigated the ecology of the aquatic biota in quasi-stagnant water within mine workings, but trophic networks are truncated and likely to be simple, with low diversity and abundance at all trophic levels, and no endemism. Most of the resident heterotrophic biota are bacteria, aquatic fungi, and protists living in biofilms on artificial surfaces of abandoned infrastructure, equipment or cut rock faces. Extremophiles are likely to dominate in waters that are highly acidic or with high concentrations of heavy metals or other toxins. Micro-invertebrates are most likely to be the highest-level predators. Some voids may have simple assemblages of macroinverterbates, but few are likely to support vertebrates unless they are connected with surface waters that provide a means of colonization.

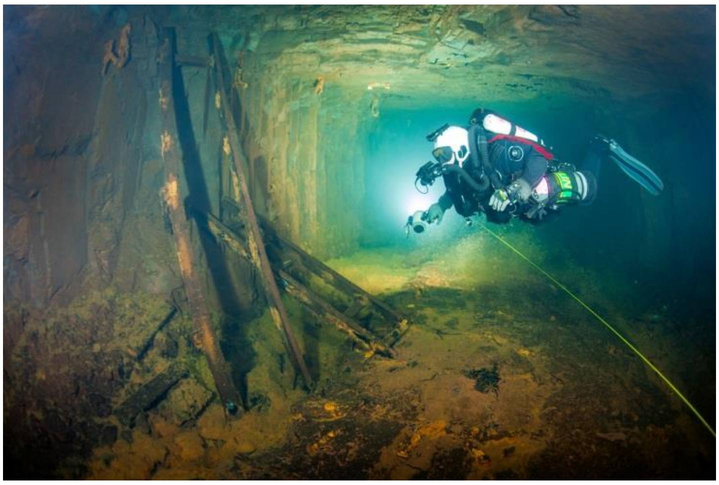

Flooded iron ore mine, Bell Island, Newfoundland, Canada.
Credit: Jill Heinerth

Ecological drivers: Like all subterranean ecosystems, light is absent or extremely dim in flooded mines. Unlike subterranean canals and pipes (SF2.1), mine waters are quasi-stagnant and not well connected to surface waters. During mine operation, water is pumped out of the mine forming a widespread cone of water table depression, with oxidation and hydrolysis of exposed minerals changing groundwater chemistry. When mines close and dewatering ceases, water table rebounds and the voids often flood. Some voids are completely inundated, while others retain a subterranean atmosphere, which may or may not be connected to the surface. Further changes in water chemistry occur after flooding due to dissolution and flushing of the oxidation products. Water is often warm due to geothermal heating. After inundation has stabilised, seepage and mixing may be slow, and stratification creates strong gradients in oxygen and solutes. Waters are acidic in most flooded mines. The ionic composition varies depending on mineralogy of the substrate, but ionic concentrations are typically high, and often contain heavy metals at levels toxic to some aquatic biota. Acid mine drainage is a common cause of pollution in surface rivers and streams, where it seeps to the surface.

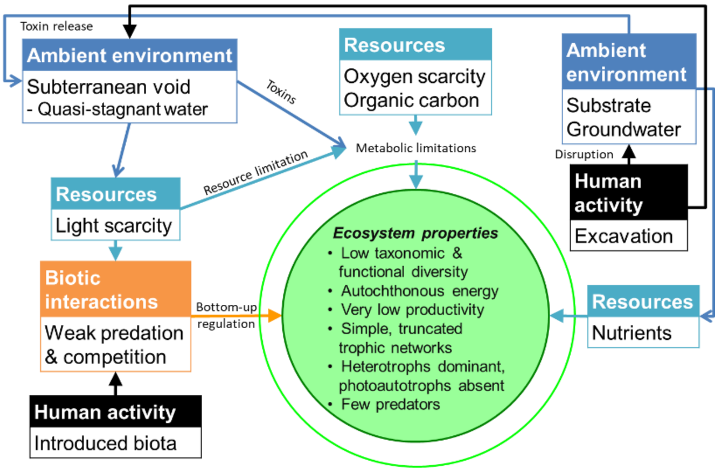

SF2.2 Flooded mines and other voids

Distribution: Common in in many mineral rich regions of the world.

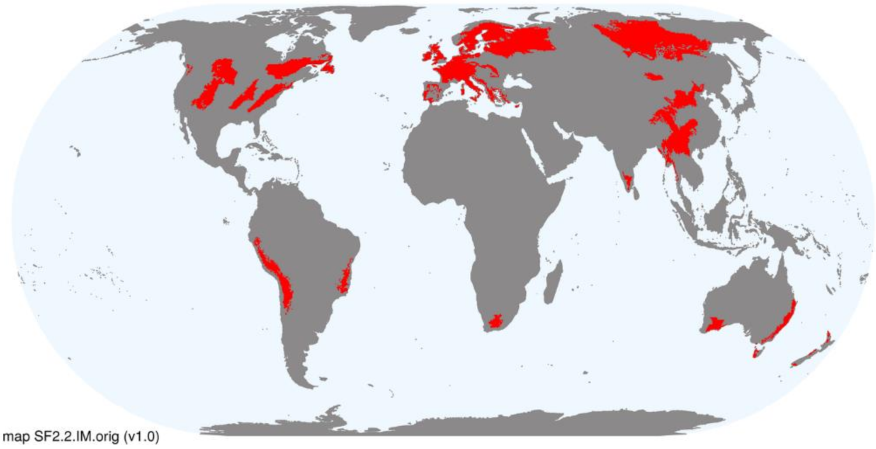

map SF2.2.IM.orig (v1.0)

References:

Nuttall, CA; Younger, PL (2004) Hydrochemical stratification in flooded underground mines: an overlooked pitfall. *Journal of Contaminant Hydrology* 69, 101-114.

Roesler AJ, Gammons CH, Druschel GK et al. (2007) Geochemistry of flooded underground mine workings influenced by bacterial sulfate reduction. *Aquatic Geochemistry* 13, 211–235.

Wright IA, Paciuszkiewicz K, Belmer N (2018) Increased water pollution after closure of Australia's longest operating underground coal mine: a 13-month study of mine drainage, water chemistry and river ecology. *Water, Air, & Soil Pollution* 229, 55.

Map is for illustrative purposes only and does not support spatial analyses unless formally validated.

SM1. Subterranean tidal biome

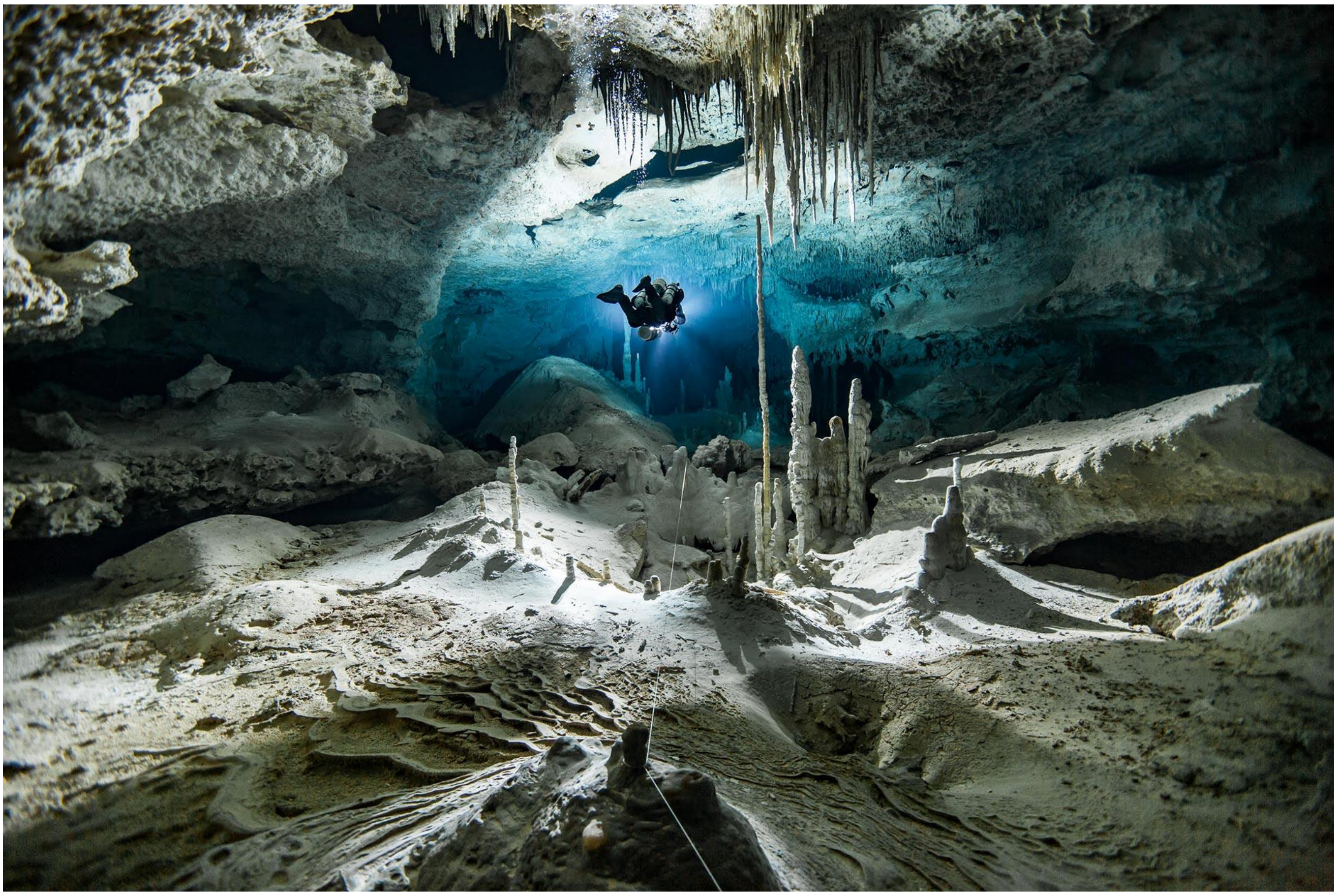

Sac Actun anchialine cave system, Quintana Roo, Mexico.

Credit: Alison Perkins

The subterranean tidal biome includes coastal pools and subterranean voids with a partially or entirely submerged connection to marine waters. Like all other subterranean ecosystems, sunlight is absent or too dim to sustain photosynthesis.

Marine shelf ecosystems (M1), terrestrial aquifers (SF1), and surface coastal systems (T1, T2, T3, T4, T5, T6, MT1) connected to these subterranean systems are their sources of allochthonous energy, nutrients, and oxygenation. Food and energy availability are influenced by in situ microbial processing (biogeochemical transformation) of these allochthonous organic matter inputs.

The marine interface, a typical feature of coastal aquifers and subterranean estuaries, also generates a marked salinity gradient in the primary zone of biogeochemical cycling. In carbonate and volcanic geologies, the salinity gradient can often be observed in the flooded pools, voids, and caves as a halocline (a sharp salinity gradient in the water column), which is not present in other subterranean environments.

In comparison to other subterranean ecosystems, diverse assemblages of chemoautotrophic and heterotrophic microbes, as well as scavengers, filter feeders, and predators. Physiological traits enabling osmotic regulation allow some species to transit across haloclines between the fresh- and saline waters. In dark sections of the subterranean marine systems where photoautotrophs are absent, trophic webs are truncated. Some of the subterranean marine biota belong to lineages otherwise restricted to the deep sea floor (M3) and share traits with those in other low-productivity, dark biomes including depigmentation, reduced visual organs, increased tactile and chemical sensitivity, low fecundity, long lifespans, and slow metabolism and growth rates.

Tides are an important means of hydrological mixing, resource flux, biotic dispersal, and perturbation. In subterranean tidal systems with more direct connections to the sea, marine suspension feeders, particularly sponges and other sessile invertebrates, are dominant. Farther into marine and anchialine caves where tidal flushing and water exchange diminishes or disappears, the fauna consists of stygobitic crustaceans, annelids and several other faunal groups (e.g. strictly subterranean aquatic fauna that complete their entire life in this environment).

Contributors: TM Iliffe, D Brankovits, V Gerovasileiou, B Gonzalez, A Martínez García, DA Keith

SM1.1 Anchialine caves

Ecosystem properties: Anchialine caves contain bodies of saline or brackish waters with subterranean connections to the sea. Since virtually all anchialine biota are marine in origin, these caves have a larger and more diverse species pool than underground freshwaters. The trophic network is truncated and dominated by heterotrophs (scavenging and filter-feeding detritivores and their predators), with photosynthetic primary producers and herbivores only present where sinkholes connect caves to the surface and sunlight. Productivity is limited by the scarcity of light and food, but less so than in insular freshwater subterranean systems (SF1.1) due to influx of marine detritus and biota. The dominant fauna includes planktonic bacteria, protozoans, annelids, crustaceans, and fish. Anchialine obligates inhabit locations deep within the caves, with marine biota increasing in frequency with proximity to the sea. Caves closely connected with the ocean tend to have stronger tidal currents and biota such as sponges and hydroids commonly associated with sea caves (SM1.3). Distinctive traits of cave obligates that reflect selection under darkness and food scarcity include varying degrees of eye loss and depigmentation, increased tactile and chemical sensitivity, reproduction with few large eggs, long lifespans, and slow metabolism and growth rates. Some anchialine biota are related to deep sea species, including shrimps that retain red pigmentation, while others include relict taxa inhabiting anchialine caves on opposite sides of ocean basins. Characteristic anchialine taxa also occur in isolated water bodies, far within extensive seafloor cave systems.

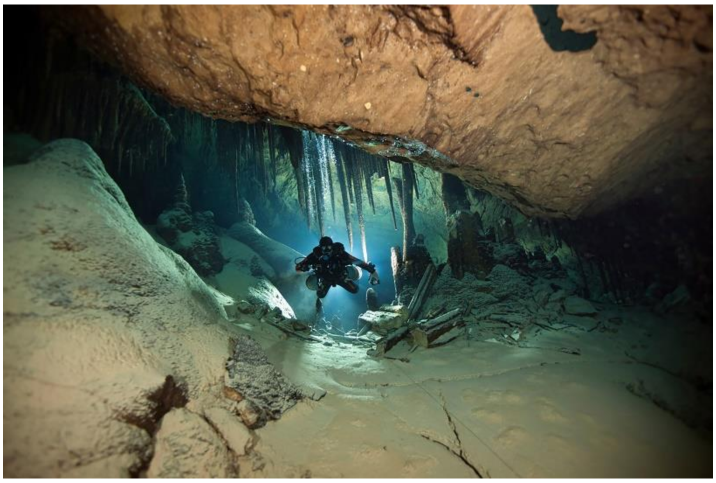

Tom Iliffe at Deep Blue Cave, Walsingham System, Bermuda.
Credit: Jill Heinerth / NOAA

Ecological drivers: Anchialine caves originate from seawater penetration into faults, fractures, and lava tubes as well as sea-level rise into limestone caves formed by solution. Cave waters are characterised by an absence or scarcity of light, low food abundance, and strong salinity gradients. Sharp haloclines, which fluctuate with tides and rainfall percolation, occur at deeper depths with increasing distance inland. Tidal connections result in suck and blow phases of water movement that diminish with increasing distance from the sea. In karst terrain with no surface runoff, anchialine caves are closely linked via hydrology to overlying subaerial coastal systems and can serve as subterranean rivers with haloclines separating seaward flowing freshwater from underlying saltwater. Temperatures are moderate, increasing at the halocline, then stabilise with depth. Dissolved oxygen declines with depth.

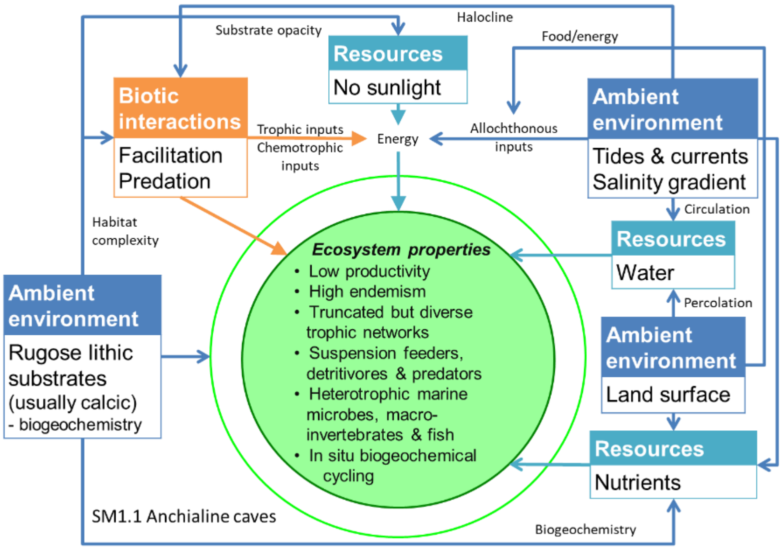

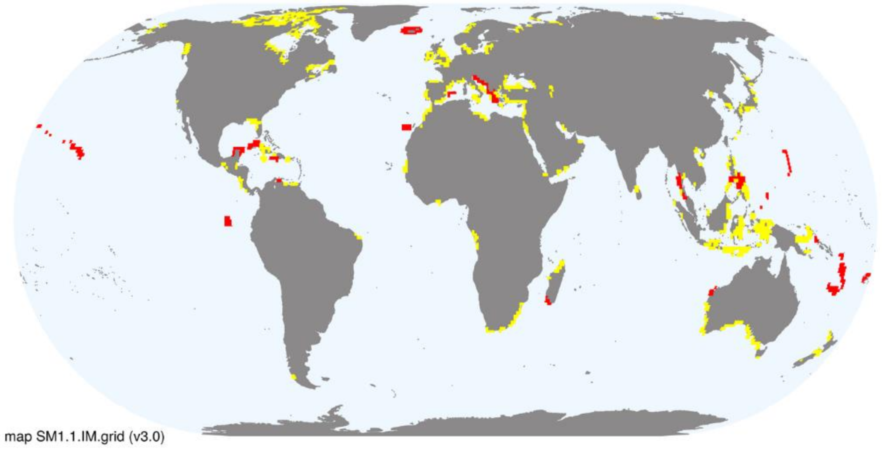

Distribution: Scattered worldwide, mostly in the Northern Hemisphere in limestone, basalt flows, and more rarely other lithic substrates.

References:

Iliffe TM (2000) Anchialine cave ecology. *Ecosystems of the World. Vol. 30. Subterranean Ecosystems* (Eds. H Wilkens, DC Culver, WF Humphreys), pp 59-76. Elsevier, Amsterdam.

Map is for illustrative purposes only and does not support spatial analyses unless formally validated.

SM1.2 Anchialine pools

Ecosystem properties: Anchialine pools, like anchialine caves (SM1.1), are tidally influenced bodies of brackish water with subterranean connections to the sea and groundwater, but with significant or full exposure to open air and sunlight. They have no surface connection to the ocean or freshwater ecosystems. Younger anchialine pools are exposed to abundant sunlight, characterized by relatively low productivity, and tend to support only benthic microalgae, cyanobacteria, and primary consumers. Older pools with more established biological communities have higher productivity with a wider range of autotrophs, including macroalgae, aquatic monocots, established riparian and canopy vegetation, and primary and secondary consumers. High productivity is attributed to a combination of sunlight exposure, rugose substrates, and relatively high natural concentrations of inorganic nutrients from groundwater. Anchialine pools may support complex benthic microbial communities, primary consumers, filter-feeders, detritivores, scavengers and secondary consumers. These consumers are primarily molluscs and crustaceans, several of which are anchialine obligates. Due to connections with deeper hypogeal habitats, obligate species may display physical and physiological traits similar to anchialine cave species. However, larger predatory fish and birds also utilize anchialine pools for food and habitat. Anchialine pools are ecologically dynamic systems due to their openness, connections with surrounding terrestrial habitats and subterranean hydrologic connections. Consequently, they are inherently sensitive to ecological phase shifts throughout their relatively ephemeral existence, with senescence initiating in as little as 100 years. However, new anchialine pools may form within a few months after basaltic lava flows.

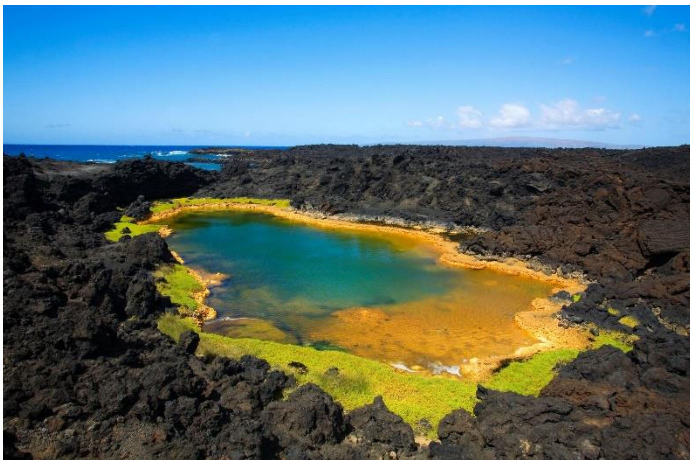

Anchialine Pond; Makena, Ahihi Kinau Natural Reserve, Maui, Hawaii.
Credit: Design Pics Inc / Alamy Stock

Ecological drivers: Anchialine pools form from subterranean mixing of seawater and groundwater, primarily through porous basalt or limestone substrates, and more rarely other lithic substrates. Tidal influences can drive large fluctuations in water level and salinity on a daily cycle, but are typically dampened with increased distance from the ocean. Sunlight, UV exposure and other environmental characteristics vary within anchialine pools and haloclines are common. The pools can also be connected to anchialine cave systems (SM1.1) through tension fissures in basalt flows, and collapsed openings in lava tubes.

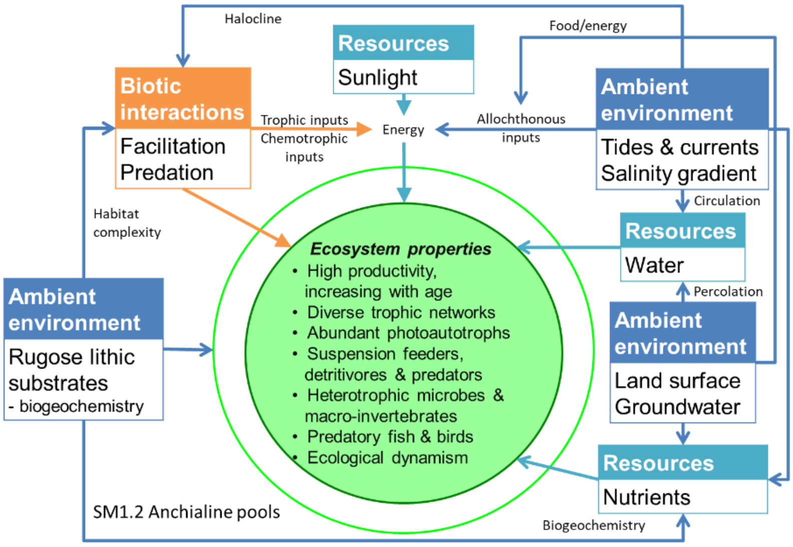

Distribution: Scattered worldwide, mostly in the northern hemisphere. Many well- known examples occur in Hawaii, Palau and Indonesia, volcanic cracks or grietas in the Galapagos Islands, and open-air entrance pools of anchialine caves (e.g. cenotes in Mexico's Yucatan Peninsula and blue holes in the Bahamas).

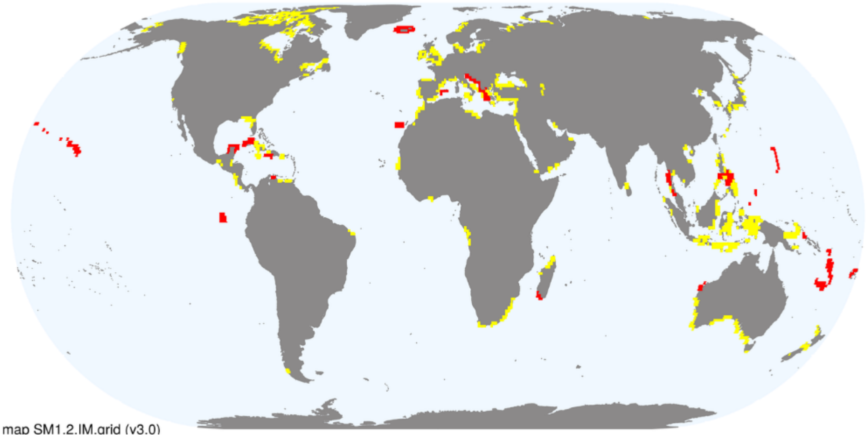

References:

Becking LE, Renema W, Santodomingo NK, Hoeksema BW, Tuti Y, de Voogd NJ (2011) Recently discovered landlocked basins in Indonesia reveal high habitat diversity in anchialine systems. *Hydrobiologia* 677, 89-105.

Por FD (1985) Anchialine pools—comparative hydrobiology. *Hypersaline ecosystems* pp. 136-144. Springer, Berlin, Heidelberg.

Map is for illustrative purposes only and does not support spatial analyses unless formally validated.

Contributors: V Gerovasileiou, TM Iliffe, V Gerovasileiou, B Gonzalez, D Brankovits, A Martínez García, DA Keith

SM1.3 Sea caves

Ecosystem properties: Sea caves (also known as marine or littoral caves) are usually formed by wave action abrasion in various rock types. In contrast to anchialine caves (SM1.1), sea caves are not isolated from the external marine environment. Thus, the biota in sea caves are mostly stygophiles (typical of dim-light cryptic and deep-water environments outside caves) or stygoxenes (species sheltering in caves during daytime but foraging outside at night). However, numerous taxa (mostly sessile invertebrates) have so far been reported only from sea caves, and thus can be considered as cave-exclusive sensu lato. Visitors often enter sea caves by chance (e.g. carried in by currents), and survive only for short periods. The diverse sea-cave biota is dominated by sessile (e.g. sponges, cnidarians, bryozoans) and motile invertebrates (e.g. molluscs crustaceans, annelids,) and fish. Photoautotrophs are restricted close to cave openings, while chemoautotrophic bacteria form extensive mats in sea caves with hydrothermal sulphur springs, similar to those in some terrestrial caves (SF1.1) and deep sea vents (M3.7). In semi-dark and dark cave sectors, the main trophic categories are filter-feeders (passive and active), detritivores, carnivores, and omnivores. Decomposers also play important roles. Filter-feeders consume plankton and suspended organic material delivered by tidal currents and waves. Other organisms either feed on the organic material produced by filter-feeders or move outside caves in order to find food. These “migrants”, especially swarm-forming crustaceans and schooling fish, can be significant import pathways for organic matter, mitigating oligotrophy in confined cave sectors.

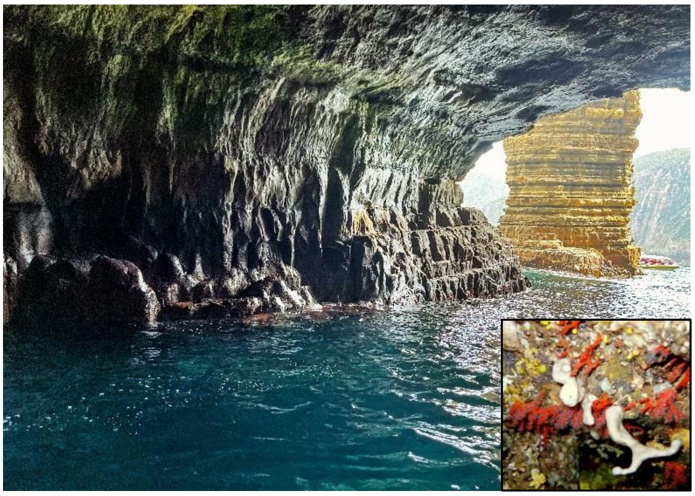

Sea cave at Cape Pillar, Tasmania, Australia. Inset: Red coral on submerged semi-dark cave wall, Mediterranean Sea
Credit: Andrew Merry / Getty Images. Inset: Vasilis Gerovasileiou

Ecological drivers: Sea caves openings vary from fully submerged and never exposed to the atmosphere to partially submerged and exposed to waves and tides. Sea caves are generally shorter and receive less input of freshwater from terrestrial sources than anchialine caves (SM1.1). Sea caves thus lack haloclines, a defining feature of anchialine caves, and are influenced more strongly by marine waters and biota throughout their extent. While salinity gradients are weak, the decrease of light and sea water renewal from the opening to the cave interior drive marked zonation of biota by creating oligotrophic conditions and limiting larval supply. Submersion level, cave morphology and micro-topography play key roles in forming such gradients.

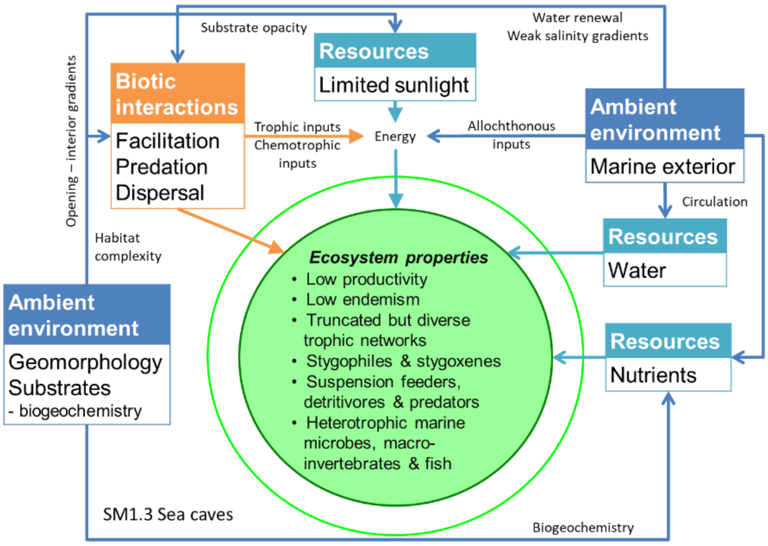

Distribution: Globally distributed in coastal headlands, rocky reefs and in coral reefs.

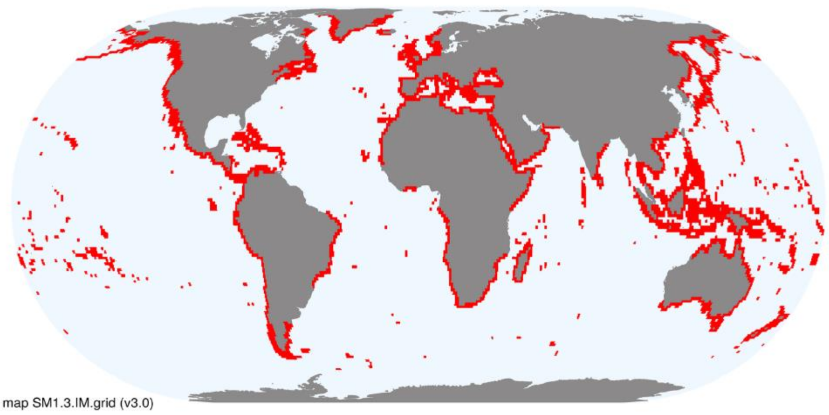

References:

Cicogna F, Bianchi CN, Ferrari G, Forti P (2003) *Le grotte marine: cinquant’anni di ricerca in Italia* Ministero dell’Ambiente e della Tutela del Territorio: Roma.

Gerovasileiou, V., Bianchi, C.N. (2021) ‘Mediterranean marine caves: a synthesis of current knowledge’. Oceanography & Marine Biology: Annual Review 59, 1-88.

Gerovasileiou V, Martínez A, Álvarez F, Boxshall G, Humphreys W, Jaume D, Becking L, Muricy G, van Hengstum P, Dekeyzer S, Decock W, Vanhoorne B, Vandepitte L, Bailly N, Iliffe T (2016) World Register of marine Cave Species (WoRCS): a new thematic species database for marine and anchialine cave biodiversity. *Research Ideas and Outcomes* 2, e10451.

Riedl R (1966) *Biologie der Meereshöhlen* Paul Parey: Hamburg.

Map is for illustrative purposes only and does not support spatial analyses unless formally validated.

TF1. Palustrine wetlands biome

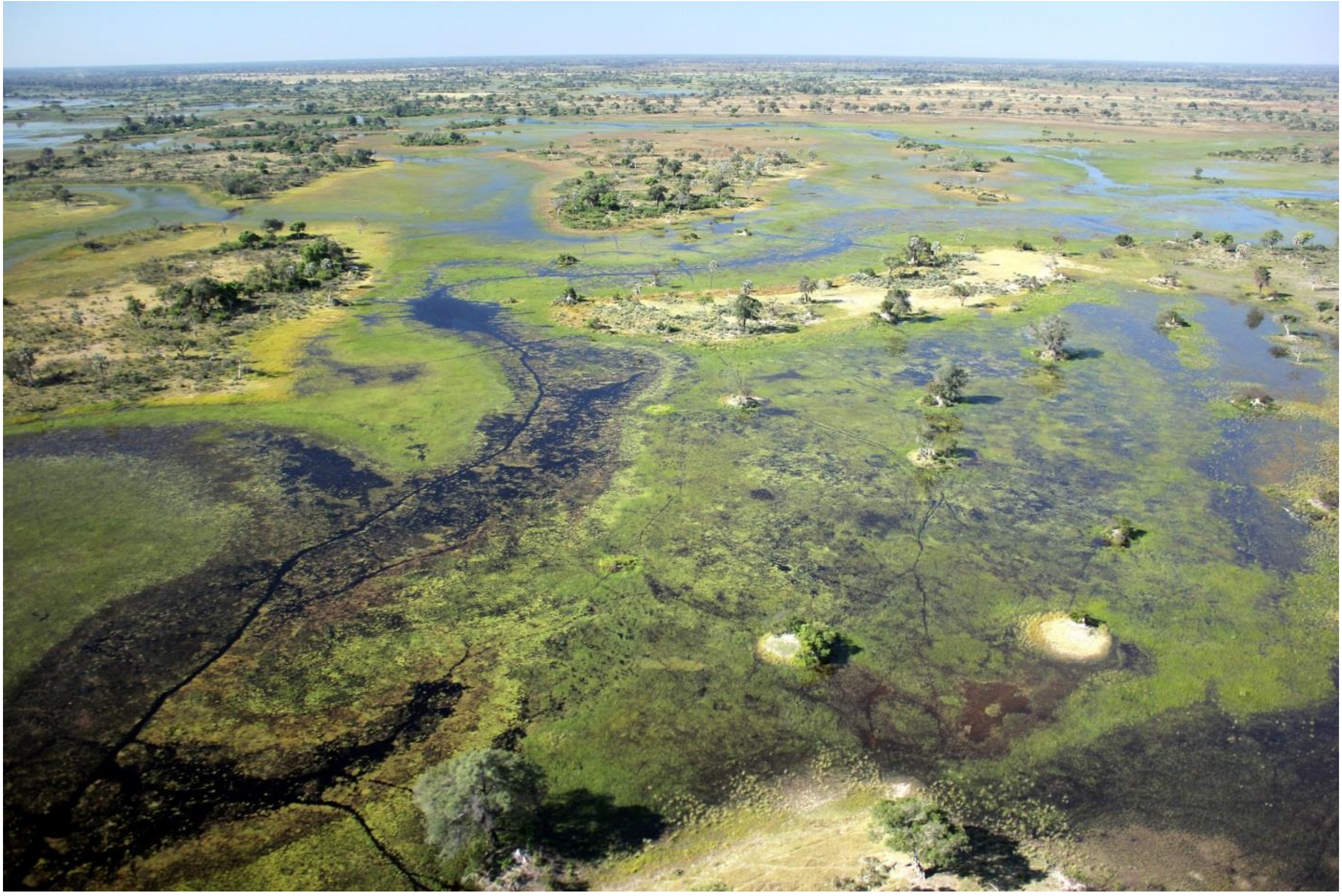

Okavango wetlands, Botswana.

Credit: Richard Kingsford

At the interface of terrestrial and freshwater realms, the Palustrine wetlands biome includes vegetated floodplains, groundwater seeps, and mires with permanent or intermittent surface water. Although water and light are abundant at least periodically, saturation of the soil may result in oxygen deprivation below the ground. This suppresses microbial activity and, in many systems, production exceeds decomposition, resulting in peat accumulation.

The water regime influences resource availability and productivity and thus regulates these ecosystems from the bottom-up. Interactions among catchment precipitation, local evapotranspiration, and substrate and surface morphology regulate run-on, run-off, infiltration, and percolation. This results in water regimes that vary from permanent shallow standing water or near-surface water tables to seasonally high water tables to episodic inundation with long inter-annual dry phases.

As a consequence of their indirect relationships with climate, wetland biomes are traditionally classified as 'azonal'. Spatial heterogeneity is a key feature of palustrine wetlands. At landscape scales, they function as resource sinks and refuges with substantially higher productivity than the surrounding matrix. Fine-scale spatial variation in the water regime often produces restricted hydrological niches and intricate mosaics of patch types with contrasting structure and biotic composition.

Autotrophs dominate complex trophic webs. Amphibious macrophytes are the dominant autotrophs, although epibenthic algae are important in some systems. Amphibious plants have specialised traits enabling growth and survival in low-oxygen substrates and often engineer habitats for heterotrophs. Microbial decomposers and invertebrate detritivores are most abundant in surface soils. A range of microscopic and macroinvertebrates with sedentary adult phases (e.g. crustaceans) have obligate associations with Palustrine wetlands, which also provide important foraging and breeding sites for macroinvertebrate and vertebrate herbivores and predators that disperse more widely across the landscape, including waterbirds.

TF1.1 Tropical flooded forests and peat forests

Ecosystem properties: Closed-canopy forests in tropical swamps and riparian zones have high biomass and LAI, with unseasonal growth and reproductive phenology. The canopy foliage is evergreen, varying in size from mesophyll to notophyll with moderate SLA. Productivity differs markedly between high-nutrient 'white water' riparian systems and low-nutrient 'black water' systems. In the latter, most of the nutrient capital is sequestered in plant biomass, litter, or peat, whereas in white water systems, soil nutrients are replenished continually by fluvial subsidies. Some trees have specialised traits conferring tolerance to low-oxygen substrates, such as surface root mats, pneumatophores, and stilt roots. Palms (sometimes in pure stands), hydrophytes, pitcher plants, epiphytic mosses, and ferns may be abundant, but lianas and grasses are rare or absent. The recent origin of these forests has allowed limited time for evolutionary divergence from nearby lowland rainforests (T1.1), but strong filtering by saturated soils has resulted in low diversity and some endemism. The biota is spatially structured by local hydrological gradients. Riparian galleries of floodplain forests also occur within savanna matrices. Trophic networks are complex but with less diverse representation of vertebrate consumers and predators than T1.1, although avian frugivores, primates, amphibians, macro-invertebrates, and crocodilian predators are prominent. Plant propagules are dispersed mostly by surface water or vertebrates. Seed dormancy and seedbanks are rare. Gap-phase dynamics are driven by individual treefall, storm events, or floods in riparian forests, but many plants exhibit leaf-form plasticity and can recruit in the shade.

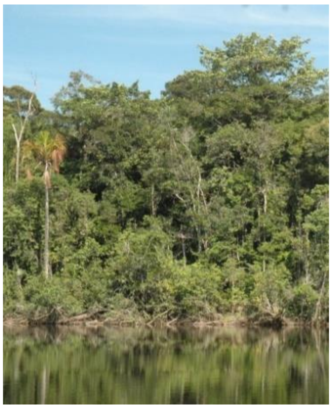
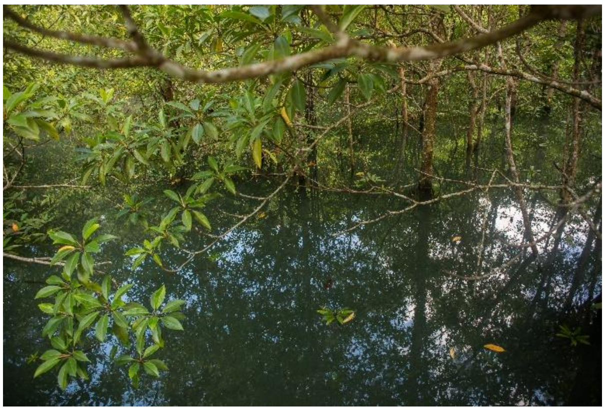

Left: White water riparian forest, Rio Carrao, Venezuela. Credit: David Keith (2012)
Right: Black water peat swamp forest, Tha Pom Klong Song Nam Krabi, Thailand
Credit: Sirachai Arunrugstichai / Getty Images

Ecological drivers: High rainfall, overbank flows or high water tables maintain an abundant water supply. Continual soil profile saturation leads to anaerobic black water conditions and peat accumulation. In contrast, white water riparian zones undergo frequent fluvial disturbance and drain rapidly. Peat forests often develop behind lake shore vegetation or mangroves, which block lateral drainage. Black water peatlands may become domed, ombrogenous (i.e. rain-dependent), highly acidic, and nutrient-poor, with peat accumulating to depths of 20 m. In contrast, white water riparian forests are less permanently inundated and floods continually replenish nutrients, disturb vegetation, and rework sediments. Hummock-hollow micro-topography is characteristic of all forested wetlands and contributes to niche diversity. Light may be limited by dense tree canopies. There is low diurnal, intra- and inter-annual variability in rainfall and temperature, with the latter rarely <10°C, which promotes microbial activity when oxygen is available.

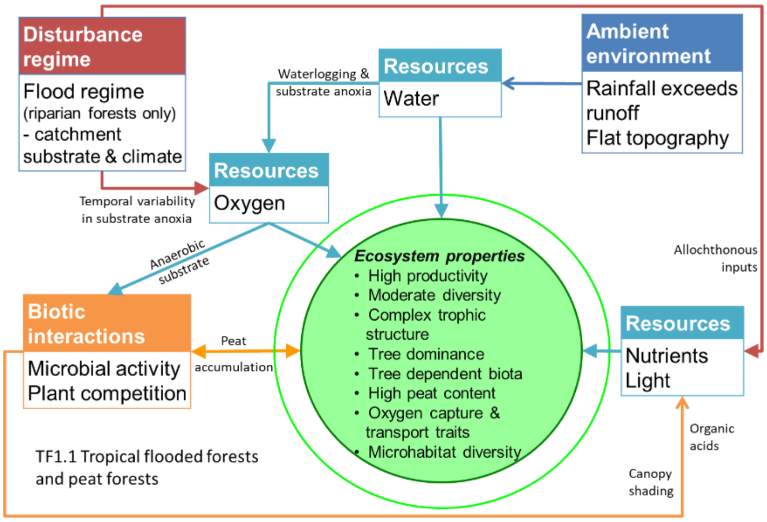

Distribution: Flat equatorial lowlands of Southeast Asia, South America, and Central and West Africa, notably in Borneo and the Amazonian lowlands.

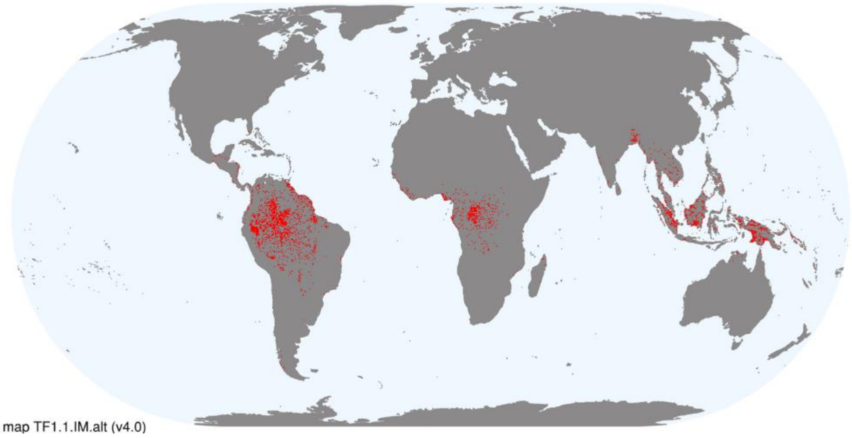
map TF1.1.IM.alt (v4.0)

References:

Page SE, Rieley JO, Wüst R (2014) Lowland tropical peatlands of Southeast Asia. *Peatlands: Evolution and records of environmental and climate changes* (Eds. IP Martini, Martínez A Cortizas, W Chesworth), pp145-172. Elsevier, Amsterdam.

Map is for illustrative purposes only and does not support spatial analyses unless formally validated.

TF1.2 Subtropical/temperate forested wetlands

Ecosystem properties: These hydrophilic forests and thickets have an open to closed tree or shrub canopy, 2–40 m tall, dependent on flood regimes or groundwater lenses. Unlike tropical forests (TF1.1), they typically are dominated by one or very few woody species. Trees engineer fine-scale spatial heterogeneity in resource availability (water, nutrients, and light) and ecosystem structure, which affects the composition, form, and functional traits of understorey plants and fauna. Engineering processes include the alteration of sediments, (e.g. surface micro-topography by the growth of large roots), the deposition of leaf litter and woody debris, canopy shading, creation of desiccation refuges for fauna and the development of foraging or nesting substrates (e.g. tree hollows). Forest understories vary from diverse herbaceous assemblages to simple aquatic macrophyte communities in response to spatial and temporal hydrological gradients, which influence the density and relative abundance of algae, hydrophytes and dryland plants. Primary production varies seasonally and inter-annually and can be periodically high due to the mobilisation of nutrients on floodplains during inundation. Nutrients accumulate on floodplains during low flows, and may drive microbial blooms, leading to aquatic anoxia, and fish kills, which may be extensive when flushing occurs. Plant and animal life histories are closely connected to inundation (e.g. seed-fall, germination fish-spawning and bird breeding are stimulated by flooding). Inundation-phase aquatic food webs are moderately complex. Turtles, frogs, birds and sometimes fish exploit the alternation between aquatic and terrestrial phases. Waterbirds forage extensively on secondary production, stranded as floodplains recede, and breed in the canopies of trees or mid-storey. Forested wetlands are refuges for many vertebrates during droughts. Itinerant mammalian herbivores (e.g. deer and kangaroos) may have locally important impacts on vegetation structure and recruitment.

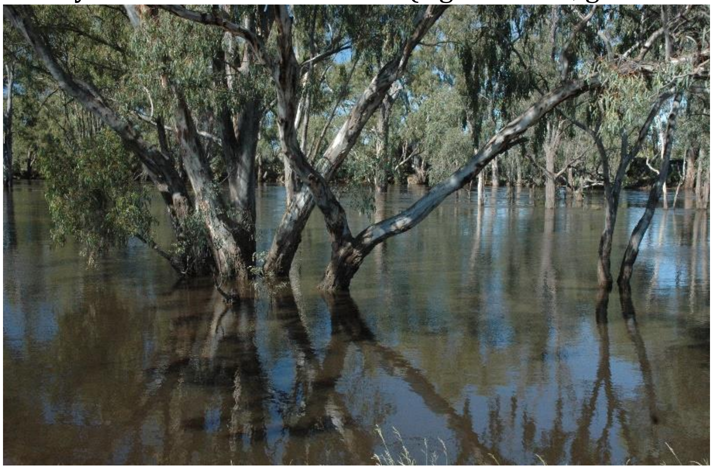

Flooded River Red Gum forest, Echuca, Australia.
Credit: David Keith

Ecological drivers: These forests occur on floodplains, riparian corridors, and disconnected lowland flats. Seasonally and inter-annually variable water supply influences ecosystem dynamics. Allochthonous water and nutrient subsidies from upstream catchments supplement local resources and promote the extension of floodplain forests and their biota into arid regions ('green tongues'). Water movement is critical for the connectivity and movement of biota, while some groundwater-dependent forests are disconnected. High-energy floods in riparian corridors displace standing vegetation and woody debris, redistribute nutrients, and create opportunities for dispersal and recruitment. Low-energy environments with slow drainage promote peat accumulation. Extreme drying and heat events may generate episodes of tree dieback and mortality. Fires may occur depending on the frequency of fire weather, ignition sources, and landscape context.

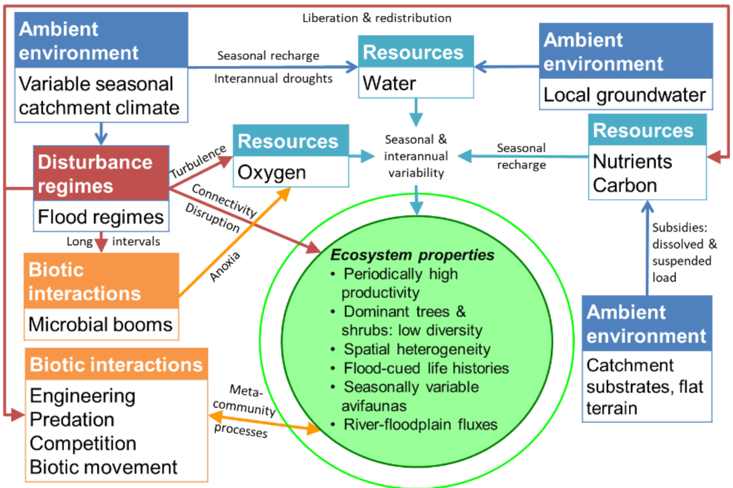

TF1.2 Subtropical/temperate forested wetlands

Distribution: Temperate and subtropical floodplains, riparian zones and lowland flats worldwide.

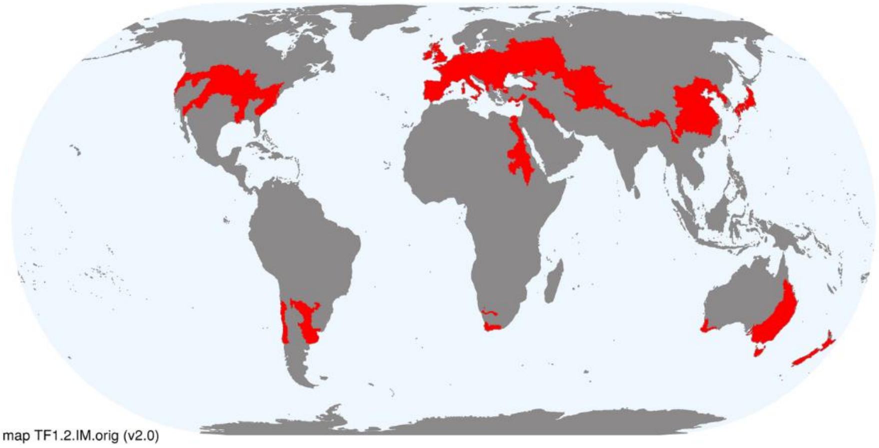

map TF1.2.IM.orig (v2.0)

References:

Mac Nally R, Cunningham SC, Baker PJ, Horner GJ, Thomson JR (2011) Dynamics of Murray-Darling floodplain forests under multiple stressors: The past, present, and future of an Australian icon. *Water Resources Research* 47, W00g05.

Map is for illustrative purposes only and does not support spatial analyses unless formally validated.

Contributors: RT Kingsford, JA Catford, MC Rains, B Robson, DA Keith

TF1.3 Permanent marshes

Ecosystem properties: These shallow, permanently inundated freshwater wetlands lack woody vegetation but are dominated instead by emergent macrophytes growing in extensive, often monospecific groves of rhizomatous grasses, sedges, rushes, or reeds in mosaics with patches of open water. These plants, together with phytoplankton, algal mats, epiphytes, floating, and amphibious herbs, sustain high primary productivity and strong bottom-up regulation. Although most of the energy comes from these functionally diverse autotrophs, inflow and seepage from catchments may contribute allochthonous energy and nutrients. Plant traits including aerenchymatous stems and leaf tissues (i.e. with air spaces) enable oxygen transport to roots and rhizomes and into the substrate. Invertebrate and microbial detritivores and decomposers inhabit the water column and substrate. Air-breathing invertebrates are more common than gill-breathers, due to low dissolved oxygen. The activity of microbial decomposers is also limited by low oxygen levels and organic deposition continually exceeds decomposition. Their aquatic predators include invertebrates, turtles, snakes and sometimes small fish. The emergent vegetation supports a complex trophic web including insects with winged adult phases, waterbirds, reptiles, and mammals, which feed in the vegetation and also use it for nesting (e.g. herons, muskrat, and alligators). Waterbirds include herbivores, detritivores, and predators. Many plants and animals disperse widely beyond the marsh through the air, water and zoochory (e.g. birds, mammals). Reproduction and recruitment coincide with resource availability and may be cued to floods. Most macrophytes spread vegetatively with long rhizomes but also produce an abundance of wind- and water-dispersed seeds.

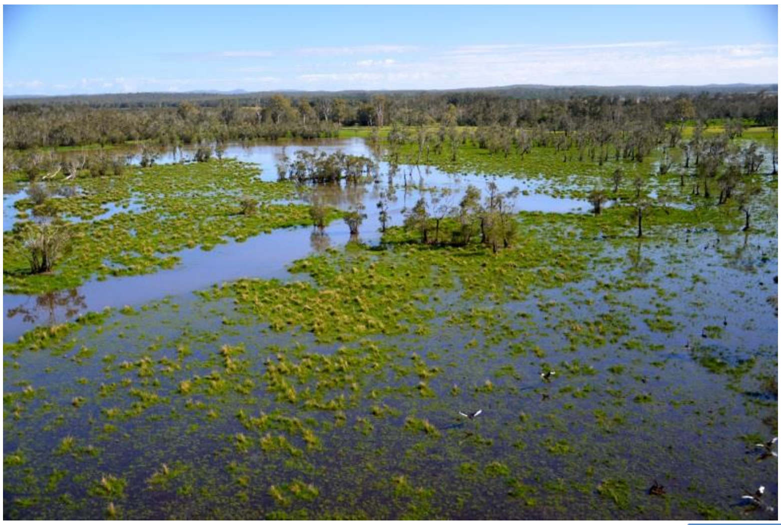

Everlasting Swamp, NSW, Australia.
Credit: John Spencer / OEH

Ecological drivers: These systems occur in several geomorphic settings including lake shores, groundwater seeps, river floodplains, and deltas, always in low-energy depositional environments. Shallow but perennial inundation and low variability are maintained by frequent floods and lake waters, sometimes independently of local climate. This sustains high levels of water and nutrients but also generates substrate anoxia. Substrates are typically organic. Their texture varies, but silt and clay substrates are associated with high levels of P and N. Salinity is low but may be transitional where wetlands connect with brackish lagoons (FM1.2, FM1.3). Surface fires may burn vegetation in some permanent marshes, but rarely burn the saturated substrate, and are less pervasive drivers of these ecosystems than seasonal floodplain marshes (TF1.4).

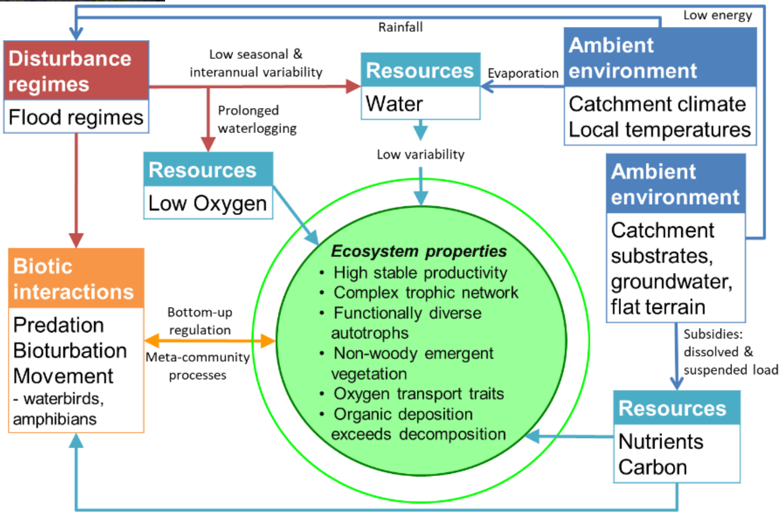

TF1.3 Permanent marshes

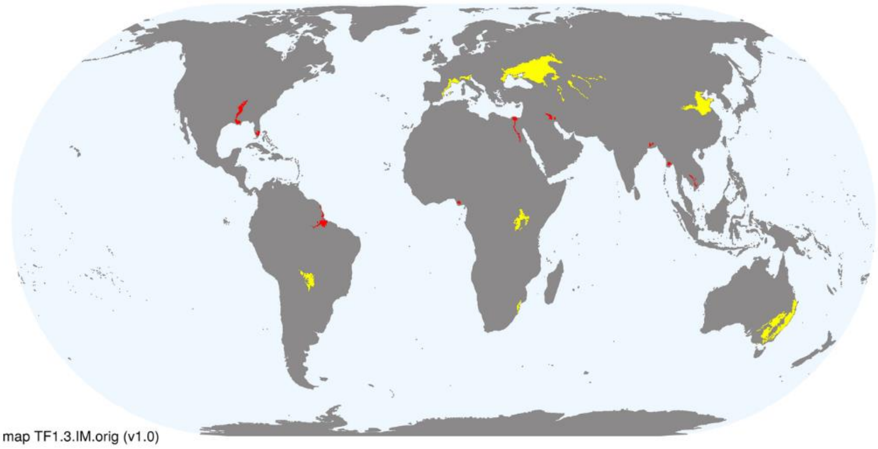
map TF1.3.IM.orig (v1.0)

Distribution: Scattered throughout the tropical and temperate regions worldwide.

References:

Grace JB, Wetzel RG (1981) Habitat partitioning and competitive displacement in cattails (Typha): experimental field studies. *The American Naturalist* 118:463-474.

Map is for illustrative purposes only and does not support spatial analyses unless formally validated.

Contributors: DA Keith, RT Kingsford, R Mac Nally, B Robson, JA Catford, MC Rains, K Irvine, KM Rodriguez-Clark, A Etter

TF1.4 Seasonal floodplain marshes

Ecosystem properties: This group includes high-productivity floodplain wetlands fed regularly by large inputs of allochthonous resources that drive strong bottom-up regulation, and smaller areas of disconnected oligotrophic wetlands. Functionally diverse autotrophs include phytoplankton, algal mats and epiphytes, floating and amphibious herbs and graminoids, and semi-terrestrial woody plants. Interactions of fine-scale spatial gradients in anoxia and desiccation are related to differential flooding. These gradients shape ecosystem assembly by enabling species with diverse life-history traits to exploit different niches, resulting in strong local zonation of vegetation and high patch-level diversity of habitats for consumers. Wetland mosaics include very productive and often extensive grasses, sedges and forbs (sedges dominate oligotrophic systems) that persist through dry seasons largely as dormant seeds or subterranean organs, as well as groves of woody perennials that are less tolerant of prolonged anoxia but access ground water or arrest growth during dry phases. Productive and functionally diverse autotrophs support complex trophic networks with zooplankton, aquatic invertebrates, fish, amphibians, reptiles, aquatic mammals, waterbirds, and terrestrial animals with diverse dietary and foraging strategies. During dry phases, obligate aquatic organisms are confined to wet refugia. Others, including many invertebrates, have dormancy traits allowing persistence during dry phases. Very high abundances and diversities of invertebrates, waterbirds, reptiles, and mammals exploit resource availability, particularly when prey are concentrated during drawdown phases of floods. Reproduction and recruitment, especially of fish, coincide with food availability cued by flood regimes.

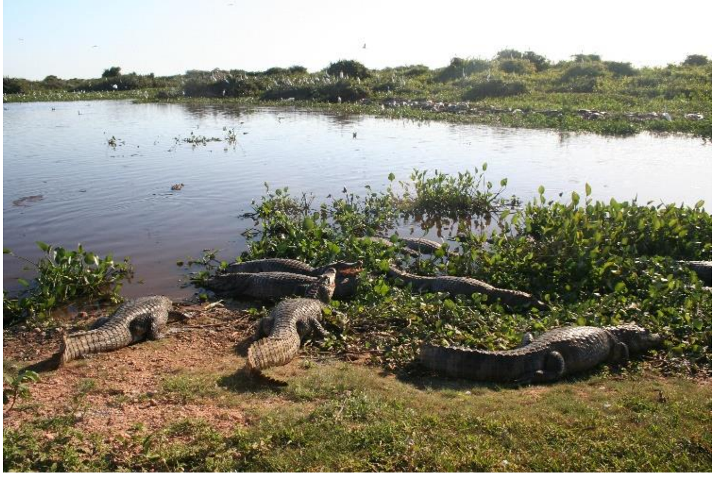

The Pantanal, Brazil.
Credit: Richard Kingsford

Ecological drivers: Regular seasonal flooding and drying is driven by river flow regimes, reflecting seasonal precipitation or melt patterns in catchments. Salinity gradients and tides influence these marshes where they adjoin estuaries, with brackish marshes on transitions to TF1.2, TF1.3 and MFT1.3. Disconnected oligotrophic systems rely on rainfall and low substrate permeability for seasonal waterlogging. Seasonal flood extent and duration vary inter-annually, especially in temperate zones. Geomorphic heterogeneity in the depositional floodplains promote spatial and temporal variability in moisture status, creating contrasting patches including perennially inundated refuges and dry 'islands' that seldom flood and dry rapidly. Substrates are fertile alluvia or infertile white sands with variable grain sizes, moisture, and organic content that reflect fine-scale depositional patterns and hydrological gradients. Fires may occur in dry seasons, releasing resources, changing vegetation structure and composition, consuming organic substrates and lowering the wetland surface.

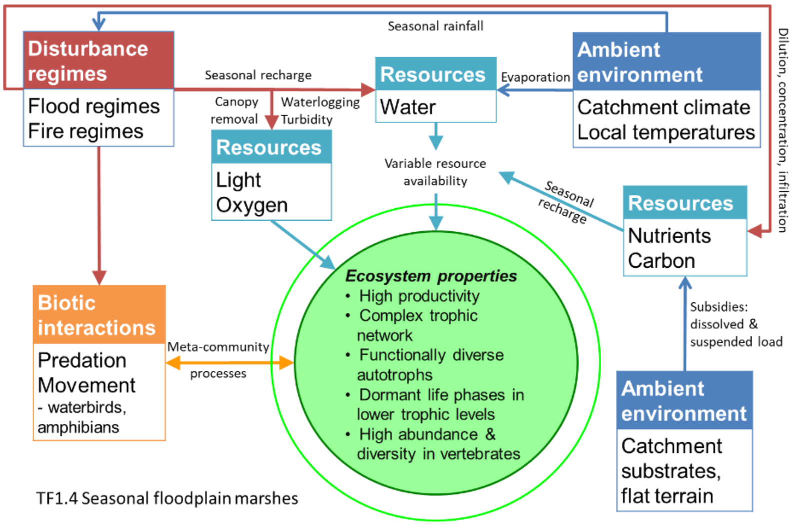

TF1.4 Seasonal floodplain marshes

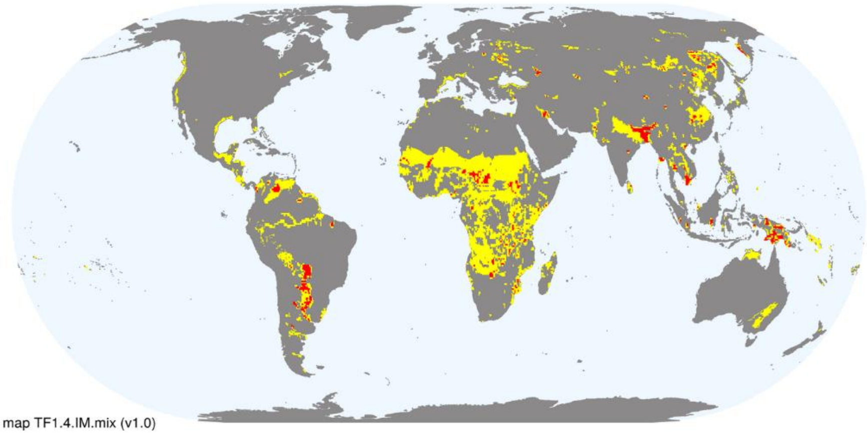
map TF1.4.IM.mix (v1.0)

Distribution: Throughout the seasonal tropics and subhumid temperate regions of the world.

References:

Damasceno-Junior GA., Semir J, Dos Santos FAM, de Freitas Leitão-Filho H (2005) Structure, distribution of species and inundation in a riparian forest of Rio Paraguai, Pantanal, Brazil. *Flora-Morphology, Distribution, Functional Ecology of Plants* 200:119-135.

Contributors: RT Kingsford, R Mac Nally, AH Arthington, JA Catford, B Robson, DA Keith

TF1.5 Episodic arid floodplains

Ecosystem properties: Highly episodic freshwater floodplains are distinct from, but associated with, adjacent river channels, which provide water and sediment during flooding. These are low-productivity systems during long, dry periods (maybe years), with periodic spikes of very high productivity when first inundated. These floodplains have a high diversity of aquatic and terrestrial biota in complex trophic networks, with ruderal life-history traits enabling the exploitation of transient water and nutrient availability. Primary producers include flood-dependent macrophytes and algae with physiological traits for water conservation or drought avoidance. Lower trophic levels (e.g. algae, invertebrate consumers) avoid desiccation with traits such as dormant life-cycle phases, deposition of resting eggs (e.g. crustaceans and rotifers), and burial in sediments banks (e.g. larvae of cyclopoid copepods). Higher trophic levels (e.g. fish, amphibians, reptiles, and waterbirds) are highly mobile in large numbers or with resting strategies (e.g. burrowing frogs). These taxa can be important mobile links for the movement of biota and resources, but floods are the primary allochthonous sources of energy and nutrients. Floods are important triggers for life-history processes such as seed germination, emergence from larval stages, dispersal, and reproduction. Common lifeforms include detritus-feeding invertebrate collector-gatherers, indicating a reliance on heterotrophic energy pathways.

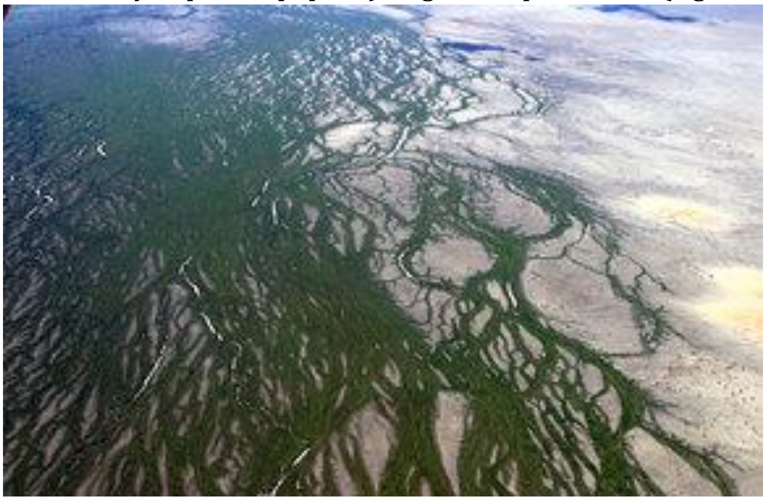

Episodic Eyre Creek arid floodplain, Queensland, Australia.
Credit: Richard Kingsford

Ecological drivers: Multi-year dry periods are punctuated by brief intervals of shallow inundation caused by the overspill from flooding river channels. These boom-bust systems have temporarily high productivity driven by water and partly by elevated levels of dissolved Carbon and nutrients (notably N and P) released from leaf litter, oxygen, and organic matter in newly inundated, shallow areas. High temperatures promote productivity and rapid drying in arid environments. Water may be turbid or clear, which affects light environments and may limit benthic algal production to the shallow littoral margins of small channels. This in turn affects aquatic food webs and Carbon dynamics. Drainage is predominantly horizontal and bidirectional (i.e. in and out of the river), but infiltration and evapotranspiration can be significant in the flat terrain and may influence salinity if there are sources of salt in the catchment or ground water.

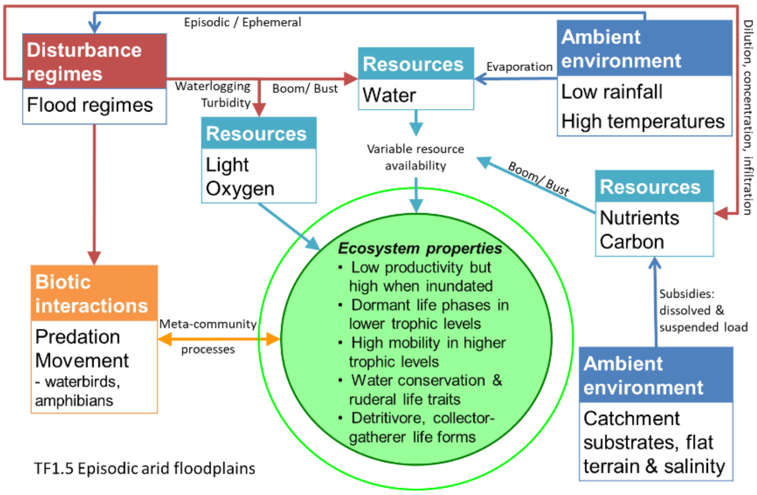

TF1.5 Episodic arid floodplains

Distribution: Connected to ephemeral rivers in semi-arid and arid regions of all continents.

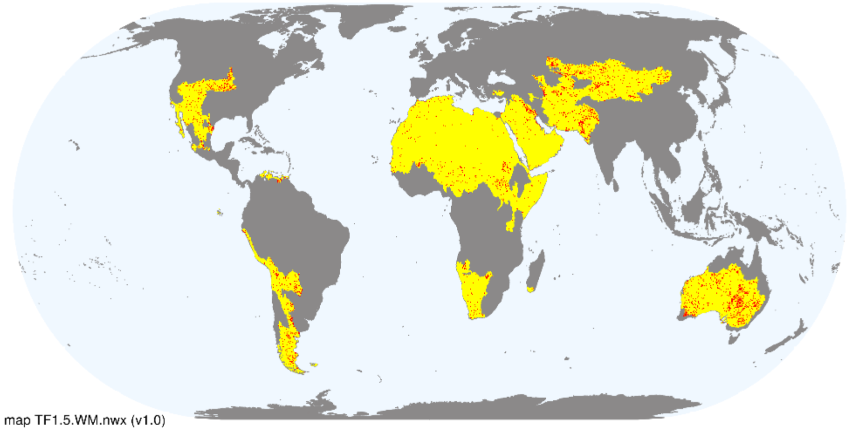

References:

Arthington AH, Balcombe S (2011) Extreme flow variability and the 'boom and bust' ecology of fish in arid-zone floodplain rivers: a case history with implications for environmental flows, conservation and management. *Ecohydrology* 4, 706-720.

Bunn SE, Balcombe SR, Davies PM, Fellows CS, McKenzie-Smith FJ (2006) Aquatic productivity and food webs of desert river ecosystems. *Ecology of desert rivers* (Ed. RT Kingsford), pp76-99. Cambridge University Press, Cambridge.

McInerney PJ, Rick J. Stoffels RJ, Shackleton ME, Davey CD (2017) Flooding drives a macroinvertebrate biomass boom in ephemeral floodplain wetlands. *Freshwater Science* 36: 726-738.

TF1.6 Boreal, temperate and montane peat bogs

Ecosystem properties: These patterned peatlands account for up to 40% of global soil carbon are dominated by a dense cover (high LAI) of hydrophytic mosses, graminoids, and shrubs, sometimes with scattered trees. Positive feedbacks between dense ground vegetation, hydrology, and substrate chemistry promote peat formation through water retention and inhibition of microbial decomposition. Moderate to low primary production is partially broken down at the soil surface by anamorphic fungi and aerobic bacteria. Burial by overgrowth and saturation by the water table promotes anaerobic conditions, limiting subsurface microbial activity, while acidity, nutrient scarcity, and low temperatures enhance the excess of organic deposition over decomposition. Plant diversity is low but fine-scale hydrological gradients structure vegetation mosaics, which may include fens (TF1.7). Mosses (notably Sphagnum spp.) and graminoids with layering growth forms promote peat formation. Their relative abundance influences microbial communities and peat biochemistry. Plant traits such as lacunate stem tissues, aerenchyma, and surface root mats promote oxygen transport into the anaerobic substrate. Woody plant foliage is small (leptophyll-microphyll) and sclerophyllous, reflecting excess carbohydrate production in low-nutrient conditions. Plants and fungi reproduce primarily by cloning, except where disturbances (e.g. fires) initiate gaps enabling recruitment. Pools within the bogs have specialised aquatic food webs underpinned by algal production and allochthonous carbon. Invertebrate larvae are prominent consumers in the trophic network of bog pools, and as adults they are important pollinators and predators. Assemblages of flies, dragonflies, damselflies, caddisflies and other invertebrates vary with the number, size and stability of pools. Carnivorous plants (e.g. sundews) support N cycling. Vertebrates are mostly itinerant but include specialised resident amphibians, reptiles, rodents, and birds. Some regions are rich in locally endemic flora and fauna, particularly in the Southern Hemisphere.

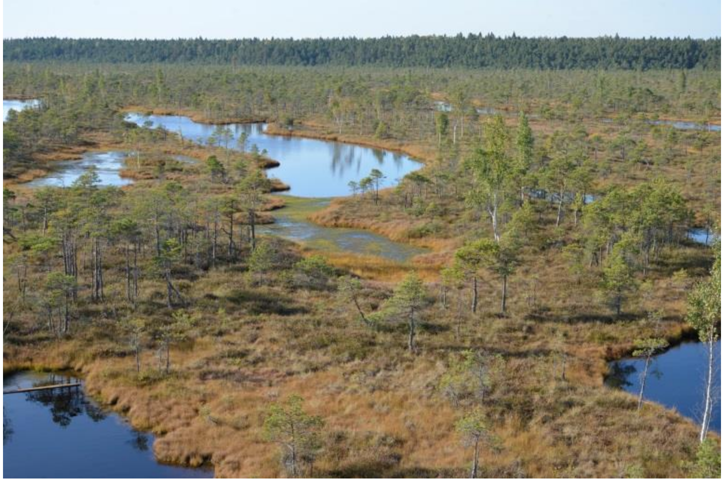

Raised peat bog with Sphagnum, scattered trees and flark pools, Kemeri Bog, Latvia.
Credit: David Keith

Ecological drivers: Bogs are restricted to cool humid climates where moisture inputs (e.g. precipitation, seepage, and surface inflow) exceed outputs (e.g. evapotranspiration, percolation, and run-off) for extended periods, enabling these systems to function as landscape sponges. Seasonally low temperatures and/or frequent cloud cover limit evapotranspiration. Substrates are waterlogged, anaerobic, highly organic (usually >30% dry weight), acidic (pH 3.5–6), and nutrient-poor. Peat growth may produce raised ombrotrophic bogs entirely fed by rain, but if minerotrophic inflows from catchments occur, they provide limited nutrient subsidies (cf. TF1.7). Fires may occur in dry summers, sometimes igniting peat with long-term consequences for ecosystem function and stability.

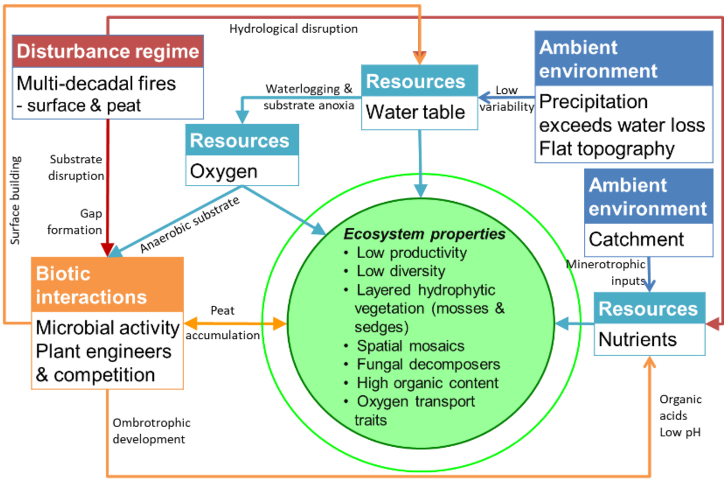

TF1.6 Boreal, temperate & montane peat bogs

Distribution: Extensive across boreal-subarctic latitudes, with small areas on tropical mountains of South America, New Guinea, and Central Africa and at cool, temperate southern latitudes in Patagonia and Australasia.

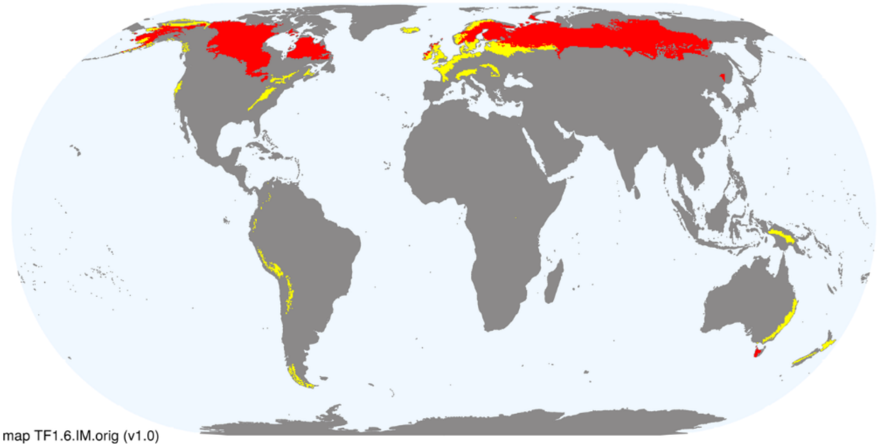

map TF1.6.IM.orig (v1.0)

References:

Palozzi JE, Lindo Z (2017) Boreal peat properties link to plant functional traits of ecosystem engineers. *Plant and Soil* 418: 277-291.

Wieder RK, Vitt DH (2006) Boreal peatland ecosystems. *Ecological studies* vol. 188. Springer-Verlag, Berlin.

Map is for illustrative purposes only and does not support spatial analyses unless formally validated.

TF1.7 Boreal and temperate fens

Ecosystem properties: Fens are peatland ecosystems dominated by hydrophytic grasses, sedges, or forbs. Fens have higher productivity but lower functional diversity than bogs (TF1.6). Productivity is subsidised by inflow of minerotrophic waters and limited by anoxic substrates. Plant diversity is very low where surface hydrology varies temporally from complete saturation to desiccation but can be high in mineral-rich fens with stable near-surface water tables. Some regions are rich in locally endemic flora and fauna. Woody plants are typically scarce or absent, though some boreal forests (T2.1) develop on minerotrophic peats. Sphagnum mosses and hummock-forming sedges are absent from rich fens but 'brown mosses' are common. Primary production is partly broken down on soil-surface layers by anamorphic fungi and aerobic bacteria. Anaerobic conditions due to high water tables limit subsurface microbial activity so that organic deposition exceeds decomposition and peat accumulates. Plant traits such as lacunate stem tissues, aerenchyma, and surface root mats promote oxygen transport into the anaerobic substrate. Methanogenic archaea and anaerobic bacteria may occur in the subsoil if N, Fe, and S are sufficient to sustain them. Fens may be spatially homogeneous or form string mosaics with bogs (e.g. aapa mires of Finland) but often display zonation reflecting differences in water chemistry (notably pH) or saturation. Patches of fen and bogs may be juxtaposed within peatland mosaics. Ongoing peat build-up may lead to transition from fen to bog systems. Plants and fungi reproduce locally by cloning, but seed and spore production enables dispersal and the colonisation of new sites. Invertebrates are dominant consumers in the trophic network, including dragonflies, caddisflies, flies, as well as calcareous specialists such as snails. Vertebrates are mostly itinerant but include specialised resident amphibians and birds.

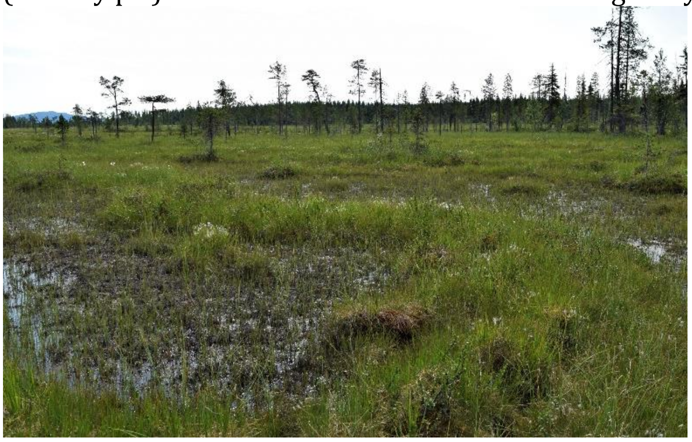

Calcareous quaking fen and sedge hummock strings within an aapa mire complex, Salla, Finland.
Credit: Teemu Tahvaneinan

Ecological drivers: Moisture inputs (precipitation, seepage, and surface inflow) exceed outputs (evapotranspiration, percolation, and run-off) for extended periods, enabling these systems to function as landscape sponges. Seasonally low temperatures and/or frequent cloud cover limit evapotranspiration. Fens typically develop by the paludification (i.e. peat accumulation) of shallow lakes or around springs and thus shallow standing water is present frequently. Such lakes may be abundant in post-glacial landscapes. Substrates are waterlogged, anaerobic, highly organic (usually >30% dry weight), slightly acidic or alkaline, and rich in mineral nutrients. Minerotrophic water (i.e. inflow from catchments) provides significant nutrient subsidies that vary with catchment geology. Fens on the arctic circle (palsa mires) have subsurface permafrost. Fires may occur in dry summers, rarely consuming peat, lowering the surface and degrading permafrost.

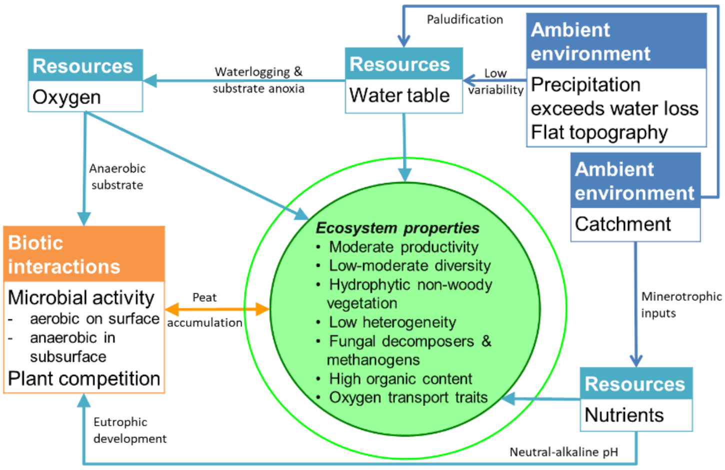

TF1.7 Boreal & temperate fens

Distribution: Extensive across boreal-subarctic latitudes and cool temperate regions, especially mountains. Very restricted in the Southern Hemisphere. Fens may also occur in tropical mountains (e.g. Andes), but are poorly known there.

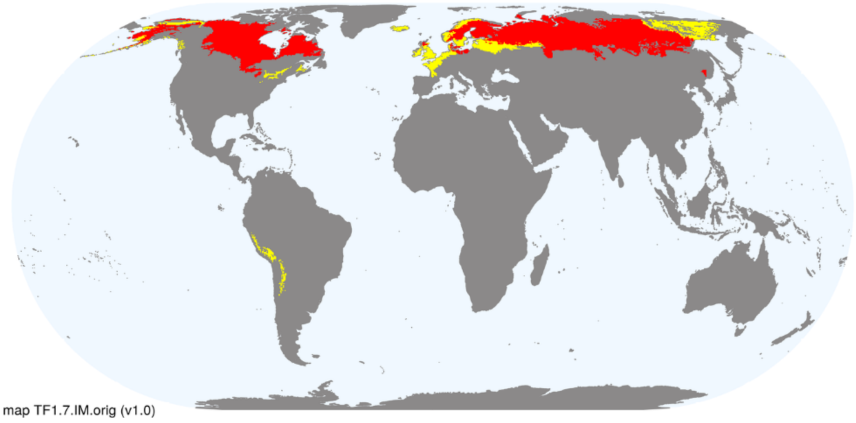

References:

Godwin KS., Shallenberger JP, Leopold DJ, Bedford BL (2002) Linking landscape properties to local hydrogeologic gradients and plant species occurrence in New York fens: a hydrogeologic setting (HGS) framework. *Wetlands* 22: 722–737.

Wieder RK, Vitt DH (2006) Boreal peatland ecosystems. *Ecological studies* vol. 188. Springer-Verlag, Berlin.

Map is for illustrative purposes only and does not support spatial analyses unless formally validated.

F1. Rivers and streams biome

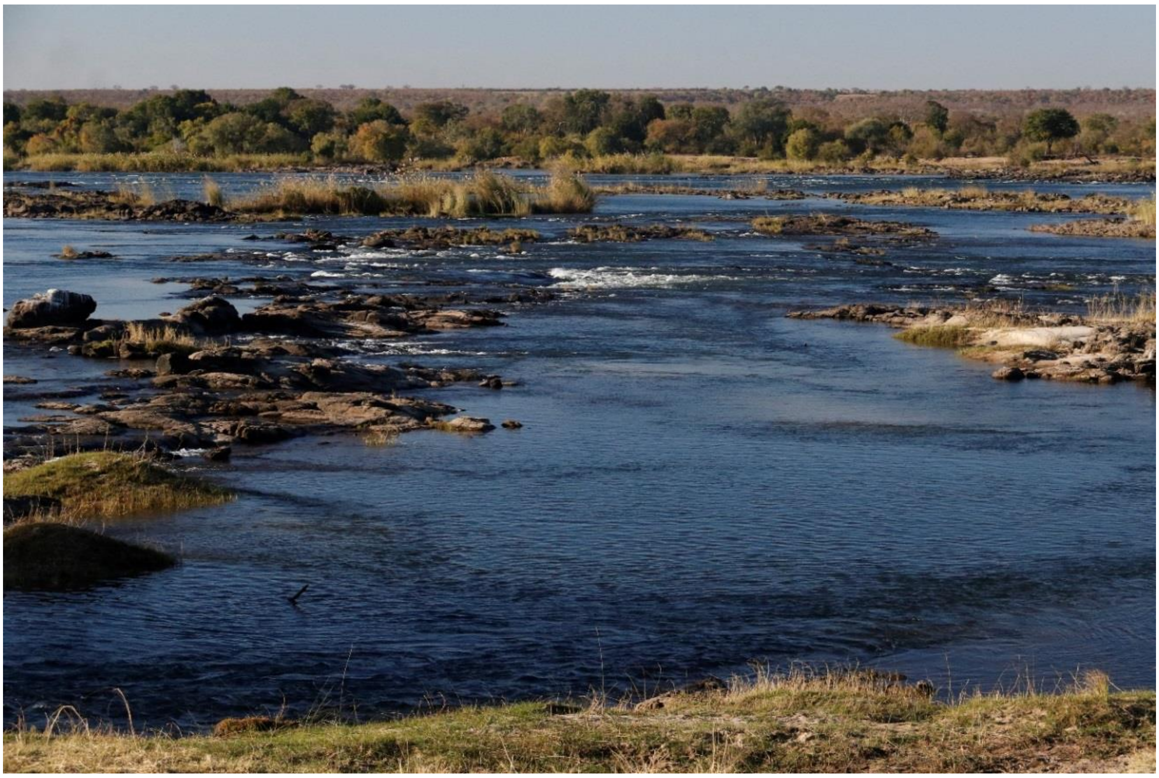

Zambezi River, Zimbabwe.

Credit: Richard Kingsford

Rivers and streams include lotic (running water) ecosystems, flowing from elevated uplands or underground springs to deltas, estuaries, and lakes. They are defined primarily by their linear structure, unidirectional flow regimes, and close interaction with the surrounding landscape. Individual rivers drain catchments separated by watersheds.

Channels that make up a river system can be classified into stream orders, with 1st order streams having no tributaries, 2nd order streams having 1st order tributaries, 3rd order having 2nd order tributaries and so on. The world's largest rivers are 10th-12th order. Flow regimes depend on stream order and rainfall patterns in the catchment (except in regulated rivers and spring-fed streams), which vary from year-round to seasonal to episodic. Stream gradients determine flow velocity and turbulence, bank and substrate structure, and habitat variability, but flow variability depends on regional climate and local weather. River systems in arid zones may remain dry for several years.

These factors act as selection filters, differentiating lotic ecosystems and their species' traits amongst flow regimes, and between uplands and lowlands. Productivity tends to increase from uplands to lowlands, and is driven both by allochthonous energy sources that contribute coarse organic matter from terrestrial ecosystems in adjacent riparian zones and upper catchments, and by autochthonous energy synthesis by biofilms or phytoplankton. Phytoplankton is important downstream in larger, slower rivers that carry smaller organic particles and more dissolved organic matter. Erosion and depositional processes depend on the gradient and position of a stream reach within a catchment, and are fundamental to the downstream passage of nutrients and organic matter, and their exchange between river ecosystems and surrounding land. Anthropogenic nutrient inputs increase downstream and vary with land use. Rivers with extensive peatlands in their catchments are rich in tannins, which reduce light penetration through the water column, increase acidity, promote microbial activity that thrives on dissolved organic carbon, and thereby reduce oxygen levels, productivity and biotic diversity, although endemism may be high. Streams in cold climates freeze over in winter, imposing seasonal constraints on productivity and the movement of organisms. Much of the biotic diversity resides in or on the stream benthos.

Trophic webs are more complex in large rivers due to greater resource availability and niche diversity, and species-catchment area relationships. Invertebrate detritivores consume fragments of organic matter, providing resources for predatory macroinvertebrates and fish, which in turn support larger predatory fish, waterbirds, reptiles, and some mammals. Specialised species-level traits are associated with different flow regimes and life history strategies often align with patterns of resource availability. For example, suspension feeding is common in high flow velocities, cold tolerance and seasonal dormancy occur in freeze-thaw streams, life cycles are geared to autumnal leaf fall in temperate forested catchments, and desiccation tolerance and dormant life stages dominate in episodic rivers.

F1.1 Permanent upland streams

Ecosystem properties: These 1st-3rd order streams generally have steep gradients, fast flows, coarse substrates, often with a riffle-pool (shallow and fast vs deeper and slow) sequence of habitats, and periodic (usually seasonal) high-flow events. Many organisms have specialised morphological and behavioural adaptations to high flow-velocity environments. Riparian trees produce copious leaf fall that provide allochthonous subsidies, and support somewhat separate foodwebs to those based on *in situ* primary production by bryophytes and biofilms. Tree shade conversely light-limits productivity, a trade-off that relaxes seasonally where deciduous trees dominate. Microbes and detritivores (e.g. invertebrate shredders) break down leaf fall and other organic matter. Microbial biofilms comprising algae, fungi and bacteria establish on rocks and process dissolved organic matter. Invertebrates include shredders (consuming coarse particles), grazers (consuming biofilm), collectors and filter feeders (consuming benthic and suspended fine particles, respectively), and predators. Many benthic macroinvertebrates, mostly insects, have aquatic larvae and terrestrial adults. Filter feeders have traits adapted to swift flows, allowing them to hold fast to substrates while capturing resources, while benthic bryophytes provide shelter for other organisms. Fish are typically small predators of aquatic invertebrates and insects on the water surface. Birds typically have specialised foraging behaviours (e.g. dippers and kingfishers). Trophic cascades involving rapid algal growth, invertebrate grazers and fish are common.

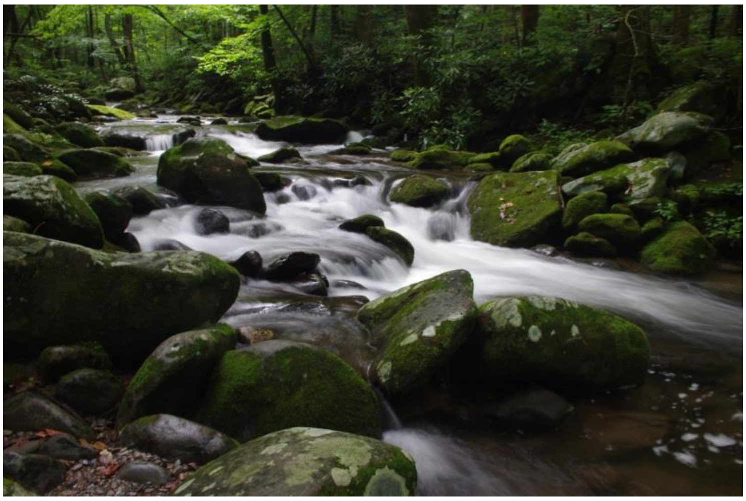

Appalachian Mountain Stream in the Spring.
Credit: Samuel H Austin / Virginia Water Science Center

Ecological drivers: Upland streams have flash flow regimes with high velocity and relatively low, but variable perennial volume. Turbulence sustains highly oxygenation. Groundwater-delivered subsidies support streamflow, with up to 50% of summer flow and 100% of winter flow originating as groundwater. This modulates stream temperatures, keeping temperatures lower in summer and higher in winter; and deliver nutrients, especially if there are N-fixing plants, along the groundwater flow path. They flow down moderate to steep slopes causing considerable erosion and sediment transport. These factors drive nutrient and organic matter transport downstream. Flow volume and variability, including periodic flood regimes, depend on rainfall seasonality, snowmelt from cold-climate catchments, as well as catchment size. Peat-rich catchments feed dark dystrophic waters to the streams.

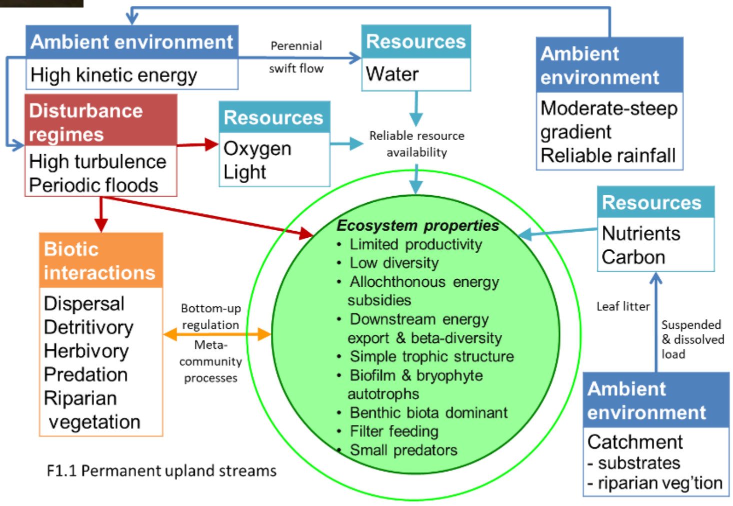

Distribution: High proportion of global stream length. In steep to moderate terrain throughout the humid tropical and temperate zones, rarely extending to boreal latitudes.

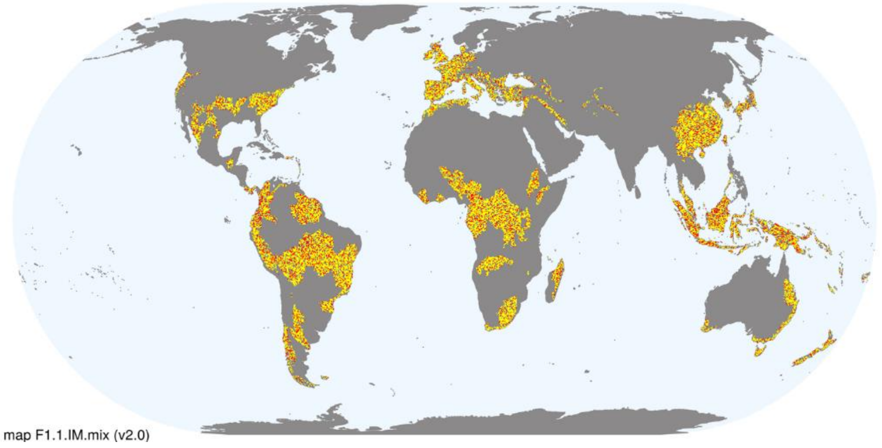

References:

Giller PS., Malmqvist B (1998) *The biology of streams and rivers* Oxford University Press, Oxford.

Meyer JL, Strayer DL, Wallace JB, Eggert SL, Helfman GS, Leonard NE (2007) The contribution of headwater streams to biodiversity in river networks. *Journal of the American Water Resources Association* 43: 86–103.

Meyer JL, Wallace JB (2001) *Lost linkages and lotic ecology: rediscovering small streams* Blackwell, Oxford.

Map is for illustrative purposes only and does not support spatial analyses unless formally validated.

F1.2 Permanent lowland rivers

Ecosystem properties: Small-medium lowland rivers (stream orders 4-9) are productive depositional ecosystems with trophic webs that are less diverse than large lowland rivers (F1.7). Macrophytes rooted in benthos or along the river margins contribute most primary production, but allochthonous inputs from floodplains and upper catchments generally dominate energy flow in the system. The biota tolerates a range of temperatures, which vary with catchment climate. Aquatic biota have physiological, morphological and even behavioural adaptations to lower oxygen concentrations, which may vary seasonally and diurnally. Zooplankton can be abundant in slower deeper rivers. Sessile (e.g. mussels) and scavenging (e.g. crayfish) macroinvertebrates are associated with the hyporheic zone and structurally complex microhabitats in moderate flow environments, including fine sediment and woody debris. Fish communities are diverse and may contribute to complex trophic networks. They include large predatory fish (e.g. sturgeons), smaller predators of invertebrates, herbivores, and detritivores. The feeding activities and movement of piscivorous birds (e.g. cormorants), diadromous fish (seawater-freshwater migrants), mammals (e.g. otters), and reptiles (e.g. turtles) extend trophic network beyond instream waters. Riparian zones vary in complexity from forested banks to shallow areas where emergent, floating and submerged macrophyte vegetation grows. Intermittently connected oxbow lakes or billabongs increase the complexity of associated habitats, providing more lentic waters for a range of aquatic fauna and flora.

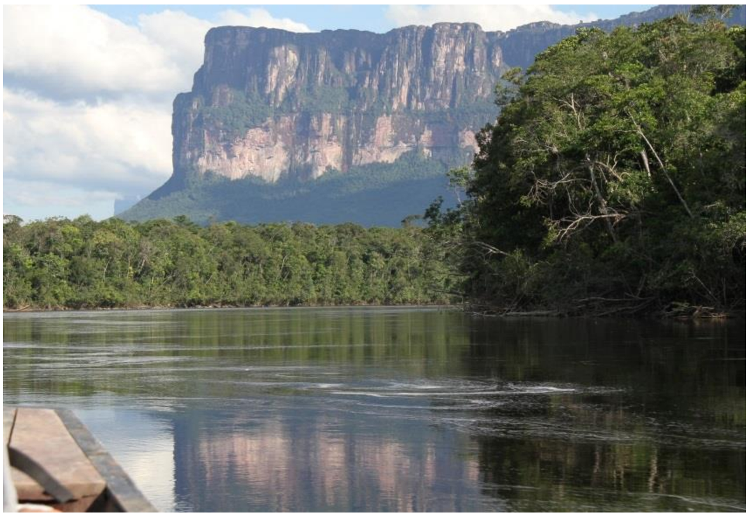

Rio Carrao, Venezuela.
Credit: David Keith

Ecological drivers: These rivers are distinguished by shallow gradients, low turbulence, low to moderate flow velocity and moderate flow volumes (<10,000m3/s). Flows are continuous but may vary seasonally depending on catchment precipitation. This combination of features is most common at low altitudes below 200 m and rarely occurs above 1,500 m. River channels are tens to a few hundred metres wide and up to tens of metres deep with mostly soft sediment substrates. They are dominated by depositional processes. Surface water and groundwater mix in the alluvium in the hyporheic zone, which plays an important role in nutrient cycling. Overbank flows increase turbulence and turbidity. Locally or temporally important erosional processes redistribute sediment and produce geomorphically dynamic depositional features (e.g. braided channels and point bars). Nutrient levels depend on riparian/floodplain inputs and vary with catchment geochemistry. Oxygen and temperatures also vary with climate and catchment features. For catchments with extensive peatlands, waters may be tannin-rich, poorly oxygenated, acidic and dark, thus reducing productivity and diversity.

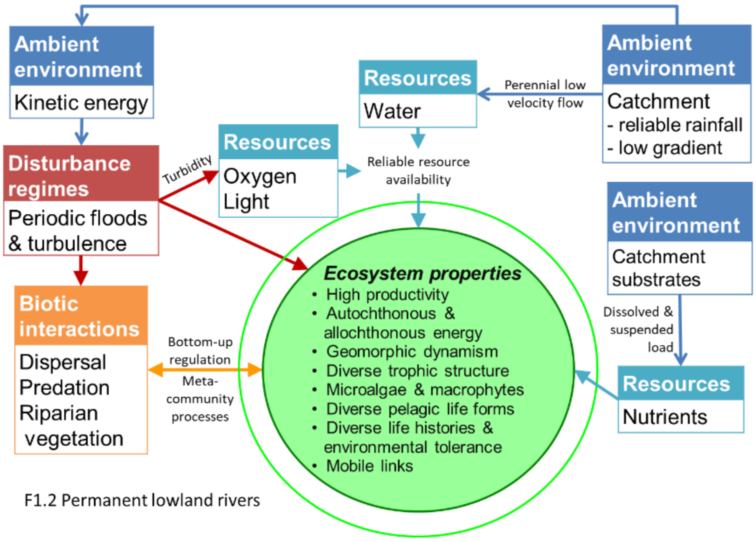

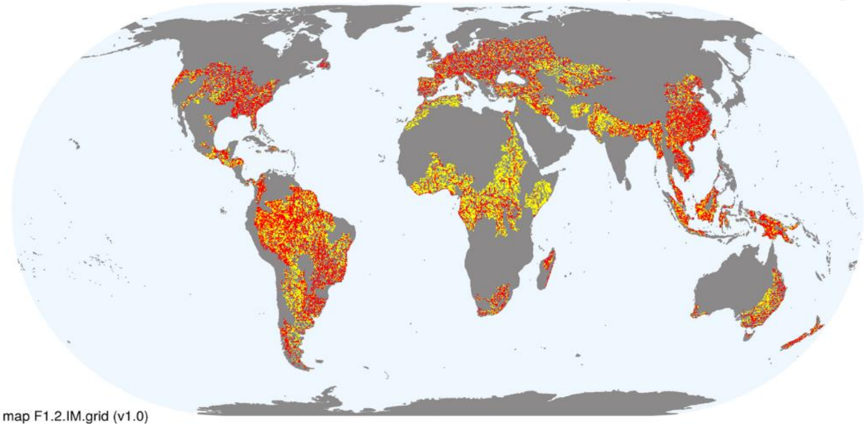

map F1.2.IM.grid (v1.0)

Distribution: Distributed throughout tropical and temperate lowlands but very uncommon in arid zones. They are absent from boreal zones, where they are replaced by F1.3.

References:

Tockner K, Malard, F, Ward JV (2000) An extension of the flood pulse concept. *Hydrological Processes* 14: 2861-2883.

Map is for illustrative purposes only and does not support spatial analyses unless formally validated.

Contributors: RT Kingsford, PS Giller, DA Keith

F1.3 Freeze-thaw rivers and streams

Ecosystem properties: In seasonally cold montane and boreal environments, the surfaces of both small streams and large rivers freeze in winter. These systems have relatively simple trophic networks with low functional and taxonomic diversity, but the biota may include local endemics. In small, shallow streams, substrate algae are the principal autotrophs, while phytoplankton occur in larger rivers and benthic macrophytes are rare. All are seasonally inactive or curtailed when temperatures are cold and surface ice reduces light penetration through the water. Bottom-up regulatory processes dominate. Subsidies of dissolved organic carbon and nutrients from spring meltwaters and riparian vegetation along smaller streams are crucial to maintaining the detritivores that dominate the trophic network. Overall decomposition rates of coarse particles are low, but can exceed rates per degree day in warmer climates as the fauna are adapted to cold temperatures. Microbial decomposers often dominate small streams, but in larger rivers, the massive increase in fine organic particles in spring meltwaters can support abundant filter feeders which consume huge quantities of suspended particles and redeposit them within the river bed. Resident invertebrates survive cold temperatures through dormant life stages, extended life cycles and physiological adaptations. Vertebrate habitat specialists (e.g. dippers, small fish, beavers, and otters) tolerate low temperatures with traits such as subcuticular fat, thick hydrophobic, and/or aerated fur or feathers. Many fish disperse from frozen habitat to deeper water refuges during the winter (e.g. deep pools) before foraging in the meltwater streams from spring to autumn. In the larger rivers, fish, and particularly migratory salmonids returning to their natal streams and rivers for breeding, are a food source for itinerant terrestrial predators such as bears. When they die after reproduction, their decomposition in turn provides huge inputs of energy and nutrients to the system.

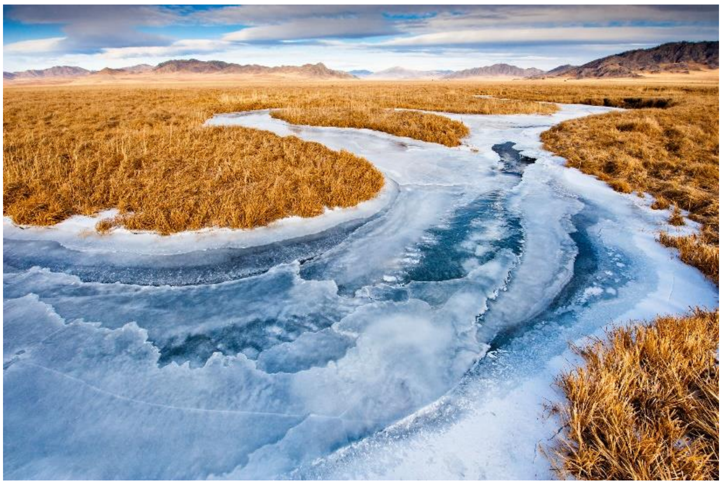

The frozen River Kan running through the Siberian steppe, Russia.
Credit: Anton Agarov / Getty Images

Ecological drivers: These rivers experience low winter temperatures and seasonal freeze-thaw regimes. Winter freezing is generally limited to the surface but can extend to the substrate forming ‘anchor ice’. Flows may continue below the ice or may be intermittent in smaller streams or dry climates. Freezing reduces resource availability by reducing nutrient inputs, allochthonous organic matter and light penetration through the water. Light may also be attenuated at high latitudes and by high turbidity in erosional streams. Meltwaters drive increased flow and flooding in spring and summer. Carbon and nutrient concentrations are greatest during spring floods, and pH tends to decrease with flow during spring and autumn. When catchments include extensive peatlands, waters may be tannin-rich, acidic and dark, thereby reducing light penetration and productivity.

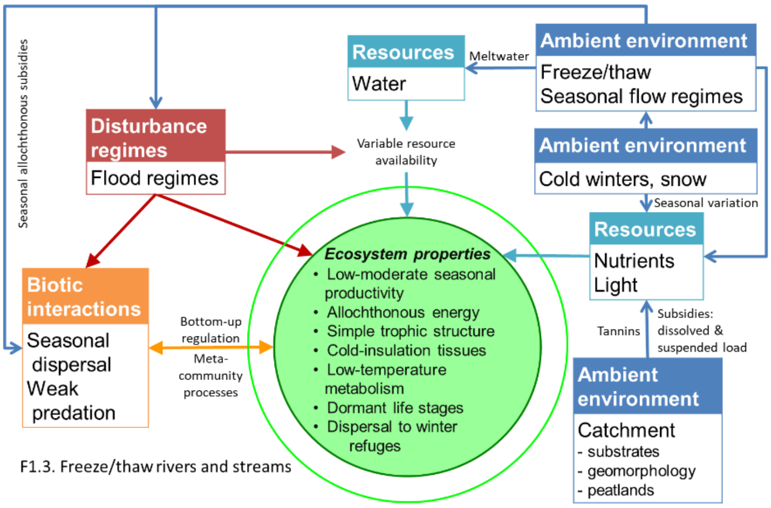

F1.3. Freeze/thaw rivers and streams

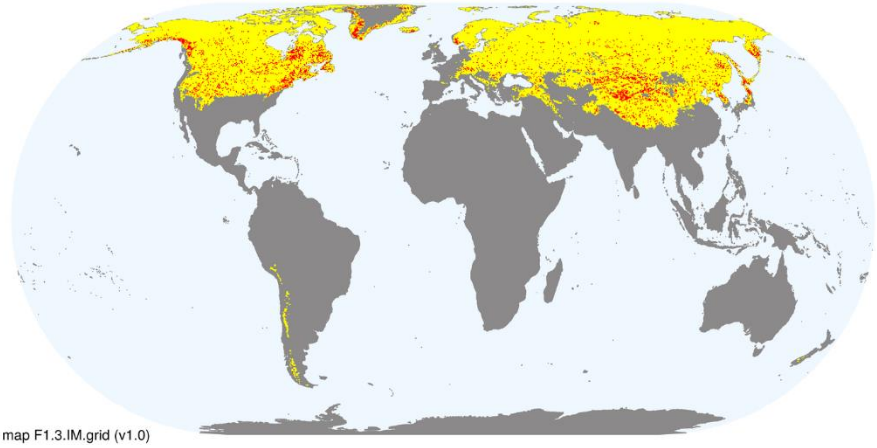

map F1.3.IM.grid (v1.0)

Distribution: Restricted to boreal, subarctic, alpine and subalpine regions, with limited examples in the subantarctic and Antarctica.

References:

Guo LD, Cai YH, Belzile C, Macdonald RW (2012) Sources and export fluxes of inorganic and organic carbon and nutrient species from the seasonally ice-covered Yukon River. *Biogeochemistry* 107: 187-206.

Olsson TI (1981) Overwintering of benthic macroinvertebrates in ice and frozen sediment in a North Swedish river. *Ecography* 4: 161-166.

Map is for illustrative purposes only and does not support spatial analyses unless formally validated.

Contributors: RT Kingsford, B Robson, PS Giller, AH Arthington, M Kelly-Quinn, DA Keith

F1.4 Seasonal upland streams

Ecosystem properties: Upland streams (orders 1-4) with highly seasonal flows generally have low to moderate productivity and a simpler trophic structure than lowland rivers. They tend to be shallow, hence benthic algae are major contributors to in-stream food webs and productivity, but riparian zones and catchments both contribute allochthonous energy and organic carbon through leaf fall, which may include an annual deciduous component. Primary production also varies with light availability and flow. Taxonomic diversity varies between streams, but can be lower than permanent streams and relatively high in endemism. Traits that enable biota to persist in narrow and shallow channels with large seasonal variations in flow velocity, episodes of torrential flow, and seasonal desiccation include small body sizes (especially in resident fish), dormant life phases and/or burrowing (crustaceans), omnivorous diets and high dispersal ability, including seasonal migration. Compared to lowland rivers, the trophic structure has a higher representation of algal and omnivorous feeders and low numbers of larger predators. Birds show specialist feeding strategies (e.g. dippers). Diversity and abundance of invertebrates and their predators (e.g. birds) fluctuate in response to seasonal flood regimes.

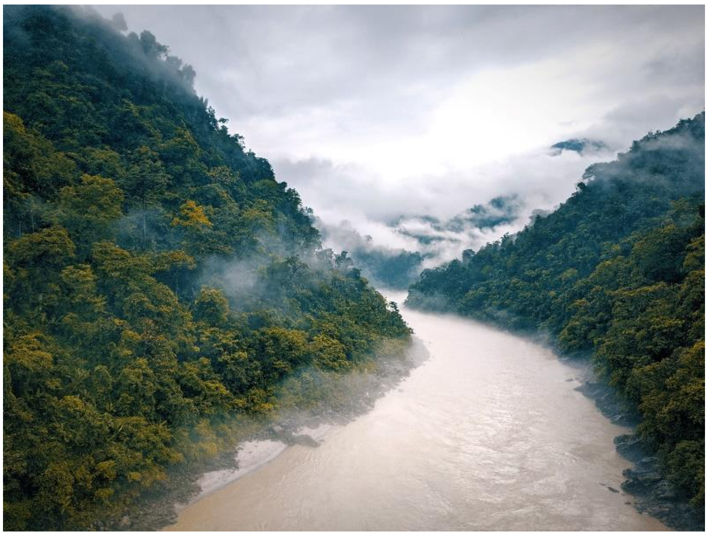

Monsoonal flow in upland stream, Mussoorie, India.
Credit: Abhraneel Basak / EyeEm / Getty Images

Ecological drivers: Flow and flood regimes in these rivers are highly variable between marked wet and dry seasons, with associated changes in water quality as solute concentration varies with volume. They may be perennial, with flows much-reduced in the dry season, or seasonally intermittent with flows ceasing and water persisting in isolated stagnant pools. Channels are narrow with steep to moderate gradients, seasonally high velocity and sometimes large volumes of water, producing overbank flows. This results in considerable turbulence, turbidity, and erosion during the wet season and coarse substrates (cobbles and boulders). Seasonal floods are critical to allochtonous subsidies and downstream exports of organic matter and nutrients.

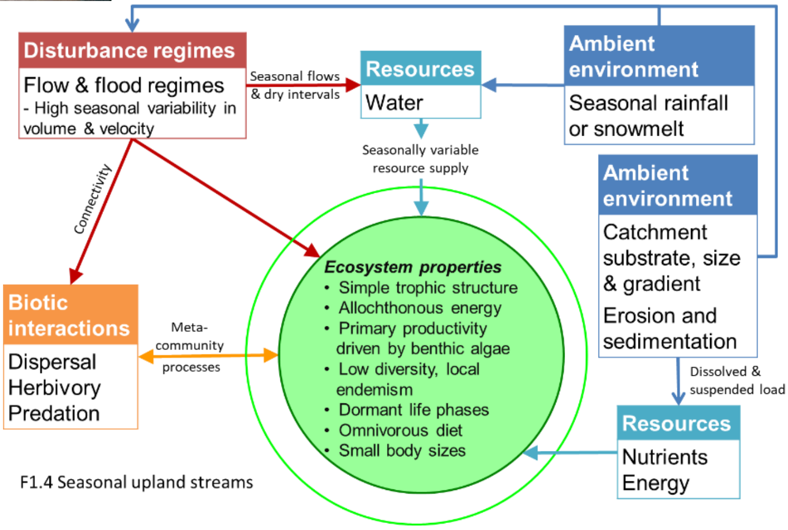

Distribution: Elevated regions in seasonal tropical, subtropical and temperate climates worldwide.

References:

Datry T, Bonada N, Boulton A (2017) *Intermittent rivers and ephemeral streams: ecology and management* (Academic Press: Burlington).

Davies PM, Bunn SE, Hamilton SK (2008) Primary production in tropical streams and rivers. *Tropical stream ecology* (Ed. D Dudgeon), pp. 23–42. Academic Press, London.

de Carvalho RA, Tejerina-Garro FL (2018) Headwater-river gradient: trait-based approaches show functional dissimilarities among tropical fish assemblages. *Marine and Freshwater Research* 69: 574-584.

Jardine RD, Bond NR, Burford MA, Kennard MJ, Ward DP, Bayliss P, Davies PM, Douglas MM, Hamilton SK, Melack JM, Naiman RJ, Pett NE, Pusey BJ, Warfe DM, Bunn SE (2015) Does flood rhythm drive ecosystem responses in tropical riverscapes?. *Ecology* 96: 684–692.

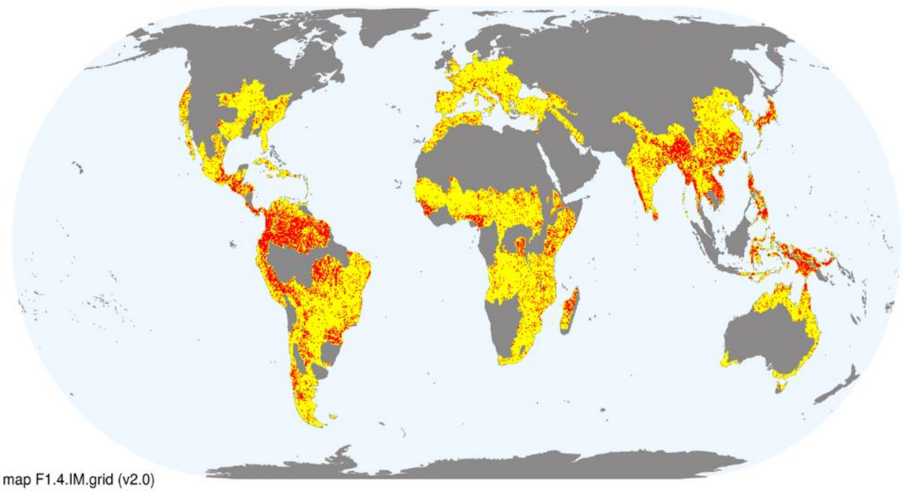

map F1.4.IM.grid (v2.0)

Map is for illustrative purposes only and does not support spatial analyses unless formally validated.

Contributors: RT Kingsford, B Robson, PS Giller, AH Arthington, DA Keith

F1.5 Seasonal lowland rivers

Ecosystem properties: These large riverine systems (stream orders 5-9) can be highly productive with trophic structures and processes shaped by seasonal hydrology and linkages to floodplain wetlands. In combination with biophysical heterogeneity, this temporal variability promotes functional diversity in the biota. Although trophic networks are complex due to the diversity of food sources and the extent of omnivory amongst consumers, food chains tend to be short and large mobile predators such as otters, large piscivorous waterbirds, sharks, dolphins, and crocodilians (in the tropics) can have a major impact on the food webs. Benthic algae are key contributors to primary productivity, although macrophytes become more important during the peak and late wet season when they also provide substrate for epiphytic algae. Rivers receive very significant resource subsidies from both algae and macrophytes on adjacent floodplains when they are connected by flows. Enhanced longitudinal hydrological connectivity during the wet season enables fish and other large aquatic consumers to function as mobile links, extending floodplain and estuarine resource subsidies upstream. Life cycle processess including reproduction, recruitment, and dispersal in most biota are tightly cued to seasonally high flow periods, often with floodplain nursery areas for river fish, amphibians and larger invertebrates.

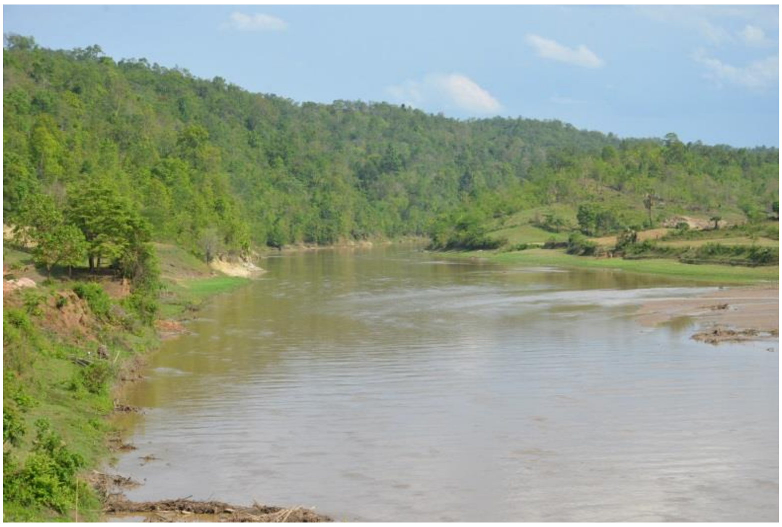

Patalon Chaung, upstream of Migyaungpon, Myanmar.
Credit: David Keith

Ecological drivers: These rivers are driven by cyclical, seasonal flow regimes. High-volume flows and floods occur during summer in the tropics or winter-spring at temperate latitudes, with two peaks in some areas. A decline of flows and reduced flood residence times during the transition to the dry season is followed by low and disconnected flows during the dry season. Turbidity, light availability, erosion, sedimentation, lateral and longitudinal connectivity, biological activity, dissolved oxygen and solute concentrations all vary with this seasonal cycle. The inter-annual variability of this pattern depends on the catchment precipitation and sources of inflow that offset or mute the influences of rainfall seasonality (e.g. snow melt in South Asia). Streams may be single, multi-channelled or complex anabranching systems.

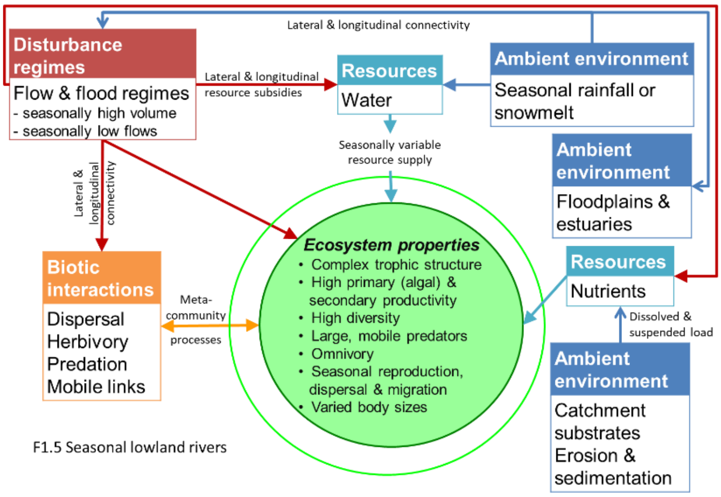

Distribution: Tropical, subtropical and temperate lowlands with seasonal inflow patterns worldwide.

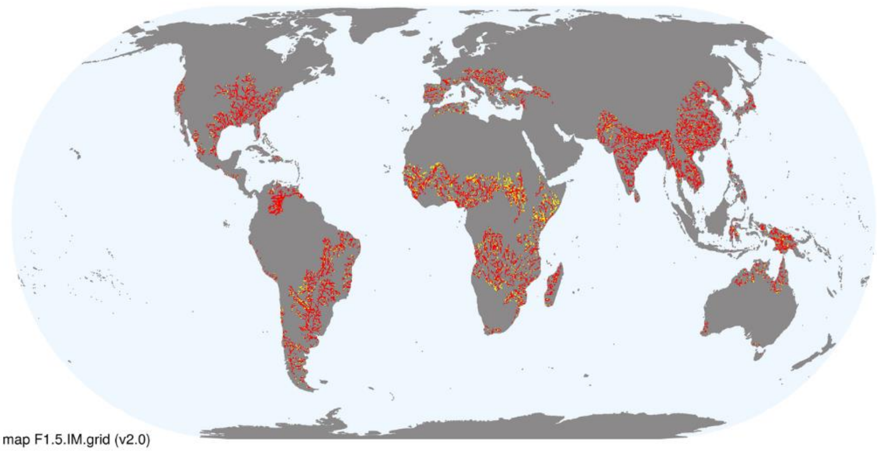

References:

Datry T, Bonada N, Boulton A (2017) *Intermittent rivers and ephemeral streams: ecology and management* (Academic Press: Burlington).

Douglas MM, Bunn SE, Davies PM (2005) River and wetland food webs in Australia's wet-dry tropics: general principles and implications for management. *Marine and Freshwater Research* 56: 329-342.

Layman CA, Winemiller KO, Arrington DA, Jepsen DB (2005) Body size and trophic position in a diverse tropical food web. *Ecology* 86:2530-2535.

Map is for illustrative purposes only and does not support spatial analyses unless formally validated.

F1.6 Episodic arid rivers

Ecosystem properties: Episodic rivers have high temporal variability in flows and resource availability, shaping a low-diversity biota with periodically high abundance of some organisms. Productivity is episodically high and punctuated by longer periods of low productivity (i.e. boom-bust dynamics). The trophic structure can be complex and dominated by autochthonous primary production. Even though riparian vegetation is sparse, allochthonous inputs from connected floodplains may be important. Top-down control of ecosystem structure is evident in some desert streams. Episodic rivers are hotspots of biodiversity and ecological activity in arid landscapes, acting as both evolutionary and ecological refuges. Most biota have ruderal life cycles, dormancy phases, or high mobility enabling them to tolerate or avoid long, dry periods and to exploit short pulses of high resource availability during flooding. During dry periods, many organisms survive as dormant life phases (e.g. eggs or seeds), by reducing metabolism, or by persisting in perennial refugia (e.g. waterholes, shallow aquifers). They may rapidly recolonise the channel network during flow (networkers). Waterbirds survive dry phases by moving elsewhere, returning to breed during flows. The abundance of water, nutrients and food during flows and floods initiates rapid primary production (especially by algae), breeding and recruitment. Zooplankton are abundant in slower reaches during periods of flow. Macroinvertebrates such as sessile filter-feeders (e.g. mussels) and scavengers (e.g. crayfish) may occur in moderate flow environments with complex microhabitats in fine sediment and amongst woody debris. Assemblages of fish and amphibians are dominated by small body sizes. Most fish species use inundated floodplains in larval, juvenile and mature life stages, and produce massive biomass after large floods. Organisms generally tolerate wide ranges of temperature, salinity, and oxygen.

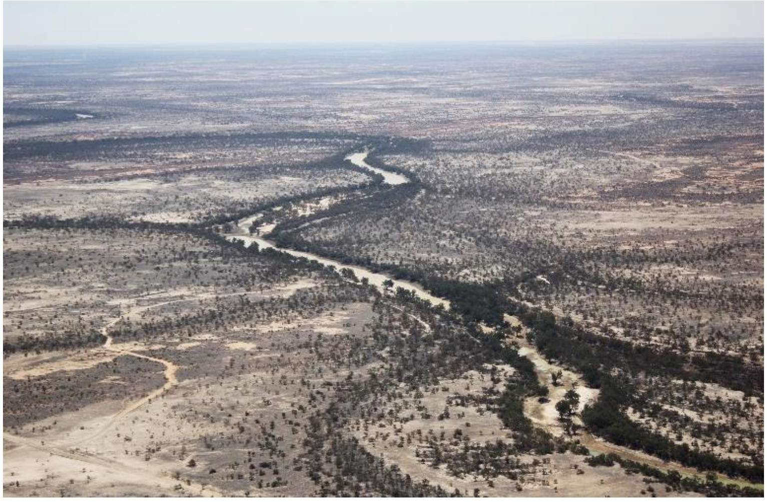

Cooper Creek in central Australia.
Credit: Richard Kingsford

Ecological drivers: These mostly lowland systems are distinguished by highly episodic flows and flood regimes that vary with catchment size and precipitation. High-volume, short duration flows (days to weeks, rarely months) punctuate long dry periods fill channels and flood wetlands. Low elevational gradients and shallow channels result in low turbulence and low to moderate flow velocity. Lowland stream channels are broad, flat, and often anastomising, with mostly soft sandy sediments. Groundwater is usually within rooting zones of perennial plants, which may establish in channels after flow events. Sediment loads drive periodically high turbidity. Locally or temporally important erosional processes have roles in geomorphic dynamism redistributing sediment in depositional features (e.g. braided channels and point bars). Upland streams are prone to erosive flash floods. High nutrient levels are due to large catchments and riparian inputs but depend on catchment geochemistry. These rivers often flow over naturally saline soils. Salinity can thus be high and increases in drying phases.

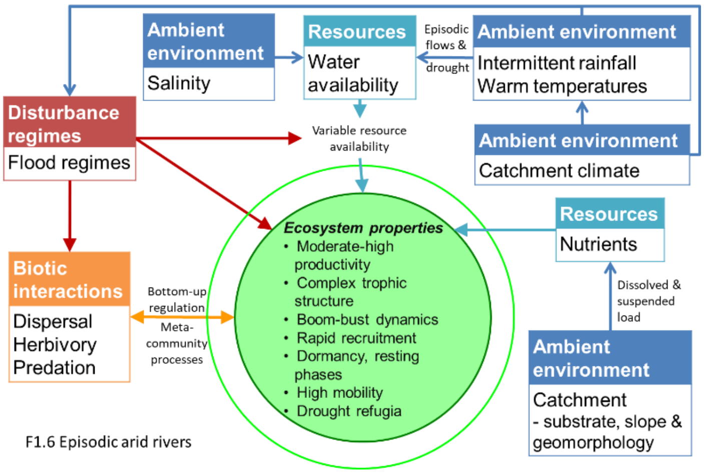

F1.6 Episodic arid rivers

Distribution: Arid and semi-arid mid-latitudes, in lowlands, and some uplands, but rarely above 1,500 m elevation.

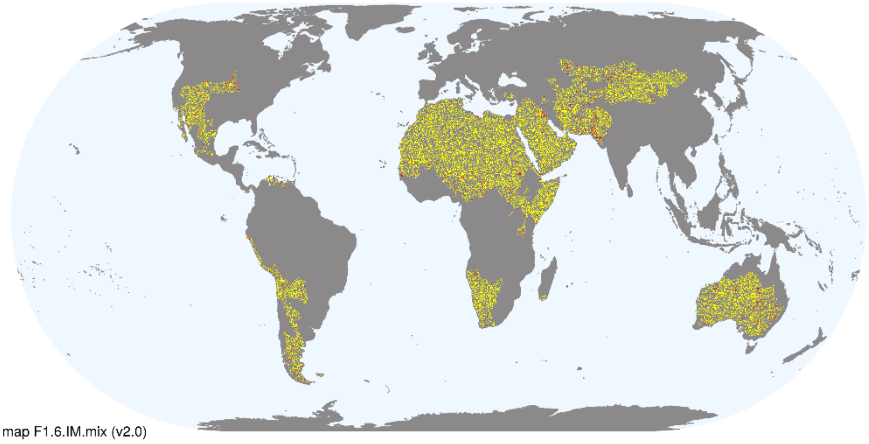

map F1.6.IM.mix (v2.0)

References:

Kingsford RT (2006) *Ecology of desert rivers* Cambridge University Press, Cambridge.

Sheldon et al. (2010) Ecological roles and threats to aquatic refuges in arid landscapes: dryland river waterholes. *Marine & Freshwater Research* 61, 885-895.

Map is for illustrative purposes only and does not support spatial analyses unless formally validated.

F1.7 Large lowland rivers

Ecosystem properties: Large lowland rivers (typically stream orders 8-12) are highly productive environments with complex trophic webs which are supported by very large flow volumes. Primary production is mostly from autochthonous phytoplankton and riparian macrophytes, with allochthonous inputs from floodplains and upper catchments generally dominating energy flow in the system. The fauna includes a significant diversity of pelagic organisms. Zooplankton are abundant, while sessile (e.g. mussels), burrowing (e.g. annelids) and scavenging (e.g. crustaceans) macroinvertebrates occur in the fine sediment and amongst woody debris. Fish communities are diverse and contribute to complex trophic networks. They include large predatory fish (e.g. freshwater sawfish, Pirhana, Alligator Gar) and in some rivers endemic River Dolphins, smaller predators of invertebrates (benthic and pelagic feeders), phytoplankton herbivores, and detritivores. The feeding activities and movement of semi-aquatic piscivorous birds (e.g. cormorants), mammals (e.g. otters), and reptiles (e.g. turtles, crocodilians) connect the trophic network to other ecosystems beyond instream waters. Riparian and large floodplain zones vary in complexity from forested banks, to productive lentic oxbow lakes and extensive and complex flooded areas where emergent and floodplain vegetation grows (e.g. reeds and macrophytes, shrubs, trees). Riparian zones can be complex but have less direct influence on large rivers than on smaller river ecosystems.

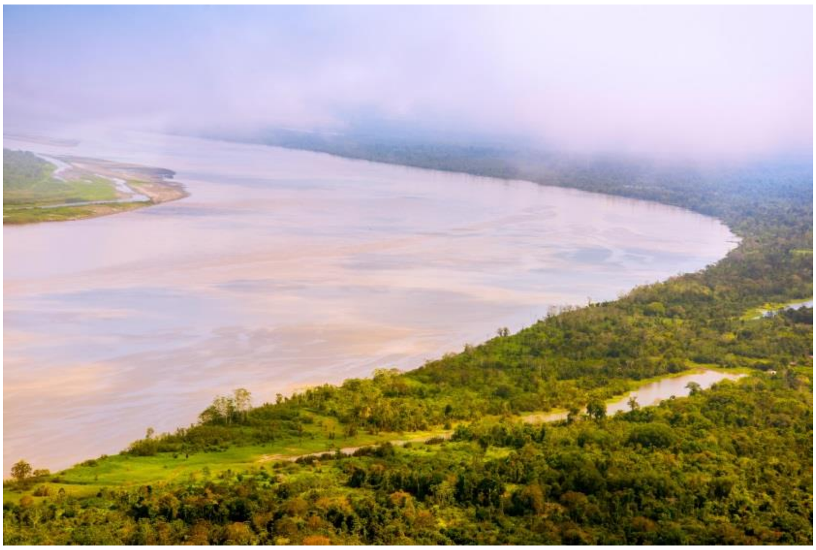

Amazon River near Iquitos, Peru.
Credit: Amazon Images / Alamy Stock

Ecological drivers: These rivers have shallow gradients with low turbulence, low to moderate flow velocity and very high flow volumes (>10,000m3/s), which are continuous but may vary seasonally depending on catchment area and precipitation (e.g. Congo up to 41,000 m3/s, Amazon up to 175,000 m3/s). River channels are wide (e.g. Amazon River; 11 km in dry season, up to 25km when flooded at its widest point) and deep (e.g. Congo up to 200m; Mississippi up to 60m) with mostly soft sediment substrates. They are dominated by depositional processes so turbidity may be high. Overbank flows increase turbulence and turbidity. Locally or temporally important erosional processes redistribute sediment and produce geomorphically dynamic depositional features (e.g. braided channels, islands and point bars). Nutrient levels are high due to large catchments and riparian/floodplain inputs but vary with catchment geochemistry. Moderate water temperatures are buffered due to large catchments.

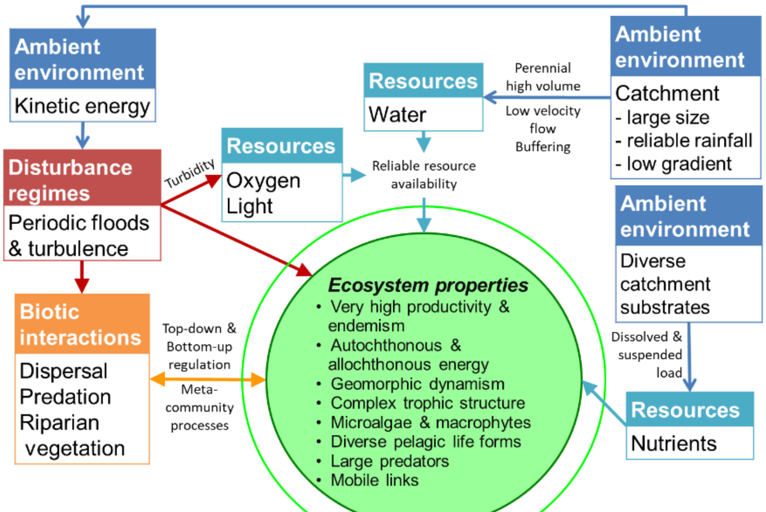

F1.7 Big lowland rivers

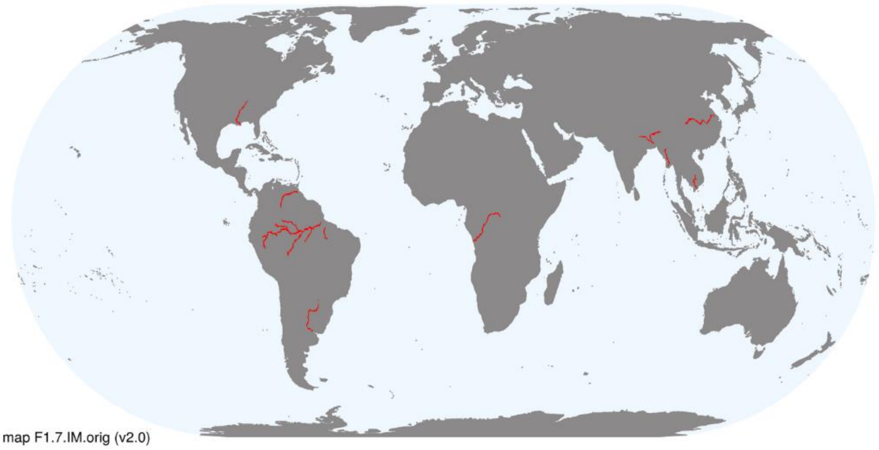

map F1.7.IM.orig (v2.0)

Distribution: Tropical and subtropical lowlands, with a few extending to temperate zones. They are absent from arid regions, and in boreal zones are replaced by F1.3.

References:

Ashworth, PJ, Lewin, J (2012) How do big rivers come to be different?. Earth-Science Reviews* 114, 84-107.

Best, J (2019) Anthropogenic stresses on the world's big rivers. *Nature Geoscience* 12,7-2.

Map is for illustrative purposes only and does not support spatial analyses unless formally validated.

F2. Lakes biome

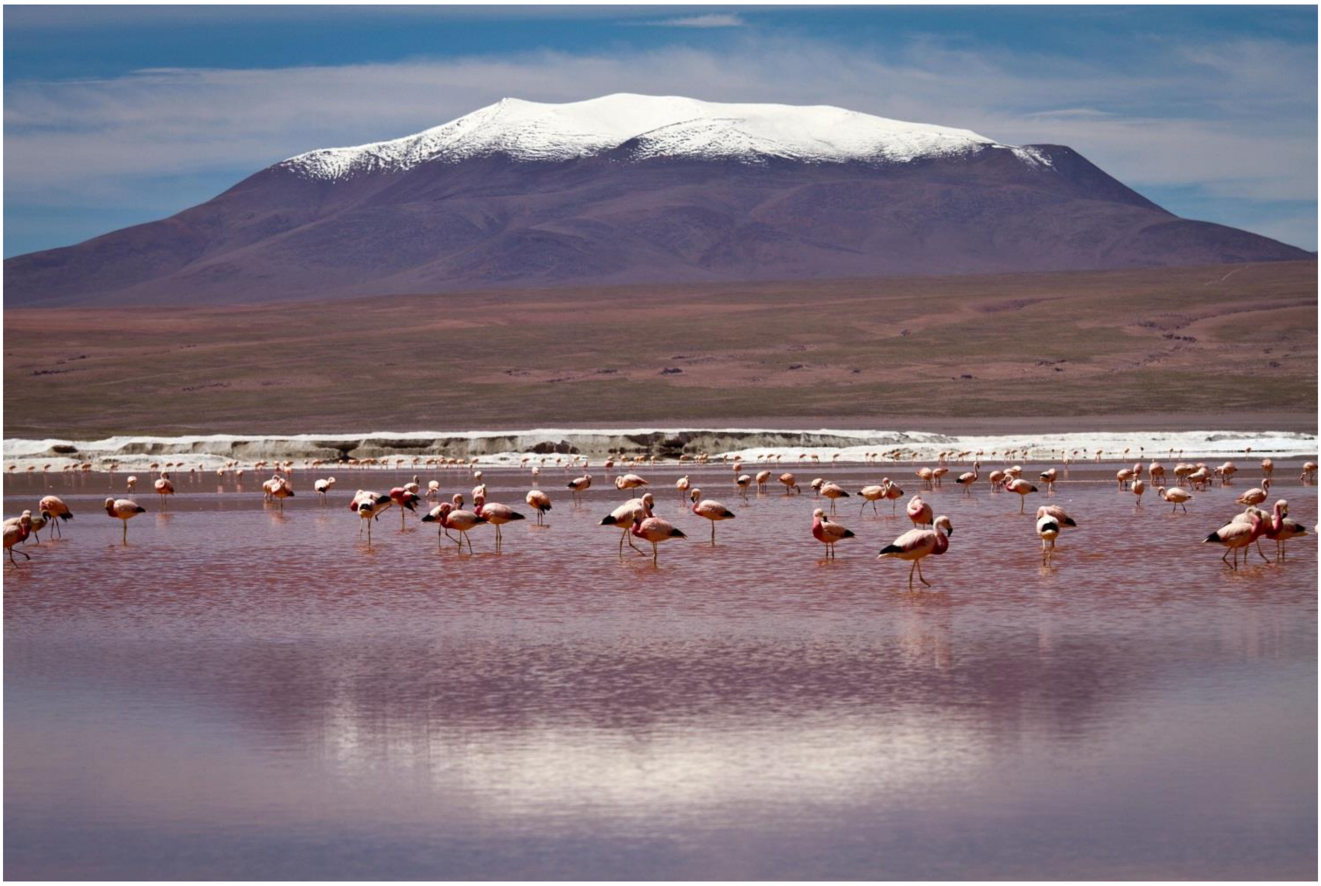

Andes Mountain Lake, Bolivia.

Credit: Sylvain Didier / Alamy Stock

The Lakes biome includes lentic ecosystems defined by their still waters. They vary in area, depth, water regime, and connectivity to other aquatic systems across a global distribution.

Gradients in water regimes, temperature, lake size, and salinity exert critical influences on the function, productivity, diversity, and trophic structure of lake ecosystems. Water regimes vary from permanent open waters to seasonal or episodic filling and drying on interannual time scales. Lakes span global climatic gradients, which influence their water regimes through catchment precipitation and evapotranspiration rates, as well as the seasonal freeze-thaw cycles of lake surfaces along latitudinal and altitudinal temperature gradients.

The azonal character of the Lakes biome, however, is due to the buffering of climatic influences by groundwater, geomorphology, and substrate. This is most evident in the water regimes of artesian springs, oases, and geothermal wetlands, as their water sources are largely independent of climate. Lake and catchment substrates influence nutrient stocks and salinity, but concentrations may vary temporally depending on water regimes and mixing. Deeper and freeze-thaw lakes are often characterised by stratification, producing depth gradients in nutrient and oxygen availability and temperatures. The deepest lakes extend to the aphotic zone. Productivity is determined by allochthonous inputs from the catchments and autochthonous inputs from phytoplankton, periphyton (i.e. biofilms), and submerged, floating and emergent macrophytes.

Trophic webs tend to increase in size and complexity with lake size due to increased resource availability and niche diversity, but small shallow lakes have greater diversity than small deep lakes due to habitat heterogeneity and light penetration to the bottom allowing development of benthic macrophytes and associated biota. Salt lakes may have high productivity but simple trophic structures, with high abundances of few species. Invertebrate detritivores consume fragments of organic matter, providing resources for macroinvertebrates, fish, waterbirds, reptiles, and mammals. Species traits appear to be strongly influenced by environmental filtering by the water regime (e.g. cold tolerance and seasonal dormancy occurs in freeze-thaw lakes, and desiccation tolerance and dormant life stages dominate in ephemeral lakes) and water chemistry (e.g. tolerance to salinity in salt lakes).

Contributors: RT Kingsford, R Mac Nally, LJ Jackson, F Essl, K Irvine, DA Keith

F2.1 Large permanent freshwater lakes

Ecosystem properties: Large permanent freshwater lakes, generally exceeding 100 km^2, are prominent landscape features connected to one or more rivers either terminally or as flow-through systems. Shoreline complexity, depth, bathymetric stratification, and benthic topography promote niche diversity and zonation. High niche diversity and large volumes of permanent water (extensive, stable, connected habitat) support complex trophic webs with high diversity and abundance. High primary productivity may vary seasonally, driving succession, depending on climate, light availability, and nutrient regimes. Autochthonous energy from abundant pelagic algae (mainly diatoms and cyanobacteria) and from benthic macrophytes and algal biofilms (in shallow areas) is supplemented by allochthonous inflows that depend on catchment characteristics, climate, season, and hydrological connectivity. Zooplankton, invertebrate consumers, and herbivorous fish sustain high planktonic turnover and support upper trophic levels with abundant and diverse predatory fish, amphibians, reptiles, waterbirds, and mammals. This bottom-up web is coupled to a microbial loop, which returns dissolved organic matter to the web (rapidly in warm temperatures) via heterotrophic bacteria. Obligate freshwater biota in large lakes, including aquatic macrophytes and macroinvertebrates (e.g. crustaceans) and fish, often display high catchment-level endemism, in part due to long histories of environmental variability in isolation. Marked niche differentiation in life history and behavioural feeding and reproductive traits enables sympatric speciation and characterises the most diverse assemblages of macroinvertebrates and fish (e.g. ~500 cichlid fish species in Lake Victoria). Large predators are critical in top-down regulation of lower trophic levels. Large lake volume buffers against nutrient-mediated change from oligotrophic to eutrophic states. Recruitment of many organisms is strongly influenced by physical processes such as large inflow events. Mobile birds and terrestrial mammals use the lakes as breeding sites and/or sources of drinking water and play key roles in the inter-catchment transfer of nutrients and organic matter and the dispersal of biota.

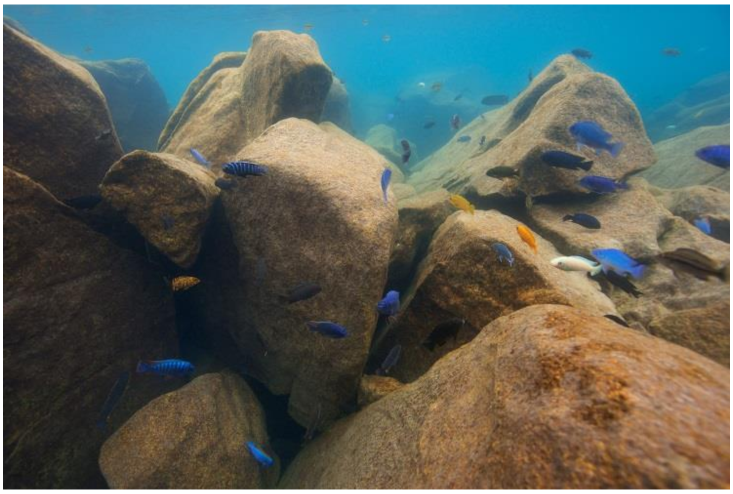

High cichlid fish diversity in Lake Malawi, Africa.
Credit: Michel Roggo

Ecological drivers: Large water volumes influence resource availability, environmental stability (through thermal buffering), and niche diversity. Water is from catchment inflows, which may vary seasonally with climate. Large lakes influence regional climate through evaporation, cooling, and convection feedbacks. These processes also influence nutrient availability, along with catchment and lake substrates and vertical mixing. Mixing may be monomictic (i.e. annual) or meromictic (i.e. seldom), especially in large tropical lakes, depending on inflow, depth, wind regimes, and seasonal temperature variation. Light varies with lake depth, turbidity, cloud cover, and latitude.

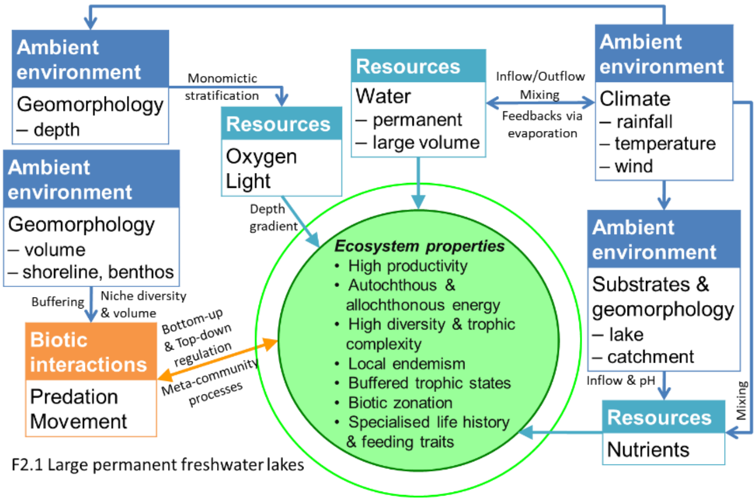

F2.1 Large permanent freshwater lakes

Distribution: Humid temperate and tropical regions on large land masses.

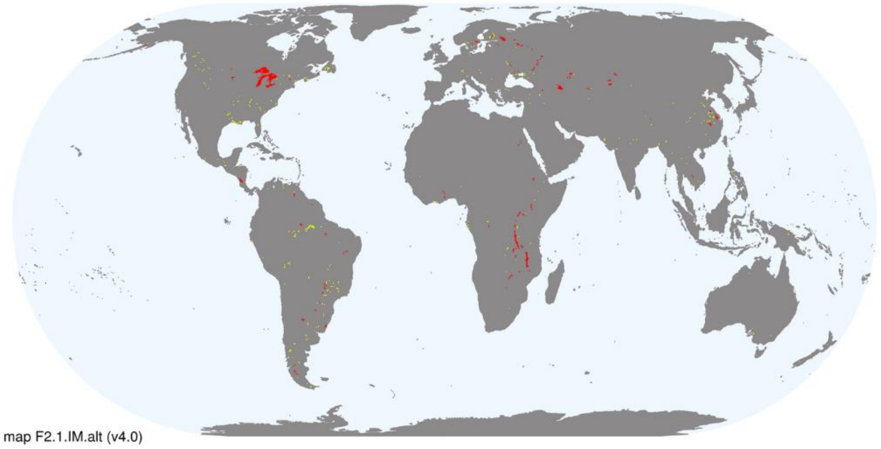

Map is for illustrative purposes only and does not support spatial analyses unless formally validated.

References:

Ludsin SA, DeVanna KM, Smith REH (2014) Physical-biological coupling and the challenge of understanding fish recruitment in freshwater lakes. *Canadian Journal of Fisheries and Aquatic Sciences* 71: 775-794.

Pennisi E (2018) Hybrids spawned Lake Victoria's rich fish diversity. *Science* 361: 539-539.

Contributors: RT Kingsford, B Robson, R MacNally, L Jackson, F Essl,
M Kelly-Quinn, K Irvine, S Bertilsson, DA Keith

F2.2 Small permanent freshwater lakes

Ecosystem properties: Small permanent freshwater lakes, pools or ponds are lentic environments with relatively high perimeter-to-surface area and surface-area-to-volume ratios. Most are <1 km^2 in area, but this functional group includes lakes of transitional sizes up to 100 km2, while the largest lakes (>100 km^2) are classified in F2.1. Niche diversity increases with lake size. Although less diverse than larger lakes, these lakes may support phytoplankton, zooplankton, shallow-water macrophytes, invertebrates, sedentary and migratory fish, reptiles, waterbirds, and mammals. Primary productivity, dominated by cyanobacteria, algae, and macrophytes, arises from allochthonous and autochthonous energy sources, which vary with lake and catchment features, climate, and hydrological connectivity. Productivity can be highly seasonal, depending on climate, light, and nutrients. Permanent water and connectivity are critical to obligate freshwater biota, such as fish, invertebrates, and aquatic macrophytes. Trophic structure and complexity depend on lake size, depth, location, and connectivity. Littoral zones and benthic pathways are integral to overall production and trophic interactions. Shallow lakes tend to be more productive (by volume and area) than deep lakes because light penetrates to the bottom, establishing competition between benthic macrophytes and phytoplankton, more complex trophic networks and stronger top-down regulation leading to alternative stable states and possible regime shifts between them. Clear lakes in macrophyte-dominated states support higher biodiversity than phytoplankton-dominated eutrophic lakes. Deep lakes are more dependent on planktonic primary production, which supports zooplankton, benthic microbial and invertebrate detritivores. Herbivorous fish and zooplankton regulate the main primary producers (biofilms and phytoplankton). The main predators are fish, macroinvertebrates, amphibians and birds, many of which have specialised feeding traits tied to different habitat niches (e.g. benthic or pelagic), but there are few filter-feeders. In many regions, shallow lakes provide critical breeding habitat for waterbirds, amphibians, and reptiles, while visiting mammals transfer nutrients, organic matter, and biota.

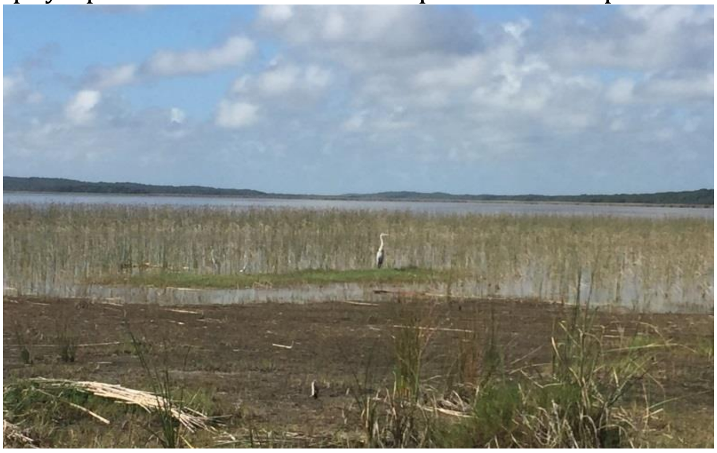

Lake Xinguti, Maputo Special Reserve, Mozambique.
Credit: David Keith

Ecological drivers: These lakes may be hydrologically isolated, groundwater-dependent or connected to rivers as terminal or flow-through systems. Nutrients depend on catchment size and substrates. Some lakes (e.g. on leached coastal sandplains or peaty landscapes) have dystrophic waters. The seasonality and amount of inflow, size, depth (mixing regime and light penetration), pH, nutrients, salinity, and tanins shape lake ecology and biota. Seasonal cycles of temperature, inflow and wind (which drives vertical mixing) may generate monomictic or dimictic temperature stratification regimes in deeper lakes, while shallow lakes are polymicitic, sometimes with short periods of multiple stratification. Seasonal factors such as light, increases in temperature, and flows into lakes can induce breeding and recruitment.

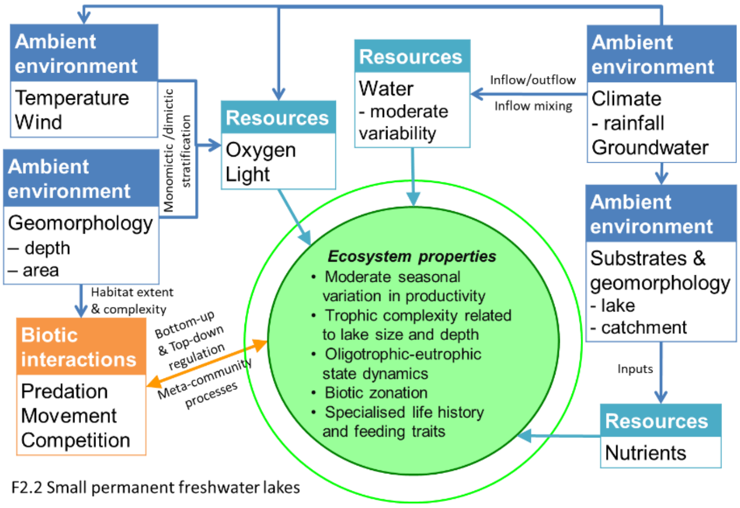

F2.2 Small permanent freshwater lakes

Distribution: Mainly in humid temperate and tropical regions, rarely semi-arid or arid zones.

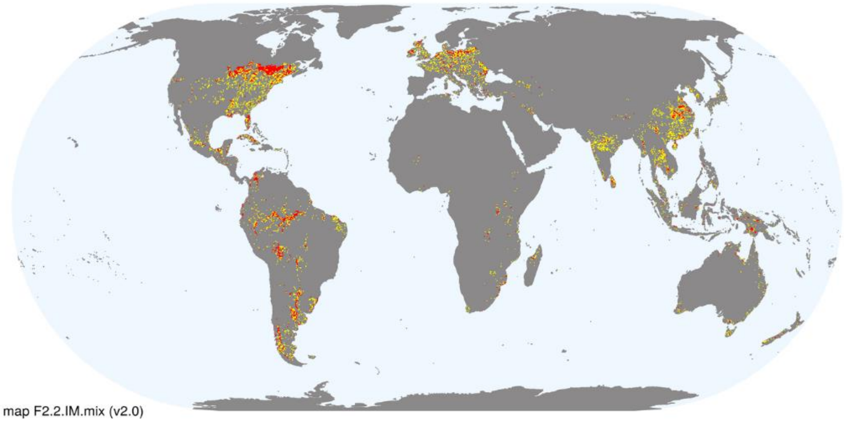

Map is for illustrative purposes only and does not support spatial analyses unless formally validated.

References:

Jeppesen E, Jensen JP, Søndergaard M, Lauridsen T, Pedersen LJ, Jensen L (1997) Top-down control in freshwater lakes: the role of nutrient state, submerged macrophytes and water depth. Hydrobiologia 342–343: 151–164.

Schindler DE, Scheuerell MD (2002) Habitat coupling in lake ecosystems. *Oikos* 98:177-189.

Contributors: RT Kingsford, R Mac Nally, MC Rains, B Robson, K Irvine, DA Keith

F2.3 Seasonal freshwater lakes

Ecosystem properties: These small (mostly <5 km^2 in area) and shallow (<2 m deep) seasonal freshwater lakes, vernal pools, turloughs, or gnammas (panholes, rock pools), have a seasonal aquatic biota. Hydrological isolation promotes biotic insularity and local endemism, which occurs in some Mediterranean climate regions. Autochthonous energy sources are supplemented by limited allochthonous inputs from small catchments and groundwater. Seasonal variation in biota and productivity outweighs inter-annual variation, unlike in ephemeral lakes (F2.5 and F2.7). Filling induces microbial activity, the germination of seeds and algal spores, hatching and emergence of invertebrates, and growth and reproduction by specialists and opportunistic colonists. Wind-induced mixing oxygenates the water, but eutrophic or unmixed waters may become anoxic and dominated by air-breathers as peak productivity and biomass fuel high biological oxygen demand. Anoxia may be abated diurnally by photosynthetic activity. Resident biota persists through seasonal drying on lake margins or in sediments as desiccation-resistant dormant or quiescent life stages, e.g. crayfish may retreat to burrows that extend to the water table, turtles may aestivate in sediments or fringing vegetation, amphibious perennial plants may persist on lake margins or in seedbanks. Trophic networks and niche diversity are driven by bottom-up processes, especially submerged and emergent macrophytes, and depend on productivity and lake size. Cyanobacteria, algae, and macrophytes are the major primary producers, while annual grasses may colonise dry lake beds. The most diverse lakes exhibit zonation and support phytoplankton, zooplankton, macrophytes, macroinvertebrate consumers, and seasonally resident amphibians (especially juvenile aquatic phases), waterbirds, and mammals. Rock pools have simple trophic structure, based primarily on epilithic algae or macrophytes, and invertebrates, but no fish. Invertebrates and amphibians may reach high diversity and abundance in the absence of fish.

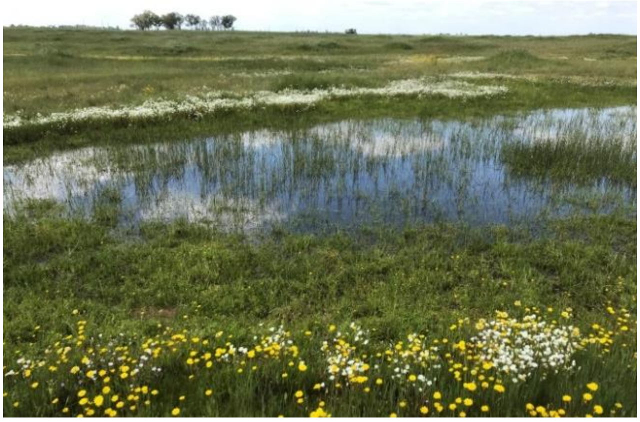

Vernal pool, Mather field, Sacramento Valley, California, USA.
Credit: Jamie Kneitel

Ecological drivers: Seasonal rainfall, surface flows, groundwater fluctuation and seasonally high evapo-transpiration drive annual filling and drying. These lakes are polymicitc, mixing continuously when filled. Impermeable substrates (e.g. clay or bedrock) impede infiltration in some lakes; in others groundwater percolates up through sand, peat or fissures in karstic limestone (turloughs). Small catchments, low-relief terrain, high area-to-volume ratios, and hydrological isolation promote seasonal fluctuation. Most lakes are hydrologically isolated, but some become connected seasonally by sheet flows or drainage lines. These hydrogeomorphic features also limit nutrient supply, in turn limiting pH buffering. Water fluctuations drive high rates of organic decomposition, denitrification, and sediment retention. High alkalinity reflects high anaerobic respiration. Groundwater flows may ameliorate hydrological isolation. Seasonal filling and drying induce spatio-temporal variability in temperature, depth, pH, dissolved oxygen, salinity, and nutrients, resulting in zonation within lakes and high variability among them.

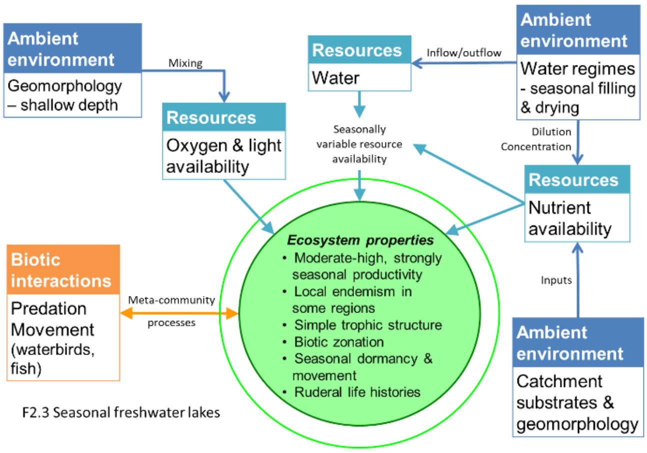

Distribution: Subhumid temperate and wet-dry tropical regions in monsoonal and Mediterranean-type climates but usually not semi-arid or arid regions.

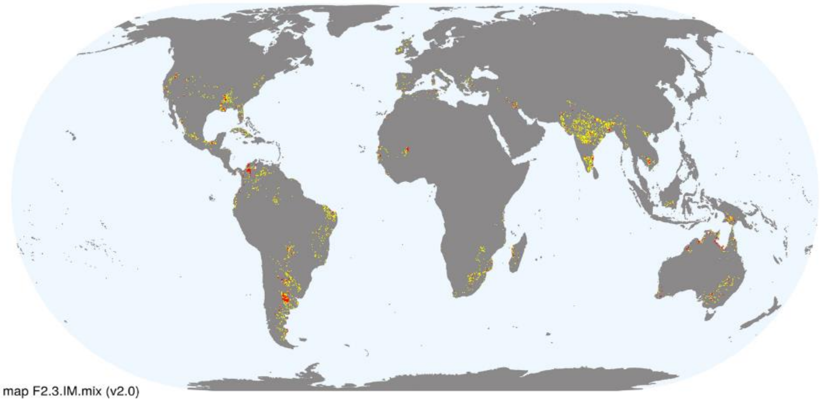

map F2.3.IM.mix (v2.0)

References:

Keeley JE, Zedler PH (1998) Characterization and global distribution of vernal pools. *Ecology, conservation and management of vernal pool ecosystems* (Eds ET Bauder et al.). California Native Plant Society, Sacramento.
Pettit N, Jardine T, Hamilton S, Sinnamon V, Valdez D, Davies P, Douglas M, Bunn S (2012) Seasonal changes in water quality and macrophytes and the impact of cattle on tropical floodplain waterholes. *Marine and Freshwater Research* 63: 788-800.
Rains MC, Fogg GE, Harter T, Dahlgren RA, Williamson RJ (2006) The role of perched aquifers in hydrological connectivity and biogeochemical processes in vernal pool landscapes, Central Valley, California. *Hydrological Processes* 20: 1157-1175.

Map is for illustrative purposes only and does not support spatial analyses unless formally validated.

Contributors: RT Kingsford, S Bertilsson, LJ Jackson, B Robson, DA Keith

F2.4 Freeze-thaw freshwater lakes

Ecosystem properties: The majority of the surface of these lakes is frozen for at least a month in most years. Their varied origins (tectonic, riverine, fluvioglacial), size and depth affect composition and function. Allochthonous and autochthonous energy sources vary with lake and catchment features. Productivity is highly seasonal, sustained in winter largely by the metabolism of microbial photoautotrophs, chemautotrophs and zooplankton that remain active under low light, nutrients, and temperatures. Spring thaw initiates a seasonal succession, increasing productivity and re-establishing complex trophic networks, depending on lake area, depth, connectivity, and nutrient availability. Diatoms are usually first to become photosynthetically active, followed by small and motile zooplankton, which respond to increased food availability, and cyanobacteria later in summer when grazing pressure is high. Large lakes with high habitat complexity (e.g. Lake Baikal) support phytoplankton, zooplankton, macrophytes (in shallow waters), invertebrate consumers, migratory fish (in connected lakes), waterbirds, and mammals. Their upper trophic levels are more abundant, diverse, and endemic than in smaller lakes. Herbivorous fish and zooplankton are significant top-down regulators of the main primary producers (i.e. biofilms and phytoplankton). These, in turn, are regulated by predatory fish, which may be limited by prey availability and competition. The biota is spatially structured by seasonally dynamic gradients in cold stratification, light, nutrient levels, and turbulence. Traits such as resting stages, dormancy, freeze-cued spore production in phytoplankton, and the ability of fish to access low oxygen exchange enable persistence through cold winters under the ice and through seasonal patterns of nutrient availability.

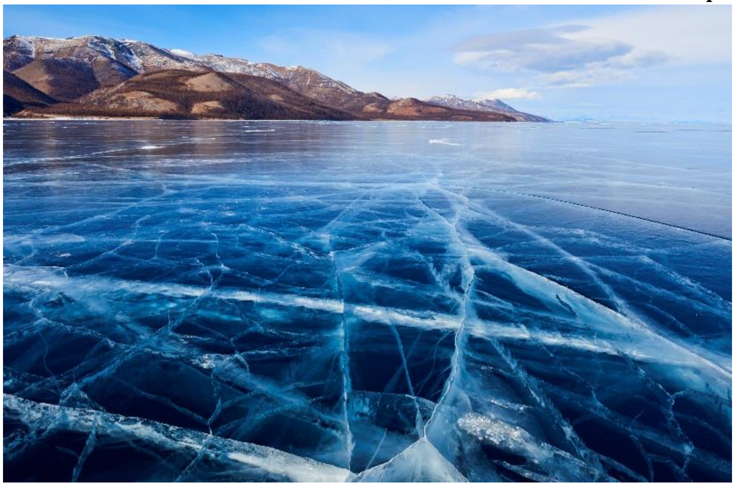

Khovsgol Lake, frozen in winter, Mongolia.
Credit: Tuul & Bruno Morandi / Getty Images

Ecological drivers: Seasonal freeze-thaw cycles typically generate dimictic temperature stratification regimes (i.e. mixing twice per year), where cold water lies above warm water in winter and vice versa in summer. Shallow lakes may mix continuously (polymicitic) during the summer and may freeze completely during winter. Mixing occurs in autumn and spring. Freezing reduces light penetration and turbulence, subduing summer depth gradients in temperature, oxygen, and nutrients. Ice also limits atmospheric inputs, including gas exchange. Very low temperatures reduce the growth rates, diversity, and abundance of fish. Many lakes are stream sources. Lake sizes vary from <1 ha to more than 30,000 km2, profoundly affecting niche diversity and trophic complexity. Freezing varies with the area and depth of lakes. Thawing is often accompanied by flooding in spring, ameliorating light and temperature gradients, and increasing mixing. Dark-water inflows from peatlands in catchments influence water chemistry, light penetration, and productivity.

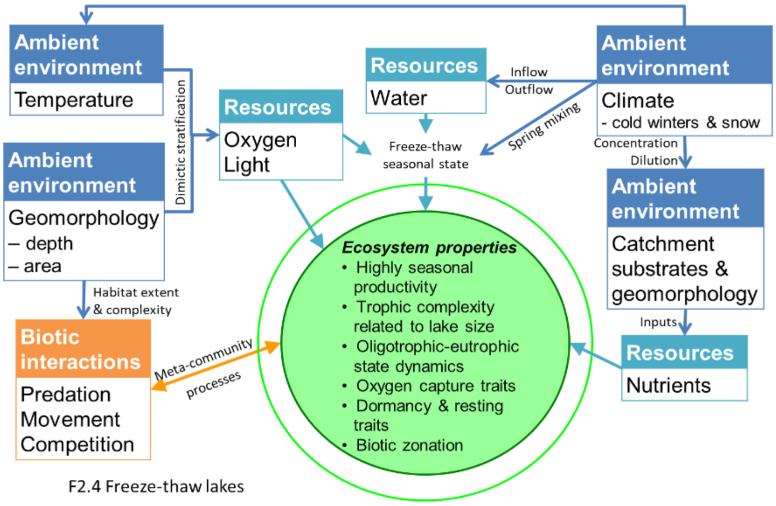

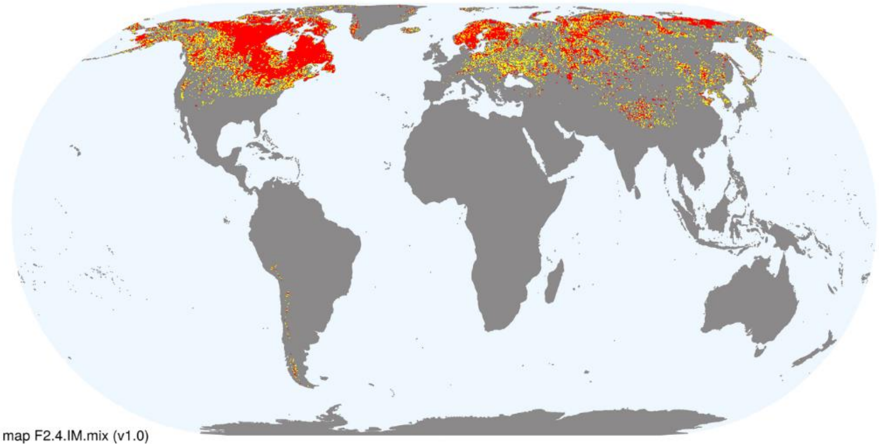

map F2.4.IM.mix (v1.0)

Distribution: Predominantly across the high latitudes of the Northern Hemisphere and high altitudes of South America, New Zealand and Tasmania.

References:

Adrian R, Walz N, Hintze, T, Hoeg S, Rusche R (1999) Effects of ice duration on plankton succession during spring in a shallow polymictic lake. *Freshwater Biology* 41: 621-634.

Bertilsson S, Burgin A, Carey CC, Fey SB, Grossart H, Grubisic LM, Jones ID, Kirillin G, Lennon JT, Shade A, Smyth RL (2013) The under-ice microbiome of seasonally frozen lakes. *Limnology Oceanography* 58: 1998–2012.

Map is for illustrative purposes only and does not support spatial analyses unless formally validated.

F2.5 Ephemeral freshwater lakes

Ecosystem properties: Shallow ephemeral freshwater bodies are also known as depressions, playas, clay pans, or pans. Long periods of low productivity during dry phases are punctuated by episodes of high production after filling. Trophic structure is relatively simple with mostly benthic, filamentous, and planktonic algae, detritivorous and predatory zooplankton (e.g. rotifers and Daphnia), crustaceans, insects, and in some lakes, molluscs. The often high invertebrate biomass provides food for amphibians and itinerant waterbirds. Terrestrial mammals use the lakes to drink and bathe and may transfer nutrients, organic matter, and 'hitch-hiking' biota. Diversity may be high in boom phases but there are only a few local endemics (e.g. narrow-ranged charophytes). Specialised and opportunistic biota exploit boom-bust resource availability through life-cycle traits that confer tolerance to desiccation (e.g. desiccation-resistant eggs in crustaceans) and/or enable rapid hatching, development, breeding, and recruitment when water arrives. Much of the biota (e.g. opportunistic insects) have widely dispersing adult phases enabling rapid colonisation and re-colonisation. Filling events initiate succession with spikes of primary production, allowing short temporal windows for consumers to grow and reproduce, and for itinerant predators to aggregate. Drying initiates senescence, dispersal, and dormancy until the next filling event.

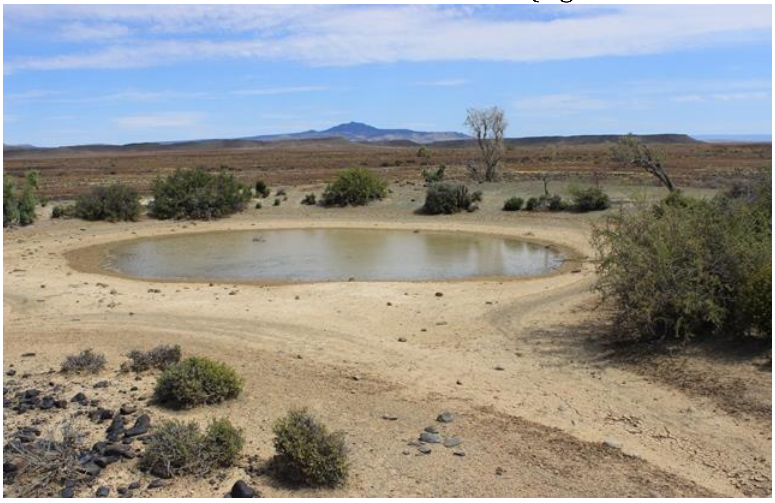

Small, episodic freshwater lake with inflow channel in the arid Tankwa-Karoo National Park, South Africa.
Credit: Dirk Roux

Ecological drivers: Arid climates have highly variable hydrology. Episodic inundation after rain is relatively short (days to months) due to high evaporation rates and infiltration. Drainage systems are closed or nearly so, with channels or sheet inflow from flat, sparsely vegetated catchments. Inflows bring allochthonous organic matter and nutrients and are typically turbid with fine particles. Clay-textured lake bottoms hold water by limiting percolation but may include sand particles. Bottom sediments release nutrients rapidly after filling. Lakes are shallow, flat-bottomed and polymicitic when filled with small volumes, so light and oxygen are generally not limiting. Persistent turbidity may limit light but oxygen production by macrophytes and flocculation (i.e. clumping) from increasing salinity during drying reduce turbidity over time. Shallow depth promotes high daytime water temperatures (when filling in summer) and high diurnal temperature variability.

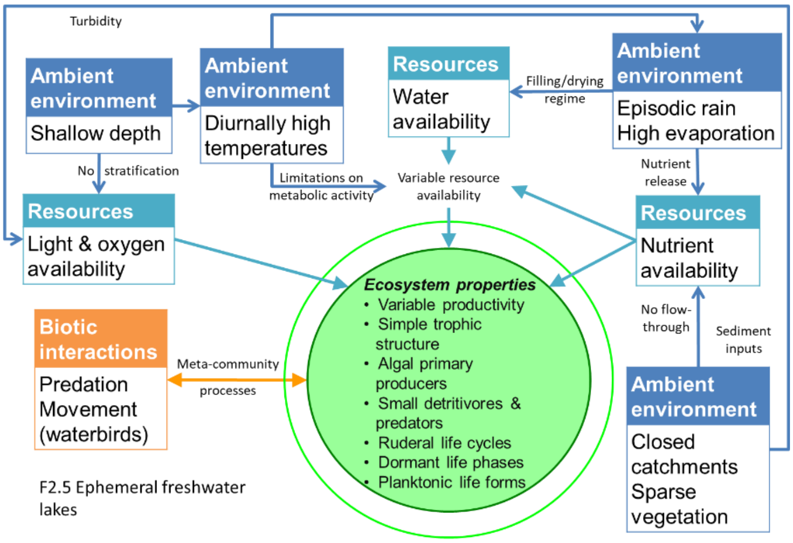

Distribution: Semi-arid and arid regions at mid-latitudes of the Americas, Africa, Asia, and Australia.

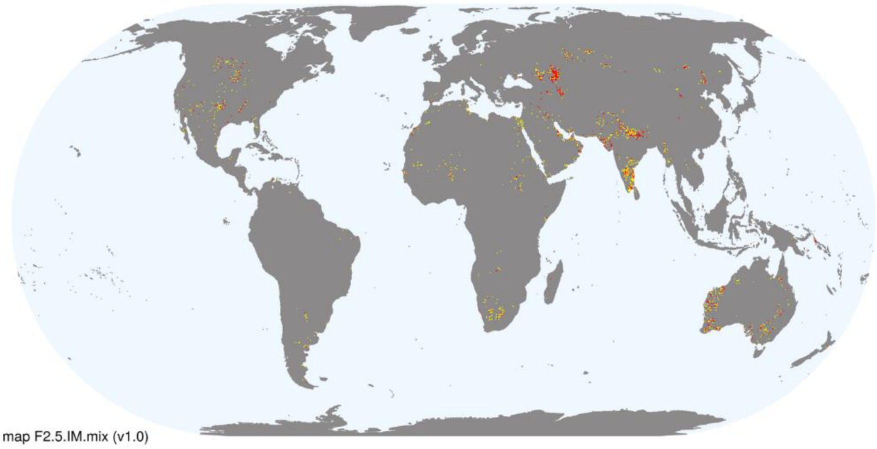

References:

Hancock MA, Timms BV (2002) Ecology of four turbid clay pans during a filling-drying cycle in the Paroo, semi-arid Australia. *Hydrobiologia* 479: 95-107.

Meintjes S (1996) Seasonal changes in the invertebrate community of small shallow ephemeral pans at Bain's Vlei, South Africa. *Hydrobiologia* 317: 51-64.

Williams WD (2000) Biodiversity in temporary wetlands of dryland regions. *Internationale Vereinigung für theoretische und angewandte Limnologie: Verhandlungen* 27: 141-144.

Map is for illustrative purposes only and does not support spatial analyses unless formally validated.

Contributors: RT Kingsford, JT Hollibaugh, B Robson, R Harper, DA Keith

F2.6 Permanent salt and soda lakes

Ecosystem properties: Permanent salt lakes have waters with periodically or permanently high sodium chloride concentrations. This group includes lakes with high concentrations of other ions (e.g. carbonate in soda lakes). Unlike in hypersaline lakes, productivity is not suppressed and autotrophs may be abundant, including phytoplankton, cyanobacteria, green algae, and submerged and emergent macrophytes. These, supplemented by allochthonous energy and C inputs from lake catchments, support relatively simple trophic networks characterised by few species in high abundance and some regional endemism. The high biomass of archaeal and bacterial decomposers and phytoplankton in turn supports abundant consumers including brine shrimps, copepods, insects and other invertebrates, fish, and waterbirds (e.g. flamingos). Predators and herbivores that become dominant at low salinity exert top-down control on algae and low-order consumers. Species niches are structured by spatial and temporal salinity gradients. Species in the most saline conditions tend to have broader ranges of salinity tolerance. Increasing salinity generally reduces diversity and the importance of top-down trophic regulation but not necessarily the abundance of organisms, except at hypersaline levels. Many organisms tolerate high salinity through osmotic regulation (at a high metabolic cost), limiting productivity and competitive ability.

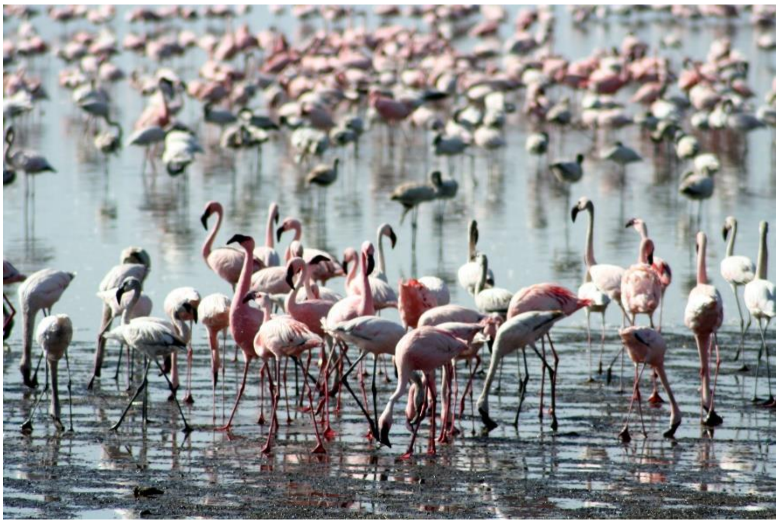

Flamingos on Lake Bogoria, a soda lake in Kenya.
Credit: Richard Kingsford

Ecological drivers: Permanent salt lakes tend to be large and restricted to semi-arid climates with high evaporation but with reliable inflow sources (e.g. snowmelt). They may be thousands of hectares in size and several metres deep. A few are much larger and deeper (e.g. Caspian Sea), while some volcanic lakes are small and deep. Endorheic drainage promotes salt accumulation, but lake volume and reliable water inflows are critical to buffering salinity below extreme levels. Salinity varies temporally from 0.3% to rarely more than 10% depending on lake size, temperature, and the balance between freshwater inflows, precipitation, and evaporation. Inflow is critical to ecosystem dynamics, partly by driving the indirect effects of salinity on trophic or engineering processes. Within lakes, salt concentrations may be vertically stratified (i.e. meromictic) due to the higher density of saltwater compared to freshwater inflow and slow mixing. Dissolved oxygen is inversely related to salinity, hence anoxia is common at depth in meromictic lakes. Ionic composition and concentration varies greatly among lakes due to differences in substrate and inflow, with carbonate, sulphate, sulphide, ammonia, and/or phosphorus sometimes reaching high levels, and pH varying from 3 to 11.

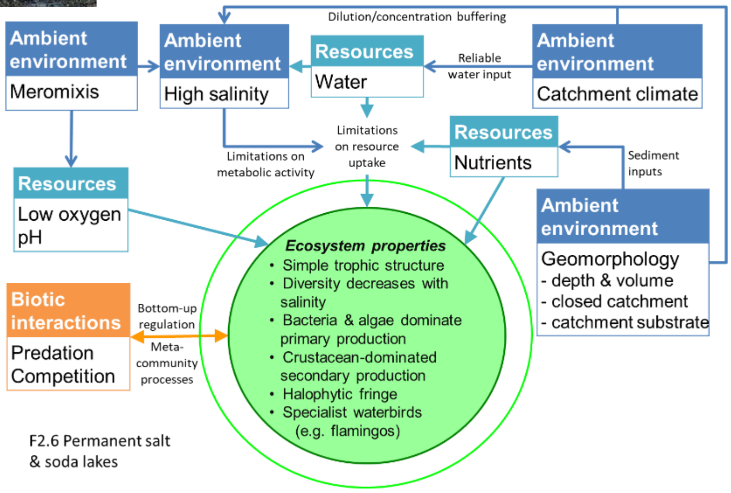

F2.6 Permanent salt & soda lakes

Distribution: Mostly in semi-arid regions of Africa, southern Australia, Eurasia, and western parts of North and South America.

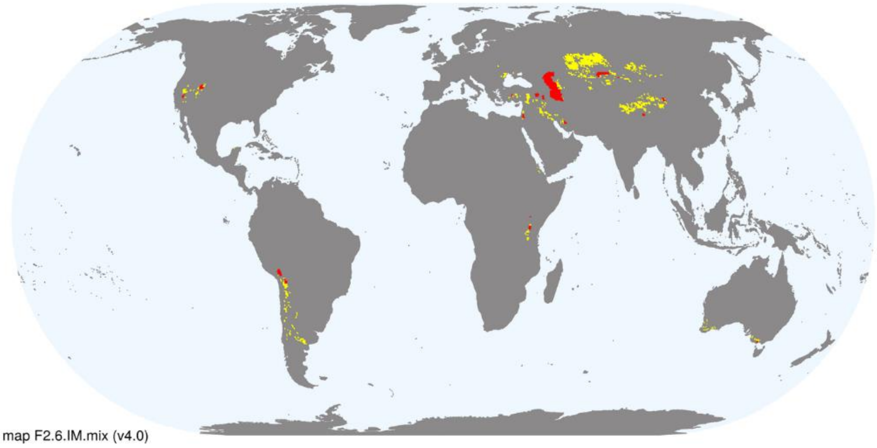

map F2.6.IM.mix (v4.0)

References:

Boros E, Kolpakova M (2018) A review of the defining chemical properties of soda lakes and pans: An assessment on a large geographic scale of Eurasian inland saline surface waters. *PLoSONE* 13(8):e0202205.

Humayoun SB, Bano N, Hollibaugh JT (2003) Depth distribution of microbial diversity in Mono Lake, a meromictic soda lake in California. *Applied and environmental microbiology* 69:1030-1042.

Williams WD (1998) Salinity as a determinant of the structure of biological communities in salt lakes. *Hydrobiologia* 381:191-201.

Map is for illustrative purposes only and does not support spatial analyses unless formally validated.

Contributors: RT Kingsford, JT Hollibaugh, K Irvine, R Harper, DA Keith

F2.7 Ephemeral salt lakes

Ecosystem properties: Ephemeral salt lakes or playas have relatively short-lived wet phases and long dry periods of years to decades. During filling phases, inflow dilutes salinity to moderate levels, and allochthonous energy and carbon inputs from lake catchments supplement autochthonous energy produced by abundant phytoplankton, cyanobacteria, diatoms, green algae, submerged and emergent macrophytes, and fringing halophytes. In drying phases, increasing salinity generally reduces diversity and top-down trophic regulation, but not necessarily the abundance of organisms – except at hypersaline levels, which suppress productivity. Trophic networks are simple and characterised by few species that are often highly abundant during wet phases. The high biomass of archaeal and bacterial decomposers and phytoplankton in turn support abundant consumers, including crustaceans (e.g. brine shrimps and copepods), insects and other invertebrates, fish, and specialist waterbirds (e.g. banded stilts, flamingos). Predators and herbivores that dominate at low salinity levels exert top-down control on algae and low-order consumers. Species niches are strongly structured by spatial and temporal salinity gradients and endorheic drainage promotes regional endemism. Species that persist in the most saline conditions tend to have broad salinity tolerance. Many organisms regulate salinity osmotically at a high metabolic cost, limiting productivity and competitive ability. Many specialised opportunists are able to exploit boom-bust resource cycles through life-cycle traits that promote persistence during dry periods (e.g. desiccation-resistant eggs in crustaceans and/or rapid hatching, development, breeding, and recruitment). Much of the biota (e.g. insects and birds) have widely dispersed adult phases enabling rapid colonisation. Filling events drive specialised succession, with short windows of opportunity to grow and reproduce reset by drying until the next filling event.

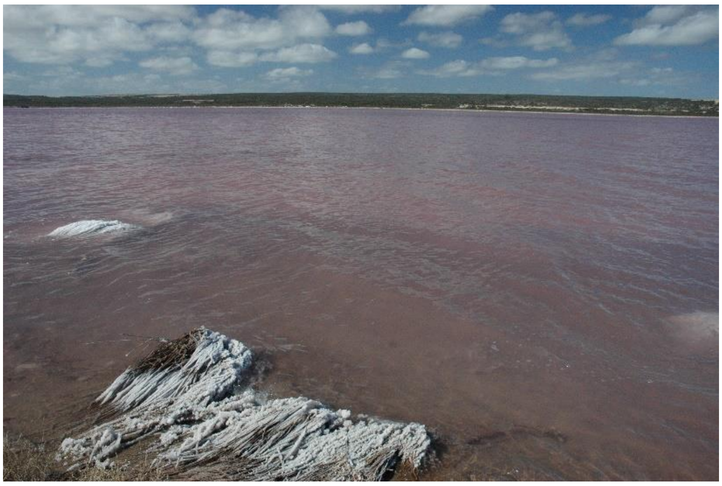

Hutt Lagoon, Port Gregory, Western Australia, pink colour caused by the alga, Dunaliella salina.
Credit: David Keith

Ecological drivers: Ephemeral salt lakes are up to 10,000 km2 in area and usually less than a few metres deep. They may be weakly vertically stratified (i.e. meromictic) due to the slow mixing of freshwater inflow with higher density saltwater. Endorheic drainage promotes salt accumulation. Salinity varies temporally from 0.3% to over 26% depending on lake size, depth temperature, and the balance between freshwater inflows, precipitation, and evaporation. Inflow is critical to ecosystem dynamics, mediates wet-dry phases, and drives the indirect effects of salinity on trophic and ecosystem processes. Dissolved oxygen is inversely related to salinity, hence anoxia is common in hypersaline lake states. Ionic composition varies, with carbonate, sulphate, sulphide, ammonia, and/or phosphorus sometimes at high levels, and pH varying from 3 to 11.

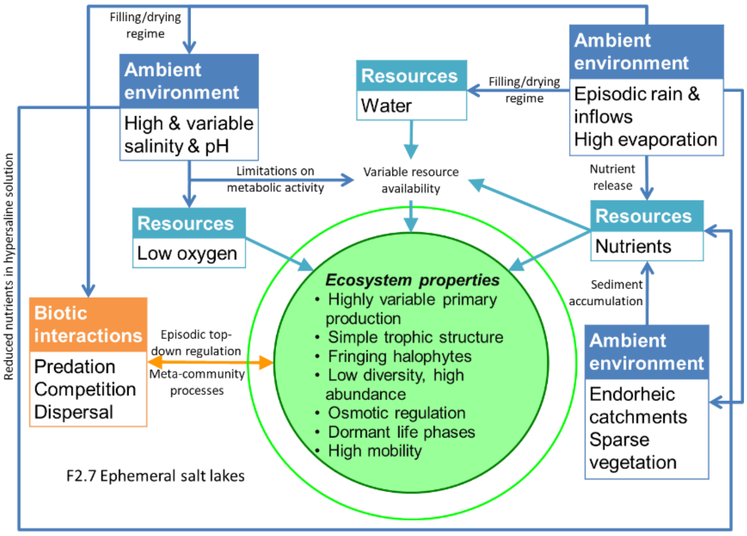

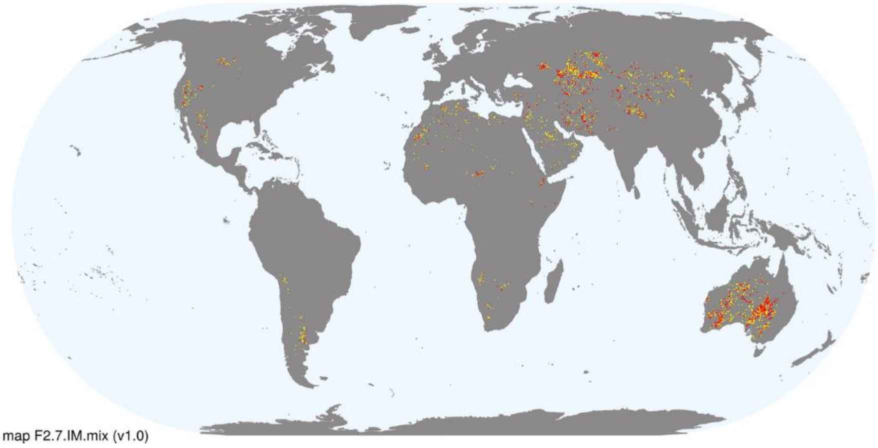

map F2.7.IM.mix (v1.0)

Distribution: Mostly in arid and semi-arid Africa, Eurasia, Australia, and North and South America.

References:

McCulloch G., Aebischer A,Irvine, K (2003) Satellite tracking of flamingos in southern Africa: the importance of small wetlands for management and conservation. *Oryx* 37, 480–483.

Seaman MT, Ashton PJ, Williams WD (1991) Inland salt waters of southern Africa. *Hydrobiologia* 210: 75-91.

Williams WD (1998) Salinity as a determinant of the structure of biological communities in salt lakes. *Hydrobiologia* 381:191-201.

Map is for illustrative purposes only and does not support spatial analyses unless formally validated.

Contributors: DJ Roux, RT Kingsford, AH Arthington, DA Keith

F2.8 Artesian springs and oases

Ecosystem properties: These groundwater-dependent systems are fed by artesian waters that discharge to the surface. They are.surrounded by dry landscapes and receive little surface inflow, being predominantly disconnected from surface-stream networks. Insularity from the broader landscape results in high levels of endemism in sedentary aquatic biota, which are likely descendants of relic species from a wetter past. Springs may be spatially clustered due to their association with geological features such as faults or outcropping aquifers. Even springs in close proximity may have distinct physical and biological differences. Some springs have outflow streams, which may support different assemblages of plants and invertebrates to those in the spring orifice. Artesian springs and oases tend to have simple trophic structures. Autotrophs include aquatic algae and floating vascular plants, with emergent amphibious plants in shallow waters. Terrestrial plants around the perimeter contribute subsidies of organic matter and nutrients through litter fall. Consumers and predators include crustaceans, molluscs, arachnids, insects, and small-bodied fish. Most biota are poorly dispersed and have continuous life cycles and other traits specialised for persistence in hydrologically stable, warm, or hot mineral-rich water. Springs and oases are reliable watering points for wide-ranging birds and mammals, which function as mobile links for resources and promote the dispersal of other biota between isolated wetlands in the dryland matrix.

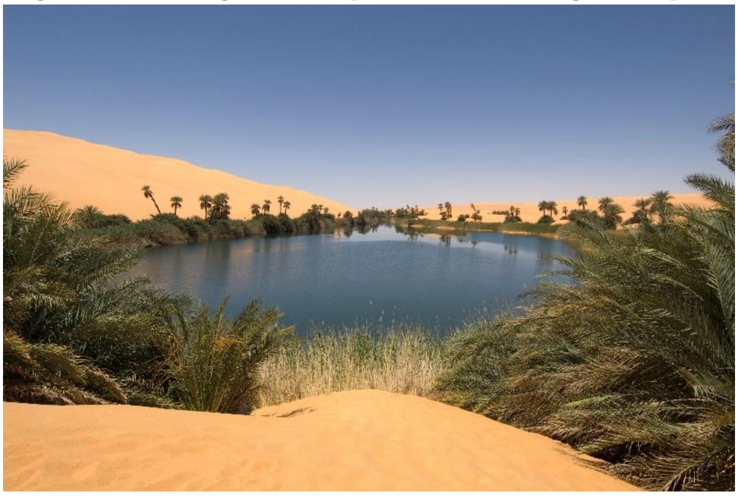

Umm El Ma lake, Erg Awbari, Sahara desert, Libya.
Credit: Delta Images / Getty Images

Ecological drivers: Flow of artesian water to the surface is critical to these wetlands, which receive little input from precipitation or runoff. Hydrological variability is low compared to other wetland types, but hydrological connections with deep regional aquifers, basin-fill sediments and local watershed recharge drive lagged flow dynamics. Flows vary over geological timeframes, with evidence of cyclic growth, waning, and extinction. Discharge waters tend to have elevated temperatures, are polymicitic and enriched in minerals that reflect their geological origins. The precipitation of dissolved minerals (e.g. carbonates) and deposition by wind and water form characteristic cones or mounds known as "mound springs". Perennial flows and hydrological isolation from other spatially and temporally restricted surface waters make these wetlands important ecological refuges in arid landscapes.

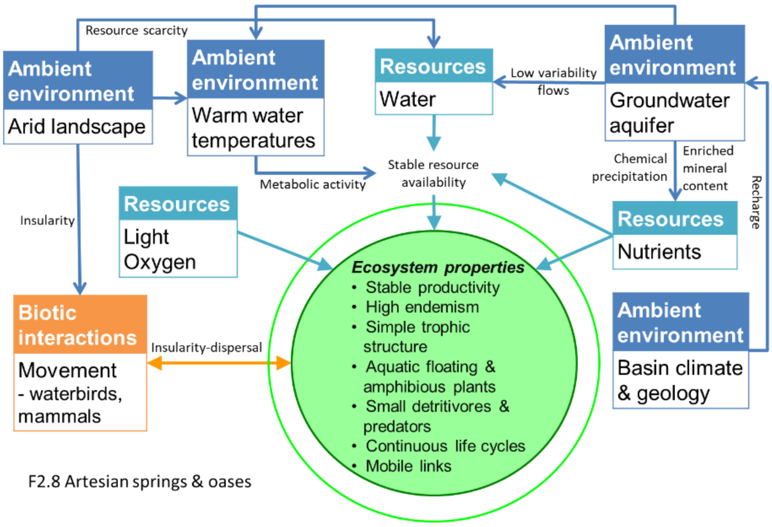

Distribution: Scattered throughout arid regions in southern Africa, the Sahara, the Middle East, central Eurasia, southwest of North America, and Australia's Great Artesian Basin.

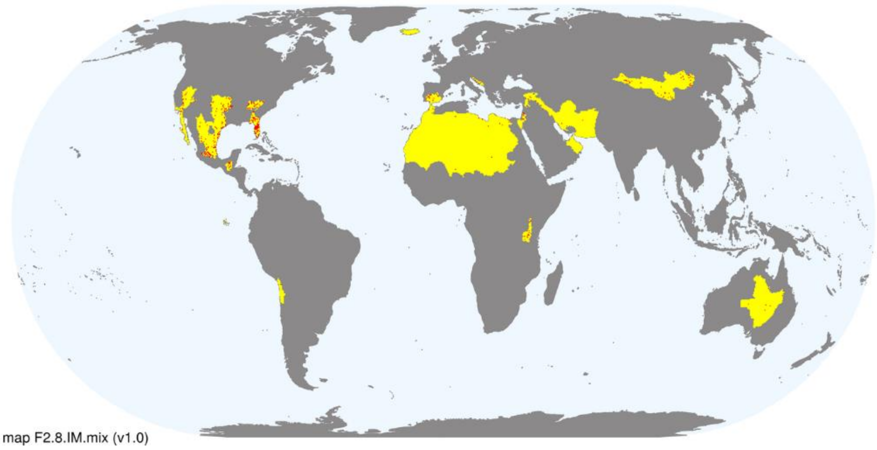

References:

Patten DT, Rouse L, Stromberg JC (2008) Isolated spring wetlands in the Great Basin and Mojave Deserts, USA: potential response of vegetation to groundwater withdrawal. *Environmental Management* 41: 398-413.

Rossini R, Fensham R, Stewart-Koster B, Gotch T, Kennard M (2018) Biographical patterns of endemic diversity and its conservation in Australia's artesian desert springs. *Diversity and Distributions* 24, 1-18.

Worthington Wilmer J, Elkin C, Wilcox C, Murray L, Niejalke D, Possingham H (2008) The influence of multiple dispersal mechanisms and landscape structure on population clustering and connectivity in fragmented artesian spring snail populations. *Molecular Ecology* 17: 3733-3751.

Map is for illustrative purposes only and does not support spatial analyses unless formally validated.

Contributors: DA Keith, A Channing, PS Giller

F2.9 Geothermal pools and wetlands

Ecosystem properties: These hot springs, geysers, mud pots and associated wetlands result from interactions of deeply circulating groundwater with magma and hot rocks that produce chemically precipitated substrates. They support a specialised but low-diversity biota structured by extreme thermal and geochemical gradients. Energy is almost entirely autochthonous, productivity is low, and trophic networks are very simple. Primary producers include chemoautotrophic bacteria and archaea, as well as photoautotrophic cyanobacteria, diatoms, algae, and macrophytes. Thermophilic and metallophilic microbes dominate the most extreme environments in vent pools, while mat-forming green algae and animal-protists occur in warm acidic waters. Thermophilic blue-green algae reach optimum growth above 45°C. Diatoms occur in less acidic warm waters. Aquatic macrophytes occur on sinter aprons and wetlands with temperatures below 35°C. Herbivores are scarce, allowing thick algal mats to develop. These are inhabited by invertebrate detritivores, notably dipterans and coleopterans, which may tolerate temperatures up to 55°C. Molluscs and crustaceans occupy less extreme microhabitats (notably in hard water hot springs), as do vertebrates such as amphibians, fish, snakes and visiting birds. Microinvertebrates such as rotifers and ostracods are common. Invertebrates, snakes and fish exhibit some endemism due to habitat insularity. Specialised physiological traits enabling metabolic function in extreme temperatures include thermophilic proteins with short amino-acid lengths, chaperone molecules that assist protein folding, branched chain fatty acids and polyamines for membrane stabilisation, DNA repair systems, and upregulated glycolysis providing energy to regulate heat stress. Three mechanisms enable metabolic function in extremely acidic (pH<3) geothermal waters: proton efflux via active transport pumps that counter proton influx, decreased permeability of cell membranes to suppress proton entry into the cytoplasm, and strong protein and DNA repair systems. Similar mechanisms enable metabolic function in waters with high concentrations of metal toxins. A succession of animal and plant communities occur with distance from the spring source as temperatures cool and minerals precipitate.

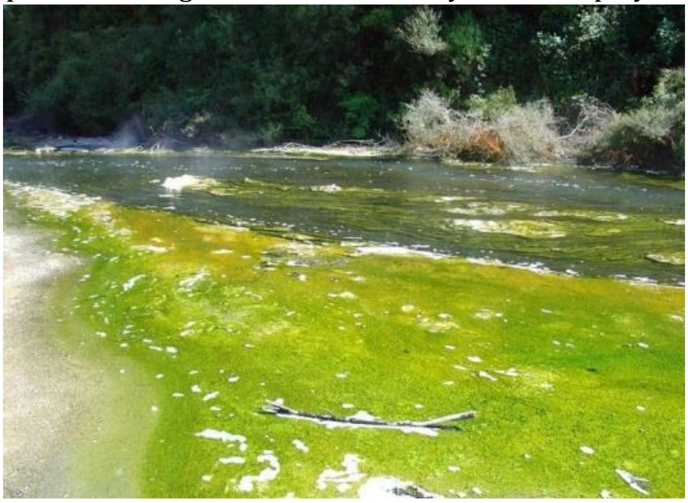

Cyanobacterial growth downstream of Waimangu Hot Springs, Taupo Volcanic Zone, New Zealand.
Credit: Sylvia Hay

Ecological drivers: Continual flows of geothermal groundwater sustain these polymicitic water bodies. Permanent surface waters may be clear or highly turbid with suspended solids as in 'mud volcanoes'. Water temperatures vary from hot (>44°C) to extreme (>80°C) on local gradients (e.g. vent pools, geysers, mounds, sinter aprons, terraces, and outflow streams). The pH is either extremely acid (2–4) or neutral-alkaline (7–11). Mineral salts are concentrated, but composition varies greatly among sites with properties of the underlying bedrock. Dissolved and precipitated minerals include very high concentrations of silicon, calcium or iron, but also arsenic, antimony, copper, zinc, cadmium, lead, polonium or mercury, usually as oxides, sulphides, or sulphates, but nutrients such as nitrogen and phosphorus may be scarce.

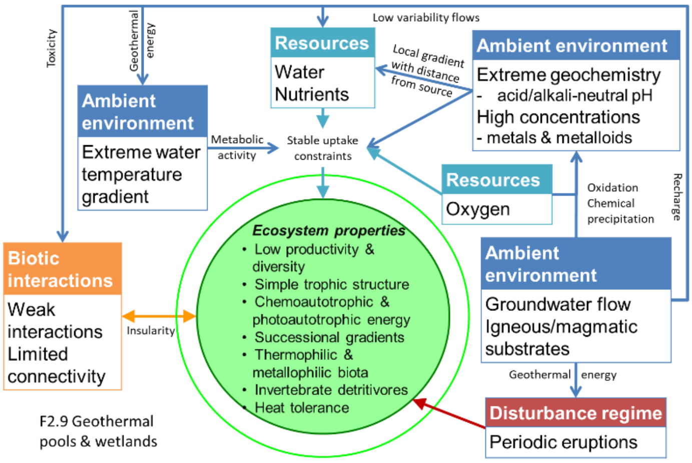

Distribution: Tectonically or volcanically active areas from tropical to subpolar latitudes. Notable examples in Yellowstone (USA), Iceland, New Zealand, Atacama (Chile), Japan and east Africa.

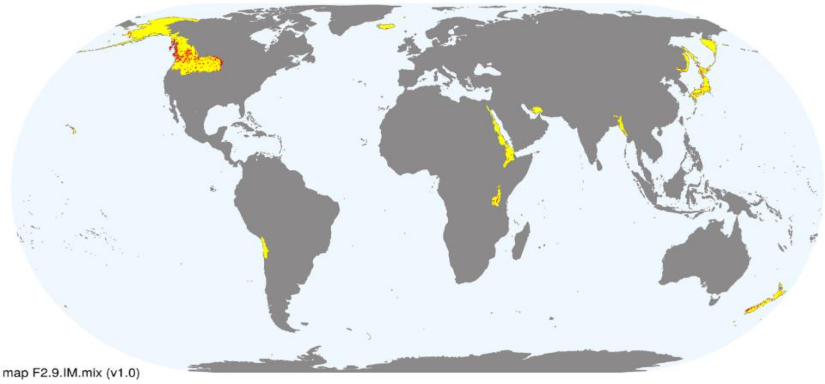

References:

Channing A (2018) A review of active hot-spring analogues of Rhynie: environments, habitats and ecosystems. *Philosophical Transactions of the Royal Society B* 373: 20160490.

Power JF, Carere CR, Lee CK, Wakerley GLJ, Evans DW, Button M, White D, Climo MD, Hinze AM, Morgan XC, McDonald IR, Cary SC, Stott MB (2018) Microbial biogeography of 925 geothermal springs in New Zealand. *Nature Communications* 9: 2876.

Map is for illustrative purposes only and does not support spatial analyses unless formally validated.

F2.10 Subglacial lakes

Ecosystem properties: Remarkable lacustrine ecosystems occur beneath permanent ice sheets. They are placed within the Lakes biome (F2) due to their relationships with some Freeze-thaw lakes (F2.4), but they share several key features with the Subterranean freshwater biome (SF1). Evidence of their existence first emerged in 1973 from airborne radar-echo sounding imagery, which penetrates the ice cover and shows lakes as uniformly flat structures with high basal reflectivity. The biota of these ecosystems is very poorly known due to technological limitations on access and concerns about the risk of contamination from coring. Only a few shallow lakes up to 1 km beneath ice have been surveyed (e.g. Lake Whillams in West Antarctica and Grímsvötn Lake in Iceland). The exclusively microbial trophic web is truncated, with no photoautotrophs and apparently few multi-cellular predators, but taxonomic diversity is high across bacteria and archaea, with some eukaryotes also represented. Chemosynthesis form the base of the trophic web, chemolithoautotrophic species using reduced N, Fe and S and methane in energy-generating metabolic pathways. The abundance of micro-organisms is comparable to that in groundwater (SF1.2) (104 – 105 cells.ml-1), with diverse morphotypes represented including long and short filaments, thin and thick rods, spirals, vibrio, cocci and diplococci. Subglacial lakes share several biotic traits with extremophiles within ice (T6.1), subterranean waters (SF1.1, SF1.2) and deep oceans (e.g. M2.3, M2.4, M3.3), including very low productivity, slow growth rates, large cell sizes and aphotic energy synthesis. Although microbes of the few surveyed subglacial lakes, and from accreted ice which has refrozen from lake water, have DNA profiles similar to those of other contemporary microbes, the biota in deeper disconnected lake waters and associated lake-floor sediments, could be highly relictual if it evolved in stable isolation over millions of years under extreme selection pressures.

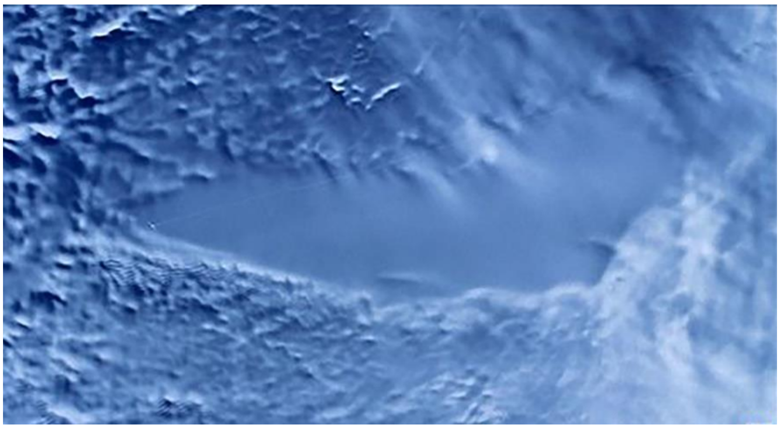

Radar image of Lake Vostok ~4 km below the icesheet surface, East Antarctica.
Credit: Goddard Space Flight Center / NASA, public domain

Ecological drivers: Subglacial lakes vary in size from less than 1 km2 to ~10,000 km2, and most are 10-20 m deep, but Lake Vostok (Antarctica) is at least 1,000 m deep. The environment is characterised by high isostatic pressure (up to ~350 atmospheres), constant cold temperatures marginally below 0°C, low-nutrient levels, and an absence of sunlight. Oxygen concentrations can be high due to equilibration with gas hydrates from the melting ice sheet base ice, but declines with depth in amictic lakes due to limited mixing, depending on convection gradients generated by cold meltwater from the ice ceiling and geothermal heating from below. Chemical weathering of basal debris is the main source of nutrients supplemented by ice melt.

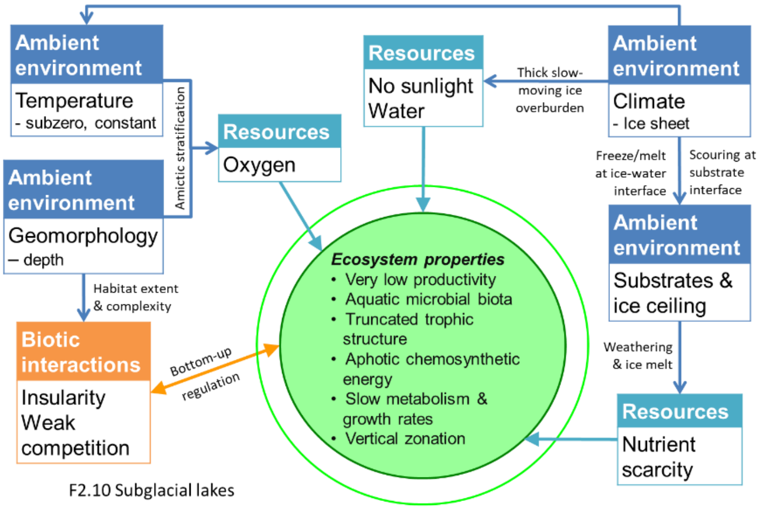

Distribution: Some ~400 subglacial lakes in Antarctica, ~60 in Greenland and a few in Iceland and Canada have been identified from radar remote sensing and modelling.

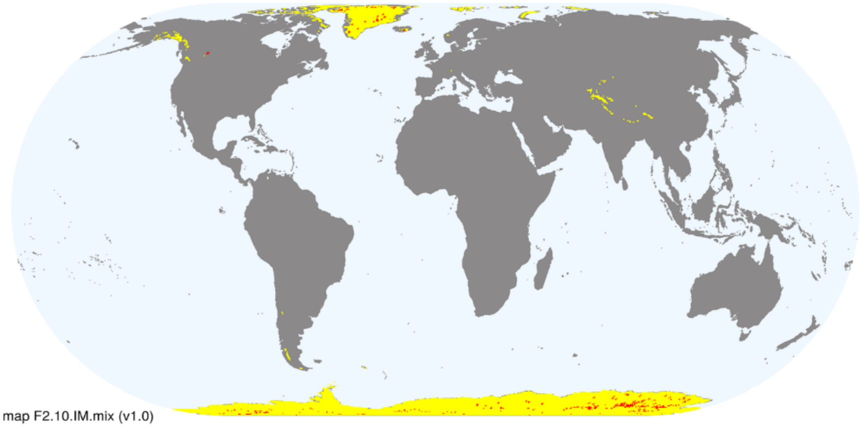

References:

Bowling JS, Livingstone SJ, Sole AJ, Chu W (2019) Distribution and dynamics of Greenland subglacial Lakes. *Nature Communications* 10: 2810.

Mikucki JA et al. (2016) Subglacial Lake Whillans microbial biogeochemistry: a synthesis of current knowledge. *Phil. Trans. R. Soc. A* 374: 20140290.

Siegert MJ, Ellis-Evans JC, Tranter M, Mayer C, Petit JR, Salamatink A, Priscu JC (2001) Physical, chemical and biological processes in Lake Vostok and other Antarctic subglacial lakes. *Nature* 414: 603-609.

Map is for illustrative purposes only and does not support spatial analyses unless formally validated.

F3. Artificial wetlands biome

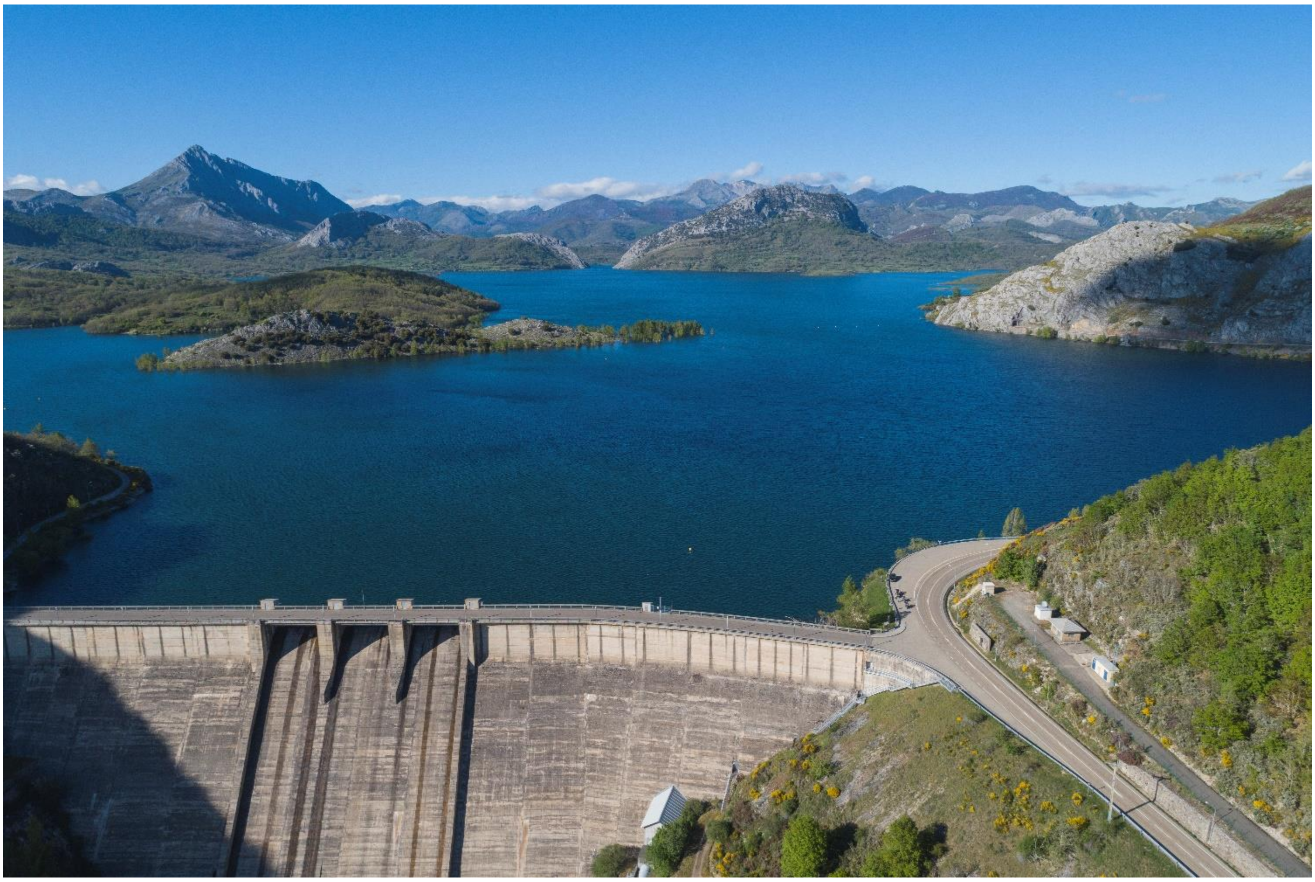

Aerial view of Vegamián reservoir and Porma River Dam, Spain.

Credit: Cavan images / Getty Images

The Artificial wetlands biome includes built structures that hold or transfer water for human use, treatment, or disposal, including large storage reservoirs, farm dams or ponds, recreational and ornamental wetlands, rice paddies, freshwater aquafarms, wastewater storages and treatment ponds, and canals, ditches and drains. These are globally distributed but are most often found in humid and subhumid tropical and temperate environments where rural and urban developments are predominant.

Most of these ecosystems contain standing water with the exception of canals and drains. For most of these ecosystems, energy, water, and nutrients come primarily from allochthonous sources, either incidentally from run-off (e.g. farm dams, ditches and storm water canals) or groundwater, or deterministically by management (e.g. rice paddies, aquafarms, and wastewater ponds), but autochthonous energy sources (in situ algae and macrophytes) can be important in some artificial waterbodies. Water chemistry varies with human use, with some wastewater ponds accumulating toxins or eutrophic levels of nutrients, while large reservoirs with undisturbed catchments may be oligotrophic.

Artificial wetlands are generally less temporally variable, more spatially homogeneous, and often support less biological diversity and trophic complexity of their natural analogues. Nonetheless, in some highly transformed landscapes, they may provide anthropogenic refuges and critical habitat for complementary suites of native biota to that remaining in depleted wetlands, including some biota that no longer occur in natural or semi-natural ecosystems, as well as a range of opportunistic colonists.

Trophic webs vary with the connectivity and depth of the water body, temperature, and substrate. The simplest artificial wetlands support only microbial biota, while the most diverse can include submerged or emergent plant communities, which promote complex habitats for invertebrates, fish, waterbirds, amphibians, reptiles, and, sometimes, amphibious mammals.

F3.1 Large reservoirs

Ecosystem properties: Rivers are impounded by the construction of dam walls, creating large freshwater reservoirs, mostly 15–250 m deep. Primary productivity is low to moderate and restricted to the euphotic zone (limnetic and littoral zones), varying with turbidity and associated light penetration, nutrient availability, and water temperature. Trophic networks are simple with low species diversity and endemism. Shallow littoral zones have the highest species diversity including benthic algae, macroinvertebrates, fish, waterbirds, aquatic reptiles, aquatic macrophytes, and terrestrial or amphibious vertebrates. Phytoplankton and zooplankton occur through the littoral and limnetic zones. The profundal zone lacks primary producers and, if oxygenated, is dominated by benthic detritivores and microbial decomposers. Fish communities inhabit the limnetic and littoral zones and may be dominated by managed species and opportunists. Reservoirs may undergo eutrophic succession due to inflow from catchments with sustained fertiliser application or other nutrient inputs.

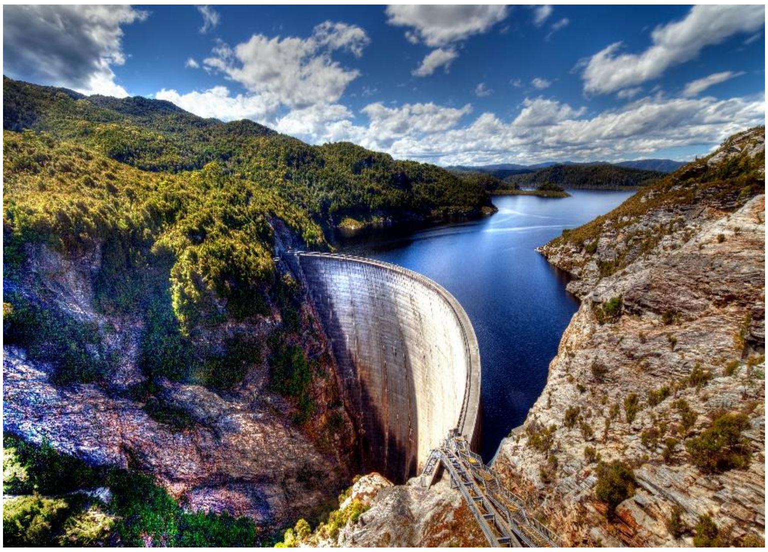

Gordon Dam, Tasmania, Australia.
Credit: Steve Daggar / Getty Images

Ecological drivers: Reservoirs receive water from the rivers they impound. Managed release or diversion of water alters natural variability. Large variations in water level produce wide margins that are intermittently inundated or dry, limiting productivity and the number of species able to persist there. Inflow volumes may be regulated. Inflows may contain high concentrations of phosphorus and/or nitrogen (e.g. from sewerage treatment effluents or fertilised farmland), leading to eutrophication. Reservoirs in upper catchments generally receive less nutrients and cooler water (due to altitude) than those located downstream. Geomorphology, substrate, and land use of the river basin influence the amount of inflowing suspended sediment, and hence turbidity, light penetration, and the productivity of planktonic and benthic algae, as well as rates of sediment build-up on the reservoir floor. Depth gradients in light and oxygen, as well as thermal stratification, strongly influence the structure of biotic communities and trophic interactions, as do human introductions of fish, aquatic plants, and other alien species.

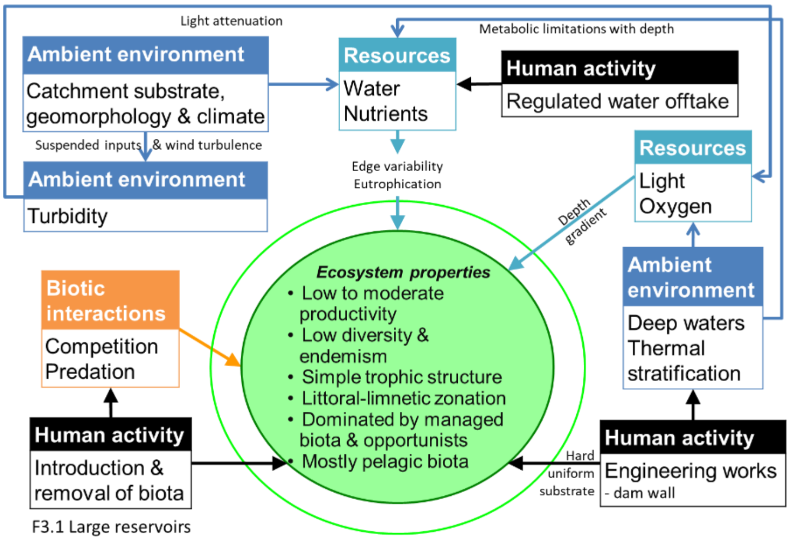

Distribution: Large reservoirs are scattered across all continents with the greatest concentrations in Asia, Europe, and North America. Globally, there are more than 3000 reservoirs with a surface area ≥ 50km². Spatial data are incomplete for some countries.

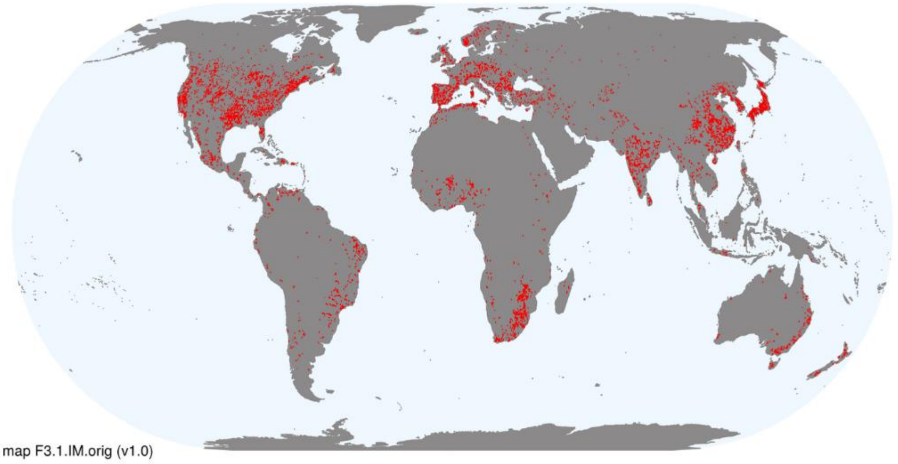

References:

Clavero M, Hermoso, V (2011) Reservoirs promote the taxonomic homogenization of fish communities within river basins. *Biodiversity and Conservation* 20, 41-57.

Lehner B, Liermann CR, Revenga C, Vörösmarty C, Fekete B, Crouzet P, Döll P, Endejan M, Frenken K, Magome J, Nilsson C (2011) High-resolution mapping of the world's reservoirs and dams for sustainable river-flow management. *Frontiers in Ecology and the Environment* 9: 494-502.

Map is for illustrative purposes only and does not support spatial analyses unless formally validated.

Contributors: JL Nel, RT Kingsford, B Robson, M Kelly-Quinn, LJ Jackson, R Harper, DA Keith

F3.2 Constructed lacustrine wetlands

Ecosystem properties: Shallow, open water bodies have been constructed in diverse landscapes and climates. They may be fringed by amphibious vegetation, or else bedrock or bare soil maintained by earthworks or livestock trampling. Emergents rarely extend throughout the water body, but submerged macrophytes are often present. Productivity ranges from very high in wastewater ponds to low in mining and excavation pits, depending on depth, shape, history and management. Taxonomic and functional diversity range from levels comparable to natural lakes to much less, depending on productivity, complexity of aquatic or fringing vegetation, water quality, management and proximity to other waterbodies or vegetation. Trophic structure includes phytoplankton and microbial detritivores, with planktonic and invertebrate predators dominating limnetic zones. Macrophytes may occur in shallow littoral zones or submerged habitats, and some artificial water bodies include higher trophic levels including macroinvertebrates, amphibians, turtles, fish, and waterbirds. Fish may be introduced by people or arrive by flows connected to source populations, where these exist. Endemism is generally low, but these waterbodies may be important refuges for some species now highly depleted in their natural habitats. Life histories often reflect those found in natural waterbodies nearby, but widely dispersed opportunists dominate where water quality is poor. Intermittent water bodies support biota with drought resistance or avoidance traits, while permanently inundated systems provide habitat for mobile species such as waterbirds.

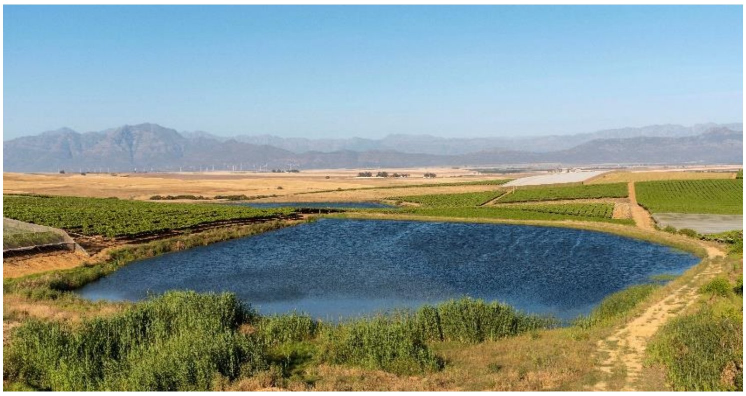

Farm pond, Riebeek-kasteel, Swartland, South Africa.
Credit: Peter Titmuss Universal Images Group / Getty Images

Ecological drivers: Water bodies are constructed for agriculture, mining, stormwater, wastewater, ornamentation, or other uses, or fill depressions left by earthworks, obstructing surface flow or headwater channels. Humans may directly or indirectly regulate inputs of water and chemicals (e.g. fertilisers, flocculants, herbicides), as well as water drawdown. Climate and weather also affect hydrology. Shallow depth and lack of shade may expose open water to rapid solar heating and hence diurnally warm temperatures. Substrates include silt, clay, sand, gravel, cobbles or bedrock, and fine sediments of organic material may build up over time. Nutrient levels are highest in wastewater or with run-off from fertilised agricultural land or urban surfaces. Some water bodies (e.g. mines and industrial wastewaters) have concentrated chemical toxins, extremes of pH or high salinities. Humans may actively introduce and remove the biota of various trophic levels (e.g. bacteria, algae, fish, and macrophytes) for water quality management or human consumption.

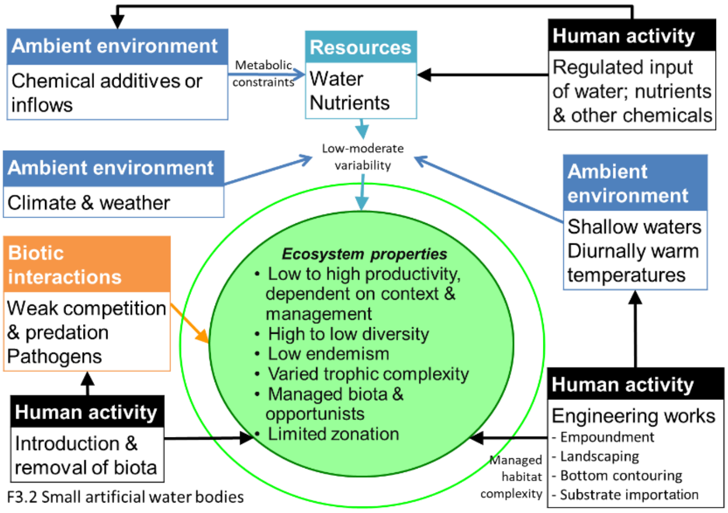

F3.2 Small artificial water bodies

Distribution: Scattered across most regions of the world occupied by humans. Farm dams covered an estimated 77,000km² globally in 2006.

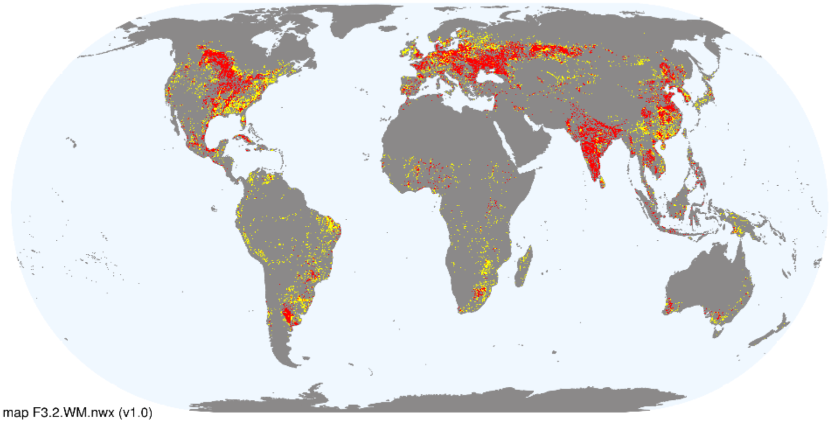

map F3.2.WM.nwx (v1.0)

References:

Chester ET, Robson BJ (2013) Anthropogenic refuges for freshwater biodiversity: Their ecological characteristics and management. *Biological Conservation* 166: 64–75.

Downing JA, Prairie YT, Cole JJ, Duarte CM, Tranvik LJ, Striegl RG, McDowell WH, Kortelainen P, Caraco NF, Melack JM, Middelburg JJ (2006) The global abundance and size distribution of lakes, ponds, and impoundments. *Limnology and Oceanography* 51: 2388-2397.

Kloskowski J, Green AJ, Polak M, Bustamante J, Krogulec J (2009) Complementary use of natural and artificial wetlands by waterbirds wintering in Doñana, south-west Spain. *Aquatic Conservation: Marine and Freshwater Ecosystems* 19: 815-826.

Map is for illustrative purposes only and does not support spatial analyses unless formally validated.

F3.3 Rice paddies

Ecosystem properties: Rice paddies are artificial wetlands with low horizontal and vertical heterogeneity fed by rain or irrigation water diverted from rivers. They are predominantly temporary wetlands, regularly filled and dried, although some are permanently inundated, functioning as simplified marshes. Allochthonous inputs come from water inflow but also include the introduction of rice, other production organisms (e.g. fish and crustaceans), and fertilisers that promote rice growth. Simplified trophic networks are sustained by highly seasonal, deterministic flooding and drying regimes and the agricultural management of harvest crops, weeds, and pests. Cultivated macrophytes dominate primary production, but other autotrophs including archaea, cyanobacteria, phytoplankton, and benthic or epiphytic algae also contribute. During flooded periods, microbial changes produce anoxic soil conditions and emissions by methanogenic archaea. Opportunistic colonists include consumers such as invertebrates, zooplankton, insects, fish, frogs, and waterbirds, as well as other aquatic plants. Often they come from nearby natural wetlands or rivers and may breed within rice paddies. During dry phases, obligate aquatic organisms are confined to wet refugia away from rice paddies. These species possess traits that promote tolerance to low water quality and predator avoidance. Others organisms, including many invertebrates and plants, have rapid life cycles and dormancy traits allowing persistence as eggs or seeds during dry phases.

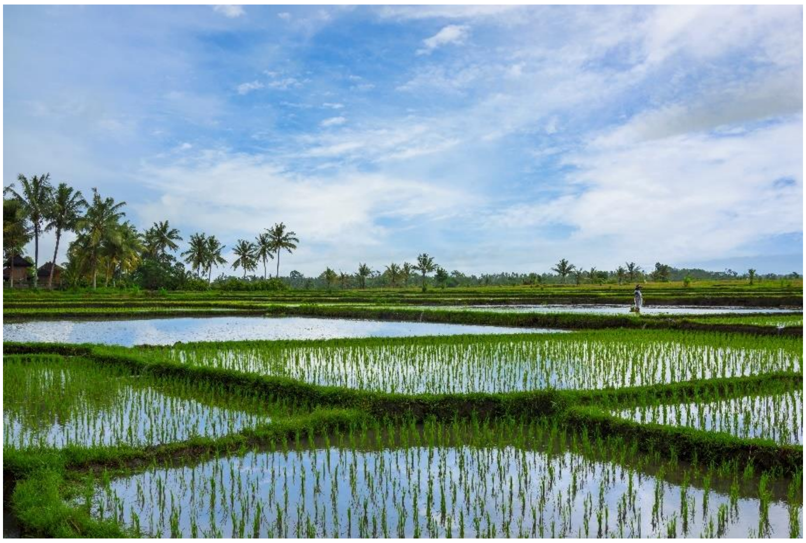

Rice paddies, Bali, Indonesia.
Credit: Darren Robb / Getty Images

Ecological drivers: Engineering of levees and channels enables the retention of standing water a few centimetres above the soil surface and rapid drying at harvest time. This requires reliable water supply either through summer rains in the seasonal tropics or irrigation in warm-temperate or semi-arid climates. The water has high oxygen content and usually warm temperatures. Deterministic water regimes and shallow depths limit niche diversity and have major influences on the physical, chemical, and biological properties of soils, which contain high nutrient levels. Rice paddies are often established on former floodplains but may also be created on terraced hillsides. Other human interventions include cultivation and harvest, aquaculture, and the addition of fertilisers, herbicides, and pesticides.

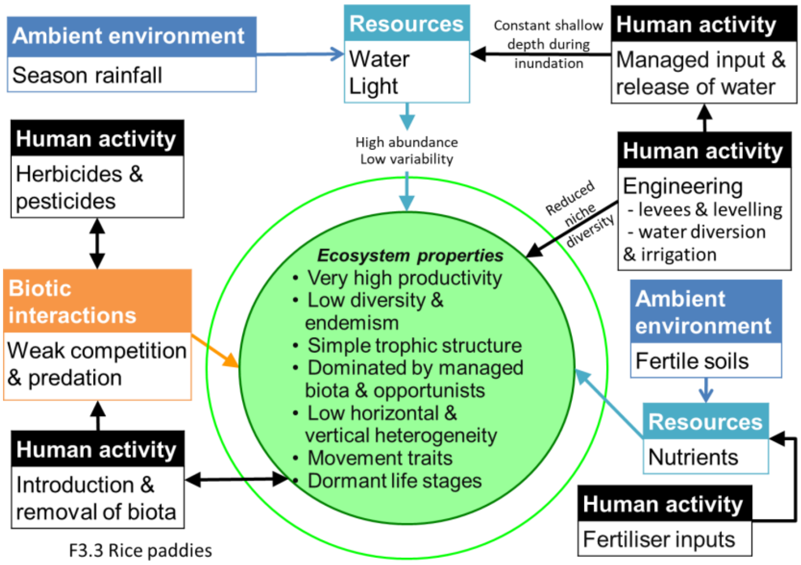

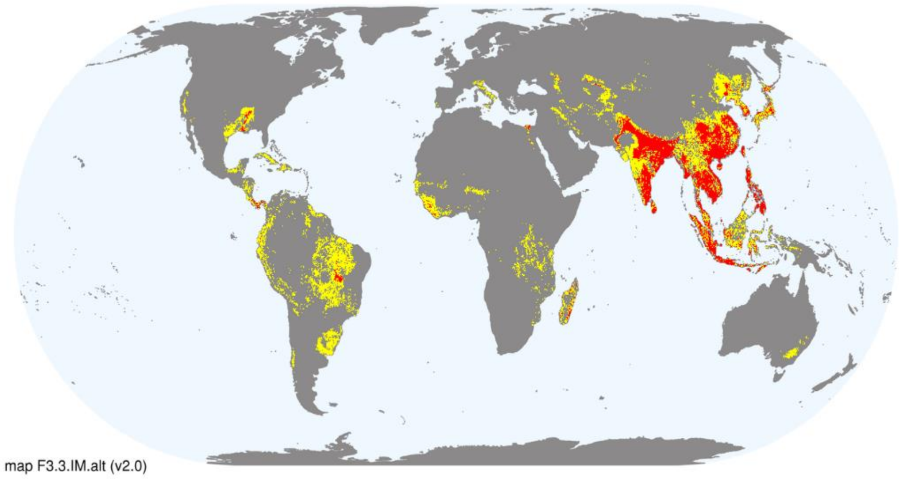

Distribution: More than a million square kilometres, mostly in tropical and subtropical Southeast Asia, with small areas in Africa, Europe, South America, North America, and Australia.

References:

Fernando CJH (1993) Rice field ecology and fish culture—an overview. *Hydrobiologia* 259: 91-113.

Liesack W, Schnell S, Revsbech NP (2000) Microbiology of flooded rice paddies. *FEMS Microbiology Reviews* 24: 625-645.

Map is for illustrative purposes only and does not support spatial analyses unless formally validated.

F3.4 Freshwater aquafarms

Ecosystem properties: Freshwater aquaculture systems are mostly permanent water bodies in either purpose-built ponds, tanks, or enclosed cages within artificial reservoirs (F3.1), canals (F3.5), freshwater lakes (F2.1 and F2.2), or lowland rivers (F1.2). These systems are shaped by large allochthonous inputs of energy and nutrients to promote secondary productivity by one or a few target consumer species (mainly fish or crustaceans), which are harvested as adults and restocked as juveniles on a regular basis. Fish are sometimes raised in mixed production systems within rice paddies (F3.3), but aquaculture ponds may also be co-located with rice paddies, which are centrally located and elevated above the level of the ponds. The enclosed structures exclude predators of the target species, while intensive anthropogenic management of hydrology, oxygenation, toxins, competitors, and pathogens maintains a simplified trophic structure and near-optimal survival and growth conditions for the target species. Intensive management and low niche diversity within the enclosures limit the functional diversity of biota within the system. However, biofilms and phytoplankton contribute low levels of primary production, sustaining zooplankton and other herbivores, while microbial and invertebrate detritivores break down particulate organic matter. Most of these organisms are opportunistic colonists, as are insects, fish, frogs, and waterbirds, as well as aquatic macrophytes. Often these disperse from nearby natural wetlands, rivers, and host waterbodies.

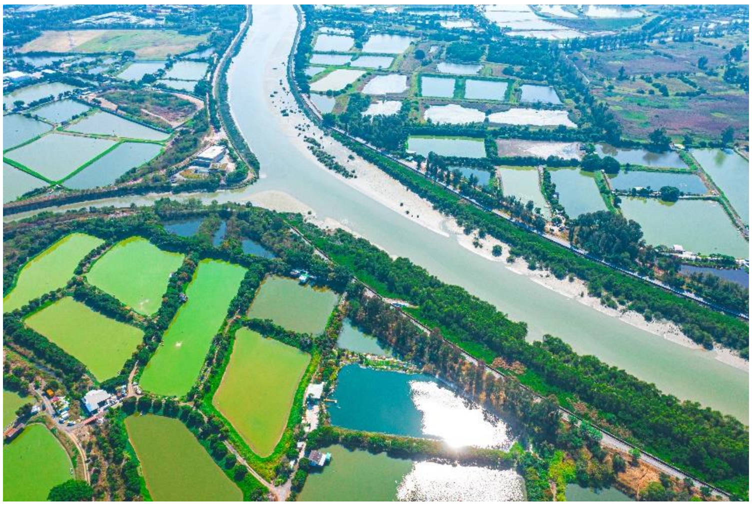

Fish ponds at Tai Sang Wai, Hong Kong, China.
Credit: Chunyip Wong / Getty Images

Ecological drivers: Aquafarms are small artificial water bodies with low horizontal and vertical heterogeneity. Water regimes are mostly perennial but may be seasonal (e.g. when integrated with rice production). Engineering of tanks, channels, and cages enables the intensive management of water, nutrients, oxygen levels, toxins, other aspects of water chemistry, as well as the introduction of target species and the exclusion of pest biota. Removal of wastewater and replacement by freshwater from lakes or streams, together with inputs of antibiotics and chemicals (e.g. pesticides and fertilisers) influence the physical, chemical, and biological properties of the water column and substrate. When located within cages in natural water bodies, freshwater aquafarms reflect the hydrological and hydrochemical properties of their host waterbody. Nutrient inputs drive the accumulation of ammonium and nitrite nitrogen, as well as phosphorus and declining oxygen levels, which may lead to eutrophication within aquaculture sites and receiving waters.

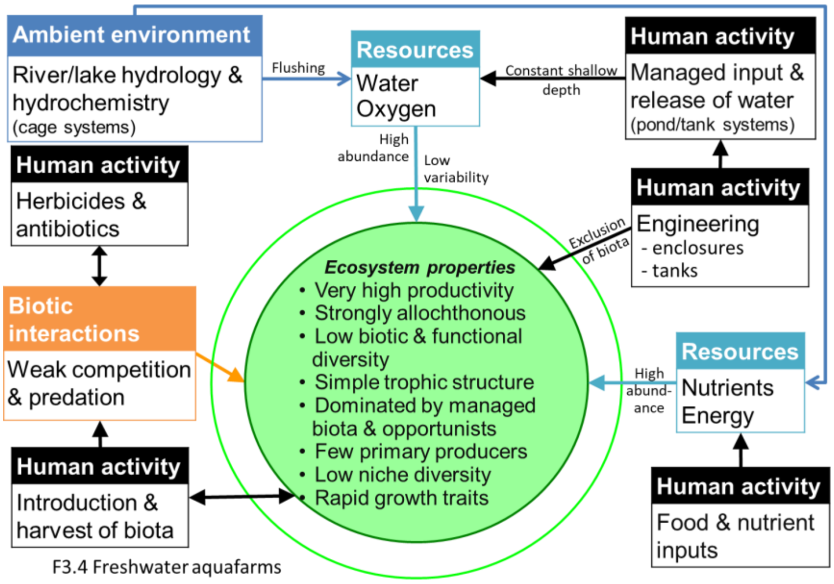

F3.4 Freshwater aquafarms

Distribution: Concentrated in Asia but also in parts of northern and western Europe, North and West Africa, South America, North America, and small areas of southeast Australia and New Zealand.

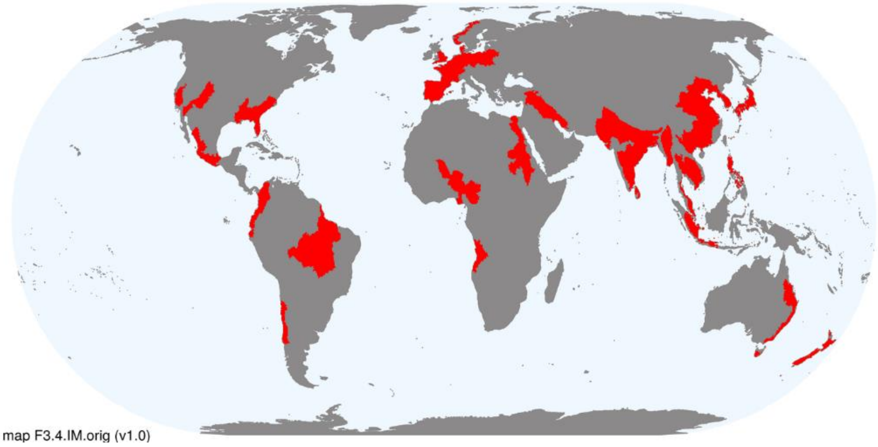
map F3.4.IM.orig (v1.0)

References:

Ottinger M, Clauss K, Kuenzer CJO (2016) Aquaculture: relevance, distribution, impacts and spatial assessments–a review. *Ocean & Coastal Management* 119: 244-266.

Map is for illustrative purposes only and does not support spatial analyses unless formally validated.

Contributors: RT Kingsford, B Robson, M Kelly-Quinn, DA Keith

F3.5 Canals, ditches and drains

Ecosystem properties: Canals, ditches and storm water drains are artificial streams with low horizontal and vertical heterogeneity. They function as rivers or streams and may have simplified habitat structure and trophic networks, though some older ditches have fringing vegetation, which contributes to structural complexity. The main primary producers are filamentous algae and macrophytes that thrive on allochthonous subsidies of nutrients. Subsidies of organic carbon from urban or rural landscapes support microbial decomposers and mostly small invertebrate detritivores. While earthen banks and linings may support macrophytes and a rich associated fauna, sealed or otherwise uniform substrates limit the diversity and abundance of benthic biota. Fish and crustacean communities, when present, generally exhibit lower diversity and smaller body sizes compared to natural systems, and are often dominated by introduced or invasive species. Waterbirds, when present, typically include a low diversity and density of herbivorous and piscivorous species. Canals, ditches and drains may provide pathways for dispersal or colonisation of native and invasive biota.

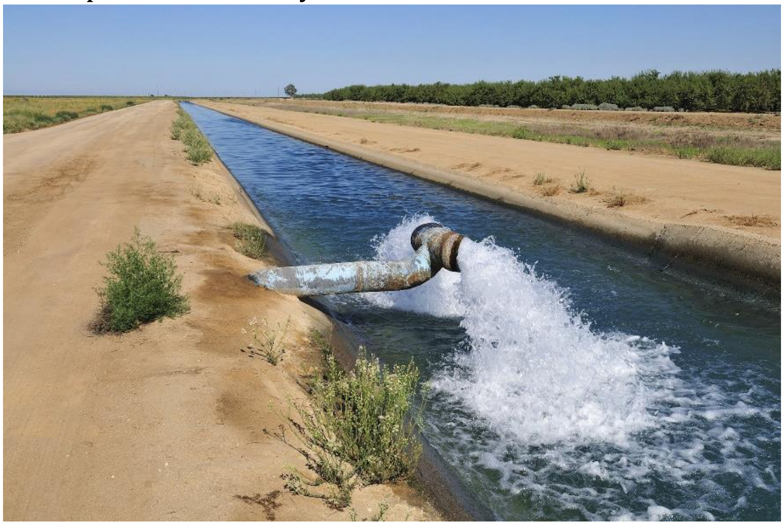

California irrigation canal, USA.
Credit: Richard Thornton / Shutterstock

Ecological drivers: Engineered levees and channels enable managed water flow for human uses, including water delivery for irrigation or recreation, water removal from poorly drained sites or sealed surfaces (e.g. storm water drains), or routes for navigation. Deterministic water regimes and often shallow depths have major influences on the physical, chemical, and biological properties of the canals, ditches and drains. Flows in some ditches may be very slow, approaching lentic regimes. Flows in storm water drains vary with rain or other inputs. Irrigation, transport, or recreation canals usually have steady perennial flows but may be seasonal for irrigation or intermittent where the water source is small. Turbidity varies but oxygen content is usually high. Substrates and banks vary from earthen material or hard surfaces (e.g. concrete, bricks), affecting suitability for macrophytes and niche diversity. The water may carry high levels of nutrients and pollutants due to inflow and sedimentation from sealed surfaces, sewerage, other waste sources, fertilised cropping, or pasture lands.

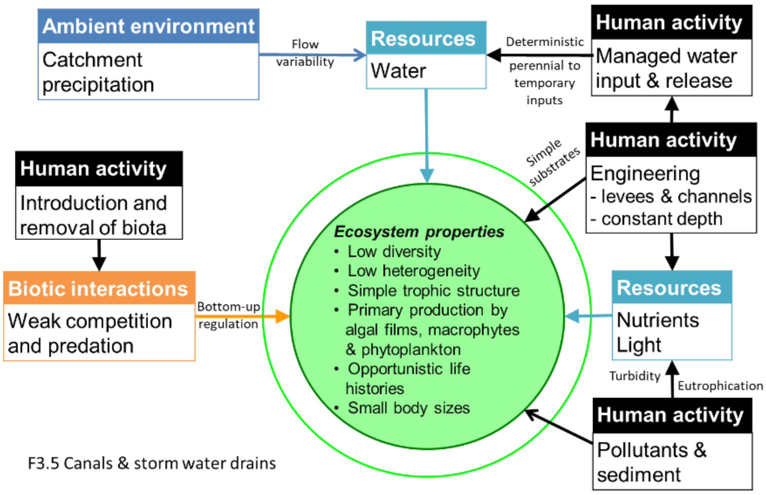

Distribution: Urban landscapes and irrigation areas mostly in temperate and subtropical latitudes. Several hundred thousand kilometres of ditches and canals in Europe.

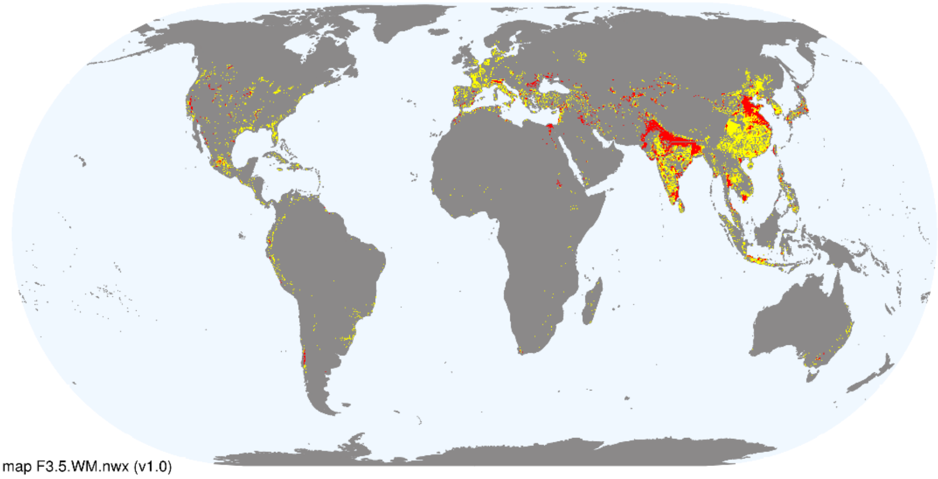

map F3.5.WM.nwx (v1.0)

References:

Chester ET, Robson BJ (2013) Anthropogenic refuges for freshwater biodiversity: Their ecological characteristics and management. *Biological Conservation* 166: 64 – 75.

Nunes AL, Tricarico E, Panov VE, Cardoso AC, Katsanevakis S (2015) Pathways and gateways of freshwater invasions in Europe. *Aquatic Invasions* 10: 359 - 370.

Ricciardi A (2006) Patterns of invasion in the Laurentian Great Lakes in relation to changes in vector activity. *Diversity and Distributions* 12: 425-433.

Verdonschot RCM, Keizer-vlek HE, Verdonschot PFM (2011) Biodiversity value of agricultural drainage ditches: a comparative analysis of the aquatic invertebrate fauna of ditches and small lakes. *Aquatic Conservation: Marine and Freshwater Ecosystems* 21, 715-727.

Map is for illustrative purposes only and does not support spatial analyses unless formally validated.

FM1. Semi-confined transitional waters biome

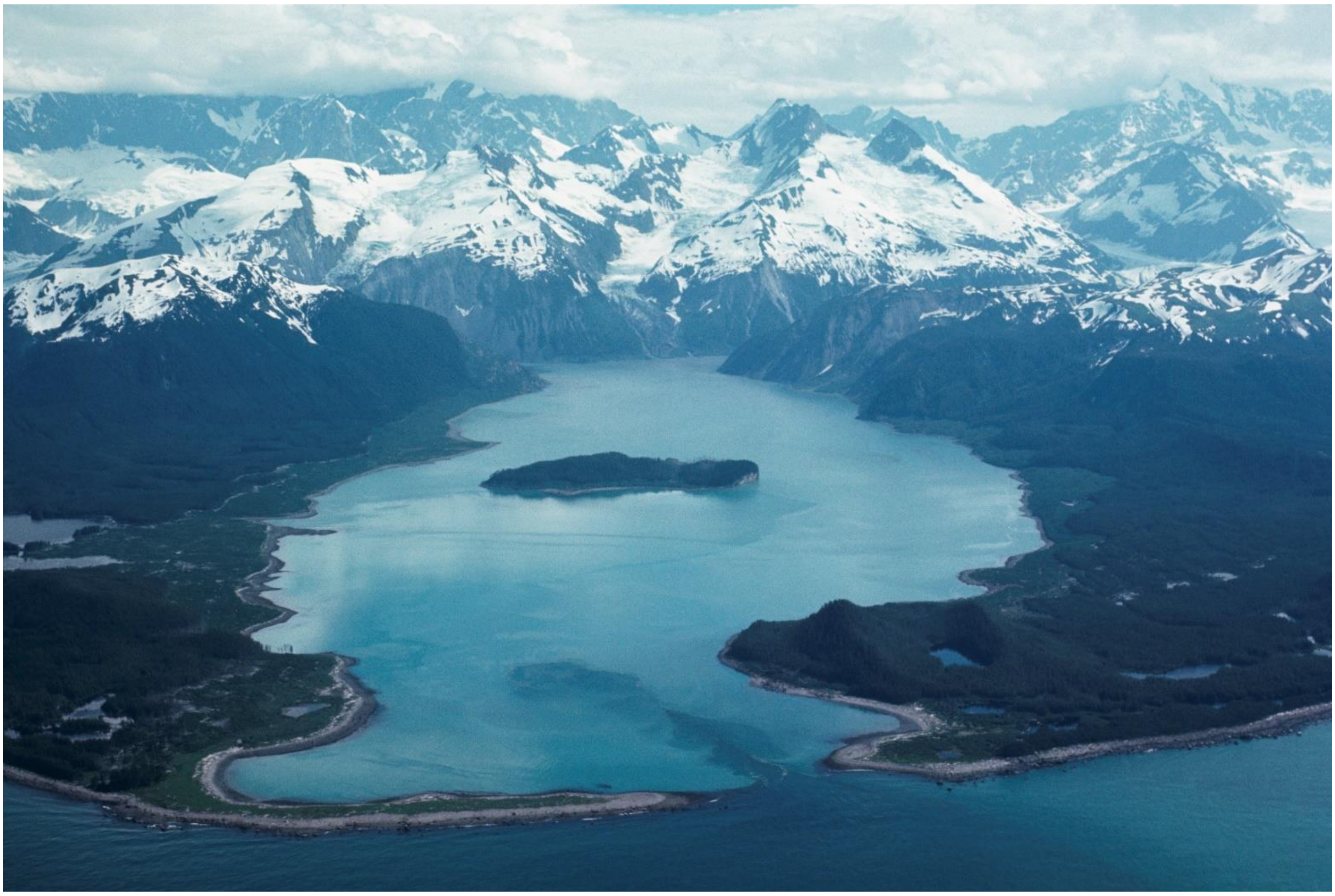

Lituya Bay, Alaska USA.

Credit: Lloyd Cluff / Getty Images

The Transitional waters biome includes coastal inlets that are influenced by inputs of both fresh and marine water from terrestrial catchments and ocean tides, waves, and currents. They include deep-water coastal inlets or fjords mostly restricted to high latitudes, as well as estuaries, bays, and lagoons, which are scattered around coastlines throughout the world.

Gradients in water regimes, water chemistry, depth, temperature, size, and salinity influence the function, productivity, diversity, and trophic structure of these transitional ecosystems. The balance between marine or freshwater influences varies seasonally and inter-annually depending on the climate and among inlets with differing geomorphology, catchment size, climate, and exposure to waves and currents. In some cases, ecosystems characteristic of the marine shelf biome (e.g. M1.1 Seagrass meadows) may have significant occurrences within semi-confined transitional waters. Some inlets are permanently connected to the ocean but others are only intermittently connected, influencing exchanges of water, nutrients, and biota among ecosystems. The dynamics of connection and closure of shallow inlets are regulated by variations in stream flow inputs and wave activity.

Strong horizontal and vertical salinity gradients (varying with freshwater and marine inputs) structure biotic communities and traits that equip species for occupying different salinity niches. Autochthonous energy generated by primary production from aquatic macrophytes, phytoplankton, macroalgae, and diatoms is subsidised by allochthonous inputs from inlet shorelines, freshwater streams, and marine incursion. These high levels of energy availability support complex trophic networks, including large populations of macroinvertebrates, fish, waterbirds, seabirds, and some mammals and reptiles. Many inlets function as fish nurseries and bird breeding sites.

FM1.1 Deepwater coastal inlets

Ecosystem properties: Deepwater coastal inlets (e.g. fjords, sea lochs) are semi-confined aquatic systems with many features of open oceans. Strong influences from adjacent freshwater and terrestrial systems produce striking environmental and biotic gradients. Autochthonous energy sources are dominant, but allochthonous sources (e.g. glacial ice discharge, freshwater streams, and seasonal permafrost meltwater) may contribute 10% or more of particulate organic matter. Phytoplankton, notably diatoms, contribute most of the primary production, along with biofilms and macroalgae in the epibenthic layer. Seasonal variation in inflow, temperatures, ice cover, and insolation drives pulses of *in situ* and imported productivity that generate blooms in diatoms, consumed in turn by jellyfish, micronekton, a hierarchy of fish predators, and marine mammals. Fish are limited by food, density-dependent predation, and cannibalism. As well as driving pelagic trophic networks, seasonal pulses of diatoms shape biogeochemical cycles and the distribution and biomass of benthic biota when they senesce and sink to the bottom, escaping herbivores, which are limited by predators. The vertical flux of diatoms, macrophytes, and terrestrial detritus sustains a diversity and abundance of benthic filter-feeders (e.g. maldanids and oweniids). Environmental and biotic heterogeneity underpins functional and compositional contrasts between inlets and strong gradients within them. Distributions of zooplankton, fish, and jellies reflect resource heterogeneity, environmental cues, and interactions with other organisms. Deep inlets sequester more organic carbon into sediments than other estuaries (FM1.2, FM1.3) because steep slopes enable efficient influx of terrestrial carbon and low-oxygen bottom waters abate decay rates. Inlets with warmer water have more complex trophic webs, stronger pelagic-benthic coupling, and utilise a greater fraction of organic carbon, sequestering it in sea-floor sediments at a slower rate than those with cold water.

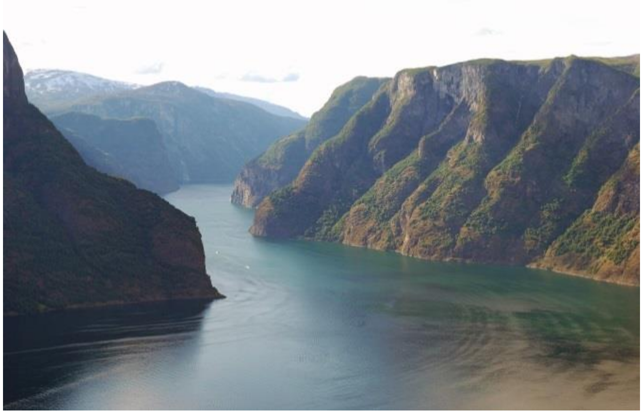

Sognefjord, Norway.
Credit: Arild Lindgaard

Ecological drivers: Deepwater coastal systems may exceed 300 km in length and 2 km in depth. Almost all have glacial origins and many are fed by active glaciers. The ocean interface at the mouth of the inlet, strongly influenced by regional currents, interacts with large seasonal freshwater inflow to the inner inlet and wind-driven advection, producing strong and dynamic spatial gradients in nutrients, salinity and organic carbon. Advection is critical to productivity and carrying capacity of the system. Advection drives water movement in the upper and lower layers of the water column in different directions, linking inlet waters with coastal water masses. Coastal currents also mediate upwelling and downwelling depending on the direction of flow. However, submerged glacial moraines or sills at the inlet mouth may limit marine mixing, which can be limited to seasonal episodes in spring and autumn. Depth gradients in light typically extend beyond the photic zone and are exacerbated at high latitudes by seasonal variation in insolation and surface ice. Vertical fluxes also create strong depth gradients in nutrients, oxygen, dissolved organic carbon, salinity, and temperature.

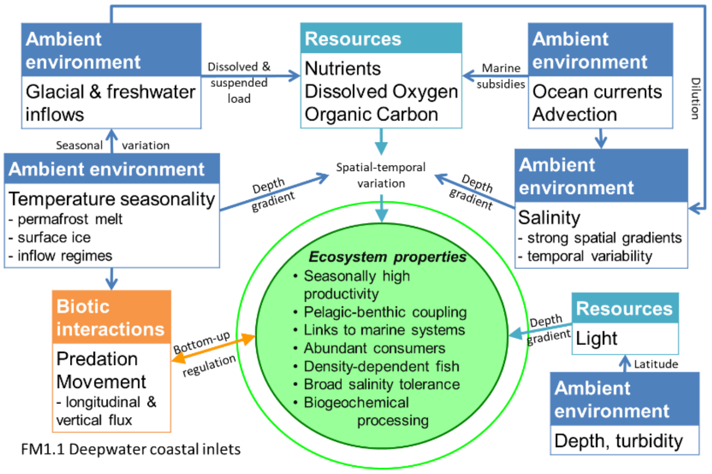

Distribution: Historically or currently glaciated coastlines at polar and cool-temperate latitudes.

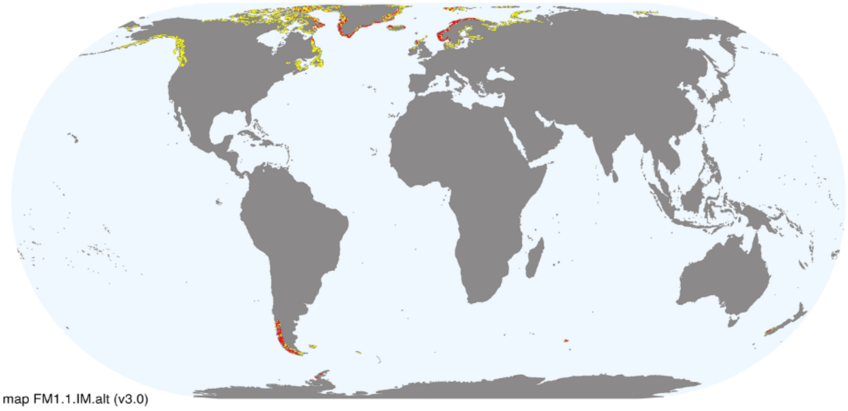

References:

Bianchi TS, Arndt S, Austin WEN, Benn DI, Bertrand S, Cui X, Faust JC, Koziorowska-Makuch K, Moy CM, Savage C, Smeaton C, Smith RW, Syvitski J (2020) Fjords as Aquatic Critical Zones (ACZs). Earth-Science Reviews 203, 103145.

Salvanes AGV (2001) Review of ecosystem models of fjords; new insights of relevance to fisheries management. *Sarsia* 86:441-463.

Zaborska A, Włodarska-Kowalczuk M, Legeżyńska J et al. (2018) Sedimentary organic matter sources, benthic consumption and burial in west Spitsbergen fjords – Signs of maturing of Arctic fjordic systems? Journal of Marine Systems 180: 112–123.

Map is for illustrative purposes only and does not support spatial analyses unless formally validated.

FM1.2 Permanently open riverine estuaries and bays

Ecosystem properties: These coastal water bodies are mosaic systems characterised by high spatial and temporal variabilities in structure and function, which depend on coastal geomorphology, ratios of freshwater inflows to marine waters and tidal volume (hence residence time of saline water), and seasonality of climate. Fringing shoreline systems may include intertidal mangroves (MFT1.2), saltmarshes and reedbeds (MFT1.3), rocky (MT1.1), muddy (MT1.2) or sandy shores (MT1.3), while seagrasses and macrophytes (M1.1), shellfish beds (M1.4) or subtidal rocky reefs (M1.6) may occur in shallow intertidal and subtidal areas. Water-column productivity is typically higher than in nearby marine or freshwater systems due to substantial allochthonous energy and nutrient subsidies from shoreline vegetation and riverine and marine sources. This high productivity supports a complex trophic network with relatively high mosaic-level diversity and an abundance of aquatic organisms. Planktonic and benthic invertebrates (e.g. molluscs and crustaceans) often sustain large fish populations, with fish nursery grounds being a common feature. Waterbirds (e.g. cormorants), seabirds (e.g. gannets), top-order predatory fish, mammals (e.g. dolphins and dugongs), and reptiles (e.g. marine turtles and crocodilians) exploit these locally abundant food sources. Many of these organisms in upper trophic levels are highly mobile and move among different estuaries through connected ocean waters or by flying. Others migrate between different ecosystem types to complete their various life-history phases, although some may remain resident for long periods. Most biota tolerate a broad range of salinity or are spatially structured by gradients. The complex spatial mixes of physical and chemical characteristics, alongside seasonal, inter-annual, and sporadic variability in aquatic conditions, induce correspondingly large spatial-temporal variability in food webs. Low-salinity plumes, usually proportional to river size and discharge, may extend far from the shore, producing tongues of ecologically distinct conditions into the marine environment.

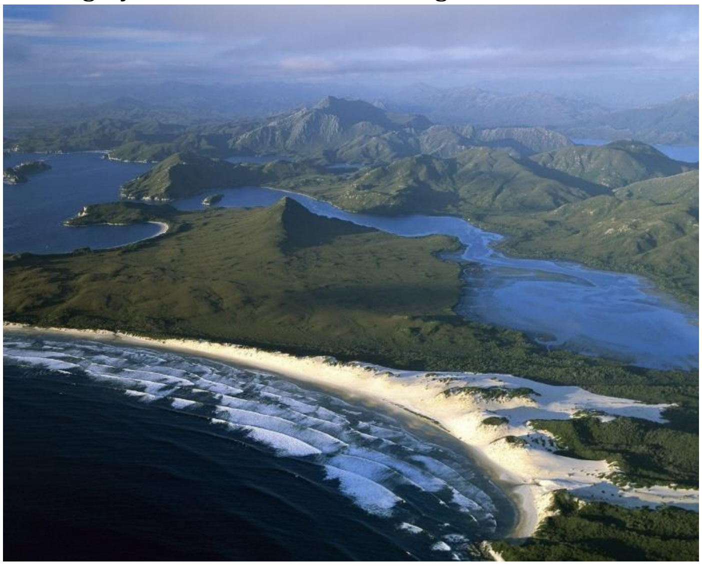

Port Davey, with permanent opening to Southern Ocean, Tasmania, Australia.
Credit: Jean-Paul Ferrero / AUSCAPE

Ecological drivers: Characteristics of these coastal systems are governed by the relative dominance of saline marine waters versus freshwater inflows (groundwater and riverine), the latter depending on the seasonality of precipitation and evaporative stress. Geomorphology ranges from wave-dominated estuaries to drowned river valleys, tiny inlets, and enormous bays. These forms determine the residence time, proportion, and distribution of saline waters, which in turn affect salinity and thermal gradients and stratification, dissolved O2 concentration, nutrients, and turbidity. The water column is closely linked to mudflats and sandflats, in which an array of biogeochemical processes occurs, including denitrification and N-fixation, and nutrient cycling.

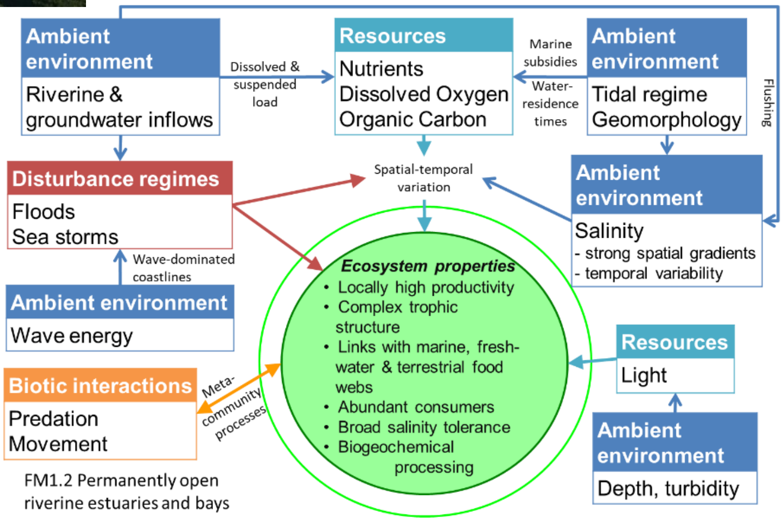

Distribution: Coastlines of most landmasses but rarely on arid or polar coasts.

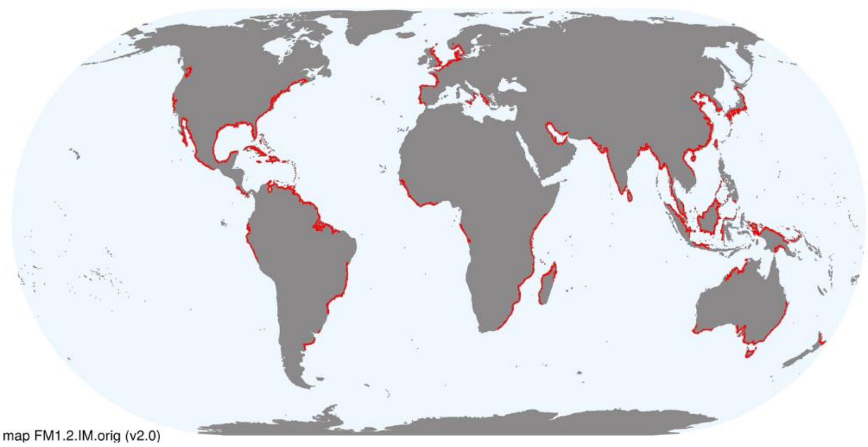

References:

Gillanders BM (2007) Linking terrestrial-freshwater and marine environments: an example from estuarine systems. *Marine ecology* (Eds. Connell, SD and Gillanders, BM). Chapter 11. Oxford University Press, Melbourne.

Map is for illustrative purposes only and does not support spatial analyses unless formally validated.

FM1.3 Intermittently closed and open lakes and lagoons

Ecosystem properties: These coastal water bodies have high spatial and temporal variability in structure and function, which depends largely on the status of the lagoonal entrance (open or closed). Communities have low species richness compared to those of permanently open estuaries (FM1.2). Lagoonal entrance closure prevents the entry of marine organisms and resident biota must tolerate significant variation in salinity, inundation, dissolved oxygen, and nutrient concentrations. Resident communities are dominated by opportunists with short lifecycles. Trophic networks are generally detritus-based, fuelled by substantial inputs of organic matter from the terrestrial environment and, to a lesser extent, from the sea. As net sinks of organic matter from the land, productivity is often high, and lagoons may serve as nursery habitats for fish. High concentrations of polyphenolic compounds (e.g. tannins) in the water and periods of low nutrient input limit phytoplankton populations. Benthic communities dominate with attached algae, microphytobenthos and micro- and macro-fauna being the dominant groups. The water column supports plankton and small-bodied fish. Emergent and fringing vegetation is a key source of detrital carbon to the food webs, and also provides important structural habitats. Saltmarsh and reedbeds (MFT1.3) can adjoin lagoons while seagrasses (M1.1) occupy sandy bottoms of some lagoons, but mangroves (MFT1.2) are absent unless the entrance opens.

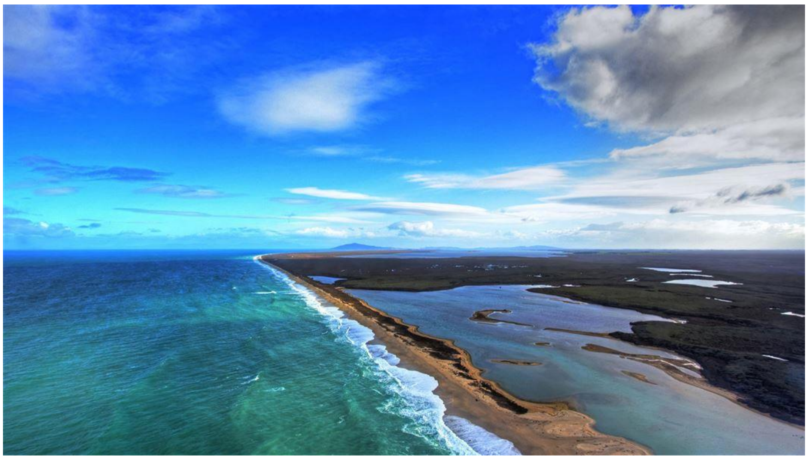

Waituna lagoon, New Zealand.
Credit: Phil Melgren / Dept of Conservation (NZ)

Ecological drivers: These are shallow coastal water bodies that are intermittently connected with the ocean. Some lagoons are mostly open, closing only once every few decades. Some open and close frequently and some are closed most of the time. The timing and frequency of entrance opening depend on trade-offs between sedimentation from fluvial and shoreline processes (which close the connection) and flushes of catchment inflow or erosive wave action (which open the entrance). Opening leads to changes in water level, tidal amplitude, salinity gradients, temperature, nutrients, dissolved oxygen, and sources of organic carbon. Human-regulated opening influences many of these processes.

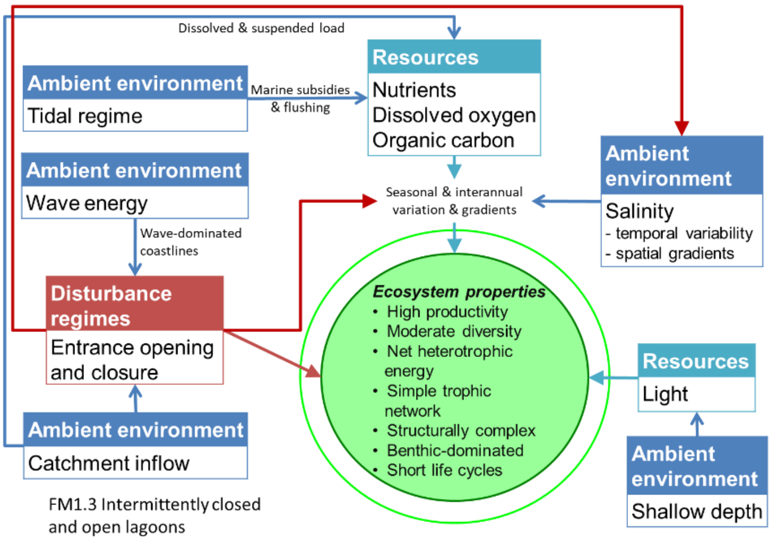

FM1.3 Intermittently closed and open lagoons

Distribution: Wave-dominated coastlines worldwide, but prevalent along microtidal to low mesotidal mid-latitude coastlines with high inter-annual variability in rainfall and wave climate. Intermittent closed open lakes and lagoons (ICOLLs) are most prevalent in Australia (21% of global occurrences), South Africa (16%), and Mexico (16%).

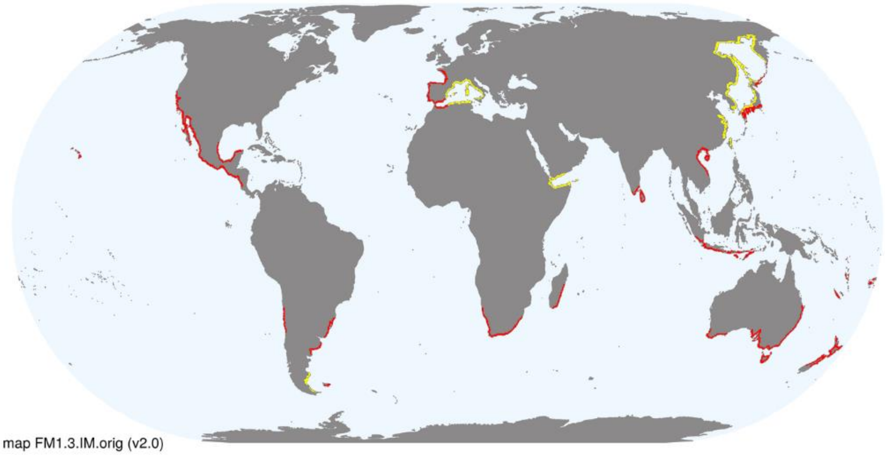

References:

Maher W, Mikac KM, Foster S, Spooner D, Williams D (2011) Form and functioning of micro size Intermittent Closed Open Lake Lagoons (ICOLLs) in NSW, Australia. *Lagoons: Biology, management and environmental impact* Friedman AG (ed). Nova Science Publisher. New York.

McSweeney SL, Kennedy DM, Rutherfurd ID, Stout JC (2017) Intermittently Closed/Open Lakes and Lagoons: Their global distribution and boundary conditions. *Geomorphology* 292:142-52.

Map is for illustrative purposes only and does not support spatial analyses unless formally validated.

M1. Marine shelf biome

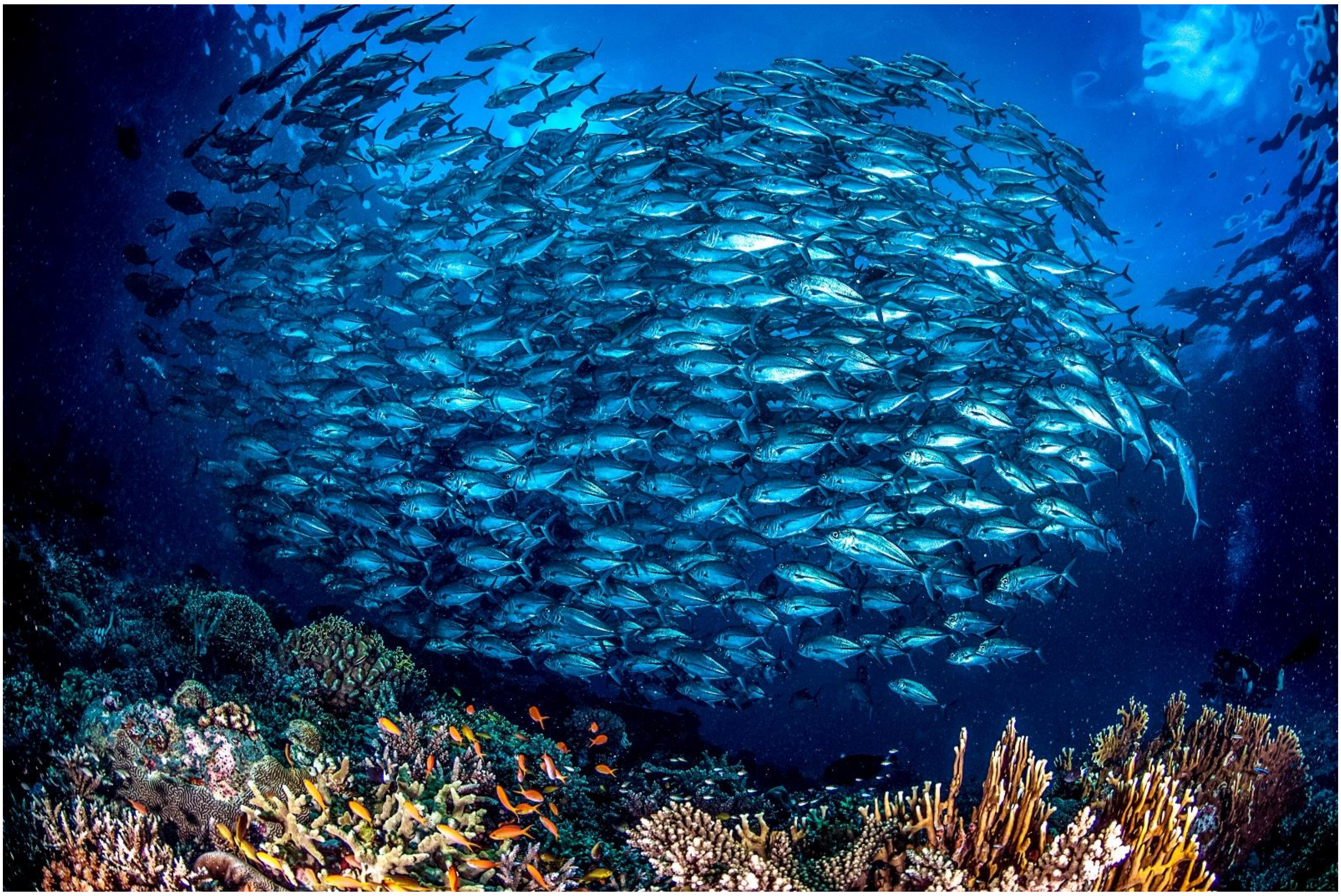

School of trevally (Caranx sexfasciatus) above a coral reef, The Philippines.

Credit: Giordano Cipriani / Getty Images

The Marine shelf biome is distributed globally between the shoreline and deep sea-floor biomes and is dominated by benthic productivity. It includes ecosystems with biogenic substrates (such as seagrass meadows, kelp forests, oyster beds, and coral reefs) and minerogenic substrates including rocky reefs, sandy bottoms, and muddy bottoms.

The availability of light and nutrients are key structuring factors, influencing productivity and ecosystem structure and function. Turbidity and depth gradients influence light availability. Productivity depends on upwelling currents that deliver nutrients from the deep ocean floor as well as the strength of nutrient inputs from the land, delivered largely by fluvial systems. Light is influenced by depth gradients but also by water clarity (cf. turbidity) and determines whether macrophytes and animals dependent on photosynthetic symbionts are able to establish and persist.

Additionally, whether the bottom type is hard or soft dictates whether sessile organisms can dominate, forming biogenic habitats that protrude into the water column. A shallow water biome, the marine shelf is shaped by kinetic wave energy and, in polar regions, also ice scour. Positive feedback loops, whereby the habitat structures formed by sessile organisms dampens kinetic energy, can enable ecotypes to persist under marginally suitable conditions. The strength of top-down control by consumers can be an important factor in determining community structure.

Depending on the benthic biota, energy sources can vary from net autotrophic to net heterotrophic. Temperature and (to a lesser extent) salinity influence the presence and identity of dominant habitat-forming biota. Currents can influence ecotypes by determining patterns of larval dispersal and the flow of resources.

Contributors: MJ Bishop, AH Altieri, SN Porter, RJ Orth, DA Keith

M1.1 Seagrass meadows

Ecosystem properties: Seagrass meadows are important sources of organic matter, much of which is retained by seagrass sediments. Seagrasses are the only subtidal marine flowering plants and underpin the high productivity of these systems. Macroalgae and epiphytic algae, also contribute to productivity, supporting both detritus production and autochthonous trophic structures, but compete with seagrasses for light. The complex three-dimensional structure of the seagrass provides shelter and cover to juvenile fish and invertebrates, binds sediments and, at fine scales, dissipates waves and currents. Seagrass ecosystems support infauna living amongst their roots, epifauna, and epiflora living on their shoots and leaves, as well as nekton in the water column. They have a higher abundance and diversity of flora and fauna compared to surrounding unvegetated soft sediments and comparable species richness and abundances to most other marine biogenic habitats. Mutualisms with lucinid molluscs may influence seagrass persistence. Mesograzers (such as amphipods and gastropods) play an important role in controlling epiphytic algal growth on seagrass. Grazing megafauna such as dugongs, manatees and turtles can contribute to patchy seagrass distributions, although they tend to 'garden' rather than deplete seagrass.

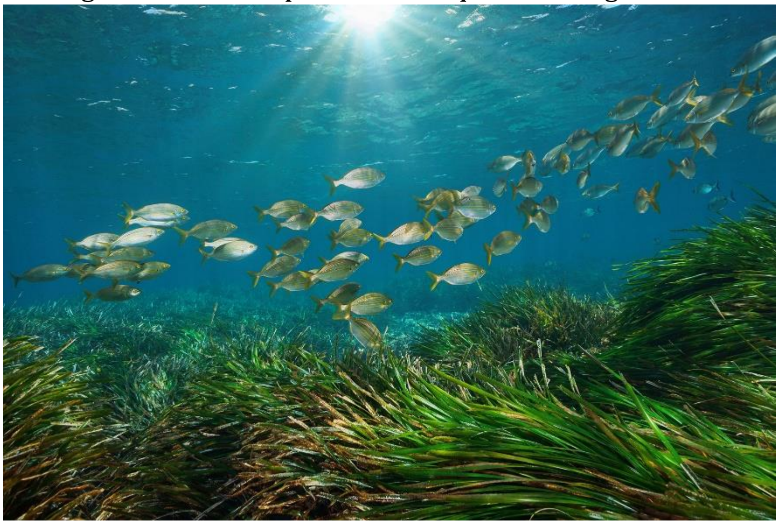

Cabo de Gata Nijar, Andalusia, Spain.
Credit: Damocean / istock photo

Ecological drivers: Typically found in the subtidal zone on soft sedimentary substrates but also occasionally on rocky substrates on low- to moderate-energy coastlines with low turbidity and on intertidal shorelines. Minimum water depth is determined mainly by wave orbital velocity, tidal exposure, and wave energy (i.e. waves disturb seagrass and mobilise sediment), while maximum depth is limited by the vertical diminution of light intensity in the water column. Seagrass growth can be limited by nitrogen and phosphorous availability, but in eutrophic waters, high nutrient availability can lead to the overgrowth of seagrasses by epiphytes and shading by algal blooms, leading to ecosystem collapse. Large storm events and associated wave action lead to seagrass loss.

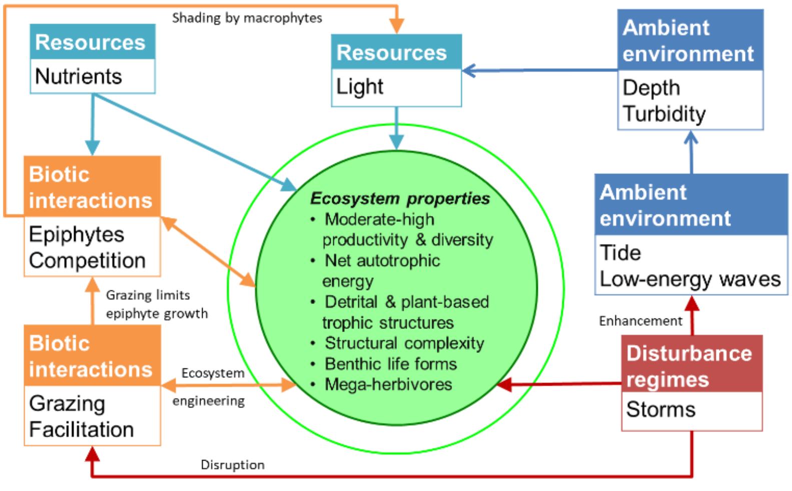

M1.1 Seagrass meadows

Distribution: Widely distributed along the temperate and tropical coastlines of the world.

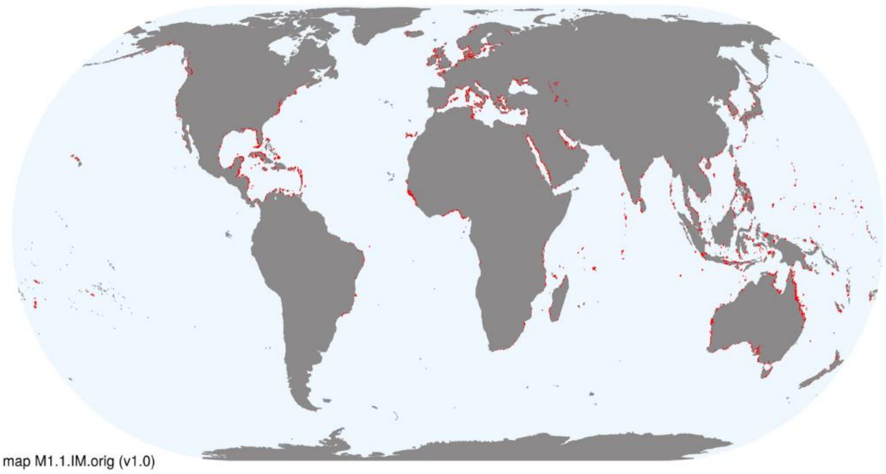
map M1.1.IM.orig (v1.0)

References:

De Boer WF (2007) Seagrass–sediment interactions, positive feedbacks and critical thresholds for occurrence: a review. *Hydrobiologia* 591: 5-24.

Larkum WD, Orth RJ, Duarte CM (2006) *Seagrasses: biology, ecology and conservation* (Springer: The Netherlands).

Orth RJ, Carruthers TJ, Dennison WC, Duarte CM, Fourqurean JW, Heck KL, Hughes AR, Kendrick GA, Kenworthy WJ, Olyarnik S, Short F (2006) A global crisis for seagrass ecosystems. *Bioscience* 56: 987-996.

Van der Heide T, Govers LL, De FouwJ, Olff H, van der Geest M, van Katwijk MM, Piersma T, van de Koppe Jl, Silliman BR, Smolders AJP, van Gils JA (2012) A three-stage symbiosis forms the foundation of seagrass ecosystems. *Science* 336,1432-1434.

Map is for illustrative purposes only and does not support spatial analyses unless formally validated.

Contributors: DA Keith, MJ Bishop, SN Porter, EJ Gregr

M1.2 Kelp forests

Ecosystem properties: Kelps are benthic brown macroalgae (Order Laminariales) forming canopies that shape the structure and function of these highly productive, diverse ecosystems. These large (up to 30 m in length), fast-growing (up to 0.5 m/day) autotrophs produce abundant consumable biomass, provide vertical habitat structure, promote niche diversity, alter light-depth gradients, dampen water turbulence, and moderate water temperatures. Traits such as large, flexible photosynthetic organs, rapid growth, and strong benthic holdfasts enable kelps to persist on hard substrates in periodically turbulent waters. These kelps may occur as scattered individuals in other ecosystem types, but other macroalgae (e.g. green and coralline) rarely form canopies with similar function and typically form mixed communities with sessile invertebrates (see M1.5 and M1.6). Some kelps are fully submerged, while others form dense canopies on the water surface, which profoundly affect light, turbulence, and temperature in the water column. Interactions among co-occurring kelps are generally positive or neutral, but competition for space and light is an important evolutionary driver. Kelp canopies host a diverse epiflora and epifauna, with some limpets having unique kelp hosts. Assemblages of benthic invertebrate herbivores and detritivores inhabit the forest floor, notably echinoderms and crustaceans. The structure and diversity of life in kelp canopies provide forage for seabirds and mammals, such as gulls and sea otters, while small fish find refuge from predators among the kelp fronds. Herbivores keep epiphytes in check, but kelp sensitivity to herbivores makes the forests prone to complex trophic cascades when declines in top predators release herbivore populations from top-down regulation. This may drastically reduce the abundance of kelps and dependent biota and lead to replacement of the forests by urchin barrens, which persist as an alternative stable state.

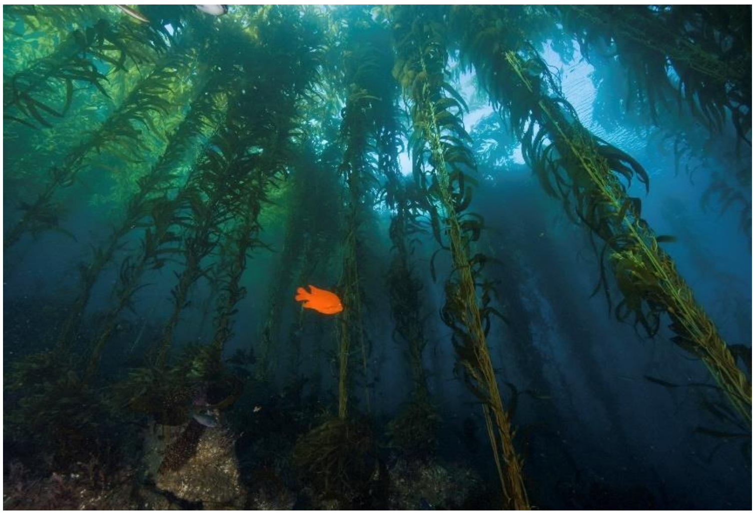

Garibaldi in giant kelp forest, Channel Islands, California, USA.
Credit: Brett Seymour / US NPS

Ecological drivers: Kelp forests are limited by light, nutrients, salinity, temperature, and herbivory. Growth rates are limited by light and proximity to sediment sources. High nutrient requirements are met by terrestrial runoff or upwelling currents, although eutrophication can lead to transition to turf beds. Truncated thermal niches limit the occurrence of kelps in warm waters. Herbivory on holdfasts influences recruitment and can constrain reversals of trophic cascades, even when propagules are abundant. Kelp forests occur on hard substrates in the upper photic zone and rely on wave action and currents for oxygen. Currents also play important roles in dispersing the propagules of kelps and associated organisms. Storms may dislodge kelps, creating gaps that may be maintained by herbivores or rapidly recolonized.

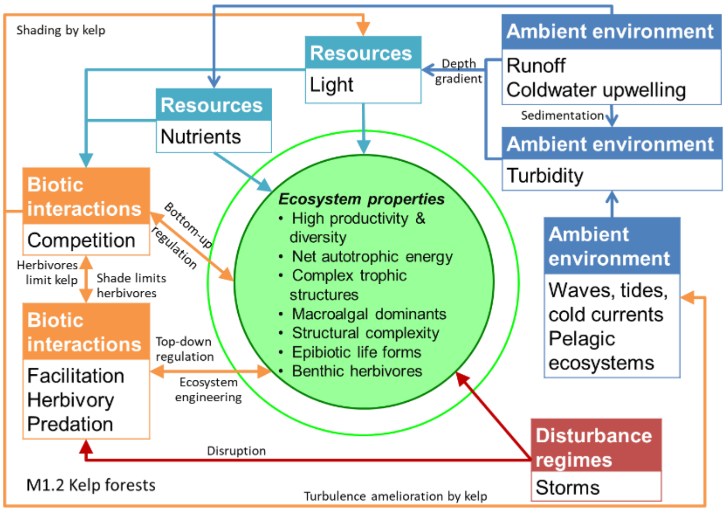

Distribution: Nearshore rocky reefs to depths of 30 m in temperate and polar waters. Absent from warm tropical waters but present in upwelling zones off Oman, Namibia, Cape Verde, Peru, and the Galapagos.

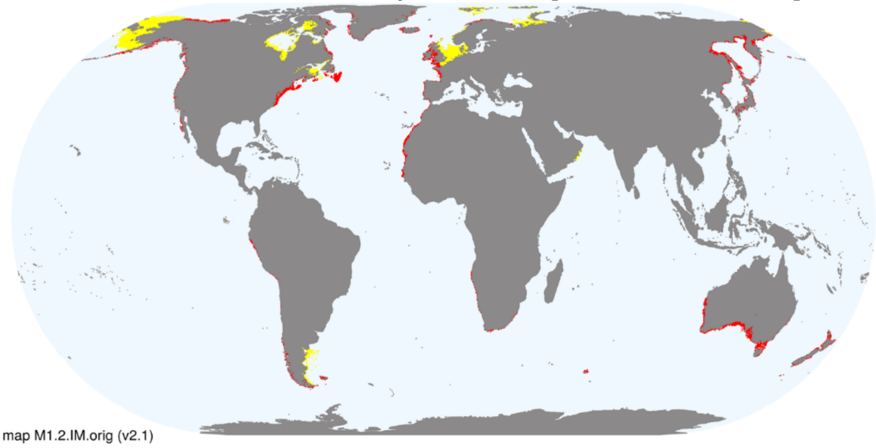

map M1.2.IM.orig (v2.1)

References:

Bennett S, Wernberg T, Anderson RJ, Bolton JJ et al. (2015) Canopy interactions and physical stress gradients in subtidal communities. *Ecology Letters* 18: 677–686.

Steneck RS, Graham MH, Bourque BJ, Corbett D, Erlandson JM, Estes JA, Tegner MJ (2002) Kelp forest ecosystems - biodiversity, stability, resilience and future. *Environmental Conservation* 29: 436–459.

Map is for illustrative purposes only and does not support spatial analyses unless formally validated.

Contributors: BA Polidoro, S Rossi, CRC Sheppard, SN Porter, DA Keith

M1.3 Photic coral reefs

Ecosystem properties: Coral reefs are biogenic structures that have been built up and continue to grow over decadal timescales as a result of the accumulation of calcium carbonate laid down by hermatypic (scleractinian) corals and other organisms. Reef-building corals are mixotrophic colonies of coral polyps in endosymbiotic relationships with photosynthesizing zooxanthellae that assimilate solar energy and nutrients, providing almost all of the metabolic requirements for their host. The corals develop skeletons by extracting dissolved carbonate from seawater and depositing it as aragonite crystals. Corals reproduce asexually, enabling the growth of colonial structures. They also reproduce sexually, with mostly synchronous spawning related to annual lunar cues. Other sessile organisms including sponges, soft corals, gorgonians, coralline algae, and other algae add to the diversity and structural complexity of coral reef ecosystems. The complex three-dimensional structure provides a high diversity of habitat niches and resources that support a highly diverse and locally endemic marine biota, including crustaceans, polychaetes, holothurians, echinoderms, and other groups, with one-quarter of marine life estimated to depend on reefs for food and/or shelter. Diversity is high at all taxonomic levels relative to all other ecosystems. The trophic network is highly complex, with functional diversity represented on the benthos and in the water column by primary producers, herbivores, detritivores, suspension-feeders, and multiple interacting levels of predators. Coral diseases also play a role in reef dynamics. The vertebrate biota includes fish, snakes, turtles, and mammals. The fish fauna is highly diverse, with herbivores and piscivores displaying a wide diversity of generalist and specialist diets (including parrot fish that consume corals), feeding strategies, schooling and solitary behaviours, and reproductive strategies. The largest vertebrates include marine turtles and sharks.

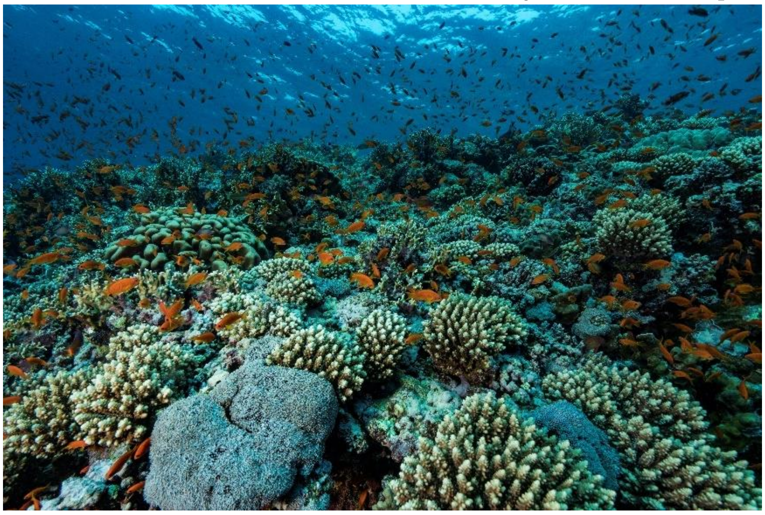

Lyretail anthias above a coral reef, Red Sea, Egypt.
Credit: Alexis Rosenfeld / Getty Images

Ecological drivers: Coral reefs are limited to warm, shallow (rarely >60 m depth), clear, relatively nutrient-poor, open coastal waters, where salinity is 3.0–3.8% and sea temperatures vary (17–34°C). Cooler temperatures are insufficient to support coral growth, while warmer temperatures cause coral symbiosis to break down (i.e. bleaching). Reef geomorphology varies from atolls, barrier reefs, fringing reefs and lagoons to patch reefs depending upon hydrological and geological conditions. Reef structure and composition vary with depth gradients such as light intensity and turbulence, exposure gradients, such as exposure itself and sedimentation. Storm regimes and marine heat waves (thermal anomalies) drive cycles of reef destruction and renewal.

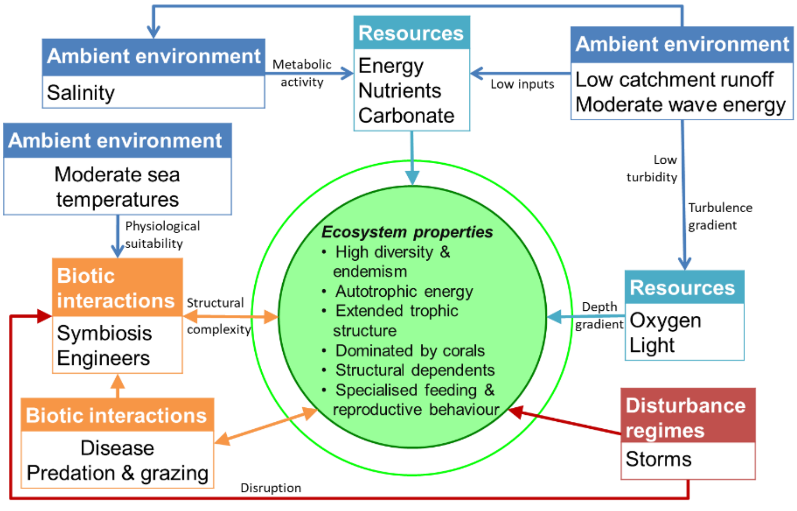

M1.3 Photic coral reefs

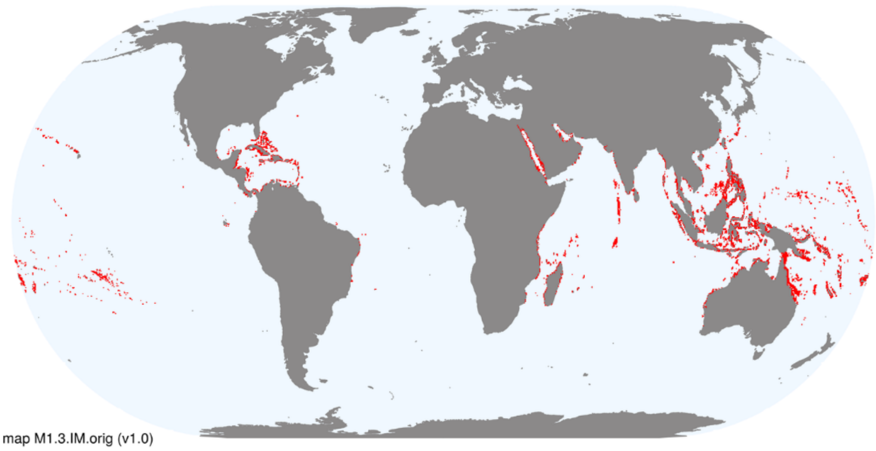

map M1.3.IM.orig (v1.0)

Distribution: Tropical and subtropical waters on continental and island shelves, mostly within latitudes of 30°N and 30°S.

References:

Sheppard C, Davy S, Pilling G, Graham N (2018) *The biology of coral reefs* 2nd Edition. Oxford University Press, Oxford.

Contributors: MJ Bishop, SE Swearer, SN Porter, AH Altieri, DA Keith

M1.4 Shellfish beds and reefs

Ecosystem properties: These ecosystems are founded on intertidal or subtidal 3-dimensional biogenic structures formed primarily by high densities of oysters and/or mussels, which provide habitat for a moderate diversity of algae, invertebrates, and fishes, few of which are entirely restricted to oyster reefs. Structural profiles may be high (i.e. reefs) or low (i.e. beds). Shellfish reefs are usually situated on sedimentary or rocky substrates, but pen shells form high-density beds of vertically orientated non-gregarious animals in soft sediments. Sessile filter-feeders dominate these strongly heterotrophic but relatively high-productivity systems. Tides bring in food and carry away waste. Energy and matter in waste is processed by a subsystem of deposit-feeding invertebrates. Predators are a small component of the ecosystem biomass, but are nevertheless important in influencing the recruitment, biomass, and diversity of prey organisms (e.g. seastar predation on mussels). Shellfish beds and reefs may influence adjoining estuaries and coastal waters physically and biologically. Physically, they modify patterns of currents, dampen wave energy and remove suspended particulate matter through filter-feeding. Biologically, they remove phytoplankton and produce abundant oyster biomass. They function in biogeochemical cycling as carbon sinks, by increasing denitrification rates, and through N burial/sequestration. Relatively (or entirely) immobile and thin-shelled juveniles are highly susceptible to benthic predators such as crabs, fish, and birds. Recruitment can depend on protective microhabitats provided either by abiogenic or biogenic structures. In intertidal environments, the survival of shellfish can increase with density due to self-shading and moisture retention.

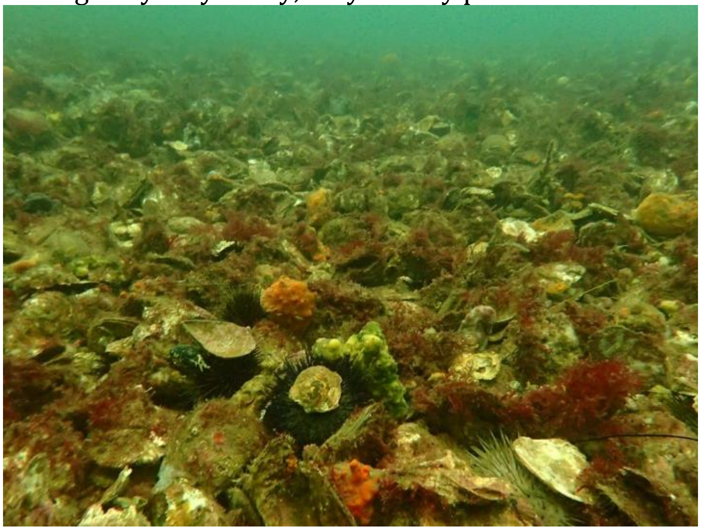

Oyster reef, Georges Bay, Tasmania, Australia.
Credit: Chris Gillies, The Nature Conservancy

Ecological drivers: The availability of hard substrate (including shells of live or dead conspecifics) can limit the establishment of reef-forming shellfish, though a few occur on soft substrates. Many shellfish are robust to changes in salinity, closing their valves for days to weeks to avoid adverse conditions, but salinity may indirectly influence survival by determining susceptibility to parasites. High suspended sediment loads caused by high energy tides, rainfall, and run-off events or the erosion of coastal catchments can smother larvae and impede filter-feeding. Most reef- or bed-building shellfish cannot survive prolonged periods of low dissolved oxygen. They are also sensitive to climate change stressors such as temperature (and associated increased risk of desiccation for intertidal species), as well as lowered pH as they are calcifiers. In subtidal environments, the formation of reefs can help elevate animals above anoxic bottom waters.

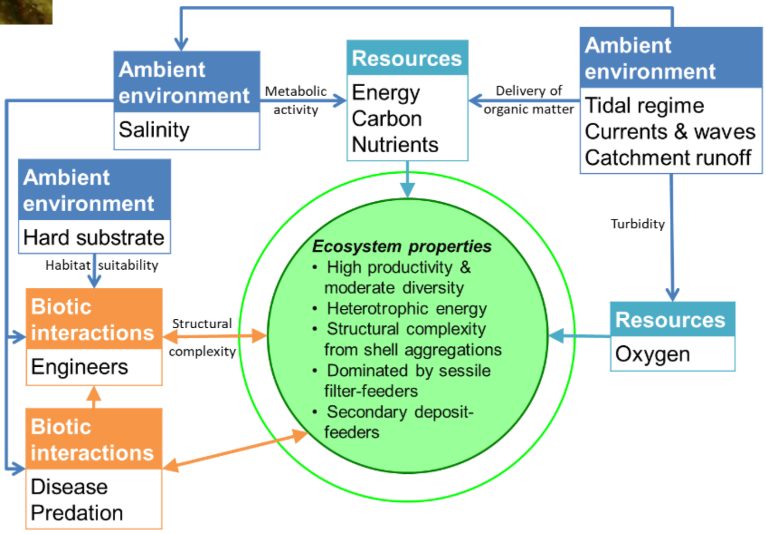

M1.4 Shellfish beds & reefs

Distribution: Estuarine and coastal waters of temperate and tropical regions, extending from subtidal to intertidal zones. Present-day distributions are shaped by historic overharvest, which has removed 85% of reefs globally.

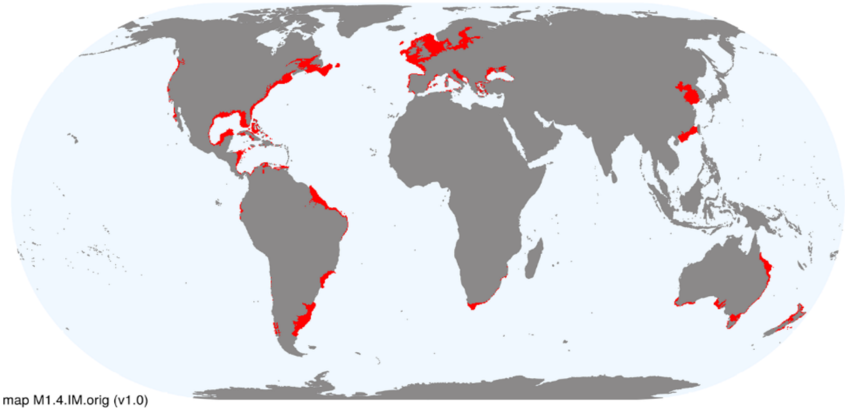

map M1.4.IM.orig (v1.0)

References:

Beck MW, Brumbaugh RD, Airoldi L, Carranza A,. Coen LD, Crawford C, Defeo O, Edgar GJ, Handcock B, Kay MC, Lenihan HS, Luckenbach M, Toropova CL, Zhang G, Guo X (2011) Oyster reefs at risk and recommendations for conservation, restoration, and management. *BioScience* 61: 107-116.

Dame RF, Patten BC (1981) Analysis of energy flows in an intertidal oyster reef. *Marine Ecology Progress Series* 5:115-124.

Map is for illustrative purposes only and does not support spatial analyses unless formally validated.

M1.5 Photo-limited marine animal forests

Ecosystem properties: These benthic systems are characterised by high densities of megabenthic, sessile heterotrophic suspension feeders or coralline algae that act as habitat engineers and dominate a subordinate autotrophic biota. Unlike coral reefs and shellfish beds, the major sessile animals in these animal forests include sponges, aphotic corals, hydroids, ascidians, hydrocorals, bryozoans, polychaetes, and bivalves (the latter only dominate in M1.4). Various coralline algae may be present in Marine animal forests, but rhodoliths, are never dominant (cf. M1.10). All these organisms engineer complex three-dimensional biogenic structures, sometimes of a single species or distinct phylogenetic groups. The structural complexity generates environmental heterogeneity and habitat, promoting a high diversity of invertebrate epifauna, with microphytobenthos and fish. Endemism may be high. Low light limits primary productivity especially by macroalgae, although microphytobenthos can be important. Energy flow and depth-related processes distinguish these systems from their deepwater aphotic counterparts (M3.7). Nonetheless, these systems are strongly heterotrophic, relying on benthic-pelagic coupling processes. Consequently, these systems are generally of moderate productivity and are often found near shallower, less photo-limited, high-productivity areas. Complex biogeochemical cycles govern nutrient release, particle retention, and carbon fixation. Biodiversity is enhanced by secondary consumers (i.e. deposit-feeding and filter-feeding invertebrates). Predators may influence the biomass and diversity of epifaunal prey organisms. Recruitment processes in benthic animals can be episodic and highly localised and, together with slow growth rates, limit recovery from disturbance.

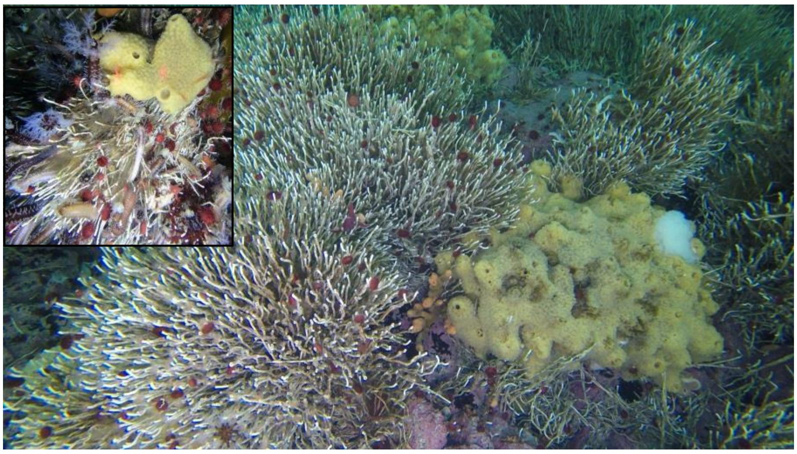

Polychaete reef ~20m depth, Ellis Fjord, Antarctica.
Credit: Jonathan Stark, Australian Antarctic Division

Ecological drivers: Light is generally insufficient to support abundant macroalgae but is above the photosynthetic threshold for coralline algae and cyanobacteria. Light is limited by diffusion through deepwater, surface ice cover, turbidity from river outflow, or tannins in terrestrial runoff. Low to moderate temperatures may further limit productivity. These systems are generally found on hard substrates but can occur on soft substrates. Currents or resuspension and lateral transport processes are important drivers of benthic-pelagic coupling, hence food and nutrient supply. Natural physical disturbances are generally of low severity and frequency, but ice scour can generate successional mosaics where animal forests occur on subpolar shelves.

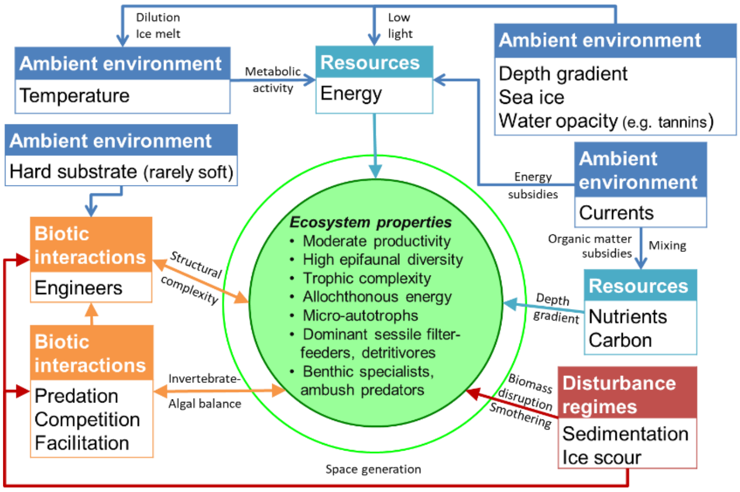

M1.5 Photo-limited marine animal forests & rhodolith beds

Distribution: Tropical to polar coastal waters extending from the shallow subtidal to the 'twilight' zone on the shelf. Present-day distributions are likely to have been modified by benthic trawling.

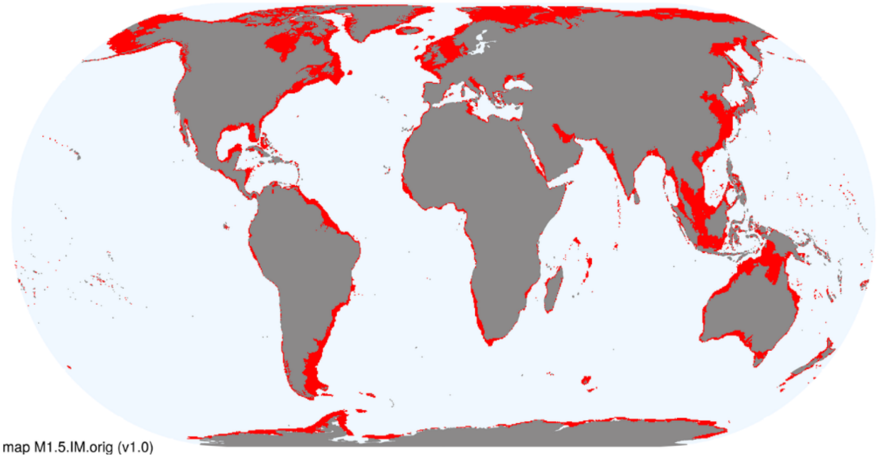
map M1.5.IM.orig (v1.0)

References:

Baldwin C, Tornabene L, Robertson D (2018) Below the mesophotic. *Scientific Reports* 8: 4920.

Riosmena-Rodríguez, R. (2017) Natural history of rhodolith/maërl beds: Their role in near-shore biodiversity and management. In: *Rhodolith/ Maërl Beds: A global perspective* (Eds. R Riosmena-Rodríguez, W Nelson, J Aguirre), pp. Springer, Cham.

Rossi S. (2013) The destruction of the 'animal forests' in the oceans: towards an over-simplification of the benthic ecosystems. *Ocean & coastal management* 84: 77-85.

Rossi S, Bramanti L, Gori, A, Orejas C (2017) *Marine Animal Forests: The ecology of benthic biodiversity Hotspots* Springer, Berlin.

Map is for illustrative purposes only and does not support spatial analyses unless formally validated.

Contributors: BA Polidoro, EJ Gregr, SE Swearer, SN Porter, DA Keith

M1.6 Subtidal rocky reefs

Ecosystem properties: Submerged rocky reefs host trophically complex communities lacking a dense macroalgal canopy (cf. M1.2). Sessile primary producers and invertebrate filter-feeders assimilate autochthonous and allochthonous energy, respectively. Mobile biota occur in the water column. Reef-associated organisms have diverse dispersal modes. Some disperse widely as adults, some have non-dispersing larvae, others with sessile adult phases develop directly on substrates, or have larval stages or spores dispersed widely by currents or turbulence. Sessile plants include green, brown, and red algae. To reduce dislodgement in storms, macroalgae have holdfasts, while smaller species have low-growing 'turf' life forms. Many have traits such as air lacunae or bladders that promote buoyancy. Canopy algae are sparse at the depths or levels of wave exposure occupied by this functional group (cf. kelp forests in M1.2). Algal productivity and abundance decline with depth due to diminution of light and are also kept in check by periodic storms and a diversity of herbivorous fish, molluscs, and echinoderms. The latter two groups and some fish are benthic and consume algae primarily in turf form or at its juvenile stage before stipes develop. Sessile invertebrates occur throughout. Some are high-turbulence specialists (e.g. barnacles, ascidians and anemones), while others become dominant at greater depths as the abundance of faster-growing algae diminishes (e.g. sponges and red algae). Fish include both herbivores and predators. Some are specialist bottom-dwellers, while others are more generalist pelagic species. Herbivores promote diversity through top-down regulation, influencing patch dynamics, trophic cascades and regime shifts involving kelp forests in temperate waters (M1.2). Mosaics of algal dominance, sessile invertebrate dominance, and barrens may shift over time. Topographic variation in the rocky substrate promotes habitat diversity and spatial heterogeneity. This provides refuges from predators but also hiding places for ambush predators including crustaceans and fish.

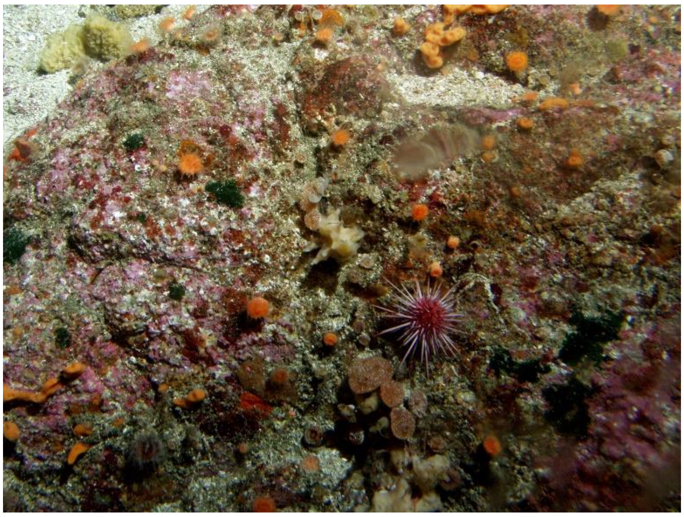

Red Urchin (Strongylocentrotus franciscanus) and invertebrates on rocky reef habitat, Cordell Bank National Marine Sanctuary, California.
Credit: Dale Roberts, NOAA / CBNMS

Ecological drivers: Minerogenic rocky substrates with variable topography and cobbles are foundational to the habitats of many plants and animals, influencing how they capture resources and avoid predation. A strong depth gradient and substrate structures (e.g. overhangs and caves) limit light availability, as does turbidity. Currents and river outflows are crucial to the delivery of resources, especially nutrients, and also play a key role in biotic dispersal. Animal waste is a key nutrient source sustaining algal productivity in more nutrient-limited systems. Salinity is relatively constant through time (3.5% on average). Turbulence from subsurface wave action promotes substrate instability and maintains high levels of dissolved oxygen. Episodic storms generating more extreme turbulence shift sand and dislodge larger sessile organisms, create gaps that may be maintained by herbivores or rapidly recolonized.

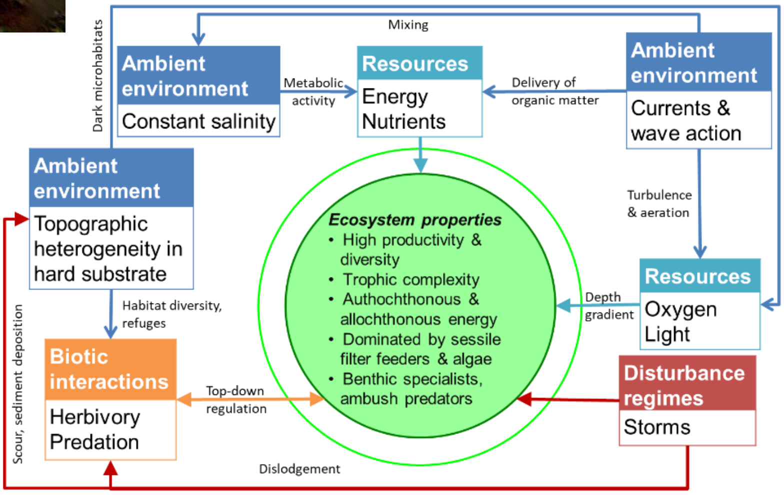

M1.6 Subtidal rocky reefs

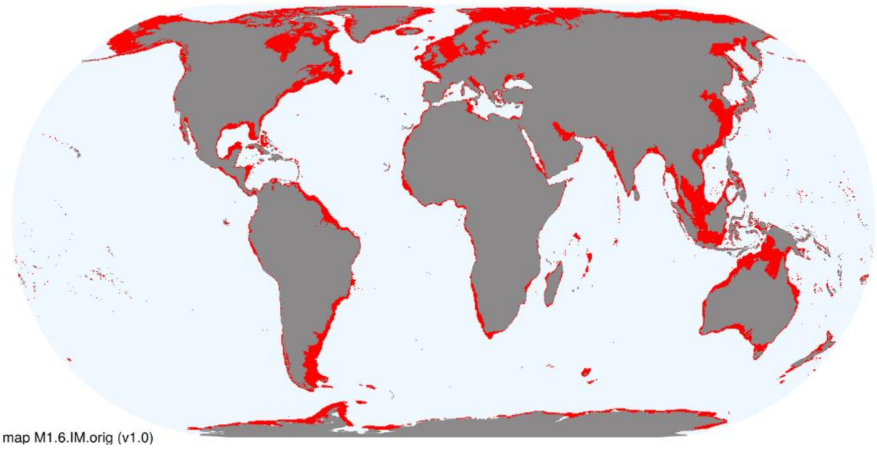

map M1.6.IM.orig (v1.0)

Distribution: Widespread globally on rocky parts of continental and island shelves.

References:

Sebens K (1985) The ecology of the rocky subtidal zone. *American Scientist* 73: 548-557.

Map is for illustrative purposes only and does not support spatial analyses unless formally validated.

M1.7 Subtidal sand beds

Ecosystem properties: Medium to coarse-grained, unvegetated, and soft minerogenic sediments show moderate levels of biological diversity. The trophic network is dominated by consumers with very few *in situ* primary producers. Interstitial microalgae and planktonic algae are present, but larger benthic primary producers are limited either by substrate instability or light, which diminishes with depth. In shallow waters where light is abundant and soft substrates are relatively stable, this group of systems is replaced by group M1.1, which is dominated by vascular marine plants. In contrast to those autochthonous systems, Subtidal sand beds rely primarily on allochthonous energy, with currents generating strong bottom flows and a horizontal flux of food. Sandy substrates tend to have less organic matter content and lower microbial diversity and abundance than muddy substrates (M1.8). Soft sediments may be dominated by invertebrate detritivores and suspension-feeders including burrowing polychaetes, crustaceans, echinoderms, and molluscs. Suspension-feeders tend to be most abundant in high-energy environments where waves and currents move sandy sediments, detritus, and living organisms. The homogeneity and low structural complexity of the substrate exposes potential prey to predation, especially from pelagic fish. Large bioturbators such as dugongs, stingrays and whales may also be important predators. Consequently, many benthic animals possess specialised traits that enable other means of predator avoidance, such as burrowing, shells, or camouflage.

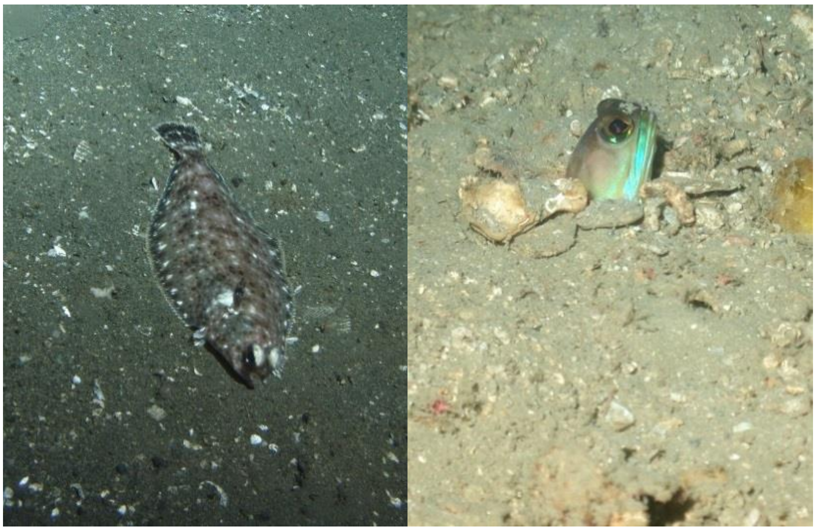

Left: Dover sole on sand, Cordell Bank National Marine Sanctuary, California.
Right: Jawfish in sandy burrow.

Credit: Rick Starr, NOAA/CBNMS (left); Andrew David, NOAA/NMFS (right)

Ecological drivers: The substrate is soft, minerogenic, low in organic matter, relatively homogeneous, structurally simple, and mobile. The pelagic waters are moderate to high-energy environments, with waves and currents promoting substrate instability. Nonetheless, depositional processes dominate over erosion, leading to net sediment accumulation. Fluvial inputs from land and the erosion of headlands and sea cliffs contribute sediment, nutrients, and organic matter, although significant lateral movement is usually driven by longshore currents. Light availability diminishes with depth. Mixing is promoted by waves and currents, ensuring low temporal variability in salinity, which averages 3.5%.

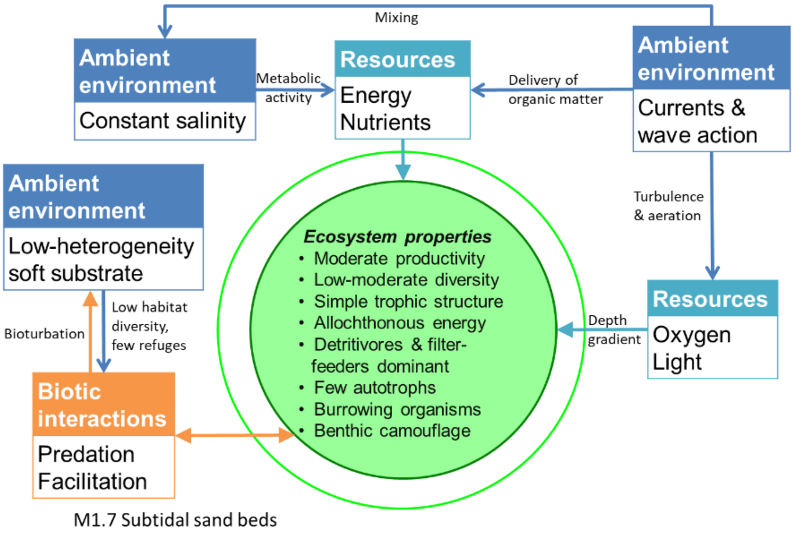

Distribution: Globally widespread around continental and island shelves.

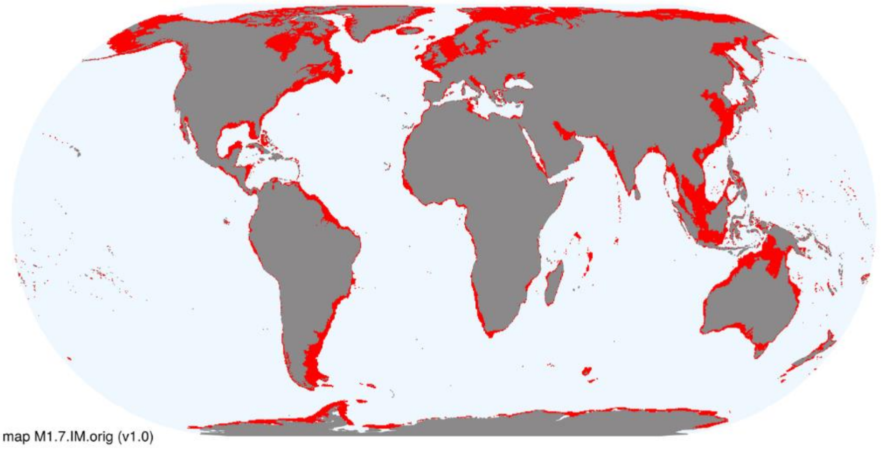

References:

Byers JE, Grabowski JH (2014) Soft-sediment communities. *Marine community ecology and conservation* (Eds. MD Bertness, JF Bruno, BR Silliman, JJ Stachowicz), pp227-249. Sinauer, Sunderland.

Snelgrove PVR (1999) Getting to the bottom of marine biodiversity: sedimentary habitats. *Bioscience* 49: 129-138.

Map is for illustrative purposes only and does not support spatial analyses unless formally validated.

M1.8 Subtidal mud plains

Ecosystem properties: The muddy substrates of continental and island shelves support moderately productive ecosystems based on net allochthonous energy sources. In situ primary production is contributed primarily by microphytobenthos, mainly benthic diatoms with green microalgae, as macrophytes are scarce or absent. Both decline with depth as light diminishes. Drift algae can be extensive over muddy sediments, particularly in sheltered waters. Abundant heterotrophic microbes process detritus. The microbial community mediates most of the biogeochemical cycles in muddy sediments, a feature distinguishing these ecosystems from subtidal sand beds (M1.7). Deposit feeders (notably burrowing polychaetes, crustaceans, echinoderms, and molluscs) are important components of the trophic network as the low kinetic energy environment promotes vertical food fluxes, which they are able to exploit more effectively than suspension-feeders. The latter are less abundant on subtidal mud plains than on rocky reefs (M1.6) and Subtidal sand beds (M1.7) where waters are more turbulent and generate stronger lateral food fluxes. Deposit feeders may also constrain the abundance of co-occurring suspension-feeders by disturbing benthic sediment that resettles and smothers their larvae and clogs their filtering structures. Nonetheless, suspension-feeding tube worms may be common over muddy sediments when settlement substrates are available. Although such interference mechanisms may be important, competition is generally weak. In contrast, foraging predators, including demersal fish, may have a major structuring influence on these systems through impacts on the abundance of infauna, particularly on settling larvae and recently settled juveniles, but also adults. Burrowing is a key mechanism of predator avoidance and the associated bioturbation is influential on microhabitat diversity and resource availability within the sediment.

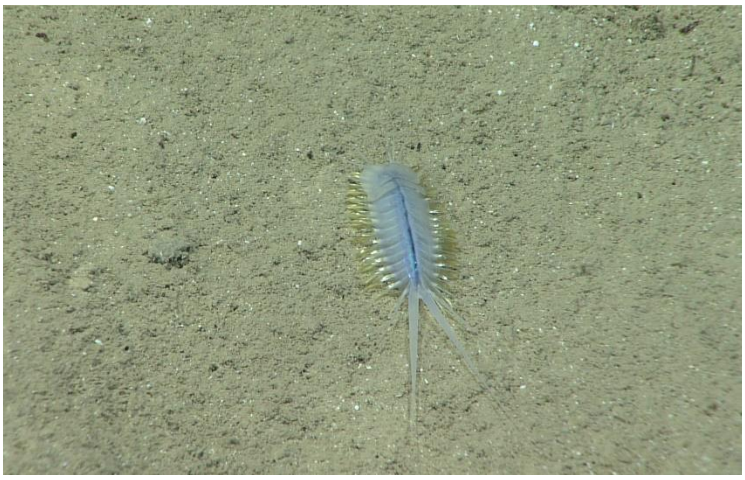

Polynoid worm on soft sediment, Caribbean Sea.
Credit: NOAA Office of Ocean Exploration and Research

Ecological drivers: These depositional systems are characterised by low kinetic energy (weak turbulence and currents), which promotes the accumulation of fine-textured, stable sediments that are best developed on flat bottoms or gentle slopes. The benthic surface is relatively homogeneous, except where structure is engineered by burrowing organisms. The small particle size and poor interchange of interstitial water limit oxygen supply, and anaerobic conditions are especially promoted where abundant in-fall of organic matter supports higher bacterial activity that depletes dissolved oxygen. On the other hand, the stability of muddy substrates makes them more conducive to the establishment of permanent burrows. Bioturbation and bio-irrigation activities by a variety of benthic fauna in muddy substrates are important contributors to marine nutrient and biogeochemical cycling as well as the replenishment of oxygen.

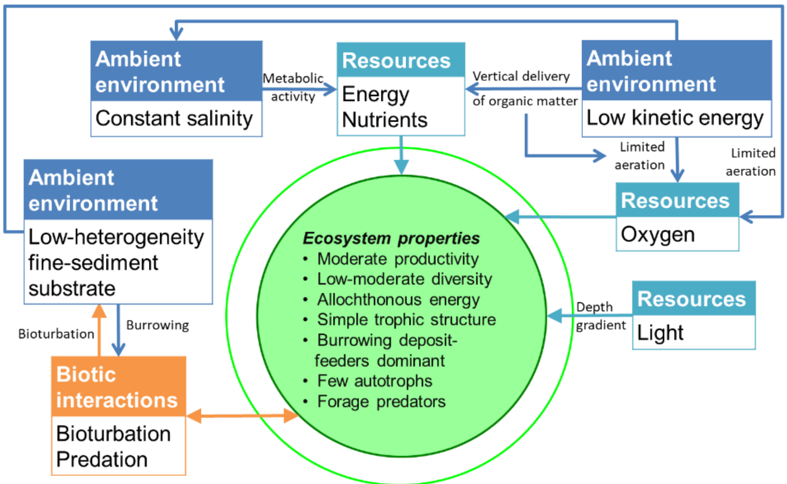

M1.8 Subtidal mud plains

Distribution: Globally distributed in the low-energy waters of continental and island shelves.

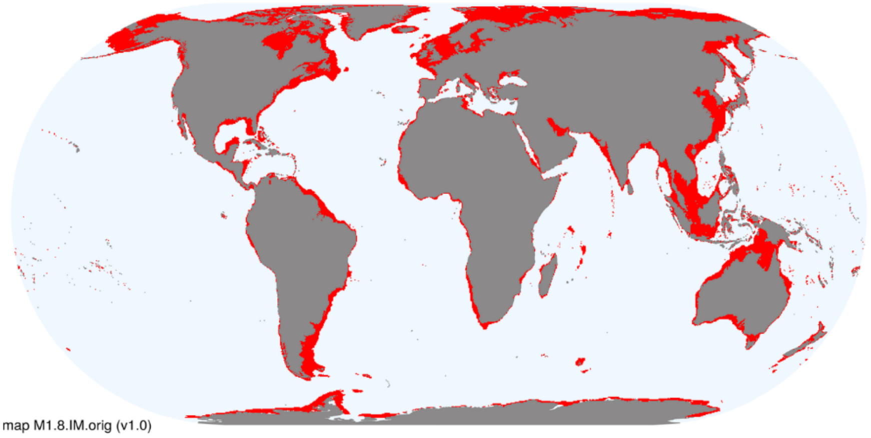

References:

Byers JE, Grabowski JH (2014) Soft-sediment communities. *Marine community ecology and conservation* (Eds. MD Bertness, JF Bruno, BR Silliman, JJ Stachowicz), pp227-249. Sinauer, Sunderland.

Snelgrove PVR (1999) Getting to the bottom of marine biodiversity: sedimentary habitats. *Bioscience* 49: 129-138.

Map is for illustrative purposes only and does not support spatial analyses unless formally validated.

Contributors: KE Watermeyer, EJ Gregr, RR Rykaczewski , LJ Shannon, IM Suthers, DA Keith

M1.9 Upwelling zones

Ecosystem properties: Upwelled, nutrient-rich water supports very high net autochthonous primary production, usually through diatom blooms. These, in turn, support high biomass of copepods, euphausiids (i.e. krill), pelagic and demersal fish, marine mammals, and birds. Fish biomass tends to be dominated by low- to mid-trophic level species such as sardine, anchovy, and herring. The abundance of these small pelagic fish has been hypothesised to drive ecosystem dynamics through 'wasp-waist' trophic control. Small pelagic fish exert top-down control on the copepod and euphausiid plankton groups they feed on and exert bottom-up control on predatory fish, although diel-migrant mesopelagic fish (M2.2) may have important regulatory roles. Abundant species of higher trophic levels include hake and horse mackerel, as well as pinnipeds and seabirds. Highly variable reproductive success of planktivorous fish reflects the fitness of spawners and suitable conditions for concentrating and retaining eggs and larvae inshore prior to maturity.

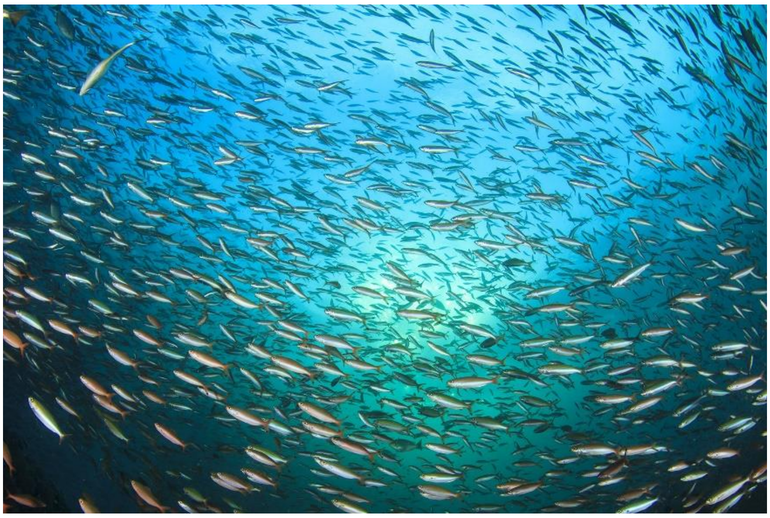

Sardine school in an upwelling zone, Malaysia.
Credit: Rich Carey / Shutterstock

Ecological drivers: Upwelling is a wind-driven process that draws cold, nutrient-rich water towards the surface, displacing warmer, nutrient-depleted waters. The strength of upwelling depends on interactions between local current systems and the Coriolis effect that causes divergence, generally on the eastern boundaries of oceans. The rate of upwelling, the offshore transportation of nutrients, and the degree of stratification in the water column once upwelling has occurred all determine the availability of nutrients to plankton, and hence the size and structure of the community that develops after an event. The main upwelling systems around the world extend to depths of up to 500 m at the shelf break, although primary production is restricted to the epipelagic zone (<200 m). Upwelling zones are characterised by low sea-surface temperatures and high chlorophyll a concentrations, high variability due to large-scale interannual climate cycles (e.g El Niño Southern Oscillation), as well as the pulsed and seasonal nature of the driving winds, and periodic low-oxygen, low pH events due to high biological activity and die-offs.

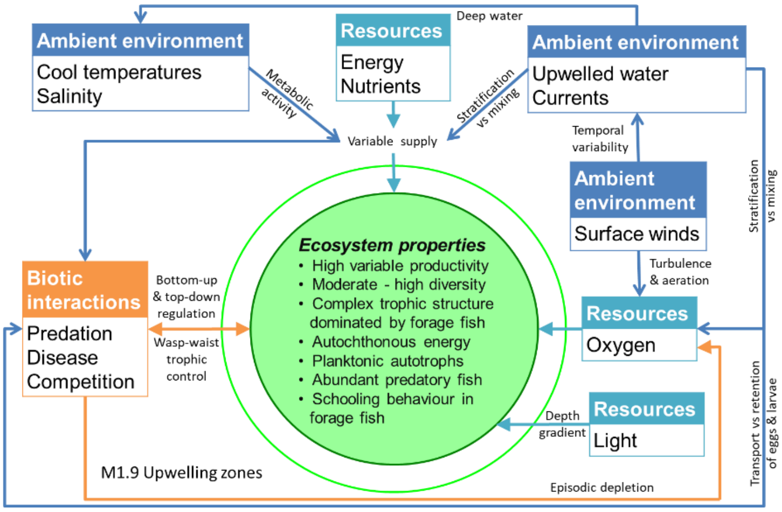

Distribution: The most productive upwelling zones are coastal, notably in four major eastern-boundary current systems (the Canary, Benguela, California, and Humboldt). Weaker upwelling processes occurring in the open ocean are included in M2.1 (e.g. along the intertropical convergence zone).

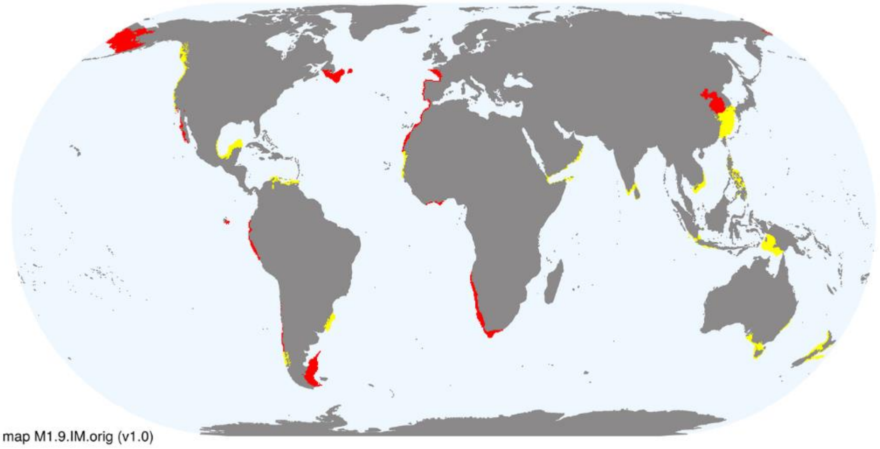

References:

Cury P, Shannon L, Shin YJ (2003) The functioning of marine ecosystems: a fisheries perspective. *Responsible fisheries in the marine ecosystem* (Eds. M Sinclair, G Valdimarsson), p103–123. FAO, Rome, & CABI Publishing, Wallingford.

Hutchings L, Pitcher GC, Probyn TA, Bailey GW (1995) The chemical and biological consequences of coastal upwelling. *Upwelling in the oceans: Modern Processes and Ancient Records* (Eds. CP Summerhayes, KC Emeis, MV Angel, RL Smith, B Zeitzschel), pp. 65–81. John Wiley, New York.

Map is for illustrative purposes only and does not support spatial analyses unless formally validated.

Contributors: DA Keith, W Nelson, E Fragkopoulou, J Assis, N Schubert

M1.10 Rhodolith/Maërl beds

Ecosystem properties: Benthic carbonate ecosystems dominated by rhodoliths –non-geniculate (non-jointed), free-living, slow-growing, long-lived coralline algae – cover 30-100% of the seafloor within the beds, providing autochthonous energy to the system. Their pigments enable red algae to absorb more green - blue light efficiently, in addition to red-orange light. Rhodolith primary productivity is likely to be lower than in sea grasses (M1.1) and kelp forests (M1.2), although macrophytes add to primary production in shallow waters. They play a role in benthic nutrient cycling and represent significant long-term carbonate stores. Rhodoliths vary from smooth semi-spherical to complex fruticose structures that may form mono- or multi- specific aggregations typically composed of living and dead rhodoliths, as well as calcic sediments produced by breakdown. They can form 3-dimensional biogenic structures that facilitate coexistence of a diversity of benthic and demersal organisms, including algae, ascidians, sponges, macroinvertebrates and fish. Compared to coral reefs (M1.3), shellfish beds (M1.4) or marine animal forests (M1.5), where rhodoliths may be minor components, they are usually less rugose and less stable, due displacement or aggregation by water motion and bioturbators such as fish and macroinvertebrates. Large rhodoliths appear to facilitate deepwater kelp as well as feeding and reproduction in fish and invertebrates, supporting high species richness. High abundance of larval stages in these groups, suggests the intermediate rugosity of the beds is important for age-dependent predator evasion. Macroinvertebrate detritivores and herbivores well represented in rhodolith beds include crustaceans, molluscs, echinoderms and polychaetes. Closely associated microinvertebrates and microbes include small gastropods, ostracods, diatoms, foraminifera and bacteria. Bacterial guilds on rhodolith surfaces include photolithoautotrophs, anoxygenic phototrophs, anaerobic heterotrophs, sulfide oxidizers and methanogens, suggesting important roles in biomineralization. The biotic assemblages of rhodolith beds vary spatially, with depth gradients and temporally over diurnal and seasonal time scales. Fish and sponges that aggregate and agglutinate individual rhodoliths are thought to promote development of reefs from rhodolith beds, counter-balancing slow recovery from disturbance.

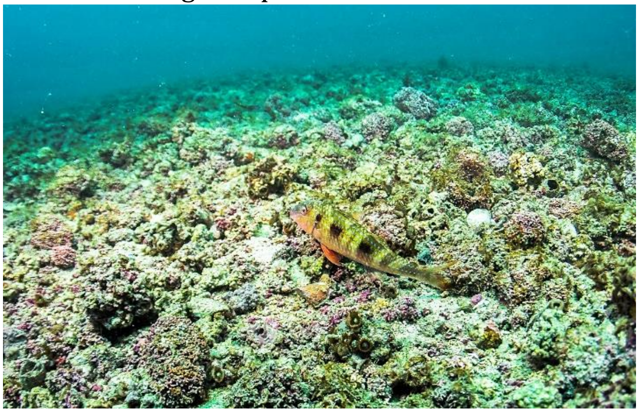

Rhodolith/Maërl bed near Arvoredo, Santa Catarina, Brazil.
Credit: Nadine Schubert

Ecological drivers: Rhodolith beds occur on coarse gravel, sandy or mixed muddy substrates. They are most common at depths of 5-150m, but may occur from the subtidal zone down to 270m below the ocean surface. Light availability, pH and hydrodynamics are important drivers of variation in biotic assemblages, as are temperatures. Rhodoliths form extensive beds on open coasts on the mid shelf and in tide-swept channels where the water column and suspended sediment diminish red light. Recurring disturbances such as bioturbation, wave action or storms physically restructure the system and initiate successional recovery.

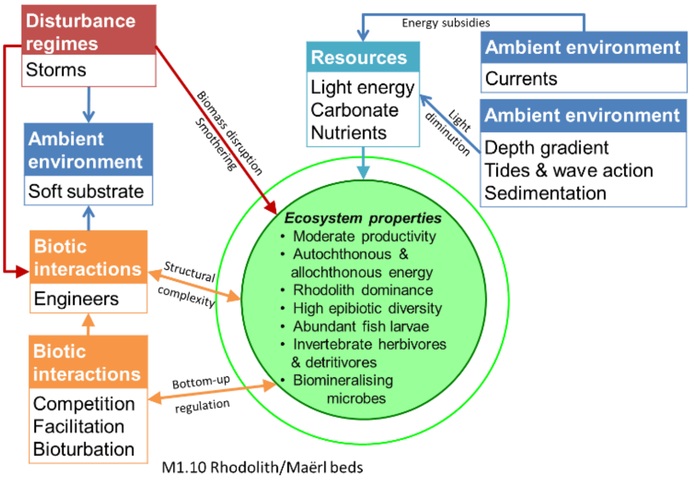

Distribution: Tropical to subpolar coastal waters, extensive areas in the north and southwest Atlantic, Mediterranean, Gulf of California and southern Australia.

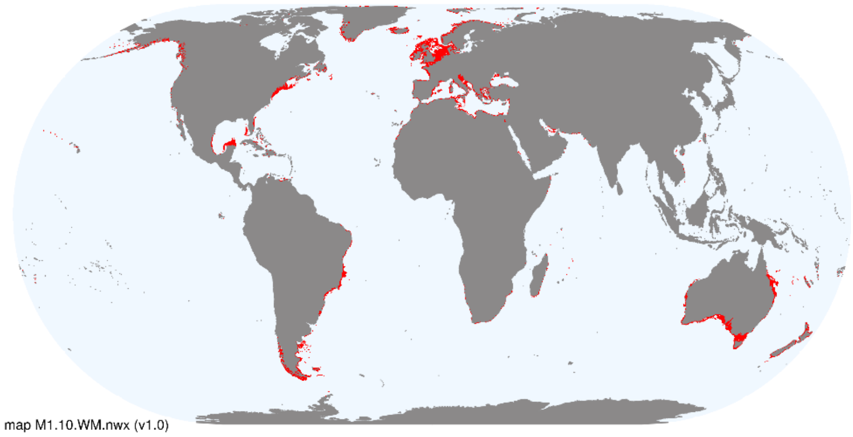

References:

Fragkopoulou E, Serrão EA, Horta PA, Koerich G, Assis J (2021) Bottom trawling threatens future climate refugia of rhodoliths globally. Frontiers in Marine Science 7, 594537.

Moura RL, Abieri ML, Castro GM et al. (2021) Tropical rhodolith beds are a major and belittled reef fish habitat. Scientific Reports 11: 794.

Pereira-Filho GH, Francini-Filho RB, Pierozzi I et al. (2015) Sponges and fish facilitate succession from rhodolith beds to reefs. Bulletin of Marine Science 91: 45-46.

Riosmena-Rodríguez, R Nelson W, Aguirre J (2017) Rhodolith/Maërl Beds: A global perspective. Springer, Cham.

Map is for illustrative purposes only and does not support spatial analyses unless formally validated.

M2. Pelagic ocean waters biome

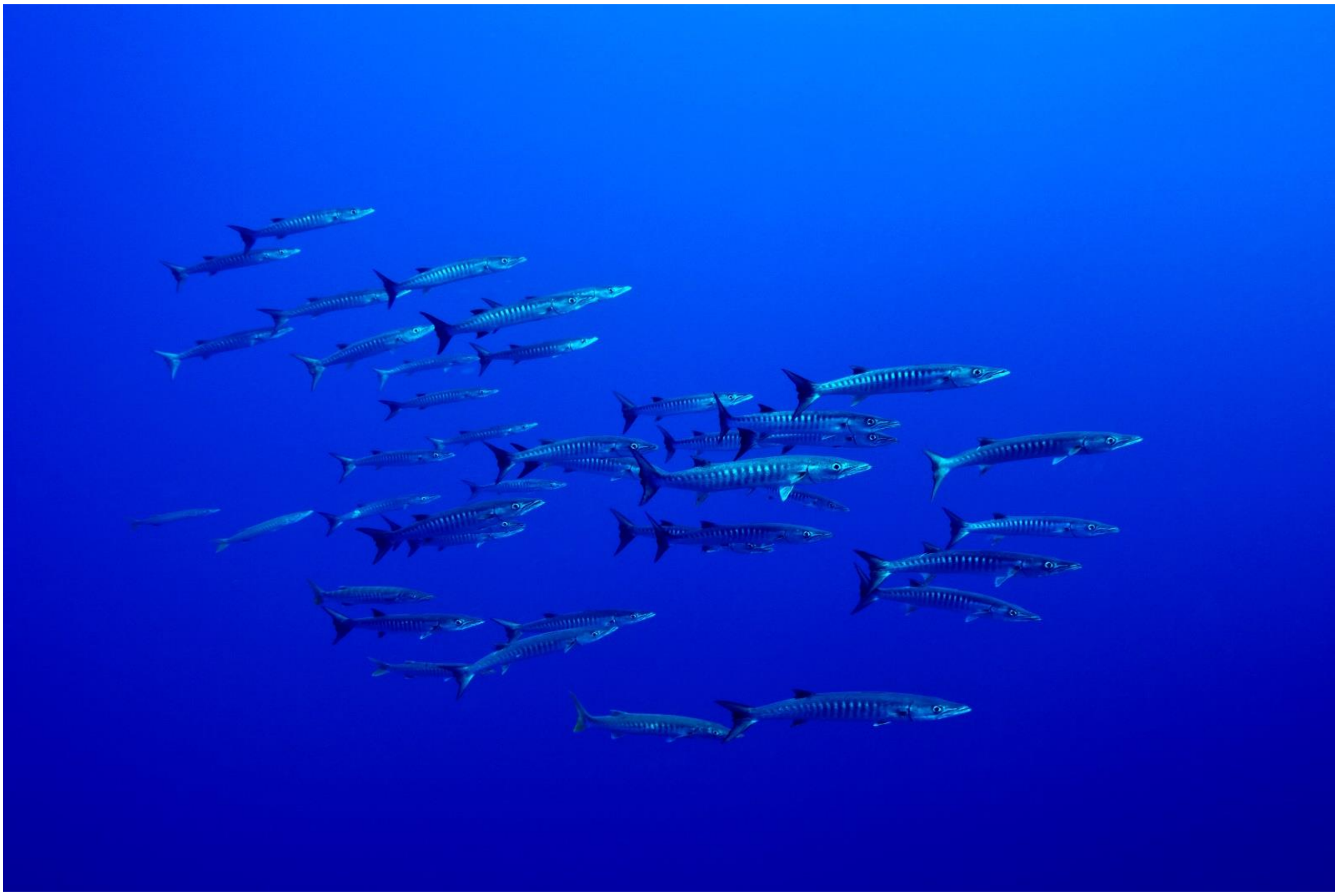

Shoal of predatory Barracuda, Kokod Point, Ringgold Isles Archipelago, Fiji.

Credit: Jason Edwards / Getty Images

The Pelagic ocean biome is the largest on earth, comprising the open-ocean water column across all latitudes. Diversity is highest in near-surface layers, particularly in niche habitats at water-mass boundaries where contrasting communities overlap. The depth gradient strongly structures the availability of light (and hence constraints on primary producers and visual predators), nutrients, and organic carbon and differentiates functional groups within the biome. Primary production is limited to the uppermost, euphotic, epipelagic zone, while deeper layers depend on allochthonous fluxes of carbon from above via sedimentation or vertically migrating organisms. This flux is diminished by consumers as it falls to deeper layers, resulting in low productivity and low diversity at the greatest depths.

A consistent Redfield ratio (Carbon:Nitrogen:Phosphorus) throughout the oceans marks feedbacks between planktonic biota and ocean chemistry, with deviations often attributable to nutrient deficiency. Iron and silica concentrations may also be limiting in some waters. Latitudinal variation in productivity relates to the local characteristics of the water column, such as temperature, mixing, and availability of nutrients and light.

Migration is a common characteristic in this biome, both horizontal between feeding and breeding areas, and diel or ontogenetic vertical migrations, such as that between the refuge provided by the low-light environment in the mesopelagic zone and the productive, upper epipelagic zone with its associated visual predators. Organisms in each depth zone display adaptations to the light environment. Bioluminescence is common in mesopelagic species, while species found at greater, aphotic depths may have enhanced sensory organs.

Contributors: KE Watermeyer, RR Rykaczewski, IG Priede, TT Sutton, DA Keith

M2.1 Epipelagic ocean waters

Ecosystem properties: The epipelagic or euphotic zone of the open ocean is the uppermost layer that is penetrated by enough light to support photosynthesis. The vast area of the ocean means that autochthonous productivity in the epipelagic layer, largely by diatoms, accounts for around half of all global carbon fixation. This in turn supports a complex trophic network and high biomass of diatoms, copepods (resident and vertical migrants), fish, cephalopods, marine mammals, and seabirds, including fast-swimming visual predators taking advantage of the high-light environment. The suitability of conditions for recruitment and reproduction depends on the characteristics of the water column, which vary spatially and impact productivity rates, species composition, and community size structure. Mid-ocean subtropical gyres, for example, are characteristically oligotrophic, with lower productivity than other parts of the ocean surface. In contrast to the rest of the epipelagic zone, upwelling zones are characterised by specific patterns of water movement that drive high nutrient levels, productivity, and abundant forage fish, and are therefore included in a different functional group (M1.9). Seasonal variation in productivity is greater at high latitudes due to lower light penetration and duration in winter compared to summer. The habitat and lifecycle of some specialised pelagic species (e.g. herbivorous copepods, flying fish) are entirely contained within epipelagic ocean waters, but many commonly occurring crustaceans, fish, and cephalopods undertake either diel or ontogenetic vertical migration between the epipelagic and deeper oceanic layers. These organisms exploit the food available in the productive epipelagic zone either at night (when predation risk is lower) or for the entirety of their less mobile, juvenile life stages. Horizontal migration is also common and some species (e.g. tuna and migratory whales) swim long distances to feed and reproduce. Other species use horizontal currents for passive migration, particularly smaller planktonic organisms or life stages, e.g. copepods and small pelagic fish larvae moving between spawning and feeding grounds. Unconsumed plankton and dead organisms sink from this upper oceanic zone, providing an important particulate source of nutrients to deeper, aphotic zones.

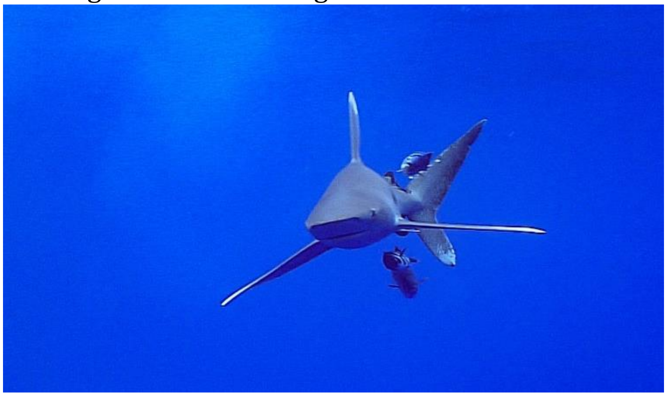

Visual predator, White-tipped ocean shark near surface, Wake Island, Pacific Ocean.
Credit: NOAA Office of Ocean Exploration and Research

Ecological drivers: The epipelagic zone is structured by a strong depth gradient in light, which varies seasonally at high latitudes. Light also varies with local turbidity, but at lower latitudes may extend to ~200 m where light attenuates to 1% of surface levels. Interaction at the surface between the ocean and atmosphere leads to increased seasonality, mixing, and warming, and makes this the most biologically and physicochemically variable ocean layer. Nutrient levels are spatially variable as a result. Salinity varies with terrestrial freshwater inputs, evaporation, and mixing, with greater variation in semi-enclosed areas (e.g. the Mediterranean Sea) than the open ocean.

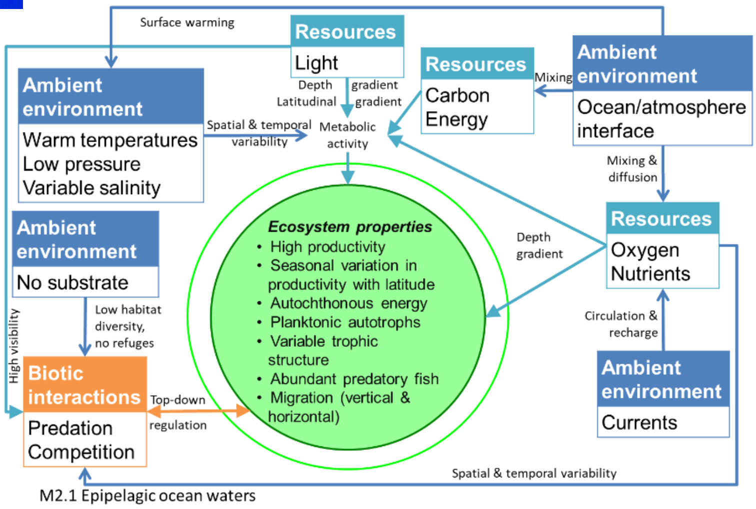

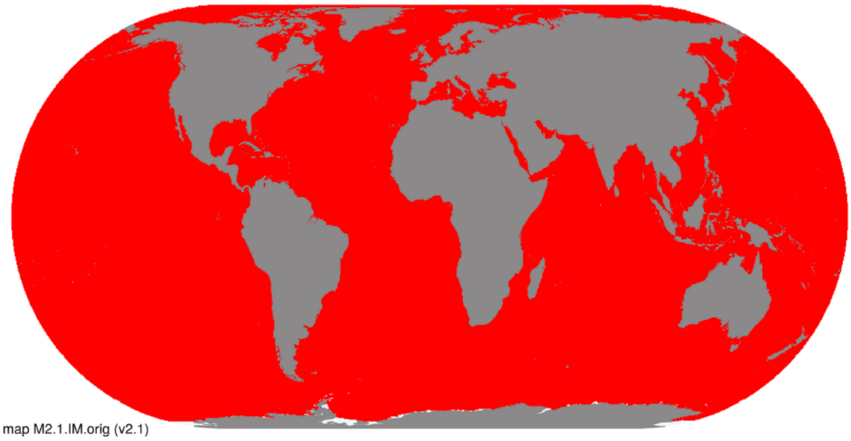

map M2.1.IM.orig (v2.1)

Distribution: The surface layer of the entire open ocean beyond the near-shore zone.

References:

Anderson TR, Martin AP, Lampitt RS, Trueman CN, Henson SA, Mayor D J (2019) Quantifying carbon fluxes from primary production to mesopelagic fish using a simple food web model. *ICES Journal of Marine Science* 76, 690–701.

Stal LJ (2016) The euphotic realm. *The Marine Microbiome* (Eds. LJ Stal, MS Cretoiu), pp. 209-225. Springer, Switzerland.

Sutton TT (2013) Vertical ecology of the pelagic ocean: Classical patterns and new perspectives. *Journal of Fish Biology* 83: 1508-1527.

Map is for illustrative purposes only and does not support spatial analyses unless formally validated.

M2.2 Mesopelagic ocean water

Ecosystem properties: The mesopelagic, dysphotic, or 'twilight' zone begins below the epipelagic layer and receives enough light to discern diurnal cycles but too little for photosynthesis. The trophic network is therefore dominated by detritivores and predators. The diverse organisms within this layer consume and reprocess allochthonous organic material sinking from the upper, photosynthetic layer. Hence, upper mesopelagic waters include layers of concentrated plankton, bacteria, and other organic matter sinking from the heterogeneous epipelagic zone (M2.1). Consumers of this material including detritivorous copepods deplete oxygen levels in the mesopelagic zone, more so than in other layers where oxygen can be replenished via diffusion and mixing at the surface or photosynthesis (as in the epipelagic zone), or where lower particulate nutrient levels limit biological processes (as in the deeper layers). Many species undertake diel vertical migration into the epipelagic zone during the night to feed when predation risk is lower. These organisms use the mesopelagic zone as a refuge during the day and increase the flow of carbon between ocean layers. Bioluminescence is a common trait present in more than 90% of mesopelagic organisms often with silvery reflective skin (e.g. lantern fish). Fish in the lower mesopelagic zone (>700 m) are less reflective and mobile due to reduced selection pressure from visual predators in low light conditions. These systems are difficult to sample, but advances in estimating fish abundances indicate that biomass is very high, possibly two orders of magnitude larger than global fisheries landings (1×10^{10} t).

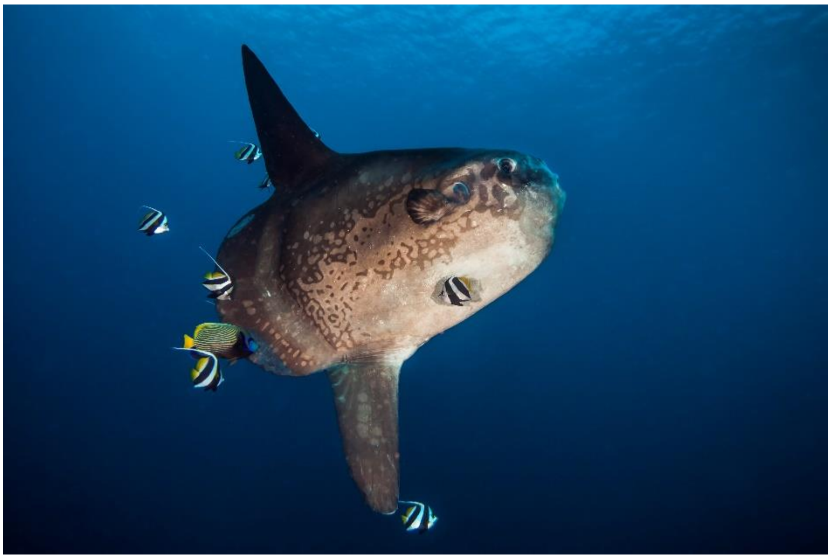

Ocean Sunfish (Mola ramseyi) cleaned by reef fish in deep water, Bali, Indonesia.
Credit: Steve Woods / Getty Images

Ecological drivers: Nutrient and energy availability depend on allochthonous fluxes of carbon from the upper ocean. Energy assimilation from sunlight is negligible. This is characteristically episodic and linked to events in the epipelagic zone. Buffered from surface forcing by epipelagic waters, the mesopelagic zone is less spatially and temporally variable, but the interface between the two zones is characterised by heterogeneous regions with greater biotic diversity. Areas of physicochemical discontinuity (e.g. current and water-mass boundaries and eddies) also result in niche habitats with increased local diversity. Oxygen minimum zones are formed in mesopelagic waters when biological activity reduces oxygen levels in a water mass that is then restricted from mixing by physical processes or features. Oxygen minimum zones support specialised biota and have high levels of biological activity around their borders.

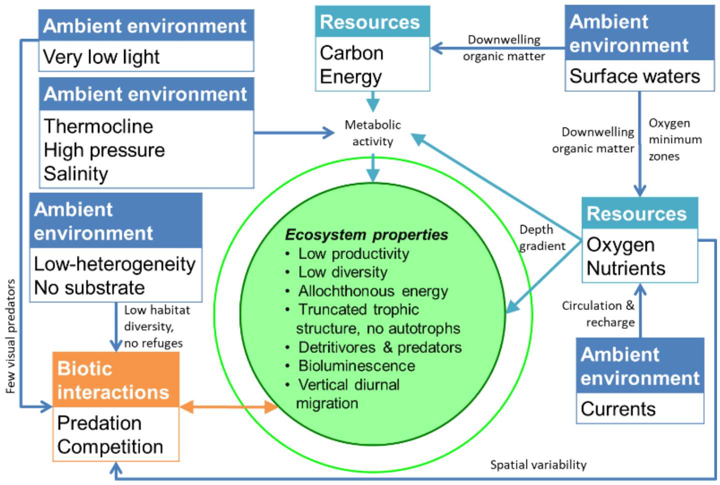

M2.2 Mesopelagic ocean waters

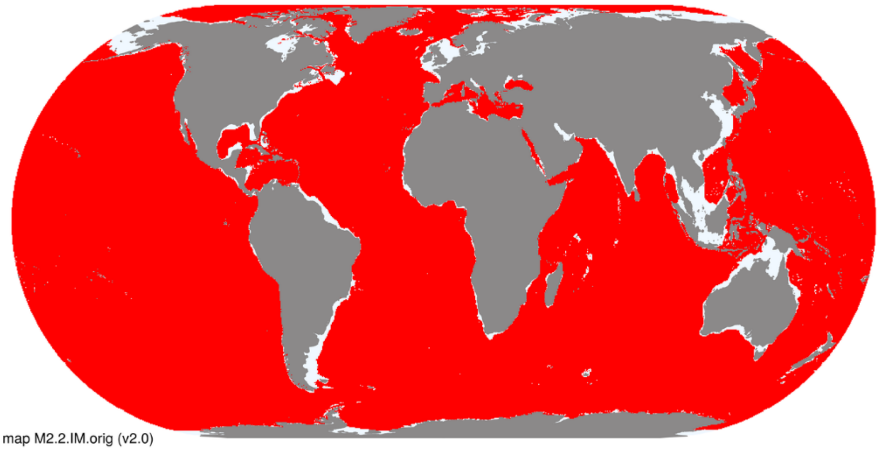

Distribution: Global oceans from a depth of ~200 m or where <1% of light penetrates, down to 1,000 m.

References:

Robinson C, Steinberg DK, Anderson TR, Aristegui J, Carlson CA, Frost JR, Ghiglione JF, Hernández-León S, Jackson GA, Koppelmann R (2010) Mesopelagic zone ecology and biogeochemistry – a synthesis. *Deep-Sea Research* II. 57: 1504-1518.

Sutton TT (2013) Vertical ecology of the pelagic ocean: Classical patterns and new perspectives. *Journal of Fish Biology* 83: 1508-1527.

Map is for illustrative purposes only and does not support spatial analyses unless formally validated.

M2.3 Bathypelagic ocean waters

Ecosystem properties: These are deep, open-ocean ecosystems in the water column, generally between 1,000–3,000 m in depth. Energy sources are allochthonous, derived mainly from the fallout of particulate organic matter from the epipelagic horizon (M2.1). Total biomass declines exponentially from an average of 1.45 mgC m-3 at 1,000 m depth to 0.16 mgC m-3 at 3,000 m. Trophic structure is truncated, with no primary producers. Instead, the major components are zooplankton, micro-crustaceans (e.g. shrimps), medusozoans (e.g. jellyfish), cephalopods, and four main guilds of fish (gelativores, zooplanktivores, micronektivores, and generalists). These organisms generally do not migrate vertically, in contrast to those in the mesopelagic zone (M2.2). Larvae often hatch from buoyant egg masses at the surface to take advantage of food sources. Long generation lengths (>20 years in most fish) and low fecundity reflect low energy availability. Fauna typically have low metabolic rates, with bathypelagic fish having rates of oxygen consumption ~10% of that of epipelagic fish. Fish are consequently slow swimmers with high water content in muscles and relatively low red-to-white muscle tissue ratios. They also have low-density bodies, reduced skeletons, and/or specialised buoyancy organs to achieve neutral buoyancy for specific depth ranges. Traits related to the lack of light include reduced eyes, lack of pigmentation, and enhanced vibratory and chemosensory organs. Some planktonic forms, medusas, and fish have internal light organs that produce intrinsic or bacterial bioluminescence to attract prey items or mates or to defend themselves. Most of the biota possess cell membranes with specialised phospholipid composition, intrinsic protein modifications, and protective osmolytes (i.e. organic compounds that influence the properties of biological fluids) to optimise protein function at high pressure.

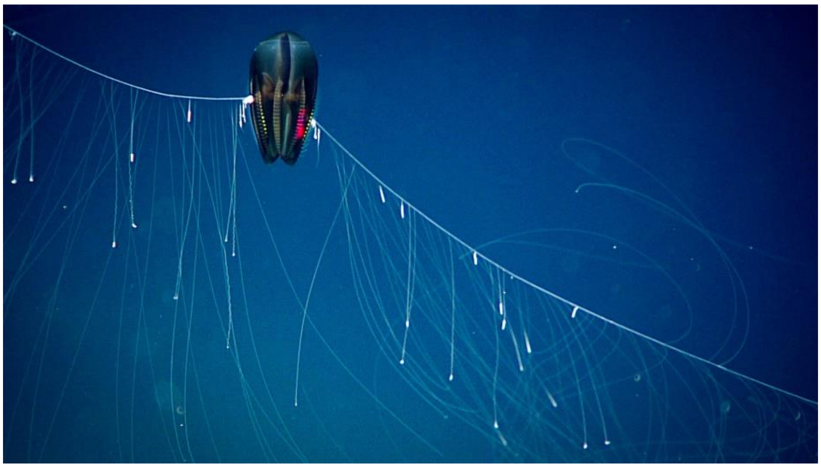

Dark ctenophore with tentacles extended, Gulf of Mexico.
Credit: NOAA Office of Ocean Exploration and Research

Ecological drivers: No light penetrates from the ocean surface to bathypelagic waters. Oxygen concentrations are not limiting to aerobic respiration (mostly 3–7 mL.L-1) and are recharged through thermohaline circulation by cooling. Oxygenated water is circulated globally from two zones (the Weddell Sea and the far North Atlantic Ocean) where ice formation and surface cooling create high-salinity, oxygenated water that sinks and is subsequently circulated globally via the 'great ocean conveyor'. Re-oxygenation frequency varies from 300 to 1,000 years, depending on the circulation route. More local thermohaline circulation occurs by evaporation in the Mediterranean and Red Seas, resulting in warm temperatures (13–15°C) at great depths. Otherwise, bathypelagic temperatures vary from −1°C in polar waters to 2–4°C in tropical and temperate waters. Nutrient levels are low and derive from the fall of organic remains from surface horizons. Pressure varies with depth from 100 to 300 atmospheres.

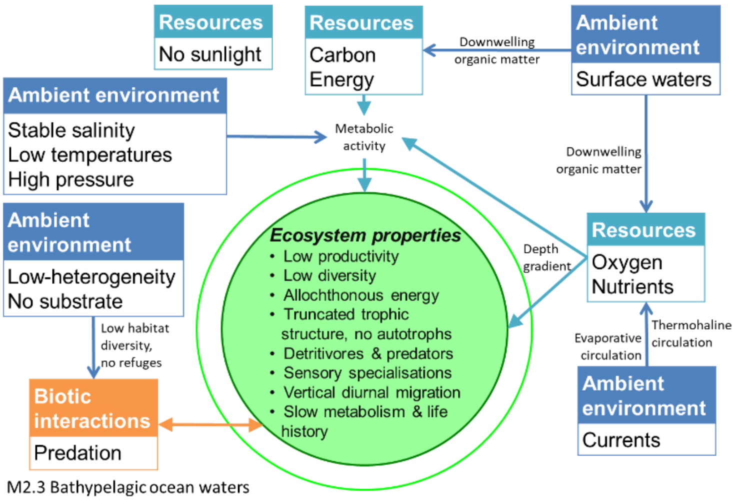

M2.3 Bathypelagic ocean waters

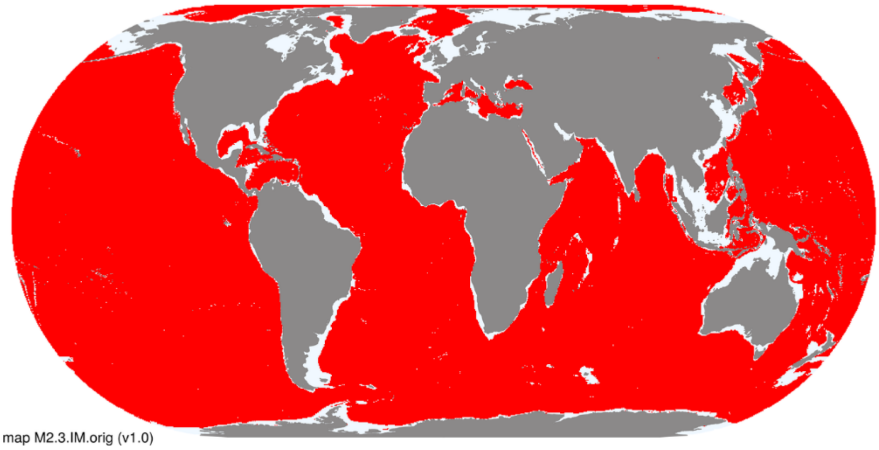

map M2.3.IM.orig (v1.0)

Distribution: All oceans and deep seas beyond the continental slope and within a depth range of 1,000 – 3,000 m.

References:

Priede, IG (2017) *Deep-sea fishes: Biology, diversity, ecology and fisheries* Chapter 1. Cambridge University Press, Cambridge.

Map is for illustrative purposes only and does not support spatial analyses unless formally validated.

Contributors: C Linardich, DA Keith

M2.4 Abyssopelagic ocean waters

Ecosystem properties: These deep, open-ocean ecosystems span depths from 3,000 to 6,000 m. Autotrophs are absent and energy sources are entirely allochthonous. Particulate organic debris is imported principally from epipelagic horizons (M2.1) and the flux of matter diminishing through the mesopelagic zone (M2.2) and bathypelagic zone (M2.3). Food for heterotrophs is therefore very scarce. Due to extreme conditions and limited resources, biodiversity is very low. Total biomass declines exponentially from an average of 0.16 mgC m-3 at 3,000 m in depth to 0.0058 mgC m-3 at 6,000 m. However, there is an order of magnitude variation around the mean due to regional differences in the productivity of surface waters. Truncated trophic networks are dominated by planktonic detritivores, with low densities of gelatinous invertebrates and scavenging and predatory fish. Fauna typically have low metabolic rates and some have internal light organs that produce bioluminescence to attract prey or mates or to defend themselves. Vertebrates typically have reduced skeletons and watery tissues to maintain buoyancy. Most of the biota possesses cell membranes with specialised phospholipid composition, intrinsic protein modifications, and protective osmolytes (i.e. organic compounds that influence the properties of biological fluids) to optimise protein function at high pressure.

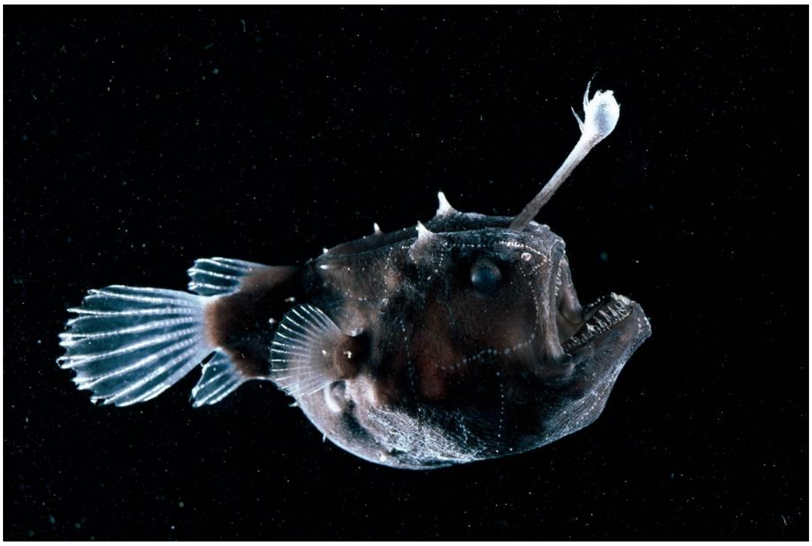

Deep sea Anglerfish (Himantolophus sp.) female with lure projecting from head to attract prey, Atlantic ocean.

Credit: Nature Picture Library / Alamy Stock

Ecological drivers: No light penetrates from the ocean surface to abyssopelagic waters. Nutrient concentrations are very low and recharge is dependent on organic flux and detrital fall from the epipelagic zone. Oxygen concentrations, however, are not limiting to aerobic respiration (mostly 3–7mL.L-1) and are generally recharged through global thermohaline circulation driven by cooling in polar regions. Water temperatures vary from below zero in polar waters up to 3°C in parts of the Atlantic. Hydrostatic pressure is extremely high (300–600 atmospheres). Currents are weak, salinity is stable, and there is little spatial heterogeneity in the water column.

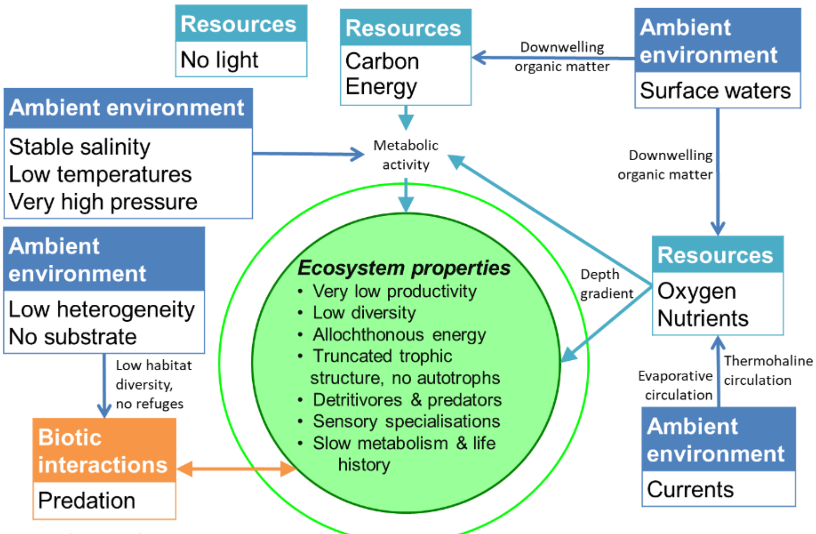

M2.4 Abyssopelagic ocean waters

Distribution: All oceans and the deepest parts of the Mediterranean Sea beyond the continental slope, mid-ocean ridges, and plateaus at depths of 3,000–6,000 m.

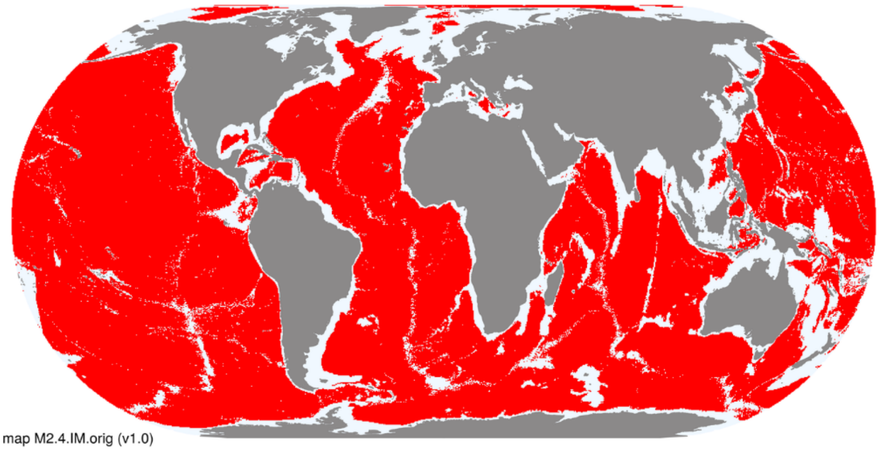

map M2.4.IM.orig (v1.0)

References:

Priede, IG (2017) *Deep-sea fishes: Biology, diversity, ecology and fisheries* Chapter 1. Cambridge University Press, Cambridge.

Map is for illustrative purposes only and does not support spatial analyses unless formally validated.

M2.5 Sea ice

Ecosystem properties: The seasonally frozen surface of polar oceans (1–2 m thick in the Antarctic and 2–3 m thick in the Arctic) may be connected to land or permanent ice shelves and is one of the most dynamic ecosystems on earth. Sympagic (i.e. ice-associated) organisms occur in all physical components of the sea-ice system including the surface, the internal matrix and brine channel system, the underside, and nearby waters modified by sea-ice presence. Primary production by microalgal and microbial communities beneath and within sea ice form the base of the food web and waters beneath sea ice develop. The standing stocks produced by these microbes are significantly greater than in ice-free areas despite shading by ice and are grazed by diverse zooplankton including krill. The sea ice underside provides refuge from surface predators and is an important nursery for juvenile krill and fish. Deepwater fish migrate vertically to feed on zooplankton beneath the sea ice. High secondary production (particularly of krill) in sea ice and around its edges supports seals, seabirds, penguins (in the Antarctic), and baleen whales. The highest trophic levels include vertebrate predators such as polar bears (in the Arctic), leopard seals, and toothed whales. Sea ice also provides resting and/or breeding habitats for pinnipeds (seals), polar bears, and penguins. As the sea ice decays annually, it releases biogenic material consumed by grazers and particulate and dissolved organic matter, nutrients, freshwater and iron, which stimulate phytoplankton growth and have important roles in biogeochemical cycling.

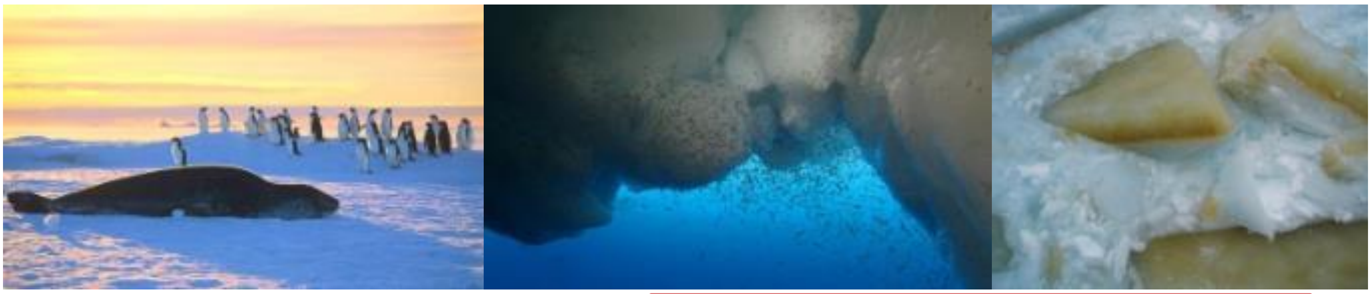

From left to right: Leopard seal on ice floe. Krill aggregation under sea ice. Sea-ice microbes and algae within the pack-ice substratum. Antarctica
Credit: Antarctic Climate and Ecosystems Cooperative Research Centre

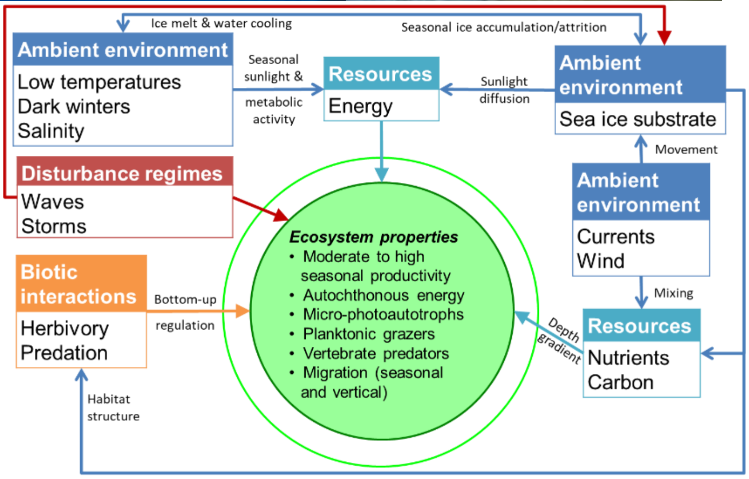

M2.5 Sea ice

Ecological drivers: Sea ice is integral to the global climate system and has a crucial influence on pelagic marine ecosystems and biogeochemical processes. Sea ice limits atmosphere-ocean gas and momentum exchanges, regulates sea temperature, reflects solar radiation, acquires snow cover, and redistributes freshwater to lower latitudes. The annual retreat of sea ice during spring and summer initiates high phytoplankton productivity at the marginal ice zone and provides a major resource for grazing zooplankton, including krill. Polynyas, where areas of low ice concentration are bounded by high ice concentrations, have very high productivity levels. Most sea ice is pack-ice transported by wind and currents. Fast ice forms a stationary substrate anchored to the coast, icebergs, glaciers, and ice shelves and can persist for decades.

Distribution: Arctic Ocean 0–45°N (Japan) or only to 80°N (Spitsbergen). Southern Ocean 55–70°S. At maximum extent, sea ice covers ~5% of the Northern Hemisphere and 8% of the Southern Hemisphere.

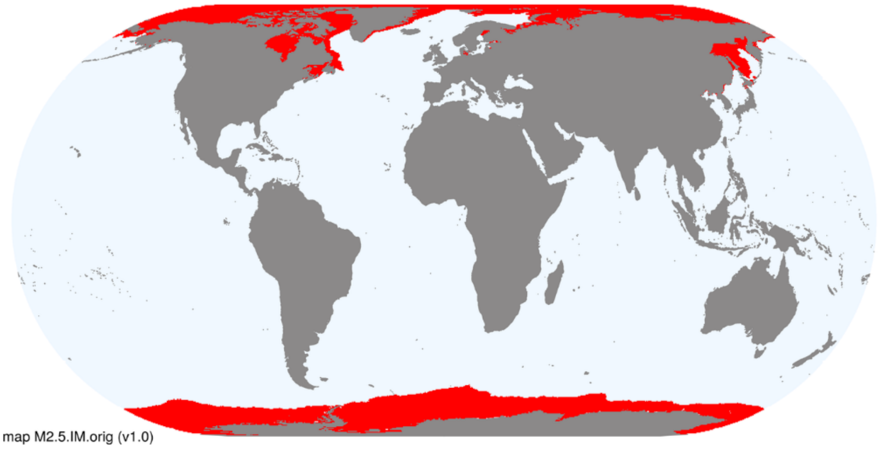
map M2.5.IM.orig (v1.0)

References:

Arrigo KR, Thomas DN (2004) Large scale importance of sea ice biology in the Southern Ocean. *Antarctic Science* 16: 471-486.

Brierley AS, Thomas DN (2002) Ecology of Southern Ocean pack ice. *Advances in Marine Biology* 43: 171-276. Academic Press.

Horner R., Ackley, SF, Dieckmann GS, Gulliksen B, Hoshiai T, Legendre L, Melnikov IA, Reeburgh WS, Spindler M, Sullivan CW (1992) Ecology of sea ice biota. *Polar Biology* 12: 417-427.

Map is for illustrative purposes only and does not support spatial analyses unless formally validated.

M3. Deep sea floors biome

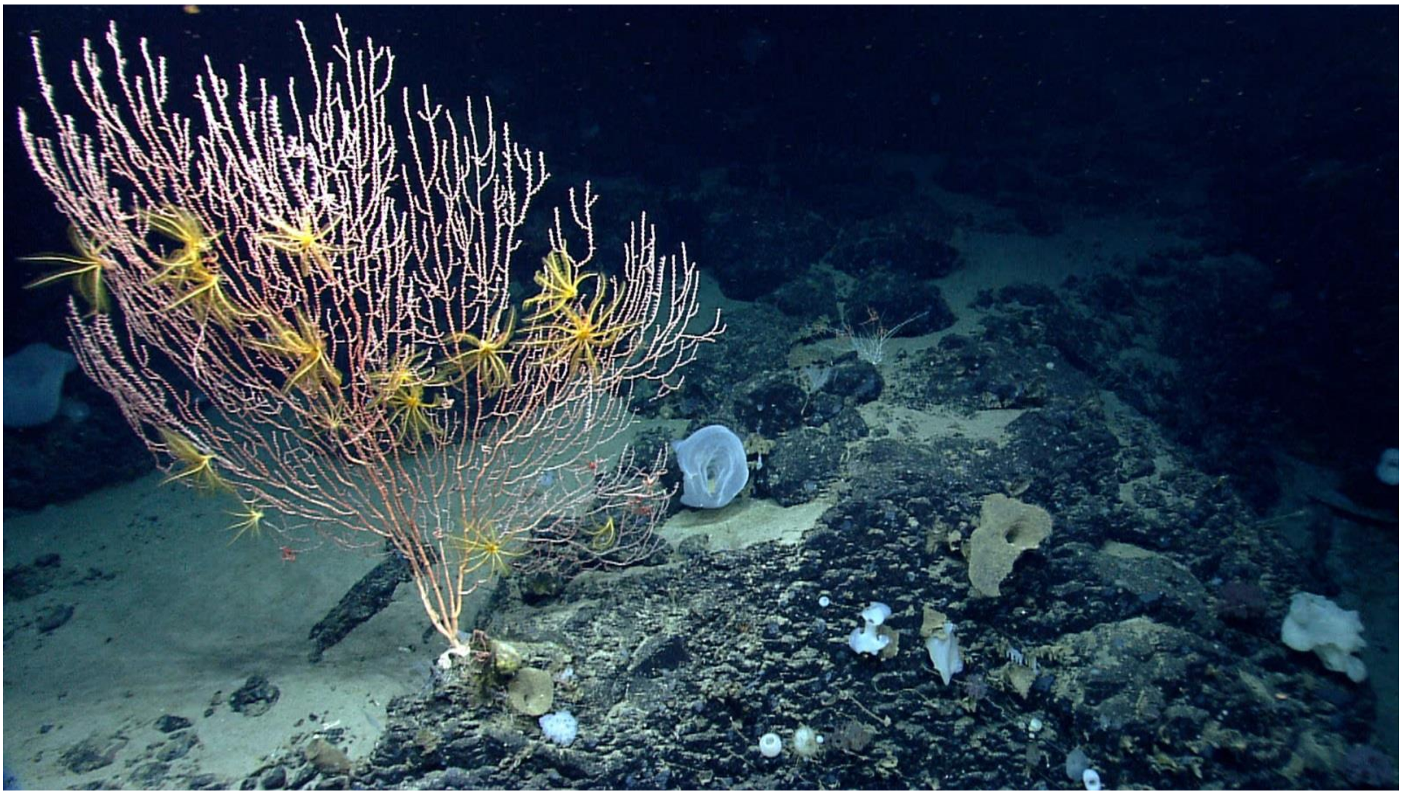

Colony of Jasonisis, a bamboo coral, with numerous crinoid associates, Mytilus seamount.

Credit: NOAA Ocean Explorer

The Deep-sea floor covers the entire oceanic benthos below ~250 m depth, where there is not enough light to support primary productivity through photosynthesis. It extends from the upper bathyal seafloor to the deepest parts of the ocean, at just under 11 km in the Mariana Trench.

Most deep-sea communities are therefore heterotrophic, depending ultimately on allochthonous energy and nutrients from the vertical flux and/or advection down-slopes of organic matter produced in the upper photic layers of ocean waters. Chemosynthetically-based ecosystems, such as those found at hydrothermal vents and cold seeps, are an exception, as chemoautotrophic microorganisms synthesise reduced compounds (e.g. hydrogen sulphide and methane) in hydrothermal and cold-seep fluids as autochthonous source of energy.

Oxygen is not limiting due to global-scale thermohaline circulation via deep ocean currents, except in bathyal (200–1,000 m) areas along the eastern Pacific, southwestern Africa, the Arabian Sea, and the Bay of Bengal, where physical and biological processes result in the formation of oxygen-minimum zones. Depth generates a strong gradient in hydrostatic pressure, increasing 1 atmosphere with every 10 m in depth, excluding fish from depths >8.5 km. Currents, geomorphology, and substrate type also influence ecosystem function.

Geomorphology differentiates several functional groups of ecosystems within the deep seafloor biome because of its influence on both the movement of currents and the vertical flux of resources, with marine canyons, seamounts, and trenches creating resource-rich hotspots. Extensive soft sediments on the abyssal plains support burrowing detritivores and predators, whereas sessile suspension feeders dominate hard substrates. Deep-sea benthic biodiversity is usually very high and mostly composed of meio-fauna and macro-fauna, with high abundances of microbes. Chemosynthetically-based ecosystems are exceptional again, as their biota is characterised by high biomass, low diversity, and high endemism. Organisms are equipped with traits that enable survival in the absence of light, high hydrostatic pressure, and low levels of nutrients and carbon.

M3.1 Continental and island slopes

Ecosystem properties: These aphotic heterotrophic ecosystems fringe the margins of continental plates and islands, extending from the shelf break (~250 m depth) to the abyssal basins (4,000 m). These large sedimentary slopes with localised rocky outcrops are characterised by strong depth gradients in the biota and may be juxtaposed with specialised ecosystems such as submarine canyons (M3.2), deep-water biogenic systems (M3.6), and chemosynthetic seeps (M3.7), as well as landslides and oxygen-minimum zones. Energy sources are derived mostly from lateral advection from the shelf and vertical fallout of organic matter particles through the water column and pelagic fauna impinging on the slopes, which varies seasonally with the productivity of the euphotic layers. Other inputs of organic matter include sporadic pulses of large falls (e.g. whale falls and wood falls). Photoautotrophs and resident herbivores are absent and the trophic network is dominated by microbial decomposers, detritivores, and their predators. Depth-related gradients result in a marked bathymetric zonation of faunal communities, and there is significant basin-scale endemism in many taxa. The taxonomic diversity of these heterotrophs is high and reaches a maximum at middle to lower depths. The biomass of megafauna decreases with depth and the meio-fauna and macro-fauna become relatively more important, but maximum biomass occurs on mid-slopes in some regions. The megafauna is often characterised by sparse populations of detritivores, including echinoderms, crustaceans, and demersal fish, but sessile benthic organisms are scarce and the bottom is typically bare, unconsolidated sediments.

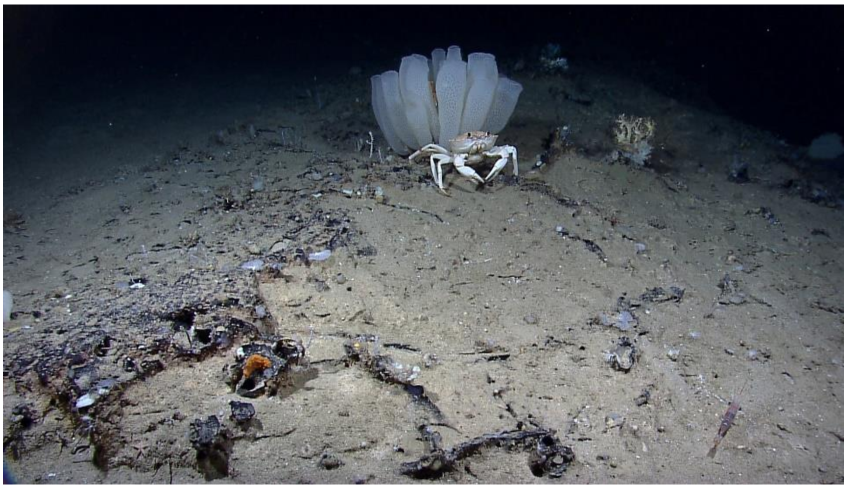

Golden crab and group of Venus flower basket glass sponges Gulf of Mexico, USA.
Credit: NOAA Office of Ocean Exploration and Research

Ecological drivers: The continental slopes are characterised by strong environmental depth gradients in pressure, temperature, light, and food. Limited sunlight penetration permits some visual predation but no photosynthesis below 250 m and rapidly diminishes with depth, with total darkness (excluding bioluminescence) below 1,000 m. Hydrostatic pressure increases with depth (1 atmosphere every 10 m). Temperature drastically shifts below the thermocline from warmer surface waters to cold, deep water (1–3°C), except in the Mediterranean Sea (13°C) and the Red Sea (21°C). Food quantity and quality decrease with increasing depth, as heterotrophic zooplankton efficiently use the labile compounds of the descending particulate organic matter. Sediments on continental slopes provide important ecosystem services, including nutrient regeneration and carbon sequestration.

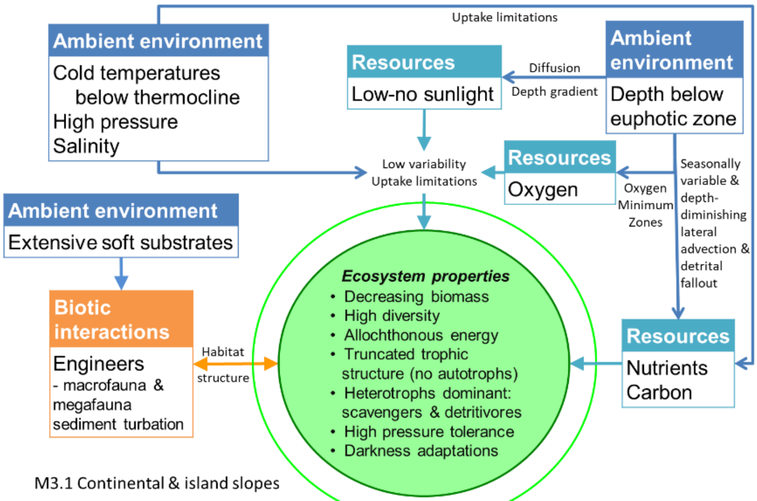

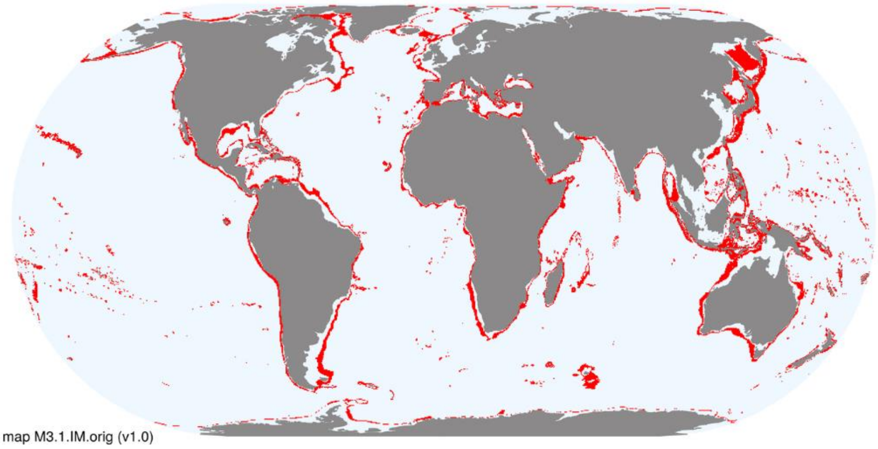

Distribution: Fringing the margins of all ocean basins and oceanic islands. Extending beneath 11% of the ocean surface at depths of 250–4,000 m.

References:

Menot L, Sibuet M, Carney RS, Levin LA, Rowe GT, Billett DSM et al. (2010) New perceptions of continental margin biodiversity. *Life in the world's oceans: diversity, distribution and abundance* Chapter 5, pp 79-101. Blackwell, Chichester.

Map is for illustrative purposes only and does not support spatial analyses unless formally validated.

M3.2 Submarine canyons

Ecosystem properties: Submarine canyons are major geomorphic features that function as dynamic flux routes for resources between continental shelves and ocean basins. As a result, canyons are one of the most productive and biodiverse habitats in the deep sea. Habitat heterogeneity and temporal variability are key features of submarine canyons, with the diversity of topographic and hydrodynamic features and substrate types (e.g. mud, sand, and rocky walls) within and among canyons contributing to their highly diverse heterotrophic faunal assemblages. Photoautotrophs are present only at the heads of some canyons. Canyons are characterised by meio-, macro-, and mega-fauna assemblages with greater abundances and/or biomass than adjacent continental slopes (M3.1) due mainly to the greater quality and quantity of food inside canyon systems. Habitat complexity and high resource availability make canyons important refuges, nurseries, spawning areas, and regional source populations for fish, crustaceans, and other benthic biota. Steep exposed rock and strong currents may facilitate the development of dense communities of sessile predators and filter-feeders such as cold-water corals and sponges, engineering complex three-dimensional habitats. Soft substrates favour high densities of pennatulids and detritivores such as echinoderms. The role of canyons as centres of carbon deposition makes them an extraordinary habitat for deep-sea deposit-feeders, which represent the dominant mobile benthic trophic guild. The high productivity attracts pelagic-associated secondary and tertiary consumers, including cetaceans, which may visit canyons for feeding and breeding.

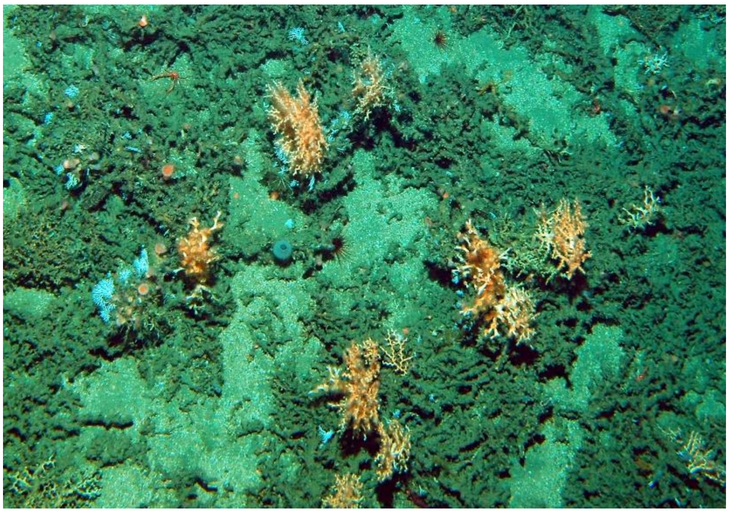

Wall of La Gaviera canyon, with cold-water corals, sponges and anemones, ~850 m depth in the Cantabrian Sea.
Credit: Francisco Sanchez, IEO

Ecological drivers: Submarine canyons vary in their origin, length, depth range (mean: 2,000 m), hydrodynamics, sedimentation patterns, and biota. Their complex topography modifies regional currents, inducing local upwelling, downwelling, and other complex hydrodynamic processes (e.g. turbidity currents, dense shelf water cascading, and internal waves). Through these processes, canyons act as geomorphic conduits of water masses, sediments, and organic matter from the productive coastal shelf to deep basins. This is particularly evident in shelf-incising canyons directly affected by riverine inputs and other coastal processes. Complex hydrodynamic patterns enhance nutrient levels and food inputs mostly through downward lateral advection but also by local upwelling, active biological transport by vertical migration of organisms, and passive fall of organic flux of varied particles sizes. Differences among canyons are driven primarily by variation in the abundance and quality of food sources, as well as finer-scale drivers including the variability of water mass structure (i.e. turbidity, temperature, salinity, and oxygen gradients), seabed geomorphology, depth, and substratum.

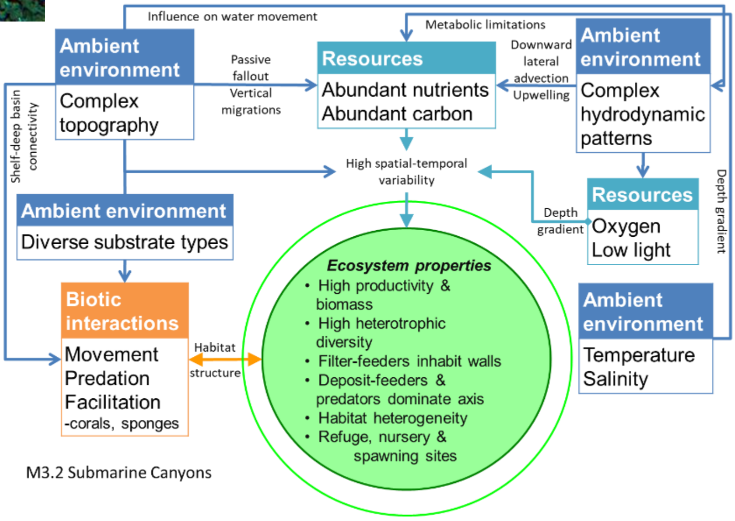

Distribution: Submarine canyons cover 11.2% of continental slopes, with 9,000 large canyons recorded globally. Most of their extent is distributed below 200 m, with a mean depth of 2,000 m.

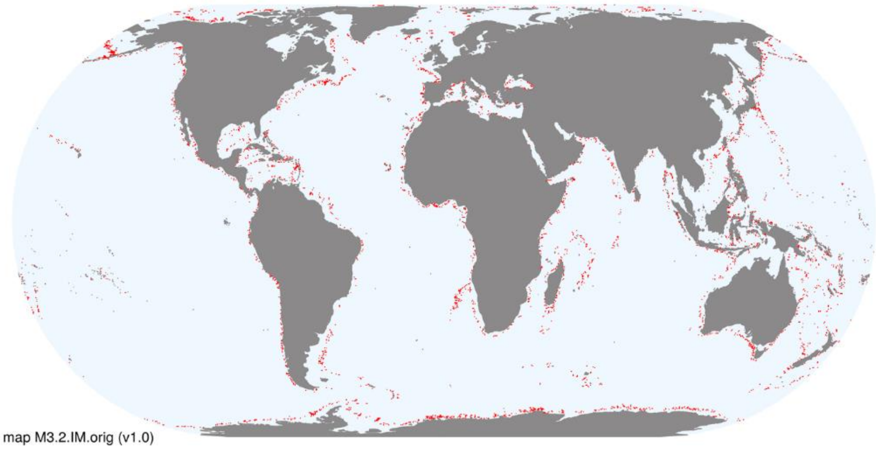

References:

Fernandez-Arcaya U, Ramirez-Llodra E, Aguzzi J, Allcock AL, Davies JS, Dissanayake A, Harris P, Howell K, Huvenne VA, Macmillan-Lawler M, Martín J (2017) Ecological role of submarine canyons and need for canyon conservation: a review. *Frontiers in Marine Science* 4: 5.

Map is for illustrative purposes only and does not support spatial analyses unless formally validated.

Contributors: E Ramirez-Llodra, IG Priede, DA Keith

M3.3 Abyssal plains

Ecosystem properties: This is the largest group of benthic marine ecosystems, extending between 3,000 and 6,000 m depth and covered by thick layers (up to thousands of metres) of fine sediment. Less than 1% of the seafloor has been investigated biologically. Tests of giant protozoans and the lebensspuren (i.e. tracks, borrows, and mounds) made by megafauna structure the habitats of smaller organisms. Ecosystem engineering aside, other biotic interactions among large fauna are weak due to the low densities of organisms. Abyssal communities are heterotrophic, with energy sources derived mostly from the fallout of organic matter particles through the water column. Large carrion falls are major local inputs of organic matter and can later become important chemosynthetic environments (M3.7). Seasonal variation in particulate organic matter flux reflects temporal patterns in the productivity of euphotic layers. Input of organic matter can also be through sporadic pulses of large falls (e.g. whale falls and wood falls). Most abyssal plains are food-limited and the quantity and quality of food input to the abyssal seafloor are strong drivers shaping the structure and function of abyssal communities. Abyssal biomass is very low and dominated by meio-fauna and microorganisms that play key roles in the function of benthic communities below 3,000 m depth. The abyssal biota, however, is highly diverse, mostly composed of macro- and meio-fauna with large numbers of species new to science (up to 80% in some regions). Many species have so far been sampled only as singletons (only one specimen per species) or as a few specimens. The megafauna is often characterised by sparse populations of detritivores, notably echinoderms, crustaceans, and demersal fish. Species distribution and major functions such as community respiration and bioturbation are linked to particulate organic carbon flux. These functions modulate the important ecosystem services provided by abyssal plains, including nutrient regeneration and carbon sequestration.

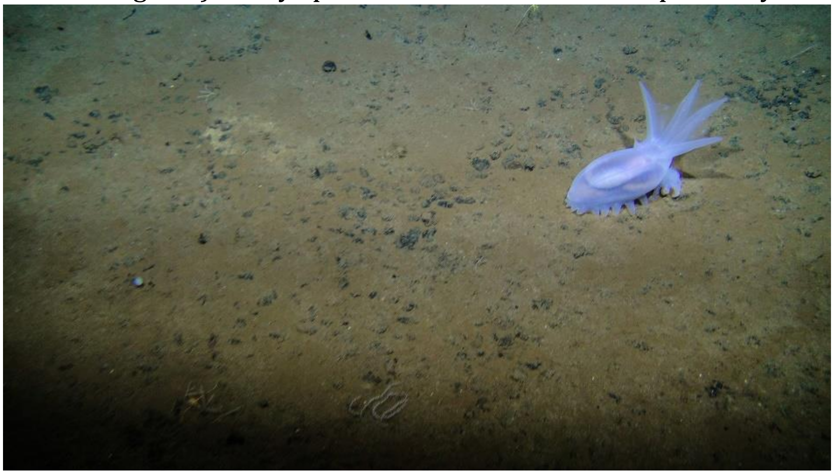

Sea cucumber Amperima sp. on the seabed in the eastern Clarion-Clipperton Fracture Zone.
Credit: Craig Smith and Diva Amon, NOAA

Ecological drivers: No light penetrates to abyssal depths. Hydrostatic pressure is very high (300–600 atmospheres). Water masses above abyssal plains are well oxygenated and characterised by low temperatures (−0.5–3°C), except in the Mediterranean Sea (13°C) and the Red Sea (21°C). The main driver of most abyssal communities is food, which mostly arrives to the seafloor as particulate organic carbon or 'marine snow'. Only 0.5–2% of the primary production in the euphotic zone reaches the abyssal seafloor, with the quantity decreasing with increasing depth.

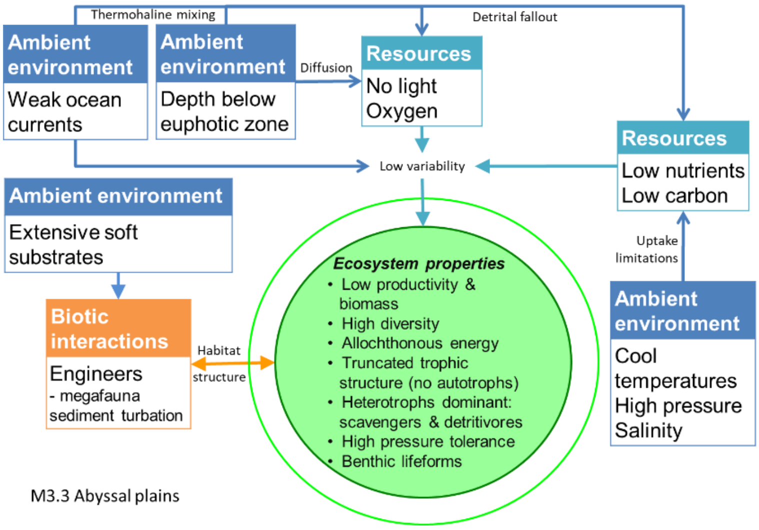

Distribution: Seafloor of all oceans between 3,000 and 6,000 m depth, accounting for 76% of the total seafloor area, segmented by mid-ocean ridges, island arcs, and trenches.

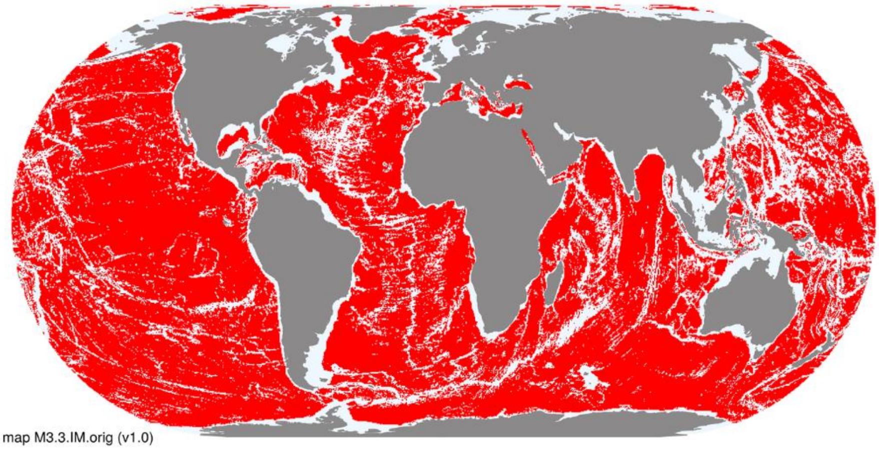

map M3.3.IM.orig (v1.0)

References:

Smith CR, De Leo FC, Bernardino AF, Sweetman AK, Martinez Arbizu P (2008) Abyssal food limitation, ecosystem structure and climate change. *Trends in Ecology and Evolution* 23: 518-528.

M3.4 Seamounts, ridges and plateaus

Ecosystem properties: Seamounts, plateaus, and ridges are major geomorphic features of the deep oceanic seafloor, characterised by hard substrates, elevated topography, and often higher productivity than surrounding waters. Topographically modified currents affect geochemical cycles, nutrient mixing processes, and detrital fallout from the euphotic zone that deliver allochthonous energy and nutrients to these heterotroph-dominated systems. Suspension-feeders and their dependents and predators dominate the trophic web, whereas deposit-feeders and mixed-feeders are less abundant than in other deep-sea systems. Autotrophs are generally absent. Summits that reach the euphotic zone are included within functional groups of the Marine shelf biome. Bathymetric gradients and local substrate heterogeneity support marked variation in diversity, composition, and abundance. Rocky walls, for example, may be dominated by sessile suspension-feeders including cnidarians (especially corals), sponges, crinoids, and ascidians. High densities of sessile animals may form deep-water biogenic beds (M3.5), but those systems are not limited to seamounts or ridges. Among the mobile benthic fauna, molluscs and echinoderms can be abundant. Seamounts also support dense aggregations of large fish, attracted by the high secondary productivity of lower trophic levels in the system, as well as spawning and/or nursery habitats. Elevated topography affects the distribution of both benthic and pelagic fauna. Seamounts and ridges tend to act both as stepping stones for the dispersal of slope-dwelling biota and as dispersal barriers between adjacent basins, while insular seamounts may have high endemism.

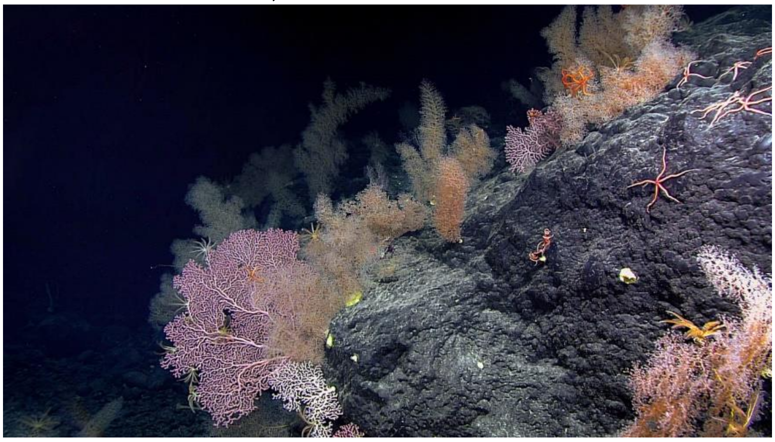

Garden of coral at depth 2,465 meters on the Sibelius Seamount.
Credit: NOAA Office of Ocean Exploration and Research

Ecological drivers: Seamounts, rising more than 1,000 m above the sediment-covered seabed, and smaller peaks, knobs, and hills are topographically isolated features, mostly of volcanic origin. Mid-ocean ridges are semi-continuous mountain chains that mark the spreading margins of adjacent tectonic plates. These prominent topographic formations interact with water masses and currents, increasing turbulence, mixing, particle retention, and the upward movement of nutrients from large areas of the seafloor. This enhances productivity on the seamounts and ridges themselves and also in the euphotic zone above, some of which returns to the system through detrital fallout. A diversity of topographic, bathymetric, and hydrodynamic features and substrate types (e.g. steep rocky walls, flat muddy areas, and biogenic habitats at varied depths) contribute to niche diversity and biodiversity. Major bathymetric clines associated with elevated topography produce gradients that shape ecological traits including species richness, community structure, abundance, biomass, and trophic modes.

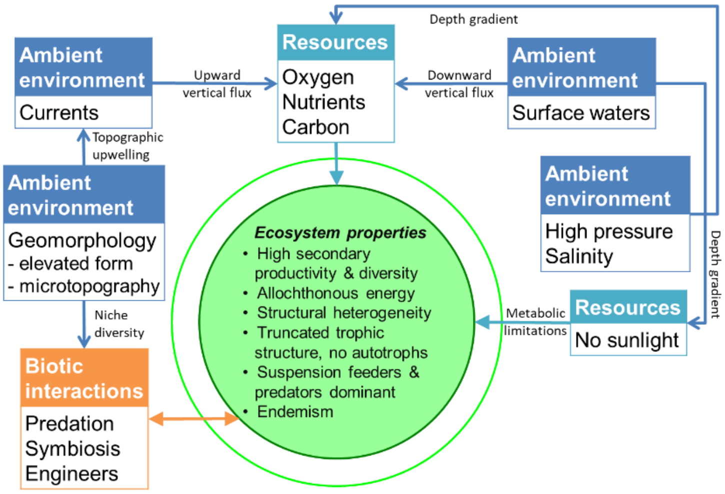

M3.4 Seamounts, ridges & plateaus

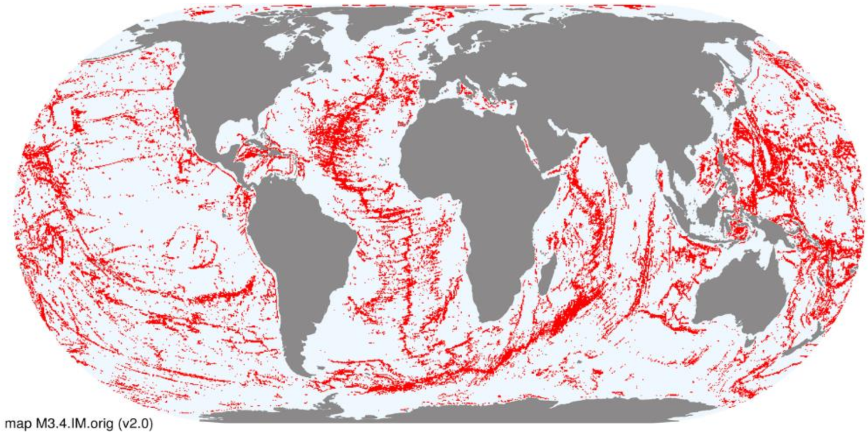

Distribution: About 171,000 seamounts, knolls, and hills documented worldwide so far, covering ~2.6% of the sea floor. Ridges cover ~9.2% of the sea floor along a semi-continuous, 55,000km long system.

References:

Rogers AD (2018) The Biology of Seamounts: 25 Years on. *Advances in Marine Biology* 79:137-224.

Schlacher A, Rowden AA, Dower JF, Consalvey M (2010) Recent advances in seamount ecology: A contribution to the Census of Marine Life. *Marine Ecology* (special issue) 31: 1-241.

Map is for illustrative purposes only and does not support spatial analyses unless formally validated.

M3.5 Deepwater biogenic beds

Ecosystem properties: Benthic, sessile suspension-feeders such as aphotic corals, sponges, and bivalves form structurally complex, three-dimensional structures or 'animal forests' in the deep oceans. In contrast to their shallow-water counterparts in coastal and shelf systems (M1.5), these ecosystems are aphotic and rely on allochthonous energy sources borne in currents and pelagic fallout. The trophic web is dominated by filter-feeders, decomposers, detritivores, and predators. Primary producers and associated herbivores are only present at the interface with the photic zone (~250 m depth). The biogenic structures are slow growing but critical to local demersal biota in engineering shelter from predators and currents, particularly in shallower, more dynamic waters. They also provide stable substrates and enhance food availability. This habitat heterogeneity becomes more important with depth as stable, complex elevated substrate becomes increasingly limited. These structures and the microenvironments within them support a high diversity of associated species including symbionts, microorganisms in coral biofilm, filter-feeding epifauna, biofilm grazers, mobile predators (e.g. polychaetes and crustaceans), and benthic demersal fish. Diversity is positively related to the size, flexibility, and structural complexity of habitat-forming organisms. Their impact on hydrography and the flow of local currents increases retention of particulate matter, zooplankton, eggs and larvae from the water column. This creates positive conditions for suspension-feeders, which engineer their environment and play important roles in benthic-pelagic coupling, increasing the flux of matter and energy from the water column to the benthic community.

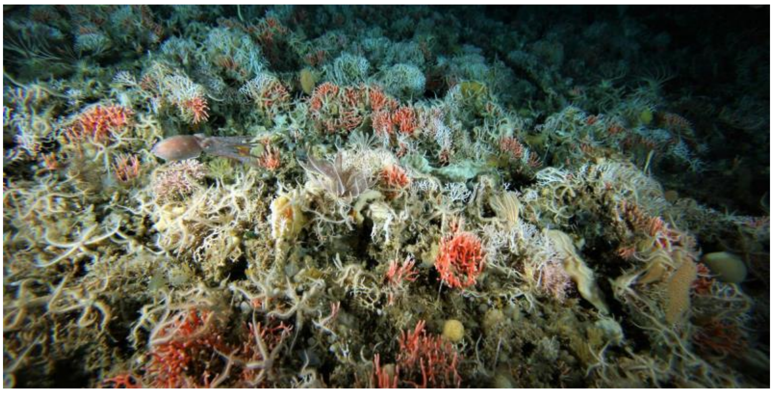

Corals and sponges on a deep Antarctic reef.
Credit: Australian Antarctic Division

Ecological drivers: The productivity of surface water, the vertical flux of nutrients, water temperature, and hydrography influence the availability of food, and hence the distribution and function of deep-water biogenic beds. Although these systems occur on both hard and soft substrates, the latter are less structurally complex and less diverse. Chemical processes are important and ocean acidity is limiting. The presence of cold-water corals, for example, has been linked to the depth of aragonite saturation. Habitat-forming species prefer regions characterised by oxygenation and currents or high flow, generally avoiding oxygen-minimum zones. Benthic biogenic structures and their dependents are highly dependent on low levels of physical disturbance due to slow growth rates and recovery times.

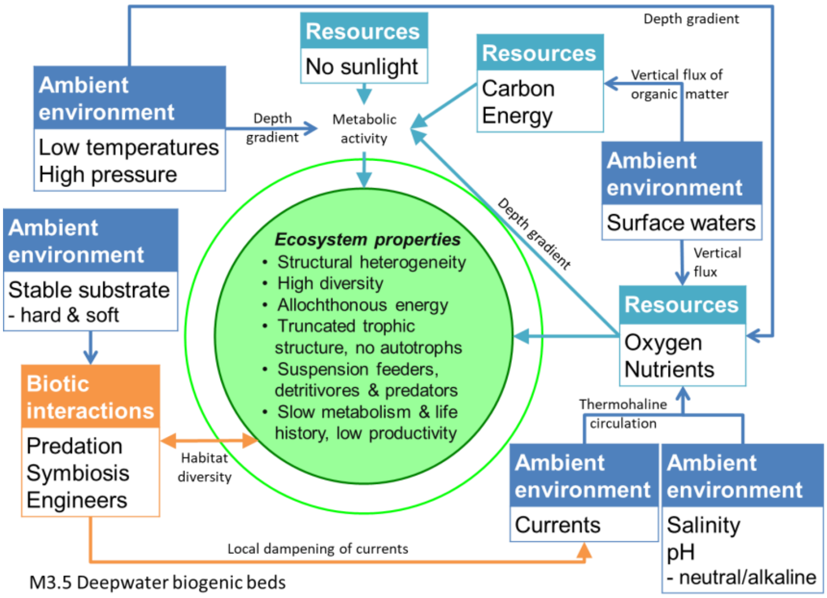

M3.5 Deepwater biogenic beds

Distribution: Patchy but widespread distribution across the deep sea floor below 250 m depth. Poorly explored and possibly less common on abyssal plains.

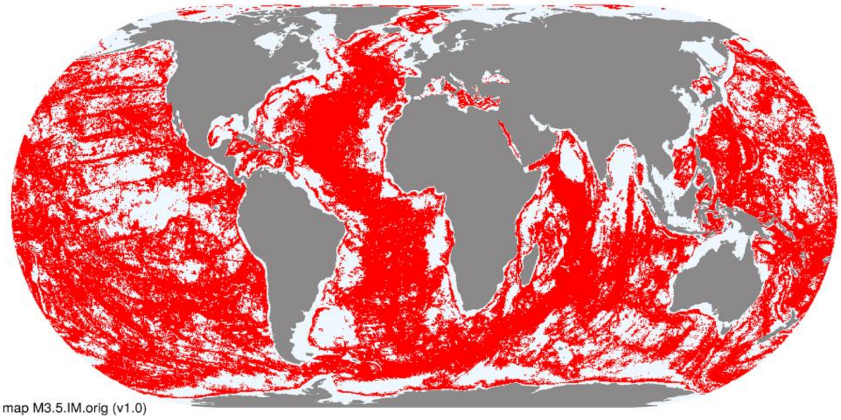

References:

Buhl-Mortensen L, Vanreusel A, Gooday AJ et al. (2010) Biological structures as a source of habitat heterogeneity and biodiversity on the deep ocean margins. *Marine Ecology* 31: 21-50.

Rossi S, Bramanti L, Gori, A, Orejas C (2017) *Marine Animal Forests: The ecology of benthic biodiversity Hotspots* Springer, Berlin.

Rossi S, Isla E, Bosch-Belmar M, Galli G, Gori A et al. (2019) Changes of energy fluxes in the marine animal forest of the Anthropocene: factors shaping the future seascape. *ICES Journal of Marine Sciences* 76, 2008-2019.

Map is for illustrative purposes only and does not support spatial analyses unless formally validated.

M3.6 Hadal trenches and troughs

Ecosystem properties: Hadal zones are the deepest ocean systems on earth and among the least explored. They are heterotrophic, with energy derived from the fallout of particulate organic matter through the water column, which varies seasonally and geographically and accumulates in the deepest axes of the trenches. Most organic matter reaching hadal depths is nutrient-poor because pelagic organisms use the labile compounds from the particulate organic matter during fallout. Hadal systems are therefore food-limited, but particulate organic matter flux may be boosted by sporadic pulses (e.g. whale falls and wood falls) and sediment transported by advection and seismically induced submarine landslides. Additional energy is contributed by chemosynthetic bacteria that can establish symbiotic relationships with specialised fauna. These are poorly known but more are expected to be discovered in the future. Hadal trophic networks are dominated by scavengers and detritivores, although predators (including through cannibalism) are also represented. Over 400 species are currently known from hadal ecosystems, with most metazoan taxa represented including amphipods, polychaetes, gastropods, bivalves, holothurians, and fish. These species possess physiological adaptations to high hydrostatic pressure, darkness, low temperature, and low food supply. These environmental filters, together with habitat isolation, result in high levels of endemism. Gigantism in amphipods, mysids, and isopods contrasts with the dwarfism in meio-fauna (e.g. nematodes, copepods, and kinorhynchs).

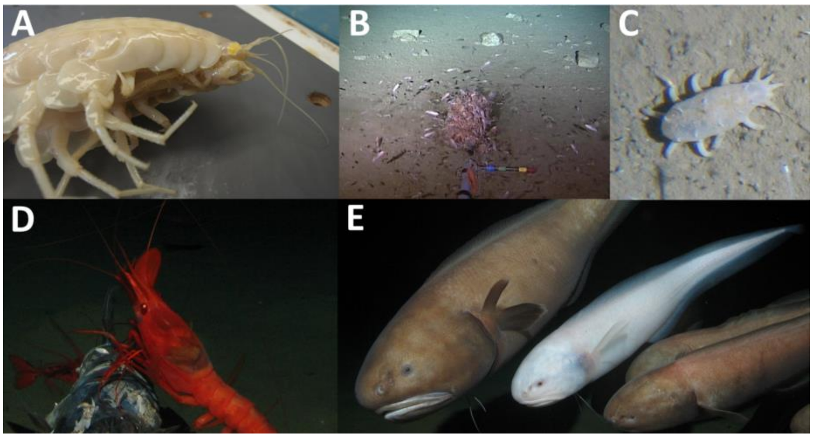

Typical hadal fauna: A) supergiant amphipod, B) dense clusters of scavenging amphipods, C) deposit-feeding holothurians, D) predatory decapods, and E) predatory fish.
Credit: Alan Jamieson

Ecological drivers: The hadal benthic zone extends from 6,000 to 11,000 m depth and includes 27 disjoint deep-ocean trenches, 13 troughs, and 7 faults. Sunlight is absent, nutrients and organic carbon are scarce, and hydrostatic pressure is extremely high (600–1,100 atmospheres). Water masses in trenches and troughs are well oxygenated by deep currents and experience constant, low temperatures (1.5–2.5°C). Rocky substrates outcrop on steep slopes of trenches and faults, while the floors comprise large accumulations of fine sediment deposited by mass movement, including drift and landslides, which are important sources of organic matter. Sediment, organic matter and pollutants tend to be "funnelled" and concentrated in the axis of the trenches.

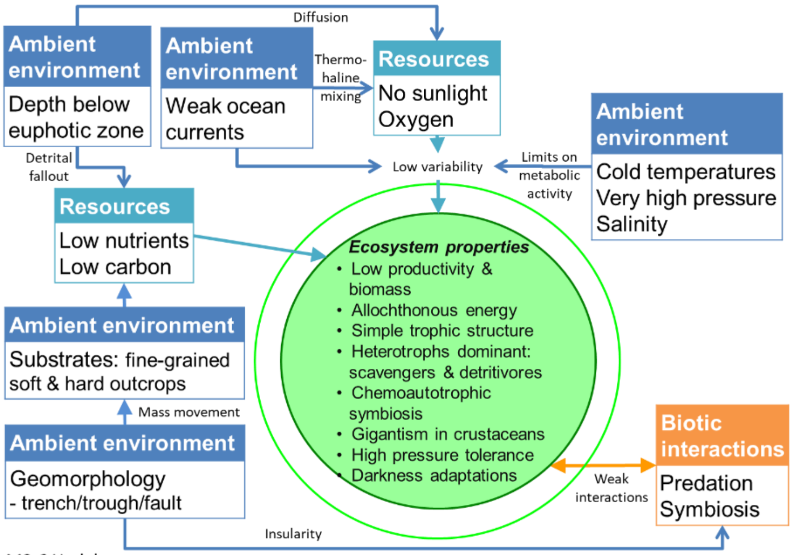

M3.6 Hadal zones

Distribution: A cluster of isolated trenches in subduction zones, faults, and troughs or basins, mostly in the Pacific Ocean, as well as the Indian and Southern Oceans, accounting for 1–2% of the total global benthic area.

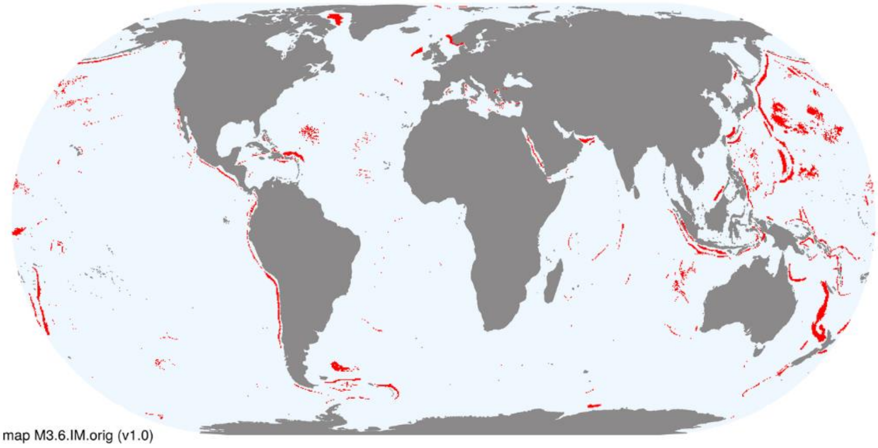

References:

Jamieson A, Fujii T, Mayor DJ, Solan M, Priede IG (2010) Hadal trenches: the ecology of the deepest places on Earth. *Trends in Ecology and Evolution* 25: 190-197.

Stewart HA, Jamieson AJ (2018) Habitat heterogeneity of hadal trenches: considerations and implications for future studies. *Progress in Oceanography* 161: 47-65.

Map is for illustrative purposes only and does not support spatial analyses unless formally validated.

M3.7 Chemosynthetic-based-ecosystems (CBE)

Ecosystem properties: Chemosynthetic-based ecosystems (CBEs) include three major types of habitats between bathyal and abyssal depths: 1) hydrothermal vents on mid-ocean ridges, back-arc basins, and active seamounts; 2) cold seeps on active and passive continental margins; and 3) large organic falls of whales or wood. All these systems are characterised by microbial primary productivity through chemoautotrophy, which uses reduced compounds (such as H2S and CH4) as energy sources instead of light. Microbes form bacterial mats and occur in trophic symbiosis with most megafauna. The continuous sources of energy and microbial symbiosis fuel high faunal biomass. However, specific environmental factors (e.g. high temperature gradients at vents, chemical toxicity, and symbiosis dependence) result in a low diversity and high endemism of highly specialised fauna. Habitat structure comprises hard substrate on vent chimneys and mostly biogenic substrate at seeps and food-falls. Most fauna is sessile or with low motility and depends on the fluids emanating at vents and seeps or chemicals produced by microbes on food-falls, and thus is spatially limited. Large tubeworms, shrimps, crabs, bivalves, and gastropods dominate many hydrothermal vents, with marked biogeographic provinces. Tubeworms, mussels, and decapod crustaceans often dominate cold seeps with demersal fish. These are patchy ecosystems where connectivity relies on the dispersal of planktonic larvae.

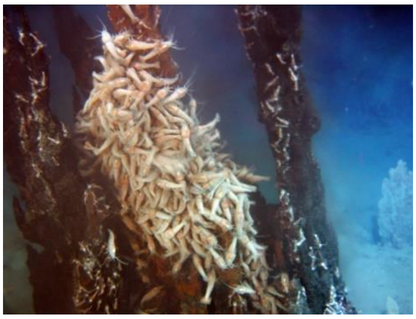

Shrimp on a hydrothermal vent chimney, Mid-Atlantic Ridge.
Credit: U. Azores

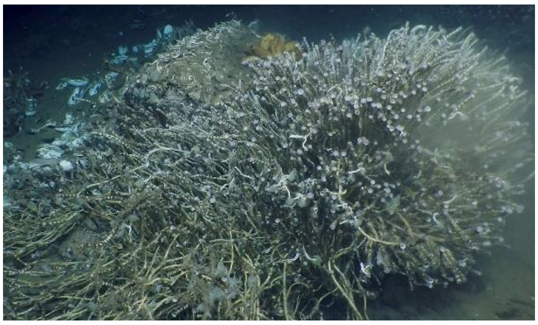

Cold seep with tubeworms, mussels and clams on the Pacific margin of Costa Rica.
Credit: Erik Cordes & ROCHITS program, WHOI

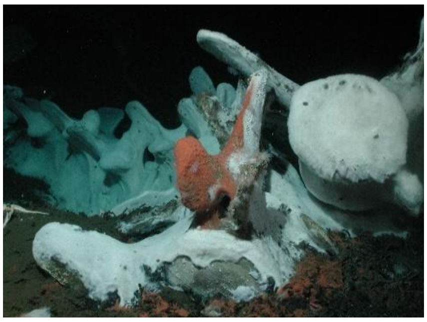

Whale fall.
Credit: Craig Smith, Uni. Hawaii, USA.

Ecological drivers: No light penetrates to deep-sea CBEs. Hydrostatic pressure is very high (30–600 atmospheres). At hydrothermal vents, very hot fluids (up to 400°C) emanate from chimneys charged with metals and chemicals that provide energy to chemoautotrophic microbes. At cold seeps, the fluids are cold and reduced chemicals originate both biogenically and abiotically. At food-falls, reduced chemicals are produced by microorganisms degrading the organic matter of the fall. The main drivers of CBEs are the chemosynthetically based primary productivity and the symbiotic relationships between microorganisms and fauna.

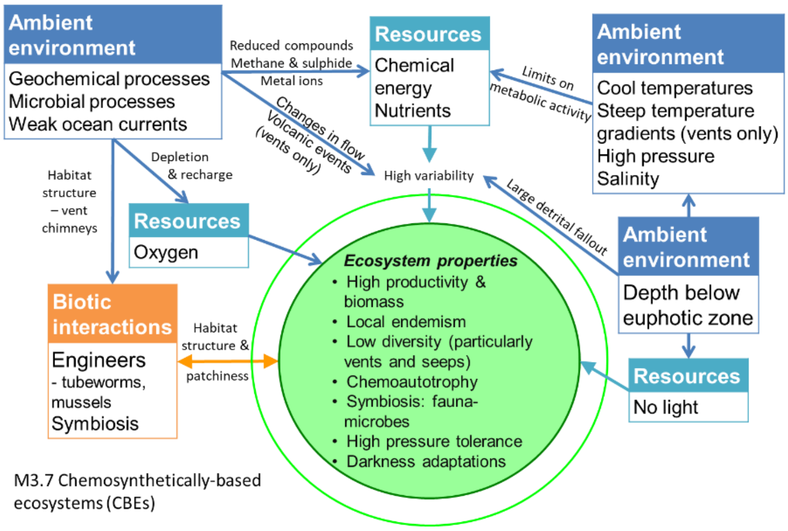

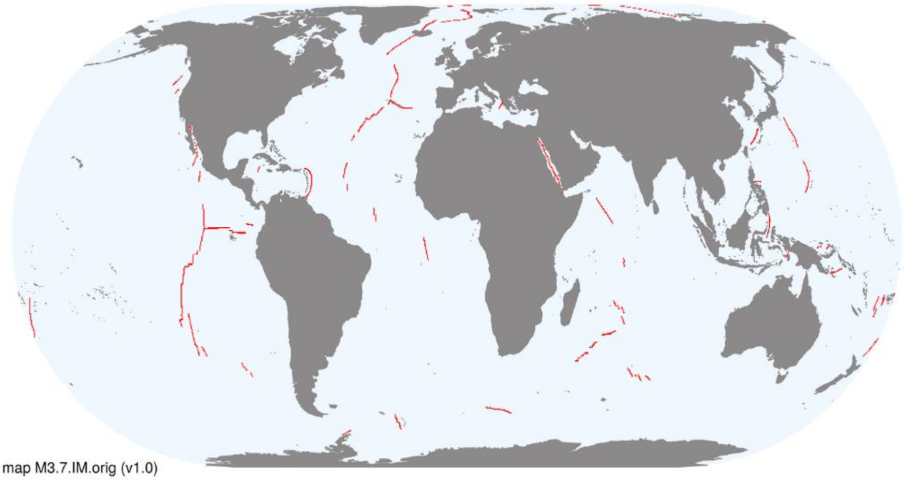

map M3.7.IM.orig (v1.0)

Distribution: Seafloor of all oceans. Vents (map) occur on mid-ocean ridges, back-arc basins, and active seamounts. Cold seeps occur on active and passive continental margins. Food-falls occur mostly along cetacean migration routes (whale falls).

References:

Tunnicliffe V, Juniper KS, Sibuet M (2003) Reducing environments of the deep-sea floor. *Ecosystems of the World* Vol 28 Ecosystems of the deep oceans, pp 81-110. Elsevier, London.

Map is for illustrative purposes only and does not support spatial analyses unless formally validated.

M4. Anthropogenic marine biome

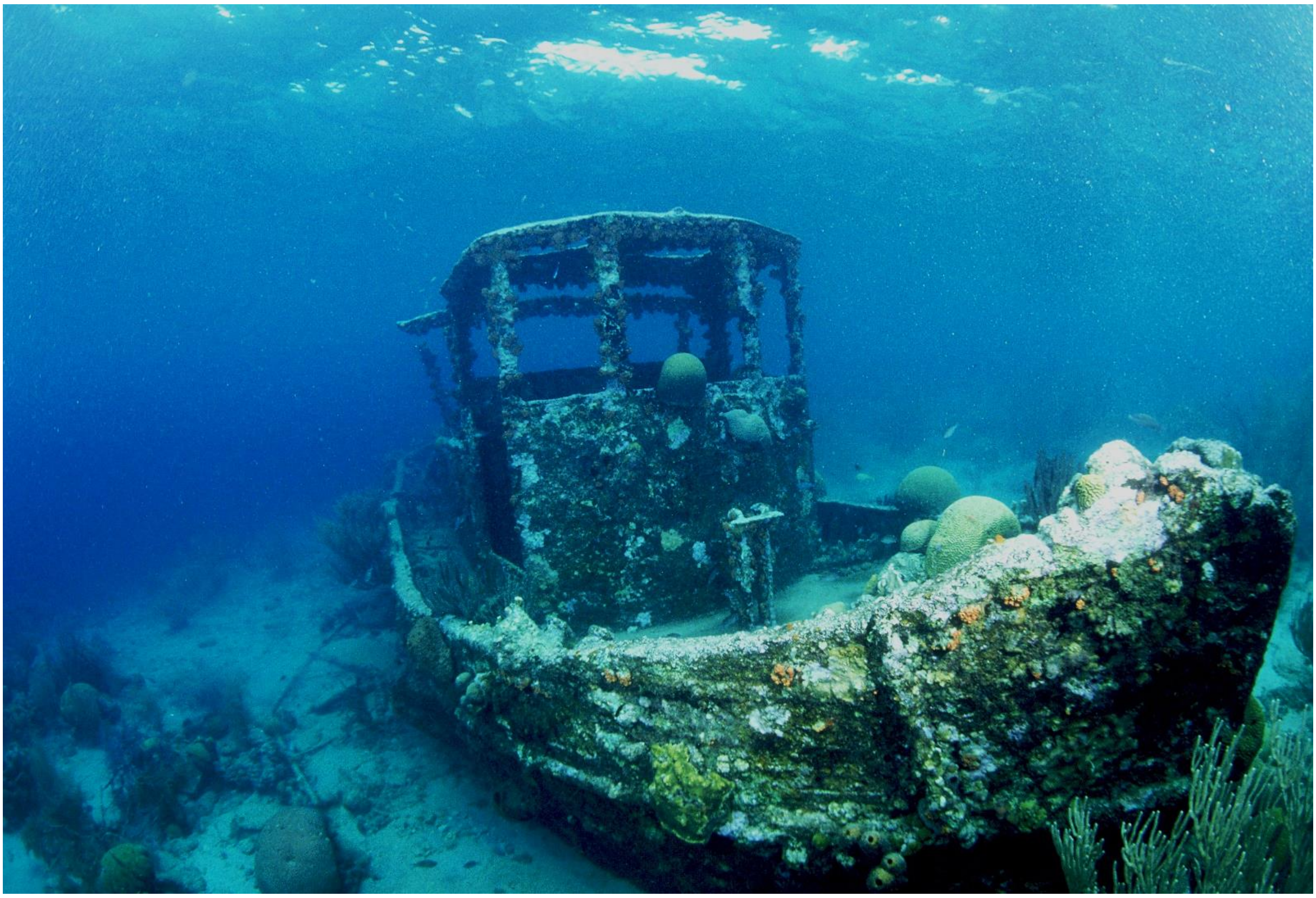

A wrecked tugboat encrusted with corals and barnacles in Caracas Bay, Curaçao Underwater Marine Park, Curaçao.

Credit: Corbis Documentary / Stuart Westmorland / Getty Images

Humans have constructed, deposited, or dumped artificial structures in the oceans that either confine managed marine organisms or attract marine biota that would not otherwise occupy such locations. These structures are distributed globally but are most common in regions of high-density occupation or transit. They include shipwrecks and mineral, gas, or energy infrastructure, pipelines, and rubble piles, as well as aquaculture infrastructure.

These installations provide an epibenthic substrate for sessile benthic organisms, as well as a demersal or pelagic environment for mobile organisms. Diversity and biomass of the epibenthic biofouling community is positively related to substrate rugosity. Most energy is supplied to these ecosystems from allochthonous sources, either passively via currents or actively through addition by humans (as is the case in aquaculture). Epibenthic and planktonic marine algae, however, make a contribution to the energy budget through local primary production.

Microbial decomposers and invertebrate detritivores in the sediments beneath and around the structures feed on particulate organic matter from the epibenthic biota (e.g. waste products and decaying bodies) or on unconsumed food delivered to managed species. The elevated productivity or visual features of artificial structures often attract larger pelagic predators, which forage in the vicinity.

Contributors: IM Suthers, JT Claisse, TP Crowe, DA Keith

M4.1 Submerged artificial structures

Ecosystem properties: These deployments include submerged structures with high vertical relief including ship wrecks, oil and gas infrastructure, and designed artificial reefs, as well as some low-relief structures (e.g. rubble piles). The latter do not differ greatly from adjacent natural reefs, but structures with high vertical relief are distinguished by an abundance of zooplanktivorous fish, as well as reef-associated fishes. Macroalgae are sparse or absent as the ecosystem is fed by currents and ocean swell delivering phytoplankton to sessile invertebrates. Complex surfaces quickly thicken with a biofouling community characterised by an abundance of filter-feeding invertebrates (e.g. sponges, barnacles, bivalves, and ascidians) and their predators (e.g. crabs and flatworms). Invertebrate diversity is high, with representatives from every living Phylum. Structures without complex surfaces, such as the smooth, wide expanse of a hull, may suffer the sporadic loss of all biofouling communities after storm events. This feeds the sandy bottom community, evident as a halo of benthic invertebrates (e.g. polychaetes and amphipods), which also benefit from the plume of waste and detritus drifting from the reef community. Artificial structures also provide a visual focus attracting the occasional pelagic fish and marine mammals, which respond similarly to fish-attraction devices and drift objects.

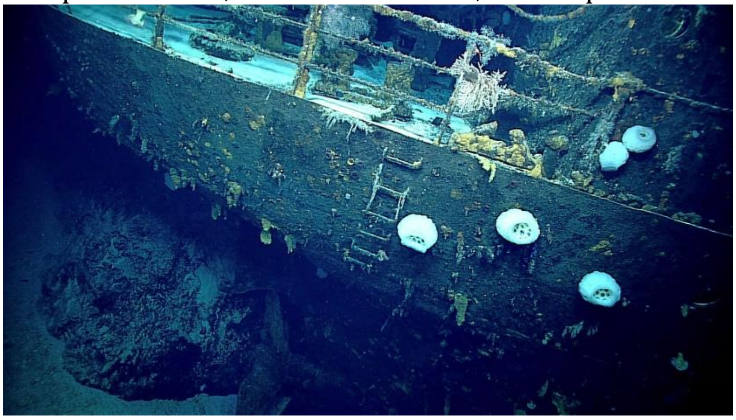

Wreck of the Amakasu Maru No.1, habitat for anemones, glass sponges, anglerfish, and other animals, Wake Atoll.
Credit: NOAA Office of Ocean Exploration & Research

Ecological drivers: The high vertical relief of many artificial structures enables biota to access plankton continuously transported by currents. They may be situated on otherwise flat, soft-bottom habitats, isolated to varying degrees from other hard substrates. High-energy waters experience low variation in temperature and salinity (except near major river systems). Currents and eddies cause strong horizontal flow, while ocean swell creates orbital current velocities at least 10-fold greater. Near large urban centres, fishing reduces populations of large predatory fish, resulting in a continuum across species and deployments from purely fish attraction to fish production (such as via the reef facilitating the planktivorous food chain). The historical, opportunistic use of materials (e.g. rubber tyres, construction materials, or inadequately decommissioned vessels) have left legacies of pollutants. Compared to artificial reefs, oil and gas infrastructure is more exposed to light/noise/chemical pollution associated with operations as well as the spread of invasive species.

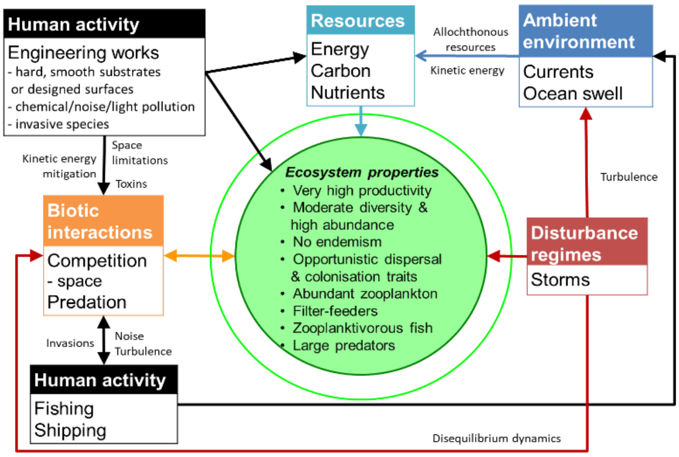

M4.1 Submerged artificial structures

Distribution: Millions of artificial reefs and fish-attraction devices are deployed in coastal waters worldwide, including >10,000 oil and gas structures, mostly in tropical and temperate waters. More than 500 oil and gas platforms were decommissioned and left as artificial reefs in US waters since 1940. Many others are candidates for reefing after decommissioning in coming decades (> 600 in the Asia-Pacific alone). Worldwide since 1984, over 130 ships and planes have purposely been sunk for recreational SCUBA-diving. Map is incomplete but shows areas with many documented wrecks and marine infrastructure.

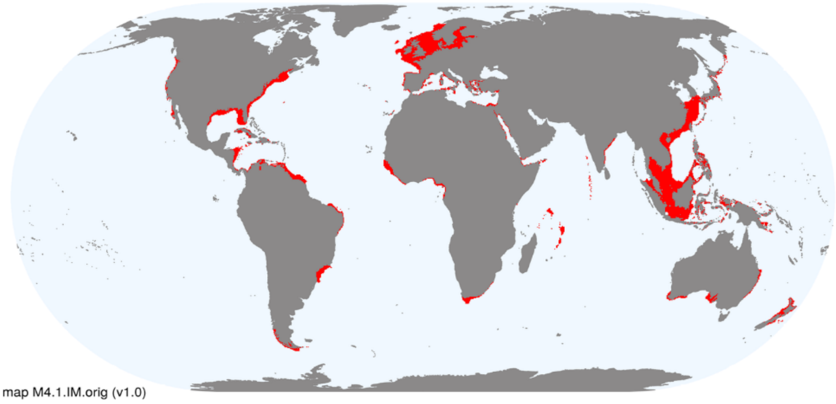
map M4.1.IM.orig (v1.0)

References:

Champion C, Suthers IM, Smith JA (2015) Zooplanktivory is a key process for fish production on a coastal artificial reef. *Marine Ecology Progress Series* 541:1-14.

Claisse JT, Pondella DJ, Love M, Zahn LA, Williams CM, Williams JP, Bull AS (2014) Oil platforms off California are among the most productive marine fish habitats globally. *Proc Natl Acad Sci. USA* 111:15462–15467.

Lima JS., Zalmo, R, Love M (2019) Overview and trends of ecological and socioeconomic research on artificial reefs. *Marine Environmental Research* 145, 81-96.

Scarborough Bull A, Love MS (2020) Worldwide oil and gas platform decommissioning: A review of practices and reefing options. *Ocean and Coastal Management* 168, 274–306.

M4.2 Marine aquafarms

Ecosystem properties: Marine aquafarms (i.e. mariculture) are localised, high-productivity systems within and around enclosures constructed for the breeding, rearing, and harvesting of marine plants and animals, including finfish, molluscs, crustaceans, algae, and other marine plants. Allochthonous energy and nutrient inputs are delivered by humans and by diffusion from surrounding marine waters. Autochthonous inputs are small and produced by pelagic algae or biofilms on the infrastructure, unless the target species are aquatic macrophytes. More commonly, target species are consumers that belong to middle or upper trophic levels. Diversity is low across taxa, and the trophic web is dominated by a super-abundance of target species. Where multiple target species are cultivated, they are selected to ensure neutral or mutualistic interactions with one another (e.g. detritivores that consume the waste of a higher-level consumer). Target biota are harvested periodically to produce food, fish meal, nutrient agar, horticultural products, jewellery, and cosmetics. Their high population densities are maintained by continual inputs of food and regular re-stocking to compensate harvest. Target species may be genetically modified and are often bred in intensive hatcheries and then released into the enclosures. Food and nutrient inputs may promote the abundance of non-target species including opportunistic microalgae, zooplankton, and pathogens and predators of the target species. These pest species or their impacts may be controlled by antibiotics or herbicides or by culling (e.g. pinnipeds around fish farms). The enclosures constitute barriers to the movement of larger organisms, but some cultivated stock may escape, while wild individuals from the surrounding waters may invade the enclosure. Enclosures are generally permeable to small organisms, propagules and waste products of larger organisms, nutrients, and pathogens, enabling the ecosystem to extend beyond the confines of the infrastructure.

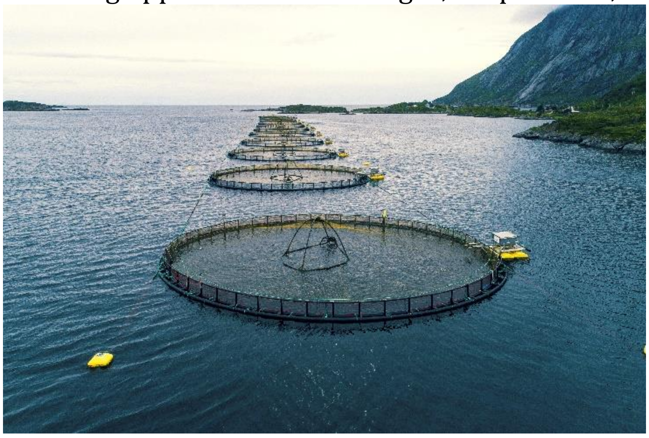

Mølnarodden Salmon fishery, Lofoten Islands, Norway.
Credit: Phillipe Turpin / Getty Images

Ecological drivers: Most marine farms are located in sheltered coastal waters but some are located in the open ocean or on land in tanks or ponds filled with seawater. Those in marine waters experience currents, tides, and flow-through of marine energy, matter, and biota characteristic of the surrounding environment. Those on land are more insular, with intensively controlled light and temperature, recirculation systems that filter and recycle water and waste, and intensive anthropogenic inputs of food and nutrients, anti-fouling chemicals, antibiotics, and herbicides. Marine enclosures have netting and frames that provide substrates for biofilms and a limited array of benthic organisms, but usually exclude the benthos. Land-based systems have smooth walls and floors that provide limited habitat heterogeneity for benthic biota.

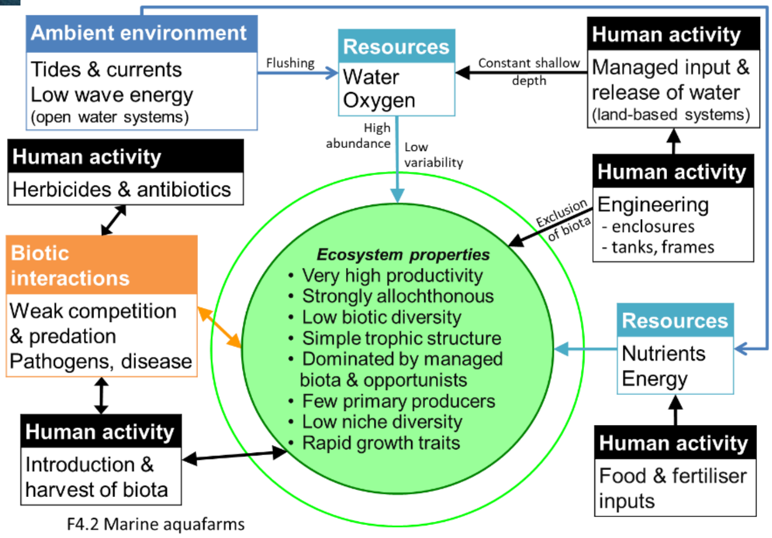

F4.2 Marine aquafarms

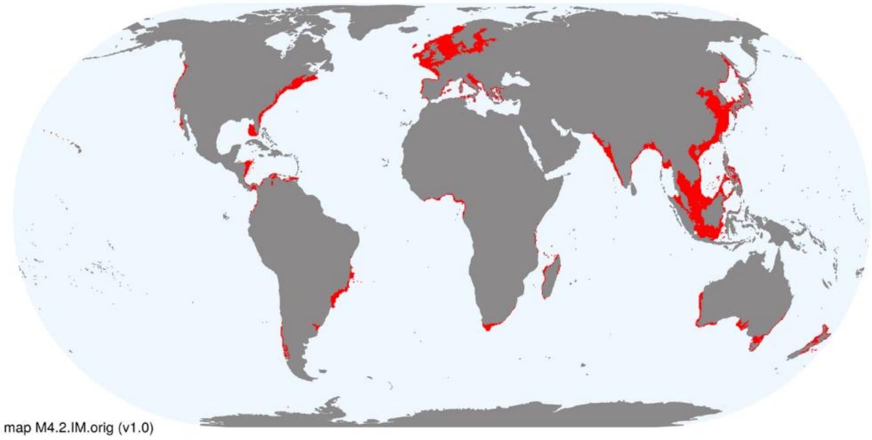

Distribution: Rapidly expanding around coastal Asia, Europe, North America and Mesoamerica, and southern temperate regions. Open-ocean facilities near Hawaii and Puerto Rico.

References:

Beveridge M (2008) *Cage Aquaculture* Wiley, Oxford.

Froehlich HE, Smith A, Gentry RR, Halpern BS (2017) Offshore aquaculture: I know it when I see it. *Frontiers in Marine Science* 4, 154.

Map is for illustrative purposes only and does not support spatial analyses unless formally validated.

MT1. Shorelines biome

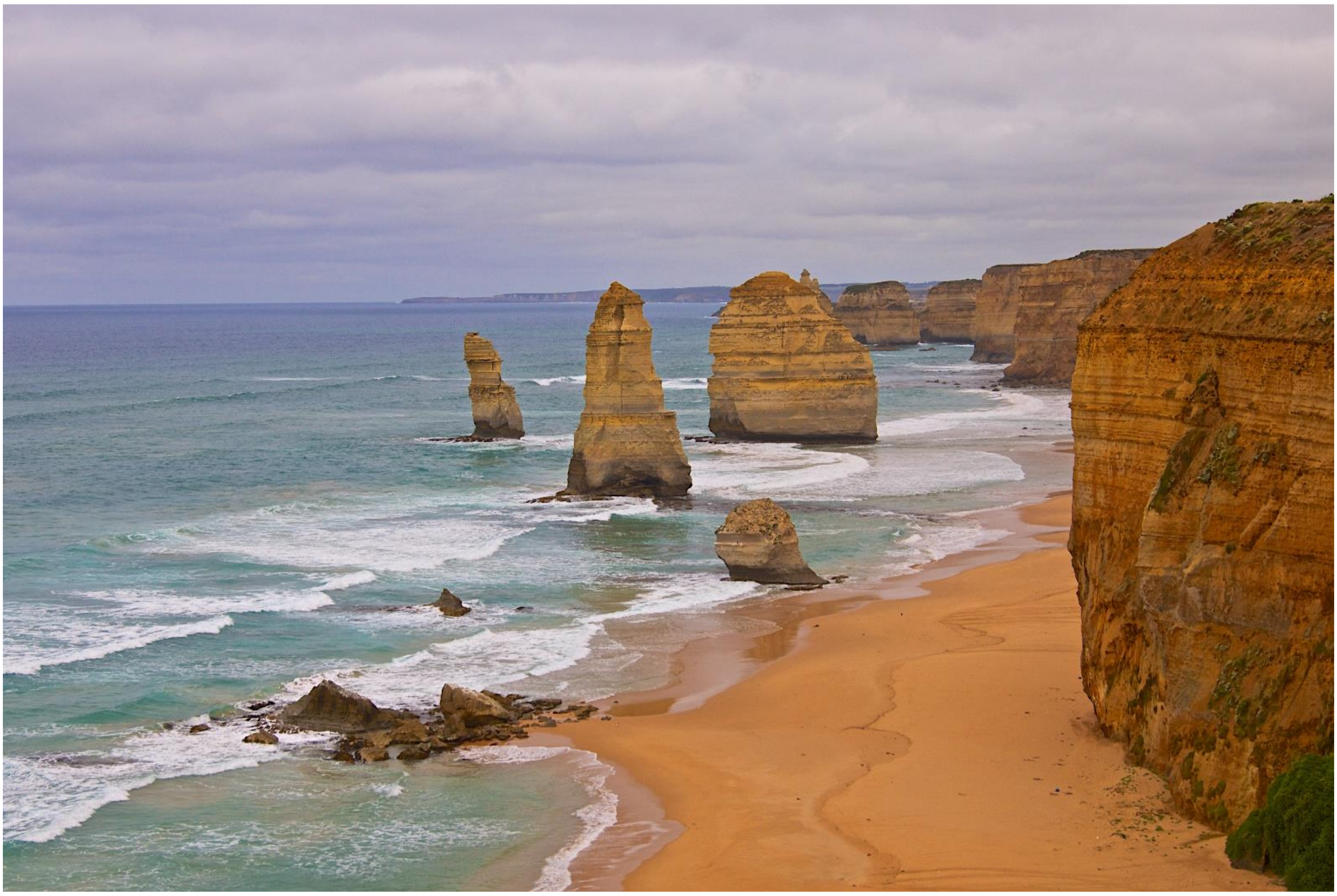

Twelve Apostles, Otway Coast, Victoria, Australia.

Credit: Hadi Zaher / Getty Images

The Shoreline systems biome comprises naturally formed, intertidal abiogenic habitats situated at the interface between land and sea. The distribution of the biome spans all latitudes (temperate to polar) at which landmasses are present.

Productivity ranges from high to low, is loosely proportional to the availability of stable hard substrate for macrophyte attachment, and is inversely proportional to the dependency on allochthonous energy sources derived from both land and sea. Productivity is also influenced by coastal upwelling, and for ecotypes of finer particle size, the nutrient content of adjacent terrestrial sediments.

Within and across ecotypes, biotic communities are strongly structured by tides, waves and particle size, ranging from contiguous rock to fine silts and clays. Tides produce a vertical gradient of increasing aerial exposure across which desiccation and temperature stress increase, time available for filter-feeding decreases, and interactions with marine and terrestrial predators vary. Waves and particle size determine substrate stability and the physical disturbance regime.

Wave action, diminishing from headlands to bays, produces horizontal gradients in community structure. Many organisms possess morphological and behavioural adaptations to prevent desiccation at low tide and dislodgement by wave forces. Burrowing animals are important in unconsolidated sediments. Competition (especially for space) is a major factor structuring communities, with its importance diminishing with decreasing particle size. Facilitative interactions (particularly those that protect organisms from desiccation stress or physical disturbance) can be important across ecosystems of all particle sizes. Biodiversity is generally high, with microscopic lifeforms dominating the biomass of systems of small particle size.

MT1.1 Rocky Shorelines

Ecosystem properties: These intertidal benthic systems, composed of sessile and mobile species, are highly structured by fine-scale resource and stress gradients, as well as trade-offs among competitive, facilitation, and predatory interactions. Sessile algae and invertebrates form complex three-dimensional habitats that provide microhabitat refugia from desiccation and temperature stress for associated organisms; these weaken competitive interactions. The biota exhibit behavioural and morphological adaptions to minimise exposure to stressors, such as seeking shelter in protective microhabitats at low tide, possessing exoskeletons (e.g. shells), or producing mucous to reduce desiccation. Morphologies, such as small body sizes and small cross-sectional areas to minimise drag, reflect adaptation to a wave-swept environment. Key trophic groups include filter-feeders (which feed on phytoplankton and dissolved organic matter at high tide), grazers (which scrape microphytobenthos and macroalgal spores from rock or consume macroalgal thalli), and resident (e.g. starfish, whelks, and crabs) and transient (e.g. birds and fish) marine and terrestrial predators. Rocky shores display high endemism relative to other coastal systems and frequently display high productivity due to the large amounts of light they receive, although this can vary according to nutrient availability from upwelling.

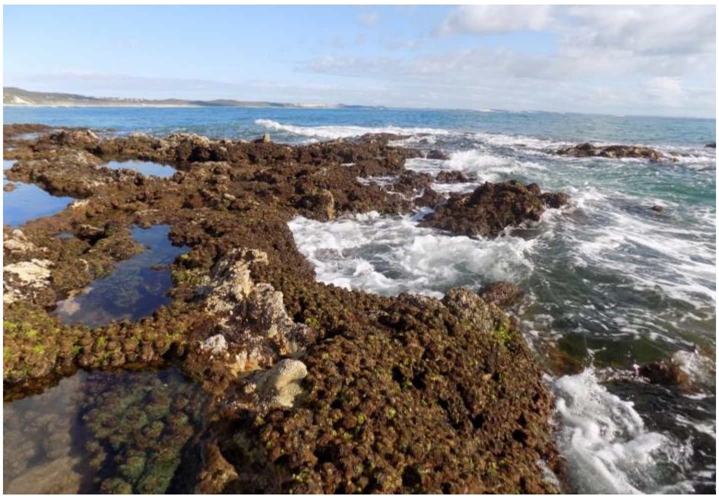

Rocky shore with colonial ascidians, southeast Qld, Australia.
Credit: Sven Lavender / Queensland Government

Ecological drivers: Tides and waves are the key ecological drivers, producing resource availability and physical disturbance gradients vertically and horizontally, respectively. Across the vertical gradient of increasing aerial exposure, desiccation and temperature stress increases, time available for filter-feeding decreases, and interactions with marine and terrestrial predators vary. The horizontal gradient of diminishing wave exposure from headlands to bays or inlets influences community composition and morphology. Many organisms rely on microhabitats formed from natural rock features (e.g. crevices, depressions, and rock pools) or habitat-forming species (e.g. canopy-forming algae, mussels, oysters, and barnacles) to persist in an environment that would otherwise exceed their environmental tolerances. Rocky shores are open systems, so community structure can be influenced by larval supply, coastal upwelling, and competition. Competition for space may limit the lower vertical distributions of some sessile species. The limited space available for the growth of marine primary producers can result in competition for food among grazers. Disturbances (i.e. storms, ice scour on subpolar shores) that free-up space can have a strong influence on community structure and diversity.

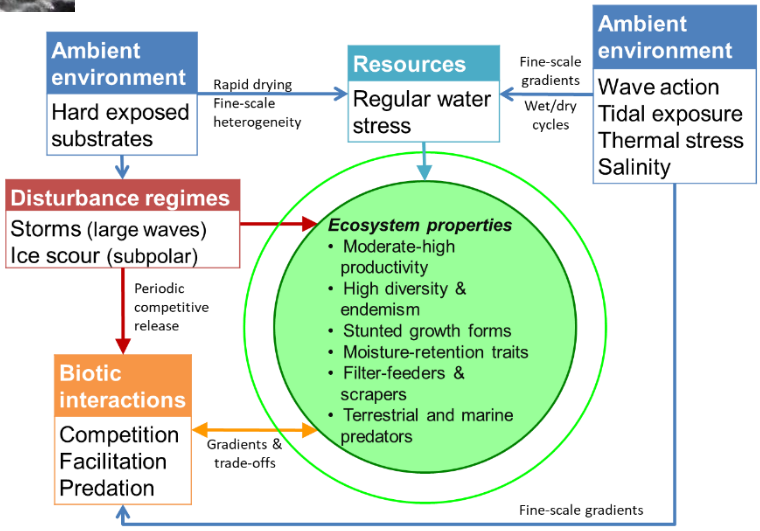

MT1.1. Rocky shores

Distribution: Found globally at the margins of oceans, where waves are eroding rocks. They are the most common ecosystems on open, high-energy coasts and also occur on many sheltered and enclosed coastlines, such as sea lochs, fjords, and rias.

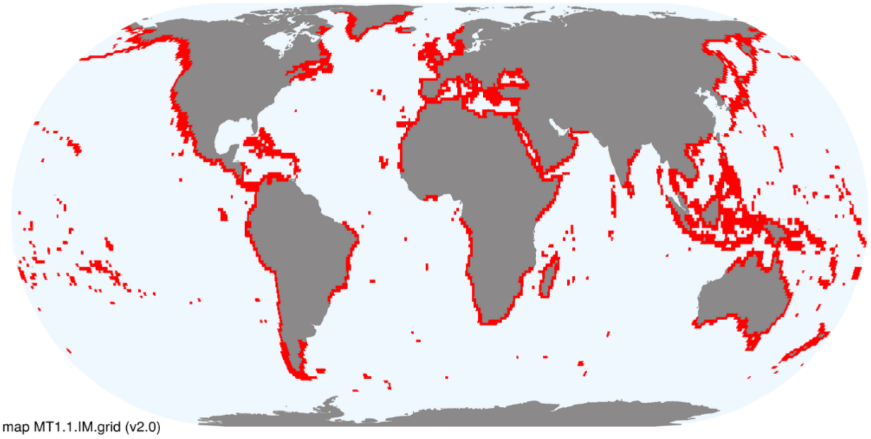

map MT1.1.IM.grid (v2.0)

References:

Connell JH (1972) Community interactions on marine rocky intertidal shores. *Annual Review of Ecology and Systematics* 3:169-192.

Thompson RC, Crowe TP, Hawkins SJ (2002) Rocky intertidal communities: past environmental changes, present status and predictions for the next 25 years. *Environmental Conservation* 29:168-91.

Map is for illustrative purposes only and does not support spatial analyses unless formally validated.

Contributors: MJ Bishop, NJ Murray, SE Swearer, DA Keith

MT1.2 Muddy Shorelines

Ecosystem properties: Highly productive intertidal environments are defined by their fine particle size (dominated by silts) and are fuelled largely by allochthonous production. Benthic diatoms are the key primary producer, although ephemeral intertidal seagrass may occur. Otherwise, macrophytes are generally absent unlike other ecosystems on intertidal mudflats (MFT1.2, MFT1.3). Fauna are dominated by deposit-feeding taxa (consuming organic matter that accumulates in the fine-grained sediments) and detritivores feeding on wrack (i.e. drift algae deposited at the high-water mark) and other sources of macro-detritus. Bioturbating and tube-dwelling taxa are key ecosystem engineers, the former oxygenating and mixing the sediments and the latter providing structure to an otherwise sedimentary habitat. Infauna residing within sediments are protected from high temperatures and desiccation by the surrounding matrix and do not display the same marked patterns of zonation as rocky intertidal communities. Many infaunal taxa are soft-bodied. Nevertheless, competition for food resources carried by incoming tides can lead to intertidal gradients in fauna. Predators include the substantial shorebird populations that forage on infauna at low tide, including migratory species that depend on these systems as stopover sites. Fish, rays, crabs, and resident whelks forage around lugworm bioturbation. Transitions to mangrove (MFT1.2), saltmarsh or reedbed (MFT1.3) ecosystems may occur in response to isostatic or sea level changes, freshwater inputs or changes in currents that promote macrophyte colonisation.

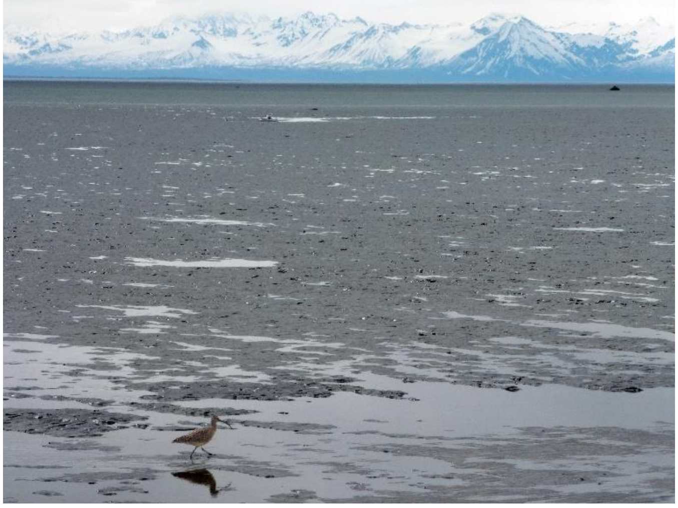

Coastal mudflats with foraging curlew, Alaska, USA.
Credit: Bill Raften / Getty Images

Ecological drivers: These are depositional environments influenced by sediment supply and the balance of erosion and sedimentation. They occur on lower wave energy coastlines with lower slopes and larger intertidal ranges than sandy shorelines, resulting in lower levels of sediment transport and oxygenation by physical processes. In the absence of burrowing taxa, sediments may display low rates of turnover, which may result in an anoxic zone close to the sediment surface. Small particle sizes limit interstitial spaces, further reducing aeration. The depth of the anoxic zone can be a key structuring factor. In contrast to sandy shorelines, they are organically rich and consequently higher in nutrients. Generally, muddy shorelines are formed from sediments supplied by nearby rivers, often remobilised from the seafloor throughout the tidal cycle.

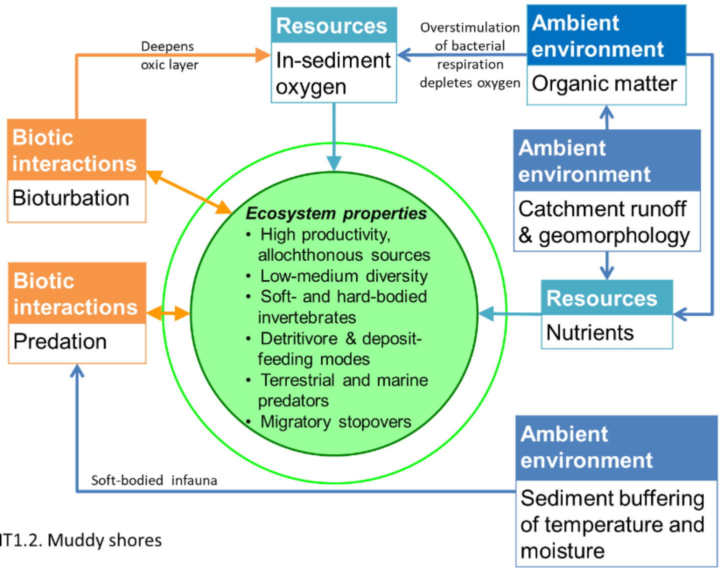

MT1.2. Muddy shores

Distribution: Muddy shorelines occur along low-energy coastlines, in estuaries and embayments where the velocity of water is so low that the finest particles can settle to the bottom.

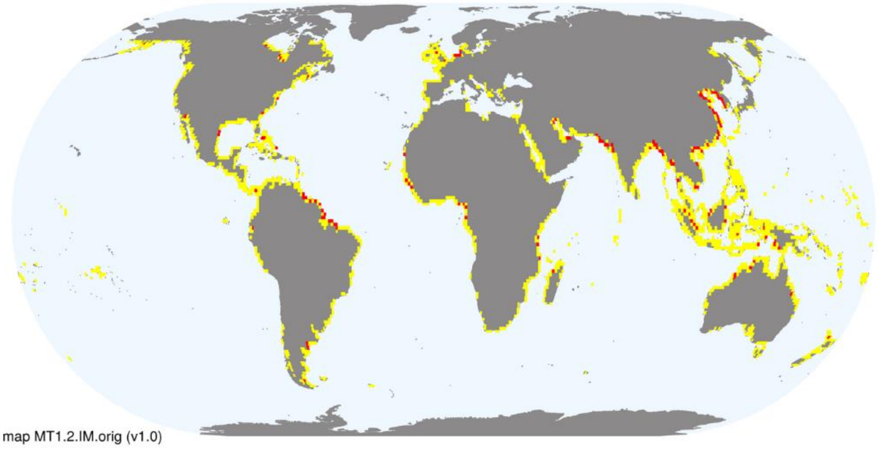

References:

Murray NJ, Phinn SR, DeWitt M, Ferrari R, Johnston R, Lyons MB, Clinton N, Thau D, Fuller RA (2019) The global distribution and trajectory of tidal flats. *Nature* 565: 222–225.

Peterson CH (1991) Intertidal zonation of marine invertebrates in sand and mud. *American Scientist* 79:236-249.

Wilson WH (1990) Competition and predation in marine soft-sediment communities. *Annual Review of Ecology and Systematics* 21: 221-241.

Map is for illustrative purposes only and does not support spatial analyses unless formally validated.

MT1.3 Sandy Shorelines

Ecosystem properties: Sandy shorelines include beaches, sand bars, and spits. These intertidal systems typically lack macrophytes, with their low productivity largely underpinned by detrital subsidies dominated by wrack (i.e. drift seaweed accumulating at the high-water mark) and phytoplankton, particularly in the surf zone of dissipative beaches. Salt- and drought-tolerant primary producers dominate adjacent dune systems (TM1.4). Meio-faunal biomass in many instances exceeds macrofaunal biomass. In the intertidal zone, suspension-feeding is a more common foraging strategy among invertebrates than deposit-feeding, although detritivores may dominate higher on the shore where wrack accumulates. Invertebrate fauna are predominantly interstitial, with bacteria, protozoans, and small metazoans contributing to the trophic network. Sediments are constantly shifting and thus invertebrate fauna are dominated by mobile taxa that display an ability to burrow and/or swash-ride up and down the beach face with the tides. The transitional character of these systems supports marine and terrestrial invertebrates and itinerant vertebrates from marine waters (e.g. egg-laying turtles) and from terrestrial or transitional habitats (e.g. shorebirds foraging on invertebrates or foxes foraging on carrion).

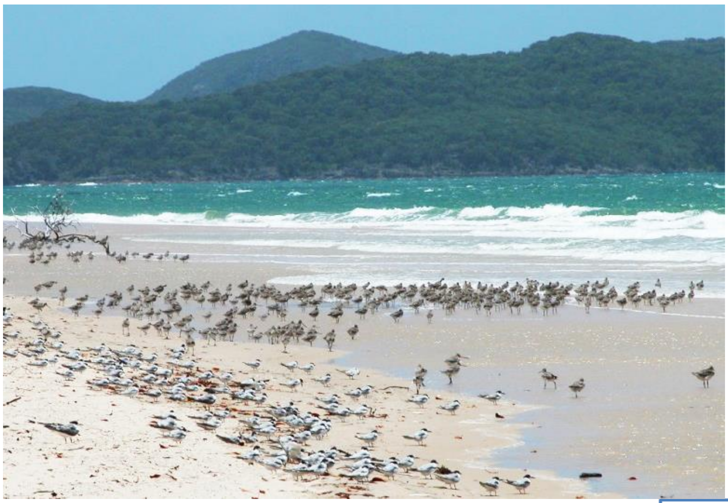

Shorebirds at high tide, Shoalwater Bay, Queensland, Australia.

Credit: Roger Jaensch

Ecological drivers: Physical factors are generally more important ecological drivers than biological factors. Particle size and wave and tidal regimes determine beach morphology, all of which influence the spatial and temporal availability of resources and niche diversity. Particle size is influenced by sediment sources as well as physical conditions and affects interstitial habitat structure. Wave action maintains substrate instability and an abundant supply of oxygen through turbulence. Tides and currents influence the dispersal of biota and regulate daily cycles of desiccation and hydration as well as salinity. Beach morphology ranges from narrow and steep (i.e. reflective) to wide and flat (i.e. dissipative) as sand becomes finer and waves and tides larger. Reflective beaches are accretional and more prevalent in the tropics; dissipative beaches are erosional and more common in temperate regions. Sands filter large volumes of seawater, with the volume greater on reflective than dissipative beaches. Beaches are linked to nearshore surf zones and coastal dunes through the storage, transport, and exchange of sand. Sand transport is the highest in exposed surf zones and sand storage the greatest in well-developed dunes.

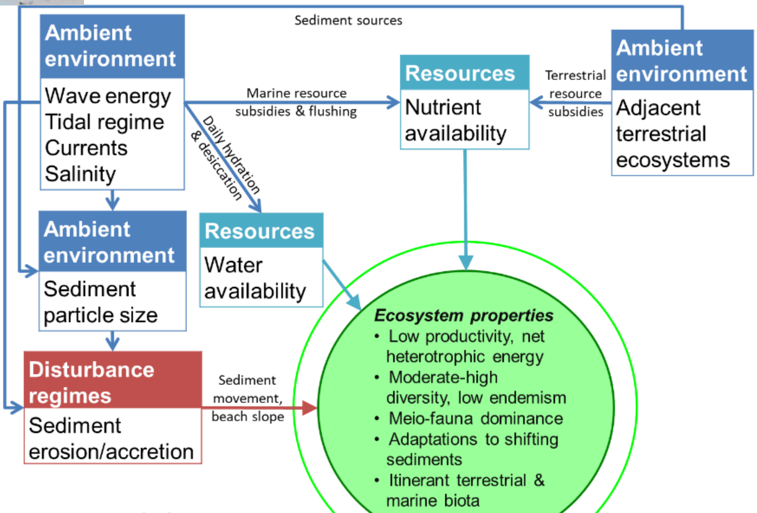

MT1.3. Sandy shores

Distribution: Sandy shores are most extensive at temperate latitudes, accounting for 31% of the ice-free global coastline, including 66% of the African coast and 23% of the European coast.

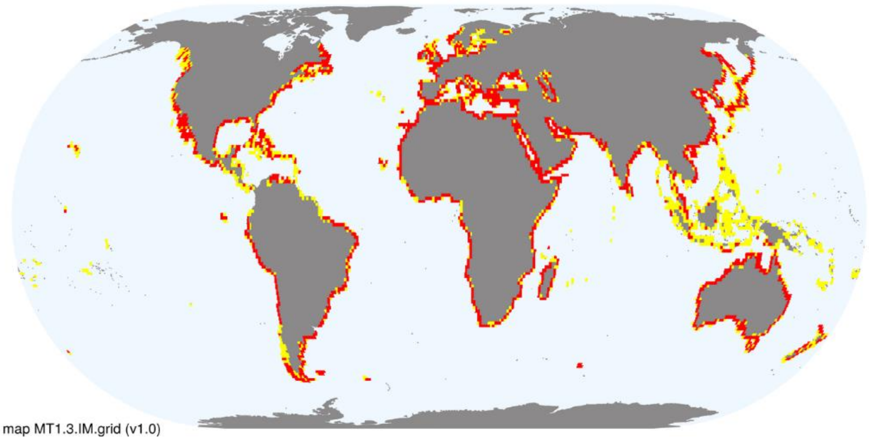

map MT1.3.IM.grid (v1.0)

References:

Luijendijk A, Hagenaars G, Ranasinghe R, Baart F, Donchyts G, Aarninkhof S (2018) The State of the World's Beaches. *Scientific reports* 8(1) 6641.

Schlacher TA, Schoeman DS, Dugan J, Lastra M, Jones A, Scapini F, McLachlan A. (2008) Sandy beach ecosystems: key features, sampling issues, management challenges and climate change impacts. *Marine Ecology* 29(S1):70-90.

Map is for illustrative purposes only and does not support spatial analyses unless formally validated.

Contributors: MJ Bishop, SL McSweeney, AH Altieri, DA Keith

MT1.4 Boulder and cobble shores

Ecosystem properties: These low-productivity, net heterotrophic systems are founded on unstable rocky substrates and share some ecological features with sandy beaches (MT1.3) and rocky shores (MT1.1). Traits of the biota reflect responses to regular substrate disturbance by waves and exposure of particles to desiccation and high temperatures. For example, in the high intertidal zone of boulder shores (where temperature and desiccation stress is most pronounced), fauna may be predominantly nocturnal. On cobble beaches, fauna are more abundant on the sub-surface because waves cause cobbles to grind against each other, damaging or killing attached fauna. Conversely, sandy beaches are where most fauna occupy surface sediments. Intermediate frequencies of disturbance lead to the greatest biodiversity. Only species with low tenacity (e.g. top shells) are found in surface sediments because they can detach and temporarily inhabit deeper interstices during disturbance events. High-tenacity species (e.g. limpets) or sessile species (e.g. macroalgae and barnacles) are more readily damaged, hence rare on cobble shores. Large boulders, however, are only disturbed during large storms and have more stable temperatures, so more fauna can persist on their surface. Encrusting organisms may cement boulders on the low shore, further stabilising them in turbulent water. Allochthonous wrack is the major source of organic matter on cobble beaches, but *in situ* autotrophs include superficial algae and vascular vegetation dominated by halophytic forbs. On some cobble beaches of New England, USA, extensive intertidal beds of the cordgrass Spartina alterniflora stabilise cobbles and provide shade, facilitating establishment of mussels, barnacles, gastropods, amphipods, crabs, and algae. In stabilising cobbles and buffering wave energy, cordgrass may also facilitate plants higher on the intertidal shore.

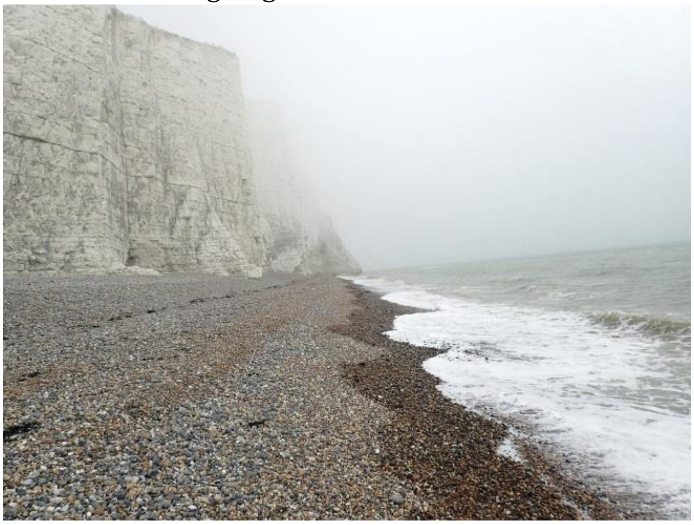

Cobble Beach, South Downs, England.
Credit: David Keith

Ecological drivers: Particle size (e.g. cobbles vs. boulders) and wave activity determine substrate mobility, hence the frequency of physical disturbance to biota. Ecosystem engineers modify these relationships by stabilising the substrate. Cobble beaches are typically steep because waves easily flow through large interstices between coarse beach particles, reducing the effects of backwash erosion. Hence swash and breaking zones tend to be similar widths. The permeability of cobble beaches leads to desiccation and heat stress at low tide along the beach surface gradient. Desiccation stress is extreme on boulder shores, playing a similar role in structuring communities as on rocky shores. The extent of the fine sediment matrix present amongst cobbles, water supply (i.e. rainfall), and the frequency of physical disturbance all influence beach vegetation. Alongshore grading of sediment by size could occur on long, drift dominated shorelines, which may influence sediment calibre on the beach.

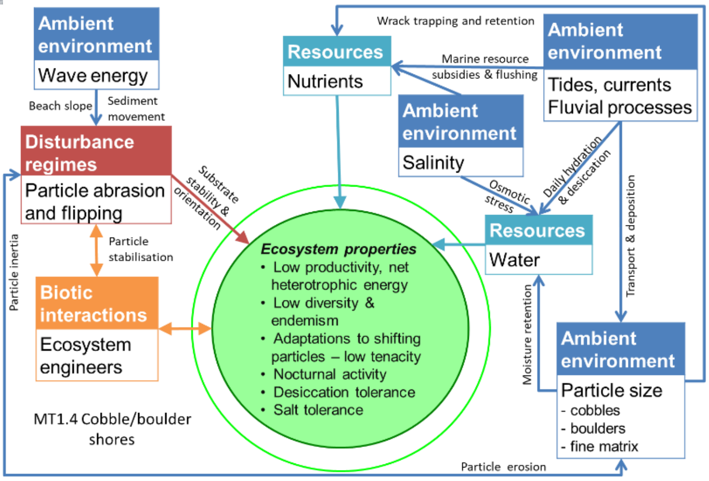

Distribution: Cobble beaches occur where rivers or glaciers delivered cobbles to the coast or where they were eroded from nearby coastal cliffs. They are most common in Europe and also occur in Bahrain, North America, and New Zealand's South Island.

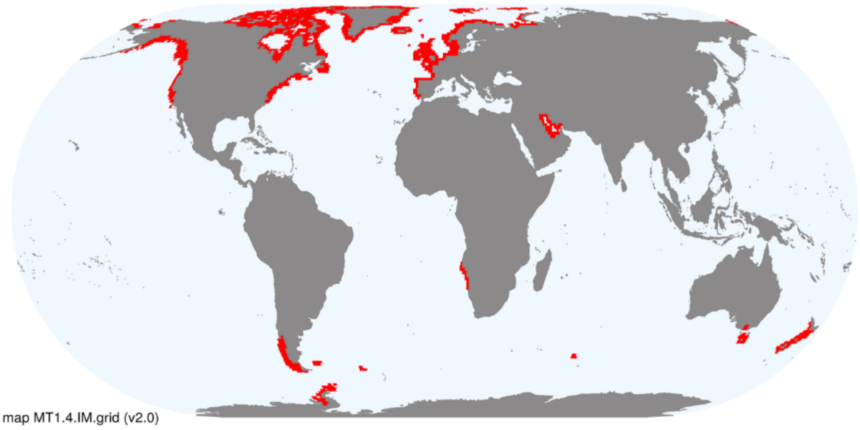

map MT1.4.IM.grid (v2.0)

Map is for illustrative purposes only and does not support spatial analyses unless formally validated.

References:

Altieri AH, Silliman BR, Bertness MD (2007) Hierarchical organization via a facilitation cascade in intertidal cordgrass bed communities. *The American Naturalist* 169: 195-206.

Scott GAM (1963) The ecology of shingle beach plants. *Journal of Ecology* 51: 517-527.

MT2. Supralittoral coastal biome

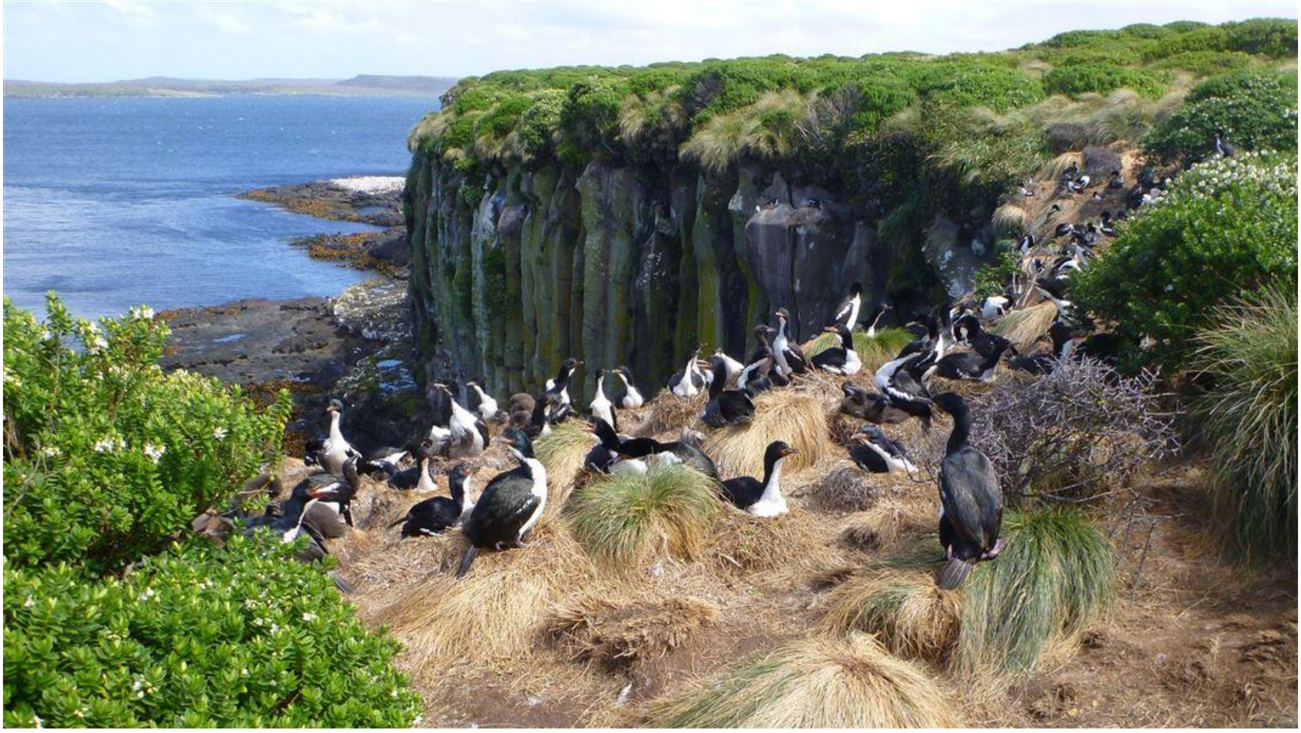

Auckland Islands sea cliff heath and rookery.

Credit: Jo Hiscock, New Zealand Department of Conservation

The Supralittoral coastal biome marks the landward extent of the transition from marine to terrestrial biomes. It is elevated above the direct influence of waves and tides (see the Shoreline biome) and beyond the direct influence of freshwater seepage or rivers (see brackish tidal biota). Supratidal coastal ecosystems extend around all the world's land masses, occupying a fringe from tens of metres to a few kilometres wide and covering the entire extent of many small islands.

Onshore winds, created by differences in air pressure related to the differing heat capacities of water and dry land, are a key driver of ecosystem function. These winds create desiccating conditions on elevated landforms such as headlands and coastal dunes, as well as continual inputs of aerosol salts and salt spray. Even though the supralittoral zone is located above high spring tide, it is exposed to recurring disturbance from storms producing exceptional waves and tides that reduce standing biomass and destabilise substrates.

These strong environmental gradients select for a specialised, low-diversity biota. Much of this biota is confined to supralittoral ecosystems and nowhere else, a key feature of these ecosystems, although it may be widely distributed behind shorelines on different land masses due to dispersal by coastal winds, oceanic currents, and/or migratory behaviour. Autochthonous energy is produced by wind-pruned vegetation with traits promoting tolerance to desiccation, high salinity, and substrate instability (e.g. stomatal regulation, extensive rhizomes or root systems, and succulence). The sea supplies allochthonous energy subsidies such as wrack and guano but also transports a portion of primary production to other ecosystems. Invertebrate detritivores and physical weathering contribute to rapid decay. Supralittoral ecosystems also provide nesting habitat for seabirds on the surface, in vegetation or in burrows, especially on islands free from terrestrial mammalian predators.

MT2.1 Coastal shrublands and grasslands

Ecosystem properties: Relatively low productivity grasslands, shrublands, and low forests on exposed coastlines are limited by salt influx, water deficit, and recurring disturbances. Diversity is low across taxa and trophic networks are simple, but virtually all plants and animals have strong dispersal traits and most consumers move between adjacent terrestrial and marine ecosystems. Vegetation and substrates are characterised by strong gradients from sea to land, particularly related to aerosol salt inputs, substrate instability and disturbance associated with sea storms and wave action. Plant traits conferring salt tolerance (e.g. succulent and sub-succulent leaves and salt-excretion organs) are commonly represented. Woody plants with ramulose and/or decumbent growth forms and small (microphyll-nanophyll) leaves reflect mechanisms of persistence under exposure to strong salt-laden winds, while modular and rhizomatous growth forms of woody and non-woody plants promote persistence, regeneration, and expansion under regimes of substrate instability and recurring disturbance. These strong environmental filters promote local adaptation, with specialised genotypes and phenotypes of more widespread taxa commonly represented on the strandline. Fauna are highly mobile, although some taxa such as ground-nesting seabirds may be sedentary for some parts of their lifecycles. Ecosystem dynamics are characterised by disturbance-driven cycles of disruption and renewal, with early phases dominated by colonists and *in situ* regenerators that often persist during the short intervals between successive disturbances.

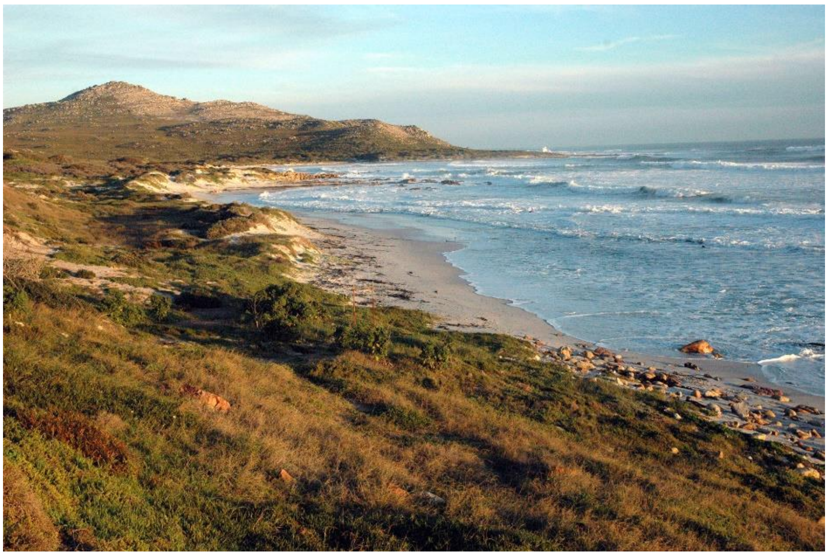

Coastal shrubland, Strait of Magellan, Chile.
Credit: David Keith

Ecological drivers: Desiccating winds promote an overall water deficit and appreciable exposure to salinity due to aerosol influx and salt spray. Warm to mild temperatures across the tropics to temperate zones and cold temperatures in the cool temperate to boreal zones are moderated by direct maritime influence. Above the regular intertidal zone, these systems are exposed to periodic disturbance from exceptional tides, coastal storm events, wind shear, bioturbation, and aeolian substrate mobility. Consolidated substrates (headlands, cliffs) may differ from unconsolidated dunes in their influence on function and biota. Geomorphological depositional and erosional processes influence substrate stability and local vegetation succession.

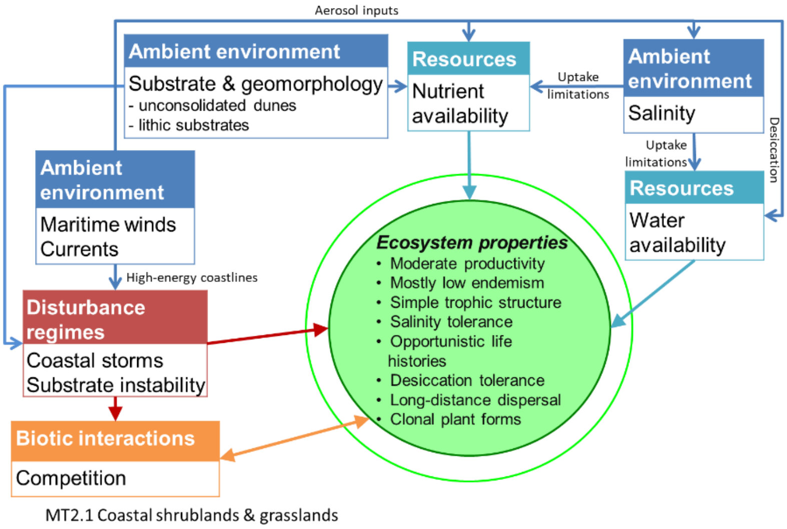

Distribution: Coastal dunes and cliffs throughout tropical, temperate, and boreal latitudes.

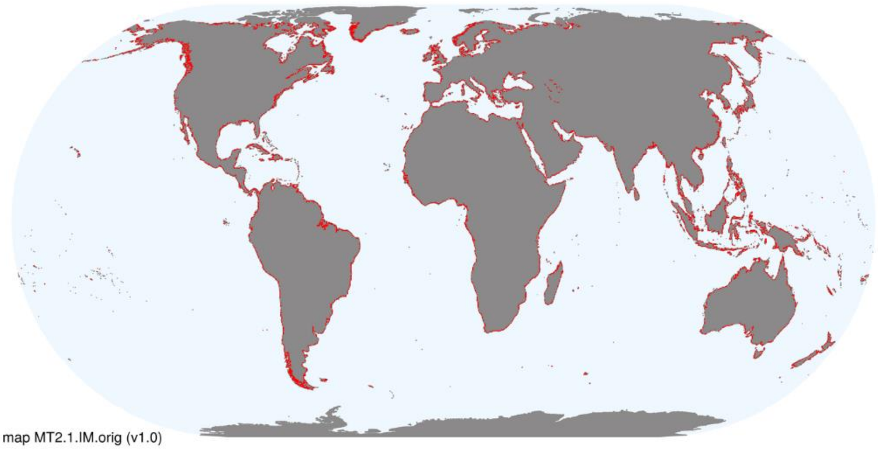

map MT2.1.IM.orig (v1.0)

References:

van der Maarel E (2001) Dry coastal ecosystems: General aspects. *Ecosystems of the world* 2C. Elsevier, Amsterdam.

Map is for illustrative purposes only and does not support spatial analyses unless formally validated.

Contributors: SBZ Gorta, SF Bokhorst, JC. Ellis, DA Keith

MT2.2 Large seabird and pinniped colonies

Ecological properties: Large seabird and pinniped colonies are localised eutrophic terrestrial ecosystems near the ocean interface that receive massive nutrient subsidies from large concentrations of roosting or nesting seabirds and pinnipeds that function as mobile links between land and sea. The marine-derived subsidies and potentially massive physical disturbance to vegetation and soils distinguish these colonies from otherwise similar ecosystems in MT2.1. Subsidies are greatest where seabird body size is typically larger (e.g. penguins) and breeding seasons are longer, particularly the sub-Antarctic and Antarctic. The waters around these ecosystems may be locally depleted in seabird prey due to prolonged predation. Colonies occupy diverse habitats, from sandy shores to rocky islands and montane forests, with vegetation composition and structure limited by physical disturbance, nutrient input, salt influx and gradient (e.g., sea spray), water deficit, surface and subsurface bioturbation-driven changes in soil condition and pH, avian seed dispersal, unstable substrates, and high exposure, often exhibiting salt tolerance and clonal reproduction. Plant assemblages exist across a gradient, influenced by seabird/pinniped disturbance, nutrient input and climate, whereby high-density colonies can completely suppress plant growth, but where disturbance and nutrient load is lower, vegetation can establish, typically in low richness but high abundance. Trophic networks are characterized high microbial activity and abundant invertebrates in soils which can lead to localised biodiversity hotspots, in contrast to the low richness of plant communities under high nutrient loading. There are typically low densities or a total absence of terrestrial mammalian predators and grazers (limited by dispersal barriers). Vibrant and specialised lichens can be abundant. Plant dispersal linked to bird migration, and nutrient transport between marine foraging areas and terrestrial breeding areas, may occur over long distances.

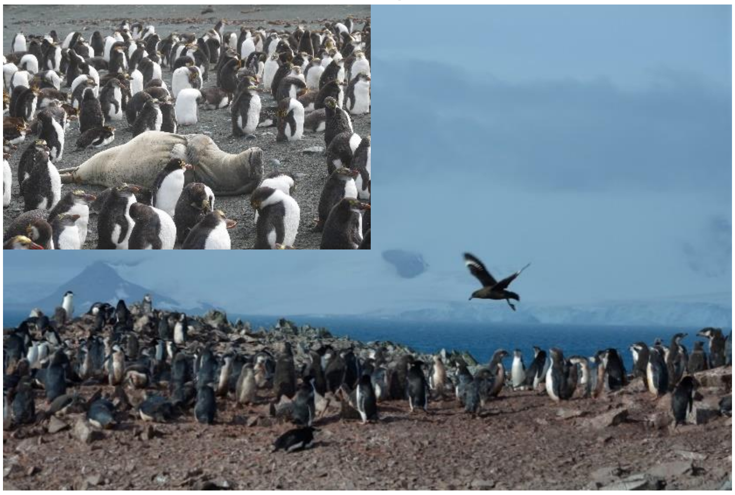

Chinstrap penguin colony with Skua, South Shetland Islands, Antarctica. Inset: Royal penguin colony with Southern elephant seal, Macquarie Island
Credit: David Keith, inset Max Breckenridge

Ecological drivers: Marine subsidies of nutrients, excreted by marine-foraging seabirds and pinnipeds, drives eutrophication, resulting in the highest terrestrial concentrations of nitrogen, phosphorus, and other nutrients on Earth's surface. Nutrients may be derived from sources proximal to, or remote from the colony, and may be continual or pulsed, depending on the colony location, size, constituent species, and seasonal variation in attendance. Substrates vary from sand to soil to rock to ice, and desiccating winds add aerosol salts and limit water availability in coastal colonies. Temperatures vary from warm to mild in tropical/temperate/boreal latitudes to freezing in polar regions. Bioturbation, coastal storms, and unstable substrates influence biotic interactions and colony abundance and distribution.

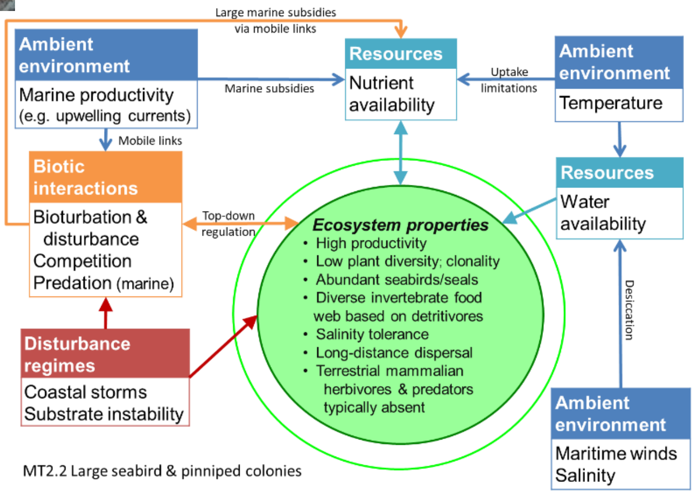

MT2.2 Large seabird & pinniped colonies

Distribution: Scattered globally on islands and coastlines, but most common in polar and subpolar regions

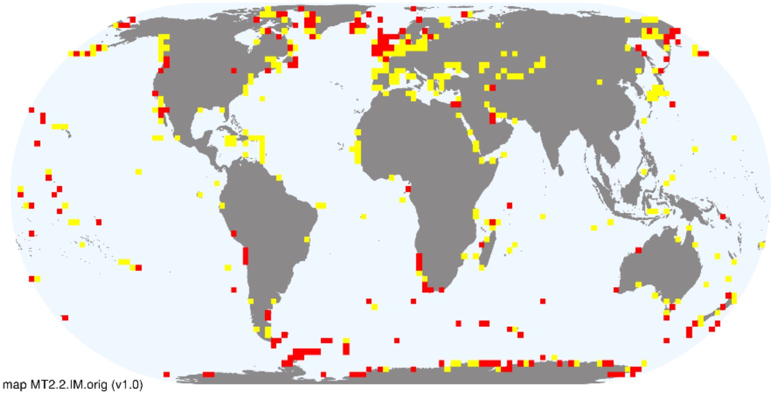
map MT2.2.IM.orig (v1.0)

References:

Ellis JC (2005) Marine Birds on land: A review of plant biomass, species richness, and community composition in seabird colonies. Plant Ecology 181, 227–241.

Otero XL, De La Peña-Lastra S, Pérez-Alberti A, Ferreira TO, Huerta-Diaz MA (2018) Seabird colonies as important global drivers in the nitrogen and phosphorus cycles. Nature Communications 9, 246.

Riddick SN, Dragosits U, Blackall TD, Daunt F, Wanless S, Sutton MA (2012) The global distribution of ammonia emissions from seabird colonies. Atmospheric Environment 55, 319e327.

Map is for illustrative purposes only and does not support spatial analyses unless formally validated.

MT3. Anthropogenic shorelines biome

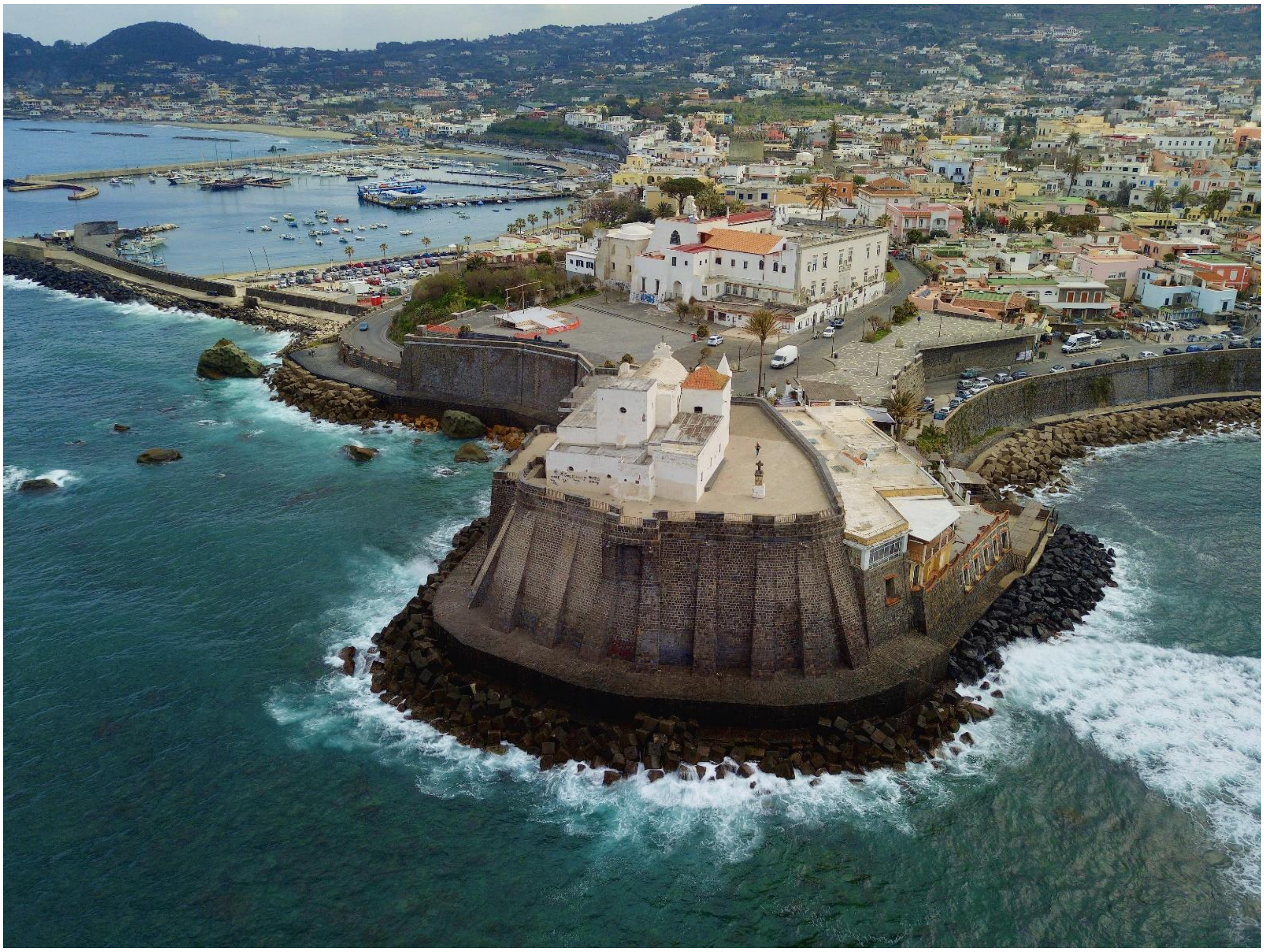

Constructed rubble shorelines and seawalls,of Forio township, Ischia, Italy

Credit: Leonardo Malaguti / Getty Images

The Anthropogenic shorelines biome is distributed globally where urbanised and industrial areas adjoin the coast, and includes some more remote structures such as artificial islands. It includes marine interfaces constructed from hard, smooth surfaces, including concrete, timber, lithic blocks, and earthen fill, adjoining, extending or replacing natural shores, or floating in proximity to them.

These relatively homogeneous substrates support an opportunistic, cosmopolitan biota with limited diversity and simplified trophic structure compared to other shoreline systems. Vertical surfaces are inhabited by algae and biofouling species but are exposed to strong tidal desiccation regimes that strongly filter potential colonists. Floating structures have downward-facing, usually smooth, surfaces, unlike almost anything in nature, which may be colonised by opportunists.

Influx of storm water and effluent enhances nutrient levels and eutrophic algae, which contribute autochthonous energy. Outflows from developed areas are also a major sources of allochthonous energy. Strong bottom-up regulation stems from these resource inputs and from low populations of predators, which are depleted or deterred by human activity.

MT3.1 Artificial shorelines

Ecosystem properties: Constructed sea walls, breakwaters, piers, docks, tidal canals, islands and other coastal infrastructure create substrates inhabited by inter-tidal and subtidal, benthic and demersal marine biota around ports, harbours, and other intensively settled coastal areas. Structurally simple, spatially homogeneous substrates support a cosmopolitan biota, with no endemism and generally lower taxonomic and functional diversity than rocky shores (MT1.1). Trophic networks are simple and dominated by filter-feeders (e.g. sea squirts and barnacles) and biofilms of benthic algae and bacteria. Low habitat heterogeneity and the small surface area for attachment that the often vertical substrate provides, regulate community structure by promoting competition and limiting specialised niches (e.g. crevices or pools) and restricting refuges from predators. Small planktivorous fish may dominate temperate harbours and ports. These can provide a trophic link, but overharvest of predatory fish and sharks may destabilise food webs and cause trophic cascades. Much of the biota possess traits that promote opportunistic colonisation, including highly dispersive life stages (e.g. larvae, eggs, and spores), high fecundity, generalist settlement niches and diet, wide ranges of salinity tolerance, and rapid population turnover. These structures typically contain a higher proportion of non-native species than the natural substrates they replace.

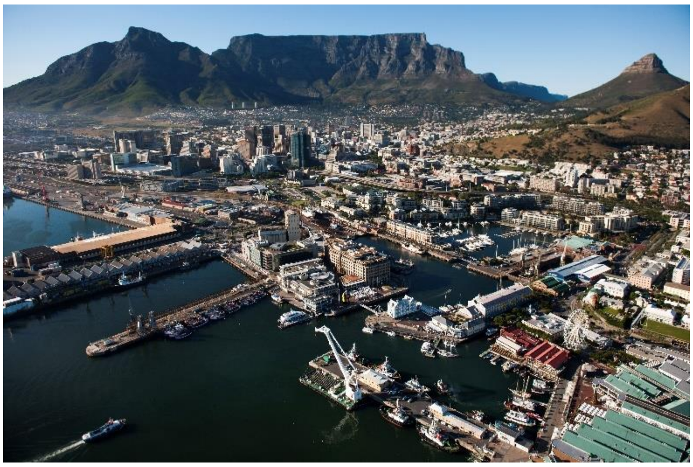

Cape Town Harbour, South Africa.
Credit: Mark Edward Harris / Getty Images

Ecological drivers: The substrate material influences the texture, chemistry, and thermal properties of the surface. Artificial structures of wood, concrete, rock, or steel have flat, uniform, and vertical surfaces that limit niche diversity and exacerbate inter-tidal gradients in desiccation and temperature. Floating structures have downward-facing surfaces, rare in nature. Some structures are ecologically engineered (designed for nature) to provide more complex surfaces and ponds to enhance biodiversity and ecosystem function. Structures may be located in high (i.e. breakwaters) or low (i.e. harbours) energy waters. Tides and waves are key drivers of onshore resource and kinetic energy gradients. Brackish water plumes from polluted storm water and sewage overflows add allochthonous nutrients, organic carbon, and open ecological space exploited by invasive species introduced by shipping and ballast water. The structures are often located close to vectors for invasive species (e.g. transport hubs). Boat traffic and storm water outflows cause erosion and bank instability and maintain high turbidity in the water column. This limits photosynthesis by primary producers, but nutrient run-off may increase planktonic productivity. Maintenance regimes (e.g. scraping) reduce biomass and reset succession.

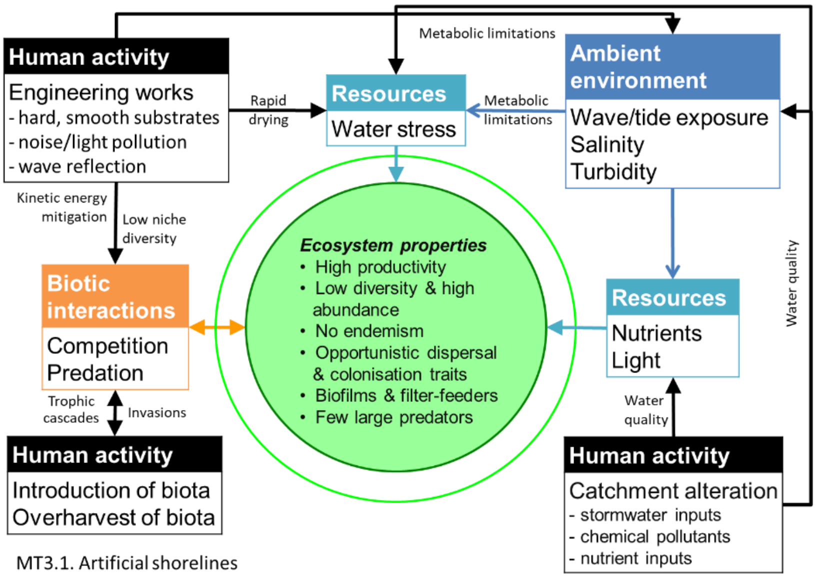

MT3.1. Artificial shorelines

Distribution: Urbanised coasts through tropical and temperate latitudes, especially in North and Central America, Europe, and North and South Asia.

References:

Dafforn KA, Glasby TM, Airoldi L, Rivero NK, Mayer-Pinto M, Johnston EL (2015) Marine urbanization: an ecological framework for designing multifunctional artificial structures. *Frontiers in Ecology and the Environment* 13: 82–90.

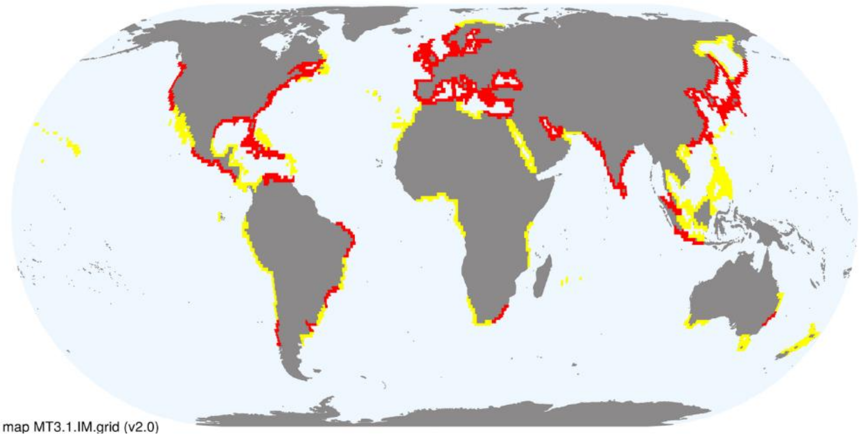
map MT3.1.IM.grid (v2.0)

Map is for illustrative purposes only and does not support spatial analyses unless formally validated.

MFT1. Brackish tidal biome

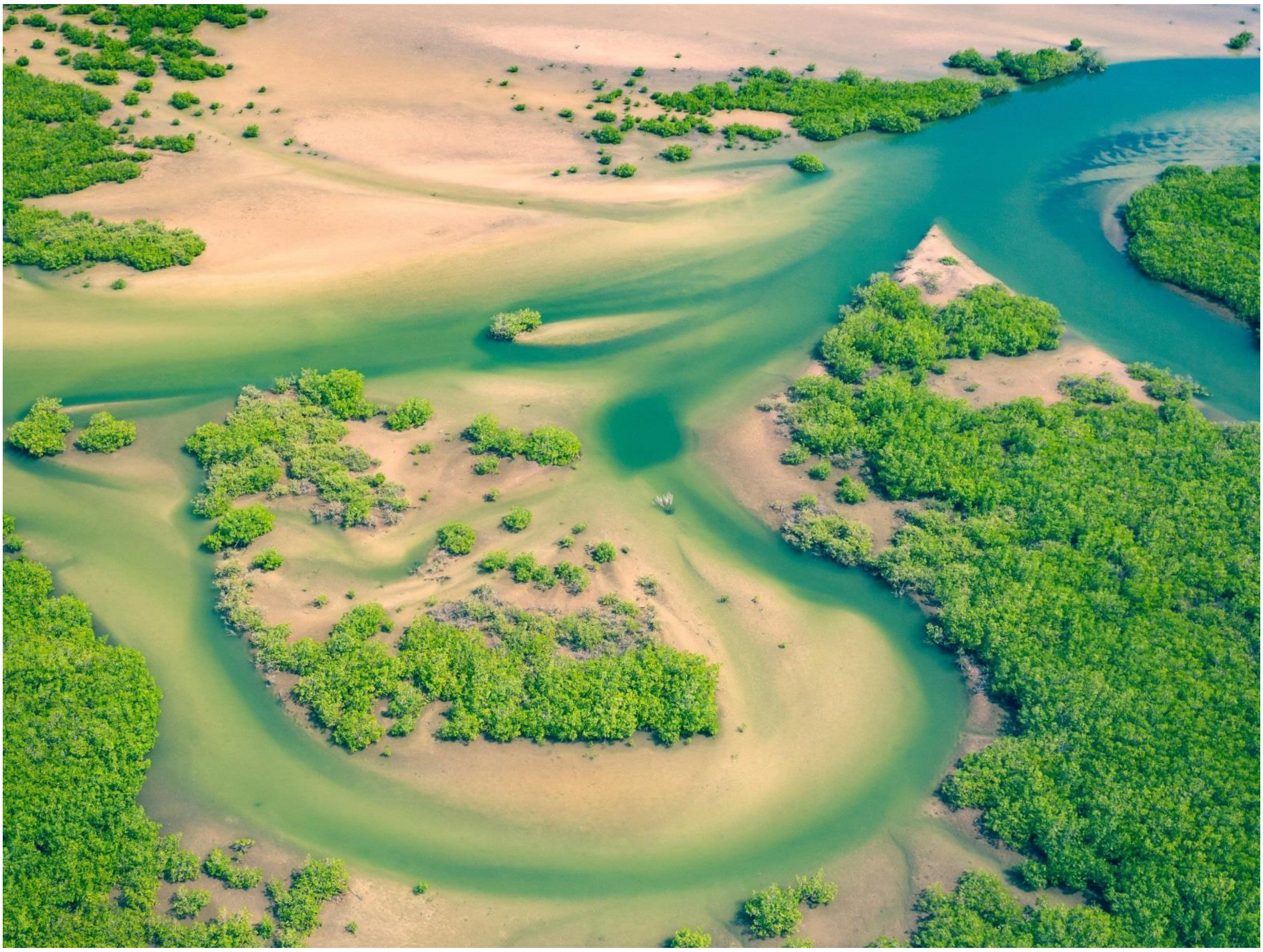

Aerial view of mangrove forest in the Saloum Delta National Park, Senegal.

Credit: Curioso Photography / Unsplash

The Brackish tidal systems biome is associated with prograding depositional shorelines at the interface of terrestrial, freshwater, and marine realms. The relative influences of marine, freshwater, and terrestrial processes vary from strongly fluvial deltas to marine-dominated intertidal forests and terrestrial-dominated coastal saltmarsh.

Autochthonous sources of energy, contributed by flowering plants and algae, are supplemented by allochthonous sources delivered by rivers, currents, and tides. These sources support high productivity and complex trophic webs that include highly mobile fish and birds that rely on brackish tidal systems to complete their lifecycles. Standing plants assimilate energy and engineer habitat structure for epifauna and epiflora as well as juvenile fish nurseries. They also promote sediment deposition by dampening wave and tidal energy.

While terrestrial systems are the ultimate source of most sediment, fluvial and marine processes redistribute it and drive patch dynamics across temporal and spatial scales. Brackish tidal systems are structured by steep local gradients in salinity and tidal exposure. Physiological traits that confer differential fitness and competitive abilities, together with differential predation pressure, mediate species turnover along gradients.

Brackish tidal systems are distributed on depositional coastlines throughout the world.

MFT1.1 Coastal river deltas

Ecosystem properties: Coastal river deltas are prograding depositional systems, shaped by freshwater flows and influenced by wave and tidal flow regimes and substrate composition. The biota of these ecosystems reflects strong relationships with terrestrial, freshwater, and marine realms at different spatial scales. Consequently, they typically occur as multi-scale mosaics comprised of unique elements juxtaposed with other functional groups that extend far beyond the deltaic influence, such as floodplain marshes (FT1.2), mangroves (MFT1.2), sandy shorelines (TM1.3), and subtidal muddy plains (M1.8). Gradients of water submergence and salinity structure these mosaics. Allochthonous subsidies from riverine discharge and marine currents supplement autochthonous sources of energy and carbon and contribute to high productivity. Complex, multi-faceted trophic relationships reflect the convergence and integration of three contrasting realms and the resulting niche diversity. Autotrophs include planktonic algae and emergent and submerged aquatic plants, which contribute to trophic networks mostly through organic detritus (rather than herbivory). Soft sediments and flowing water are critical to in-sediment fauna dominated by polychaetes and molluscs. Freshwater, estuarine, and marine fish and zooplankton are diverse and abundant in the water column. These provide food for diverse communities of wading and fishing birds, itinerant marine predators, and terrestrial scavengers and predators (e.g. mammals and reptiles). Virtually all biota have life-history and/or movement traits enabling them to exploit highly dynamic ecosystem structures and disturbance regimes. High rates of turnover in habitat and biota are expressed spatially by large fluctuations in the mosaic of patch types that make up deltaic ecosystems.

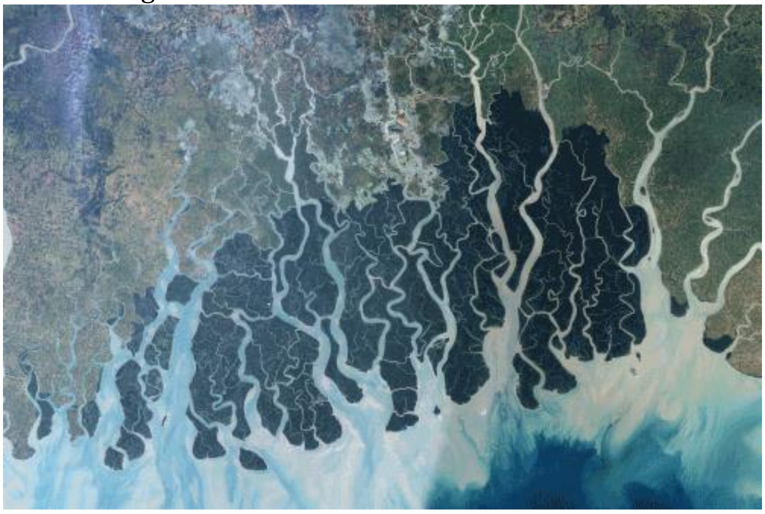

Sundarbans, Ganges Delta, India & Bangladesh.
Credit: Jesse Allen, NASA Earth Observatory

Ecological drivers: River inflows structure the dynamic mosaics of coastal river deltas. Inflows depend on catchment geomorphology and climate and influence water levels, nutrient input, turbidity (hence light penetration), tidal amplitude, salinity gradients, temperature, dissolved oxygen, and organic carbon. Rates of delta aggradation depend on interactions among riverine sedimentation and ocean currents, tides, and wave action, which disperse sediment loads. Coastal geomorphology influences depth gradients. These processes result in complex, spatio-temporally variable mosaics of distributary channels, islands, floodplains, mangroves, subtidal mud plains, and sand beds. Regimes of floods and storm surges driven by weather in the river catchment and ocean, respectively, have a profound impact on patch dynamics.

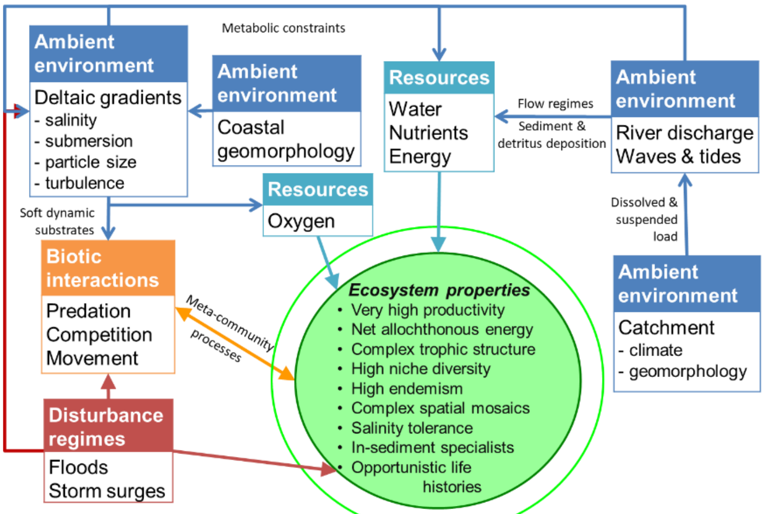

MFT1.1 Coastal River Deltas

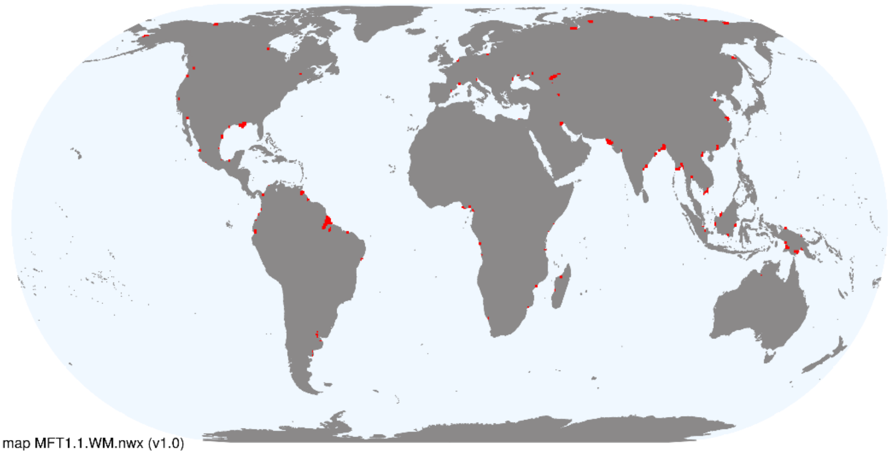

Distribution: Continental margins where rivers connect the coast to high-rainfall catchments, usually with high mountains in their headwaters.

References:

Bianchi TS, Allison MA (2009) Large-river delta-front estuaries as natural "recorders" of global environmental change. *Proceedings of the National Academy of Sciences of the USA* 106:8085-8092.

Orton GJ, Reading G (1993) Variability of deltaic processes in terms of sediment supply, with particular emphasis on grain-size. *Sedimentology* 40:475-512.

Map is for illustrative purposes only and does not support spatial analyses unless formally validated.

Contributors: MJ Bishop, AH Altieri, TS Bianchi, DA Keith

MFT1.2 Intertidal forests and shrublands

Ecosystem properties: Mangroves are structural engineers and possess traits including pneumatophores, salt excretion glands, vivipary, and propagule buoyancy that promote survival and recruitment in poorly aerated, saline, mobile, and tidally inundated substrates. They are highly efficient in nitrogen use efficiency and nutrient resorption. These systems are among the most productive coastal environments. They produce large amounts of detritus (e.g. leaves, twigs, and bark), which is either buried in waterlogged sediments, consumed by crabs, or more commonly decomposed by fungi and bacteria, mobilising carbon and nutrients to higher trophic levels. These ecosystems are also major blue carbon sinks, incorporating organic matter into sediments and living biomass. Although highly productive, these ecosystems are less speciose than other coastal biogenic systems. Crabs are among the most abundant and important invertebrates. Their burrows oxygenate sediments, enhance groundwater penetration, and provide habitat for other invertebrates such as molluscs and worms. Specialised roots (pneumatophores) provide a complex habitat structure that protects juvenile fish from predators and serves as hard substrate for the attachment of algae as well as sessile and mobile invertebrates (e.g. oysters, mussels, sponges, and gastropods). Mangrove canopies support invertebrate herbivores and other terrestrial biota including invertebrates, reptiles, small mammals, and extensive bird communities. These are highly dynamic systems, with species distributions adjusting to local changes in sediment distribution, tidal regimes, and local inundation and salinity gradients.

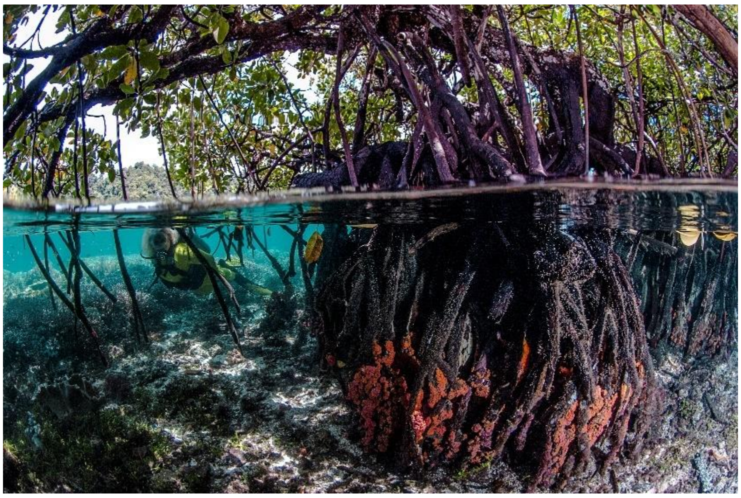

Mangroves at high tide, Raja Ampat, West Papua, Indonesia.
Credit: Giordano Cipriani / Getty Images

Ecological drivers: Mangroves are physiologically intolerant of low temperatures, which excludes them from regions where mean air temperature during the coldest months is below 20°C, where the seasonal temperature range exceeds 10°C, or where ground frost occurs. Many mangrove soils are low in nutrients, especially nitrogen and phosphorus. Limited availability of nitrogen and phosphorus Regional distributions are influenced by interactions among landscape position, rainfall, hydrology, sea level, sediment dynamics, subsidence, storm-driven processes, and disturbance by pests and predators. Rainfall and sediment supply from rivers and currents promote mangrove establishment and persistence, while waves and large tidal currents destabilise and erode mangrove substrates, mediating local-scale dynamics in ecosystem distributions. High rainfall reduces salinity stress and increases nutrient loading from adjacent catchments, while tidal flushing also regulates salinity.

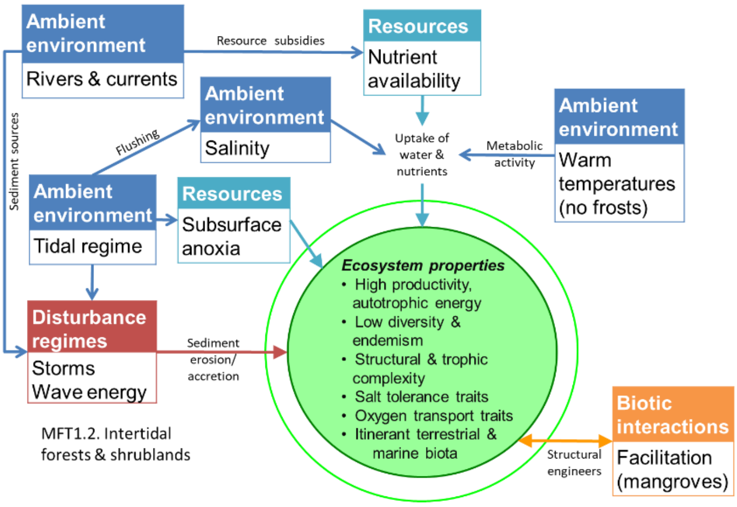

Distribution: Widely distributed along tropical and warm temperate coastlines of the world. Large-scale currents may prevent buoyant seeds from reaching some areas.

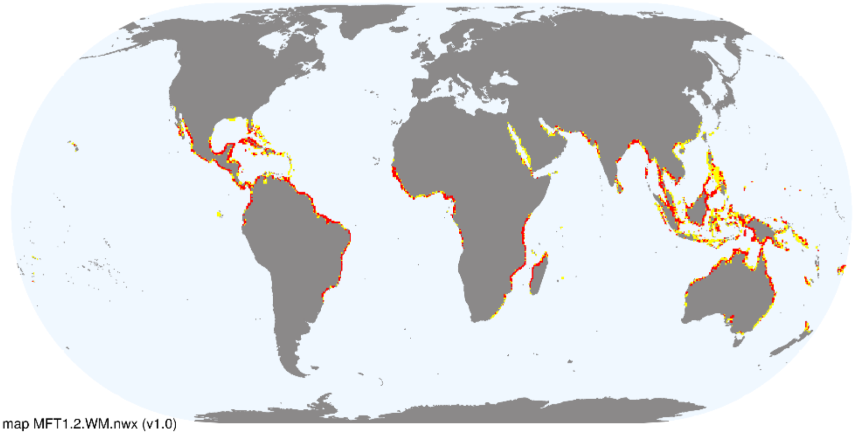

References:

Duke N, Ball M, Ellison J (1998) Factors influencing biodiversity and distributional gradients in mangroves. *Global Ecology & Biogeography Letters* 7:27-47.

Feller IC, Lovelock CE, Berger U,, McKee KL, Joye SB, Ball MC (2010) Biocomplexity in mangrove ecosystems. *Annual Review of Marine Science* 2, 395–417.

Krauss KW, Lovelock CE, McKee KL, López-Hoffman L, Ewe SM, Sousa WP (2008) Environmental drivers in mangrove establishment and early development: a review. *Aquatic Botany* 89:105-27.

Map is for illustrative purposes only and does not support spatial analyses unless formally validated.

Contributors: DA Keith, AH Altieri, J Loidi, MJ Bishop

MFT1.3 Coastal saltmarshes and reedbeds

Ecosystem properties: Coastal saltmarshes are vegetated by salt-tolerant forbs, grasses, and shrubs, with fine-scale mosaics related to strong local hydrological and salinity gradients, as well as competition and facilitation. Plant traits such as succulence, salt excretion, osmotic regulation, reduced transpiration, C4 photosynthesis (among grasses), modular growth forms, and aerenchymatous tissues confer varied degrees of tolerance to salinity, desiccation, and substrate anoxia. Adjacent marine and terrestrial ecosystems influence the complexity and function of the trophic network, while freshwater inputs mediate resource availability and physiological stress. Angiosperms are structurally dominant autotrophs, but algal mats and phytoplankton imported by tidal waters contribute to primary production. Cyanobacteria and rhizobial bacteria are important N-fixers. Tides and run-off bring subsidies of organic detritus and nutrients (including nitrates) from marine and terrestrial sources, respectively. Nitrogen is imported into saltmarshes mainly as inorganic forms and exported largely as organic forms, providing important subsidies to the trophic networks of adjacent estuarine fish nurseries (FM1.2). Fungi and bacteria decompose dissolved and particulate organic matter, while sulphate-reducing bacteria are important in the decay of substantial biomass in the anaerobic subsoil. Protozoans consume microbial decomposers, while *in situ* detritivores and herbivores include a range of crustaceans, polychaetes, and molluscs. Many of these ingest a mixture of organic material and sediment, structuring, aerating, and increasing the micro-scale heterogeneity of the substrate with burrows and faecal pellets. Fish move through saltmarsh vegetation at high tide, feeding mainly on algae. They include small-bodied residents and juveniles of larger species that then move offshore. Itinerant terrestrial mammals consume higher plants, regulating competition and vegetation structure. Colonial and solitary shorebirds breed and/or forage in saltmarsh. Migratory species that play important roles in the dispersal of plants, invertebrates, and microbes, while abundant foragers may force top-down transformational change.

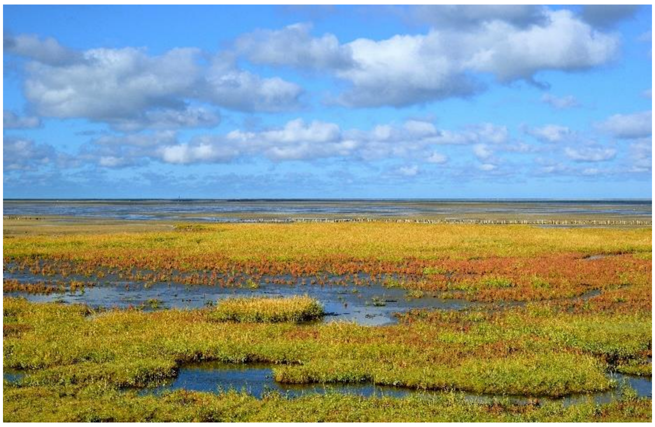

Glasswort saltmarshes, Schleswig-Holstein Wadden Sea NP Germany
Credit: Frederick / Getty Images

Ecological drivers: High and variable salt concentration is driven by alternating episodes of soil desiccation and flushing, associated with cycles of tidal inundation and drying combined with freshwater seepage, rainfall, and run-off in the upper intertidal zone. These interacting processes produce dynamic fine-scale hydrological and salinity gradients, which may drive transformation to intertidal forests (MFT1.2). Marshes are associated with low-energy depositional coasts but may occur on sea cliffs and headlands where wind deposits salt from wave splash (i.e. salt spray) and aerosol inputs. Salt approaches hypersaline levels where flushing events are infrequent. Other nutrients make up a low proportion of the total ionic content. Subsoils are generally anaerobic, but this varies depending on seepage water and the frequency of tidal inundation. Tidal cycles also influence temperature extremes, irregularities in photoperiod, physical disturbance, and deposition of sediment.

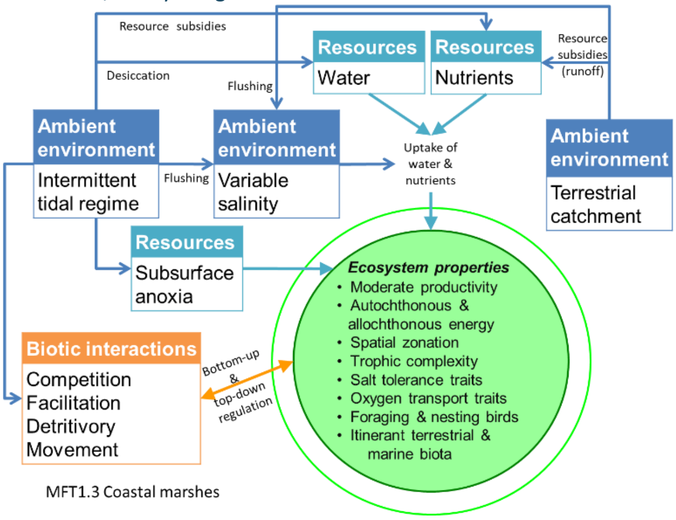

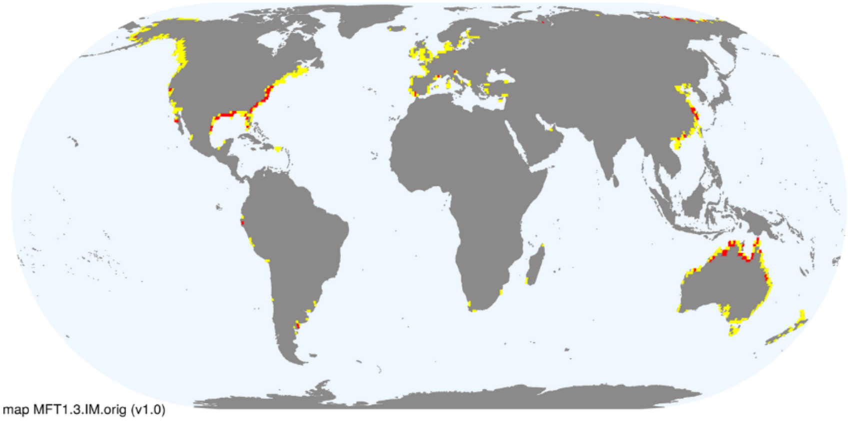

map MFT1.3.IM.orig (v1.0)

Distribution: Widely distributed, mostly on low-energy coasts from arctic to tropical and subantarctic latitudes.

References:

Adam P (1990) *Saltmarsh ecology* Cambridge University Press, Cambridge.

Bertness MD, Shumway SW (1993) Competition and facilitation in marsh plants. *American Naturalist* 142, 718-724.

Jefferies RL, Jano AP and Abraham KF (2006) A biotic agent promotes large-scale catastrophic change in the coastal marshes of Hudson Bay. *Journal of Ecology* 94, 234–242.

Map is for illustrative purposes only and does not support spatial analyses unless formally validated.
