# Supplementary material for: A function-based typology for Earth’s ecosystems
Source: Nature. 2022 Oct 12;610(7932):513–8. doi: 10.1038/s41586-022-05318-4 (PMC9581774; doi:10.1038/s41586-022-05318-4)
Supplement: Supplementary file 9 — Applications of the IUCN Global Ecosystem Typology. A review of the role of the typology in applications including reporting on global conservation and sustainability goals and targets, natural capital accounting, ecosystem risk assessment, identification of key biodiversity areas, conservation planning ecosystem monitoring, comparative ecosystem research and environmental education. [file 41586_2022_5318_MOESM9_ESM.pdf]

Appendix S6. Applications of the IUCN Global Ecosystem Typology

'*A function-based typology for Earth's ecosystems*'

David A. Keith, Jose R. Ferrer-Paris, Emily Nicholson, Melanie J. Bishop, Beth A. Polidoro, Eva Ramirez-Llodra, Mark G. Tozer, Jeanne L. Nel, Ralph Mac Nally, Edward J. Gregr, Kate E. Watermeyer, Franz Essl, Don Faber-Langendoen, Janet Franklin, Caroline E. R. Lehmann, Andres Etter, Dirk J. Roux, Jonathan S. Stark, Jessica A. Rowland, Neil A. Brummitt, Ulla C. Fernandez-Arcaya, Iain M. Suthers, Susan K. Wiser, Ian Donohue, Leland J. Jackson, R. Toby Pennington, Thomas M. Iliffe, Vasilis Gerovasileiou, Paul Giller, Belinda J. Robson, Nathalie Pettorelli, Angela Andrade, Arild Lindgaard, Teemu Tahvanainen, Aleks Terauds, Michael A. Chadwick, Nicholas J. Murray, Justin Moat, Patricio Pliscoff, Irene Zager, Richard T. Kingsford

Nature 2022

Introduction

Our ecosystem assembly model and global typology will support multi-disciplinary action on ecosystem governance, management and conservation, sustainable development, communication and education around the world. The typology has built-in versatility for a range of applications related to ecosystem functioning and biodiversity, because:

i) the scalable hierarchy enables representation of different features at particular hierarchical levels and facilitates applications across a range of spatial and organisational scales (see Rationale in Table S1.1); and
ii) the typology represents both ecosystem functions (in the upper levels) and biodiversity (in the lower levels).

Here we identify eight groups of applications that would benefit from the IUCN Global Ecosystem Typology (Fig. 3 in main text). All of these applications require either a function-based framework for ecosystem assessment (global analysis of Sustainable Development Goals, and ecosystem accounts), assemblages of biodiversity above the species level (e.g. landscape/seascape conservation planning, representation analyses of protected areas) or both (e.g. Red List of Ecosystems risk assessments). This diversity of applications is recognised in Resolution 7.061 *Partnerships and further development of a Global Ecosystem Typology* (https://portals.iucn.org/library/fr/node/49200), adopted at the 2020 World Conservation Congress, in which the IUCN endorses "applying the Global Ecosystem Typology to support global, regional and national efforts to assess and manage risks to ecosystems". The diverse themes and sales of these applications suggest that the IUCN Global Ecosystem Typology will provide a much-needed information infrastructure to address dual overarching goals to conserve biodiversity and sustain ecosystem services.

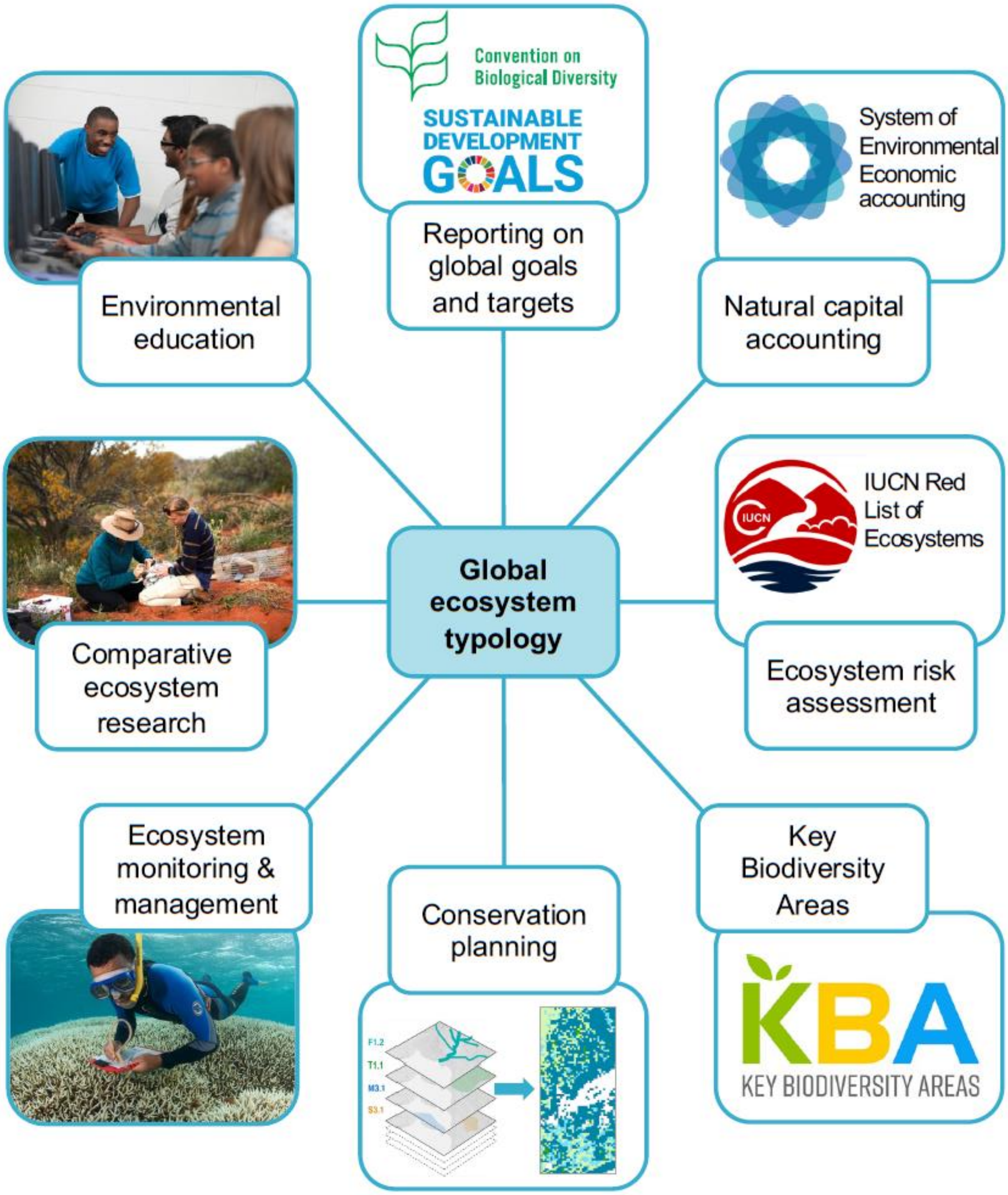

Figure 4 [reproduced from main text]. Current and potential applications of the Global Ecosystem Typology to conserve biodiversity and sustain ecosystem services. Policy links exist between multiple initiatives (not shown), where the typology allows consistency in the treatment of ecosystems across applications.

Reporting on global goals and targets

Global environmental reporting mechanisms established under international agreements include the United Nations Sustainable Development Goals (SDGs), the Intergovernmental Science-Policy Platform on Biodiversity and Ecosystem Services (IPBES), the Aichi Targets of the Strategic Plan for Biodiversity (2011-2020) (CBD 2010), and its successor, the post-2020 global biodiversity framework (OWEG 2021a), under the Convention on Biological

Diversity (CBD). The IUCN Global Ecosystem Typology has direct relevance to each of these global policy initiatives.

The IUCN Global Ecosystem Typology was recommended as the methodological basis for headline indicators for ecosystems in the draft monitoring framework for the post-2020 global biodiversity framework (OWEG 2021b), across marine, freshwater and terrestrial ecosystem types. It will enable national data to be scaled up for global synthesis, and to structure reporting in a manner that reflects trends in ecosystem biodiversity, functions and services (Nicholson et al. 2021), including via recently developed indicators that report on ecosystem extent, health and overall risk (Rowland et al. 2020). The typology will also support disaggregation of global data by biome and ecosystem functional group, enabling deeper understanding of global patterns in biodiversity loss. This will inform effective action to mitigate impacts and target restoration planning (Etter et al. 2020).

Similarly, the typology will directly support implementation of SDGs 14 (Life in the oceans) and 15 (Life on land), and indirectly contribute to work on SDGs 4 (Quality education), 6 (Clean water and sanitation), 11 (sustainable cities and communities) and 13 (Climate action), by structuring and synthesising information about change in biomes and EFGs. The Global Ecosystem Typology also has potential to support future IPBES global assessments of biodiversity and the services by providing a consistent framework for comparison between, and synthesis across regional analysis. The lack of indicators for synthesising the state of ecosystems, in particular their degradation, was highlighted as a knowledge gap in the 2019 global assessment (IPBES 2019).

Natural capital accounting

In 2012 the United Nations adopted a System for Environmental Economic Accounts (SEEA) as an international statistical standard for quantifying stocks and flows of natural resources (Obst & Vardon 2014). In 2021, after an extensive consultation and development phase, this approach was extended to ecosystems when the United Nations adopted the SEEA-Ecosystem Accounting (SEEA-EA) as the global statistical standard for ecosystem accounting (UNCEEA 2021). SEEA-EA standards quantify ecosystem extent, condition, services and benefits, linking ecosystems to human benefits (Hein et al. 2015). SEEA-EA explicitly considers the spatial diversity of ecosystems and the interconnectedness of ecosystem components, and thus a comprehensive set of ecosystem spatial units is a key requisite for ecosystem accounting. National implementation has risen steadily since 2012, with an estimated that 91 countries compiling SEEA accounts by early 2020 (CBD 2020). By the end of 2019, 24 countries had published ecosystem accounts under the Experimental Ecosystem Accounting programme.

The IUCN Global Ecosystem Typology was adopted as the reference classification for implementing SEEA-EA (UNCEEA 2021). The core of the reference classification is Level 3, which will be used to summarise globally across national ecosystem accounts. Contributors to SEEA-EA are expected to use the best quality high-resolution classification and maps available for their jurisdiction when developing their national accounts (i.e. Level 6 units), and assign the units of that classification to Ecosystem Functional Groups (Level 3) to enable consistent international reporting. Examples linking fine-grained national classifications to Level 3 of the IUCN typology are given in Tables S3.3 and S3.4. This flexible use of the IUCN typology will enable jurisdictions to report detailed national accounts, while contributing data that can be scaled up and summarised to units of the international reference classification (UNSD 2019). For countries that currently lack a national classification for

ecosystem accounting, the IUCN Global Ecosystem Typology may be used to develop one. Murray et al. (2020) provide a recent example of using the Global Ecosystem Typology to scale down to locally-derived, locally-relevant ecosystem types in Myanmar.

When structured by Level 3 of the Global Ecosystem Typology, national and global ecosystem accounts should quantify major functional shifts and re-organisation of ecosystems. For example, transformation of the Aral Sea during 1980-2000 (Micklin 2006) from a large freshwater body (EFG F2.1) to ephemeral grasslands (degraded forms of T5.1) and hypersaline water bodies (F2.6), can be readily tracked at Level 3 of the typology. While we acknowledge uncertainties in ecosystem identification, such major shifts between groups should be relatively easy to detect and have major implications for human wellbeing and biodiversity. Other increasingly common types of ecosystem transformation are also readily detected and reportable through Level 3 of the typology. These include transitions driven by land use change (T1-T6 to T7, or T7.2 to T7.3), changes in fire regimes (e.g. T1 to T4), or climate-related shifts such as drying of freshwater aquatic systems (various transitions involving F1, F2 or FT1), advance of alpine treelines (e.g. T6.4 to T2.1) or repeated coral bleaching and ocean acidification (M1.3 to M1.6 or M1.7). Conversely, shifts that result from forest reconstruction (e.g. T7.2 to T2.2) or land use abandonment (e.g. T7.5 to T2.2) should also be detectable and reportable as shifts between different Ecosystem Functional Groups.

The upper levels of the typology should be robust to compositional changes that do not produce major functional shifts. However, more subtle shifts reshuffling of species composition, for example, in response to climate change or land degradation, could be represented at lower levels of the typology in national-level accounts (with consequent uncertainties in diagnosis); and upscaled to changes in global condition accounts.

More broadly, one approach to sustaining ecosystem services is to identify, protect and manage the ecosystems that provide them (Bordt & Saner 2019). The Global Ecosystem Typology offers a consistent and comprehensive framework incorporating ecosystem functions as basis for attributing ecosystem services to ecosystem types. We suggest that such an approach is likely to be more informative for ecosystem management than land cover or land use categories (Di Gregorio & Jansen 2000). Land use/land cover classification can provide detailed treatment of land use types, but tend to lump functionally contrasting ecosystems into broad structural groupings such as forest, grassland and 'unvegetated'. Attribution of services to ecosystem units could be done at any level of the IUCN Global Ecosystem Typology depending on needs.

Ecosystem risk assessment: A global Red List of Ecosystems

The Red List of Ecosystems (RLE) protocol (Keith et al. 2013) was adopted as the international standard for ecosystem risk assessment by IUCN Council in 2014 (Keith et al. 2015), following World Conservation Congress 2012 Resolution 5.055 *Consolidation of the IUCN Red List of Ecosystems* (IUCN 2012). Since then, there has been a substantial policy impact, primarily through systematic and strategic RLE assessments at the national level (Bland et al. 2019). The Global Ecosystem Typology provides a framework for comparison and synthesis of national and regional assessments, which were based on different local classifications representing Level 6 of the typology (Subglobal ecosystem types). Level 3 of the typology (Ecosystem Functional Groups) is being used as a template for developing national ecosystem classifications where none currently exist (e.g. Murray et al. 2020).

The first global Red List of Ecosystems is planned for completion by 2025 (Rodriguez et al. 2015). One of the key motivations for development of the Global Ecosystem Typology was

to define the assessment units for this analysis (Keith et al. 2015). Level 4 of the typology (Regional ecosystem subgroups) will be used as proxy assessment units (Fig. 2 in main text; Appendix S3), while Level 5 units (Global ecosystem types) will be developed over time from the bottom-up as a basis for future assessments. The pivotal role of the typology in advancing Red Lists of Ecosystems is explicitly recognised in World Conservation Congress Resolution 61 - *Partnerships and further development of a Global Ecosystem Typology* (IUCN 2020).

Ecosystem-specific application of risk assessment protocols, such as the Red List of Ecosystems criteria, provide an intermediate means of risk assessment that avoid some significant limitations of generic composite indices of pressures, yet are less data-demanding than stochastic process models. The IUCN Global Ecosystem Typology provides guidance for defining the units for such assessments because of its representation of both functions and biodiversity at different levels.

Key Biodiversity Areas

A global standard for identification of Key Biodiversity Areas (KBAs) was published by IUCN (2016). Under this standard, protection a of site may make a 'key' contribution to conserving biodiversity if it contains a large representation of one or more threatened ecosystem types or of any ecosystem types that are restricted in their occurrence elsewhere (IUCN 2016). In order to meet criterion A2 of the criteria for identification as a Key Biodiversity Area, an area must contain a minimum proportion of the total global extent of a globally threatened ecosystem type. Criterion B4 specifies a requirement for representation of at least 20% of the global extent of any ecosystem type. The Global Ecosystem Typology and its associated spatial data are essential to assess KBA criterion A2 and would greatly assist assessments of criterion B4.

Conservation Planning

The Global Ecosystem Typology should help to broaden the focus of conservation planning on land and sea beyond the identification of Key Biodiversity Areas and protection of individual threatened species. Functionally-defined, high-order units of biodiversity are likely to provide more faithful surrogates for lower levels of biodiversity than biophysical units or umbrella species groups (Rodrigues & Brooks 2007) because *a priori* they represent ecological processes that govern the assembly of biological communities (Fig. 1 in main text; Appendix S2). Moreover, integration of conservation targets for both ecosystems and species improves the efficiency and effectiveness of conservation outcomes than planning for those targets separately (Polak et al. 2015).

Evidence is emerging for early uptake of our IUCN Global Ecosystem Typology in conservation planning applications at national levels. Murray et al. (2020) used our typologial framework to develop a national classification and map of ecosystems for conservation planning in Myanmar to assess risks to different ecosystem types, which is now being used to identify Key Biodiversity Areas and priorities for expanding the network of protected areas in that country. It will also be used to structure Myanmar's next National Biodiversity Strategy and Action Plan for reporting to Convention Biological Diversity.

Etter et al. (2020) used outcomes of Red List of Ecosystem assessments to develop national priorities for ecosystem restoration in Colombia. The national ecosystem types considered in that study are now integrated into Level 6 of the Global Ecosystem Typology, facilitating knowledge transfer to and from restoration practitioners working in functionally related

ecosystems in other countries. Further applications to planning for ecosystem restoration are examined by Valderrábano et al. (2021).

The Global Ecosystem Typology could also help conservation planners to extend the focus of spatial prioritisation for conservation by setting targets for functional groups of ecosystems that make important contributions to sustaining to ecosystem functions and highly valued ecosystem services (Bordt & Saner 2019).

In a policy context, a range of regulatory instruments and incentive schemes exist to protect or restore ecosystems across different countries and subnational jurisdictions (Alaniz et al. 2019). Indeed, many of these are already in place in some countries (Bland et al. 2019). The Global Ecosystem Typology will help to improve priorities to identify the most appropriate policy options to sustain different functional groups of ecosystems in particular socio-political settings. It will also support policy clarity for practitioners who need to identify and manage particular types of ecosystems on the ground.

Ecosystem monitoring and management

The hierarchical structure of the IUCN Global Ecosystem Typology should facilitate stronger links between global imperatives for conservation and sustainability, and local management actions that seek to address them. The typology supports ecosystem management applications, including problem diagnosis and design of locally relevant solutions, because the units of classification are broadly recognisable and functionally relevant on the ground. As well, thorough descriptions of biomes and Ecosystem Functional Groups (Appendix S4), and advanced resources for user support (https://global-ecosystems.org/) serve as enablers for local action. Models of dynamics and assembly for should inform the selection of indicators and design of monitoring for different ecosystem types in the same functional group (Bland et al. 2018).

Grouping ecosystems that share common mechanisms of response to environmental change, anthropogenic threats and remedial management actions (Fig. S6.1) establishes a powerful basis for adaptive management to reduce risks of ecosystem collapse (Keith et al. 2011; Williams 2011). Moreover, such a classificatory system provides a framework for information storage and retrieval and knowledge transfer by drawing attention to experience on nature-based solutions for managing functionally similar ecosystems (Keesstra et al. 2018; Cohen-Shacham et al. 2019).

Seagrass meadows (Ecosystem Functional Group M1.1) provide a specific example. Seagrass ecosystems are globally distributed, but share similar threats from intensifying levels of shipping and boating with associated turbulence, turbidity and uprooting of the structural dominants by anchors of recreational and commercial vessels. Alternative techniques for translocation and establishment, new designs of boat moorings that lift chains above the seafloor, and cost-effective citizen science initiatives to assist restoration of degraded meadows are developing with applications by local authorities in Australia and New Zealand (Tan et al. 2020). By promoting further thematic reviews and collaborations on functionally related ecosystems, the typology can support local managers in other regions by facilitating transfer of knowledge and helping them to justify investment and implementation of effective strategies for ecosystems exposed to similar threats. Tan et al. (2020) not only provide alternative seagrass management and restoration options for consideration, but they review available evidence on what works where, providing local decision makers with informed choices about how to tackle management problems in their local settings.

More broadly, at the outset of the UN Decade of Ecosystem Restoration, new approaches applying risk assessment science to ecosystem restoration are under development (Valderrábano et al. 2021). The Global Ecosystem Typology is recognised as a useful tool for framing consistent descriptions of ecosystems when planning restoration priorities and strategies for risk reduction (Nicholson 2021; Etter et al. 2021), and for knowledge transfer on ecosystems with similar restoration requirements around the world, building capacity and increasing a shared global understanding (Nelson 2021).

Comparative ecosystem research

The Global Ecosystem Typology will support research efforts by providing a framework and common language for comparison and generalisation among ecosystems (Fig. S6.1). Descriptive profiles (Appendix S4) should provide sufficient detail for researchers and others to identify which Ecosystem Functional Groups their study systems belong to, providing a more systematic and robust means of extending research hypotheses and findings across ecosystem ecology, evolutionary biogeography, ecological economics, socio-ecology and other disciplines. Common concepts and terminology should help to avoid misinterpretations caused in part by local nomenclatural traditions, such as those that hampered understanding of global and regional distributions of dry forests and savannas (Ratnam et al. 2011; Pennington et al. 2018). The typology could also draw attention to knowledge gaps for major functional groups of ecosystems that have received little research attention (Ramirez –Llodra et al. 2010; Edwards et al. 2012; Anesio & Laybourn-Parry 2012; Riosmena-Rodríguez et al. 2017).

Environmental education

Our Global Ecosystem Typology provides both a framework and a resource to foster learning about the diversity of both natural and anthropogenic ecosystems on Earth, the intrinsic values of nature and how ecosystems support human well-being. These areas of learning are critical to Sustainable Development Goal 4 ‘Quality education’ and Aichi target 1 to promote awareness of the values of biodiversity and the steps people can take to conserve and use it sustainably (CBD 2010). Exposure to inspirational content on nature is influential in motivating positive behavioural changes and environmental outcomes, and especially formative in childhood (Chawla 1999; Kellert 2005; Bekessy et al. 2018).

We are developing professionally presented online resources and smart-phone apps to help people recognise their local ecosystems in a global context, and connect them with nature locally and abroad. We also plan to support illustrated reference material for schools and other educators. This will fill a significant gap, as presently there is no central point of reference that presents the full range of ecosystems on Earth in a widely accessible style.

**Realm**<br>**Biome**<br><br>**Ecosystem Functional Group**	F Freshwater<br>F2 Lakes<br><br>F2.1 Large permanent freshwater lakes	T Terrestrial<br>T2 Temperate-boreal forests & woodlands<br>T2.5 Temperate pyric humid forests	M Marine<br>M1 Marine shelves<br><br>M1.2 Kelp forests
**Archetype**	Aral Sea<br>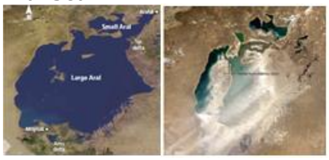	Mountain Ash forests<br>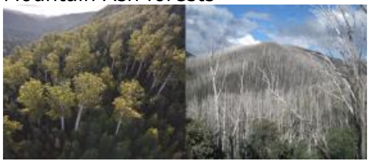	Alaskan kelp forests<br>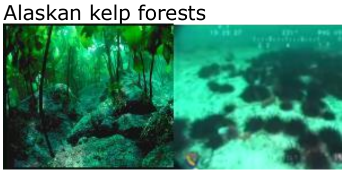
**Key processes**	Water volume-chemistry interactions & trophic web	Vegetation-fire feedbacks, trees as foundation species	Trophic web, interactions with pelagic system, foundation species
**Key threats**	Water extraction	Logging & fire regime shift	Exploitation of marine mammals & fish
**Functionally similar ecosystem types**	Lake Chad, Salton Sea	Alpine ash, Blue mtns ash, karri forests	Patagonian kelp forests, NZ kelp forests, Tasmanian kelp forests
**Trans-formations to anthromes**	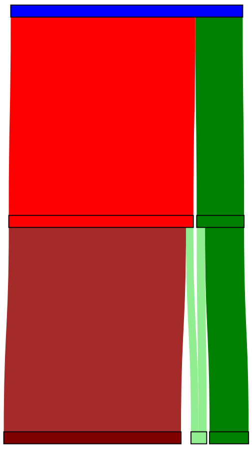	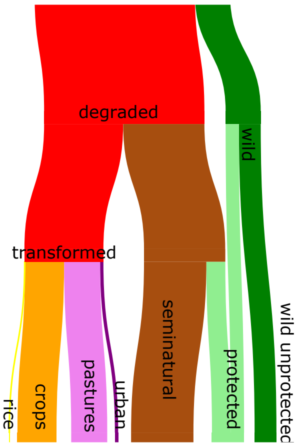	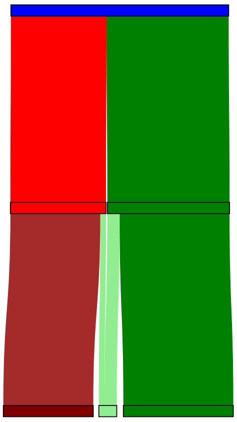

Figure S6.1. Schematic illustrating how the global ecosystem typology, specifically, Ecosystem Functional Groups, can support generalisations, predictions and knowledge transfer for ecosystem management and comparative research. Further details on the examples are given in Appendix S2 of Keith et al. (2013) and Burns et al. (2015).

References

Alaniz AJ, Perez-Quezada JF, Galleguillos M, Vasquez AE, **Keith, D A** (2019) Operationalizing the IUCN Red List of Ecosystems in public policy. *Conservation Letters*, e12665. [doi: 10.1111/conl.12665]

Anesio AM, Laybourn-Parry J (2012) Glaciers and ice sheets as a biome. Trends in Ecology and Evolution 27: 219-225.

Bekessy SA, Runge MC, Kusmanoff AM, Keith DA, Wintle BA (2018) Ask not what nature can do for you: A critique of ecosystem services as a communication strategy. Biological Conservation 224: 71-74.

Bland LM, Nicholson E, Miller RM, Andrade A, Carré A, Etter A, Ferrer-Paris JR. Herrera B, Kontula T, Lindgaard A. Pliscoff P, Skowno A, Valderrábano M, Zager I, Keith DA (2019) Impacts of the IUCN Red List of Ecosystems on conservation policy and practice. Conservation Letters: e12666 [doi: 10.1111/conl.12666]

Bland LM, Watermeyer KE, Keith DA, Nicholson E, Regan TJ, Shannon LJ (2018) Assessing risks to marine ecosystems with indicators, ecosystem models and experts. Biological Conservation 227: 19-28.

Bordt M, Saner MA (2019) Which ecosystems provide which services? A meta-analysis of nine selected ecosystem services assessments. One Ecosystem 4: e31420. [doi:10.3897/oneeco.4.e31420]

Burns EL, Lindenmayer DB, Stein J, Blanchard W, McBurney L, Blair D, Banks SC (2015) Ecosystem assessment of mountain ash forest in the Central Highlands of Victoria, south-eastern Australia. Austral Ecology 40: 386–399.

CBD (2010) Strategic Plan for Biodiversity 2011-2020, and the Aichi Biodiversity Targets: Living in harmony with nature. United Nations, New York. [https://www.cbd.int/doc/strategic-plan/2011-2020/Aichi-Targets-EN.pdf, downloaded 21/09/2019].

CBD (2020) Global Biodiversity Outlook 5. Secretariat of the Convention on Biological Diversity Montreal. [https://www.cbd.int/gbo/gbo5/publication/gbo-5-en.pdf]

Chawla L (1999) Life paths into effective environmental action. Journal of Environmental Education 31:15–26.

Cohen-Shacham E, Andrade A, Dalton J, Dudley N, Jones M, Kumar C, Maginnis S, Maynard S, Nelson CR, Renaud FG, Welling R, Walters G (2019) Core principles for successfully implementing and upscaling Nature-based Solutions. Environmental Science & Policy 98: 20-29. [https://doi.org/10.1016/j.envsci.2019.04.014]

Di Gregorio A, Jansen LJM (2000) Land cover classification system (LCCS): Classification concepts and user manual. United Nations Food and Agriculture Organization (FAO), Rome.

Edwards KJ, Becker K, Colwell F (2012). The deep, dark energy biosphere: intraterrestrial life on earth. Annual Review of Earth and Planetary Sciences 40, 551–568.

Etter A, Andrade A, Nelson CR, Cortés J, Saavedra K (2020) Assessing restoration priorities for high-risk ecosystems: An application of the IUCN red list of ecosystems. Land Use Policy 99: 104874.

Etter A, Hallett JG, McBreen J. Nelson CR (2021) Using ecosystem risk assessment to guide strategic restoration planning. In: Using ecosystem risk assessment science in ecosystem restoration: A guide to applying the Red List of Ecosystems to ecosystem restoration (Eds M Valderrábano, C Nelson, E Nicholson, A Etter, J Carwardine, JG Hallett, J McBreen, E Botts), pp 31-50. IUCN, Gland.

IPBES (2019). Global assessment report on biodiversity and ecosystem services of the Intergovernmental Science-Policy Platform on Biodiversity and Ecosystem Services. IPBES Secretariat. Bonn, Germany.

IUCN (2012) Consolidation of the IUCN Red List of Ecosystems. Resolution 5.055. World Conservation Congress 2012. IUCN, Gland. [https://portals.iucn.org/library/node/44022]

IUCN (2016) A global standard for the identification of Key Biodiversity Areas, Version 1.0. First edition. IUCN, Gland. [https://portals.iucn.org/library/sites/library/files/documents/2016-048.pdf]

IUCN (2020) Partnerships and further development of a Global Ecosystem Typology. Resolution 7.061. World Conservation Congress 2020. IUCN, Gland. [https://portals.iucn.org/library/fr/node/49200]

Keesstra S, Nunes J, Novara A, Finger D, Avelar D, Kalantari Z, CerdaA (2018) The superior effect of nature based solutions in land management for enhancing ecosystem services. Science of the Total Environment 610: 997-1009. [doi: 10.1016/j.scitotenv.2017.08.077]

Keith DA, Martin TG, McDonald-Madden E, Walters C (2011) Uncertainty and adaptive management for biodiversity conservation. Biological Conservation 144: 1175–1178.

Keith DA, Rodríguez JP, Rodríguez-Clark KM, Nicholson E, Aapala K, Alonso A, Asmussen M, Bachman S, Basset A, Barrow EG, Benson JS, Bishop MJ, Bonifacio R, Brooks TM, Burgman MA, Comer P, Comín FA, Essl F, Faber-Langendoen D, Fairweather PG, Holdaway RJ, Jennings M, Kingsford RT, Lester RE, Mac Nally R, McCarthy MA, Moat J, Oliveira-Miranda MA, Pisanu P, Poulin B, Regan TJ, Riecken U, Spalding MD, Zambrano-Martínez S (2013) Scientific foundations for an IUCN Red List of Ecosystems. PLoS ONE 8(5), e62111.

Keith DA, Rodríguez JP, Brooks TM, Burgman MA, Barrow EG, Bland L, Comer PJ, Franklin J, Link J, McCarthy MA, Miller RM, Murray NJ, Nel J, Nicholson E, Olivera-Miranda MA, Regan TJ, Rodríguez-Clark KM, Rouget M Spalding MD (2015). The IUCN Red List of Ecosystems: motivations, challenges and applications. Conservation Letters 8: 214-226. [doi: 10.1111/conl.12167]

Kellert SR (2005) Building for life: Designing and understanding the human-nature connection. Island Press, Washington DC.

Micklin P (2006) The Aral Sea crisis and its future: An assessment in 2006. Eurasian Geography and Economics 47: 546–567.

Murray NJ, Keith DA, Tizard R, Duncan A, Win Htut T, Hlaing N, Htat N, Oo AH, Zay Ya K, Grantham H (2020) Threatened Ecosystems of Myanmar. An IUCN Red List of Ecosystems Assessment. Version 1.0. Wildlife Conservation Society, Yangon. ISBN: 978-0-9903852-5-7. https://www.myanmar-ecosystems.org/

Nelson CR (2021) Monitoring the effects of restoration activities on ecosystem area, integrity and risk of collapse. In: Using ecosystem risk assessment science in ecosystem restoration: A guide to applying the Red List of Ecosystems to ecosystem restoration (Eds M Valderrábano, C Nelson, E Nicholson, A Etter, J Carwardine, JG Hallett, J McBreen, E Botts), pp 61-74. IUCN, Gland.

Nicholson E (2021) Identifying which ecosystems are most at risk using the IUCN Red List of Ecosystems. In: Using ecosystem risk assessment science in ecosystem restoration: A guide to applying the Red List of Ecosystems to ecosystem restoration (Eds M Valderrábano, C Nelson, E Nicholson, A Etter, J Carwardine, JG Hallett, J McBreen, E Botts), pp 17-29. IUCN, Gland.

Nicholson E, Watermeyer KE, Rowland JA, Sato CF, Stevenson SL, Andrade A, Brooks TM, Burgess ND, Cheng S.-T, Grantham H, Hill SL, Keith DA, Maron M, Metzke D, Murray NJ, Nelson CR, Obura D, Plumptre A, Skowno AL,Watson JEM (2021) Scientific foundations

for an ecosystem goal, milestones and indicators for the post-2020 global biodiversity framework Nature Ecology and Evolution, in press. [doi: 10.1038/s41559-021-01538-5]

Obst C, Vardon M (2014) Recording environmental assets in the national accounts. Oxford Review of Economic Policy 30: 126–144. [https://doi.org/10.1093/oxrep/gru003]

OEWG (2021a) First draft of the Post 2020 Global Biodiversity Framework. CBD/WG2020/3/3, Open-Ended Working Group on the Post-2020 Global Biodiversity Framework. Convention on Biological Diversity. United Nations, New York. https://www.cbd.int/doc/c/abb5/591f/2e46096d3f0330b08ce87a45/wg2020-03-03-en.pdf

OEWG (2021b) Proposed headline indicators of the monitoring framework for the Post-2020 Global Biodiversity Framework, CBD/WG2020/3/3/Add.1. Open-Ended Working Group On The Post-2020 Global Biodiversity Framework. https://www.cbd.int/doc/c/d716/da69/5e81c8e0faca1db1dd145a59/wg2020-03-03-add1-en.pdf

Pennington RT, Lehmann CER, Rowland LM (2018) Tropical savannas and dry forests. Current Biology 28: R527–R548.

Polak T, Watson JEM, Fuller RA, Joseph LN, Martin TG, Possingham HP, Venter O, Carwardine J (2015) Efficient expansion of global protected areas requires simultaneous planning for species and ecosystems. Royal Society Open Science 2:150107. [http://dx.doi.org/10.1098/rsos.150107]

Ramirez-Llodra E, Brandt A, Danovaro R, De Mol B, Escobar E, et al. (2010) Deep, diverse and definitely different: unique attributes of the world's largest ecosystem. Biogeosciences 7: 2851–2899.

Ratnam J, Bond WJ, Fensham RJ, Hoffmann WA, Archibald S, Lehmann CER, Anderson MT, Higgins SI, Sankaran1 M (2011) When is a 'forest' a savanna, and why does it matter? Global Ecology and Biogeography 20: 653–660. [doi: 10.1111/j.1466-8238.2010.00634.x]

Riosmena-Rodríguez R, Nelson W, Aguirre J (2017) Rhodolith/Maërl beds: A Global perspective. Springer, Cham. [https://doi.org/10.1007/978-3-319-29315-8]

Rodrigues ASL, Brooks TM (2007) Shortcuts for biodiversity conservation planning: The effectiveness of surrogates. Annual Review of Ecology Evolution and Systematics 38: 713-737. [doi: 10.1146/annurev.ecolsys.38.091206.095737]

Rodríguez JP, Keith DA, Rodríguez-Clark KM, Murray NJ, Nicholson E, Regan TJ, Miller RM, Barrow EG, Bland LM, Boe K, Brooks TM, Oliveira-Miranda MA, Spalding M, Wit P (2015). A practical guide to the application of the IUCN Red List of Ecosystems criteria. Philosophical Transactions of the Royal Society B 370, 20140003 [doi: 10.1098/rstb.2014.0003]

Rowland JA, Bland LM, Keith DA, Juffe-Bignoli D., Burgman MA, Etter A, Ferrer-Paris JR, Miller RM, Nicholson E (2019) Ecosystem indices to support global biodiversity conservation. Conservation Letters 13(1): e12680.

UNSD (2019) System of Environmental-Economic Accounting 2012– Experimental Ecosystem Accounting. Chapter 4. Accounting for ecosystem extent. Version 1.0. United Nations Statistics Division, New York.

Tan YM, Dalby O, Kendrick GA, Statton J, Sinclair EA, Fraser MW, Macreadie PI, Gillies CL, Coleman RA, Waycott M, van Dijk KJ, Verges A, Ross JD, Campbell ML, Matheson FE, Jackson EL, Irving AD, Govers LL, Connolly R, McLeod IM, Rasheed MA, Kirkman H,

Flindt MR, Lange T , Miller AD, Sherman CD (2020) Seagrass restoration is possible: Insights and lessons from Australia and New Zealand. Frontiers in Marine Science 7: 617. [10.3389/fmars.2020.00617]

UNCEEA (2021) System of Environmental-Economic Accounting—Ecosystem Accounting. UN Committee of Experts on Environmental-Economic Accounting (UNCEEA), United Nations Statistical Division, New York.

Valderrábano M, Nelson C, Nicholson E, Etter A, Carwardine J, Hallett J, McBreen J, IUCN, Botts E (2021) Restoring ecosystems using risk-assessment science: A guide to applying the Red List of Ecosystems to ecosystem restoration. IUCN, Gland.

Vardon M, Keith H, Obst C, Lindenmayer D (2019) Putting biodiversity into the national accounts: Creating a new paradigm for economic decisions. Ambio 48: 726–731. [doi.org/10.1007/s13280-018-1114-z]

Williams BK (2011) Adaptive management of natural resources--framework and issues. Journal of Environmental Management 92:1346-1353. [doi: 10.1016/j.jenvman.2010.10.041]
