# Supplementary material for: A function-based typology for Earth’s ecosystems
Source: Nature. 2022 Oct 12;610(7932):513–8. doi: 10.1038/s41586-022-05318-4 (PMC9581774; doi:10.1038/s41586-022-05318-4)
Supplement: Supplementary file 8 — Development, review and revision process for the IUCN Global Ecosystem Typology. A full description of the review peer review processes undertaken during typology development. Tabulation of all reviewer comments and detailed responses of authors for all 110 ecosystem functional groups in level 3 of the typology. [file 41586_2022_5318_MOESM8_ESM.pdf]

Appendix S5. Development, review and revision process for the IUCN Global Ecosystem Typology

'*A function-based typology for Earth's ecosystems*'

David A. Keith, Jose R. Ferrer-Paris, Emily Nicholson, Melanie J. Bishop, Beth A. Polidoro, Eva Ramirez-Llodra, Mark G. Tozer, Jeanne L. Nel, Ralph Mac Nally, Edward J. Gregr, Kate E. Watermeyer, Franz Essl, Don Faber-Langendoen, Janet Franklin, Caroline E. R. Lehmann, Andres Etter, Dirk Roux, Jonathan S. Stark, Jessica A. Rowland, Neil A. Brummitt, Ulla C. Fernandez-Arcaya, Iain M. Suthers, Susan K. Wiser, Ian Donohue, Leland J. Jackson, R. Toby Pennington, Thomas M. Iliffe, Vasilis Gerovasileiou, Paul Giller, Belinda J. Robson, Nathalie Pettorelli, Angela Andrade, Arild Lindgaard, Teemu Tahvanainen, Aleks Terauds, Michael A Chadwick, Nicholas J. Murray, Justin Moat, Patricio Pliscoff, Irene Zager, Richard T. Kingsford

Nature 2022

Introduction

Rapidly developing information infrastructure to support global policy initiatives for conservation and sustainable management of ecosystems under the forthcoming post-2020 for the United Nations Convention on Biological Diversity include the UN System of Environmental-Economic Accounting – Experimental Ecosystem Accounting (SEEA EEA), listing criteria for the IUCN Red List of Ecosystems (RLE) and Key Biodiversity Areas (KBA). These and other initiatives (Appendix S7) require a standardised, globally consistent, spatially explicit typology and terminology for managing the world's ecosystems and their services. Consequently, a motion is before the World Conservation Congress 2020, "*acknowledging the scientific advances in developing comprehensive and practical Global Ecosystem Typology (GET) and the importance for comprehensive data on the status of the world's ecosystems to monitor and assess changes in that status*"; and "*to promote and support Members … in applying the GET to support global, regional and national efforts to assess and manage risks to ecosystems*" (https://www.iucncongress2020.org/motion/074).

Development of the typology

IUCN Commission on Ecosystem Management initiated and co-ordinated through an extensive development and review process for the Global Ecosystem Typology. The initial structure of the typology was developed at a forum supported by the PLuS Alliance of universities (https://www.plusalliance.org/ at Kings College London in May 2017. The forum, attended by 48 specialists in marine, freshwater and terrestrial ecosystems, agreed on a draft hierarchical structure for the typology and established provisional lists of units for the top three levels of the hierarchy. In deciding how to subdivide realms, and then biomes, working group members drew on the literature (a sample of which is cited in the profiles), their specialist research experience

in respective ecosystems and that of their research networks. Lead contributors among the workshop participants then drafted descriptive profiles for each Ecosystem Functional Group in consultation with working group members and other experts. The definition and arrangement of units within the typological hierarchy was iteratively reviewed and adapted by workshop participants as the profiles were prepared (out of session, post-workshop) to ensure coverage of the range of variation in ecosystems throughout the biosphere.

Review and revision of the typology

The IUCN Commission on Ecosystem Management co-ordinated an extensive global consultation, review, testing and revision process in four phases. The first phase of consultation involved presentations and interactive discussion and feedback at a series of international meetings. These presentations described the objectives, conceptual basis and structure of the typology to elicit input on those aspects and to engage participants out of session in author teams to refine the description of the units. The 18 presentations and associated discussions were held around the world at dedicated interactive workshops arranged by IUCN Commission on Ecosystem Management and international conference symposia, each attended by 20-100 participants, at the following locations and dates:

London, UK May 2017– IUCN workshop/meeting
Cartagena, Colombia, July 2017 – IUCN workshop/meeting
Cartagena, Colombia, July 2017 – ICCB symposium
Naypyidaw, Myanmar, Sept 2017 – IUCN workshop/meeting
Newcastle, Australia, November 2017 – ESA symposium
Canberra, Australia, May 2018 – Boden conference
Davos Switzerland, June 2018 – Polar2018 symposium
Gland Switzerland June 2018 – IUCN workshop/meeting
Amman, Jordan, Sept 2018 – IUCN workshop/meeting
Dubai, UAE, Oct 2018 – Eye ON Earth symposium
Kuala Lumpur, Malaysia July 2019 – ICCB symposium
Maputo, Mozambique, Mar 2019 – IUCN workshop/meeting
Mombasa, Kenya Mar 2019 – IUCN workshop/meeting
New York, USA, June 2019 – UN SEEA-EEA forum of experts
Jurmala, Latvia, Sept 2019 – IUCN workshop/meeting
Helsinki, Finland, Oct 2019 – IUCN workshop/meeting
Sydney, Australia, Nov 2019 – Ocean Ecosystems Accounting symposium
Global (virtual) June 2020 – UN SEEA Experimental Ecosystem Accounting Expert Forum

Based on input from working group members and from the participants in interactive consultation meetings listed above, v1.0 of the Global Ecosystem Typology was completed in October 2019, with descriptions and indicative maps for 102 Ecosystem Functional Groups (EFGs) arranged within 25 functional biomes and five realms. In February 2020, v1.01 of the typology with one additional functional group, was published in a report made available through the IUCN Red List of ecosystems website (https://iucnrle.org/about-rle/ongoing-initiatives/global-ecosystem-typology/).

The second phase was an intensive peer review of the descriptive profiles and maps by leading ecosystem scientists whose expertise spanned all biomes in the typology. The peer review process was managed by Professor Emily Nicholson (Deakin University). A total of 60 specialists undertook 261 reviews of the 110 Ecosystem Functional Groups, a mean of 2.4 reviews per EFG. Their contributions represent the collective views of global research networks numbering hundreds of experts. A comprehensive revision of v1.01 of the typology and the descriptive profiles was undertaken in response to reviewer comments. The revisions included the addition of five Ecosystem Functional Groups to the typology to encompass variation that was not well unaccounted for in v1.01, major revisions to profiles of seven existing EFGs and at least one minor adjustment to the text, diagrams or maps for all remaining EFGs. A full synopsis of the anonymised reviewer comments, responses and revisions is given in Table S5.1.

Simultaneously with the peer review, we undertook a review of open source global map data sets available for ecosystems to identify those relevant to mapping the distribution of EFGs. This enabled us to replace first-generation coarse-scale indicative maps with second generation maps of greater spatial resolution that were matched to the concepts of respective EFGs (see Appendix S4 for details of source data for maps of respective EFGs). These revisions were incorporated into v2.1 of the typology.

The third phase of review involved testing the typology by developing cross-walks with established national classifications of ecological units. The majority of these classifications were for terrestrial environments based on features of surface vegetation, some were for freshwater environments and a few covered marine environments. The cross-walks were based on expert elicitation. Experts were asked to estimate membership values (0-1) for each unit of a national classification, representing their degree of confidence that belongs to each global functional group (e.g. membership values of '1' represent no reasonable doubt that national unit 'X" belongs to functional group 'Y'; '0' represents no reasonable doubt that national unit 'X" DOES NOT belong to functional group 'Y', and intermediate values indicate varying levels of uncertainty in membership). The cross-walk testing included a collaboration with the United Nations System for Environmental Ecosystem Accounts Experimental Ecosystem Accounting Revision (UN SEEA-EEA Revision) through its Virtual Expert Forum on SEEA Experimental Ecosystem Accounting in June 2020 (https://seea.un.org/content/seea-experimental-ecosystem-accounting-revision).

The detailed methods and results of this work will be published elsewhere as a basis for integrating established fine-scale ecosystem classifications into Level 6 of the global typology. However, information on uncertain relationships obtained from the cross-walks was used to resolve ambiguities in the descriptions of EFGs and develop a description for one additional group (T7.5). These revisions were incorporated into v2.1 of the typology.

The fourth phase was a further peer review to conform with IUCN's publication process. Two experts, one terrestrial specialist and one marine specialist, were engaged to review the entire report including descriptive profiles for functional biomes and EFGs within their respective areas of expertise. The revisions arising from these reviews were incorporated into v2.1 of the typology. Finally, all profiles were reviewed and edited for conceptual consistency by the lead author, and subject to a final production edit.

Overall, the process outlined above involved up to six iterations of review and revision for each descriptive profile, resulting in refinements of content, revision of some group definitions or recognition of additional groups. The extensive exposure to expert scrutiny in the third phase of review demonstrated the robustness of the typology, since most of the earlier structure and detail was re-affirmed. The review process also demonstrated a mechanism by which new knowledge can be incorporated through update, revision and release of future versions of the typology.

Table S5.1. Anonymised peer review comments, responses and revisions. Each row represents a different review. Multiple rows for the same Ecosystem Functional Group or biome represent different individual reviewers.

Ecosystem Functional Group (EFG)	General comment	Specific comments	Response type	Detailed response
Terrestrial				
Biome T1 Tropical-subtropical forests		P.1 All fine, except there are large ecotonal areas with drier biomes where fire becomes more important.	Minor edits to text	Added "Fires may occur in ecotonal areas between these forests and savannas"
Biome T1 Tropical-subtropical forests	I love the general approach, combining top-down and bottom-up approaches, but there are significant problems with T1.1 and T1.3. I have minor additional comments and suggestions for T1.2, T1.4, T7.4	1. T.1.1: This biome is first described as 'moderate to highly productive', but thereafter is all highly productive. I don't understand what is meant by it occurring 'primarily' at lower latitudes: it is ALL at lower latitudes.	Multiple edits to text	1. i) The biome is described as 'moderate to highly productive' because it includes four functional groups of forest that span this range. T1.1 is the most productive of these, and therefore is described as "highly productive". The other three groups within T1 include some forests with moderate levels of productivity, hence the description should be OK<br>ii) The phrase "primarily at lower latitudes" is the T1 biome text. Agree that the qualification is unwarranted - deleted 'primarily' from 'lower latitudes'
T1.1Tropical-subtropical lowland rainforests	I examined all the tropical systems, especially those I personal experience with. I am especially knowledgeable about Latin America, and in particular, systems occurring in Peru. In general, sufficient variation is covered, and I found the product useful.	P. 2 Sometimes humid closed tropical lowland forests have extensive understory dominance by bamboos.	Minor edits to text	Added "bamboos (sometimes abundant)" to list of plant life forms

Ecosystem Functional Group (EFG)	General comment	Specific comments	Response type	Detailed response
T1.1Tropical-subtropical lowland rainforests	I love the general approach, combining top-down and bottom-up approaches, but there are significant problems with T1.1 and T1.3. I have minor additional comments and suggestions for T1.2, T1.4, T7.4	1. T1.1 and T1.3: Both are described as 'Tropical-subtropical' and the maps show large areas in subtropical China and eastern South America. However, both biomes are described as frost free, with T1.1 having 'minima rarely <10 oC'. And T1.3 '>0oC in all months of the year'. This is not correct. All, or almost all, evergreen forests outside the solar tropics are subject to at least occasional frosts, and at the poleward and altitudinal boundaries, these forests may experience winter temperatures > 10 degrees below zero. Given the nomenclature and the subtropical extent of these forests it is important that they are not excluded or treated as anomalous. If you are not going to have a separate subtropical forest category, you have to make clear that subzero temperatures are not anomalous. Note also that the map considerably underestimates the extent of these subtropical forests. See the map in Corlett, R.T. and Hughes, A.C. 2015. Subtropical Forests, pp. 46-55 in Handbook of Forest Ecology, Routledge. This also summarizes other aspects of their biology and probably should be cited. Note that 'no frost' is also mentioned on page 22.<br>2. T.1.1: This biome is first described as 'moderate to highly productive', but thereafter is all highly productive. I don't understand what is meant by it occurring 'primarily' at lower latitudes: it is ALL at lower latitudes. It is NOT true that it	Multiple edits to text, diagram and map	General: The forests referred to as "subtropical" by the reviewer (and the majority of latitudinally delimited forests described in Corlett & Hughes 2015) are indeed classified in a separate group: T2.4 as "Warm temperate rainforests". Thus reviewer comments regarding temperature range, frosts and distribution relate mainly to T2.4 (not so much T1.1). These evergreen forests lack the full structural complexity of T1.1 and extend into the subtropical latitudes, being extensive in subtropical southeast China and also occur all the other areas mentioned in Corlett & Hughes (2015) as well as some temperate areas (southern Japan, NZ, southeast Aust, eastern Cape Sth Africa) - see Grubb et al. 2013 Biol Rev. The compositional descriptions in Corlett & Hughes (2015) also fit evergreen forests in T2.4 very well (i.e. Fagacaea, Lauraceae, Cunoniaceae, Podocarpaceae, Myrtaceae, etc).<br>1. We amended the text to clarify distinctions and temperature relationships between T1.1 and T2.4 as follows:<br>i) Added new 2nd sentence: "At subtropical latitudes they transition to warm temperate forests (T2.4)."<br>ii) In Drivers section, qualified (mean winter minima rarely <10°C)" as follows: "(mean winter minima rarely <10°C except in subtropical transitional zones).<br>iii) Modified map to re-interpret most forests in that region (including Araucarian) as T2.4, rather than T1.1<br>2. i) The biome is described as 'moderate to highly productive' because it includes four functional groups f forest that span this range. T1.1 is the most productive of these, and therefore is described as "highly productive". The other three groups within T1 include some forests with moderate levels of productivity, hence the description should be OK<br>ii) The phrase "primarily at lower latitudes" is the T1 biome text. Agree that the qualification is unwarranted - deleted 'primarily' from 'lower latitudes'<br>iii) rephrased description of phenology as follows to more accurately describe the variation within the group: "Growth and

Ecosystem Functional Group (EFG)	General comment	Specific comments	Response type	Detailed response
		mostly has ‘unseasonal or weakly seasonal’ phenology. Most of the area mapped, including all the subtropical area and a lot of the tropical area, has an annual phenology. What are ‘territorial vertebrates’ and why are they separated out? I think the importance of tropical storms is exaggerated. They are important in broad coastal strips in parts of Asia, Madagascar, the Caribbean, and Australia, but 90% of this biome is not subject to canopy-destroying storms on decadal time scales.		reproductive phenology may be seasonal or unseasonal, and reproductive masting..."<br>iv) Deleted "territorial" descriptor of vertebrates’<br>v) Rephrased text on tropical storms to emphasise their limited geographic context as follows: "In some coastal regions outside equatorial latitudes (mostly >10° and excluding extensive forests in continental America and Africa), decadal regimes of tropical storms drive cycles of canopy destruction and renewal." Added "(some coastal regions)" qualifier to Storms box in Diagram
T1.2 Tropical-subtropical dry forests and thickets	I examined all the tropical systems, especially those I personal experience with. I am especially knowledgeable about Latin America, and in particular, systems occurring in Peru. In general, sufficient variation is covered, and I found the product useful.	P.3 Here get ecotonal areas with grasses and mixed woodland/grassland types.	Minor edits to text	Added Grasses are rare or absent, "except on savanna ecotones," and similar phrase regarding fire
T1.2 Tropical-subtropical dry forests and thickets	I love the general approach, combining top-down and bottom-up approaches, but there are significant problems with T1.1 and T1.3. I have minor additional comments and suggestions for T1.2, T1.4, T7.4	‘scrubs’ is horrible and has strong negative connotations – ‘wasteland’ – in most variations of the English language. You need a neutral term, like shrubland or thickets. I did not understand what ‘low exclusivity’ meant.	Amendment to name	Replaced "scrubs" with "thickets"

Ecosystem Functional Group (EFG)	General comment	Specific comments	Response type	Detailed response
T1.3 Tropical-subtropical montane rainforests	I love the general approach, combining top-down and bottom-up approaches, but there are significant problems with T1.1 and T1.3. I have minor additional comments and suggestions for T1.2, T1.4, T7.4	1. T1.1 and T1.3: Both are described as ‘Tropical-subtropical’ and the maps show large areas in subtropical China and eastern South America. However, both biomes are described as frost free, with T1.1 having ‘minima rarely <10 oC’. And T1.3 ‘>0oC in all months of the year’. This is not correct. All, or almost all, evergreen forests outside the solar tropics are subject to at least occasional frosts, and at the poleward and altitudinal boundaries, these forests may experience winter temperatures > 10 degrees below zero. Given the nomenclature and the subtropical extent of these forests it is important that they are not excluded or treated as anomalous. If you are not going to have a separate subtropical forest category, you have to make clear that subzero temperatures are not anomalous. Note also that the map considerably underestimates the extent of these subtropical forests. See the map in Corlett, R.T. and Hughes, A.C. 2015. Subtropical Forests, pp. 46-55 in Handbook of Forest Ecology, Routledge. This also summarizes other aspects of their biology and probably should be cited. Note that ‘no frost’ is also mentioned on page 22.<br>2. T.1.1: This biome is first described as ‘moderate to highly productive’, but thereafter is all highly productive. I don’t understand what is meant by it occurring ‘primarily’ at lower latitudes: it is ALL at lower latitudes. It is NOT true that it	Multiple edits to text	General: The forests referred to as "subtropical" by the reviewer (and the majority of latitudinally delimited forests described in Corlett & Hughes 2015) are classified in a separate group T2.4 as "Warm temperate rainforests". Thus reviewer comments regarding temperature range, frosts and distribution relate mainly to T2.4 (not so much T1.1). These evergreen forests lack the full structural complexity of T1.1 and extend into the subtropical latitudes, being extensive in subtropical southeast China and also occur all the other areas mentioned in Corlett & Hughes (2015) as well as some temperate areas (southern Japan, NZ, southeast Aust, eastern Cape Sth Africa) - see Grubb et al. 2013 Biol Rev. The compositional descriptions in Corlett & Hughes (2015) also fit evergreen forests in T2.4 very well (i.e. Fagacaea, Lauraceae, Cunoniaceae, Podocarpaceae, Myrtaceae, etc).<br>1. We amended the text to clarify distinctions and temperature relationships between T1.1 and T2.4 as follows:<br>i) Added new 2nd sentence: "They transition to lowland rainforests (T1.1) with decreasing altitude and to warm temperate forests (T2.4) at subtropical latitudes."<br>ii) In Drivers section, rephrased temperature text as follows: "Temperatures are mild-cool with occasional frost. Seasonal variability is low-moderate and but diurnal variability is moderate-high. Winter monthly mean minima may be around 0°C in some areas."<br>2. i) The biome is described as ‘moderate to highly productive’ because it includes four functional groups of forest that span this range. T1.3 is is described as "moderate productivity fuelled by autochthonous energy, limited by cool temperatures, possibly by high exposure to UV-B radiation, and sometimes by shallow soil and/or wind exposure". The other three groups within T1 include some forests with moderate levels of productivity and one (T1.1) with high productivity, hence the description should be OK<br>ii) The phrase "primarily at lower latitudes" is the T1 biome text. Agree that the qualification is unwarranted - deleted ‘primarily’ from 'lower latitudes'

Ecosystem Functional Group (EFG)	General comment	Specific comments	Response type	Detailed response
		mostly has 'unseasonal or weakly seasonal' phenology. Most of the area mapped, including all the subtropical area and a lot of the tropical area, has an annual phenology. What are 'territorial vertebrates' and why are they separatedout? I think the importanceof tropical storms is exaggerated. They are important in broad coastal stripsin parts of Asia, Madagascar, the Caribbean, and Australia, but 90% of this biome is not subject to canopy-destroying storms on decadal time scales.		iii) Rephrased description of phenology as follows to more accurately describe the variation within the group: "Growth and reproductive phenology is usually seasonal."<br>iv) Deleted "territorial" descriptor of vertebrates'<br>v) Storms arise from regional weather systems (e.g. cyclones) only in some regions or local turbulence and orographic effects of mountains. Text refers to extreme rainfall events and high winds, suitably qualified "in some regions". Rephrased text to emphasise qualification as follows: "...and, in some regions, are exposed to local or regional storms." Added "(some coastal regions)" qualifier to Storms box in Diagram
T1.3 Tropical-subtropical montane rainforests		P.4 Some places in cloud forests are dominated by understory bamboos. Since bamboos are grasses, technically not true that grasses are absent. Rest is fine.	Minor edits to text	Added grasses are rare or absent, "except for bamboos in some areas"
T1.4 Tropical heath forests	I examined all the tropical systems, especially those I personal experience with. I am especially knowledgeable about Latin America, and in particular, systems occurring in Peru. In general, sufficient variation is covered, and I found the product useful.	P.5 Note that the west Amazonia includes spots into Peru and even rarely Ecuador, so map leaves out western margin of Amazon.	No change	Noted, the mapped distribution does extend to east Peru. More detailed maps will be made available as data

Ecosystem Functional Group (EFG)	General comment	Specific comments	Response type	Detailed response
Biome T2 Temperate-boreal forests & woodlands		Boreal – derived from the Greek word for north wind – is primarily a term used to describe high latitude northern hemisphere ecosystems. While I accept that boreal now has a broader connotation and is used to describe climates similar to those of the far north, it still retains its geographic and biogeographic meaning. If an animal is described as boreal, we assume its natural range is in the far north. As it is ambiguous, I really do not think it has a place in a typological system designed to be global in reach unless qualified in some way. For instance, as far as I know it is not used at all in the Southern Hemisphere.<br>I make two suggestions for how to remedy this. Biome T2 could be referred to as Temperate-austroboreal forests and woodlands, or boreal could be replaced by 'cool'. I would prefer the latter solution as it unambiguous and forms a natural series with 'polar-alpine' which occupies the 'cold' slot. This formulation is used in T3.3 Cool temperate heathlands for example. 'Austro' on the other hand is not used much in the Southern Hemisphere as an ecological descriptor in the same way as boreal, but more as a geographic term.	No change	We thank the reviewer for thoughtful suggestions towards more systematic nomenclature. For the global ecosystem typology, we opted for a vernacular approach to terminology to enable familiarity to a broad range of users. 'Temperate forests is widely understood to include all forests outside the tropics in the southern hemisphere. The same is not true for the northern hemisphere, where forests of the far north are widely understood as 'boreal', but they are not recognised as 'temperate'. Therefore we believe both 'temperate' and 'boreal' terms are essential to convey the full text on this biome.
T2.3 Oceanic temperate rainforests		1. I am pleased to see this concept included in the global system because, although these temperate systems growing	Minor edits to text	1. We agree that oceanic moderation of cool temperate climates has significant implications for forest for and function ni small but widely dispersed locations around the world.

Ecosystem Functional Group (EFG)	General comment	Specific comments	Response type	Detailed response
		under coolish to moderately warm climates with muted seasonal variation cover only a small area of the globe, they are important where they occur. In our formulation of the concept of an ‘oceanic temperate forest’ climate (McGlone et al. 2016, 2017), we included coastal northwest Europe, much of south eastern Australia, Southern Chile, southeastern Brazil and scattered tropo-montane forests throughout.<br>2. We discussed how, although the climate parameters fit the concept, historical factors meant that the plant physiognomy of these areas often differed greatly. For the purposes of the current global typology, I accept that this would lead to confusion, and it is probably for the best that the OTF concept is retained only for largely evergreen microphyll dominant forests. That would exclude the deciduous forests of northwest Europe as they lack a strong representation of evergreen trees, be those conifer or angiosperm. The cool montane tropical forests pose a problem as they lack any winter season and one could expect them to function somewhat differently to OTF even though they in some respects fit the climatic criteria otherwise, and have a striking physiognomic resemblance to the lowland forests of the wetter areas of New Zealand.		2. The reviewer's commentary and our interpretation of ecosystem functional groups are in broad agreement. The contrasting evolutionary histories reflect difference in species pools that form a template for ecosystem assembly, resulting in functional (and physiognomically) contrasting forests in post glacial Europe and North America (functional group T2.2) and the montane tropics (functional group T1.3), despite the resemblance of some aspects of their contemporary climates. We added the following text to distinguish T2.3 and T2.4 as follows: "The smaller range of leaf sizes and SLA, varied phenology, frost tolerance, broader edaphic association, and wetter, cooler climate distinguish these forests from warm temperate forests (T2.4)." We also amended the name to Oceanic 'cool' temperate forests. See T2.4 responses for further comment.

Ecosystem Functional Group (EFG)	General comment	Specific comments	Response type	Detailed response
T2.3 Oceanic temperate rainforests		1. Why not also western Wales, Scotland and Norway?<br>2. Yes I think I would do this [i.e. include western Europe, Japan and Atlantic Canada]	No change	1. Although some climatic features of Wales, Scotland and Norway are within the range of conditions for this functional group, the ecological traits do not match well, possibly dues to legacies of late Pleistocene glaciation. The broad-leaf deciduous forests of Wales, Scotland and Norway are a strong match with T2,2, and the coniferous forests a strong match for T2.1. There is insufficient evidence of 'rainforest' features in this region consistent with T2.3, and thus the separation of these forests from others in western Europe cannot be strongly justified<br>2. This comment relates to the same issue as above, but also for Japan and Atlantic Canada (climate within scope but inconsistencies in ecological traits). Text acknowledges that some authors suggest similarities on the basis of climate
T2.4 Warm temperate laurophyll forests		1. There has been a debate in New Zealand about the application of this term. I refer you to a follow-up paper (McGlone et al. 2017) I and my co-authors wrote in response to criticism of McGlone et al. (2016) by Peter Grubb and co-authors. In essence, the point of difference between the Grubb approach and ours, was that we used climatic criteria to draw boundaries, but they (at least they said they didn’t) did not. The key issue was the classification of the forests in the north of New Zealand (north of 38°S with some coastal extension further south). They argued that these forests fit on physiognomic grounds nicely into their broad concept of ‘warm temperate rainforest’. However, we countered that there are very few physiognomic differences between the northern New Zealand forests and those further to the south and it was impossible	Minor edits to text and map	1. This debate has been formative on our thinking in developing the temperate forest component of the global typology.<br>2. We agree that Warm temperate forests proposed by Grubb et al. involve a number of exceptions to their definitional criteria. The concept of T2.4 aligns broadly with the first of the alternative WTRF concept proposed by McGlone et al. (2017): an "operational physiognomic forest type—for instance ‘evergreen non-sclerophyll microphyll forest’—and accept that it will extend into climate zones or vegetation types that do not fit [Grubb's] WTRF concept”. See point 3 below.<br>3. The concept of T2.3 aligns reasonably well with the suggested ‘Oceanic cool temperate forest’. A strictly ‘Oceanic warm temperate forest’ is more problematic because of the difficulty in distinguishing functional traits and processes in more continental warm temperate forests in the Himalayan slopes, southern China, SE USA, from more oceanic areas in southern Japan, east China, Pacific NW America, SE Australia and lowland parts of NZ as well as those in Mediterranean-type climates in central Chile and South Africa. We therefore adopted a broad circumscription for T2.4 to encompass forests in all these areas, and thank the reviewer for pointing out that Atlantic Araucarian forests of

Ecosystem Functional Group (EFG)	General comment	Specific comments	Response type	Detailed response
		to draw a line that was not arbitrary. Furthermore, we demonstrated that their suite of physiognomic traits used to define ‘warm temperate forest’ were very wide and haphazardly followed, that cryptic criteria were used, and we also commented that the use of climatic terms to describe this biome undercut their claim.<br>2. Our conclusion regarding their concept of Warm Temperate Rainforest was:“In our opinion, the current WTRF definition cannot be operationalised as it is subjective and riddled with exceptions. We see only two ways forward for the WTRF concept. Either create a more operational physiognomic forest type—for instance ‘evergreen non-sclerophyll microphyll forest’—and accept that it will extend into climate zones or vegetation types that do not fit the current WTRF concept; or introduce additional bioclimatic criteria—as per Kira (1991)—and accept that there will be forests within its ambit that are physiognomically or structurally distinct.”<br>3. We suggested that a better route would be to use ‘Oceanic warm temperate forest’ and ‘Oceanic cool temperate forest’. We also commented that the forests of southeastern China and Japan did not fit this concept largely because their continental setting led to greater seasonal extremes. On the other hand, we noted that the Araucarian forests of southeastern		southeastern Brazil belong to this group and have adjusted the map accordingly. We added the following text to highlight distinctions between T2.3 and T2.4 as follows: "Their foliage is often leathery with intermediate SLA values, notophyll-microphyll leaf sizes, and is overwhelmingly evergreen. Deciduous species are sometimes scattered within the forest canopies. These features, with drier, warmer climatic and edaphic associations distinguish them from oceanic cool temperate forests (T2.3).<br>4. We agree that T2.3 is present in north (and south) NZ and Chile. The current map reflects this. We also agree that 'warm temperate' forests in the north of NZ have very similar analogues on the South Island (e.g. on the Catlins coast lowlands). We have made small adjustment to make south island occurrence clearer. Unfortunately the coarse resolution of out first-generation maps is insufficient to show the smaller occurrences on the South Island, but we aim to improve on this with second-generation maps.<br>5. The minor adjustments described above bring the circumscription closer to that suggested by the reviewer, but there are still some differences, which enable functionally contrasting temperate forests to be recognised within regions such as NZ, Chile, Australia and the southern Himalayan fall. The focus of the typology on functional outcomes rather than primarily on climatic causes of functional difference underpins this rationale.

Ecosystem Functional Group (EFG)	General comment	Specific comments	Response type	Detailed response
		Brazil did match OTF. They bear a strong resemblance to the forests of northern New Zealand, sharing tree genera in common. I have since visited the Canary Islands and their montane evergreen forests bear a striking resemblance to many associations in New Zealand.<br>4. Even if the Grubb et al. concept of Warm Temperate Forest is accepted, the map in the IUCN topology has some anomalies. Northern New Zealand is depicted as being dominated by this biome while very similar forests in southern Chile at the same latitude and sharing almost the same climatic space are largely assigned to T2.3 Oceanic Temperate Rainforest.<br>5. I understand that a global system cannot be distorted by too much attention to local problems, and the following are suggestions only. However, all that is needed to adjust for the anomalies I have pointed out is for: the Warm Temperate Forest category to be restricted to largely continental regions with pronounced seasonality (mainly Japan, southeastern China and parts of the southeastern United States) and the Oceanic Temperate Forest biome extended to include the warmer (but still highly oceanic) portion of its range. That is, northern New Zealand, the Valdivian rainforest, the Araucarian forests of southeastern Brazil and pockets of non-sclerophyll forest in Tasmania and southeastern Australia and perhaps		

Ecosystem Functional Group (EFG)	General comment	Specific comments	Response type	Detailed response
		patches of forest elsewhere as in the Canaries, coastal South Africa etc. As commented in the previous section, despite marked similarities in floristics, physiognomy and climate envelope to the OTF, the tropical montane associations are a better fit for T1.3 Tropical-subtropical montane rainforests.<br>McGlone MS, Buitenwerf R, Richardson SJ 2016. The formation of the oceanic temperate forests of New Zealand. New Zealand Journal of Botany 54(2): 128-155.<br>McGlone MS, Buitenwerf R, Richardson SJ 2017. Oceanic temperate forest versus warm temperate rainforest: a reply to Grubb et al. (2017). New Zealand Journal of Botany 55(3): 378-385.		
T2.4 Warm temperate laurophyll forests		Why not call them: Warm temperate evergreen laurophyll forests. I'm afraid the those indicated in W North America do not fit in this unit but in the Mediterranean unit T2.6	Amendment to name	A universally applicable name for this group is problematic. Our approach is to use vaernacular names that highlight distinguishing features, while broadly familiar to the diverse users of the typology. It is true that these forests are evergreen and that most trees have laurophylli leaves. However many other forest functional groups are evergreen, and many users (including non-specialists in vegetation science) would not be familiar with "laurophyll" so we decided against those terms. However, the group includes some ecosystems not traditionally known as 'rainforests' (including those of southwest USA with evergreen Magnolia, Quercus, etc). Balancing these trade-offs, and other options for names (e.g. moist forests, which may include various pyric forests, e.g. T2.5) we amended the name to "Warm temperature laurophyll forests." The southern USA also includes flammable forests referrable to T2.6, mainly in California.

Ecosystem Functional Group (EFG)	General comment	Specific comments	Response type	Detailed response
T2.4 Warm temperate laurophyll forests	I love the general approach, combining top-down and bottom-up approaches, but there are significant problems with T1.1 and T1.3. I have minor additional comments and suggestions for T1.2, T1.4, T7.4	T1.1 and T1.3: Both are described as ‘Tropical-subtropical’ and the maps show large areas in subtropical China and eastern South America. However, both biomes are described as frost free, with T1.1 having ‘minima rarely <10°C’. And T1.3 ‘>0°C in all months of the year’. This is not correct. All, or almost all, evergreen forests outside the solar tropics are subject to at least occasional frosts, and at the poleward and altitudinal boundaries, these forests may experience winter temperatures > 10 degrees below zero. Given the nomenclature and the subtropical extent of these forests it is important that they are not excluded or treated as anomalous. If you are not going to have a separate subtropical forest category, you have to make clear that subzero temperatures are not anomalous. Note also that the map considerably underestimates the extent of these subtropical forests. See the map in Corlett, R.T. and Hughes, A.C. 2015. Subtropical Forests, pp. 46-55 in Handbook of Forest Ecology, Routledge. This also summarizes other aspects of their biology and probably should be cited. Note that ‘no frost’ is also mentioned on page 22.	Multiple edits to text, diagram and map	The forests referred to as "subtropical" by the reviewer (and the majority of latitudinally delimited forests described in Corlett & Hughes 2015) are indeed classified in a separate group: T2.4 as "Warm temperate rainforests". Thus reviewer comments regarding temperature range, frosts and distribution relate mainly to T2.4 (not so much T1.1). These evergreen forests lack the full structural complexity of T1.1 and extend into the subtropical latitudes, being extensive in subtropical southeast China and also occur all the other areas mentioned in Corlett & Hughes (2015) as well as some temperate areas (southern Japan, NZ, southeast Aust, eastern Cape Sth Africa) - see Grubb et al. 2013 Biol Rev. The compositional descriptions in Corlett & Hughes (2015) also fit evergreen forests in T2.4 very well (i.e. Fagacaeae, Lauraceae, Cunoniaceae, Podocarpaceae, Myrtaceae, etc).<br>1. We amended the text to clarify distinctions and temperature relationships between T1.1 and T2.4 as follows:<br>i) Added new 2nd sentence: "At subtropical latitudes they transition to warm temperate forests (T2.4)."<br>ii) In Drivers section, qualified (mean winter minima rarely <10°C)" as follows: "(mean winter minima rarely <10°C except in subtropical transitional zones).<br>iii) Modified map to re-interpret most forests in that region (including Araucarian) as T2.4, rather than T1.1<br>2. i) The biome is described as ‘moderate to highly productive’ because it includes four functional groups f forest that span this range. T1.1 is the most productive of these, and therefore is described as "highly productive". The other three groups within T1 include some forests with moderate levels of productivity, hence the description should be OK<br>ii) The phrase "primarily at lower latitudes" is the T1 biome text. Agree that the qualification is unwarranted - deleted ‘primarily’ from 'lower latitudes'<br>iii) rephrased description of phenology as follows to more accurately describe the variation within the group: "Growth and reproductive phenology may be seasonal or unseasonal, and

Ecosystem Functional Group (EFG)	General comment	Specific comments	Response type	Detailed response
				reproductive masting..."<br>iv) Deleted "territorial" descriptor of vertebrates'<br>v) Rephrased text on tropical storms to emphasise their limited geographic context as follows: "In some coastal regions outside equatorial latitudes (mostly >10° and excluding extensive forests in continental America and Africa), decadal regimes of tropical storms drive cycles of canopy destruction and renewal." Added "(some coastal regions)" qualifier to Storms box in Diagram
T2.5 Temperate pyric humid forests		it seems to be a humid variant of the following one. I think it's too much a biome for the tall eucalypts of Australia	No change	1. This debate has been formative on our thinking in developing the temperate forest component of the global typology.<br>2. We agree that Warm temperate forests proposed by Grubb et al. involve a number of exceptions to their definitional criteria. The concept of T2.4 aligns broadly with the first of the alternative WTRF concept proposed by McGlone et al. (2017): an "operational physiognomic forest type—for instance 'evergreen non-sclerophyll microphyll forest'—and accept that it will extend into climate zones or vegetation types that do not fit [Grubb's] WTRF concept". See point 3 below.<br>3. The concept of T2.3 aligns reasonably well with the suggested 'Oceanic cool temperate forest'. A strictly 'Oceanic warm temperate forest' is more problematic because of the difficulty in distinguishing functional traits and processes in more continental warm temperate forests in the Himalayan slopes, southern China, SE USA, from more oceanic areas in southern Japan, east China, Pacific NW America, SE Australia and lowland parts of NZ as well as those in Mediterranean-type climates in central Chile and South Africa. We therefore adopted a broad circumscription for T2.4 to encompass forests in all these areas, and thank the reviewer for pointing out that Atlantic Araucarian forests of southeastern Brazil belong to this group and have adjusted the map accordingly. We added the following text to highlight distinctions between T2.3 and T2.4 as follows: "Their foliage is often leathery with intermediate SLA values, notophyll-microphyll leaf sizes, and is overwhelmingly evergreen. Deciduous species are sometimes scattered within the forest

Ecosystem Functional Group (EFG)	General comment	Specific comments	Response type	Detailed response
				canopies. These features, with drier, warmer climatic and edaphic associations distinguish them from oceanic cool temperate forests (T2.3).<br>4. We agree that T2.3 is present in north (and south) NZ and Chile. The current map reflects this. We also agree that 'warm temperate' forests in the north of NZ have very similar analogues on the South Island (e.g. on the Catlins coast lowlands). We have made small adjustment to make south island occurrence clearer. Unfortunately the coarse resolution of out first-generation maps is insufficient to show the smaller occurrences on the South Island, but we aim to improve on this with second-generation maps.<br>5. The minor adjustments described above bring the circumscription closer to that suggested by the reviewer, but there are still some differences, which enable functionally contrasting temperate forests to be recognised within regions such as NZ, Chile, Australia and the southern Himalayan fall. The focus of the typology on functional outcomes rather than primarily on climatic causes of functional difference underpins this rationale.
T2.6 Temperate pyric sclerophyll forests and woodlands		1. These forests and woodlands are not pyric (or pyrophilous) in all the cases. In the Mediterranean region, fire promotes scrub, or grassland, by burning the forest. The forest is not related with that disturbance. Only some versions of pine woodlands can be related to fire. Another point is why not to use the expanded term "Mediterranean" for this type? I miss Central Chile for this type.<br>2. sclerophyllous and microphyllous	No change	1. This group includes some but not all forests in Mediterranean climate regions, and substantial areas of temperate forest in non-Mediterranean climates. Non-flammable forests, such as those in Chile, are not part of T2.6. Pines, some evergreen oaks and eucalypts all have fire response traits, one of the characteristics that unts this group of systems.<br>2. Microphyll leaf size is mentioned in the third sentence: "Trees have microphyll foliage ..."

Ecosystem Functional Group (EFG)	General comment	Specific comments	Response type	Detailed response
T3.1 Seasonally dry tropical shrublands		T3.1--not sure that I recognise this assemblage! but I suppose you've somehow got to lump these vaguely similar tropical types of oligotrophic shrubby types together--for example, areas of campos rupestre I have seen in Brazil range from grasslands (with herbs and some shrubs) to rocky pavements with shrubby/herbaceous veg. Realistically they would seem connected by a Gondwanic origin but have drifted northwards...be that as it may!	Minor edits to text and refs	Added shrubby grasslands to description of structure and noted C4 grasses may be dominant. Added Fernandes (2016) to refs
T3.1 Seasonally dry tropical shrublands	I examined all the tropical systems, especially those I personal experience with. I am especially knowledgeable about Latin America, and in particular, systems occurring in Peru. In general, sufficient variation is covered, and I found the product useful.	P.14 Get some similar (rare) systems in Peru, up on tabletop mountains with sandstone underneath, also with some shared similarities with the distant Tepuis of Venezuela.	Minor edits to text	Peruvian tabletops added to Distribution description
T3.1 Seasonally dry tropical shrublands		Itigi type thickets ecoregion in Africa might fit in here?	No change	Itigi thickets belong to the T1.2 Tropical-subtropical dry forests and thickets functional group, with characteristic closed structure, dry-season deciduous dominants and limited grass cover. They lack the sclerophyll character of T3.1, which also has a stronger influence of nutiritional poverty and fire than T1.2

Ecosystem Functional Group (EFG)	General comment	Specific comments	Response type	Detailed response
T3.2 Seasonally dry temperate heaths and shrublands		1. Winters - in higher elevations not so mild<br>2. Soil textures - not always sandy, often clayey<br>3. Soils are typically acid - not tipically, often are base-rich<br>4. Soils derived from siliceous substates - often derived from limestone, marl and dolomite<br>5. Diagram Cimate box - Dry summers, they can be hot or not so much<br>6. Diagram Nutrients box - "poverty" nor always, there are many areas on limestone, marl, dolomite, etc. which are not base reaction nutrient poor.<br>7. Diagram Substrates box - nutrients are not always acidic, often are base rich. Soils are shallow and often degraded by human action (fire, overgrazing, erosion)<br>8. Diagram Disturbance box - fire and grazing	Multiple edits to text and diagram	1. Amended text as follows: Winters are mild, "or cool at high elevation"s"<br>2. Amended text as follows: Sandy soil textures "or reverse-texture effects of clay-loams" exacerbate an overall water deficit<br>3. Added example to emphasise exception as follows: though this varies between regions, "e.g, base-rich limestones, marle and dolomites in southern Europe"<br>4. See response to point 3<br>5. Summer water deficit may be produced by low summer rainfall, high summer temperatures (thus high evapotranspiration) or a combination of both. Replace "- hot summers" with "- summers hot &/or low rainfall"<br>6. Nutrients may be at low levels overall or imbalanced (e.g. with high Ca). Replaced "Nutrient poverty" with "Nutrient poverty or imbalance"<br>7. Added "- rarely base-rich (e.g. limestone)" to reflect exceptions common in Europe, but not other major regions<br>8. Grazing regimes have a minor role compared to fire (except domestic herbivores which are beyond scope of profile), although native browsers may have large local effect even when present in low densities. Hence, no change to diagram, but but added the following to text: "Native browsers may have local effects on vegetation."

Ecosystem Functional Group (EFG)	General comment	Specific comments	Response type	Detailed response
T3.3 Cool temperate heathlands		1. Not many ferns<br>2. yes, it is evrgreen and ericoid but often there are also evergreen genistoid shrubs, ofthe spiniy, of the genera Genista and Ulex<br>3. seasonally low temperature, light is not a limiting factor in those latitudes<br>4. High water table - only in depressions and sites where water table is high<br>5. forest and not forests, grasslands, etc.<br>6. this cyclic dynamic is mostly human induced by management regime<br>7. Diagram Energy box - solar flux limitation is nort really effective, but winter temperature limitation	Minor edits to text	1.Deleted "ferns" from text<br>2. Added the following phrase: "...or reduced to spiny stems"<br>3. Rephrased to clarify context of light limitation as follows: "Seasonally low light may limit productivity at the highest latitudes."<br>4. Rephrased to more accurately describe conditions: "Cool temperatures and low soil oxygen due to periodically wet subsoil limit decomposition..."<br>5. Rephrased as follows: "...forest and anthropogenic ecosystems..." to encompassed the most commonly co-occurring systems<br>6. The underlying dynamics is autogenic, though sometimes expressed in response to human-induced disturbance and management. Added "autogenic" cyclic dynamics to clarify and Watt (1947) to references<br>7. Diagram Energy box - qualified text as follows: "Solar flux (sometimes limiting). Cool winters already mentioned in Ambient environment box
T3.3 Cool temperate heathlands		Seems a bit overboard in the UK to me	Amendments to map	Agree, the actual extent of heathlands is resticted to parts of the west coast. First generation maps for review were constrained by ecoregion resolution. Second generation maps are higher resolution
T3.4 Rocky pavements, screes and lava flows	I examined all the tropical systems, especially those I personal experience with. I am especially knowledgeable about Latin America, and in particular, systems occurring in Peru. In general, sufficient variation is covered, and I found the product useful.	P.17 These shrub vegetations are common across Peru highlands, not just in the ones that have recent vulcanism. So, map perhaps understates area covered.	No change	The current map includes some non-volcanic highland areas in Peru. Unlikely to be much more widespread than shown

Ecosystem Functional Group (EFG)	General comment	Specific comments	Response type	Detailed response
T3.4 Rocky pavements, screes and lava flows		1. Sentence 1. This functional group covers a wide climatic range, so it is difficult to generalize about ecological traits. My overall feeling is that the text is spot-on for arid habitats, but perhaps neglects more humid settings (I work mainly in Iceland, so I am probably biased!) Specifically, I would say that the importance of bryophytes - a very important constituent of the vegetation in humid settings - is underplayed. Therefore, in the first sentence, it might be worth saying "Cryptogam (lichens and bryophytes) and shrub-dominated vegetation..."<br>2. Sentence 2. I would regard slope stability as an important limitation on ecosystem development in these settings.<br>3. Sentence 3. Ground-nesting birds, along with reptiles, might be included as a vertebrate group found in these settings.<br>4. Sentence 4. In my opinion, lichens and bryophytes also play a critical role in proto-soil formation, especially in terms of C contributions.<br>5. Sentence 9: "Species composition and vegetation structure are unstable..." I don't quite understand this statement. I would say that the stringent environmental filters that apply in these habitats select for a very limited (and rather tough) flora that is not prone to abrupt change (unless exposed to extreme allogenic factors).<br>6. Schematic: These habitats occur in cold as well as hot locations. Therefore, under	Multiple edits to text, diagram and map	1. Rephrased sentence 1 as follows: "Vegetation dominated by crytogams (lichens, bryophytes), of the with scattered shrubs- with very low LAI develops on skeletal rocky substrates."<br>2. Added phrase to Sentence 2 to clarify role of disturbance on slope stability as an important limitation on ecosystem development: ...and periodic disturbance "through mass movement"<br>3. Reworded last phrase of Sentence 3 as follows: "Reptiles and ground-nesting birds are among the few resident vertebrates."<br>4. Add phrase to Sentence 4to note important role of lichens and bryophytes in proto-soil formation: "..., and proto-soil development, especially through carbon accumulation"<br>5. Rephrased Sentence 9 to clarify influence of recurring disturbance t surface as follows: "Species composition and vegetation structure are dynamic in response to surface instability and ..."<br>6. Schematic: Changed 'high temperatures' to "High temperature variation" and labelled arrow to Water scarcity box as follows" "Periodic desiccating conditions". To eliminate duplication, deleted "Simple trophic network" from Traits circle<br>7. Distribution: Added Steep slopes of Himalayas, Baluchistan & south Iranian mountains, Atlas and central Papuan highlands

Ecosystem Functional Group (EFG)	General comment	Specific comments	Response type	Detailed response
		'Ambient temperatures' I would say point to extremes of temperature (both hot and cold), rather than just 'high temperatures'. Under 'Ecological traits', there are two statements regarding Trophic structure: could these be compressed into one?<br>7. Distribution: I would note that scree surfaces are ubiquitous in areas of high relief (e.g., the Himalaya), but I guess that these are dealt with in another section? It might be worth removing the heading 'screes' from the T3.4 heading and transferring it to the montane sections.		
Biome T4 Savannas and grasslands		T4 savannas and grasslands--your introductory first line mentions a continuous grass layer--that simply doesn't apply generically and you would be better saying 'more-or-less continuous'	Minor edits to text	Added qualifier 'mostly continuous
Biome T4 Savannas and grasslands		What is the difference between grassland and savanna? Are there non-forested savannas? What happens if human action removes the trees?	No change	We use a vernacular approach to nomenclature using terms that a broad readership can associate with particular ecosystems. Neither savannas nor grasslands on their own encompass the full range ecosystems in this group (many people associate savannas with the tropics and subtropics, while grasslands may exclude wooded savannas with continuous grassy understories. Hence we use both terms to encompass the full range

Ecosystem Functional Group (EFG)	General comment	Specific comments	Response type	Detailed response
Biome T4 Savannas and grasslands	Overall this reads well. Here my minor suggestions	Terminology: very good that you apply tundra strictly to the arctic land cover north of the arctic treeline. I dislike the term being used outside that setting. I noticed however, that you are doing exatly this with Savanna. You apply a term that belongs to subtropical periodically dry woodland/grassland systems to any open woodland. I feel the term Savanna should be used for the Savanna proper only. It may include Australian and SAm subtropical open woodlands, but not outside the subtropics. In all other cases I suggest to use 'open woodland'	No change	We use a vernacular approach to nomenclature, aimed at terms that are familiar to the broadest possible range of users. "Savannas" are defined by the grassy character and associated functions, not by open woodland structure. Some savannas are treeless. For biome T4 we use the name "Savannas and grasslands", to encompass grassy ecosystems through the tropics and temperate zone
T4.1 Trophic savannas		P.19 Seems strange to only map out for Africa and India.	No change	The diverse and abundant megafauna that drive function of the trophic savannas are only present in Africa and Asia. The savannas of Australia and the Americas are structured primarily by fire
T4.1 Trophic savannas	I have had a look at all the savanna sections (4.1 Trophic; 4.2 Pyric; 4.3 Hummock). All descriptions are good; no major errors of omission or commission as far as I can see. The global terminology (Trophic; Pyric; Hummock) is appropriate.		No change	

Ecosystem Functional Group (EFG)	General comment	Specific comments	Response type	Detailed response
T4.1 Trophic savannas		Not quite clear to me why miombo and mopane are in another system	No change	Miombo and mopane are systems dominated by pyric tussock grasses and thicket shrubs with browsers and low-moderate densities of grazers. Hence they (T4.2) are shaped primarily by fire regimes, rather than by higher densities of mega-grazers that maintain patches of lawn grasses (T4.1) (see Archibald et al. 2013)
T4.2 Pyric tussock savannas	I examined all the tropical systems, especially those I personal experience with. I am especially knowledgeable about Latin America, and in particular, systems occurring in Peru. In general, sufficient variation is covered, and I found the product useful.	P. 20 Not all C4 grasses here are tussock forming.	Minor edits to text	Agreed, but text does not imply that tussocks are only grass forms. Added 'abundant' to clarify
T4.2 Pyric tussock savannas	I have had a look at all the savanna sections (4.1 Trophic; 4.2 Pyric; 4.3 Hummock). All descriptions are good; no major errors of omission or commission as far as I can see. The global terminology (Trophic; Pyric; Hummock) is appropriate.		No change	

Ecosystem Functional Group (EFG)	General comment	Specific comments	Response type	Detailed response
T4.2 Pyric tussock savannas		In my opinion, this unit could be merged with the previous one. It overlaps in many regions of the old world (Africa, Asia) and replaces the trophic savannas in Australia and Americas. In the Americas big herbivores were extinct when humans colonized the area about 12-13ky ago, so we can expect that savannas would also linked to the herbivory of big mammals as it is in Africa or Asia. I consider that to this biome level, both units should be merged	No change	Although the superficial appearance of Trophic (T4.1) and Pyric (T4.2) is similar, there are also substantial functional and structural differences that are well established in literature (see selected citations in profiles). The growth forms of grass, dominant groups of plant and animal biota, N-cycling, C-cycling, climatic and edaphic associations all differ. Distributions also differ, but include substantial overlaps. Megaherbivores may be present in pyric savannas (present or historically), but do not drive and engineer those systems to the degree they so for trophic savannas. Specialist savannas reviewers agree with the separation
T4.3 Hummock savannas		T4.3 hummock grasslands--again not sure that I recognise this as is effectively the infertile rocky component of T4.2 overlapping with the north Australian tropical shrublands of T3.1. Note repeat lines in first reference, and fires are certainly more frequent than decadal / multi-decadal. You might better state sub-decadal as per T4.2	Minor edits to text	Deleted 'multi-decadal' and added 'variable' decadal. This allows for some variation above an below 10 yr fire recurrence while maintaining a descriptor that is more frequent than fires in other savannas (sub-decadal). Corrected ref citation Lehmann et al
T4.3 Hummock savannas	I have had a look at all the savanna sections (4.1 Trophic; 4.2 Pyric; 4.3 Hummock). All descriptions are good; no major errors of omission or commission as far as I can see. The global terminology (Trophic; Pyric; Hummock) is appropriate.	The only real technical issue I had was in 4.3 re. "high C:N ratios". I discussed this with David this arvo. This is intuitively and, I assume, generally true. However, some arid zone plants are chockers with rubisco, and this would serve to decrease the C:N ratios. Now, I don't know if this is the case for Triodia in particular or whether any high-Rubisco species would serve to change the community averaged C:N ratio all that much.	Minor edits to text	Changed "...and high C:N ratios" to "...and mostly high C:N ratios, although N may be elevated in rubisco-enriched C4 grasses."

Ecosystem Functional Group (EFG)	General comment	Specific comments	Response type	Detailed response
T4.3 Hummock savannas		Also in South Island of New Zealand?	No change	This group is dominated by long-lived, spiny hummock grasses, confined to subtrpical, warm temperate cimates with strong 'winter' drought, and not present in New Zealand. The grasslands in South Island NZ are tussock systems that beling to group T4.5
T4.4 Temperate wooded savannas	Thanks for the opportunity to review some profiles for the forthcoming global ecosystem typology. I read the 4 profiles you asked me to look at. They are written well and are accurate summaries. I have a few comments on just one of them		No change	
T4.4 Temperate wooded savannas	Overall this reads well. Here my minor suggestions	Terminology: very good that you apply tundra strictly to the arctic land cover north of the arctic treeline. I dislike the term being used outside that setting. I noticed however, that you are doing exatly this with Savanna. You apply a term that belongs to subtropical periodically dry woodland/grassland systems to any open woodland. I feel the term Savanna should be used for the Savanna proper only. It may include Australian and SAm subtropical open woodlands, but not outside the subtropics. In all other cases I suggest to use 'open woodland'	No change	We use a vernacular approach to nomenclature, aimed at terms that are familiar to the broadest possible range of users. "Savannas" are defined by ter grassy character and associated functions, not by open woodland structure. Some savannas are treeless. For T4.4 we considered the name "Temperate wooded savannas", as the term "savannas" is applied increasingly by authors to emphasise the grassy character of temperate ecosystems and their analogies to tropical savannas. Ultimately, however, we opted for the more generic name "Temperate woodlands" to accommodate sites and phases in which grasses may not be abundant

**Ecosystem Functional Group (EFG)**	**General comment**	**Specific comments**	**Response type**	**Detailed response**
T4.4 Temperate wooded savannas		This unit is very heterogeneous as it is the result of a regular disturbance regime, regular fire and/or regular grazing. This entails that the canopy low density is determined by such disturbances and if they cease, vegetation succession will lead to closed forests. In this sense it is a fire and/or grazing dependent biome. As it is distributed in both hemispheres, the floristically and functionally heterogeneity is enormous. In addition to this, the text is written with an Australian perspective, highlighting the role of the fire, while in the northern hemisphere, particularly in Eurasia, such woodlands are linked to a particular husbandry regime which combines grazing of the herb layer, profiting from the seed of the trees and wood collecting for domestic fire or for charcoal making.	No change	Agree that this group is extensive and heterogeneous. However, it does not included anthropogenically modified forest (areas with such forest are identified as degraded examples within other functional groups). The primary limits on tree growth, stature and density are water deficit and/or cool temperatures. Fire is not a major driver (as it is in pyric systems), but may occur in some regions. These points are covered in the preamble of existing profile text
Biome T5 Deserts and semi-deserts		Correct the caption: Bahía de los Ángeles	Minor edits to text	Caption corrected to: Bahía de los Ángeles

Ecosystem Functional Group (EFG)	General comment	Specific comments	Response type	Detailed response
T5.1 Semi-desert steppes	These profiles look really cool. Thanks for letting me look at them. I think they are extremely well written and I have only a few comments	T5.1 semiarid steppe: Delete the stray ‘with’: 5th line of Key Ecosystem Drivers Ambient environment: here and elsewhere I would not be calling soils 'non-acid'. The soils are calcareous, this is the generally accepted terminology. I think the other defining feature of these landscapes that you’ve mapped is that limestone, in various forms e.g. indurated, is very close to the surface, and this drives hydrological processes and infiltration run-off relationships and vegetation patterns. It’s a major characteristics of North African steppe, the western US and large areas of semiarid eastern Australia.	Minor edits to text	Replaced non-acid with calcareous. Added sentence about role of indurated subsoil on infiltration runoff relationships
T5.2 Thorny deserts and semi-deserts	These profiles look really cool. Thanks for letting me look at them. I think they are extremely well written and I have only a few comments	T5.2 looks fine to me (except the term non-acid)	No change	
T5.3 Sclerophyll deserts and semi-deserts	These profiles look really cool. Thanks for letting me look at them. I think they are extremely well written and I have only a few comments	T5.3 looks good to me	No change	
T5.3 Sclerophyll deserts and semi-deserts		Spiny forest in southern Madagascar?	No change	Yes spiny 'forest' in sth Madagascar belongs to this group

Ecosystem Functional Group (EFG)	General comment	Specific comments	Response type	Detailed response
T5.4 Cool temperate deserts	These profiles look really cool. Thanks for letting me look at them. I think they are extremely well written and I have only a few comments	T5.4 The section on biocrusts is a bit US-centric and strangely detailed, and I'm assuming that it came from Belnap's paper. Biocrusts is one word. Actinobacteria and Proteobacteria are but two phyla that are very common in dryland soils globally. I would replace this section with the following: "Biocrusts with cyanobacteria, mosses, and lichens are prominent on fine-textured substrates and become dominant where it is too cold for vascular plants. They play critical roles in soil stability and water and nutrient availability."<br>I don't see reference to the encroachment of thorny, N-fixing shrubs across extensive areas of Eurasian grasslands in response to centuries of herbivory (e.g. Caragana spp.).	Minor edits to text	Replaced text as suggested. Added phrase to herbivore text: "with heavy grazing promoting replacement of grasses by N-fixing shrubs"
T5.5 Hyper-arid deserts	These profiles look really cool. Thanks for letting me look at them. I think they are extremely well written and I have only a few comments	T5.5 looks good to me	No change	

Ecosystem Functional Group (EFG)	General comment	Specific comments	Response type	Detailed response
T6.1 Ice sheets, glaciers and perennial snowfields	I think these read very well. They're short, and so miss some things but that's inevitable given the space available. I'd say you could put something in on governance of these environments (see the attached note on subglacial lake governance, which you might find useful).	No specific comment	No change	
T6.1 Ice sheets, glaciers and perennial snowfields	The IUCN global ecosystem typology looks like a great idea and initiative. I kept my focus on the T6.1 section, but scanned through a couple of other sections for comparison.	1. The reference used for T6.1 is from 2014 when the concept of biome classification for glaciers and ice sheets is proposed. I think that the overall description of the habitats in T6.1 fits in well with the paper. However, I would also suggest to use a follow-up paper, where we make a better distinction of the habitats within glaciers and name the ecosystem engineers (i.e., organic matter producers) for the different types of habitat.<br>2. There is also an updated description of the feedback between microbial growth and ice melting that I think would be good to be included.<br>3. I think it is worthy to mention carbon fixation in this context. Somehow, I felt that the role N-fixation is a bit elevated in the description of T6.1. I agree it is important, but C-fixation is more relevant and far more a factor that provides feedback between biology, chemical	Multiple edits to text and refs	1. Added the following text about habitats: "On the surface, the main primary producers are snow (mainly Chlamydomonadales) and ice algae (mainly Zygnematales) with contrasting traits." and the following"Surface heterogeneity and dynamism create cryoconite holes, rich oases for microbial life (especially cyanobacteria, ppokaryotic heterotrophs and viruses) and active biogeochemical cycling."<br>2. Added the following text: "Snow and ice algae and cyanobacteria on the surface are ecosystem engineers. Their accumulation of organic matter, leads to positive feedbacks between melting and microbial activity that discolours snow and reduces albedo. Organic matter produced at the surface can also be transported through the ice to dark subglacial environments, fuelling microbial processes involving heterotrophic and chemoautotrophic prokaryotes and fungi."<br>3. Carbon fixation addressed in the text added in response to point 2<br>4. Anesio et al. (2017) added to reference list<br>5. Unfortunately there was insufficient space to add Stibal et al.<br>6. Deleted the following sentence: “Pinnipeds and polar bears utilise sea ice for breeding and feeding”<br>7. Added mention of P-limitation as follows: Nutrients, especially N "and P", are extremely scarce, ...

Ecosystem Functional Group (EFG)	General comment	Specific comments	Response type	Detailed response
		composition and physical aspect of the ice.<br>4. The newer reference I would suggest to include is: https://www.nature.com/articles/s41522-017-0019-0<br>5. There is another perspective small paper just coming out in Nature Ecology and Evolution by Stibal et al. and I will send this as soon as it is out in a couple of weeks.<br>6. There is nothing particularly that I feel is wrong,but there are a couple of sentences referring to sea ice that are out ofcontext in T6.1. Sea ice is described in M2.5 and mention of sea ice in T6.2distracts from information about glaciers and ice sheets. The example is thesentence below: “Pinnipeds and polar bears utilise sea ice for breeding and feeding” – sentence is out of context, considering that the chapter is about ice sheets and glaciers.<br>7. Finally, I would also mention that the system may be P-limited, in addition of N limited (at the surface of the ice).		

Ecosystem Functional Group (EFG)	General comment	Specific comments	Response type	Detailed response
T6.2 Polar-alpine rocky outcrops	I examined all the tropical systems, especially those I personal experience with. I am especially knowledgeable about Latin America, and in particular, systems occurring in Peru. In general, sufficient variation is covered, and I found the product useful.	P. 31 I don’t think viruses warrant mention	Minor edits to text	Deleted viruses
T6.2 Polar-alpine rocky outcrops	I think these read very well. They're short, and so miss some things but that's inevitable given the space available. I'd say you could pt something in on governance of these environments (see the attached note from me and Chuck Kennicutt on subglacial lake governance, which you might find useful).	No specific comment	No change	
T6.2 Polar-alpine rocky outcrops	The IUCN global ecosystem typology looks like a great idea and initiative. I kept my focus on the T6.1 section, but scanned through a couple of other sections for comparison.		No change	

Ecosystem Functional Group (EFG)	General comment	Specific comments	Response type	Detailed response
T6.2 Polar-alpine rocky outcrops		cliffs and screes occur also in lower altitudes, they can even reach the sea-level in mid-latitudes. The spscificity of cliffs and screes is no the cold but the rocky conditions: fissures and accumulation of stones, they determine the true conditions of the cliffs and screes.	No change	Cliffs and screes in high alpine and polar settings are highly distinctive from their ice-covered surrounds (T6.1) in terms of their biota, physical conditions and processes. Extreme cold temperatures and high winds are key drivers. In non-cryogenic landscapes, these landforms are capable of supporting a wider range of biota (e.g. vascular plants, invertebrates, nesting birds), including species from adjacent ecosystems, and their weathering processes are different, freezing temperatures is not a major driver so much as desiccation. Mass movement is common to both, but even here the mechanisms differ somewhat. Cliffs, screes and other rocky landforms in ice-free landscapes are included in functional group T3.4, in recognition of these differences.
T6.3 Polar tundra	Overall this reads well.	Terminology: very good that you apply tundra strictly to the arctic land cover north of the arctic treeline. I dislike the term being used outside that setting.	Minor edits to text	1. We use 'tundra' in this strict sense in the arctic and also apply it to more restricted analogous treeless ecosystems in the subantarctic and antarctic<br>2. Added the following text: "Tundra around the world, is delimited by the physiological temperature limits of trees, which are excluded where the growing season (i.e. days >0.9°C) is less than 90-94 days duration, with mean temperatures less than 6.5°C across the growing season. ." Added Paulsen & Korner (2014) to the reference list
T6.4 Temperate alpine grasslands and shrublands	I examined all the tropical systems, especially those I personal experience with. I am especially knowledgeable about Latin America, and in particular, systems occurring in Peru. In general, sufficient variation is covered, and I found the product useful.	P. 34 I doubt it is high LAI by global standards	Minor edits to text	LAI is high for single-layered vegetation, but higher in multi-layered forests. Deleted to avoid confusion

Ecosystem Functional Group (EFG)	General comment	Specific comments	Response type	Detailed response
T6.4 Temperate alpine grasslands and shrublands		1. Rephrased "Treeless mountain systems are dominated by..." as follows: "Mountain systems beyond the cold climatic treeline are dominated by ..."<br>2. Replaced "meadow" with "grassland" throughout<br>3. Added the following text: "Throughout the world, alpine ecosystems are defined by the physiological temperature limits of trees, which are excluded where the growing season (i.e. days >0.9°C) is less than 90-94 days, with mean temperatures less than 6.5°C across the growing season." Added Körner (2012) and Paulsen & Körner (2014) to the reference list	Minor edits to text and refs	1. Rephrased "Treeless mountain systems are dominated by..." as follows: "Mountain systems beyond the cold climatic treeline are dominated by ..."<br>2. Replaced "meadow" with "grassland" throughout<br>3. Added the following text: "Throughout the world, alpine ecosystems are defined by the physiological temperature limits of trees, which are excluded where the growing season (i.e. days >0.9°C) is less than 90-94 days, with mean temperatures less than 6.5°C across the growing season." Added Korner (2012) and Paulsen & Korner (2014) to the reference list
T6.4 Temperate alpine grasslands and shrublands		This type in often merged with th previous one T6.3 and in a global system I would consider cenvenient to do so. There are units much more heterogeneous in this classification than the result of merging polar tundras with alpine meadows	No change	There are varied opinions among specialists on the distinction between tundra and alpine ecosystems. It is true that there is more variation among vegetated temperate alpine ecosystems than within tundra, but there are distinctive functional differences, including permafrost, insect abundance and roles, the role of seasonal sunlight variation, migratory herbivores and other fauna, as well as greater importance of regional species pools in alpine systems. Distinction of the two is well supported by cited literature and reviews of specialists.
T6.5 Tropical alpine meadows and shrublands		Map is small but also some of this on the volcanoes in Eastern DRC and Rwanda. See Afroalpine ecoregions in Africa for maps	Amendments to map	Yes, the map already sshows these occurrences (they are included in the Spatial data in Zenodo) archive, plus one nearby in Uganda (Rwenzori Mtns WHA), but they were too small to show up. An ellipse has been added to the map in the report to draw attention to these for display purposes.

**Ecosystem Functional Group (EFG)**	**General comment**	**Specific comments**	**Response type**	**Detailed response**
T7.1 Croplands	Thanks for the opportunity to review some profiles for the forthcoming global ecosystem typology. I read the 4 profiles you asked me to look at. They are written well and are accurate summaries. I have a few comments on just one of them		No change	
T7.1 Croplands	Overall, this reads well and is fine	1. "…these non-target biota, and in particular the weedy plants, can become dominant…" As written could suggest that other biota mentioned earlier – mice and starlings – become dominant, but I don't think that is the case.<br>2. 'soil organic carbon content."<br>3. "southern and southwestern Australasia…": Southern Australia covers all examples; is Australasia necessary, when also using Oceania?	Minor edits to text	1. Added phrase as follows: …these non-target biota,"especially weedy plants, "can become dominant...<br>2. Added word as follows: ...soil organic carbon "content."<br>3. Deleted "and southwestern" and corrected "Australasia" to "Australia"
T7.1 Croplands		An Anthrome and not a Biome?	Minor edits to text	We interpret "anthrome" as a type of biome shaped by human activity, but use biome throughout for consistency. Added text to Biome description to explain "anthrome

Ecosystem Functional Group (EFG)	General comment	Specific comments	Response type	Detailed response
T7.2 Sown pastures and old fields	Thanks for the opportunity to review some profiles for the forthcoming global ecosystem typology. I read the 4 profiles you asked me to look at. They are written well and are accurate summaries. I have a few comments on just one of them		No change	
T7.2 Sown pastures and old fields	Overall, this reads well and is fine	Change “Usually C3 perennial pasture grasses...” to “C3 and C4 perennial pasture grasses...”	Minor edits to text	Changed “Usually C3 perennial pasture grasses...” to “C3 and C4 perennial pasture grasses...”
T7.3 Plantations	I examined all the tropical systems, especially those I personal experience with. I am especially knowledgeable about Latin America, and in particular, systems occurring in Peru. In general, sufficient variation is covered, and I found the product useful.	P. 39 There are often tree plantations in Mexico and all through Andean countries, including the tropical ones. Often pine or eucalypts	Amendments to map	Occurrences in Andean countries added to map

Ecosystem Functional Group (EFG)	General comment	Specific comments	Response type	Detailed response
T7.3 Plantations		1. "…molecular engineering to…".<br>2. "Intermittent flooding may occur when pastures replace palustrine wetlands" – is this sentence carried over from another section? Suggest remove.<br>3. Distribution – "…southern Australiasia…"<br>4. Perhaps an additional comment would be that in some cases plantations can form an alternate habitat for endangered species, but because of harvesting they cannot be relied upon as an alternative. (Australian examples would be koalas in eucalypt plantations, or Carnaby's Cockatoos in pine plantations.) Additionally, local species may graze on plantations and thus controlled.	Minor edits to text	1. Added space between words as suggested<br>2. Deleted sentence as suggested<br>3. Replaced "Australasia" with "Australia"<br>4. Added the following sentence: "Cyclical harvest may render the habitat periodically unsuitable for some biota."
T7.4 Urban and infrastructure lands	I examined all the tropical systems, especially those I personal experience with. I am especially knowledgeable about Latin America, and in particular, systems occurring in Peru. In general, sufficient variation is covered, and I found the product useful.	P.40 Ecological effects of the cities go well out in countrysides that they influence and depend on	No change	The existing text describes these effects as follows: Unlike most other terrestrial ecosystems, the energy, water and nutrient sources of urban systems are highly allochthonous and processes within urban systems drive profound and extensive global changes in land use, land cover, biodiversity, hydrology, and climate through both resource consumption and waste discharge.

Ecosystem Functional Group (EFG)	General comment	Specific comments	Response type	Detailed response
T7.4 Urban and infrastructure lands	I love the general approach, combining top-down and bottom-up approaches, but there are significant problems with T1.1 and T1.3. I have minor additional comments and suggestions for T1.2, T1.4, T7.4	1. I am not sure what is meant by water being ‘highly allochthonous’: this is only really true in arid areas.<br>2. I think you should mention air pollution, both chemical and particulate, and perhaps also light and noise pollution.	Minor edits to text	1. Water is highly allochthonous because most cities pipe large volumes of water in from reservoirs or desalination plants. Large amounts of local rainfall are also redistributed by sealed surfaces<br>2. Added the following text: "Chemical and particulate air pollution, as well as light and noise pollution may affect biota."
T7.5 Semi-natural pastures and oldfields		This reads well. No suggested changes to the text. One small typo in the references: “Semi-natural vegetation in agricultural land: European map and links to ecosystem service supply”<br>Do you have one where grazed lands become dominated by woody plants? E.g. parts of southern Africa	Minor edits to refs	Reference corrected<br>Shrubs. That phenomenon is a bit more limited but common in some semi-arid parts of eastern Aust and US as well. We deal with it as a degraded state of the source system for various reasons, including the difficulty of distinguishing the features (and mapping)
T7.5 Semi-natural pastures and oldfields		Some of the national land use land cover classes of India could not be mapped under any of the classes. Examples<br>–Built-up : Rural, Quarries<br>–Barren/ Unculturable/ Wasteland : Gullied/ Ravinous, Dense / Closed and Open category of scrub land	Minor edits to text	Some landuse/land cover classes are detectable symptoms of human activity, and are not always ecosystems of themselves. These typically form part of agricultural, urban or industrial systems (Bioe T7), or else managed or degraded states of other ecosystem types (T1-T6). These anthropogeic land luse/land cover classes may be interpreted as patch types within these broader functional groups of ecosystems. To recognise these (and the mapping of such classes), we added an explicit list of patch types that may be recognised and mapped in fine-scale land use classifications. Added text as follows: These systems are structurally complex and highly heterogeneous fine-scale spatial mosaics of diverse patch types "that may be recognised in fine-scale land use classifications. These include: a) buildings; b) paved surfaces; c) transport infrastructure: d) treed areas; e) grassed areas; f) gardens; g) mines or quarries; h) bare ground; and i) refuse areas."

**Ecosystem Functional Group (EFG)**	**General comment**	**Specific comments**	**Response type**	**Detailed response**
T7.5 Semi-natural pastures and oldfields			No change	
Subterranean				
Biome S1 Subterranean lithic systems		1. "These systems exhibit low levels of environmental variability and physical stability..." Comment: This could be read as low levels of physical stability – which is not the case<br>2. "...no photoautotrophs and few predators." Few obligate predators perhaps – more generalists that are predatory"	Minor edits to text	1. Rephrased sentence to clarify stability as follows: "These physically stable systems exhibit low levels of environmental variability. Rarely, mass movements, for example rock falls, may re-organise the physical structure of subterranean ecosystems."<br>2. Changed "few predators" to "few obligate predators"

Ecosystem Functional Group (EFG)	General comment	Specific comments	Response type	Detailed response
S1.1 Aerobic caves	Nice job! This looks great.<br>Please see attached comments on SF1.1 and SF1.2.<br>You have done a great job summarising so much info into 1 page and my comments are not critical given the space limitations you have – except that I think up front SF1.1 should probably mention caves rather than waiting until the ecological drivers section.<br>I know that everything is on a continuum – and to me SF1.1 and 1.2 are on a continuum of void size that influences hydrogeology and the types and sizes of critters that can live there.	1. "...may be a few specialist predators confined to resource-rich hotspots" Comment: Yep…<br>see comment above [i.e. predators are ostly generalists]<br>2. "The majority of biota are obligate subterranean organisms that complete their life cycles below ground." Comment: Could add that<br>the proportion of surface spp decreases rapidly with distance in to the cave – non obligate spp are generally limited to near the cave entrance (ie the ecotone)<br>3. "These are generalist detritivores and some are also opportunistic predators, reflecting the selection pressure of food scarcity." Comment: Bit of a repeat from 2nd sentence so cold combine to save space if needed<br>4. "limestone or gypsum" Comment: Dolomite is also pretty common in Europe but is a carbonate like limestone	Minor edits to text	1. Agree. Specialists are rare and localised. Added exampes to emphasise the point as follows: there may be a few specialist predators confined to resource-rich hotspots, such as bat latrines of seeps.<br>2. Added the following sentence: "The relative abundance and diversity of temporary inhabitants decline rapidly with distance from the cave entrance."<br>3. This sentence includes addtional detail about generalist detritivores, hence we retained it<br>4. Added "dolomite" to "limestone or gypsum"
S1.1 Aerobic caves	No issues raised		No change	
S1.2 Endolithic systems	No issues raised		No change	
S2.1 Anthropogenic subterranean voids	No issues raised		No change	

Ecosystem Functional Group (EFG)	General comment	Specific comments	Response type	Detailed response
Subterranean-Freshwater				
Biome SF1 Subterranean freshwaters	Nice job! This looks great.<br>Please see attached comments on SF1.1 and SF1.2.<br>You have done a great job summarising so much info into 1 page and my comments are not critical given the space limitations you have – except that I think up front SF1.1 should probably mention caves rather than waiting until the ecological drivers section.<br>I know that everything is on a continuum –to me SF1.1 and 1.2 are on a continuum of void size that influences hydrogeology and the types & sizes of critters that can live there.	1. This is really the largest of all the freshwater biomes – in terms of area and volume of water<br>2. Add condition for fish occurrence, ", where voids in the subsurface are of sufficient size."	Minor edits to text	1. Added the following text to the first sentence: ...beneath the earth's surface :and potentially has the largest volume of water of all the freshwater biomes."<br>2. Added phrase to second last sentence as follows: Small predatory fish may occur in streams and lakes, "where voids in the subsurface are of sufficient size."

Ecosystem Functional Group (EFG)	General comment	Specific comments	Response type	Detailed response
SF1.1 Underground streams and pools	Nice job! This looks great.<br>Please see attached comments on SF1.1 and SF1.2.<br>You have done a great job summarising so much info into 1 page and my comments are not critical given the space limitations you have – except that I think up front SF1.1 should probably mention caves rather than waiting until the ecological drivers section.<br>I know that everything is on a continuum – and to me SF1.1 and 1.2 are on a continuum of void size that influences hydrogeology and the types and sizes of critters that can live there.	1. To me these are flooded caves in some way or other. I think this description would benefit from saying that up front.. maybe ‘large water filled cavities occurring in caves and other subsurface voids’<br>2. Microbial mats - These are very much a ‘cave’ phenomenon<br>3. Occurrence of transient vertebrates - And in which void size is big enough<br>4. Limestone - "Or other soluble rocks such as dolomite"	Multiple edits to text	1. Amended first sentence to mention flooded caves explicitly as fllows: "Subterranean streams, pools, and aquatic voids (flooded caves) are low-productivity systemsdevoid of light."<br>2. We describe microbial mats as a key feature of this group, but nt of groundwater systems (SF1.2)<br>3. Added fish to list of other biota with volume constraint as follows: ...molluscs, arachnids, "and fish in larger voids." Moved sentence on transient vertebrates to end of section and added phrase to acknowledge volume constraints: Transient vertebrates occur "only in waters of larger subterranean voids" that are well connected to surface streams with abundant food."<br>4. Rephrased cave formation sentence to acknowledge other soluble lithogies as follows: "...from chemical weathering of soluble rocks such as limestone or dolomite,..."

Ecosystem Functional Group (EFG)	General comment	Specific comments	Response type	Detailed response
SF1.2 Groundwater ecosystems	Nice job! This looks great.<br>Please see attached comments on SF1.1 and SF1.2.<br>You have done a great job summarising so much info into 1 page and my comments are not critical given the space limitations you have – except that I think up front SF1.1 should probably mention caves rather than waiting until the ecological drivers section.<br>I know that everything is on a continuum – and to me SF1.1 and 1.2 are on a continuum of void size that influences hydrogeology and the types and sizes of critters that can live there.	Nice summary. Maybe only comment to add is that nutrients and biota decrease with depth & distance rfom inputs. Some meiofauna found to depths of several Km but most meiofauna in ~100 m depth. Bacteria area everywhere but at densities several orders of magnitude lower than surface waters!	Minor edits to text	Replaced existing text ("Taxonomic diversity is low but increases with connectivity to surface waters.") with the following: "Diversity and abundance of biota decline with depth and connectivity to surface waters, as do nutrients (e.g. most meiofauna is limited to 100m depth)."
SF1.2 Groundwater ecosystems		Add the following to introductory sentence: "...are found below the groundwater level (phreatic zone). For example, they ..."	Minor edits to text	Added following phrase to first sentence: "...are found within or below groundwater (phreatic) zones"

**Ecosystem Functional Group (EFG)**	**General comment**	**Specific comments**	**Response type**	**Detailed response**
Biome SF2 Anthropogenic subterranean freshwaters		Include flooded mines in this biome	Minor edits to text	Amended text as follows: ...includes aquatic systems in underground canals, drains, sewers, water pipes, "and flooded mines "...<br>and as follows: ...low to high flow velocity "or, in the case of flooded mines, negligible flow. "
SF2.1 Water pipes and subterranean canals	Flooded mines are not currently accommodated in the typology.	Add flooded mines to title and include text to cover full range of variation	No change	Agree that flooded mines are an important gap. After consulting additional literature, we were convinced that these systems warrant a different functional group because of their more varied geochemistry, development with time since abandonment, lack of flowing water and insularity from other aquatic systems. We used the text provided by the reviewers as the nucleus of a new profile for that group

**Ecosystem Functional Group (EFG)**	**General comment**	**Specific comments**	**Response type**	**Detailed response**
SF2.2 Flooded mines and other voids	Flooded mines are not currently accommodated in the typology. Add the following text to address them: "Abandoned and now flooded underground mines occur in many mineral rich regions of the world. Such mines frequently contain extensive reservoirs of geothermally warmed groundwater, colonized by stygobitic invertebrates from nearby natural subterranean habitats. During operation, pumping of water out of the mine formed a widespread cone of water table depression, with oxidation and hydrolysis of exposed minerals changing groundwater chemistry. When mines closed and dewatering ceased, they flooded and the water table began to rise, rebounding into the former cone of depression. Further changes in water chemistry occurred after flooding due to dissolution and flushing	Add flooded mines to title and include following text to cover full range of variation: "Abandoned and now flooded underground mines occur in many mineral rich regions of the world. Such mines frequently contain extensive reservoirs of geothermally warmed groundwater, colonized by stygobitic invertebrates from nearby natural subterranean habitats. During operation, pumping of water out of the mine formed a widespread cone of water table depression, with oxidation and hydrolysis of exposed minerals changing groundwater chemistry. When mines closed and dewatering ceased, they flooded and the water table began to rise, rebounding into the former cone of depression. Further changes in water chemistry occurred after flooding due to dissolution and flushing of the oxidation products. Although acid mine drainage is a common cause pollution of surface rivers and streams, few studies have been done to investigate the aquatic fauna and ecology of the quasi-stagnant water stored within mine workings.	Drafted additional profile	See full text, diagram, map and references in profile

Ecosystem Functional Group (EFG)	General comment	Specific comments	Response type	Detailed response
	of the oxidation products. Although acid mine drainage is a common cause pollution of surface rivers and streams, few studies have been done to investigate the aquatic fauna and ecology of the quasi-stagnant water stored within mine workings.			

Ecosystem Functional Group (EFG)	General comment	Specific comments	Response type	Detailed response
Subterranean-Marine				
Biome SM1 Tidal subterranean systems	Needs broadening to include the full range of variation	1. Add "pools" to subterranean voids<br>2.Add: sunlight is "mostly" absent<br>3.Add: "... terrestrial aquifers (SF1), and surface coastal habitats " to allochthonous energy sentence<br>4. Add the following text: "Food and energy availability are influenced by in situ microbial processing (biogeochemical transformation) of these allochthonous organic matter inputs."<br>5. Add the following text to the marine interface sentence: "...a typical feature of coastal aquifers and subterranean estuaries..." and "...that is the primary zone of biogeochemical cycling"<br>6. Add the followig sentence: "In carbonate and volcanic geologies, the salinity gradient can often be observed in the flooded pools, voids, and caves as a halocline (sharp salinity gradient in the water column)."<br>7. Move and amend the traits sentence as follows: "Physiological traits enabling osmotic regulation allow some species to transit across haloclines between the fresh- and saline waters. In lightless sections of the subterranean marine systems where photoautotrophs are absent, trophic webs are truncated."<br>8. Rephrase "Some of these organisms have close relatives on the deep sea floor ..." to "Some of the subterranean marine biota belong to lineages otherwise	Multiple edits to text	1. Added "pools" to subterranean voids<br>2.Added: sunlight is "mostly" absent<br>3.Added: "... terrestrial aquifers (SF1), and surface coastal habitats (T, MT)" to allochthonous energy sentence<br>4. Added the following text: "Food and energy availability are influenced by in situ microbial processing (biogeochemical transformation) of these allochthonous organic matter inputs."<br>5. Added the following text to the marine interface sentence: "...a typical feature of coastal aquifers and subterranean estuaries..." and "...that is the primary zone of biogeochemical cycling"<br>6. Added the following sentence: "In carbonate and volcanic geologies, the salinity gradient can often be observed in the flooded pools, voids, and caves as a halocline (sharp salinity gradient in the water column)."<br>7. Moved and amended the traits sentence as follows: "Physiological traits enabling osmotic regulation allow some species to transit across haloclines between the fresh- and saline waters. In lightless sections of the subterranean marine systems where photoautotrophs are absent, trophic webs are truncated."<br>8. Rephrased "Some of these organisms have close relatives on the deep sea floor ..." to "Some of the subterranean marine biota belong to lineages otherwise restricted to the deep sea floor ..."<br>9. Rephrased "Tides also influence the relative abundance of marine suspension feeders, particularly sponges and other sessile invertebrates." to "In subterranean tidal ecosystems with more direct connections to the sea, marine filter-feeders, particularly sponges and other sessile invertebrates dominate."<br>10. Added the following text: "Farther into marine and anchialine caves where tidal flushing and water exchange diminishes or disappears, the fauna consists of stygobitic crustaceans, annelids and several other faunal groups (e.g.

Ecosystem Functional Group (EFG)	General comment	Specific comments	Response type	Detailed response
		restricted to the deep sea floor ..."<br>9. Rephrase "Tides also influence the relative abundance of marine suspension feeders, particularly sponges and other sessile invertebrates." to "In subterranean habitats with more direct connections to the sea, marine filter-feeders, particularly sponges and other sessile invertebrates dominate."<br>10. Add the following text: "Farther into marine and anchialine caves where tidal flushing and water exchange diminishes or disappears, the fauna consists of stygobitic crustaceans, annelids and several other faunal groups (e.g. strictly subterranean aquatic fauna that complete their entire life in this environment). "		strictly subterranean aquatic fauna that complete their entire life in this environment). "
SM3.1 Anchialine caves		1. Delete "limited exposure to the open air"<br>2. Replace "streams and pools" with "freshwater"<br>3. Replace "biota include" with "dominant fauna includes"<br>4. Replace "connection. Sponges and hydroids occur in strongly tidal cave waters." with "Those caves closely connected with the ocean tend to have stronger tidal currents and biota such as sponges and hydroids commonly associated with sea caves"<br>5. Replace "Some biota have close relatives in deep ocean systems, including shrimps that retain red pigmentation in both systems." with "Some anchialine biota are related to deep sea species, while	Multiple edits to text, diagram and map	1. Deleted "limited exposure to the open air"<br>2. Replaced "streams and pools" with "freshwater"<br>3. Replaced "biota include" with "dominant fauna includes"<br>4. Replaced "connection. Sponges and hydroids occur in strongly tidal cave waters." with "Those caves closely connected with the ocean tend to have stronger tidal currents and biota such as sponges and hydroids commonly associated with sea caves"<br>5. Replace "Some biota have close relatives in deep ocean systems, including shrimps that retain red pigmentation in both systems." with "Some anchialine biota are related to deep sea species, including shrimps that retain red pigmentation, while others include relict taxa inhabiting anchialine caves on opposite sides of the Atlantic Ocean. Characteristic anchialine taxa also occur in isolated water bodies, far within extensive seafloor cave systems."<br>6. Replace "Salt concentration increases with depth (often with a sharp halocline boundary), declines with distance from the sea, and varies with tides and rainfall percolation" with "Sharp

Ecosystem Functional Group (EFG)	General comment	Specific comments	Response type	Detailed response
		others include relict taxa inhabiting anchialine caves on opposite sides of the Atlantic Ocean. Characteristic anchialine taxa also occur in isolated water bodies, far within extensive seafloor cave systems."<br>6. Replace "Salt concentration increases with depth (often with a sharp halocline boundary), declines with distance from the sea, and varies with tides and rainfall percolation" with "Sharp haloclines, which fluctuate with tides and rainfall, occur at deeper depths with increasing distance inland.<br>7. Delete "increasing stratification of the water column and "<br>8. Replace "Bedrock porosity influences the infiltration of fresh groundwater and hence salinity gradients." with "In karst terrains where surface runoff is absent, anchialine caves are intimately linked via hydrology to overlying subaerial coastal habitats and can serve as subterranean rivers with haloclines separating seaward flowing freshwater from underlying saltwater"<br>9. Replace "...but increase and become less variable..." with, "...increasing at the halocline and then stabilize..."<br>10. Map: Given that the color codes for these maps are described as "The maps show areas of the world containing major (coloured red) or minor occurrences (coloured yellow) of each ecosystem functional group" on page 5, I don't see		haloclines, which fluctuate with tides and rainfall, occur at deeper depths with increasing distance inland.<br>7. Delete "increasing stratification of the water column and "<br>8. Replace "Bedrock porosity influences the infiltration of fresh groundwater and hence salinity gradients." with "In karst terrains where surface runoff is absent, anchialine caves are intimately linked via hydrology to overlying subaerial coastal habitats and can serve as subterranean rivers with haloclines separating seaward flowing freshwater from underlying saltwater"<br>9. Replace "...but increase and become less variable..." with, "...increasing at the halocline and then stabilize..."<br>10. Map: Given that the color codes for these maps are described as "The maps show areas of the world containing major (coloured red) or minor occurrences (coloured yellow) of each ecosystem functional group" on page 5, I don't see how the current version of this map is indicative of the major and minor occurrences of anchialine caves. I suggest that the map be redrawn, eliminating the yellow shading and coloring Yucatan, Bahamas, Cuba and Hispaniola red.<br>11. Diagram: Added "In situ biogeochemical cycling" to Traits circle. Added arrow from Land surface box to Allochthonous inputs and labelled "food/energy"<br>• Added row from "Land surface" box to "Nutrients" box<br>• Added "Biogeochemistry" to Substrate box and to arrow from there to "Nutrients" box, as well as existing arrow feeding to "Trophic inputs"<br>Corrected typo 'chemotrophic'.<br>There are some significant functional differences that limit structural comparison. Aquifers are generally insular, with more limited biotic exchange with surface systems and none with marine systems. They also have fewer and smaller allochthonous sources of energy & nutrients. Anchialine caves are more similar to subterranean streams than groundwater aquifers due t limited free water in groundwater systems.

Ecosystem Functional Group (EFG)	General comment	Specific comments	Response type	Detailed response
		how the current version of this map is indicative of the major and minor occurrences of anchialine caves. I suggest that the map be redrawn, eliminating the yellow shading and coloring Yucatan, Bahamas, Cuba and Hispaniola red.<br>11. Diagram: This conceptual model is not entirely clear to me. I think it would benefit from adding in situ biogeochemical cycling. I think it also needs clarification that (in addition to occasional and cryptic chemoautotrophs, and marine inputs) subaerial coastal habitats can also be major sources of food/energy in anchialine caves. I could revise the diagram if I get the raw editable version of it. Major points I would address:<br>• Land surface also contributes to allochthonous inputs and nutrients, not just water.<br>• Biogeochemistry affects autochthonous and allochthonous (partially) sources.<br>Minor: typo in 'chemotrophic'.<br>Overall, I think this diagram should be comparable in many aspects to the freshwater aquifer ecosystem but in its current form it's hard to compare them		
Freshwater-Terrestrial				

Ecosystem Functional Group (EFG)	General comment	Specific comments	Response type	Detailed response
TF1.1 Tropical flooded forests and peat forests		P.54 I wonder if can be true that there is limited time for evolution. Palms are ancient and so palm-dominated wetlands are likely also ancient.	No change	The inference about limited time for evolutionary divergence comes from Page et al. (2014). The palms inhabiting forested wetlands are not entirely restricted to them, and likely could have evolved in older rainforest habitats. Indeed, the relatively few species endemic to these ecosystems prompted Page to suggest that this was because of their young age.
TF1.1 Tropical flooded forests and peat forests	I love the general approach, combining top-down and bottom-up approaches, but there are significant problems with T1.1 and T1.3. I have minor additional comments and suggestions for T1.2, T1.4, T7.4	No problems	No change	

Ecosystem Functional Group (EFG)	General comment	Specific comments	Response type	Detailed response
TF1.2 Subtropical/temperate forested wetlands		1. Why the quotation marks? suggest that this needs some additional words to ensure definition/meaning is clear<br>2. "fish kill" - worth providing very brief explanation of process?<br>3. Traits - you might like to note that herbaceous/ground layer plant diversity can be high, and includes various (distinct) communities that collectively transition wet-dry phases in both space and time (e.g. forming zones of vegetation based on elevation/water depth), with soil and aerial seedbanks, plus incoming floodwaters, being an important seed source for recruitment<br>4. Drivers - add "dispersal and" recruitment<br>5. Drivers - as well as water being crucial for dispersal and disturbance, i would also say that flooding provides temporary/seasonal habitat/refugia for organisms at critical points in their life cycles (e.g. fish spawning and bird breeding when floodplains are inundated).<br>6. Diagram - competition not mentioned in Biotic Interactions<br>7. Diagram - importance of water for connectivity and dispersal not mentioned in figure<br>8. we also review these systems here: Catford, J. A., J. Roberts, S. J. Capon, R. H. Froend, S. M. Windecker, and M. M. Douglas. 2017. Wetland vegetation of inland Australia in D. A. Keith, editor. Australian Vegetation. Cambridge.	Multiple edits to text	1. Replaced "terrestrial" with "dryland" to make more self-evident<br>2. Rearranged text to improve explanation as follows: "Nutrients and coarse detritus accumulate on floodplains during low flows, and may drive microbial blooms, leading to aquatic anoxia and fish kills, which may be extensive when flushing occurs."<br>3. Elaborated on ground layer as follows: "Forest understories vary from diverse herbaceous assemblages to simple aquatic macrophyte communities in response to spatial and temporal hydrological gradients, which influence the density and relative abundance of algae, hydrophytes and dryland plants".<br>4. Added "dispersal and" recruitment<br>5. Amended relevant sentence in Traits section: Plant and animal life histories are closely connected to inundation (e.g. seed-fall, germination fish-spawning and bird breeding are stimulated by flooding).".<br>6. Diagram – Added "competition" to Biotic Interactions box<br>7. Diagram – Added "connectivity" to arrow from Flood regimes box to traits. Dispersal already mentioned in Biotic interactions box connected by arrow from flood regimes and arrow to traits labelled 'Meta-community processes.<br>8. Local-scope reference not added due to space constraints

Ecosystem Functional Group (EFG)	General comment	Specific comments	Response type	Detailed response
TF1.2 Subtropical/temperate forested wetlands		1. Also, we now know that trees alter the composition of wetland sediments that makes it easier for invertebrates to withstand dry periods. See reference: Strachan et al. (2016) Fringing trees may provide a refuge from prolonged drying for urban wetland invertebrates. Urban Ecosystems, 19, 1213-1230<br>2. I think Blackwater events should be mentioned by name here and the connections to large amounts of coarse detritus and insufficient flows to oxygenate substrata should also be included, as these drive the microbial blooms, anoxia and fish kills<br>3. Many of these wetlands are seasonal and not connected to other waterbodies so they do not have fish. This should be stated here, as it makes other animals (e.g. turtles, odonata) the top aquatic predators in these wetlands. The absence of fish also makes them good breeding habitat for frogs. Freshwater turtles also use these wetlands and should be added to this list. They may aestivate in the dry bed of these wetlands.<br>4. this is true, but needs to be clear that this category is not only about riparian forested wetlands, but all palustrine wetlands. atm it reads as too selectively about floodplain wetlands.<br>5. what we refer to as 'closed canopy' i.e. tree-covered freshwater seasonal wetlands appear to fit in this category. They are not associated with rivers but are filled from	Multiple edits to text and diagram	1. Amended sediment text, "alteration of sediments, (e.g. surface micro-topography by the growth of large roots)". Added "creation of desiccation refuges for fauna" Insufficient space to add the reference on the detail<br>2. Amended text as follows to clarify "Nutrients and coarse detritus accumulate on floodplains during low flows, and may drive microbial blooms, leading to aquatic anoxia and fish kills, which may be extensive when flushing occurs." Blackwater event is primarily an Australian term, so a more widespread phenomenon, so have retained generic description<br>3. Rephrased as follows to add turtles and qualify fish: "Turtles, frogs, birds and sometimes fish exploit the alternation between aquatic and terrestrial phases"<br>4. Changes from "Riparian forests" to "Forested wetlands". Non-forested palustrine wetlands are addressed in other functional groups (e.g. FT1.3, FT1.4), as trees have a major effect on function (even though they interact and may be juxtaposed with forested systems on the same floodplains).<br>5. Agree these are important, albeit less common expressions of the functional group. The opening sentence makes it clear that they are included in this group, "These hydrophilic forests and scrubs have an open to closed tree or shrub canopy, 2–40 m tall, dependent on flood regimes or groundwater lenses." Amended text in Drivers section to make it clearer there: "These forests occur on floodplains, riparian corridors and disconnected lowland flats." Also: Water movement is critical for the connectivity and movement of biota, "while some groundwater-dependent forests are disconnected."

Ecosystem Functional Group (EFG)	General comment	Specific comments	Response type	Detailed response
		beneath by groundwater. They are also usually darkly coloured waters, coloured by humic substances coming from decaying vegetation. This means that the only primary producers beneath the trees are floating macrophytes. The description of this category does not appear to include these wetlands but it should as they dont fit anywhere else. They are or were found across southwestern Australia, especially on the Swan Coastal Plain and the sandplains on the south coast of western Australia. They are also found in SE Queensland. Further to this Palustrine wetlands may occur as seasonal or perennial expressions of groundwater tables that underly sandplains; these wetlands may have no surface water connections and are usually fishless.		
TF1.3 Permanent marshes	let me say this is a really cool project, and you've clearly made a wonderful start. I'll look forward to the final product!	TF1.3 Permanent marshes: Fires can be important drivers of these ecosystems, especially in low-gradient settings where microtopography is subtle and organic soils accumulate, like the Everglades. Here, large muck fires can burn regularly, commonly set off by lightning. These fires can burn persistently, locally both resetting vegetation and lowering ground-surface elevations as the organic soils and combusted.	Minor edits to text	This comment seems more relevant to TF1.4, in which substrates dry out and become flammable periodically. The soil remains permanently saturated in TF1.3 (by definition), so although the vegetation may burn, muck fires would be rare. Amended the final sentence to clarify as follows: "Surface fires may burn vegetation in some permanent marshes, but rarely burn the saturated substrate, and are less drivers of these ecosystems than seasonal marshes (TF1.4)."

Ecosystem Functional Group (EFG)	General comment	Specific comments	Response type	Detailed response
TF1.3 Permanent marshes		1. Traits - worth noting importance (or not) to migratory species? and to C storage?<br>2. Delete “long” rhizomes<br>3. “Many plants and animals disperse widely through the air and water.” - this statement seems a bit vague. do you mean dispersal within the marsh? so local? either cut or add clarifying text to contextualise<br>4. …cued “to” floods.<br>5. “wind-dispersed seeds” change to “wind- and water-dispersed seeds” adaptations for wind dispersal usually also enable water dispersal: e.g. Catford, J. A., and R. Jansson. 2014. Drowned, buried and carried away: effects of plant traits on the distribution of native and alien species in riparian ecosystems. New Phytologist 204:19-36<br>6. Diagram - which direction is the low energy arrow going in?	Multiple edits to text	1. Added “carbon storage” after “high primary production”. Added, “some of which are migratory” to Waterbirds sentence.”<br>2. Deleted “long” rhizomes at first mention, but retained second mention, as many plants have them in these systems – Phragmites, Typha, Cladium, Baumea, Cyperus, others<br>3. Added: disperse widely ”beyond the marsh”<br>4. Replaced “by” with “to”<br>4. Changed to “wind- and water-dispersed seeds”<br>6. “Low energy” arrow points right to left from box with “flat terrain” to box with “flood regimes”, as indicated by arrow head on latter box

Ecosystem Functional Group (EFG)	General comment	Specific comments	Response type	Detailed response
TF1.3 Permanent marshes		1. This is not quite right. Microbes are certainly limited as are gill-breathing invertebrates. However, what we see in these places are predominantly air breathing invertebrates. Things like pulmonate snails, many dipterans and coleopterans. I have suggested a change to the text here.<br>2. bunch of aquatic-feeding animals missing here. In SWWA, tiger snakes can be tremendously abundant in such wetlands (check out the episode of Deadly 60 when he visits Herdsman Lake). Our snake-necked turtle Chelodina colliei is also very abundant in such wetlands and mostly feeds in the water.<br>3. zoochory is common in these types of environments. Ref: Van Leeuwen et al. (2013) How did this snail get here? Several dispersal vectors inferred for an aquatic invasive species. Freshwater Bology, 58, 88-99.<br>4. Are there not of permanent marshes areas in top end? Need to add these areas.	Multiple edits to text and diagram	1. Accepted suggested change and added detail from this comment as follows: "Air-breathing invertebrates are more common than gill-breathers, due to low dissolved oxygen. The activity of microbial decomposers is also limited by ..."<br>2. Added snakes and turtles<br>3. Added "zoochory (e.g. birds, mammals)"<br>4. Seasonal marshes are much more extensive in this area, although some permanent ones exist. Their occurrence is too small to map at this scale

**Ecosystem Functional Group (EFG)**	**General comment**	**Specific comments**	**Response type**	**Detailed response**
TF1.4 Seasonal floodplain marshes	let me say this is a really cool project, and you've clearly made a wonderful start. I'll look forward to the final product!	TF1.3 Permanent marshes: Fires can be important drivers of these ecosystems, especially in low-gradient settings where microtopography is subtle and organic soils accumulate, like the Everglades. Here, large muck fires can burn regularly, commonly set off by lightning. These fires can burn persistently, locally both resetting vegetation and lowering ground-surface elevations as the organic soils and combusted.	Minor edits to text	See response to TF1.3. Added text, "Fires may occur in dry seasons, releasing resources, changing vegetation structure and composition, consuming organic substrates and lowering the wetland surface"
TF1.4 Seasonal floodplain marshes		1.Traits - see my comments on TF1.2 about zones of vegetation/distinct communities in space/time<br>2. “traits that promote predator avoidance and tolerance to low water quality” - if you can fit in, some brief examples here would be great<br>3. Diagram - top of this fig seems to have been sliced off<br>4. “rely” not “relay”<br>5. Map - yellow area in the Australia map doesn't match the description of humid temperate	Multiple edits to text	1. Local zonation is addressed in text as follows” These gradients shape ecosystem assembly by enabling species with diverse life-history traits to exploit different niches, resulting in strong local zonation of vegetation and high patch-level diversity of habitats for consumers.”<br>2. Deleted “and possess traits that promote predator avoidance and tolerance to low water quality” as no examples could readily be found in literature.<br>3. Diagram – corrected image<br>4. Replaced “relay” with “rely”<br>5. Distribution – corrected “humid” to “subhumid”

Ecosystem Functional Group (EFG)	General comment	Specific comments	Response type	Detailed response
TF1.4 Seasonal floodplain marshes		1. invertebrates are more than crustaceans and insects, and zoops are crustaceans and rotifers. What about molluscs, the wide variety of water mites and worms? I do not see the need to specifically identify 2 groups out of all that are often very diverse in such wetlands?<br>2. but not aquatic mammals? I must make a note to tell the rakali that the typology says that they mustnt use seasonal marshes, no matter how many tasty molluscs and crayfish live there. What about coypu? capybara? hippo? Instead, insert aquatic mammals and forget the terrestrial spp. Less important to mention them here when we have important groups of aquatic mammls that should be included.<br>3. not just eggs, ephippia or dormancy in adult or larval stages. needs to be more general to encompass these other life forms. We find adult crayfish and isopods for example, also adult ostracods aestivating in sediments.<br>4. this is true, but all put together they are still less diverse and conduct less ecological function than the invertebrates, which are also very diverse in this ecosystem type, so should be mentioned here.<br>5. in Australia, both the top end and southern states, many of these floodplain wetlands are alongside estuaries and span the full gradient from fresh to saltwater	Multiple edits to text and diagram	1. Replaced "crustaceans, insects" with "aquatic invertebrates"<br>2. Added "aquatic mammals", replaced "terrestrial birds and mammals" with "terrestrial animals"<br>3. Deleted "as eggs" to generalise<br>4. Added invertebrates to list of abundant and diverse taxa<br>5. Estuarine saltmarshes are described in functional groups MFT1.3 and brackish reed marshes occupy the transition between that group and FT1.4. Gradients such as this are pervasive throughout the biosphere. Discrete classifications are an artificial means of representing continuous variation, but nonetheless practically very useful. We address this throughout the typology by describing variation within and between related functional groups. In this case we have added text to highlight the importance of extended salinity gradients in floodplain marshes adjoining estuaries, and note the brackish transition between this and estuarine marshes (MFT1.3) or fringing vegetation of coastal lagoons (FM1.2, FM1.3).Added "Salinity gradients and tides influence these marshes where they adjoin estuaries, with brackish marshes on transitions to TF1.2, TF1.3 and MFT1.3. "<br>6. See pont 5. Added, "Salinity gradients and tides influence these marshes where they adjoin estuaries, with brackish marshes on transitions to TF1.2, TF1.3 and MFT1.3. "<br>7. Bgean new sentence with "Disconnected…"<br>8. Seasonal variation is mentioned in first sentence of the Drivers section. The point is that seasonal floods vary between years. Added "Seasonal" to beginning of sentence: :Seasonal flood extent and duration varies…" .<br>9. Tidal influence now mentioned in the text (see points 5 & 6)<br>10. Deleted sentence, "Flooding often occurs in spring…"<br>11. The area mapped in SE Australia is the easter Murray Darling Basin, not mountains (not project is equal area and causes some distortion). Added areas along south coast of East and west Australia

Ecosystem Functional Group (EFG)	General comment	Specific comments	Response type	Detailed response
		both spatially and temporally. These wetlands are very productive and important - as the text above says - but this type of wetland is missing from the whole classification system. I think this section should be expanded to include these wetlands especially as they are not included in the marine-freshwater ecosystem types.<br>6. and tidal inundation (or the interaction between the two)<br>7. even before I added a clause, this sentence did not make sense and needs to be divided into two.<br>8. they also vary tremendously with season, regardless of the wet-dry tropics or southern states of Australia.<br>9. systems like the Gellibrand River in Victoria or the Serpentine River in SWWA have flooplain wetlands along the lowland river and into and along the estuary to the ocean mouth. Some (e.g. Creery wetlands, Vasse-Wonnerup wetlands) are Ramsar sites. So, these cannot be ignored and they belong here. salinity is a very influential factor, closely related to temporal changes in hydrology and whether the water is coming from river flooding or tidal flooding.<br>10. this is very true in some regions, but equally it is very untrue in some other areas. across the whole of one continent, this is true for the top half but not for the southern half. This just seems like too sweeping a generalisation. I dont see why		

Ecosystem Functional Group (EFG)	General comment	Specific comments	Response type	Detailed response
		this statement is necessary or what it adds? It would be better to use the words to properly encompass all wetlands of this type, by removing this and including the estuary floodplain wetlands.<br>11. again, this is a very odd map, it says that in Australia, the only places that there are floodplain wetlands is in the mountains of the south east and in the top end. These wetlands occur in every river along the south coast of Victoria. For example, the Gellibrand river has an amazing set of floodplain wetlands that run from freshwater to saltwater in a longitudinal gradient. In spring flooding all the wetlands become freshwater, in autumn only the tidal wetlands are inundated. If the river mouth is closed, then the wetlands can be overflowing with water, creating management problems. There are also many rivers on the south and south west coasts of WA and the east and southeast coast of Victoria and Tasmania that have these wetlands along lowland rivers down into the estuaries. Redo the map for Australia.		

Ecosystem Functional Group (EFG)	General comment	Specific comments	Response type	Detailed response
TF1.4 Seasonal floodplain marshes		I miss the importance for fish breeding supported by the flushing of production after flooding. Example:<br>Castello L, Isaac VJ, Thapa R. (2015). Flood pulse effects on multispecies fishery yields in the Lower Amazon. Royal Society Open Science 2: 150299<br>McClain, M. E., & Naiman, R. J. (2008). Andean Influences on the Biogeochemistry and Ecology of the Amazon River. BioScience, 58(4), 325–338. https://doi.org/10.1641/b580408	Minor edits to text	Added reference to fish breeding as follows: Reproduction and recruitment, "especially of fish," coincide with food availability cued by flood regimes.

Ecosystem Functional Group (EFG)	General comment	Specific comments	Response type	Detailed response
TF1.5 Episodic arid floodplains	Firstly, I commend this enterprise; it will undoubtedly be very useful in many ways. Congratulations to all for an amazing product.<br>My 'review' is quite minor, due to other time commitments. It comprises comments on so the sections named above, provided as Sticky Notes in the attached IUCN documents, and further comments in the second document attached.<br>The conceptual models are impressive and will no doubt be elaborated as users of this material develop more detailed descriptions of the categories of rivers and streams over time. I have added a few extra references to some of the categories assigned to me, and to the section on artesian springs.<br>I do feel that more types of waterbody may need to be added, such as dune lakes and ICOLLS.	I found the F1 categories to be sufficient to cover most stream and river types. Needs a reference on the dependence of many fish species on inundated floodplains as habitat/food resources for larval, juvenile and mature fish. Production of fish biomass is related to area of floodplain inundated and length of time inundated.e.g. Arthington, A. H. and Balcombe, S. 2011. Extreme flow variability and the 'boom and bust' ecology of fish in arid-zone floodplain rivers: a case history with implications for environmental flows, conservation and management. Ecohydrology 4: 706-720.	Minor edits to text and map	Added the suggested reference

**Ecosystem Functional Group (EFG)**	**General comment**	**Specific comments**	**Response type**	**Detailed response**
	Overall I found the full suite of Ecosystem Functional Groups to be adequate at this level of resolution.<br>I found the F1 categories to be sufficient to cover most stream and river types. I have added comments on some of the entries (e.g. about energy sources, habitat structure, importance of floodplain inundation, and incidence of dystrophic waters and their low biodiversity but often high levels of endemicity). I have also suggested a few more references in some F1 ecosystem types.<br>The flow charts of processes are very good, but on the whole I found them to be rather similar across the F1 categories. I can see that it would be difficult to make them any more distinctive at this level of resolution.<br>I hope my brief contributions are useful to the project.			

Ecosystem Functional Group (EFG)	General comment	Specific comments	Response type	Detailed response
TF1.5 Episodic arid floodplains		Traits – “worth mentioning birds that opportunistically use these wetlands in their thousands?”	Minor edits to text	Added: …are highly mobile “in large numbers” or…
TF1.5 Episodic arid floodplains		1. I added ostracods and copepods as they will be more diverse and abundant than either anostraca or notostracans<br>2. Reptiles? Cooper Creek contains turtles, for example<br>3. So arid floodplain wetlands are less common in arid regions? I assume this is a typo - someone has deleted the word 'outside' without changing the text or similar?	Minor edits to text	1, Used broader term, "invertebrate consumers"<br>2. Added reptiles<br>3. Episodic floodplains are more common in semi-arid and arid regions than hyper-arid regions. Rephrased as follows: "Connected to ephemeral rivers in semi-arid and arid regions of all continents". Deleted "less common in arid areas".

Ecosystem Functional Group (EFG)	General comment	Specific comments	Response type	Detailed response
TF1.6 Boreal, temperate and montane peat bogs		1. I would add caddisflies here too<br>2. snakes important in australia. tiger snakes love peat wetlands<br>3. You have missed the south coast peat bogs in australia's only global biodiversity hotspot. these are host to the albany pitcher plant, the sunset frog and species of SR endemic crayfish Engaewa, for example. The regions where these occur are scott river national park, d'entrcasteaux national park etc. Basically from Augusta to Esperance. But there are also peat bogs elsewhere in the southwest and on the Fleurieu Peninsula in south Australia. I have added references. Please alter the map for Australia.<br>Refs: Pemberton (2005) Australian peatlands: a brief consideration of their origin, distribution, natural values and threats. J. Roy. Soc. WA., 88, 81-89. Pinder 2005 A review of biodiversity in wetlands with organic sediments on the Swan Coastal Plain, Western Australia, with an emphasis on aquatic invertebrates. Journal of the Royal Society of Western Australia, 88:129.132.	Minor edits to text and map	1. Replaced lepidopterans with caddisflies, as latter are more important<br>2. Replaced "skinks" with "reptiles" to include snakes. Some are habitat specialists, other not<br>3. Bogs known from this area and Fleurieu Peninsula (SA) were omitted from indicative map due to their small extent. Relevant ecoregion now added for Windy Hbr-Walpole-Albany area

Ecosystem Functional Group (EFG)	General comment	Specific comments	Response type	Detailed response
TF1.6 Boreal, temperate and montane peat bogs	I think the freshwater EFGs don't require any other additions. The only one I would question is F2.2. I read through the text of F1 to F6, FT1.6 & 7, and F3.1 to 3.5. The text is general enough to capture the variety within any one EFG. I think some additions to the text on the invertebrate fauna of TF1.6 & TF1.7 is required. I have specific comments on the attached pdf.	1. Lower case on carbon<br>2. Forms or promote (typo)<br>3. Algae can form an important component of the food web in bog pools.<br>4. most are carnivores<br>5. mix of scientific and common names<br>6. Need to better capture the characteristics of invertebrate fauna<br>7. Note that these are inhabitants of the open-water (pools) within the peat bog [of lepidoptera I think]<br>8. Pool size, numbers and types in a pool system complex and water level stability dare key factors that determine the invertebrate assemblages.	Multiple edits to text	1. Changed "Carbon" to lower case<br>2. Corrected typo<br>3. Added text as follows: "Pools within the bogs support specialised aquatic food webs underpinned by algal production and allochthonous carbon. "<br>4. Deleted reference to herbivores<br>5. Used common names<br>6. Replaced existing text, "Invertebrates are dominant consumers in the trophic network, with dipterans, dragonflies, and lepidopterans prominent herbivores as larvae and pollinators and predators as adults.", with "Invertebrate larvae are prominent consumers in the trophic network of bog pools, and as adults they are important pollinators and predators. Assemblages of flies, dragonflies, damselflies, caddisflies and other invertebrates vary with the number, size and stability of pools."<br>7. Text now acknowledges inhabitants of the open-water (pools) within the peat bog and explicit mention of lepidopterans was removed.<br>8. See response to point 3

Ecosystem Functional Group (EFG)	General comment	Specific comments	Response type	Detailed response
TF1.7 Boreal and temperate fens	I think the freshwater EFGs don't require any other additions. The only one I would question is F2.2. I read through the text of F1 to F6, FT1.6 & 7, and F3.1 to 3.5. The text is general enough to capture the variety within any one EFG. I think some additions to the text on the invertebrate fauna of TF1.6 & TF1.7 is required. I have specific comments on attached pdf.	see comment in TF1.6. Need to better capture the characteristics of the invertebrate fauna. For example there are well-known calcareous specialists of fens - e.g. snails of the genus Vertigo	Minor edits to text	Replaced the existing text with the following: "Invertebrates are dominant consumers in the trophic network, including dragonflies, caddisflies, flies, as well as calcareous specialists such as snails."
TF1.7 Boreal and temperate fens	I examined all the tropical systems, especially those I personal experience with. I am especially knowledgeable about Latin America, and in particular, systems occurring in Peru. In general, sufficient variation is covered, and I found the product useful.	P.60 Fens are also in tropical Andes, just not formally described yet (many things called bogs there are probably really fens by international standards).	Minor edits to text	Added "Fens may also occur in tropical mountains (e.g. Andes), but are poorly known there." to Distribution text

Ecosystem Functional Group (EFG)	General comment	Specific comments	Response type	Detailed response
Freshwater				
Biome F1 Rivers and streams		I hope I have not gone over the top here in trying to provide a broader overview of the river and streams systems. Apologies if I have! The interaction with the surrounding landscape is fundamental to the ecosystem function and energy and nutrient dynamics. And the longitudinal changes help to provide a background to separate different lotic biomes.	Multiple edits to text	Added detail on stream order, climate/weather, organic matter properties, variation between upland/lowland, exchanges with riparian systems, anthropogenic influences, benthos and alignment of life history processes with resource supply.
Biome F1 Rivers and streams		Some rivers flow into inland lakes (endorheic systems) or coastal lakes and lagoons<br>Elaborate, "Stream gradients determine flow velocity and turbulence, bank and substrate structure, and habitat variability..."<br>I believe that most studies show primacy of autochthonous energy sources even in headwater streams with high riparian cover. "<br>Add to erosions and sedimentation text, "...and to aquatic habitat diversity and patchiness"	Minor edits to text	Lakes' cover endorhec and coastal connected lakes, limited space for elaboration.<br>Added text on bank structure and habitat variability'<br>Amended energy source text to,"Productivity tends to increase from uplands to lowlands and is driven both by allochthonous energy sources that contribute coarse organic matter from terrestrial ecosystems in adjacent riparian zones and upper catchments, and by autochthonous energy synthesis by biofilms or phytoplankton."<br>Added suggested text to erosion & sedimentation sentence"

Ecosystem Functional Group (EFG)	General comment	Specific comments	Response type	Detailed response
F1.1 Permanent upland streams	Firstly, I commend this enterprise; it will undoubtedly be very useful in many ways. Congratulations to all for an amazing product.<br>My 'review' is quite minor, due to other time commitments. It comprises comments on so the sections named above, provided as Sticky Notes in the attached IUCN documents, and further comments in the second document attached.<br>The conceptual models are impressive and will no doubt be elaborated as users of this material develop more detailed descriptions of the categories of rivers and streams over time. I have added a few extra references to some of the categories assigned to me, and to the section on artesian springs.<br>I do feel that more types of waterbody may need to be added, such as dune lakes and ICOLLS.<br>Overall I found the full	I found the F1 categories to be sufficient to cover most stream and river types. F1.1 diagram: Benthic organs?? Not sure what this means. Perhaps organs to enable benthic attachment?	Minor edits to diagram	Changed 'Benthic organs' to "Bentic organisms dominant"

Ecosystem Functional Group (EFG)	General comment	Specific comments	Response type	Detailed response
	suite of Ecosystem Functional Groups to be adequate at this level of resolution.<br>I found the F1 categories to be sufficient to cover most stream and river types. I have added comments on some of the entries (e.g. about energy sources, habitat structure, importance of floodplain inundation, and incidence of dystrophic waters and their low biodiversity but often high levels of endemicity). I have also suggested a few more references in some F1 ecosystem types.<br>The flow charts of processes are very good, but on the whole I found them to be rather similar across the F1 categories. I can see that it would be difficult to make them any more distinctive at this level of resolution.<br>I hope my brief contributions are useful to the project.			

Ecosystem Functional Group (EFG)	General comment	Specific comments	Response type	Detailed response
F1.1 Permanent upland streams	let me say this is a really cool project, and you've clearly made a wonderful start. I'll look forward to the final product!	F1.1 Permanent upland streams: Some of our recent work is underscoring the important of groundwater-delivered subsidies. These subsidies support streamflow, with as much as 50% of summertime flow and 100% of wintertime flow coming originating as groundwater; modulate stream temperatures, keeping temperatures lower in summer and higher in winter; and deliver nutrient, especially if there are N-fixing species like alder along the groundwater flow path.	Minor edits to text	Added the following text, "Groundwater-delivered subsidies support streamflow, with up to 50% of summer flow and 100% of winter flow originating as groundwater. This modulates stream temperatures, keeping temperatures lower in summer and higher in winter; and deliver nutrients, especially if there are N-fixing plants along the groundwater flow path. "
F1.1 Permanent upland streams	I hope I have not gone over the top here in trying to provide a broader overview of the river and streams systems. Apologies if I have! The interaction with the surrounding landscape is fundamental to the ecosystem function and energy and nutrient dynamics. And the longitudinal changes help to provide a background to separate different lotic biomes.	Additional descriptive detail on stream order, edits to productivity contributions and variability, riparian vegetation, queried generalisation on endemism and phytoplankton	Major revisions to text and diagram	Added range of stream orders (1-3), deleted references to high endemism from text & diagram, added detail on importance and variability of leaf fall, added detail on characteristic biota, added detail on energy subsidies and exports to text and diagram, added biotic interactions to diagram, added detail on flood disturbance to text, added info of stream length to Distribution text

Ecosystem Functional Group (EFG)	General comment	Specific comments	Response type	Detailed response
F1.1 Permanent upland streams	This is a great product and clearly reflects a lot of expertise, time and effort. Thanks to you and your team for that! And thanks for inviting my opinions. I would love to hear back from you how my comments are received.	My second and more pressing concern is related - the absence of mention of how few free-flowing rivers (FFRs) actually remain in the world. See Grill et al. 2019 (https://www.nature.com/articles/s41586-019-1111-9). While the descriptions do not explicitly claim to reflect undisturbed systems, the lack of mention of the pervasive and ubiqutous impacts of dams and reservoirs suggests otherwise. I'm sure your team has thought of this, and I would love to hear what the thinking has been. I'm having a hard time supporting publication of this as is without weaving the ubiquity and impacts of dams/reservoirs throughout the freshwater EFGs. While the descriptions do not explicitly claim to reflect a dominance or norm of undisturbed systems, the lack of mention of the pervasive and ubiqutous impacts of dams and reservoirs suggests otherwise.	No change	Yes, the scope of the typology focussed on the ecological features that distinguish a group of ecosystems from others. In the profiles, there is limited scope for describing how different ecosystems have been affected by varied levels and types of human activities unless these are the major drivers that shape the identity of the system and maintain its features - some 15 anthropogenic ecosystems describe those cases. We will make this intent clearer in the background text. The effect of human influence and the consequences (e.g. few remaining free-flowing rivers will be addressed in global risk assessments as a basis for Red Lists of Ecosystems and national and global levels

Ecosystem Functional Group (EFG)	General comment	Specific comments	Response type	Detailed response
F1.1 Permanent upland streams	I think the freshwater EFGs don't require any other additions. The only one I would question is F2.2. I read through the text of F1 to F6, FT1.6 & 7, and F3.1 to 3.5. The text is general enough to capture the variety within any one EFG. I think some additions to the text on the invertebrate fauna of TF1.6 & TF1.7 is required. I have specific comments on the attached pdf.	1. What is upland?<br>2. Phytoplankton – is probably insignificant in upland streams, certainly in temperate regions<br>3. Might also mention spaty nature (has an influence on chemical conditions such as pH)<br>4. key recent reference is Riley et al. (2018): Riley, W. D., Potter, E. C. E., Biggs, J., Collins, A. L., Jarvie, H. P., Jones, J. I., Kelly-Quinn, M., Ormerod, S. J., Sear, D. A., Wilby, R. L., Broadmeadow, S., Brown, C. D., Chanin, P., Copp, G. H., Cowx, I. G., Grogan, A., Hornby, D. D., Huggett, D., Kelly, M. G., Naura, M., Newman, J. R. & Siriwardena, G. M. 2018. Small Water Bodies in Great Britain and Ireland: Ecosystem function, human-generated degradation, and options for restorative action. Sci Total Environ, 645, 1598-1616.	Minor edits to text	1. Redrafted poening sentence to define upland streams as follows: "These 1st-3rd order streams generally have steep gradients, fast flows, coarse substrates, often with a riffle-pool (shallow and fast vs deeper and slow) sequence of habitats, and periodic (usually seasonal) high-flow events."<br>2. Deleted phytoplankton<br>3. Define spaty<br>4. Thank yu for Riley et al. (2018) reference. We have given priority to global reviews

Ecosystem Functional Group (EFG)	General comment	Specific comments	Response type	Detailed response
F1.2 Permanent lowland rivers	Firstly, I commend this enterprise; it will undoubtedly be very useful in many ways. Congratulations to all for an amazing product.<br>My 'review' is quite minor, due to other time commitments. It comprises comments on so the sections named above, provided as Sticky Notes in the attached IUCN documents, and further comments in the second document attached.<br>The conceptual models are impressive and will no doubt be elaborated as users of this material develop more detailed descriptions of the categories of rivers and streams over time. I have added a few extra references to some of the categories assigned to me, and to the section on artesian springs.<br>I do feel that more types of waterbody may need to be added, such as dune lakes and ICOLLS.<br>Overall I found the full	I found the F1 categories to be sufficient to cover most stream and river types	No change	

Ecosystem Functional Group (EFG)	General comment	Specific comments	Response type	Detailed response
	suite of Ecosystem Functional Groups to be adequate at this level of resolution.<br>I found the F1 categories to be sufficient to cover most stream and river types. I have added comments on some of the entries (e.g. about energy sources, habitat structure, importance of floodplain inundation, and incidence of dystrophic waters and their low biodiversity but often high levels of endemicity). I have also suggested a few more references in some F1 ecosystem types.<br>The flow charts of processes are very good, but on the whole I found them to be rather similar across the F1 categories. I can see that it would be difficult to make them any more distinctive at this level of resolution.<br>I hope my brief contributions are useful to the project.			

Ecosystem Functional Group (EFG)	General comment	Specific comments	Response type	Detailed response
F1.2 Permanent lowland rivers	Firstly, I commend this enterprise; it will undoubtedly be very useful in many ways. Congratulations to all for an amazing product.<br>My 'review' is quite minor, due to other time commitments. It comprises comments on so the sections named above, provided as Sticky Notes in the attached IUCN documents, and further comments in the second document attached.<br>The conceptual models are impressive and will no doubt be elaborated as users of this material develop more detailed descriptions of the categories of rivers and streams over time. I have added a few extra references to some of the categories assigned to me, and to the section on artesian springs.<br>I do feel that more types of waterbody may need to be added, such as dune lakes and ICOLLS.<br>Overall I found the full	Do you mean floating leaved macrophytes? Some lowland rivers also support submerged macrophytes. Diversity may also be low in these dystrophic waterways.	Minor edits to text	Corrected text to "emergent, floating and submerged macrophyte vegetation". Added low diversity to last sentence on black waters

Ecosystem Functional Group (EFG)	General comment	Specific comments	Response type	Detailed response
	suite of Ecosystem Functional Groups to be adequate at this level of resolution.<br>I found the F1 categories to be sufficient to cover most stream and river types. I have added comments on some of the entries (e.g. about energy sources, habitat structure, importance of floodplain inundation, and incidence of dystrophic waters and their low biodiversity but often high levels of endemicity). I have also suggested a few more references in some F1 ecosystem types.<br>The flow charts of processes are very good, but on the whole I found them to be rather similar across the F1 categories. I can see that it would be difficult to make them any more distinctive at this level of resolution.<br>I hope my brief contributions are useful to the project.			

Ecosystem Functional Group (EFG)	General comment	Specific comments	Response type	Detailed response
F1.2 Permanent lowland rivers	I hope I have not gone over the top here in trying to provide a broader overview of the river and streams systems. Apologies if I have! The interaction with the surrounding landscape is fundamental to the ecosystem function and energy and nutrient dynamics. And the longitudinal changes help to provide a background to separate different lotic biomes.	Firstly, I think it would be worth considering separating F1.2 Permanent Lowland Rivers into two; i) permanent medium-sized lowland rivers, of stream order 4-6/7 (which are very different from upland (order 1-3) permanent streams) and ii) permanent large lowland rivers (stream order 8-10+). The later are totally different kind of aquatic realm (e.g. Columbia, Amazon, Yangtse, Pearl, Mississippi), often associated with large urban areas in developed world, large scale management and with a very different kind of fauna including large mammals, very large fish, large reptiles, extensive flood plains (and different from F1.5). The latter are a very different scale from the former with a different biology and functional rates etc.	Major revisions and reclassification	Split the unit into two Functional Groups on basis of stream order and flow volume as recommended. Redrafted text to reflect differences between these groups
F1.2 Permanent lowland rivers	let me say this is a really cool project, and you've clearly made a wonderful start. I'll look forward to the final product!	F1.2 Permanent lowland rivers: Surface water and groundwater can exchange and mix in the alluvium under and adjacent to the rivers, forming the hyporheic zone. This complex mix of different waters both plays an important role in nutrient cycling and supports a rich macroinvertebrate biota endemic to the hyporheic zone.	Minor edits to text	Added reference to hyporhoeic zone relating to macroinvertebrates and added the following text on ecological drivers: "Surface water and groundwater mix in the alluvium under and adjacent to the rivers, forming the hyporheic zone, which plays an important role in nutrient cycling"

Ecosystem Functional Group (EFG)	General comment	Specific comments	Response type	Detailed response
F1.2 Permanent lowland rivers		There are perennial lowland rivers in the NT and Kimberley. their uplands may be seasonal or episodic, but the lowlands are not. Please include these regions.	No change	Only a few Seasonal lowland rivers actually cease flowing in the dry season, however the flow volumes display high seasonal variability, and hence they are included in F1.5, not F1.2. In northern Australia, catchments of lowland streams (e.g. Fitzroy, Alligator, Jardine) are relatively small and climatically uniform relative to others elsewhere in the seasonal tropics, hence flows display strong seasonal signature
F1.2 Permanent lowland rivers	This is a great product and clearly reflects a lot of expertise, time and effort. Thanks to you and your team for that! And thanks for inviting my opinions. I would love to hear back from you how my comments are received.	My second and more pressing concern is related - the absence of mention of how few free-flowing rivers (FFRs) actually remain in the world. See Grill et al. 2019 (https://www.nature.com/articles/s41586-019-1111-9). While the descriptions do not explicitly claim to reflect undisturbed systems, the lack of mention of the pervasive and ubiqutous impacts of dams and reservoirs suggests otherwise. I'm sure your team has thought of this, and I would love to hear what the thinking has been. I'm having a hard time supporting publication of this as is without weaving the ubiquity and impacts of dams/reservoirs throughout the freshwater EFGs. While the descriptions do not explicitly claim to reflect a dominance or norm of undisturbed systems, the lack of mention of the pervasive and ubiqutous impacts of dams and reservoirs suggests otherwise.	No change	Yes, the scope of the typology focussed on the ecological features that distinguish a group of ecosystems from others. In the profiles, there is limited scope for describing how different ecosystems have been affected by varied levels and types of human activities unless these are the major drivers that shape the identity of the system and maintain its features - some 15 anthropogenic ecosystems describe those cases. We will make this intent clearer in the background text. The effect of human influence and the consequences (e.g. few remaining free-flowing rivers will be addressed in global risk assessments as a basis for Red Lists of Ecosystems and national and global levels

Ecosystem Functional Group (EFG)	General comment	Specific comments	Response type	Detailed response
F1.2 Permanent lowland rivers	I think the freshwater EFGs don't require any other additions. The only one I would question is F2.2. I read through the text of F1 to F6, FT1.6 & 7, and F3.1 to 3.5. The text is general enough to capture the variety within any one EFG. I think some additions to the text on the invertebrate fauna of TF1.6 & TF1.7 is required. I have specific comments on the attached pdf.	1. Define lowland<br>2. Qualify wide and deep<br>3. Lowland rivers in Ireland?	Minor edits to text	1. Added detail to the opening sentence to define lowland as follows: Small-medium lowland rivers "(stream orders 4-9) are productive depositional ecosystems"<br>2. Added deatil as follows: River channels are "tens to a few hundred metres wide and up to tens of metres deep"...<br>3. Yes lowland rivers occur in Ireland

Ecosystem Functional Group (EFG)	General comment	Specific comments	Response type	Detailed response
F1.3 Freeze-thaw rivers and streams	Firstly, I commend this enterprise; it will undoubtedly be very useful in many ways. Congratulations to all for an amazing product.<br>My 'review' is quite minor, due to other time commitments. It comprises comments on so the sections named above, provided as Sticky Notes in the attached IUCN documents, and further comments in the second document attached.<br>The conceptual models are impressive and will no doubt be elaborated as users of this material develop more detailed descriptions of the categories of rivers and streams over time. I have added a few extra references to some of the categories assigned to me, and to the section on artesian springs.<br>I do feel that more types of waterbody may need to be added, such as dune lakes and ICOLLS.<br>Overall I found the full	I found the F1 categories to be sufficient to cover most stream and river types	No change	

Ecosystem Functional Group (EFG)	General comment	Specific comments	Response type	Detailed response
	suite of Ecosystem Functional Groups to be adequate at this level of resolution.<br>I found the F1 categories to be sufficient to cover most stream and river types. I have added comments on some of the entries (e.g. about energy sources, habitat structure, importance of floodplain inundation, and incidence of dystrophic waters and their low biodiversity but often high levels of endemicity). I have also suggested a few more references in some F1 ecosystem types.<br>The flow charts of processes are very good, but on the whole I found them to be rather similar across the F1 categories. I can see that it would be difficult to make them any more distinctive at this level of resolution.<br>I hope my brief contributions are useful to the project.			

**Ecosystem Functional Group (EFG)**	**General comment**	**Specific comments**	**Response type**	**Detailed response**
F1.3 Freeze-thaw rivers and streams	I hope I have not gone over the top here in trying to provide a broader overview of the river and streams systems. Apologies if I have! The interaction with the surrounding landscape is fundamental to the ecosystem function and energy and nutrient dynamics. And the longitudinal changes help to provide a background to separate different lotic biomes.	1. Secondly for F1.3 Freeze-thaw rivers and streams, it is not clear whether you are trying to cover both small upland streams that freeze (e.g. in northern palearctic like scandinavia) and larger northern rivers that freeze over. These are very different I think and the text is somewhat confusing jumping from one to the other offering generalities that seem to cover both but certainly would not do so.. Not sure there needs to be two separate realms but certainly the text needs to differentiate these I feel.<br>2. Not completely clear whether you are trying to cover both smallish upland streams that freeze (e.g. in northern palearctic) and larger northern rivers that freeze over (e.g. in northern Siberia, China, Alps, Himalayas). These are quite different I think. The text is a bit confusing in places currently mixing small streams with large rivers.<br>3. Also I wondered about the inclusion of glacial streams/rivers which are unique in effectively providing pristine, empty lotic systems that are gradually colonised down-stream by a succession of insects then fish as the water temperature increases and surrounding landscape develops. In some ways they are like the hot spring fed streams which is constantly flowing and is gradually colonised down-stream this time as the temperature cools. I guess these are relatively minor types of system in the overall scheme of things.	Major revisions to text and diagram	Strengthened contrast between large and small freezethaw streams with several additions to text. Glacial melt streams are one of several variants of Freeze/thaw streams, hence not explicitly mentions. Adopted most additions but omitted least important points due to space constraints

Ecosystem Functional Group (EFG)	General comment	Specific comments	Response type	Detailed response
F1.3 Freeze-thaw rivers and streams	I think the freshwater EFGs don't require any other additions. The only one I would question is F2.2. I read through the text of F1 to F6, FT1.6 & 7, and F3.1 to 3.5. The text is general enough to capture the variety within any one EFG. I think some additions to the text on the invertebrate fauna of TF1.6 & TF1.7 is required. I have specific comments on the attached pdf.	Not generally a feature of the Irish landscape	Amendments to map	Inclusion of Ireland is due to limited resolution of first generation maps, which are tied to freshwater ecoregions. Ireland is in the same freshwater ecoregion as Scotland, where freeze-thaw lakes are common. Second generation maps will be higher resolution

Ecosystem Functional Group (EFG)	General comment	Specific comments	Response type	Detailed response
F1.4 Seasonal upland streams	Firstly, I commend this enterprise; it will undoubtedly be very useful in many ways. Congratulations to all for an amazing product.<br>My 'review' is quite minor, due to other time commitments. It comprises comments on so the sections named above, provided as Sticky Notes in the attached IUCN documents, and further comments in the second document attached.<br>The conceptual models are impressive and will no doubt be elaborated as users of this material develop more detailed descriptions of the categories of rivers and streams over time. I have added a few extra references to some of the categories assigned to me, and to the section on artesian springs.<br>I do feel that more types of waterbody may need to be added, such as dune lakes and ICOLLS.<br>Overall I found the full	I found the F1 categories to be sufficient to cover most stream and river types. An important reference: Ecology, 96(3), 2015, pp. 684–692 2015 by the Ecological Society of America Does flood rhythm drive ecosystem responses in tropical riverscapes? TIMOTHY D. JARDINE,1,2,3,10 NICHOLAS R. BOND,2 MICHELE A. BURFORD,2 MARK J. KENNARD,1,2 DOUGLAS P. WARD,1,2 PETER BAYLISS,1,4 PETER M. DAVIES,1,5 MICHAEL M. DOUGLAS,1,6 STEPHEN K. HAMILTON,7 JOHN M. MELACK,8 ROBERT J. NAIMAN,5,9 NEIL E. PETTIT,1,5 BRADLEY J. PUSEY,1,5 DANIELLE M. WARFE,5 AND STUART E. BUNN1,2	Added reference	Added Jardine et al.

Ecosystem Functional Group (EFG)	General comment	Specific comments	Response type	Detailed response
	suite of Ecosystem Functional Groups to be adequate at this level of resolution.<br>I found the F1 categories to be sufficient to cover most stream and river types. I have added comments on some of the entries (e.g. about energy sources, habitat structure, importance of floodplain inundation, and incidence of dystrophic waters and their low biodiversity but often high levels of endemicity). I have also suggested a few more references in some F1 ecosystem types.<br>The flow charts of processes are very good, but on the whole I found them to be rather similar across the F1 categories. I can see that it would be difficult to make them any more distinctive at this level of resolution.<br>I hope my brief contributions are useful to the project.			

Ecosystem Functional Group (EFG)	General comment	Specific comments	Response type	Detailed response
F1.4 Seasonal upland streams		Suggested change 'streams' to 'rivers'. Mention shallow channel depth. Add burrowing and omnivory as example traits. Queried non-migratory biota and suggested mobility as charactersitic trait. Add reference to isolated pools in dry season and coarse substrates in channels. In diagram, add seasonal drying to Abiotic environment, hebivory to biotic interactions, light to resurces, and replace algal and invertebrate diets with ominvory and add resilience in Traits.	Multiple edits to text and diagram	Made additions as recommended, but retained 'stream' in name to indicate lower stream order than lowland 'rivers'. Added reference to mobility but reference to non-migratory biota to highlight contrast with lowland rivers
F1.4 Seasonal upland streams	There is one section that is in a real muddle and needs to be completely rewritten – it is labelled differently in two places as: F1.4 Seasonal upland streams or F1.4 Monsoonal upland rivers. These are two quite different things: one is found all over the world, the other is restricted to monsoonal regions! Either, the current text can be retained and labelled F1.4 Monsoonal upland rivers. But, then a new section F1.x for Seasonal Upland Streams needs to be written. Or, rewrite the section completely and redraw the maps and diagrams so	1. ? news to me! They have algal and allochthonous carbon just like their perennial counterparts, and may have more species...I do not know of any evidence that this is the case. delete or prove it.<br>2. given that this is the most common type of lotic system in the world, it makes no sense to specify tropical or savanna systems. mediterranean systems, temperate etc, all have riparian forests and allochthonous inputs.<br>3. given that this peak is in summer in some continents and autumn in others, better to avoid. Plus the peak occurs in non-deciduous forests too...<br>4. studies vary tremendously on this. In some cases, the diversity of seasonal streams is a subset of that in nearby perennial streams, but in other places they are not. They may be less diverse, but not always. Given that the typology is so general,best to avoid sweeping	Major revision of text and map	The confusion was due to a revision that had only partly been implemented in the previous version. Rather than separate monsoonal from other upland seasonally intermittent stream types, we opted to generalise across all seasonal stream types and distinguish them from non-seasonal intermittent streams, which have a much more variable and lower frequency of flow.<br>1. Both seasonal (F1.4) and permanent (F1.1) upland streams have simpler trophic structure than lowland streams, which generally support more levels and greater diversity of predators (large fish, mammals, birds). The low trophic complexity of lowland streams (permanent and seasonal) and high trophic complexity of lowland streams (permanent and seasonal) is noted in text and diagrams of respective profiles. The text contains no statement about the relative complexity of trophic networks for permanent and seasonal upland streams, but to make the comparison clear, we amended text to "...and a simpler trophic structure than lowland rivers."<br>2. Deleted "savannas and tropical dry forests in "<br>3. Text contains no reference to timing of peak input via leaf fall. Changed "...which often includes an annual deciduous component" to "which may include an annual deciduous component"<br>4. Rephrased to acknowledged variation as follows: "Taxonomic

Ecosystem Functional Group (EFG)	General comment	Specific comments	Response type	Detailed response
	that it covers seasonal upland streams properly (as well as monsoonal ones). The current text is inadequate, and you will be criticised extensively by the many experts who work on these systems. Indeed, given that there have been many papers in the last few years emphasizing how this river type is neglected, I foresee papers in journals like Conservation Letters pointing out that the IUCN has fallen into this trap! IRES are the most common type of freshwater body globally and occur everywhere except permanently frozen and permanently dry regions of the globe. An enormous amount of work has been done on these in the last decade, they are termed IRES (intermittent rivers and ephemeral streams) and there is a recent book that summarises this research: Intermittent Rivers & Ephemeral streams: Ecology & Management.	generalisations that just given more ammunition to people who dont regard seasonal rivers as "real" rivers.<br>5. I dont think these are generally true. One of the largest lotic crayfishes Euastacus bispinosus can live in seasonal streams. Many migratory fish breed in seasonal streams in spring and then move downstream as flows decline. I think the authors are imposing traits from a few spp onto the rest, and these are not representative. In truth, these systems are found almost everywhere and are so diverse in terms of fauna (because the evolutionary histories are so diverse) that it does not make sense to generalise in this way.<br>6. This just isnt true. There are as many invertebrate carnivores as there are in nearby perennial streams, ditto fish. I dont know where this info is coming from.<br>7. this diagram is not suitable for non-monsoonal IRES<br>8. this contradicts the categorization!<br>9. maybe in the tropics, but this type occurs in temperate, mediterranean and arid climates outside the tropics. instead, it would be better to emphasize the importance of perennial refuges within IRES networks.<br>10. This map is ridiculous! Please see the map in the book reference provided. These streams occur everywhere except the most arid and most frozen places on Earth. I recommend deleting this whole		diversity varies between streams, but can be lower than permanent streams and relatively high in endemism."<br>5. Amended mention of small body sizes qualify as follows: ...small body sizes (especially in "resident" fish). Deleted "Few of the biota a migratory" and added phrase to "...high dispersal ability" as follows: "..., including seasonal migration"<br>6. The text was not comparing seasonal to permanent upland streams, it was comparing upland streams to lowland rivers (see point 5). To clarify, added "Compared to lowland rivers, the trophic structure has …" to beginning of sentence.<br>7. Replaced "monsoonal climate" with "Seasonal precipitation or snowmelt"<br>8. Qualified use of the term "intermittent" with following text: "They may be perennial or intermittent, with flows decreasing much-reduced or ceasing in the dry season, or seasonally intermittent with flows ceasing and water persisting in isolated stagnant pools. "<br>9. Turbulence and turbidity is not unique to tropical seasonal uplands streams, but is a function of gradients, substrates and peak flow volumes. No change made.<br>10. The earlier version of the map showed only the tropical and subtropical areas of distribution based on an earlier draft of the typology. We have updated it to include the temperate component of the distribution. Datry et al. (2017) does not include a comprehensive map of seasonal upland streams, but its maps of sampled locations have been incorporated.<br>11. Replaced Davies et al. (2008) with Datry et al. (2017)

Ecosystem Functional Group (EFG)	General comment	Specific comments	Response type	Detailed response
	(2017) Eds: Bonada, Boulton, Datry. Academic Press, Burlington. The book has chapters written by experts and I strongly suggest that you use this book as a reference and ask the editors to write the new section for you and that you call them F1.x Intermittent streams and ephemeral wetlands, to match what researchers are calling them.	map and using one from the book which is based on actual research.<br>11. These are just two random examples. why use these when we have a book that summarises global research on IRES in far more detail:Intermittent Rivers & Ephemeral streams: ecology & management. (2017) Eds: Bonada, Boulton, Datry. Academic Press, Burlington.		
F1.4 Seasonal upland streams	I think the freshwater EFGs don't require any other additions. The only one I would question is F2.2. I read through the text of F1 to F6, FT1.6 & 7, and F3.1 to 3.5. The text is general enough to capture the variety within any one EFG. I think some additions to the text on the invertebrate fauna of TF1.6 & TF1.7 is required. I have specific comments on the attached pdf.	1. Lower case on carbon<br>2. are you excluding seasonal upland streams in temperate and mediterranean areas?	Major revision and reclassification	1. Changed carbon to lower case<br>2. Profile now includes seasonal upland strems in temperate and Mediterranean-type climates

Ecosystem Functional Group (EFG)	General comment	Specific comments	Response type	Detailed response
F1.5 Seasonal lowland rivers	Firstly, I commend this enterprise; it will undoubtedly be very useful in many ways. Congratulations to all for an amazing product.<br>My 'review' is quite minor, due to other time commitments. It comprises comments on so the sections named above, provided as Sticky Notes in the attached IUCN documents, and further comments in the second document attached.<br>The conceptual models are impressive and will no doubt be elaborated as users of this material develop more detailed descriptions of the categories of rivers and streams over time. I have added a few extra references to some of the categories assigned to me, and to the section on artesian springs.<br>I do feel that more types of waterbody may need to be added, such as dune lakes and ICOLLS.<br>Overall I found the full	I found the F1 categories to be sufficient to cover most stream and river types. Needs a reference on the dependence of many fish species on inundated floodplains as habitat/food resources for larval, juvenile and mature fish. Production of fish biomass is related to area of floodplain inundated and length of time inundated.e.g.<br><br>Arthington, A. H. and Balcombe, S. 2011. Extreme flow variability and the 'boom and bust' ecology of fish in arid-zone floodplain rivers: a case history with implications for environmental flows, conservation and management. Ecohydrology 4: 706-720.	Added reference	Added the suggested reference

Ecosystem Functional Group (EFG)	General comment	Specific comments	Response type	Detailed response
	suite of Ecosystem Functional Groups to be adequate at this level of resolution.<br>I found the F1 categories to be sufficient to cover most stream and river types. I have added comments on some of the entries (e.g. about energy sources, habitat structure, importance of floodplain inundation, and incidence of dystrophic waters and their low biodiversity but often high levels of endemicity). I have also suggested a few more references in some F1 ecosystem types.<br>The flow charts of processes are very good, but on the whole I found them to be rather similar across the F1 categories. I can see that it would be difficult to make them any more distinctive at this level of resolution.<br>I hope my brief contributions are useful to the project.			

Ecosystem Functional Group (EFG)	General comment	Specific comments	Response type	Detailed response
F1.5 Seasonal lowland rivers	Another section in a similar muddle and labelled differently in two places is: F1.5 Seasonal lowland rivers, F1.5 Monsoonal Lowland rivers. Again, these are two quite different things, although as above, one could be nested in the other. In addressing issue 1, above, I think you can solve this one too by renaming the current section as Monsoonal Lowland Rivers and adding a new section called IRES (Intermittent Rivers and ephemeral streams) to cover that much more common biome. The dynamics of IRES are very different to monsoonal systems. I have put comments on the pdf and deleted the text that refers only to monsoonal rivers, to show you how much of what is there is incorrect for seasonal lowland rivers, which fit into IRES.	1. text reads as monsoonal lowland rivers not seasonal. that is, the text is not correct if the intent is to apply it to all non-perennial lowland rivers (a with the previous category). See major comments on review. Below I have deleted text that would be incorrect for lowland rivers generally, however, I think you should keep this as a monsoonal lowland rivers category and create a new one for IRES.<br>2. Replace "seasonal flooding dynamics" with "seasonal hydrology"<br>3. Delete "Although trophic networks are complex due to the diversity of consumers, large mobile predators such as otters, waterbirds, sharks, dolphins, and crocodilians are omnivorous, so food chains are short."<br>4. Replace "during the wet season" with "(if present) while rivers are flowing"<br>5. Delete "crocodilian"<br>6. Replace "wet season flows" with "hydrology"<br>7. this diagram is not suitable for non-monsoonal IRES<br>8. Replace "wet season flows" with "hydrology"<br>9. Delete "High-volume flows and floods occur during the summer wet season, coinciding with warm temperatures. These are followed by a decline of flows and reduced flood residence times during the transition to the dry season followed by low and disconnected flows during the dry season."	Major revision of text and map	1. The confusion was due to a revision that had only partly been implemented in the previous version. Rather than separate monsoonal from temperate lowland seasonally intermittent river types, we opted to generalise across all lowland rivers with a strong seasonal flow variation signature. Unlike upland seasonal streams, flows of lowland rivers may not completely cease each year, but there is nonetheless large and regular variation in flows, distinguishing them from non-seasonal intermittent streams, which have a much more variable and lower frequency of flow.<br>2. Replaced "seasonal flooding dynamics" with "seasonal hydrology"<br>3. It is true that not all of thes predators are present, but (with the exception of crocodilians) they are examples of large omnivorous predators that may occur throughout the latitudinal range of lowland seasonal rivers. Sentence was modified to note this exception and incorporate suggestions of other reviews as follows: "Although trophic networks are complex due to the diversity of food sources and the extent of omnivory amongst consumers, food chains tend to be short and large mobile predators such as otters, large piscivorous waterbirds, sharks, dolphins, and (in tropical rivers) crocodilians can have a major impact on the food webs."<br>4. Replaced "during the wet season" with "when they are connected by flows"<br>5. Replaced "crocodilian" with "other large aquatic "<br>6. Replaced "wet season flows" with "seasonal hydrology"<br>7. Replaced "monsoonal climate" with "Seasonal precipitation or snowmelt"<br>8. Rephrased sentence as follows: "Life cycle processes including reproduction, recruitment, and dispersal in most biota are tightly cued to seasonally high flow periods, often with floodplain nursery areas for river fish, amphibians and larger invertebrates."<br>9. Rephrased sentence to generalise as follow: "High-volume flows and floods occur during summer in the tropics or winter-

**Ecosystem Functional Group (EFG)**	**General comment**	**Specific comments**	**Response type**	**Detailed response**
		10. Add "solute concentrations" to seasonally variying features<br>11. Delete "The strength and inter-annual variability of this pattern changes depending on the seasonality of catchment rainfall and the influences of non-monsoonal sources of water such as snow melt (e.g. in South Asia)."<br>12. "single and multi-channelled" Delete "and"<br>13. Delete "There may be two flood peaks during the year in catchment ocated close to the equatorial zone."<br>14. Distribution - delete incorrect text<br>15. Delete incorrect references		spring at temperate latitudes."<br>10. Added "solute concentrations" to seasonally varying features<br>11. Rephrased as follows: "The strength and inter-annual variability of this pattern depends on the seasonality of catchment rainfall and sources of water that offset or mute the influences of rainfall seasonality (e.g. snow melt in South Asia)."<br>12. Deleted "and"<br>13. Deleted "There may be two flood peaks during the year in catchments located close to the equatorial zone," but mentioned "two [flow] peaks in some areas" in sentence about seasonal flow patterns<br>14. Distribution - replaced previous text with: "Tropical, subtropical and temperate lowlands with seasonal inflow patterns worldwide." Amended map to include temperate components of distribution<br>15. Replaced Layman et al. (2005) with Datry et al. (2017)
F1.5 Seasonal lowland rivers		Add range of stream orders, note interactions with floodplains and role as nurseries. Note omnivory and emphasise role of large predators. Suggested corresponding additions to diagrams	Multiple edits to text and diagram	Added range of stream orders, noted additional large predators and their impact on food chains. Added mention of floodplain nurseries. Made corresponding additions to diagram. Retained scope on arid streams since long periods of no flow and short duration flows are key characteristics. Did not add reference to upwelling/downwelling because streams are almost universally shallow. Adopted all other suggested additions to text and diagram.

Ecosystem Functional Group (EFG)	General comment	Specific comments	Response type	Detailed response
F1.6 Episodic arid rivers	Firstly, I commend this enterprise; it will undoubtedly be very useful in many ways. Congratulations to all for an amazing product.<br>My 'review' is quite minor, due to other time commitments. It comprises comments on so the sections named above, provided as Sticky Notes in the attached IUCN documents, and further comments in the second document attached.<br>The conceptual models are impressive and will no doubt be elaborated as users of this material develop more detailed descriptions of the categories of rivers and streams over time. I have added a few extra references to some of the categories assigned to me, and to the section on artesian springs.<br>I do feel that more types of waterbody may need to be added, such as dune lakes and ICOLLS.<br>Overall I found the full	I found the F1 categories to be sufficient to cover most stream and river types. Add "Most fish species use inundated floodplains in larval, juvenile and mature life stages, and produce massive biomass after large floods."	Minor change to text	Added "Most fish species use inundated floodplains in larval, juvenile and mature life stages, and produce massive biomass after large floods." to end of traits text

Ecosystem Functional Group (EFG)	General comment	Specific comments	Response type	Detailed response
	suite of Ecosystem Functional Groups to be adequate at this level of resolution.<br>I found the F1 categories to be sufficient to cover most stream and river types. I have added comments on some of the entries (e.g. about energy sources, habitat structure, importance of floodplain inundation, and incidence of dystrophic waters and their low biodiversity but often high levels of endemicity). I have also suggested a few more references in some F1 ecosystem types.<br>The flow charts of processes are very good, but on the whole I found them to be rather similar across the F1 categories. I can see that it would be difficult to make them any more distinctive at this level of resolution.<br>I hope my brief contributions are useful to the project.			

**Ecosystem Functional Group (EFG)**	**General comment**	**Specific comments**	**Response type**	**Detailed response**
F1.6 Episodic arid rivers		Queried whether scope should be broaded beyond arid to encompass all intermittent streams. Suggested minor additions for algae, influence of riparian vegetation, top-down regulation, insects and crustacea, qualification for abundant zooplankton in slow flows. Mention complex upwelling/downwelling in interittent streams. Amend diagram to include herbivory and mention long dry periods	Multiple edits to text and diagram	Added text in riparian vegetation, top-down regulation, zooplankton in slow reaches and amphibians.

Ecosystem Functional Group (EFG)	General comment	Specific comments	Response type	Detailed response
F1.6 Episodic arid rivers		1. muddled here again. this section is about the arid zone rivers which are distinct from intermittent streams in general.<br>2. Clear hotspots of biodiversity and ecological activity occur around perennial waterbodies that act as both evolutionary and ecological refuges throughout arid zones. There are a variety of types of these associated with rivers, including discharge and outcrop springs, subterranean aquifers, relict streams and riverine waterholes<br>3. I think the text here should reflect the defintion of taxa as either movers, networkers or permanent refuge dwellers, after Sheldon et al. (2010) Ecological roles and threats to aquatic refugis in arid landscapes: dryland river waterholes. Marine & Freshwater Research, 61, 885-895.<br>4. I think the typology of arid zone waterbodies by Davis et al. (2013) should also be cited here: Davis et al. (2013) Evolutionary refugia and ecological refuges: key concents for conserving Australian arid zone freshwater biodiversity under climate change. Global Change Biology, 19, 1970-1984.	Multiple edits to text	1. Replaced "intermittent" with "episodic"<br>2. Added "Episodic rivers are hotspots of biodiversity and ecological activity in arid landscapes, acting as both evolutionary and ecological refuges"<br>3. Rephrased text to emphasise this point as follows: "During dry periods, many organisms survive as dormant life phases (e.g. eggs or seeds), by reducing metabolism, or by persisting in perennial refugia (e.g. waterholes, shallow aquifers). They may rapidly recolonise the channel network during flow (networkers). Waterbirds survive dry phases by moving elsewhere, returning to breed during flows."<br>4. Added Sheldon et al. (2010) in preference to Davis et al. (2013) due to key subject matter and limited space

Ecosystem Functional Group (EFG)	General comment	Specific comments	Response type	Detailed response
F1.7 Large lowland rivers	I have looked through the two new profiles and I think you have done a good job on separating the large rivers into two. I have made some suggested track changes on both.	Firstly, I think it would be worth considering separating F1.2 Permanent Lowland Rivers into two; i) permanent medium-sized lowland rivers, of stream order 4-6/7 (which are very different from upland (order 1-3) permanent streams) and ii) permanent large lowland rivers (stream order 8-10+). The later are totally different kind of aquatic realm (e.g. Columbia, Amazon, Yangtse, Pearl, Mississippi), often associated with large urban areas in developed world, large scale management and with a very different kind of fauna including large mammals, very large fish, large reptiles, extensive flood plains (and different from F1.5). The latter are a very different scale from the former with a different biology and functional rates etc.	Major revisions to text and diagram	Added detail on diversity of pelagic fauna, small influence of riparian vegetation. Did not add suggested sentence on human influences, consistent with scope and anthropogenic. profiles. Examples of flow volume and depth added. Minor amendments to diagram.

Ecosystem Functional Group (EFG)	General comment	Specific comments	Response type	Detailed response
F1.7 Large lowland rivers	Firstly, I commend this enterprise; it will undoubtedly be very useful in many ways. Congratulations to all for an amazing product.<br>My 'review' is quite minor, due to other time commitments. It comprises comments on so the sections named above, provided as Sticky Notes in the attached IUCN documents, and further comments in the second document attached.<br>The conceptual models are impressive and will no doubt be elaborated as users of this material develop more detailed descriptions of the categories of rivers and streams over time. I have added a few extra references to some of the categories assigned to me, and to the section on artesian springs.<br>I do feel that more types of waterbody may need to be added, such as dune lakes and ICOLLS.<br>Overall I found the full	I found the F1 categories to be sufficient to cover most stream and river types	No change	

Ecosystem Functional Group (EFG)	General comment	Specific comments	Response type	Detailed response
	suite of Ecosystem Functional Groups to be adequate at this level of resolution.<br>I found the F1 categories to be sufficient to cover most stream and river types. I have added comments on some of the entries (e.g. about energy sources, habitat structure, importance of floodplain inundation, and incidence of dystrophic waters and their low biodiversity but often high levels of endemicity). I have also suggested a few more references in some F1 ecosystem types.<br>The flow charts of processes are very good, but on the whole I found them to be rather similar across the F1 categories. I can see that it would be difficult to make them any more distinctive at this level of resolution.<br>I hope my brief contributions are useful to the project.			

Ecosystem Functional Group (EFG)	General comment	Specific comments	Response type	Detailed response
F2.1 Large permanent freshwater lakes	Please see attached some comments on the sections asked for. I added brief comment also on ephemeral salt lakes. I have tried to focus on the ecological function, and applaud you for well written summaries -not an easy task. Some sections attracted no need on my mind for comment, others little more. I added a couple of reprints that may help-but no expectation that the references would be used.	1. "Marked niche differentiation in life history and behavioural feeding traits characterises the most diverse assemblages of macroinvertebrates and fish (e.g. ~500 cichlid fish species in Lake Victoria). Given the extreme anthropogenic impact on ecology and functional groups in Lake Victoria I do not think this is the best example to use here. Lake Malawi might be a better example-large intact fish fauna, low effect (relatively) from introductions, important effect of specialisations (see below).<br>2: Suggest it worth mentioning that the ancient lakes (e.g Afrcian Rift Valley, Baikal, Titicaca) have more diversity because of evolutionary history, and hence more specialisation affecting functional groupings.<br>3. Seems very fish focussed re EFG on fish. I suggest adding the importance of other groups. Example would be the diversity of shrimp in Lake Tanganyika, and how the more abundant open-water species affect the trophic dynamics, and potential effect on that of over-fishing.<br>4. In L. Malawi, high turnover of phytoplankton, maintains high primary productivity. Microbial loop at (especially at high temperatures) important for mineralization.<br>5. In large lakes lakes, seasonal cycles affect EFG and phytoplankton and zooplankton succession<br>6. Also consider more focus on the lower	Multiple edits to text and diagram	1. We checked estimates of cichlid richness in Lake Victoria (c500), Lake Tanganika (c.250 spp) and Lake Malawi (450-800 spp). Lake Victoria seems representative despite human influence on introductions and water quality.<br>2: There are some excellent recent papers on high speciation rates in these lakes. We added the following text to provide a little more information on the mechanisms: ...high catchment-level endemism, "in part due to long histories of environmental variability in isolation." and further: Marked niche differentiation in life history and behavioural feeding "and reproductive traits enables sympatric speciation and" characterises the most diverse assemblages ...<br>3. Fish are mentioned as examples of high diversity and for their roles in top-down regulation. Several other major groups of biota are mentioned, including crustaceans, which are also diverse but do not drive top-down regulation. Limited space precludes addition of further detail.<br>4. Added following phrase: Zooplankton, invertebrate consumers, and herbivorous fish "sustain high planktonic turnover " and support.... Also added following sentence: "This bottom-up web is coupled to a microbial loop, which returns dissolved organic matter to the web (rapidly in warm temperatures) via heterotrophic bacteria."<br>5. Added phrase as follows: High primary productivity may vary seasonally, "driving succession," depending on climate, light availability...<br>6. Added, "This bottom-up web..." in addition to existing mention of top-down regulation. In diagram, added "bottom-up" to arrow from Biological interactions box<br>7. Rephrased sentence to acknowledge importance of meromictic large lakes in the tropics as follows: Mixing may be monomictic (i.e. annual) or meromictic (i.e. seldom), "especially in the tropics, " ...

Ecosystem Functional Group (EFG)	General comment	Specific comments	Response type	Detailed response
		trophic levels and the top-down, bottom-up effects.<br>7. "Mixing may be monomictic (i.e. annual) or meromictic (i.e. rare) depending on inflow, depth, wind regimes, and seasonal temperature variation." Meromictic-important for EFG in large tropical lakes. Maybe globally rare, but extremely relevant for those lakes		
F2.2 Small permanent freshwater lakes	Overall this is an impressive synthesis and I thinks it be very valuable. There are of course site some simplifications/generalizations, but that is inevitable in this type of work.	"most diverse lakes" not correct terminology. The lake is not diverse per se	Minor edits to text	Rephrased to clarify context of diversity, ."Although less diverse than larger lakes, these lakes may support..."
F2.2 Small permanent freshwater lakes	I am also not entirely satisfied by the categories of large and small permanent lakes because these do no match the usual categories used by wetland ecologists (which are based on function). The usual (functional) categories are deep lakes and shallow lakes. Deep lakes have a hypolimnion – a zone deep enough to be permanently dark. This means that primary production only occurs in	1. Add to productivity sentence, ", but ecosystem function varies greatly depending on whether a hypolimnion exists and stratification occurs. In shallow lakes, light reaches the bed and the entire volume is available for primary production, which can establish a scenario of competition between phytoplankton and benthic macrophytes. Macrophyte-dominated lakes support higher biodiversity than phytoplankton-dominated lakes."<br>2. these differences and the consequences for deep vs shallow lakes for eutrophication have been central to lake ecology for decades, and should be	Multiple edits to text and diagram	General: Agree the distinction of depth is very important. A number of classifications (e.g. RAMSAR) follow size, recognising that large lakes have many distinctive features. Most lakes deep enough to extend beyond the photic zone are "large" ((F2.1) and/or freeze/thaw glacial (F2.4), but there are many important exceptions of smaller deep lakes. In order to maintain a manageable number of functional groups across the typology (Occam's Razor), this and variation within a number of other groups is managed by describing the key factors that influence variability in functionality. To emphasise the distinctions of depth further, we have enhanced detail about functional distinctions between small shallow and small deep lakes (see detail below).<br>1. Some of this is content is covered by existing text. To add further detail, we rephrased as follows: Shallow lakes tend to be more productive (by volume and area) than deep lakes "because

Ecosystem Functional Group (EFG)	General comment	Specific comments	Response type	Detailed response
	the littoral fringe and the upper water layers, the epilimnion or euphotic zone. In contrast, shallow lakes have the entire water volume and lake bed open for primary production. The two types of lake function entirely differently and require different management. The forces that form the two lake types also differ. Deep lakes are usually formed by vulcanism, plate tectonics and glaciation, whereas shallow lakes are formed by fluvial or coast-building (dune formation or coastal lagoon formation) processes. I urge you to consider changing the categories to Deep Perennial Lakes and Shallow Perennial Lakes or adding these categories and removing the small permanent lake category. The current text for small perennial lakes contains several poorly written sentences and some that do not say much, and overall does not really	included here. They affect ecosystem management and biodiversity.<br>3. research shows that some invertebrate species from perennial lakes retain the ability to withstand desiccation. Chester et al. (2013) Novel methods for managing freshwater refuges against climate change in southern Australia: anthropogenic refuges for freshwater biodiversity. NCCARF, Gold Coast.<br>4. again, many amphibious macrophytes live in permanent lakes<br>5. yes, and if you add the suggested text above, it becomes clear why this is the case (i.e. light).<br>6. and zooplankton are in turn consumed by fish, birds or invertebrate predators<br>7. This sentence isnt clear, because zoops are invertebrates and because zoops are the main consumers of phytoplankton. I have rewritten for clarity, Shallow lakes are some of the most biodiverse and productive aquatic ecosystems. They generally show high alpha and beta diversity, especially where they are macrophyte-dominated.<br>8. This sentence also doesnt make sense because it says detritvores vary rather than the number or diversity of detritivores vary. However, as they do this everywhere, it doesnt seem to be a very useful sentence. Instead, explaining basic dyanmics of small deep vs small shallow lakes is a better use of the word space available.		light penetrates the entire water column, establishing competition between benthic macrophytes and phytoplankton", with more complex trophic networks and stronger top-down regulation leading to alternative stable states and possible regime shifts between them. "Macrophyte-dominated lakes support higher biodiversity than phytoplankton-dominated lakes ."<br>2. Addressed by edit in response to pt 1<br>3. While interesting, this detail is less important to the core description and could not be included due to space constraints.<br>4. Benthis macrophytes (including amphibious) address in response to pt 1<br>5. Addressed by edit in response to pt 1<br>6. Added more general text as follow: ", while he main predators are macroinvertebrates, fish, amphibians and birds."<br>7. Addressed by edit in response to pt 6. High diversity of shallow macrophyte-dominated lakes is mentioned in a previous sentence, "Clear lakes in macrophyte-dominated states support higher biodiversity than phytoplankton-dominated eutrophic lakes."<br>8. Deleted sentence<br>9. Deleted sentence. Revised text (pt 1) now states, "Shallow lakes tend to be more productive (by volume and area) than deep lakes because light penetrates the entire water column, establishing competition between benthic macrophytes and phytoplankton, with more complex trophic networks and stronger top-down regulation leading to alternative stable states and possible regime shifts between them. "<br>10. deleted<br>11. Amended text to: ...critical breeding habitat for waterbirds, "amphibians and reptiles."<br>12. Added groundwater to text and diagram<br>13. Retained emphasis on catchment<br>14. Added text as follows: "Some lakes (e.g. on leached coastal sandplains or peaty landscapes) have dystrophic waters."<br>15. Added "or dimictic" to monmictic

Ecosystem Functional Group (EFG)	General comment	Specific comments	Response type	Detailed response
	represent the extensive knowledge base for this ecosystem type. I have added extensive notes on the pdf.	9. this [regime shift] is now stated more clearly above<br>10. this is odd and rather trivial - it is of very minor significance compared to other lake functions.<br>11. Add frogs and repltiles to critical habitat sentence<br>12. groundwater can play a very significant role here yet is not included in the diagram. This does not reflect the extensive knowledge base on shallow lakes very well.<br>13. Change "catchment rainfall amount and seasoanlity" to "annual rainfall and seasonality"<br>14. Small permanent lakes are often dystrophic, affecting ecosystem function and productivity.<br>15. dimictic much more common<br>16. Add salinity and colour to drivers<br>17. This [distribution] is not true either.		16. Added "salinity and tannins" to drivers<br>17. Assume concern is about occurrences in arid and semi-arid zones. There are some small permanent lakes in arid zones, but a large majority of lakes in those environments belong to other functional groups. Amended text to say "rarely" semi-arid or arid.

Ecosystem Functional Group (EFG)	General comment	Specific comments	Response type	Detailed response
F2.2 Small permanent freshwater lakes	Please see attached some comments on the sections asked for. I added brief comment also on ephemeral salt lakes. I have tried to focus on the ecological function, and applaud you for well written summaries -not an easy task. Some sections attracted no need on my mind for comment, others little more. I added a couple of reprints that may help-but no expectation that the references would be used.	1. Trophic structure and complexity depend on lake size, depth, location, and connectivity. Shallow lakes tend to be more productive (by volume and area) than deep lakes, with more complex trophic networks and stronger top-down regulation by predators." Suggest added point/emphasis of Importance of the littoral zone and benthic pathways in terms of overall production and trophic interactions<br>Vadeboncoeur et al (2002). Putting the Lake Back Together: Reintegrating Benthic Pathways into Lake Food Web Models: Lake ecologists tend to focus their research on pelagic energy pathways, but, from algae to fish, benthic organisms form an integral part of lake food webs BioScience, Volume 52, Issue 1, January 2002, Pages 44–54, https://doi.org/10.1641/0006-3568(2002)052[0044:PTLBTR]2.0.CO;2<br>2. "...may generate monomictic temperature stratification regimes in deeper lakes, while shallow lakes are polymicitic..." Maybe too nuanced [to mention], but even shallow lakes can have parodic and often short loved periods of multiple stratification.	Minor edits to text and map	1. Added the following text: "Littoral zones and benthic pathways are integral to overall production and trophic interactions."<br>2. Added following phrase:: shallow lakes are polymicitic, "sometimes with short periods of multiple stratification."

Ecosystem Functional Group (EFG)	General comment	Specific comments	Response type	Detailed response
F2.2 Small permanent freshwater lakes	I think the freshwater EFGs don't require any other additions. The only one I would question is F2.2. I read through the text of F1 to F6, FT1.6 & 7, and F3.1 to 3.5. The text is general enough to capture the variety within any one EFG. I think some additions to the text on the invertebrate fauna of TF1.6 & TF1.7 is required. I have specific comments on the attached pdf.	1. F2.2 it covers a wide range of sizes and perhaps does not adequately capture the diversity. Ian - this is perhaps more within your expertise - should pools be lumped in with lakes up to 100km2 in area?<br>2. be more specific. This group spans a wide range of geological/topographical settings and so will be hydrochemically variable.	Minor edits to text	1. We elaborated on the diversity of lakes within this group with the following text: These lakes may be hydrologically isolated, groundwater-dependent or connected to rivers as terminal or flow-through systems. ... Some lakes (e.g. on leached coastal sandplains or peaty landscapes) have dystrophic waters." The group includes a range of lake sizes up to 100km2. There is no clear distinction of lake sizes within this range, but many of the smallest lakes are not permanent (see F2.3, F2.5)<br>2. The variability of geological/topographical settings makes it difficult to be specific about hydrochemical properties. To illustrate the broad range of variation, we added the following text: "Some lakes (e.g. on leached coastal sandplains or peaty landscapes) have dystrophic waters."
F2.3 Seasonal freshwater lakes	let me say this is a really cool project, and you've clearly made a wonderful start. I'll look forward to the final product!	F2.3 Seasonal freshwater lakes: The description implies a lack of surface-water connectivity. While that is the more common condition on an annual basis, surface-water connectivity can be ephemeral or even seasonal and is an important way that landscapes connect. In some cases, like vernal pools in California, these become the headward extent of stream systems when the stream systems are at full expansion (e.g., wet time in the wet season)	Minor edits to text	Added "Most lakes are hydrologically isolated, but some become connected seasonally by sheet flows or to drainage lines. " to text

Ecosystem Functional Group (EFG)	General comment	Specific comments	Response type	Detailed response
F2.3 Seasonal freshwater lakes		1.Add "hatching and emergence of invertebrates,"<br>2. this infers that these environments always become anoxic which is not correct. Wind mixing oxygenates waters, and anoxia is not inevitable. It can happen, especially in eutrophic wetlands, but not usually in oligotrophic ones.<br>3. Wind-induced mixing oxygenates these wetlands, although eutrophic or unmixed waters may become anoxic due to high biolgical oxygen demand. Anoxia may only occur nocturnally because of high primary productivity; this will be accompanied by dominance by air-breathing animals.<br>4. When these wetlands dry out, many plants and animals take refuge in sediments as dormant or quiescent stages. Animals such as crayfish may retreat to burrows that extend to the water table. Other animals, such as turtles, may aestivate in sediments or fringing vegetation.<br>5. the presence of submerged and emergent macrophytes is the single greatest driver of animal biodiversity.<br>6. -many annual grasses and herbs live on dry lake beds, dying at or just before seasonal inundation.<br>7. gnammas are so different, i think they need a brief paragraph of their own. Gnammas form a distinct ecosystem type within this category because they are based on rock with very few sediments;	Multiple edits to text and map	1. Added " hatching and emergence of invertebrates,"<br>2. Deleted and replaced with text in pt 3<br>3. Added following text to replace that deleted in pt 2: "Wind-induced mixing oxygenates these wetlands, but eutrophic or unmixed waters may become anoxic and dominated by air-breathing invertebrates due to high biological oxygen demand as productivity and biomass reach a peak. Anoxia may be abated diurnally by photosynthetic activity."<br>4. Some existing text covered related issues, however we rephrased it as follows: "Resident plants and animals persist through seasonal drying in sediments or on lake margins as desiccation-resistant dormant or quiescent life stages, e.g. crayfish may retreat to burrows that extend to the water table, turtles may aestivate in sediments or fringing vegetation, amphibious, perennial plants may persist on lake margins or in seedbanks."<br>5. Added"especially submerged and emergent macrophytes"<br>6.Added, "Cyanobacteria, algae, and macrophytes are the major primary producers, while annual grasses may colonise dry lake beds."<br>7. Space constraints limit elaboration. Added "Rock pools have simple trophic structure, based primarily on epilithic algae or macrophytes, and invertebrates, but no fish."<br>8. Patchiness covered by existing reference to zonation<br>9. Added: ...seasonal rainfall, "surface flows, groundwater fluctuation" and …<br>10. Deleted ", mostly in monsoonal and Mediterranean climates with marked tropical winters or temperate summers, respectively"<br>11. Added text as follows: ...impede infiltration "in some lakes; in others groundwater percolates up through sand or peat".<br>12. Added following text to clarify: "Most lakes are hydrologically isolated, but some become connected seasonally by sheet flows or drainage lines. "<br>13. Qualifier "usually" accommodates GDEs, but phrase deleted

Ecosystem Functional Group (EFG)	General comment	Specific comments	Response type	Detailed response
		they are always fishless and in some regions contain high plant and invertebrate endemicity. Food webs are usually very simple and based on either epilithic algae and/or macrophyte growth. The shapes and locations of gnammas (in clusters) also influences their biodiversity.<br>8. patchiness in vegetation<br>9. - groundwater fluctuations or surface flows<br>10. Delete ", mostly in monsoonal and Mediterranean climates with marked tropical winters or temperate summers, respectively"<br>11. in some small lakes; in others, groundwater percolates upwards through sand and peat to fill wetlands.<br>12. again, this is not true of all wetlands. certainly not groundwater dependent wetlands.<br>13. "...usually in upper catchments or undulating lowlands" again, many are GDEs so this is not universally true.<br>14. There are gnammas across the whole greater western woodlands region in Southern WA so please increase the shaded area to reflect this. If red is some indication of importance, then SWWa should all be in red as the most biodiverse gnammas described so far in the world occur there. gnammas also occur in the Kimberley in Western Australia, please add this. Reference: Carey et al. (2019) Do active-dispersing insects dominate the invertebrate fauna of rockpools in the wet-		to make space for other edits<br>14. Amended map to include additional areas of seasonal lakes including rock pools. In the Kimberley, Arafura, the Caatinga and east Africa. Seasonal lakes do have important occurrences on inselbergs in the SW Aust wheatbelt (as well as the Darling Range and south coast). The wheatbelt pools could not be included in the current map due to constraints imposed by freshwater ecoregions<br>15. Replaced Keeley & Zedler (1998) with a tropical seasonal lake reference: Pettit N, Jardine T , Hamilton S , Sinnamon V, Valdez D, Davies P, Douglas M, Bunn S (2012) Seasonal changes in water quality and macrophytes and the impact of cattle on tropical floodplain waterholes. Marine and Freshwater Research 63, 788-800

Ecosystem Functional Group (EFG)	General comment	Specific comments	Response type	Detailed response
		dry tropics, Kimberley<br>15. So, this category includes groundwater-dependent wetlands, vernal pools and gnammas, but we have 2 references on vernal pools and totally ignore the others? Instead, how about using at least one more recent and comprehensive reference : Invertebrates of freshwater temporary ponds in mediterranean climates (2016). Eds Boix & Baxter, Springer International publishing, Switzerland.		
F2.3 Seasonal freshwater lakes	Please see attached some comments on the sections asked for. I added brief comment also on ephemeral salt lakes. I have tried to focus on the ecological function, and applaud you for well written summaries -not an easy task. Some sections attracted no need on my mind for comment, others little more. I added a couple of reprints that may help-but no expectation that the references would be used.	Missing turloughs, seasonally (winter flooded lakes associated with karst) common in Ireland but also occur elsewhere. Display highly seasonal succession patterns of algae and invertebrates, Usually low/absent fish numbers.	Minor edits to text and map	Added turloughs to Introductory sentence as follows: ...seasonal freshwater lakes, vernal pools, "turloughs", or gnammas..." Added the following phrase to Drivers section: groundwater percolates up through sand, peat or "fissures in karstic limestone (turloughs)." Added Ireland to map.

Ecosystem Functional Group (EFG)	General comment	Specific comments	Response type	Detailed response
F2.4 Freeze-thaw freshwater lakes	Overall this is an impressive synthesis and I thinks it be very valuable. There are of course site some simplifications/generalizations, but that is inevitable in this type of work.	1. "frozen for at least 40% of the year" this is certainly not correct for the zone illustrated in the map. Better state "... is normally frozen for a variable period of "<br>2. not only photoautotrophs, also chemoautotrophs play an important role. Better be inclusive<br>3. Diatoms are first ..." insert 'normally' first<br>4. cyanobacteria typically emerge later in summer when there is already high grazing pressure from zooplankton.<br>5. this is not really correct as the thermal gradients are normally stronger in summer (but nor protected by the ice cap) and the higher biological activity in summer would then create stronger gradients in oxygen, nutrients etc. n.<br>6. not sure you would see this in southern france and germany..excepot for at very high latitudes	Multiple edits to text	1. Agree that freezing regime is too restrictive, however more guidance is needed than "for a variable part of the year". Text replaced with phrase with a broader range of freezing regimes, acknowledging variability "The majority of surface of these lakes is frozen for at least a month in most years."<br>2. Added chemoautotrophs<br>3. Inserted "usually" first<br>4. Rephrased as follows: "followed by small and motile zooplankton, which respond to increased food availability, and cyanobacteria later in summer when grazing pressure is high."<br>5. Replaced "enhancing depth gradients" with subduing summer depth gradients<br>6. First generation indicative map is based on Freshwater ecoregions, which encompass all of western Europe, including some parts of ecoregions where freeze-thaw lakes do not occur. Second-generation maps are finer resolution and will exclude extraneaous areas

Ecosystem Functional Group (EFG)	General comment	Specific comments	Response type	Detailed response
F2.4 Freeze-thaw freshwater lakes	Where do small lakes of glacial origin (i.e. kettle lakes, tarms, moraine lakes) that are ice covered for 2-4 months of the year fit in this scheme? Such as occur in highland Tasmania and the Australian Alps? They don't fit in the freeze-thaw lakes as they aren't frozen for long enough and they aren't mentioned in small perennial lakes either. They differ substantially from the latter because they are often highly oligotrophic with very rocky sediments, are often highly coloured (dystrophic) and tend to be depauperate. Although in Tasmania they harbour ancient crustacean species (i.e. Anaspides). They should be represented somewhere, but the small permanent lake category is overstuffed as it is. I think it would be best to be a bit more flexible and include them in freeze thaw lakes, as this category seems to fit best.	1. These lakes are also often the origin of upland streams and rivers.<br>2. This is an extremely deep lake of tectonic origin and very different to moraine or kettle lakes. I think different lake origins and how they affect ecosystem function should be here somewhere.<br>3. with small areas of glacial terrain in Tasmania and New Zealand	Minor edits to text and map	General: Scope of F2.4 now broadened to include a wider range of freezing regimes. Tasmania now included in yellow<br>1. Added following text to Drivers section: "Many lakes are stream sources."<br>2. Added following text after opening sentence: "Their varied origins (tectonic, riverine, fluvioglacial), size and depth affect composition and function."<br>3. Added "New Zealand and Tasmania" to Distribution text and Tasmania to map

Ecosystem Functional Group (EFG)	General comment	Specific comments	Response type	Detailed response
	This means adding Tasmania and Australian alps to the map and including shorter freeze duration.			
F2.4 Freeze-thaw freshwater lakes	Please see attached some comments on the sections asked for. I added brief comment also on ephemeral salt lakes. I have tried to focus on the ecological function, and applaud you for well written summaries -not an easy task. Some sections attracted no need on my mind for comment, others little more. I added a couple of reprints that may help-but no expectation that the references would be used.	Description seems very good, but I do not have personal experience with this lake type	No change	

Ecosystem Functional Group (EFG)	General comment	Specific comments	Response type	Detailed response
F2.5 Ephemeral freshwater lakes	Please see attached some comments on the sections asked for. I added brief comment also on ephemeral salt lakes. I have tried to focus on the ecological function, and applaud you for well written summaries -not an easy task. Some sections attracted no need on my mind for comment, others little more. I added a couple of reprints that may help-but no expectation that the references would be used.	No comment-seems comprehensive	No change	
F2.5 Ephemeral freshwater lakes		these features are often saline and are part of the salt lake continuum	Minor edits to text	Existing text says: "...flocculation (i.e. clumping) from increasing salinity during drying reduce turbidity over time." Added phrase to more explicitly recognise continuum as follows: Bottom sediments release nutrients rapidly after filling "and solute concentrations increase as drying progresses, placing these systems on a continuum with salt lakes."

Ecosystem Functional Group (EFG)	General comment	Specific comments	Response type	Detailed response
F2.6 Permanent inland salt lakes		1. chemistry 101 - a SODA lake cannot be NaCl! Often sodium carbonate NaHCO3 or NaHCO3 + CO3. See either: Boulton et al (2014) Australian Freshwater Ecology: Processes & Management or Boros & Kolpakova (2018) A review of the defining chemical properties of soda lakes and pans: An assessment on a large geographic scale of Eurasian inland saline surface waters. Plos One.<br>2. and some have volcanic origin<br>3. sw vic ones studied by Williams	Minor edits to text	1. This group includes both salt (NACl in the strict sense) and soda (carbonate) lakes. For brevity, we used the more common expression 'salt lakes' in the functional group title, since Na2CO3, NaHCO3, KCl and other constituents are all inorganic salts. The reviewer's comments indicate that the text could be misinterpreted as meaning that salt and soda are synonymous. It appears from that PLos reference that many soda lakes (45.9%, the highest number) are dominated by NaCl. This, and biotic similarities, justifies inclusion of salt (sensu stricto) and soda lakes within a single functional group of ecosystems.<br>2. Added text as follows: A few are much larger and deeper (e.g. Caspian Sea), "while some volcanic lakes are small and deep."<br>3. Noted, but too local to include in global description except as mapped. Vic lakes already included, added those in south WA.
F2.6 Permanent inland salt lakes		1. Both F2.6 Permanent salt lakes and F2.7 Ephemeral Salt lakes have a Freshwater Realm. Is this correct?<br>2. In both cases, there may also be groundwater contributions both in terms of water balance and chemistry.	Minor edits to text	1. Correct. As explained in preamble, Freshwater encompasses non-marine saline ecosystems, following other international systems<br>2. Added text as follows: "Groundwater also contributes to water balance and chemistry."

Ecosystem Functional Group (EFG)	General comment	Specific comments	Response type	Detailed response
F2.6 Permanent inland salt lakes	Overall not bad, except that a distinction should be made between salt lakes and soda lakes, which are quite different. Salt lakes result from dried up sea basins and have a salt composition similar to that of seawater (Utah's Great Salt Lake is a good example). Soda lakes are highly variable ecologically because the composition of the salts dissolved in their waters depends on what leaches out of the lake's watershed. Importantly, they often have high pH (hence "soda lakes"), rarely low pH, and the dominant salt is not sodium chloride but rather sodium carbonate/sulfate, which profoundly affects their geochemistry. They are often quite productive and often have high phosphate concentrations. Depending on lake bathymetry and mixing depth, saline and soda lakes also undergo seasonal or multi-year	1. Overall not bad, except that a distinction should be made between salt lakes and soda lakes, which are quite different.<br>2. Salt lakes result from dried up sea basins and have a salt composition similar to that of seawater (Utah's Great Salt Lake is a good example). Soda lakes are highly variable ecologically because the composition of the salts dissolved in their waters depends on what leaches out of the lake's watershed. Importantly, they often have high pH (hence "soda lakes"), rarely low pH, and the dominant salt is not sodium chloride but rather sodium carbonate/sulfate, which profoundly affects their geochemistry. They are often quite productive and often have high phosphate concentrations.<br>3. Depending on lake bathymetry and mixing depth, saline and soda lakes also undergo seasonal or multi-year episodes of stratification driven by elevated freshwater inflow during high runoff years which, coupled with high productivity in the overlying water column, can lead to anoxia (which is not primarily a response to salinity per se). They are extremely sensitive to climate change and to human manipulation of watershed hydrology.<br>4. They extend north into Canada, and into Peru, Bolivia, Paraguay<br>Brazil. Spain, southern France. Many in eastern Europeassociated with evaporite	Multiple edits to text and map	1. Emphasised the distinction between salt and soda lakes, and noted their uniting features by: i) amending title to "Permanentn salt and soda lakes"; ii) replacing the opening sentences with text as follows: "These lakes are united by relatively constant, high inorganic solute concentrations (notably sodium ions), and an associated specialist biota."; and iii) addtional edits under point 2.<br>2. Added the following text to emphasise contrast between salt and soda lakes: "Salt lakes are mostly legacies of marine incursions (since retreated) and dominated by sodium chloride (cf. seawater). Soda lakes derive more diverse solutes from catchment leachates, with sodium carbonate or sulphate dominant." Existing text notes range of pH, sometime abundance of phosphorus and high productivity.<br>3. Noted periodicitty of meromixis by replacing text: "Within lakes, salt concentrations may be vertically stratified (i.e. meromictic) due to the higher density of saltwater compared to freshwater inflow and slow mixing" as follows: "Ionic concentrations may be vertically stratified (i.e. meromictic), oftenseasonally, due to slow mixing after periodic inflows episodes of low- density freshwater." Human impacts are beyond scope of the profiles and will be addressed in a separate project.<br>4. Edited map to include occurrences in Canada, Bolivia, Peru, Brazil, Paraguay, Spain and south France. Other locations already mapped include salt lakes.<br>5. Subglacial lakes in the Antarctic, and likely Greenland, are includes in separate EGF 2.10 Subglacial lakes due to unique light, and temperature environment and microbial biota

Ecosystem Functional Group (EFG)	General comment	Specific comments	Response type	Detailed response
	episodes of stratification driven by elevated freshwater inflow during high runoff years which, coupled with high productivity in the overlying water column, can lead to anoxia (which is not primarily a response to salinity per se). They are extremely sensitive to climate change and to human manipulation of watershed hydrology. They extend north into Canada, and into Peru, Bolivia, Paraguay Brazil. Spain, southern France. Many in eastern Europe associated with evaporite mineral deposits. I think they are also common in Western Australia and I am not aware of any in New Zealand, the Yucatan Peninsula or Jamaica or Puerto Rico. Many subglacial lakes in the Antarctic, and likely Greenland, are salt/soda lakes	mineral deposits. I think they are also common in Western Australia and I am not aware ofany in New Zealand, the Yucatan Peninsula or Jamaica or Puerto Rico.<br>5. Manysubglacial lakes in the Antarctic, and likely Greenland, are salt/soda lakes		

Ecosystem Functional Group (EFG)	General comment	Specific comments	Response type	Detailed response
F2.7 Ephemeral salt lakes	Please see attached some comments on the sections asked for. I added brief comment also on ephemeral salt lakes. I have tried to focus on the ecological function, and applaud you for well written summaries -not an easy task. Some sections attracted no need on my mind for comment, others little more. I added a couple of reprints that may help-but no expectation that the references would be used.	"The high biomass of archaeal and bacterial decomposers and phytoplankton in turn support abundant consumers including crustaceans (e.g. brine shrimps and copepods) insects and other invertebrates, fish, and specialist waterbirds (e.g. banded stilts..." Seasonably flooded pans can be essential breeding sites for water birds-most noted probably is the Magkadikgadi salt pan in Botswana and its large breeding population of flamingos, and the subsequent connectivity with small water bodies in the region after the pans dry out. McCulloch, G., Aebischer, A., & Irvine, K. (2003). Satellite tracking of flamingos in southern Africa: the importance of small wetlands for management and conservation. Oryx, 37(04), 480–483. https://doi.org/10.1017/S0030605303000851	Minor edits to text and refs	Added flamingos as example of specialist birds. Added reference
F2.7 Ephemeral salt lakes		1. Both F2.6 Permanent salt lakes and F2.7 Ephemeral Salt lakes have a Freshwater Realm. Is this correct?<br>2. In both cases, there may also be groundwater contributions both in terms of water balance and chemistry.	Minor edits to text	1. Correct. As explained in preamble, Freshwater encompasses non-marine saline ecosystems, following other international systems<br>2.Added "groundwater" to relevat sentences as follows: ...and the balance between freshwater inflows, "groundwater," precipitation, and evaporation.... and : Inflow "and groundwater" are critical to ecosystem dynamics...

Ecosystem Functional Group (EFG)	General comment	Specific comments	Response type	Detailed response
F2.7 Ephemeral salt lakes	Overall not bad, except that a distinction should be made between salt lakes and soda lakes, which are quite different. Salt lakes result from dried up sea basins and have a salt composition similar to that of seawater (Utah's Great Salt Lake is a good example). Soda lakes are highly variable ecologically because the composition of the salts dissolved in their waters depends on what leaches out of the lake's watershed. Importantly, they often have high pH (hence "soda lakes"), rarely low pH, and the dominant salt is not sodium chloride but rather sodium carbonate/sulfate, which profoundly affects their geochemistry. They are often quite productive and often have high phosphate concentrations. Depending on lake bathymetry and mixing depth, saline and soda lakes also undergo seasonal or multi-year	1. this class could also include solar salterns, which are common on the Mediterranean coasts, Mexico, California, Chile/Peru and probably Australia. Though depending on how the salterns are operated there will be semi-permanent hypersaline basins associated with them.<br>2. Endorheic systems can have non-marine salt composition (see above), including high concentrations of arsenic, selenium, boron. Salinity varies up to salt saturation, which varies depending on composition of the salt, but can be >40%.<br>3. The text here implies longevity of the features that is in conflict with the descriptor. And as above, they are more widely distributed than shown on the map.	Minor edits to text	1. Solar salterns are an anthropogenic ecosystem type currently included within functional group F3.2 constructed lacustrine wetlands. This group includes a very broad range of hydrochemistry including various industrial waste ponds and sewage ponds, as welll as farm dams. It may warrant subdivision in future<br>2. Added arsenic and boron to ionic composition. Increased range of salinity value frm "over 26%" to "up to 40% and added hydrochemistry to list of dependencies.<br>3. Ephemeral refers to wet-dry phases. Rephrased text to clrify as follows: "Salinity varies from 0.3% up to 40% through wet-dry phases depending on ..."

Ecosystem Functional Group (EFG)	General comment	Specific comments	Response type	Detailed response
	episodes of stratification driven by elevated freshwater inflow during high runoff years which, coupled with high productivity in the overlying water column, can lead to anoxia (which is not primarily a response to salinity per se). They are extremely sensitive to climate change and to human manipulation of watershed hydrology. They extend north into Canada, and into Peru, Bolivia, Paraguay Brazil. Spain, southern France. Many in eastern Europe associated with evaporite mineral deposits. I think they are also common in Western Australia and I am not aware of any in New Zealand, the Yucatan Peninsula or Jamaica or Puerto Rico. Many subglacial lakes in the Antarctic, and likely Greenland, are salt/soda lakes			

Ecosystem Functional Group (EFG)	General comment	Specific comments	Response type	Detailed response
F2.8 Artesian springs and oases		Insert "because they are fed by artesian waters that discharge to the surface through faults and other formations" in first sentence.	Minor edits to text and refs	Inserted suggested phrase
F2.9 Geothermal wetlands		To match the title, could the list be changed to "hot springs, geysers, mud pots and associated wetlands"?<br>Delete 'permanent'<br>add "deeply-circulating"?<br>Probably best delete 'igeous' to leave "hot rocks". It is true that most geothermal systems interact with igneous rocks at depth, but not exclusively.<br>General note. I think the key thing missing from the introductory sentences is that the substrates are chemically precipitated sediments/rocks and mineral soils. Silica sinter, travertine (calcium carbonate) clays (around mud pots).<br>I wonder if the filamentous could be lost here. There are unicellular chlorophyte algae in the same environments. Is it possible to use "mat-forming algae" here?<br>My hunch is that this passage is trying to describe the down temperature transition across the "photosynthetic fringe" where you get mats of cyanobacteria and eventually chlorophyte algae. You also see mat forming filamentous algae in alkaline springs though the groups present are generally different.<br>Add' and geothermal wetlands' to sinter aprons<br>Again, I think filamentous is too specific	Multiple edits to text	Added 'associated wetlands'<br>Deleted 'Permanent'<br>added "deeply-circulating"<br>Deleted igneous'<br>Added "that produce chemically precipitated substrates"<br>Replaced 'filamentous with 'mat-forming'<br>Added wetlands'<br>Deleted 'filamentous'<br>Added 'animal protists'<br>Deleted annelids<br>Covered by molluscs<br>Added amphibians, fish and snakes<br>Added visiting birds<br>Added snakes and fish to endemism<br>Changed to 'bedrock'<br>Emphasised silicon, calcium and iron, with other minerals and changed order as suggested<br>Added 'tectonically'<br>Deleted subduction zones due to limited space<br>Added Japan and east Africa

Ecosystem Functional Group (EFG)	General comment	Specific comments	Response type	Detailed response
		here. There are also other cell patterns/organizations present.<br>There are also animal-protists present often at high temperatures, low pH.<br>I really cannot recall literature relating to annelids in terrestrial geothermal settings.<br>I do know of thermal spring gastropods e.g. endemics in the genus Physella.<br>There are some notable vertebrate occurrences in thermal springs and their run-off streams. I have observed the toad Rhinella spinulosa at El Tatio, Chile.<br>There is an endemic<br>Himalayan thermal spring snake Thermophis baileyi. There are critical list fish as well e.g. The Julimes pupfish (Cyprinodon julimes) which lives in water up to 46C.<br>The thermal spring habitats are also important sources of water for birds. In the African Rift Lakes, the thermal springs that feed them are often less saline than the lakes that are evaporation prone. Flamingo use the thermal water. In Yellowstone the thermal wetlands are habitiat for migratory water birds (I have published on a Coot, Fulica americana, fossil from Yellowstone hot springs. I guess it could be argued that these are visitors<br>As do the snakes and fish just mentioned.<br>could this be changed to bed-rock? An example, subsurface waters of the Yellowstone geothermal system flow to the surface through igneous rocks alone in		

Ecosystem Functional Group (EFG)	General comment	Specific comments	Response type	Detailed response
		some areas (resulting in silica deposition as silica sinter). However, at Mammoth Hot Springs they flow through a thick sequence of sedimentary rocks and deposit travertine (calcium carbonate).<br>As they are the major elements in terms of "deposited rock types", at the front of the list I would have silicon (silica sinter); calcium (travertine depositing springs) and iron (at iron-oxide sinter depositing springs). Then go on to the othe elements. You may want to keep it as is, but you have lead in the list and lead and polonium are part of the same isotope decay chain so maybe should sit together?<br>Tectonically and volcanically active - covers all the settings I can think of. The Himalaya Geothermal Belt is not volcanically active - but is tectonically active. The Yellowstone hot spot is not (arguably) tectonically active, but is volcanically. and mantle plume hot spots. NZ Chile, are Subduction. Yellowstone, Iceland, are hot spots.<br>If there were room to add to this list, I would add Japan and the East African Rift.		

Ecosystem Functional Group (EFG)	General comment	Specific comments	Response type	Detailed response
F2.9 Geothermal wetlands		1. Move primary producers sentence forward with Energy sentence. s<br>2. Add "Thermophylic blue green algae have a growth temperature optimum above 45°C. "<br>3. Add to Mollusc sentence: "and crustaceans occupy less extreme microhabitats, the latter two macroinvertebrate groups particularly in hard water hot springs. Microinvertebrates are also common including rotifers and ostracods."<br>4. Add: "Longitudinal succession of animal and plant communities occur as temperatures cool and minerals precipitate and settle with distance downstream from the spring source."<br>5. Additions to figure: Under Biotic interactions add 'Longitudinal succession'. Under Ecological traits add "Thermophilic algae" and "Specialised grazers"	Multiple edits to text and diagram	1. Moved primary producers sentence forward with Energy sentence.<br>2. Added: "Thermophylic blue green algae reach optimum growth above 45oC. "<br>3. Molluscs and crustaceans occupy less extreme microhabitats (notably in hard water hot springs), as do vertebrates such as amphibians, fish, snakes and visiting birds. Microinvertebrates such as rotifers and ostracods are common."<br>4. Added: "A succession of animal and plant communities occur with distance from the spring source as temperatures cool and minerals precipitate."<br>5. Amended figure as follows: Added "Local gradient with distance from source" on arrow from Biotic interactions to Resources. Added 'Successional gradients' to Ecological traits. Under Ecological traits changed to 'Thermophilic and metallophilic microbes' to 'Thermophilic and metallophilic biota' to accommodate invertebrates and algae". Omitted 'aquatic macrophytes and 'Specialised grazers' due to space constraints

Ecosystem Functional Group (EFG)	General comment	Specific comments	Response type	Detailed response
F2.10 Subglacial lakes	I think these read very well. They're short, and so miss some things but that's inevitable given the space available. I'd say you could pt something in on governance of these environments (see the attached note from me and Chuck Kennicutt on subglacial lake governance, which you might find useful).	No specific comment	No change	

Ecosystem Functional Group (EFG)	General comment	Specific comments	Response type	Detailed response
Biome F3 Artificial fresh waters	Section F3 – section introduction. Contains some statements that are patently untrue – I have provided multiple references that show that the text is incorrect and I have deleted these sentences in the pdf – please remove them. They could negatively affect biodiversity conservation – we now have many endangered freshwater species dependent solely on anthropogenic habitat. Throughout section F3, the writers have focussed on the negative and ignored the many papers published in high quality journals giving examples of where anthropogenic habitats are highly diverse and/or providing vital habitat for native/threatened/endangered species (see examples in Chester & Robson 2013, and plenty more recent ones too). While it is true that many anthropogenic habitats are terribly depauperate, others are not. Indeed,	1. In some arid areas, amphibians now totally reliant on these to avoid extirpation. see Canals et al. 2011. Emerging conflicts for the environmental use of water in high-valuable rangelands. Can livestock water ponds be managed as artificial wetlands for amphibians? Ecological Engineering, 37, 1443-1452<br>2. Add ponds, ditches<br>3. found everywhere there are urban and rural land uses.<br>4. This is also false. Many of these waterbodies contain significant autochthonous production by macrophytes or algae. Water may often come in naturally from groundwater or runoff. This sentence is misleading and unecessary.<br>5. this is not the case - far too sweeping a statement. It completely depends what you are comparing them to and what they are like. You need to read the following references: Williams et al., 2003. Comparative biodiversity of rivers, streams, ditches and ponds in an agricultural landscape in Southern England. Biol Cons, 115, 329-341. Verdonschot et al. 2011. Biodiversity value of agricultural drainage ditches: a comparative analysis of the aquatic invertebrate fauna of ditches and small lakes. Aquatic Conservation, 21, 715-727<br>6. These and others are cited in Chester & Robson (2013) Anthropogenic refuges for freshwater biodiversity: their ecological	Multiple edits to text	General: We understand the reviewer's concern about maximising opportunities to manage artificial wetlands for their biodiversity values. Below we detail changes made to acknowledge those values. However, the generalisations made about diversity and functionality relate to comparisons of artificial wetlands with functional reference wetlands are and robust across global scales, which is the relevant context for a global typology. The references cited do not refute the generalisations, but rather point to important cases where artificial wetlands contribute to diversity and maintenance of landscape function and biodiversity. The changes listed below aim to clarify this context and recognise the significance of artificial wetlands more appropriately.<br>1. Added the following text: "Nonetheless, in some highly transformed landscapes, they may provide anthropogenic refuges and critical habitat for complementary suites of native biota to that remaining in depleted wetlands, including some biota that no longer occur in natural or semi-natural ecosystems, as well as a range of opportunistic colonists."<br>2. Added ponds, ditches<br>3. Added phrase to sentence as follows: These are globally distributed but are most often found in humid and subhumid tropical and temperate environments "where rural and urban developments are predominant."<br>4. Agree autochthonous production can be important in some waterbodies, but the generalisation is robust. Amended text to qualify the generalisation as follows: "For most of these ecosystems," energy, water, and nutrients come generally primarily from allochthonous sources, either incidentally from run-off (e.g. farm dams and storm water canals) "or groundwater", or deterministically by management (e.g. rice paddies, aquafarms, and wastewater ponds), "but autchthonous energy sources (in situ algae and macrophytes) can be important in some artificial waterbodies".<br>5. The relevant comparison for this profile is between artificial

Ecosystem Functional Group (EFG)	General comment	Specific comments	Response type	Detailed response
	they may be very valuable, especially in urban and rural landscapes where freshwater biota are under enormous pressure. Importantly, we can manage anthropogenic habitats to maximise their biodiversity capacity. Given that so much of our planet is now agricultural or urban land, if we are to have any species left at all, this is vital and urgent. I think the IUCN typology should give examples of biodiverse, healthy, functioning anthropogenic habitats to show what can and should be done to improve their condition and biodiversity value. This section should mention some of the basic, generic things that can be done in managing anthropogenic habitat to improve its capacity to support biodiversity. Sadly, this misinformation and lack of awareness of the literature on anthropogenic wetlands	characteristics and management. Biological Conservation, 166, 64-75<br>7. presence of these plants is the single most important factor.<br>8. actually, we know nowhere near enough about these to know their role for amphibious mammals, but at least platypus and beavers use them!		wetlands and their natural analogues (palustrine wetlands, rivers and lakes) on a global scale. The generalisation is robust in that context, but there are obviously exceptions. Local comparisons of agricultural ditches and ponds with depleted rivers and streams, or between ditches and small lakes (as in the cited refs) point to some locally important exceptions, but do not invalidate the generalisation globally. In addition to new text added to highlight exceptions (see pt 1), qualifications were added to text as follows to acknowledge variations: Artificial wetlands are "generally" less temporally variable, more spatially homogeneous, and "often supporting less" biological diversity and trophic complexity of their natural analogues.<br>6. Thank you for the reference. Added the term "anthropogenic refuge" to text (see pt 1)<br>7. Amended text as follows: ...most diverse can include "submerged or emergent plant communities, which promote habitat complex habitats and resource supply for" invertebrates, ...<br>8. Noted. Replaced "rarely" with "sometimes"

Ecosystem Functional Group (EFG)	General comment	Specific comments	Response type	Detailed response
	over the last 20 years continues on into subsequent sections where there are many statements that are not supported by evidence. I have supplied references to show the errors in some cases but became tired of this after a while and just deleted the incorrect text and replaced it with correct statements.			

Ecosystem Functional Group (EFG)	General comment	Specific comments	Response type	Detailed response
F3.1 Large reservoirs		1. Benthic detritivores and decomposers are only active in the profunal zone if it is oxygenated<br>2. these sorts of reservoirs, like in the photo, are far too deep to go through natural succession like this. they may be eutrophic due to human activities, but naturally would take at least hundreds of years. I suggest some alternative text.<br>3. Add"Many reservoirs lack fringing vegetation or aquatic macrophytes and have very low diversity of invertebrates and fish reliant on phytoplankton."<br>4. Change "Human management to release or divert water compounds natural variability in inflow and evaporation and may result in large variations in water level, producing wide-edged zones that are intermittently inundated or dry. This limits the productivity of water margins and the number of species able to persist there" to "Human management to release or divert water into and out of the reservoir result in large variations in water level, producing wide-edged zones that are intermittently inundated or dry so that few species able to persist there"<br>5. No it doesnt, not in the sorts of deep reservoirs you refer to. wind mixing never reaches the bed. This only occurs in dams that are metres deep, not tens of m.<br>6. This is not a suitable reference on its own as it contains zero ecology. Suggest you add: Dudgeon et al (2006) Freshwater biodiversity: importance, threats, status	Multiple edits to text	1. Added "if oxygenated" to sentence on profundal zone<br>2. Agree the analogy with large natural lakes was inappropriate. We removed this and amended sentence to clarify the cause of eutrophication.<br>3. Added: "Many reservoirs lack fringing vegetation or aquatic macrophytes and have very low diversity of invertebrates and fish reliant on phytoplankton."<br>4. Rephrased to clarify as follow:: "Managed release or diversion of water alters natural variability. Large variations in water level, produce wide margins that are intermittently inundated or dry, limiting productivity and the number of species able to persist there."<br>5. Agree - deleted "The wind-driven re-suspension of sediments increases the supply of nutrients and organic particles in the water column and reduces light available for photosynthesis. "<br>6. Added Clavero & Hormoso (2011)

Ecosystem Functional Group (EFG)	General comment	Specific comments	Response type	Detailed response
		and conservation challenges. Biol Reviews, 81, 163-182. Clavero & Hormoso (2011) Reservoirs promote the taxonomic homogenization of fish communities within river basins. Biodiversity & Conservation, 20-41-5..		
F3.1 Large reservoirs	Please forgive my delayed review; I hope it's still useful. This is a great product and clearly reflects a lot of expertise, time and effort. Thanks to you and your team for that! And thanks for inviting my opinions. I would love to hear back from you how my comments are received.	1. My initial reaction to this question was to say that we need some biogeographic distinction, especially within the F3.1 Large Reservoirs. Tropical reservoirs behave very differently to temperate reservoirs, especially with regards to productivity. I went back and saw that you have an added section in the document about adding lower levels of classification, so that was good to see. All I can say is that this is key, and perhaps should not be relegated to a follow-up effort.<br>2. As well, the importance of whether a reservoir is affected by upstream flow regulation or not is critical yet not mentioned.	Minor edits to text	1. Tropical and temperate reservoirs share basic functional traits (as described in the profile) that distinguish them ecologically from other freshwater functional groups of ecosystems. Despite these uniting features, each of these groups has different expressions in climatically different regions. These different expressions (including tropical and temperate reservoirs) are recognised at levels 4 and 5 of the typology.<br>2. Added the following text: "Inflow volumes may be regulated."

Ecosystem Functional Group (EFG)	General comment	Specific comments	Response type	Detailed response
F3.1 Large reservoirs	I think the freshwater EFGs don't require any other additions. The only one I would question is F2.2. I read through the text of F1 to F6, FT1.6 & 7, and F3.1 to 3.5. The text is general enough to capture the variety within any one EFG. I think some additions to the text on the invertebrate fauna of TF1.6 & TF1.7 is required. I have specific comments on the attached pdf.	Would be useful to include comment on the estimated global number of dams.	Minor edits to text	Added the following to Distribution text: "Globally, there are more than 3000 reservoirs with a surface area >50km2."

Ecosystem Functional Group (EFG)	General comment	Specific comments	Response type	Detailed response
F3.2 Constructed lacustrine wetlands		1. and/or contain emergent or submerged macrophytes<br>2. evidence for this? The 52 farm ponds I am studying atm almost all have submerged vegetation across all of the deeper areas. There is not enough published evidence on these wetlands to make statements like this<br>3. in some areas they are bedrock, so no rooted plants.<br>4. If you actually want to know about this, try reading papers like Linton & Goulder 2000. Botanical conservation value related to origin and management of ponds. Aquatic Conservation, 10, 77-91<br>5. (il)logical tautology - the sensitive spp that can be found in these wetlands are absent from ones with pollution - so yes, the polluted ones have a tolerant biota, but the non-polluted ones may not. delete.<br>6. this is also not substantiated by the literature - delete<br>7. this [trophic structure text] is also not substantiated by the literature - delete<br>8. Fish arrive via surface connections or are introduced by people. More and more often, rare, threatened or endangered species (e.g. odonates, mussels, amphibians) are surviving in anthropogenic ponds having been extirpated from natural waterbodies.<br>9. this is nonsense. Delete "but most species are widely dispersed opportunists, hence endemism is low."<br>10. this figure is incorrect and needs	Major revision of text and diagram	1. Added detail about submerged macrophytes, but retained text on amphibious macrophytes. Farm dams in production landscapes are activity managed to maintain open water, and only close over with emergent macrophytes when abandoned. Revised text reads, "They may be fringed by amphibious vegetation, or else bare ground is maintained by earthworks or livestock trampling. They often have submerged macrophytes and more rarely emergent macrophytes extending across the entire wetland."<br>2. As above, added detail about submerged macrophytes, but retained text on amphibious macrophytes. Farm dams in production landscapes are activity managed to maintain open water, and only close over with emergent macrophytes when abandoned. Revised text reads, "They may be fringed by amphibious vegetation, or else bare ground is maintained by earthworks or livestock trampling. They often have submerged macrophytes and more rarely emergent macrophytes extending across the entire wetland."<br>3. Added bedrock<br>4. Added: depending on "depth, shape, history and" management.<br>5. Deleted "Biota generally tolerate high pollutant levels. "<br>6. The profile needs to represent the full range of artificial water bodies, including the large numbers in intensive agricultural or industrial zones and semi-arid climates where many of the features associated with diversity are poorly developed. Generalisation was qualified to acknowledge that some artificial water bodies (those most likely to be of interest for study conservation biologists) have similar diversity to comparable natural ones, but many artificial ones lack the required habitat heterogeneity due to continual livestock use, inflows with high sediment, nutrient or pollutant loads, and periodic maintenance works. The deletion recommended by the reviewer would render the remaining text uninformative - biological diversity of all water bodies depends on the factors listed. Revised text seeks a

Ecosystem Functional Group (EFG)	General comment	Specific comments	Response type	Detailed response
		substantial modification to indicate the wide range of prevailing conditions, positive as well as negative.<br>11. Add household water storage, claypits, gravel pits, quarry pools, stormwater. They may also be constructed in natural landscapes for fire control (fire dams). Dlete groundwater seepage.<br>12. avoid colloquial use of the word 'chemicals', not scientific usage.<br>13. other materials, fish or plants,<br>14. Climate and weather affect hydrological patterns.<br>15. these are found everywhere in rural and urban areas, how can you say the terrain is flat!<br>16. Add "and absence of fringing trees may"<br>17. Replace "Substrates are typically mud or gravel, but cobbles and debris of varied sizes may be added and organic material may accumulate over time, increasing habitat complexity" with "Substrates vary widely, from silt, clay, sand and gravel through to cobbles or bedrock or a comination thereof. Particle sizes may decline if there is sedimentation or eutrophication."<br>18. Clearly you didnt actually read this paper! Because we refute, with evidence, many of the ignorant statements written above. Either correct the text or remove this reference. it does not support my revised version of the text above, but not		balance of these features: :Taxonomic and functional diversity varies from levels comparable to natural water bodies to are typically lower levels, and depending on productivity, the presence of aquatic or fringing vegetation, water chemistry, management regimes and proximity to other waterbodies or vegetation. "<br>7. As above. Only some artificial water bodies have upper trophic levels well represented in other systems.<br>8. Added fish<br>9. We think this is a defensible generalisation across the full range of artificial water bodies if appropriately qualified. We are unaware of any endemics that evolved in artificial water bodies, though there may be some microbial biota (hence 'low') and certainly some native biota that are highly depleted in natural habitats. For artificial water bodies that were constructed (by definition) on non-aquatic sites, the aquatic biota must be composed of colonists and many are widespread opportunists, rather than organisms with dispersal limitations. The rephrased text reads: "Endemism is generally low, but these waterbodies may be important refuges for some species now highly depleted in their natural habitats. Life histories often reflect those found in natural waterbodies nearby, but widely dispersed opportunists dominate where water quality is poor. "<br>10. Figure amended to reflect text edits: Productivity change from low-moderate to low-high; diversity changed from low to low-high; simple trophic structure changed to varied trophic complexity; managed and opportunistic bita noted as present rathert han dominant; channelisation changed to landscaping; chemicals changed to other chemicals; chemical toxins changed to chemical additives or pollutants; floods & droughts changed to climate and weather (in Ambient Environment)<br>11.Expanded text on origins of artificial waterbodies as follows: "Water bodies are constructed for agriculture, mining, ornamentation, stormwater, wastewater or other uses, or fill depressions left by earthworks, obstructing surface flow or

Ecosystem Functional Group (EFG)	General comment	Specific comments	Response type	Detailed response
		the original.		headwater channels." Deleted groundwater seepage<br>12. Chemicals is a scientifically correct term referring to flocculants, pesticides, herbicides, etc., as well as fertilisers (nutrients) added directly (e.g. to control 'water weed') or indirectly through inflow from managed catchments. Added examples of chemicals in parentheses nutrients<br>13. Added<br>14. Replaced "Droughts and storms contribute to resource variability" with "Climate and weather affect hydrological patterns"<br>15. Deleted to save space - there are fewer rural and urban areas and associated water bodies in mountainous terrain than flat terrain.<br>16. Added "and lack of shade may"<br>17. Revised text as follows: "Substrates include silt, clay, sand, gravel, cobbles or bedrock, and fine sediments of organic material may accumulate over time."<br>18. Revised in line with suggestions or clarified context or generalisations. Reference retained.
F3.2 Constructed lacustrine wetlands	Overall, this reads well and is fine	1. "…concentrated chemical toxins, "extremes of pH" or high salinities…".<br>2. …"for water quality management, and in the case of fish for human consumption."	Minor edits to text	1. Added "extremes of pH"<br>2. Added "or human consumption."

Ecosystem Functional Group (EFG)	General comment	Specific comments	Response type	Detailed response
F3.2 Constructed lacustrine wetlands	I think the freshwater EFGs don't require any other additions. The only one I would question is F2.2. I read through the text of F1 to F6, FT1.6 & 7, and F3.1 to 3.5. The text is general enough to capture the variety within any one EFG. I think some additions to the text on the invertebrate fauna of TF1.6 & TF1.7 is required. I have specific comments on the attached pdf.	1. including motorway attenuation ponds<br>2. Extent of the littoral area is also important as is the presence of complex habitat mosaics<br>3. Some groups can be diverse, e.g. Coleoptera<br>4. "These water bodies are constructed for agriculture, mining, ornamentation, or wastewater treatment"<br>5. Suggested reference: Becerra, G., Harrington, R. and Kelly-Quinn, M. (2012) A review of the potential of surface flow constructed wetlands to enhance macroinvertebrate diversity in agricultural landscapes with particular reference to Integrated Constructed Wetlands (ICWs). Hydrobiologia, 692 (1):121-130.	Multiple edits to text and refs	1. Motorway attenuation ponds are one of several purpose-built examples of artificial waterbodies. Unfortunately there is insufficient space to mention very many. We cite wastewater ponds and exacavation pits as extreme examples<br>2. Incorporated influence of the littoral area and presence of complex habitat mosaics with the following elaboration of text: "Taxonomic and functional diversity range from levels comparable to natural lakes to much less, depending on productivity, complexity of aquatic or fringing vegetation, water quality, management and proximity to other waterbodies or vegetation."<br>3. Coleoptera noted but it would be inappropriate to single them out from ther macrinvertebrates within space constraints<br>4. Noted, but insufficient space to add "treatment" to wastewater. The more general term also encompasses water bodies used wastewater storage without purposeful treatment<br>5. Replaced Downing et al. (2006) with Becerra, et al. (2012)

**Ecosystem Functional Group (EFG)**	**General comment**	**Specific comments**	**Response type**	**Detailed response**
F3.3 Rice paddies	Section F3.3 rice paddies – this is not my specific area of expertise but the text seemed rather vague to me. There are quite a few studies from a range of countries that show that rice paddies have biodiversity value under certain circumstances but not others – surely the summary here should tease these major points out? I have put a list of potential references in a comment box on the pdf, but there are likely some more recent ones available too. The diagram appears only to represent rice farming hostile to biodiversity not the many examples of rice systems that include native biodiversity – redraw to reflect both.	See general comment	No change	The existing text outlines the biodiversity of rice paddies and the factors that affect it as follows: "Opportunistic colonists include consumers such as invertebrates, zooplankton, insects, fish, frogs, and waterbirds, as well as other aquatic plants. Often they come from nearby natural wetlands or rivers and may breed within rice paddies. During dry phases, obligate aquatic organisms are confined to wet refugia away from rice paddies. These species possess traits that promote tolerance to low water quality and predator avoidance. Others organisms, including many invertebrates and plants, have rapid life cycles and dormancy traits allowing persistence as eggs or seeds during dry phases."
F3.3 Rice paddies	This is fine		No change	

Ecosystem Functional Group (EFG)	General comment	Specific comments	Response type	Detailed response
F3.3 Rice paddies	I think the freshwater EFGs don't require any other additions. The only one I would question is F2.2. I read through the text of F1 to F6, FT1.6 & 7, and F3.1 to 3.5. The text is general enough to capture the variety within any one EFG. I think some additions to the text on the invertebrate fauna of TF1.6 & TF1.7 is required. I have specific comments on the attached pdf.	1. Not an area that I am familiar with but seems reasonable.<br>2. define rapid life histories	Minor edit to text	1. Thank you<br>2. Added phrase to define 'rapid' as follows: "...have rapid life cycles completed in weeks or months ..."
F3.4 Freshwater aquafarms	Overall, this reads well and is fine.	Is there a category for saltwater aquafarms? E.g. mangroves converted into shrimp farms? Deep ocean aquafarms?	No change	Functional Group M4.2 includes marine aquafarms

Ecosystem Functional Group (EFG)	General comment	Specific comments	Response type	Detailed response
F3.4 Freshwater aquafarms	I have had a quick look at F 3.4 as well and make one proposed edit (see attached).	Propose: ‘Water lost through seepage and evaporation may be compensated for by addition of water from nearby lakes or streams. In more intensively managed pond systems, freshwater may also be added to address poor water quality and DO conditions, together with inputs of antibiotics and chemicals (e.g. pesticides and fertilisers), thereby influencing the physical, chemical, and biological properties of the water column and substrate’. (to replace "Removal of wastewater and replacement by freshwater from lakes or streams, together with inputs of antibiotics and chemicals (e.g. pesticides and fertilisers) influence the physical, chemical, and biological properties of the water column and substrate"]	Minor edits to text	Rephrased ""Removal of wastewater and replacement by freshwater from lakes or streams, together with inputs of antibiotics and chemicals (e.g. pesticides and fertilisers) influence the physical, chemical, and biological properties of the water column and substrate" as follows: "Water lost through seepage and evaporation may be replenished by water from nearby lakes or streams. In more intensively managed pond systems, freshwater may also be added to improve water quality and dissolved oxygen, together with inputs of antibiotics and chemicals (e.g. pesticides and fertilisers). These additives influence the physical, chemical, and biological properties of the water column and substrate."

Ecosystem Functional Group (EFG)	General comment	Specific comments	Response type	Detailed response
F3.5 Canals and storm water drains	F3.5 Canals and storm water drains. I really dislike the photo here, it shows a concrete lined ditch and pipes that would hold very little diversity, only representing hostile environments. Yet we know that whole countries, like the Netherlands, only have ditches as lotic ecosystems, but some of them hold quite good biodiversity, as they do elsewhere in Europe (see example reference on doc pdf). In Spain, endangered mussels are now found only in canals. I think it would be helpful to add a photo of a healthy canal with plenty of macrophytes and clear water. The photo used communicates a bias if used alone. It certainly doesn't show what canals can achieve in terms of sustaining freshwater biodiversity.<br>Also, I do not think that stormwater drains should be included at all – they should be dropped.	1. Delete stormwater drains, add urban and agricultural ditches<br>2. Some ditches that are ~>50 or 100 years old have developed considerable heterogeneity due to the development of fringing vegetation and woody debris. Agricultural ditches can comprise large amounts of aquatic habitat (e.g. 300 000 km in the Netherlands, 128 000 km in U.K.) In some countries (e.g. The Netherlands) ditches now comprise the only lotic habitat available for freshwater species<br>3. reference for this: Verdonschot et al. 2011. Biodiversity value of agricultural drainage ditches: a comparative analysis of the aquatic invertebrate fauna of ditches and small lakes. Aquatic Conservation, 21, 715-727<br>4. Rephrase "...are filamentous algae and macrophytes that…" to "…are macrophytes and attached algae…"<br>5. The lining materials of canals and ditches varies widely, from concrete and brick to wood, clay or simple earth. These materials determine the types of plants that can develop and whether fringing vegetation or erosion can create habitat heterogeneity.<br>6. does a 500 gram mussel count as small? not in the invertebrate world.<br>7. Text "Sealed or otherwise uniform canal bottoms and banks limit the diversity and abundance of benthic biota." not true. concrete lined channels	Multiple edits to text and refs	1. Rephrased the title to reflect terms in wide usage: "Canals, ditches and drains". Stormwater drains cannot be deleted because they better placed here than in any other group. The purpose of the typology is to include all ecosystems, bot only those with higher biodiversity values.<br>2. Added phrase: "..., though some older ditches have fringing vegetation, which contributes to structural complexity" Added "…including several hundred thousand kilometres of ditches and canals in Europe.<br>3. Added reference<br>4. Retained phrasing with algae first. Virtually all of these systems have algae, while macrophytes are important producers in some<br>5. Drivers section refers to "sealed or earthen substrates"<br>6. Added qualifier "mostly" small. Large molluscs may be present but are not characteristic of the system globally. They may sometimes be removed to prevent fouling.<br>7. Rephrased to acknowledge effect of different linings as follows: "While earthen banks and linings may support macrophytes and a rich associated fauna, sealed or otherwise uniform substrates limit the diversity and abundance of benthic biota."<br>8. Amended text as follows: "Fish and crustacean communities, when present, generally exhibit lower diversity and smaller body sizes compared to natural systems, and are often dominated by introduced or invasive species. Waterbirds, when present,typically include a low diversity and density of herbivorous and piscivorous species. "<br>9. Rephrased as follow: "Canals, ditches and drains may provide pathways for the dispersal or colonisation of native and invasive biota."<br>10. Added "Flows in some ditches may be very slow, approaching lentic regimes. "<br>11. Omitted "with flow"<br>12. Reduced sentence to ensure that substrate and water regimes

Ecosystem Functional Group (EFG)	General comment	Specific comments	Response type	Detailed response
	Stormwater drains hold water only for minutes to hours, so they can never be habitat for aquatic species. It would be much better instead to add urban and agricultural drainage ditches which can be very diverse (or not). I suggest a change in title and content, especially as ditches have been missed elsewhere. The diagram in this section needs to be redrawn to reflect positive canals and ditches not just negative ones.	can be limiting, but in fact there is a wide diversity of lining materials<br>8. Replace "Fish and crustacean communities, when present, exhibit low diversity and are often dominated by introduced or invasive species." with "Plant communities vary widely, but the presence of macorphytes and fringing vegetation are associated with higher animal diversity. Animdal diversity also varies widely, from a few invasive species to a full suite of native species, including endangered species, depending on the location, materials and age of the channel."<br>9. Add "dispersal" to sentence on canals as pathways<br>10. However, in many regions, ditches have negligible water flow, and may act like linear wetlands.<br>11. and lots of other things - omit the why<br>12. now covered above. Dlete "The earthen or hard surfaces (e.g. concrete) of banks and bottoms generally lack the heterogeneity present in natural streams, hence niche diversity is low. Deterministic water regimes and often shallow depths also limit niche diversity and have major influences on the physical, chemical, and biological properties of the canals and storm water drains."<br>13. lots of evidence in many places that it [water quality] is fine.		are covered as an ecosystem driver. Amended text on substrates now reads: "Substrates vary from earthen material to hard surfaces (e.g. concrete, bricks), affecting suitability for macrophytes and niche diversity." Text on water regimes moved forward before examples. Now reads: "Deterministic water regimes and often shallow depths have major influences on the physical, chemical, and biological properties of the canals, ditches and drains.<br>13. Changed "...water often carries high levels of …" to "...water may carry high levels of …"

Ecosystem Functional Group (EFG)	General comment	Specific comments	Response type	Detailed response
F3.5 Canals and storm water drains	I think the freshwater EFGs don't require any other additions. The only one I would question is F2.2. I read through the text of F1 to F6, FT1.6 & 7, and F3.1 to 3.5. The text is general enough to capture the variety within any one EFG. I think some additions to the text on the invertebrate fauna of TF1.6 & TF1.7 is required. I have specific comments on the attached pdf.	Not sure this is always the case, with regard to: “Their individuals [fish, crustaceansare smaller in size compared to natural sys[tems.”	Minor edits to text	Added qualifier to acknowledge exceptions as follows: "Fish and crustacean communities, when present, generally exhibit lower diversity and smaller body sizes compared to natural systems..."
Freshwater-Marine				
Biome FM1 Transitional waters	Overall, it looked very well written and my edits are fairly minor. My time has been stretched much more than I expected so I did not get to any additional ecosystems and I apologize for that!	1. I would suggest adding 'mostly' in front of restricted because there are deep water tropical systems such as the so-called tropical fjord Gulfo Dulce in Costa Rica'<br>2. Presumably 'water regimes' refers to physical, chemical, and biological conditions in aggregate?	Minor edits to text and diagram	1. I would suggest adding 'mostly' in front of restricted because there are deep water tropical systems such as the so-called tropical fjord Gulfo Dulce in Costa Rica'<br>2. Added 'chemical properties, to 'water regimes' which refers to physical conditions which, with other liste factors affect ecological/biological properties as listed?

Ecosystem Functional Group (EFG)	General comment	Specific comments	Response type	Detailed response
FM1.1 Deepwater coastal inlets		1. "They utilise a greater fraction of organic carbon..." They do not utilise more...They sequester more organic carbon into their sediments than many other estuaries, because they have steep slopes which allowing for terrestrial carbon to be transported into the fjord very efficiently and low oxygen bottom water which slow down decay processes.<br>2. Need to add the reference i sent you, that is by far the most extensive and updated on fjords.	Minor edits to text and refs	1. It may not have been clear that this generalisation about carbon utilisation refers to a comparison between warm- and cold-water fjords (both included within this functional group), not a comparison between fjords and other types of estuaries (in FM1.2 and FM1.3). We amended the text (based on Zaborska et al. 2018) as follows to clarify the context: "These fjords with warm water utilise a greater fraction of organic carbon and sequester it in sea-floor sediments at a slower rate than those with cold water.." In addition, we added a sentence to compare fjords with other estuaries based on the reviewer's comment as follows: "Deepwater inlets sequester more organic carbon into their sediments than other estuaries (FM1.2, FM1.3) because their steep slopes enable efficient influx of terrestrial carbon and low-oxygen bottom waters abate decay rates."<br>2. Replaced Slavanes (2001) with Bianchi TS, Arndt S, Austin WEN, Benn DI, Bertrand S, Cui X, Faust JC, Koziorowska-Makuch K, Moy CM, Savage C, Smeaton C, Smith RW, Syvitski J (2020) Fjords as Aquatic Critical Zones (ACZs). Earth-Science Reviews 203, 103145
FM 1.2 Permanently open riverine estuaries and bays	It reads very well and I can only imagine the time it took to synthesise all of the information into meaningful summaries, congrats! I don't have any major comments, just a few minor questions and suggestions that are hopefully helpful	FM1.2. Could you also write that fringing habitat includes intertidal and subtidal rocky shores? There is no discussion of anthropogenic drivers in this section, which I assume was deliberate since the anthropogenic sections are separate, but the image clearly shows structures and land-based activities that would have a significant impact on hydrodynamics and pollution in the system. Perhaps the image could be changed to depict a more unmodified estuary?	Minor edits to text	Added reference to rocky shores (MT1.1) as fringing and subtidal rocky reefs (M1.6) as occurring within estuaries. Anthropogenic influence is beyond scope of these profiles and will be addressed in a separate project

Ecosystem Functional Group (EFG)	General comment	Specific comments	Response type	Detailed response
FM 1.2 Permanently open riverine estuaries and bays	Overall, it looked very well written and my edits are fairly minor. My time has been stretched much more than I expected so I did not get to any additional ecosystems and I apologize for that!	1. should 'fringing vegetation' be 'shorelines' instead? Otherwise, it sounds odd to categorize muddy shores and sandy shores as 'fringing vegetation'<br>2. Add ", and rocky substrate" after M1.4).<br>3. 'Freshwater plumes' Maybe replace with 'Lower salinity'? Plumes needn't be strictly freshwater in order to export estuarine conditions outside the physical boundaries of the estuary (e.g., McHugh 1967)<br>4. Diagram. add 'freshwater' to the list of food web linkages. Also, here 'food web' is two words, in the text of the first paragraph, it is written as one word	Minor edits to text and diagram	1. changed 'fringing vegetation' be 'fringing shoreline systems' '<br>2. Added rocky shorelines and rocky reefs "<br>3. Replaced 'Freshwater plumes' with 'Lower salinity'<br>4. Added 'freshwater' to the list of food web linkages. Corrected 'food web' to two words, in the text
FM 1.2 Permanently open riverine estuaries and bays		Paper by Murry et al 2018 in Nature talk about global loss of mudflats, very good paper.	No change	Yes excellent paper, but mainly relevant to mapping the distribution of MT1..2 Muddy shores ecosystems, so not cited here
FM 1.3 Intermittently closed coastal lagoons	It reads very well and I can only imagine the time it took to synthesise all of the information into meaningful summaries, congrats! I don’t have any major comments, just a few minor questions and suggestions that are hopefully helpful	FM1.3. Along with the saltmarsh, seagrass communities are quite important in many of the lagoons I’ve worked in so I think it would be good to add that detail. While reading this I kept thinking about how extensively managed ICOLLs tend to be. Many don’t have a natural opening/closing regime anymore because that is controlled by regular dredging to prevent flooding. Perhaps there isn’t space for this, but it would be good to capture that anthropogenic driver if possible.	Minor edits to text	Added "and seagrasses (M1.1) occupy sandy bottoms of some lagoons." Human influence is out of scope of these profiles, but added brief reference to artificial opening, "Human-regulated opening influences many of these processes"

Ecosystem Functional Group (EFG)	General comment	Specific comments	Response type	Detailed response
FM 1.3 Intermittently closed coastal lagoons	Overall, it looked very well written and my edits are fairly minor. My time has been stretched much more than I expected so I did not get to any additional ecosystems and I apologize for that!	I would suggest including emergent/fringing vegetation (e.g., Phragmites) is another key source of detrital carbon to these food webs, that also provide important structural habitat in ICOLS	Minor edits to text and diagram	Included sentence before reference to saltmarsh and reedbeds as follows: "Emergent and fringing is a key source of detrital carbon to the food webs, and also provides important structural habitats." Added Fringing vegetation to Ambient environment box in Diagram
FM 1.3 Intermittently closed coastal lagoons		may want to say polyphenolic compounds (e.g., tannins)...since not all are tannins	Minor edits to text	Rephrased as follows: High "concentrations of polyphenolic compounds (e.g." tannins) in the water ...
FM 1.3 Intermittently closed coastal lagoons	To me, all profiles adequately cover the variation within those biomes and their global distribution as far as I know.	1. Could also be a function of many ICOLLs being in more temperate environments?<br>2. Photos: Names of sites would be helpful.<br>3. "depends" not "depend"<br>4. Balance between on and offshore net sediment transport in the entrance channel?<br>5. Also most common in temperate to semi-arid climates (if important).<br>6. "Intermittently??" - should this be the same as title of chapter?	Minor edits to text	1. Added phrase to accommodate role of latitude: ...mangroves (MFT1.2) are absent unless "the waters are warm and" the entrance opens frequently.<br>2. Locations being sought<br>3. Corrected "depend",<br>4. Rephrased to improve clarity as follows: "...depend on the balance between onshore and offshore sedimentation processes (which close the entrance) and..."<br>5. Thank you, for additional climatic information. However, limited space precludes addition, and point is partly covered by following phrase: "...mid-latitude coastlines with high inter-annual variability in rainfall and wave climate."<br>6. Replaces "Intermittent" with "Intermittently"

Ecosystem Functional Group (EFG)	General comment	Specific comments	Response type	Detailed response
Marine				
M1.1 Seagrass meadows		This looks good. I made one comment in the text to indicate you may want to say 'unvegetated soft sediment' as some seagrasses do grow well in 'softer' sediments.<br>Also, for your references, I would eliminate the DeBoer reference and include the following:<br>W. D. Larkum, R. J. Orth, and C. M. Duarte (eds). 2006. Seagrasses: Biology, Ecology and Conservation. Springer. The Netherlands. 691pp.<br>This book is the most referenced seagrass book yet and has chapters on all aspects of seagrass , including the info in the DeBoer reference	Minor edits to text and refs	1. Added the following word to phrase: surrounding "unvegetated" soft sediments<br>2. Added Larkum et al (2006) to ref list
M1.1 Seagrass meadows		1. macro and epiphytic algae compete with seagrass for light.. so i am not sure saying that the presence of these components enhances the productivity of seagrass meadows?<br>2. Note that many seagrass meadows occur in estuarine environments which are actually found in what the authors refer to as the transitional waters biome e.g. in lagoons and estuaries and not just the Marine Shelf Biome	Multiple edits to text	1. Replaced Seagrasses are the only subtidal marine flowering plants. Their high productivity is enhanced by macroalgae and epiphytic algae, supporting both detritus production and autochthonous trophic structures "" with the following text: "Seagrasses are the only subtidal marine flowering plants and underpin high productivity of these systems. Macroalgae and epiphytic algae, also contribute to productivity, supporting both detritus production and autochthonous trophic structures, but compete with seagrasses for light."<br>2. Yes Seagrasses may occur both on marine shelves and in confined transitional waters. We mention them in profiles for other functional groups (FM1.2, FM1.3) with which they may co-occur in the latter biome. Added the following text to Transitional waters biome profile: "In some cases, ecosystems characteristic of the marine shelf biome (e.g. M1.1 Seagrass meadows) may have significant occurrences within semi-confined transitional waters."

Ecosystem Functional Group (EFG)	General comment	Specific comments	Response type	Detailed response
M1.1 Seagrass meadows	Nice overviews of these marine systems. My comments are below. For some sections I included references. I recognize that there is space for just a reference or two in section, but thought the authors/editors would appreciate the basis for some of my comments in their revisions.	1. Overall looks good. Delete comma so that text reads ". . .living amongst their roots, epifauna and epifloral living on their shoots and leaves, as well as nekton. . .".<br>2. Depending on style, i.e. should have a comma so that it is i.e.,.<br>3. Says "waves erode seagrass". Really the waves are eroding the sediments in seagrass beds, not the seagrass itself as the sentence states.<br>4. Add some mention of turtles as either important grazer or ecological driver (though their historical importance has been diminished by overharvest of the turtles).<br>5. Storms can be important driver ofseagrass loss and successional dynamics – I see this is noted in the box&arrow diagram but could be mentioned in the text if space permits.<br>6. Say something about mutualism with lucinids that could play an important role in seagrass persistence.<br>7. Ref: Van der Heide, T., L. L. Govers, J. De Fouw, H. Olff, M. van der Geest, M. M. van Katwijk, T. Piersma, J. Van De Koppel, B. R. Silliman, A. J. P. Smolders, and J. A. van Gils. 2012. A three-stage symbiosis forms the foundation of seagrass ecosystems. Science 336:1432-1434	Multiple edits to text	1. Deleted comma.<br>2. Style template uses no comma after i.e. or e.g. throughout.<br>3. Rephrased "waves erode seagrass" as follows: "waves disturb seagrass and mobilise sediment"<br>4. Added turtles as follows: "Grazing megafauna such as dugongs, manatees and turtles can contribute..."<br>5. Text addresses storms as follows: "Large storm events and associated wave action lead to seagrass loss."<br>6. Added following text about lucinids: "Mutualisms with lucinid molluscs may influence seagrass persistence."<br>7. Added Van der Heide et al. (20102) to ref list
M1.2 Kelp forests	This desciptive profile looks good	No specific comments	No change	

Ecosystem Functional Group (EFG)	General comment	Specific comments	Response type	Detailed response
M1.2 Kelp forests	I found this section particularly well written	1. The map is misleading and inaccurate. The text states that kelp forests occur off Oman but the map does not indicate such<br>2. Also it indicate kelp forests in red along the east coast of south Africa to Mozambique..this is completely in accurate.	Amendments to map	1. Added minor occurrence on Oman coast<br>2. The area shown in the east of South Africa is the Natal marine bioregion, which does not extend as far north as Mozambique. There are records of Ecklonia maxima from that area, but we adjusted them to minor occurrences. See Werner & Filbee-Dexter (2019) map
M1.3 Photic coral reefs	This desciptive profile looks good	No specific comments	No change	
M1.3 Photic coral reefs	here you have few suggestions for the text you sent me some days ago. In my opinion, the text is very good and I only made some appreciations that can be considered (or not). Good job!	1. Add "gorgonians" to list of other sessile organisms<br>2. Add "and temperature anomalies" to final sentence in Drivers section<br>3. Diagram - Add mixotrophic to autotrophic energy<br>4. Diagram - Replace corals with scleractinians<br>5. Diagram - Add thermal anomalies to storms<br>6. Diagram - Replace "moderate sea temperatures" with "Small shifts in sea temperatures"	Minor edits to text and diagram	1. Added "gorgonians" to list of other sessile organisms<br>2. Added "and temperature anomalies" to final sentence in Drivers section<br>3. Diagram - Replaced "autotrophic energy" with "Autochthonous energy". Added "mixotrophic" to main text<br>4. Diagram - Replaced corals with scleractinians<br>5. Diagram - Added thermal anomalies to storms<br>6. Diagram - On arrow from "Moderate sea temperatures" box to Biotic interactions box, replaced "psychological suitability" with "Thermal sensitivity"

Ecosystem Functional Group (EFG)	General comment	Specific comments	Response type	Detailed response
M1.3 Photic coral reefs	I read your coral reef page. Very good. Good luck with this huge compilation!	1.Corals dont spawn synvronously in every place. Mostly they do, but you often see some going it alone!<br>2.Your sentence "Diversity is represented at high and low taxonomic levels." doesnt mean anything. At higher levels they are of course the most diverse system in the world.<br>3. "Reef structure and composition vary with depth gradients in light intensity and with turbulence, which diminishes with distance from the ocean front" Try this as: Reef structure and composition vary with gradients with depth, such as light intensity and turbulence, and exposure gradients, such as exposure itself and sedimentation.	Minor edits to text	General: Agree the distinction of depth is very important. A number of classifications (e.g. RAMSAR) follow size, recognising that large lakes have many distinctive features. Most lakes deep enough to extend beyond the photic zone are "large" ((F2.1) and/or freeze/thaw glacial (F2.4), but there are many important exceptions of smaller deep lakes. In order to maintain a manageable number of functional groups across the typology (Occam's Razor), this and variation within a number of other groups is managed by describing the key factors that influence variability in functionality. To emphasise the distinctions of depth further, we have enhanced detail about functional distinctions between small shallow and small deep lakes (see detail below).<br>1. Some of this is content is covered by existing text. To add further detail, we rephrased as follows: Shallow lakes tend to be more productive (by volume and area) than deep lakes "because light penetrates the entire water column, establishing competition between benthic macrophytes and phytoplankton", with more complex trophic networks and stronger top-down regulation leading to alternative stable states and possible regime shifts between them. "Macrophyte-dominated lakes support higher biodiversity than phytoplankton-dominated lakes ."<br>2. Addressed by edit in response to pt 1<br>3. While interesting, this detail is less important to the core description and could not be included due to space constraints.<br>4. Benthis macrophytes (including amphibious) address in response to pt 1<br>5. Addressed by edit in response to pt 1<br>6. Added more general text as follow: ", while he main predators are macroinvertebrates, fish, amphibians and birds."<br>7. Addressed by edit in response to pt 6. High diversity of shallow macrophyte-dominated lakes is mentioned in a previous sentence, "Clear lakes in macrophyte-dominated states support higher biodiversity than phytoplankton-dominated eutrophic lakes."

Ecosystem Functional Group (EFG)	General comment	Specific comments	Response type	Detailed response
				8. Deleted sentence<br>9. Deleted sentence. Revised text (pt 1) now states, "Shallow lakes tend to be more productive (by volume and area) than deep lakes because light penetrates the entire water column, establishing competition between benthic macrophytes and phytoplankton, with more complex trophic networks and stronger top-down regulation leading to alternative stable states and possible regime shifts between them. "<br>10. deleted<br>11. Amended text to: ...critical breeding habitat for waterbirds, "amphibians and reptiles."<br>12. Added groundwater to text and diagram<br>13. Retained emphasis on catchment<br>14. Added text as follows: "Some lakes (e.g. on leached coastal sandplains or peaty landscapes) have dystrophic waters."<br>15. Added "or dimictic" to monmictic<br>16. Added "salinity and tannins" to drivers<br>17. Assume concern is about occurrences in arid and semi-arid zones. There are some small permanent lakes in arid zones, but a large majority of lakes in those environments belong to other functional groups. Amended text to say "rarely" semi-arid or arid.
M1.3 Photic coral reefs	Nice overviews of these marine systems. My comments are below. For some sections I included references. I recognize that there is space for just a reference or two in section, but thought the authors/editors would appreciate the basis for some of my comments in their revisions.	No comments	No change	

Ecosystem Functional Group (EFG)	General comment	Specific comments	Response type	Detailed response
M1.4 Shellfish beds and reefs	Nice overviews of these marine systems. My comments are below. For some sections I included references. I recognize that there is space for just a reference or two in section, but thought the authors/editors would appreciate the basis for some of my comments in their revisions.	Statement that “Shellfish cannot survive prolonged periods of low dissolved oxygen” is true for many species, but there are some notably tolerant species such as Mercenaria mercenaria. Although the particular species that form beds and reefs may be among those with lower tolerances for hypoxia.<br>See:<br>Altieri, A. H. 2008. Dead zones enhance key fisheries species by providing predation refuge. Ecology 89:2808-2818.<br>Hochachka, P. W., and G. N. Somero. 2002. Biochemical adaptation. Oxford University Press, New York, New York, USA.	Minor edits to text	Qualified the statement, “Shellfish cannot survive prolonged periods of low dissolved oxygen” as follows: "Most reef- or bed-building" shellfish cannot survive prolonged periods of low dissolved oxygen.
M1.3 Photic coral reefs		1. You should probably mention soft coral too<br>2. i felt that this sentence on coral disease was a little out of place. It should possibly go in the section below on Key ecological drivers.<br>3. and certain butterfly fish and parrot fish<br>4. Seals generally not characteristic but only on exceptional coral reefs..i would not mention seals<br>5. i would include marine heat waves. These are probably the most important disturbance regime and are becoming more frequent	Multiple edits to text	1. Added soft corals after sponges<br>2. The Drivers section focuses primarily on physical drivers. Disease is best placed with other biotic traits<br>3. Replaced wrasses with parrot fish<br>4. Deleted seals<br>5. Added marine heat waves

Ecosystem Functional Group (EFG)	General comment	Specific comments	Response type	Detailed response
M1.4 Shellfish beds and reefs		1. 'endemism is low' I assume this is referring to associated biota, suggesting that few species depend on shellfish beds for habitat.<br>2. "predators are a sall component of the ecosystem" Hmm, I'm not sure we can really say this. Perhaps in terms of biomass but not in terms of process. The concept of keystone predation was based on seastar predation on intertidal mussels! Might just be an issue with what is meant by 'small component'<br>3. They are also important in biogeochemical cycling - they can be carbon sinks and can remove nitrogen from the system through N burial/sequestration and by increasing denitrification rates.<br>4. They are also sensitive to climate change stressors such as temperature (and associated increased risk of dessication for intertidal species), as well as lowered pH as they are calcifiers.	Multiple edits to text	1. Yes rephrased to clarify as follows: "...few of which are entirely restricted to oyster reefs"<br>2. Revised text to clarify as follows: Predators are a small component of the ecosystem "biomass", but are nevertheless important in influencing the recruitment, biomass, and diversity of prey organisms "(e.g. seastar predation on mussels)".<br>3. Added following text: "They are also important in biogeochemical cycling as carbon sinks, through N burial/sequestration and by increasing denitrification rates."<br>4. Added following text: "They are also sensitive to climate change stressors such as temperature (and associated increased risk of dessication for intertidal species), as well as lowered pH as they are calcifiers."
M1.4 Shellfish beds and reefs		1. For nutrients, don't the authors mean carbon? i.e. particulate not dissolved nutrients?<br>2. microhabitats provided either by abiogenic or biogenic structures. Comment: including the adults themselves in the case of Perna perna	Minor edits to text	1. Tides bring both nutrients and carbon into the system. Added carbon to the top Resources box<br>2. Although useful, this is a minor additional point, and there is insufficient space to include it without deleting other text.
M1.5 Photo-limited Marine animal forests		1. In fact coral reefs are considered also marine Animal Forests, but the shallow and mixotrophic ones...	Multiple edits to text	1. Added the following phrase to beginning of second sentence: "Unlike coral reefs (also an animal forest)," the major sessile animals include ...

Ecosystem Functional Group (EFG)	General comment	Specific comments	Response type	Detailed response
		2. eco-engineer (sensu Jones et al 1994) Jones, C.J., Lawton, J.H., Shachak, M., 1994. Organisms as ecosystem engineers. Oikos 69, 373e386.<br>3. "phylogenetic groups" not "group"<br>4. "benthic-pelagic coupling" add "processes"<br>5. "... often found near shallower, less photo-limited, highproductivity areas" Not necessarily: the Coralligene for example is one example of Marine Animal Forest and is a oligotrophic habitat<br>6. Marine Animal Forests are elsewhere, so ice scouring is only one particular (natural) disturbance<br>7. Also modified by climate change (warming, acidification and changes in primary productivity)<br>8. Diagram - I'm not sure if here you want to mention human or natural impacts: Bottom Trawling, Climate change (especially changes in water column and benthic primary productivity)<br>Rossi S, Isla E, Bosch-Belmar M, Galli G, Gori A, Gristina M, Ingrosso GM, Milisenda G, Orejas C, Piraino S, Rizzo L, Schubert N, Soares M, Solidoro C, Thurstan R, Viladrich N, Willis T, Ziveri C (2019) Changes of energy fluxes in the marine animal forest of the Anthropocene: factors shaping the future seascape. ICES Journal of Marine Sciences 76: 2008-2019 doi:10.109		2. Added definition of engineer to Glossary and Jones et al (1994) to reference list<br>3. Replaced "group" with "groups"<br>4. Added word as follows: benthic-pelagic coupling "processes"<br>5. Animal forests themselves are not high productivity but are often found adjacent to such systems. Add following text to clarify: "...these systems are generally of moderate productivity and" are often found near shallower, less photo-limited, high-productivity areas often found near shallower, less photo-limited, high productivity areas"<br>6. Marine Animal Forests are elsewhere, so ice scouring is only one particular (natural) disturbance<br>7 & 8. Human influences are beyond the scope of these profiles and will be addressed in a separate project - thank you for the reference

Ecosystem Functional Group (EFG)	General comment	Specific comments	Response type	Detailed response
M1.5 Photo-limited Marine animal forests	It reads very well and I can only imagine the time it took to synthesise all of the information into meaningful summaries, congrats! I don’t have any major comments, just a few minor questions and suggestions that are hopefully helpful		No change	This is a relatively new term in the literature - see cited book in ref list in the profile. It includes biogenic systems structured by a range of sessile invertebrate taxa, including, but not limited to sponge beds. For that reason, we opt to retain it and expect that usage will become more widespread
M1.5 Photo-limited Marine animal forests	Nice overviews of these marine systems. My comments are below. For some sections I included references. I recognize that there is space for just a reference or two in section, but thought the authors/editors would appreciate the basis for some of my comments in their revisions.	Overall good. States “though the latter are rarely dominant, cf. M1.4” in reference to bivalves. This seems to undermine the section M1.4, and contradicts evidence that bivalves can be “dominant” over habitats where they form reefs and beds which can be quite extensive and define large areas	Minor edits to text	Text was unclear, "rarely dominant" refers to shellfish in functional group M1.5, cf. M1.4 where their dominance is axiomatic.Rephased sentence as follows to clarify: "Unlike coral reefs and shellfish beds (also animal forests), the major sessile animals include sponges, aphotic corals, hydroids, ascidians, hydrocorals, bryozoans, polychaetes, and bivalves (the latter only dominate in M1.4)."

Ecosystem Functional Group (EFG)	General comment	Specific comments	Response type	Detailed response
M1.5 Photo-limited Marine animal forests		1. Major sessile animals include… surely ascidians too? What about benthic ctenophores?<br>2. " ..from their deepwater aphotic counterparts." but deepwater aphotic counterparts also lack macroalgae. So it purely comes done to the presence of microphytobenthos? Is this a justifiable basis on which to separate the two?<br>3. Comment: possibly also due to plankton emanating from upwelling<br>4. Diagram: "water clarity" consider replacing with "turbidity"	Minor edits to text	1. Added ascidians. Insufficient space to add ctenophores and other groups.<br>2. "lack" of macrophytes was incorrect. They may be present but never dominant cf. M3.7 from which all algae are absent from M3.7, creating differences in energy pathways. They are also distinguished by other depth-related processes (e.g. visual predators, pressure, etc.). To reflect this relationship more accurately, we redrafted the text as follows: "Low light limits primary productivity especially by macroalgae, although microphytobenthos can be important. Energy flow and depth-related processes distinguish these systems from their deepwater aphotic counterparts (M3.7)."<br>3. This seems likely to be a minor effect. Macroalgae dominant in many upwelling zones<br>4. Diagram: Turbidity implies suspended solids, however, light transmission can be affected by dissolved compounds as well. We use "Water opacity (e.g. tanins)" to make the point in the Diagram
M1.6 Subtidal rocky reefs	This desciptive profile looks good	No specific comments	No change	
M1.6 Subtidal rocky reefs		1. "...larval stages and spores are dispersed widely..." Reproductive modes among reef associated organisms is diverse (direct developing species, species with non-dispersing larvae, etc) so this statement is misleading.<br>2. Some species of green, brown and red algae are turfing species. Many other species of these three groups are not.<br>3. Holdfasts - These are found in macroalgae, not in turfing species<br>4. "Canopy algae are sparse at the depths or levels of wave exposure occupied by	Multiple edits to text and diagram	1. Rephrased as follows: "Reef-associated organisms have diverse dispersal modes. Some disperse widely as adults, some have non-dispersing larvae, others with sessile adult phases develop directly on substrates or have larval stages and spores dispersed widely by currents and during episodes of turbulence."<br>2. rephrased as follows: "To reduce dislodgement in storms, the macroalgae have holdfasts, while smaller species have low-growing 'turf' life forms."<br>3. See response #2<br>4. It is true that canopy algae may reach high densities, however such systems are assigned to a different functional group (M1.2) that occurs within a smaller range, mostly nested within the

Ecosystem Functional Group (EFG)	General comment	Specific comments	Response type	Detailed response
		this functional group (cf. kelp forests in M1.2)" - Untrue. Canopy-forming seaweeds can be quite dense and distributed over vast areas - for example Ecklonia radiata which is the dominant seaweed on reefs from central coast of WA and the way to northern NSW.<br>5. I don't feel this section captures the key ecological traits. For example the role that canopy-forming macrolagae play as ecosystem engineers and habitat-formers.<br>6. This has a very physical driver focus. Key interactions on reefs are nutrient sources and linkages between animal waste and algal productivity particularly in more nutrient-limited systems (non-upwelling systems). Subtidal reefs are also the poster child for top-down and bottom control, illustrating the trophic complexity of reef systems but also the consequences of loss of top-down control, the resulting trophic cascades (overabundance of urchins and overgrazing, formation of barrens, loss of kelp, etc). In terms of bottom-up control, it's around transitions between different forms of algae (turfing versus canopy forming) and the associated impacts on biodiversity when structural complexity is reduced). These are are well-known dynamics on subtidal rocky reefs, at least in temperate waters.		distribution of subtidal rocky reefs (M1.6). To make this clearer, we now mention it in the opening sentence, "Submerged rocky reefs host trophically complex communities lacking a dense macroalgal canopy (cf. M1.2)", as well as shifting the reference to M1.2 in the canopy density sentence: "Many also possess traits such as air lacunae within tissues or bladders that promote buoyancy. Canopy algae are sparse at the depths or levels of wave exposure occupied by this functional group (cf. kelp forests in M1.2)."<br>5. See response to point #4. Canopy-forming macroalgae play key functional roles in functional group M1.2, but not M1.6. This is the key distinguishing feature between them. See added text in point #4 to make this clearer<br>6. Some of this comment relates to functional group M1.2 Kelp forests (see response to comment #4). In the Drivers section, we added text as follows: "Animal waste is a key nutrient source sustaining algal productivity in more nutrient-limited systems." In the Traits section, we added text to the sentence on herbivores as follows: Herbivores promote diversity through top-down regulation, "influencing patch dynamics, trophic cascades and regime shifts involving kelp forests in temperate waters (M1.2). We also added top-down regulation to the diagram.

Ecosystem Functional Group (EFG)	General comment	Specific comments	Response type	Detailed response
M1.6 Subtidal rocky reefs		1. I would insert “often” between “are” and “low-growing”. This is because turf algae are considered a distinct functional group (<2cm in height) to macroalgae which is generally less than 50cm in height. Then there are the kelps..<br>2. Note that the fish can also be considered benthic because they are reef fish..<br>3. it is worth mentioning ascidians which can be prolific!<br>4. turbidity and riverine inputs are also an important driver not mentioned<br>5. Storms also shift sand around which is a key factor<br>6. Diagram: Ambient environment should perhaps include “sand movements”<br>7. Diagram: Disturbance regimes: sand inundation and scour<br>8. The reference is very old and there is a large body of academic papers on shallow rocky reefs<br>9. there is a problem with this map. It cannot be that the east coast of South Africa has no subtidal rocky reefs? Unless it is just the resolution? This could be fixed by giving the grey polygon for land a yellow border.	Multiple edits to text and diagram	1. Rephrased as follows: “…while smaller species have low-growing ‘turf’ life forms…”<br>2. Added text as follows: The latter two groups “and some fish” are benthic…<br>3. Added ascidians<br>4. Added phases to include turbidity as follows: A strong depth gradient and substrate structures (e.g. overhangs and caves) limit light availability, “as does turbidity.” Added phrases to include riverine input as follows: Currents “and river outflows” are crucial to the delivery of resources…<br>5. Added phrase as follows: Episodic storms generating more extreme turbulence “shift sand and” dislodge larger sessile organisms<br>6. Added arrow from Storms box to Substrate box labelled “Scour, sediment deposition”<br>7. As above<br>8. A search of WoS based on “rocky subtidal” returned 993 reference, but all recent, well-cited papers address specific aspects (trophic cascades, coastal infrastructure, invasions, local reefs, etc). Although old, the cited ref is a global review that fits reference selection criteria<br>9. Yes it is limited resolution. Rocky reefs are mapped on the shelf throughout, which happens to be narrow in that area. We will covert all maps to 0.5 degree resolution

Ecosystem Functional Group (EFG)	General comment	Specific comments	Response type	Detailed response
M1.7 Subtidal sand beds		1. There is a large difference between unconsolidated and consolidated sand beds, which impact on the stability and thus the disturbance regime of the habitat..<br>2. Some species of green, brown and red algae are turfing species. Many other species of these three groups are not.	Minor edits to text	1. Unconsolidated and consolidated sand beds, are described in different Ecosystem Functional Groups (M1.1 and M1.7, respectively), recognising differences in the stability and disturbance regimes, as well as other functional distinctions..<br>2. Added text to Traits section as follows: "Large bioturbators such as dugongs, stingrays and whales may also be important predators." Other comments are relevant to M1.8
M1.8 Subtidal mud plains		1. Key ecological traits in muddy sediments are the microbial community and in shallower water the microphytobenthos. Both perform critical ecological functions yet the text coverage is very macro-scale focussed. At microbes mediate most of the biogeochemical cycles in muddy sediments, I would think this should be the focus of this section, which would also make it more distinguishable from the subtidal sand plains text above.<br>2. Drift algae can be extensive over muddy sediments, particularly in sheltered bays and estuaries.<br>3. Suspension feeding tube worms such as Sabella can be common over muddy sediments when there are settlement substrates available.	Multiple edits to text	1. Rephrased primary production sentence as follows: "In situ primary production is contributed primarily by microphytobenthos, mainly benthic diatoms with green microalgae, as macrophytes are scarce or absent." Added the following text: "Abundant heterotrophic microbes process detritus. The microbial community mediates most of the biogeochemical cycles in muddy sediments, a features distinguishing these ecosystems from subtidal sand beds (M1.7)."<br>2. Added the following text: "Drift algae can be extensive over muddy sediments, particularly in sheltered waters."<br>3. Added the following text: "Suspension feeding tube worms may be common over muddy sediments when settlement substrates are available."

Ecosystem Functional Group (EFG)	General comment	Specific comments	Response type	Detailed response
M1.9 Upwelling zones	I’ve reviewed the upwelling zones section M1.9 - please find my comments as sticky notes in the pdf attached. This is a very useful document. Many thanks for the opportunity to look at it.	spawning condition (of fish) also plays an important role in recruitment success - spawners in better conditions produce more, larger and higher quality egss, for instance.<br>Hutchings, L., Barange, M., Bloomer, S.F., Boyd, A.J., Crawford, R.J.M., Huggett, J.A., Kerstan, M., Korrubel, J.L., De Oliveira, J.A.A., Painting, S.J., Richardson, A.J., Shannon, L.J., Schulein, F.H., Van der Lingen C.D., and Verheye, H.M. 1998. Multiple factors affecting South African anchovy recruitment in the spawning, transport and nursery areas. In Pillar, S.C., Moloney, C.L., Payne, A.I.L. and Shillington, F.A. Benguela dynamics: impacts of variability on shelf-sea environments and their living resources. S. Afr. J. mar. Sci. 19:211-255.<br>suggest to add currents to Upwelling waters Box.<br>and insert another arrow straight to the green circle, namely "transport versus retention of eggs and larvae"<br>I'd suggest removing the word "occasional" since some upwelling systems eg the Northern Benguela, are characterised by frequent, large scale low oxygen events.<br>Additional good upwelling refs:<br>Cury, P., Bakun, A., Crawford, R. J., Jarre, A., Quinones, R. A., Shannon, L. J., & Verheye, H. M. (2000). Small pelagics in upwelling systems: patterns of interaction and structural changes in	Minor edits to text, diagram and refs	Added spawning condition to explanation of variability, added currents to diagram, added arrow from currents box to biotic interactions for transport/retention of eggs, replaced 'intermittent' with 'periodic', added Demarq(2009), space limits preclude further refs

Ecosystem Functional Group (EFG)	General comment	Specific comments	Response type	Detailed response
		"wasp-waist" ecosystems. ICES Journal of Marine Science, 57(3), 603-618. Jarre-Teichmann, A., Shannon, L.J., Moloney, C.L. and Wickens, P.A. 1998. Comparing trophic flows in the southern Benguela to those in other upwelling ecosystems. In Pillar, S.C., Moloney, C.L., Payne, A.I.L. and Shillington, F.A. Benguela dynamics: impacts of variability on shelf-sea environments and their living resources. S. Afr. J. mar. Sci. 19:391-414. Demarcq, H. (2009). Trends in primary production, sea surface temperature and wind in upwelling systems (1998–2007). Progress in Oceanography, 83(1-4), 376-385		

Ecosystem Functional Group (EFG)	General comment	Specific comments	Response type	Detailed response
M1.9 Upwelling zones	These sections, overall, look sufficient to me. I made comments on the attached pdf. Summaries are included below. I did not spend time looking over other sections aside from these three. My comments are minor, and in only one case, does an important sentence need to be edited	1. This looks good for a one-page description. I would advise caution in stating that the phytoplankton blooms are diatom blooms. I think it's a bit of an oversimplification to indicate that these are diatom blooms (although they do often have a higher proportion of diatoms than typical in other regions).<br>2. I would add a note about the importance of those small pelagic fish populations to commercial industries.<br>3. I would not embrace the wasp-waist idea so whole-heartedly. Note that it's a hypothesis. My impression is that the wasp-waist idea of sardine, anchovy, and herring has fallen a bit out of favor in the last decade as biological oceanographers have become increasingly aware of the ecological roles of incredibly abundant mesopelagic fishes (as noted in section M2.2).<br>4. In the final sentence of the "drivers" section, I would add "low-ph" along with low-oxygen events. Low pH (acidification) typically accompanies the low oxygen events (as CO2 is--like oxygen consumption--associated with remineralization of organic matter). Here, I'd remiss if I did not mention that the systems are sensitive to interannual climate variability (like ENSO). The seasonal variability is noted, but it's the massive interannual variability that frustrates industries and scientists.	Minor edits to text	1. Added qualifier 'usually through' to 'diatom blooms' as suggested<br>2. Commercial industries noted but these are beyond the scope of the ecological profiles - no change<br>3. Added qualifier to wasp-wait, "has been hypothesised to drive ecosystem dynamics through 'wasp-waist' trophic control" and added phrase "...although diel-migrant mesopelagic fish (M2.2) may<br>have important regulatory roles."<br>4. Added reference to climate cycles "low-ph" after "low-oxygen" events by rephrasing as follows: "high variability due to large-scale interannual climate cycles (e.g El Niño Southern Oscillation), as well as the pulsed and seasonal nature of the driving winds, and periodic low-oxygen, low pH events due to high biological activity and die-offs."

Ecosystem Functional Group (EFG)	General comment	Specific comments	Response type	Detailed response
M1.10 Rhodolith/ Maerl beds		1. The red color comes from three pigments (phycoerythrin, phycocyanin, and allophycocyanin), which allow them to absorb in the blue and green range of the visible light, the range that can enter deepest into the water column.<br>2. Regarding the importance of allochthonous energy, to date there exist only two studies that show that these habitats might play a role in nutrient cycling, but rather by supplying nutrients to the water column than the other way around. Also, information on rhodolith bed productivity is extremely scarce and hence, saying that it is lower than in other habitats might be overstating it.<br>3. Not correct, their size varies within a large range, depending on location and rhodolith species and there is no average size.<br>4. No evidence available so far. Also, calcium and magnesium are not nutrients. Add "They play a role in benthic nutrient cycling and represent significant long-term carbonate stores. "<br>5. Not only fishes, but also other macrofauna can move rhodoliths. This is included above "rhodoliths get moved by water motion and bioturbators".<br>6. The draft map seems to exaggerate the distribution of rhodolith beds, even	Multiple revisions to text and map	1. Modified and shortened sentence as follows: "Their red pigments enable red algae to absorb more blue and green light than most photoautotrophs." Modified sentence in Ecological drivers as follows: "By exploiting diffused blue and green light more effectively than other photoautotrophs, rhodoliths form extensive beds on open coasts on the mid shelf and in tide-swept channels where the water column and suspended sediment diminish red light."<br>2. Deleted reference to allochthonous energy due to lack of formal studies (though filter feeders and mobile fauna surely benefit from external energy). Similarly, although quantified, several authors comment on productivity levels, hence modified sentence to qualify to generalisation as follows: "Rhodolith primary productivity is likely to be lower than in sea grasses (M1.1) and kelp forests (M1.2), although macrophytes add to primary production in shallow waters."<br>3. Deleted generalisation about nodule size (sourced from Riosmena-Rodríguez, R Nelson W, Aguirre J(2017)<br>4. Replaced sentence on nutrient cycling as suggested<br>5. Replaced sentence with explicit mention of both fish and macroinvertebrates: "Compared to coral reefs (M1.3), shellfish beds (M1.4) or marine animal forests (M1.5), in which rhodoliths may occur as minor components, they are usually less rugose and less stable, due displacement or aggregation by water motion and bioturbators such as fish and macroinvertebrates."<br>6. Replaced course map with modelled distribution from Fragkopoulou et al. 2021.<br>7. Previous image replaced with permission.

Ecosystem Functional Group (EFG)	General comment	Specific comments	Response type	Detailed response
		compared to the modelled distribution (see below from Fragkopoulou et al. 2021; green- polar/cold-temperate species, red- tropical/warm-temperate species, blue- overlapping regions). See Fragkopoulou E, Serrão EA, Horta PA, Koerich G, Assis J (2021) Bottom trawling threatens future climate refugia of rhodoliths globally. Frontiers in Marine Science 7, 594537.<br>7. This is one of my photos you can use. It is from Brazilians southernmost rhodolith bed in Arvoredo, Santa Catarina		

Ecosystem Functional Group (EFG)	General comment	Specific comments	Response type	Detailed response
M1.10 Rhodolith/ Maerl beds	Much of the document is fine – but there are some odd sentences which look to have been partially edited and no longer flow/make sense.	1. 30-50% cover [of rhodoliths] within beds<br>2. Sentence on pigments is incorrect. Red algae have a number of light harvesting pigments. The phycobilins that are typical of red algae consist of phycoerythrobilin (red) and phycocyanobilin (blue) –<br>There are three classes of phycobilins – phycoerythrins absorb in the green region (495-570nm), phycocyanns absorb in the green-yellow region (550-630 nm) and allophycocyanins in the orange -red region (650-670 nm).<br>The algal pigments that absorb in the blue-green region are fucoxanthin, B-carotene, and siphonoxanthin.<br>The underwater light environment is rich in green light and poorly exploited by chlorophylls.<br>3. Not sure what point this sentence is making: "However, allochthonous energy is likely important in sustaining the trophic web because rhodolith primary productivity is lower than in sea grasses (M1.1) and kelp forests (M1.2), although macrophytes add to primary production in shallow waters"<br>4. "on average c. 6 cm diameter". This is quite large – I would have though a smaller diameter might be more typical – ca. 3-5cm<br>5. "are usually less rugose and less stable than coral reefs (M1.3), shellfish beds (M1.4) or marine animal forests (M1.5), in which rhodoliths may occur as minor	Multiple revisions to text and map	1. Added 'within beds'<br><br>2 Corrected sentence as follows: "Their pigments enable red algae to absorb green-blue light efficiently, in addition to red-orange light."<br><br>3. Allochthonous energy sources are proportionately more important in rhodolith beds because autochthonous macrophytes are more abundant in seagrass and kelp systems.<br><br>4. c 6 cm was a published estimate (Riosmena-Rodríguez et al. 2017), It is not important detail to the description and was therefore deleted.<br><br>5. Sentence restructured as follows: "Compared to coral reefs (M1.3), shellfish beds (M1.4) or marine animal forests (M1.5), in which rhodoliths may occur as minor components, they are usually less rugose and less stable, due displacement or

Ecosystem Functional Group (EFG)	General comment	Specific comments	Response type	Detailed response
		components”. This part of the sentence seems to be disconnected from the earlier section – doesn’t flow or make particular sense -?<br>6. “Most common at depths of 50-150m”. No – frequently also in shallower waters (ca. 5-20m) – and sometimes even in upper subtidal zone.<br>7. “Rhodoliths are able to exploit diffused blue light more effectively than other photoautotrophs, which may explain extensive beds on open coasts on the mid shelf and in tide-swept channels where the water column and suspended sediment diffuse light.” Not true – there are other algae well adapted to lowlight environments and also to the spectral composition of deeper waters - mesophotic communities of macroalgae include a lot of green algae which are well adapted to the spectral composition – and there are also brown algae and other reds which are well adapted both in terms of photosynthetic pigments and morphologies for growth in low light /light in blue-green wavelengths.<br>8. The map seems reasonable but I think there needs to be indication of the beds in the Gulf of Mexico (mentioned in text but red only showing on Gulf of California side). A bit surprised by the extent of red off eastern Canada -?		aggregation by water motion and bioturbators such as fish and macroinvertebrates.”<br>6. Corrected as follows: “They are most common at depths of 50-150m”.<br>7. Revised text as follows: “Rhodoliths form extensive beds on open coasts on the mid shelf and in tide-swept channels where the water column and suspended sediment diminish red light.”<br>8. Text reference to Gulf of Mexico corrected to Gulf of California. Map replaced with more recent model of Fragkapoulou et al. (2021), which shows a smaller area off eastern Canada/US.

Ecosystem Functional Group (EFG)	General comment	Specific comments	Response type	Detailed response
		1. "non-geniculate (non-jointed), free-living, slow-growing, long-lived coralline algae". Replace unjointed with unattached.<br>2. Add, "In particular environments, rhodoliths can function as refugia for species that would otherwise be absent, such as deep kelp forests (M1.2) of Laminaria abyssalis."<br>3. Add pH and temperature as important drivers.<br>4. Add, "while bottom fishing disturbances cause profound or irreversible damage."<br>5. Replace draft map with data from: Fragkopoulou E, Serrão EA, Horta PA, Koerich G, Assis J (2021) Bottom trawling threatens future climate refugia of rhodoliths globally. Frontiers in Marine Science 7, 594537		1. Unjointed is correct meaning for non-geniculate (see http://www.mobot.org/mobot/latindict/keyDetail.aspx?keyWord=geniculatus#:~:text=geniculatus%2C%2Da%2C%2Dum,like%20a%20bent%20knee%2C%20lit.), while "free-living" addresses unattached form in the description text<br>2. Addition information is limited by space, but modified text as follows: Large rhodoliths appear to facilitate "*deepwater kelp as well as*" feeding and reproduction in fish and invertebrates.<br>3. Added pH and temperature to relevant text: "Light availability, *pH* and hydrodynamics are important drivers of variation in biotic assemblages, as are *temperatures*."<br>4. While this is an important issue, the profiles are limited to describing the reference (unaltered) state of the ecosystem group. Human influence will be addressed in a future publication.<br>5. Map replaced with new spatial model made available by authors.

Ecosystem Functional Group (EFG)	General comment	Specific comments	Response type	Detailed response
M2.1 Epipelagic ocean waters	Very nice piece of work here. I have examined the sections, and any comments I have before are listed by section, IN CAPS.	JUST ONE COMMENT HERE – I WOULD AMEND THE STATEMENT “Mid-ocean subtropical gyres, for example, are characteristically oligotrophic, with lower diversity and productivity than other parts of the ocean surface” to say “Mid-ocean subtropical gyres, for example, are characteristically oligotrophic, with lower productivity than other parts of the ocean surface.” I SAY THIS BECAUSE IN MANY REGIONS, THE DIVERSITY OF THE GYRES CAN BE EXTREMELY HIGH FOR SOME TROPHIC LEVELS, MUCH HIGHER THAN HIGHER LATITUDES.	Minor edits to text	Deleted 'diversity as suggested

**Ecosystem Functional Group (EFG)**	**General comment**	**Specific comments**	**Response type**	**Detailed response**
M2.1 Epipelagic ocean waters	Overall I find the document to be well-written and the EFGs chosen are appropriate. There is no need for major changes. Otherwise I have no major comments. I have made annotations to the attached text. I found that the important points have been captured in the very succinct text and diagrams in an exemplary way. Congratulations to the authors!	In section M2.1 Epipelagic open waters, I expected more about the phytoplankton and zooplankton that drive the system. These are important organisms but the text focusses on the higher the trophic levels "crustaceans, fish, cephalopods, marine mammals, and seabirds". You may not wish to expand the text and I appreciate describing the planktonic food chains in different ocean regions could be very challenging. I suggest inserting a little more about the base of the marine food chain, at least mentioning diatoms and copepods. A useful reference is: Anderson, T. R., Martin, A. P., Lampitt, R. S., Trueman, C. N., Henson, S. A., and Mayor, D. J. Quantifying carbon fluxes from primary production to mesopelagic fish using a simple food web model. – ICES Journal of Marine Science, 76: 690–701. doi:10.1093/icesjms/fsx234<br>And replace "lower" with "deeper"	Minor edits to text	Rephrased existing text, "Removal of wastewater and replacement by freshwater from lakes or streams, together with inputs of antibiotics and chemicals (e.g. pesticides and fertilisers) influence the physical, chemical, and biological properties of the water column and substrate." as follows: "Water lost through seepage and evaporation may be replenished by water from nearby lakes or streams. In more intensively managed pond systems, freshwater may also be added to improve water quality and dissolved oxygen, together with inputs of antibiotics and chemicals (e.g. pesticides and fertilisers). These additives influence the physical, chemical, and biological properties of the water column and substrate."

Ecosystem Functional Group (EFG)	General comment	Specific comments	Response type	Detailed response
M2.1 Epipelagic ocean waters	These sections, overall, look sufficient to me. I made comments on the attached pdf. Summaries are included below. I did not spend time looking over other sections aside from these three. My comments are minor, and in only one case, does an important sentence need to be edited	1. I felt that this section is good, but somewhat biased towards the subtropical gyres of the pelagic realm. There may be room to have "tropical and subtropical pelagic" and "arctic and subarctic pelagic" functional groups. The latter would be characterized more by seasonal variability and lower levels of light penetration (due to lack of sunlight during winters and high phytoplankton productivity during the summers), whereas the current description applies more to the clear, warm tropical and subtropical waters. This bias towards the subtropical and tropical areas is also clear in the diagrammic assembly model.<br>2. I am uncertain whether the statement regarding lower diversity in oligotrophic waters is clearly supported. See, for example, the Introduction of https://doi.org/10.1093/plankt/fbp119. I also thought this statement is somewhat in conflict with the diagrammic assembly model that indicates "high diversity."<br>3. Be careful not to suggest that the deep ocean lacks nutrients. It is abundant in inorganic nutrients, but organic (particulate) nutrient concentrations are low.	Minor edits to text and diagram	1. We agree that differences between the tropical/subtropical epipelagic systems and the polar/subpolar epipelagic waters warrant greater emphasis. Recognising different functional groups is an option, however, we think it is better to describe the latitudinal gradient in a single functional group, and recognise subdivisions at lower levels of classification (levels 4 and 5) where compositional differences are also evident. We referred to subtropical gyres as examples of large-scale variability in epipelagic waters. To address latitudinal gradients and reduce apparent emphasis on subtropical gyres, we added the following text to the traits section, "Seasonal variation in productivity is greater at high latitudes due to lower light penetration and duration in winter compared to summer, " and amended text in the drivers section, "The epipelagic zone is structured by a strong depth gradient in light, which varies seasonally at high latitudes. Light also varies with local turbidity, but at lower latitudes may extend to ~200 m where light attenuates to 1% of surface levels." In the diagram, we added latitudinal gradient to the arrow from the Light box, and "Seasonal variation in productivity with latitude to Traits circle.<br>2. Deleted diversity from text and diagram<br>3. Added "particulate" source of nutrients to text

Ecosystem Functional Group (EFG)	General comment	Specific comments	Response type	Detailed response
M2.2 Mesopelagic ocean waters	Very nice piece of work here. I have examined the sections, and any comments I have before are listed by section, IN CAPS.	I WOULD AMEND THE FOLLOWING STATEMENT "The diverse organisms within this layer consume and reprocess allochthonous organic material sinking from the upper, photosynthetic layer. Hence, upper mesopelagic waters include layers of concentrated plankton, bacteria, and other organic matter sinking from the more diverse and heterogeneous epipelagic zone (M2.1)" TO SAY "The diverse organisms within this layer consume and reprocess allochthonous organic material sinking from the upper, photosynthetic layer. Hence, upper mesopelagic waters include layers of concentrated plankton, bacteria, and other organic matter sinking from the heterogeneous epipelagic zone (M2.1)" (THE DAYTIME MESOPELAGIC ZONE IS GENERALLY MORE DIVERSE THAN THE DAYTIME EPIPELAGIC).	Minor edits to text	Deleted 'more diverse' as suggested

Ecosystem Functional Group (EFG)	General comment	Specific comments	Response type	Detailed response
M2.2 Mesopelagic ocean waters	Overall I find the document to be well-written and the EFGs chosen are appropriate. There is no need for major changes. Otherwise I have no major comments. I have made annotations to the attached text. I found that the important points have been captured in the very succinct text and diagrams in an exemplary way. Congratulations to the authors!	Not all species migrate and not all individuals of a species migrate. An important component of mesopelagic biomass does not move. suggest change "All" to "Many"<br>It is not clear what "reflective" means in this context. Need to explain it somehow or delete it. "Bioluminescence is present in more than 90% of mesopelagic organisms often with silvery reflective skin (e.g. lantern fish). Fish in the lower mesopelagic zone (>700 m) are less reflective and mobile due to reduced selection pressure from visual predators in low light conditions."	Minor edits to text	Corrected 'All' to 'Many'. Replaced bioluminence & reflective text
M2.2 Mesopelagic ocean waters	These sections, overall, look sufficient to me. I made comments on the attached pdf. Summaries are included below. I did not spend time looking over other sections aside from these three. My comments are minor, and in only one case, does an important sentence need to be edited	1. This section is also good. In a manner similar to the previous epipelagic section, it's necessary to make it clear that the statement regarding low nutrient availability applies to particulate organic nutrients (not to inorganic nutrients, which are typically high in both the mesopelagic and deeper layers).<br>2. Do not state that “all” species undertake diel vertical migration. Instead, consider saying that some species undertake diel vertical migration.	Minor edits to text	1. Added ...or where lower "particulate" nutrient levels ....<br>2. Replaced “All” with "Many" species undertake diel vertical migration.
M2.3 Bathypelagic ocean waters	Very nice piece of work here. I have examined the sections, and any comments I have before are listed by section, IN CAPS.	M2.3. BATHYPELAGIC – MOST BATHYPELAGIC FAUNA DO NOT VERTICALLY MIGRATE	Minor edits to text	Corrected text to "These organisms generally do not migrate vertically, in contrast to those in the mesopelagic zone (M2.2)"

Ecosystem Functional Group (EFG)	General comment	Specific comments	Response type	Detailed response
M2.3 Bathypelagic ocean waters	Overall I find the document to be well-written and the EFGs chosen are appropriate. There is no need for major changes. Otherwise I have no major comments. I have made annotations to the attached text. I found that the important points have been captured in the very succinct text and diagrams in an exemplary way. Congratulations to the authors!	The term "bathyal" is used for depths 200 m -3000 m in the benthic environment, Stick to the term "bathypelagic"	Minor edits to text	Replaced bathyl with bathypelagic
M2.4 Abyssopelagic ocean waters	Overall I find the document to be well-written and the EFGs chosen are appropriate. There is no need for major changes. Otherwise I have no major comments. I have made annotations to the attached text. I found that the important points have been captured in the very succinct text and diagrams in an exemplary way. Congratulations to the authors!	Nil	No change	

Ecosystem Functional Group (EFG)	General comment	Specific comments	Response type	Detailed response
M2.5 Sea ice	Overall I find the document to be well-written and the EFGs chosen are appropriate. There is no need for major changes. Otherwise I have no major comments. I have made annotations to the attached text. I found that the important points have been captured in the very succinct text and diagrams in an exemplary way. Congratulations to the authors!	No specific comment	No change	
M2.5 Sea ice	I think these read very well. They're short, and so miss some things but that's inevitable given the space available. I'd say you could pt something in on governance of these environments (see the attached note from me and Chuck Kennicutt on subglacial lake governance, which you might find useful).	No specific comment	No change	

Ecosystem Functional Group (EFG)	General comment	Specific comments	Response type	Detailed response
M3.1 Continental and island slopes	Overall I find the document to be well-written and the EFGs chosen are appropriate. There is no need for major changes. Otherwise I have no major comments. I have made annotations to the attached text. I found that the important points have been captured in the very succinct text and diagrams in an exemplary way. Congratulations to the authors!	patches of hard substrate such as rocky outcrops on steep slopes and on canyon walls are important. Also add "and pelagic fauna impinging on the slopes"	Minor edits to text	added detail on rocky outcrops and pelagic fauna impinging on slopes
M3.1 Continental and island slopes	Very nice piece of work here. I have examined the sections, and any comments I have before are listed by section, IN CAPS.	Nil	No change	
M3.1 Continental and island slopes	here you have few suggestions for the text you sent me some days ago. In my opinion, the text is very good and I only made some appreciations that can be considered (or not). Good job!	Once upon a time, these bottoms were also inhabited by Marine Animal Forests, especially cnidarians and sponges...bottom trawling swiped all out	No change	Noted. Text mentions these animal forests on the slopes (M3.5) as follows: "...may be juxtaposed with specialised ecosystems such as submarine canyons (M3.2), deep-water biogenic systems (M3.6), and chemosynthetic seeps (M3.7), ..."

**Ecosystem Functional Group (EFG)**	**General comment**	**Specific comments**	**Response type**	**Detailed response**
M3.2 Submarine canyons	Very nice piece of work here. I have examined the sections, and any comments I have before are listed by section, IN CAPS.	Nil	No change	
M3.2 Submarine canyons	Overall I find the document to be well-written and the EFGs chosen are appropriate. There is no need for major changes. Otherwise I have no major comments. I have made annotations to the attached text. I found that the important points have been captured in the very succinct text and diagrams in an exemplary way. Congratulations to the authors!	Nil	No change	
M3.3 Abyssal plains	Very nice piece of work here. I have examined the sections, and any comments I have before are listed by section, IN CAPS.	Nil	No change	

Ecosystem Functional Group (EFG)	General comment	Specific comments	Response type	Detailed response
M3.3 Abyssal plains	Overall I find the document to be well-written and the EFGs chosen are appropriate. There is no need for major changes. Otherwise I have no major comments. I have made annotations to the attached text. I found that the important points have been captured in the very succinct text and diagrams in an exemplary way. Congratulations to the authors!	occasional large carrion falls can be important (e.g. dead whales). These can later become important chemosynthetic environments but initially there is a big local input of organic matter.	Minor edits to text	Added "Large carrion falls are major local inputs of organic matter and can later become important chemosynthetic environments (M3.7). "
M3.4 Seamounts, ridges and plateaus	Overall I find the document to be well-written and the EFGs chosen are appropriate. There is no need for major changes. Otherwise I have no major comments. I have made annotations to the attached text. I found that the important points have been captured in the very succinct text and diagrams in an exemplary way. Congratulations to the authors!	I suggest delete this word, or substitute with " slope dwelling" or similar.	Minor edits to text	Replaced mountain with slope-dwelling

Ecosystem Functional Group (EFG)	General comment	Specific comments	Response type	Detailed response
M3.4 Seamounts, ridges and plateaus	Very nice piece of work here. I have examined the sections, and any comments I have before are listed by section, IN CAPS.	Nil	No change	
M3.4 Seamounts, ridges and plateaus	here you have few suggestions for the text you sent me some days ago. In my opinion, the text is very good and I only made some appreciations that can be considered (or not). Good job!	As in the submarine canyons, here you find huge Marine Animal Forests inn the form of Cold Water Coral reefs and sponge grounds	No change	Noted. Text mentions these animal forests on the seamounts (M3.5) as follows: "High densities of sessile animals may form deep-water biogenic beds (M3.5), ..."
M3.5 Deepwater biogenic beds	Overall I find the document to be well-written and the EFGs chosen are appropriate. There is no need for major changes. Otherwise I have no major comments. I have made annotations to the attached text. I found that the important points have been captured in the very succinct text and diagrams in an exemplary way. Congratulations to the authors!	Nil	No change	

Ecosystem Functional Group (EFG)	General comment	Specific comments	Response type	Detailed response
M3.5 Deepwater biogenic beds	Very nice piece of work here. I have examined the sections, and any comments I have before are listed by section, IN CAPS.	Nil	No change	
M3.5 Deepwater biogenic beds	here you have few suggestions for the text you sent me some days ago. In my opinion, the text is very good and I only made some appreciations that can be considered (or not). Good job!	In this case, also bottom trawling and climate change (ocean acidification and low levels of oxygen) are major threats for these communities<br>Rossi S, Isla E, Bosch-Belmar M, Galli G, Gori A, Gristina M, Ingrosso GM, Milisenda G, Orejas C, Piraino S, Rizzo L, Schubert N, Soares M, Solidoro C, Thurstan R, Viladrich N, Willis T, Ziveri C (2019) Changes of energy fluxes in the marine animal forest of the Anthropocene: factors shaping the future seascape. ICES Journal of Marine Sciences 76: 2008-2019 doi:10.1093/icesjms/fsz147	Added reference	Human influences are beyond the scope of these profiles and will be addressed in a separate project - thank you for the excellent reference. We found space to add it
M3.6 Hadal trenches and troughs	Very nice piece of work here. I have examined the sections, and any comments I have before are listed by section, IN CAPS.	Nil	No change	

Ecosystem Functional Group (EFG)	General comment	Specific comments	Response type	Detailed response
M3.6 Hadal trenches and troughs	Overall I find the document to be well-written and the EFGs chosen are appropriate. There is no need for major changes. Otherwise I have no major comments. I have made annotations to the attached text. I found that the important points have been captured in the very succinct text and diagrams in an exemplary way. Congratulations to the authors!	Could mention the topographic "funneling" effect whereby sediment, organic matter and pollutants tend to be concentrated in the axis of the trenches.	Minor edits to text	Added " the topographic "funneling" effect whereby sediment, organic matter and pollutants tend to be concentrated in the axis of the trenches. "
M3.6 Hadal trenches and troughs	here you have few suggestions for the text you sent me some days ago. In my opinion, the text is very good and I only made some appreciations that can be considered (or not). Good job!	Nil	No change	
M3.7 Chemosynthetically-based ecosystems	Very nice piece of work here. I have examined the sections, and any comments I have before are listed by section, IN CAPS.	Nil	No change	

Ecosystem Functional Group (EFG)	General comment	Specific comments	Response type	Detailed response
M3.7 Chemosynthetically-based ecosystems	Overall I find the document to be well-written and the EFGs chosen are appropriate. There is no need for major changes. Otherwise I have no major comments. I have made annotations to the attached text. I found that the important points have been captured in the very succinct text and diagrams in an exemplary way. Congratulations to the authors!	Nil	No change	
M4.1 Submerged artificial structures	It reads very well and I can only imagine the time it took to synthesise all of the information into meaningful summaries, congrats! I don’t have any major comments, just a few minor questions and suggestions that are hopefully helpful	M4.1. This section includes oil and gas infrastructure, but doesn’t talk about some of the differences associated with these as compared to structures that have been purposely sunk as artificial reefs. Oil and gas infrastructure are more impacted by human activities because of the light/noise/chemical pollution associated with operation and drilling activities. They’ve also be associated with the spread of invasive species. Recommend differentiating some of these drivers.	Minor edits to text and diagram	Added text "Compared to artificial reefs, oil and gas infrastructure is more exposed to light/noise/chemical pollution associated with operations as well the spread of invasive species." Added 'light/noise/chemical pollution' and 'invasive species' to diagram

Ecosystem Functional Group (EFG)	General comment	Specific comments	Response type	Detailed response
M4.1 Submerged artificial structures	Many thanks for the opportunity to comment on this. It looks like a fantastic resource.	MT4.1 Submerged artificial structures<br>I'm not so keen on the Claisse ref. I think it's misleading. Why not include a review instead? Suggestion<br>- Lima, J.S., Zalmon, I.R. and Love, M., 2019. Overview and trends of ecological and socioeconomic research on artificial reefs. Marine environmental research. 145: 81-96<br>https://www.sciencedirect.com/science/article/abs/pii/S0141113618307888	Minor edits to refs	Replaced ref as suggested

Ecosystem Functional Group (EFG)	General comment	Specific comments	Response type	Detailed response
M4.1 Submerged artificial structures		1. Ecological traits - "but structures with high vertical relief are distinguished by an abundance of zooplanktivorous fish" - While (In Ecological Drivers section below - I would agree that "continuously transport plankton" is what supports the biological production of these habitats, I would suggest revising the first quoted sentence above to "...distinguished by an abundance of zooplanktivorous and other reef associated fishes." For all the platforms off of California the majority of the fishes, and to some degree elsewhere, could be better described as reef associated but are not direct zooplankton feeders, so I think saying it is primarily zooplanktivorous fishes would not be accurate (in many cases at least).<br>2. Distribution - could be updated using the attached recent reference (Scarborough-Bull 2019) which provides a global review of the distributions of oil and gas platforms and therefore this section could expand beyond "...Asia-Pacific alone, over 600 oil and gas projects..." to have additional regional estimates, or at least a single global estimate.	Minor edits to text and refs	1. Added phrase: distinguished by an abundance of zooplanktivorous fish, "as well as reef-associated fishes."<br>2. Amended Distribution text: "Millions of artificial reefs and fish-attraction devices are deployed in coastal waters worldwide, including >10,000 oil and gas structures, mostly in tropical and temperate waters. More than 500 oil and gas platforms were decommissioned and left as artificial reefs in US waters since 1940. Many others are candidates for reefing after decommissioning in coming decades (> 600 in the Asia-Pacific alone). "- Added Scarborough-Bull (2019) ref
M4.1 Submerged artificial structures		Delete quaifier "occasional" from pelagic fish	Minor edits to text	Deleted "occasional"

Ecosystem Functional Group (EFG)	General comment	Specific comments	Response type	Detailed response
M4.2 Marine aquafarms	Many thanks for the opportunity to comment on this. It looks like a fantastic resource.	MT4.2 Marine aquafarms<br>1. Formatting point - the last sentence of the key ecological drivers section comes out beyond the rest of the text. Not important but just thought I would point it out in case.<br>2. Diagram - Should "enhanced parasites" be added to the "biotic interactions" box, or indeed diseases?	Minor edits to diagram	1. Thank you but there is no error in formatting.<br>2. Added pathogens, disease to Biotic interactions box in diagram
M4.2 Marine aquafarms	I am content that this is a comprehensive and reasonable representation of this particular ecosystem functional group.	1.Allochthonous, with the exception of marine plant cultivation …<br>2. unless the target species are aquatic macrophytes or filter feeding molluscs<br>3. Target biota harvested… Comment: Not sure what you mean here by 'target biota'. If you are referring to the farmed aquatic organisms, then I can't think of any that might be harvested for fish meal, unless as a co-product derived from processing of farmed fish.<br>4. I wouldn't use the term 'genetically modified' as it is broadly accepted to mean that alien germplasm has been inserted (i.e. it is a GMO). Although I don't quite agree with the term, as it is somewhat value judgement loaded, the more usual term in this context is 'genetically improved', which refers to conventional selective breeding for such traits as faster growth, greater fillet yield, resistance to disease, etc. I am attaching a recent publication by the FAO which explains.<br>5. Remove 'intensive' before 'hatchery'<br>6. Add : "... by scaring" or culling	Multiple edits to text	1. Cultivated marine plants contribute autochthonous energy, even though the pants themselves are introduced by humans (no change)<br>2. Only primary producers contribute autochthonous energy, molluscs are consumers (no chnage)<br>3. Replaced "Target" biota with "Farmed" biota throughout. Deleted "fish meal"<br>4. Replaced "genetically modified" with "genetically improved for such traits as faster growth, greater fillet yield, resistance to disease, etc." Added followng word: bred "selectively" and then...<br>5. Deleted 'intensive' before 'hatchery'<br>6. Added : "... by scaring" or culling<br>7. Rephrased as follows: "The enclosures "impose" barriers to the movement of larger organisms. [Did not add "wild" organisms because sentence applied to both wild and farrmed larger animals<br>8. Added the follownig text: "Escapees may interact with wild biota through completion, predation and genetic introgression."<br>9. Added word as suggested: ...enabling the "farm" ecosystem to extend...<br>10. Deleted "anti-fouling compounds" from sentence on land-based aquaculture systems. Added to sentence n marine systems as follows: Marine enclosures have netting and frames that provide substrates for biofilms and a limited array of benthic organisms (controlled with anti-fouling chemicals), but ..."

Ecosystem Functional Group (EFG)	General comment	Specific comments	Response type	Detailed response
		7. Add words (in bold italics): The enclosures can constitute barriers to the movement of larger wild organisms<br>8. You don't say much about escapes but there is considerable concern about impacts on predation on and competition with wild stocks, as well as genetic introgression and its impact on fitness (e.g. feral farmed Atlantic salmon and wild Atlantic salmon).<br>9. Suggested added work: enabling the farm ecosystem to extend<br>10. Anti-fouling compounds aren't generally used on land-based aquaculture systems.		
Marine-Terrestrial				
MT1.1 Rocky shores	Many thanks for the opportunity to comment on this. It looks like a fantastic resource.	MT1.1. Rocky shores<br>1. Diagram.Disturbance regimes could also include ice-scour<br>2. Perhaps change "tidal exposure" to include both Exposure to wave action and exposure to aerial conditions (the horizontal and vertical gradients respectively)<br>3. Should there be anything in there about humans? Like overharvesting, impacts of trampling	Minor edits to text	MT1.1. Rocky shores<br>1. Added ice sour to Disturbance box in Diagram.<br>2. Added "t wave action" Ambient environment box<br>3. Human influences (e.g.overharvesting, impacts of trampling) are beyond the scope of the profiles and will be addressed in ecosystem assessments
MT1.1 Rocky shores	To me, all profiles adequately cover the variation within those biomes and their global distribution as far as I know.	Diagram: Add Wetting/drying cycles? Thermal stress?	Minor edits to diagram	Diagram: Added "ThermaL stress" to Ambient environment box, and "wet/dry cycles" to arrow from there t Resources box

Ecosystem Functional Group (EFG)	General comment	Specific comments	Response type	Detailed response
MT1.1 Rocky shores	Nice overviews of these marine systems. My comments are below. For some sections I included references. I recognize that there is space for just a reference or two in section, but thought the authors/editors would appreciate the basis for some of my comments in their revisions.	Overall good. Says “where waves have eroded softer rocks”. Really wave (and ice) have eroded sediments. Intertidal areas can be comprised of relatively soft rocks such as sandstone and mudstone	Minor edits to text	Simiplied sentence as follows: "Found globally at the margins of oceans, where waves are eroding rocks."
MT1.1 Rocky shores		1. I would reverse the clauses in this sentence to avoid the possible implication that all rocky shores are waveswept - 'In wave-swept environments, morphological adapatations include...'<br>2. Add 'and terrestrial run-off.' to last sentence of Traits section<br>3. Salinity could/should also be mentioned here - gradients from estuaries to fully marine open coasts.	Minor edits to text	1. Rephrased sentence as follows: "In wave-swept environments, adaptations include morphologies, such as small body sizes and small cross-sectional areas to minimise drag. "<br>2. Added 'and terrestrial run-off.' to last sentence of Traits section<br>3. Rephrased to include salinity gradient as follows: "Horizontal gradients of diminishing salinity and wave exposure from headlands to bays or inlets influences community composition and morphology. "

Ecosystem Functional Group (EFG)	General comment	Specific comments	Response type	Detailed response
MT1.2 Muddy shores		I would assume they would also compete with other vegetation types in other localities, such as saltmarsh and reed beds such as Spartina. Given the text about subtidal muddy and sandy substrates, it's probably worth it to indicate that this habitat type is also unvegetated.	Minor edits to text	Added phrase to existing text in traits section after ephemeral seagrasses mention as follows: "Otherwise, macrophytes are generally absent unlike other ecosystems on intertidal mudflats (MFT 1.2, MFT1.3)." Added a sentence at the end of trait section as follows: "Transitions to mangrove (MFT1.2), saltmarsh or reedbed (MFT1.3) ecosystems may occur in response to isostatic or sea level changes, freshwater inputs or changes in currents that promote macrophyte colonisation.." Deleted the following sentence from Distribution section: "In warmer regions, muddy shorelines compete with mangroves."
MT1.3 Sandy shores	To me, all profiles adequately cover the variation within those biomes and their global distribution as far as I know.	1. Do these also need to be defined in terms of sediment grain size (i.e. sand class?).<br>2. Relative wave vs tidal energy.<br>3. Diagram: Tidal and nearshore currents? in Ambient box	Minor edits to text and diagram	1. Rephrased 2nd sentence in Drivers section as follows to define particle size from "Particle size..." to: "Sand grain size (>85% grains 0.0625-2mm diam.)..."<br>2. Incorporated relative energy of waves and tides into text as follows: Sand grain size (>85% grains 0.0625-2mm diam.), wave and tidal regimes, "and their relative energy," determine beach morphology...<br>3. Added "Currents to Ambient environment box. Insfficient space for more detail
MT1.4 Boulder-cobble shores	Nice overviews of these marine systems. My comments are below. For some sections I included references. I recognize that there is space for just a reference or two in section, but thought the authors/editors would appreciate the basis for some of my comments in their revisions.	1. Overall looks good. For "Distribution" section, add that cobbles beaches occur where now-retreated glaciers have deposited cobbles.<br>2. Add to sentence to state that "Large boulders, however, are only disturbed during large storms and have more stable temperatures so more fauna can persist on their surface.<br>3. Ref: Gedan, K. B., J. Bernhardt, M. D. Bertness, and H. M. Leslie. 2011. Substrate size mediates thermal stress in the rocky intertidal. Ecology 92:576-582	Minor edits to text and refs	1. Added glaciers as follows: "Cobble beaches occur where rivers or glaciers delivered large quantities of cobbles ..."<br>2. Added phrase to sentence as suggested: Large boulders, however, are only disturbed during large storms "and have more stable temperatures" so more fauna can persist on their surface.<br>3. Added Gerdan et al. (2011) to ref list

Ecosystem Functional Group (EFG)	General comment	Specific comments	Response type	Detailed response
MT1.4 Boulder-cobble shores	Attached is the pdf with just a couple of comments. Otherwise the diagrams and distribution (from what I know) of this biome type all look good to me	1. "Cobble beaches are typically steep because waves easily flow through large interstices between coarse beach particles, reducing the effects of backwash erosion." - Also because of typically steeper nearshore gradients, swash zone and breaking zone tend to be of similar width (like gravel beaches).<br>2. "Cobble beaches occur where rivers deliver large quantities of cobbles to the coast or where they are eroded from nearby coastal cliffs." - Alongshore grading of sediment (i.e. by size) could occur on long, drift dominated shorelines which may influence sediment caliber on the beach.<br>3. Diagram: "Abrasion" cf grinding<br>4. Diagram: Presence of fine matrix amongst cobbles	Minor edits to text and diagram	1. Added the following text: "Hence, swash and breaking zones tend to be similar widths."<br>2. Added the following text: "Alongshore grading of sediment by size could occur on long, drift-dominated shorelines, which may influence sediment calibre on the beach."<br>3. Diagram: "Abrasion" cf grinding<br>4. Diagram: Presence of fine matrix amongs cobbles
Biome MT2 Supralittoral coastal systems	I have reviewed the chapter: MT2.1 Coastal shrublands and grasslands. I've made just some minor comments (which are described directely on the pdf). Don't hesitate if you need some clarifications. Thank you very much	I think this is a key feature, at least form the biodiversity point of view. I'd evidence better this biota could be found just here and nowhere else.<br>Somethting like this: Much of this biota is confined to supralittoral ecosystems and nowhere else ahlthough it may be widely distributed across different land masses by coastal winds, oceanic currents, and/or migratory behaviour .	Minor edits to text	Amended sentence as suggested, "Much of this biota is confined to supralittoral ecosystems and nowhere else, a key feature of these ecosystems, although it may be widely distributed behind shorelines on different land masses due to dispersal by coastal winds, oceanic currents, and/or migratory behaviour."

Ecosystem Functional Group (EFG)	General comment	Specific comments	Response type	Detailed response
MT2.1 Coastal shrublands and grasslands	I examined all the tropical systems, especially those I personal experience with. I am especially knowledgeable about Latin America, and in particular, systems occurring in Peru. In general, sufficient variation is covered, and I found the product useful.	P.122 Also get some wetlands associated with freshwater aquifer coming to ground surface before land meets the sea.	No change	These would be classified with relevant wetland group (e.g. F2.8)
MT2.1 Coastal shrublands and grasslands	I have reviewed the chapter: MT2.1 Coastal shrublands and grasslands. I’ve made just some minor comments (which are described directely on the pdf). Don’t hesitate if you need some clarifications. Thank you very much	1. I'd evidence not only salt tolerance but also substrate instability .<br>2. Modular and rhizomatous gowth is not only related to woody plants, it's even more related to herbaceous ones, so I suggest to include this sentece in the previus paragraph as suggested before<br>3. Diagram. I'd include substrate instability . For example: Desiccation and substrate instability tollerance<br>4. Map Probably this a low resolution picture but It seems to me that the coasts allover the world are included, without any exception. It is right?	Multiple edits to text and diagram	1. Added phrase to emphasise substrate instability mong major gradients: Vegetation and substrates are characterised by strong gradients from sea to land, "particularly related to aerosol salt inputs, substrate instability and disturbance associated with sea storms and wave action."<br>2. Added phrase to include herbaceous plants with reference to modular growth forms: ...modular and rhizomatous growth forms of "both woody and non-woody plants" promote persistence...<br>3. Diagram. Moved "substrate instability" from arrow label to Disturbance box, added arrow from Maritime winde box to Water availability box and labelled "desiccation"<br>4. Map Yes , map is indicative at low resolution. Most coasts have such supralitttoral ecosystems by finer-resolution cannot be shown here

Ecosystem Functional Group (EFG)	General comment	Specific comments	Response type	Detailed response
MT2.1 Coastal shrublands and grasslands		1. This unit is very inspecific as it includes the universally recognized three coastal systems all over the world: cliffs, dunes and marshes. Each of them have very different geomorphologic and biotic conditions and should be considered separately. Marshes are considered later in type MFT1.3, but cliffs and dunes are absent.<br>2. Diagram - Low endemism in Traits circle - Depend on the region, in the Mediterranean coasts Limonium and Armeria have a huge number of endemics, in addition to other genera, basically in coastal cliffs.	Minor edits to text	1. Marshes (as noted by reviewer) are included in MFT1.3, and not part of this group. Freely draining littoral dunes and cliffs are more similar to one another than either is to marshes. They are united by the influence of maritime aerosols, storm regimes and different degrees of substrate instability, as well as an oceanic biota, although there are important differences functionally and compositionally. These differences warrant consideration for separation into different functional groups in a future version of the typology, but currently thy are difficult to separate at a global level because of their juxtaposition at fine spatial scales. Added the following text to emphasize uniting ecosystems drivers: Vegetation and substrates are characterised by strong gradients from sea to land, "particularly related to aerosol salt inputs, substrate instability and disturbance associated with sea storms and wave action." Added the following text to acknowledge distinctions based on substrate: "Consolidated substrates (headlands, cliffs) may differ from unconsolidated dunes in their influence on function and biota." In diagram, added "-unconsolidated dunes" and "-lithic substrates" to Substrates & geomorphology box<br>2. Diagram - Added qualifier as follows: "Mostly low endemism

Ecosystem Functional Group (EFG)	General comment	Specific comments	Response type	Detailed response
MT2.2 Large seabird and pinniped colonies		1. I don't think this information is needed unless a clear causal connection can be made between the type of diet and a response in the terrestrial biome. I know of one paper which showed that differences in bird diet affected nearby plant communities. Differential responses of Arctic vegetation to nutrient enrichment by plankton- and fish-eating colonial seabirds in Spitsbergen - A. Zwolicki, K. Zmudczyńska-Skarbek, J. Matuła, B. Wojtuń and L. Stempniewicz. Frontiers in Plant Science 2016 Vol. 7 Issue 1959, DOI: 10.3389/fpls.2016.01959. But am not aware of any other papers that compared other ecosystem aspects (I am working on a review with an MSc student on the effects of bird derived nutrients on invertebrate groups but that is not finished). For the time being I think we have to view birds and seals as a source of nutrients without being able to distinguish between their ocean diet.<br>2. Assuming that this ecosystem typology deals with the terrestrial biome, the depletion of marine resources is not directly relevant here, unless we want to deal with temporal changes in nutrient input .<br>3. Perhaps change to: "Seabird and pinniped affected ecosystems occur worldwide and local plant communities are impacted by high nutrient loading, increased soil pH and large surface and	Multiple edits to text and diagram	1. Agree, sentence on dietary detail deleted.<br>2. Sentence rephrased to make context of surrounding waters clearer<br>3. Sentence modifed to include this and other information: "Colonies occupy diverse habitats, from sandy shores to rocky islands and montane forests, with vegetation composition and structure limited by physical disturbance, nutrient input, salt influx and gradient (e.g., sea spray), water deficit, surface and subsurface bioturbation-driven changes in soil condition and pH, avian seed dispersal, unstable substrates, and high exposure, often exhibiting salt tolerance and clonal reproduction."<br>4. Sentence revised to clarify (see response 3 above)<br>5. Repetition deleted<br>6. Added sentence as follows: "Trophic networks are characterized high microbial activity and abundant invertebrates in soils which can lead to localised biodiversity hotspots, in contrast to the low richness of plant communities under high nutrient loading."<br>7. Diagram: Biotic interactions:<br>-Bioturbation and disturbance - No change<br>-Competition: refers to in situ interactions within the ecosystem betwen plants and colonial animals (e.g. for nesting space, mates), but insuffiecint space in diagram to elaborate<br>-Predation: refers to in situ and proximal marine predation that shapes the colonial community (e.g. skuas, orcas). Insufficient space to elaborate in diagram.<br>8. Diagram Re the Ecosystem properties:<br>- Deleted 'declines with temperature'<br>- Revised to 'low plant diversity'<br>- Revised to 'Diverse invertebrate food web based on detritivores'<br>- Self-evident but key feature, hence retained<br>- Added 'mammalian'<br>- Added reference to salinity tolerance and clonal plant forms to text

Ecosystem Functional Group (EFG)	General comment	Specific comments	Response type	Detailed response
		sub-surface bioturbation through animal activity. The close proximity to the ocean induces further limitation for the vegetation as typically found for coastal ecosystems such as salt spray among various others (see MT1.1?)"<br>4. Not clear from the outset that this [colonial species] refers to the vegetation.<br>5. Already mentioned in the previous sentence [low richness of primary producers]<br>6. Soil communities within colonies tend to show high microbial activity and increased abundance of invertebrates (both detritivores and predators) which in polar regions can lead to localized biodiversity hotspots, in clear contrast to the plant community which suffers under the high nutrient loading.<br>7. Diagram: Biotic interactions:<br>-Bioturbation and disturbance impact by the animals has a logical link to the ecosystem properties<br>-Competition: not clear if this deals with the competition within the plant community or among the birds/seals (which in the latter case is not directly relevant as the birds and seals are 'merely' a vector for nutrients)<br>-Predation (marine) not clear how why this feeds into the ecosystem properties of the terrestrial biome. Unless you make an arrow from predation to Marine productivity and therethrough affect the marine subsidies to nutrient availability		- Added reference to vibrant specialized lichens to text<br>- Re-ordered items as suggested

Ecosystem Functional Group (EFG)	General comment	Specific comments	Response type	Detailed response
		on land.<br>8. Diagram Re the Ecosystem properties:<br>-I think the ‘declines with temperature’ can be removed as this is an aspect of climate and not related to the nutrient subsidies.<br>-Low diversity should be ‘low plant diversity’<br>-Simple trophic structure; not sure if supported by all data; my work in Antarctica indicates that invertebrate communities within the penguin colonies have highest diversity<br>-‘abundant birds/seals’ seems superfluous<br>-Herbivores and predators needs inclusion of ‘large’ or ‘mammalian’ as invertebrates tend to thrive<br>-Salinity tolerance and clonal plant forms are good points but probably need mentioning in the main text as well.<br>- May be worth mentioning colourful and specialized lichens that thrive near/among bird colonies. These tend to be very bright and noted by tourists while they don’t mind trampling the vegetation to get near their pet bird colony.<br>- Perhaps rethink the order items; plant productivity – plant diversity – growth forms and adaptations – other ecosystem components (food web) – site for long distance dispersal		

Ecosystem Functional Group (EFG)	General comment	Specific comments	Response type	Detailed response
MT2.2 Large seabird and pinniped colonies		1. I would also say that there is massive physical disturbance to plants/soils in these colonies and that is a distinguishing factor as well. Thinking of cormorant colonies in temperate areas where plants are annihilated due to both nutrient concentrations and physical disturbance.<br>2. This [reference to vegetation height] is confusing to me – there are no plants at all in dense colonies due to high nutrients + disturbance. It's more of a gradient based on density of animals and climate/precipitation.<br>3. Bioturbation also important as shown in the figure	Minor edits to text	1. Revised sentnece to incorporate physical disturbance as follows: "The marine-derived subsidies and potentially massive physical disturbance to vegetation and soils distinguish these colonies from otherwise similar ecosystems in MT2.1."<br>2. Revised text to clarify relationships of vegetation structure as follows: "Plant assemblages exist across a gradient, influenced by seabird/pinniped disturbance, nutrient input and climate, whereby high-density colonies can completely suppress plant growth, but where disturbance and nutrient load is lower, vegetation can establish, typically in low richness but high abundance. "<br>3. Added phrase on bioturbation to text: "Bioturbation, coastal storms, and unstable substrates influence biotic interactions and colony abundance and distribution."
Biome MT3 Anthropogenic shorelines	It reads very well and I can only imagine the time it took to synthesise all of the information into meaningful summaries, congrats! I don't have any major comments, just a few minor questions and suggestions that are hopefully helpful	MT3.1. It would be useful to explain the term ecological engineering. I recommend including some reference to the floating structures as well that can occupy more space sometimes than some of the fixed, hard infrastructure. These provide downward facing, floating habitat that isn't like much else found naturally in the oceans. While reading this I also started to think about where artificial islands might sit with respect to EFGs. They certainly create a lot of new artificial shorelines so perhaps a mention about them here would be relevant.	Minor edits to text	Added following phrase on artificial islands and floating structures: "...and includes some more remote structures such as artificial islands. ", "...adjoining, extending or replacing natural shores, or floating in proximity to them.", "Floating structures have downward-facing, usually smooth, surfaces, unlike almost anything in nature, which may be colonised by opportunists."

Ecosystem Functional Group (EFG)	General comment	Specific comments	Response type	Detailed response
MT3.1 Artificial shores	It reads very well and I can only imagine the time it took to synthesise all of the information into meaningful summaries, congrats! I don’t have any major comments, just a few minor questions and suggestions that are hopefully helpful	MT3.1. It would be useful to explain the term ecological engineering. I recommend including some reference to the floating structures as well that can occupy more space sometimes than some of the fixed, hard infrastructure. These provide downward facing, floating habitat that isn’t like much else found naturally in the oceans. While reading this I also started to think about where artificial islands might sit with respect to EFGs. They certainly create a lot of new artificial shorelines so perhaps a mention about them here would be relevant.	Minor edits to text	1. Added islands to opening sentence.<br>2. Reworded the phrase with 'ecologically engineered' to make it more self-explanatory "Some structures are ecologically engineered (designed for nature) to provide..." .<br>3..Added/edited text to read, "Floating structures have downward-facing surfaces, rare in nature... "
MT3.1 Artificial shores	Many thanks for the opportunity to comment on this. It looks like a fantastic resource.	MT3.1. Artificial shores<br>1. Not sure everyone will know what “ecologically engineered” means in sentence below. Might need a brief explanation in brackets (i.e. designed to make space for nature). Ecologically engineered structures have more complex surfaces and ponds to enhance biodiversity and ecosystem function.<br>2. Diagram. Perhaps also the human activity (engineering works) also needs to have an arrow going to ambient environment as structures alter wave exposure and turbidity etc…	Minor edits to text	1. Rephrased to clarify as suggested, "Some structures are ecologically engineered (designed for nature) to provide..."<br>2. Diagram
Marine-Freshwater-Terrestrial				

Ecosystem Functional Group (EFG)	General comment	Specific comments	Response type	Detailed response
MFT1.2 Intertidal forests and shrublands		for the salt marshes, mangroves and sea grasses there is no mention of the term blue carbon, which seem odd, their is a new paper by Macreadie et al. 2019 that summarizes their ecomonic importance published in nature communications.	Minor edits to text	Added phrases as follows: This in turn mobilises carbon and nutrients to higher trophic levels, "but these ecosystems are also major blue carbon sinks due to organic matter incorporated into sediments, as well as living biomass."
MFT1.2 Intertidal forests and shrublands	Nice overviews of these marine systems. My comments are below. For some sections I included references. I recognize that there is space for just a reference or two in section, but thought the authors/editors would appreciate the basis for some of my comments in their revisions.	1.Overall looks good. Make specific mention of the importance of nutrient limitation and herbivores.<br>2. Not clear if "pests" is meant to cover herbivores. See works by Feller including the following:<br>3. additional refs: Feller, I. C., C. E. Lovelock, U. Berger, K. L. McKee, S. B. Joye, and M. C. Ball. 2010. Biocomplexity in Mangrove Ecosystems. Pages 395-417 Annual Review of Marine Science.<br>Feller, I. C. 1995. Effects of nutrient enrichment on growth and herbivory of dwarf red mangrove (Rhizophora mangle). Ecological Monographs 65:477-505.	Minor edits to text and refs	1. Added the following text to Traits section: "They are highly efficient in nitrogen use efficiency and nutrient resorption." In the Drivers section, added the following text: "Many mangrove soils are low in nutrients, especially nitrogen and phosphorus."<br>2. Added explicit mention of herbivores as follows: "Mangrove canopies support "invertebrate herbivores and other" terrestrial biota ...<br>3. Added Feller et al. (2010) to ref list

Ecosystem Functional Group (EFG)	General comment	Specific comments	Response type	Detailed response
MFT1.3 Coastal saltmarshes		1. there are also C3 grasses<br>2. The map is not coverimg most of the coasts of the world	No change	1. C3 grasses are present in these ecosystems, but the sentence is about traits that "confer varied degrees of tolerance to salinity, desiccation, and substrate anoxia", hence C3 photosynthetic pathway is not mentioned<br>2. The map is based on published mapping by McOwen et al. (2017), summarised within a template of 1° grid cells. Hence small occurrences are not discernable at this scale. McOwen CJ, Weatherdon LV, van Bochove JW, Sullivan E, Blyth S, Zockler C, Stanwell-Smith D, Kingston N, Martin CS, Spalding M, Fletcher S (2017) A global map of saltmarshes. Biodiversity Data Journal 5, e11764

Ecosystem Functional Group (EFG)	General comment	Specific comments	Response type	Detailed response
MFT1.3 Coastal saltmarshes	Nice overviews of these marine systems. My comments are below. For some sections I included references. I recognize that there is space for just a reference or two in section, but thought the authors/editors would appreciate the basis for some of my comments in their revisions.	1. The arrow leading away from the “Biotic interactions” box says “Bottom-up regulation”, but herbivory is a “top-down” factor.<br>2. Check map of salt marsh distribution as some large areas seem to be missing such as Hudson Bay (and likely others?). There seems to be an overemphasis on physical drivers of saltmarshes.<br>3. Text should be added to highlight the role of competition, facilitation (by plants and animals), and herbivory in determining patterns of zonation, productivity & succession, and plant persistence respectively.<br>4. See body of work by Bertness including:<br>Bertness, M. D. 1991. Zonation of Spartina patens and Spartina alterniflora in a New England salt marsh. Ecology 72:138-148.<br>Bertness, M. D., and S. W. Shumway. 1993. Competition and facilitation in marsh plants. American Naturalist 142:718-724.<br>Bertness, M.D. and B.R. Silliman. 2013. Salt Marsh Communities. M. D. Bertness, J. F. Bruno, B. R. Silliman, and J. J. Stachowicz, editors. Marine Community Ecology and Conservation. Sinauer Associates, Sunderland, Massachusetts.	Multiple edits to text, diagram and map	1. Diagram: Added top-down to bottom-up reglation arrow; added Competiition and Facilitation to BiologicalInteractions box<br>2. Map: Saltmarshes do occur in Hudosn Bay, but have been depleted by snow geese (Jefferies et a. 2007). The base map is taken from MOwen et al. (2017), which omits Hudson Bay. We will add the occurrence mapped by Jeffereies et al. (2007).<br>3. Added follwoing text after first sentence: "Competition and facilitation mediate plant coexistence and strong zonation." rephrased last sentence of Traits section as follows: Migratory species that play important roles in the<br>dispersal of plants, invertebrates, and microbes, while abundant foragers may<br>force top-down transformational change.<br>4. Added Bertness & Shumway (1993) and Jefferies et al. (1993)

List of peer reviewers

Professor Alicia T.R. Acosta	Department of Environmental Biology, Università Roma, Italy
Dr Andrew H. Altieri	Department of Environmental Engineering Sciences, University of Florida, Gainesville, USA
Professor Alexandre M.B. Anesio	Department of Environmental Science, Aarhus University, Denmark
Professor Angela H. Arthington	Australian Rivers Institute, Griffith University, Australia
Dr Jorge Assis	Centro de Ciências do Mar (CCMAR), Universidade do Algarve, Faro, Portugal
Professor Stefan Bertilsson	Department of Aquatic Sciences and Assessment, Swedish University of Agricultural Sciences, Uppsala, Sweden
Professor Malcom Beveridge	University of Stirling, Stirling, Scotland
Dr P. Bhanumati	National Statistical Office, Ministry of Statistics and Programme Implementation, New Delhi, India
Professor Thomas S. Bianchi	Department of Geological Sciences, University of Florida, Miami, USA
Dr Patrick Bogaart	United Nations Statistics Division, The Hague, Netherlands
Dr Stef F. Bokhorst	Department of Ecological Science, Vrije Universiteit, Amsterdam, The Netherlands
Dr David Brankovits	Marine Biology Department, Texas A&M University, Galveston, USA
Professor Neil D Burgess	UN Environment Programme World Conservation Monitoring Centre (UNEP-WCMC), England;<br>Copenhagen University, Copenhagen, Denmark
Dr Jane Catford	Kings College London, England
Dr Alan Channing	School of Earth & Ocean Sciences, Cardiff University, Wales
Associate Professor Jeremy T Claisse	Biological Sciences, California State Polytechnic University, Pomona, USA
Professor Richard T. Corlett	Xishuangbanna Tropical Botanical Garden, China

Professor Tasman P. Crowe	School of Biology and Environmental Science, University College Dublin, Ireland
Dr Nick A. Cutler	School of Geography, Politics & Sociology, Newcastle University, England
Dr Katherine A. Dafforn	Department of Earth and Environmental Sciences, Macquarie University,Sydney, Australia
Professor David Eldridge	University of NSW, Australia
Dr Julie C. Ellis	School of Veterinary Medicine, University of Pennsylvania, Philadelphia, USA
Dr Louise B. Firth	School of Biological and Marine Sciences, University of Plymouth, England
Dr Eliza Fragkopoulou	Centro de Ciências do Mar (CCMAR), Universidade do Algarve, Faro, Portugal
Dr Vasilis Gerovasileiou	Hellenic Centre for Marine Research, Anavyssos, Greece
Professor David Gibson	Department of Plant Biology, Southern Illinois University, Carbondale, USA
Professor Paul S. Giller	University College Cork, Ireland
Dr Brett C. Gonzalez	Smithsonian National Museum of Natural History, Washington DC, USA
Professor Richard Harper	School of Veterinary and Life Sciences, Murdoch University, Perth, Australia
Dr Justin C. Havird	Department of Biology, Colorado State University, Fort Collins, USA
Professor J. Tim Hollibaugh	Department of Marine Sciences, University of Georgia, Athens, USA
Professor Grant C. Hose	Department of Biological Sciences, Macquarie University, Australia
Professor Thomas M. Iliffe	Marine Biology Department, Texas A&M University, Galveston, USA
Professor Kenneth Irvine	IHE Delft Institute for Water Education, Delft, Netherlands
Associate Professor Mary Kelly-Quinn	School of Biology and Environmental Science, University College Dublin, Ireland
Dr Tytti Kontula	Finnish Environment Institute, Helsinki, Finland

Professor Christian H. Körner	Department of Environmental Sciences, University of Basel, Basel, Switzerland
Megan Lamson	University of Hawaii, Hilo, USA
Professor Javier Loidi	Department of Plant Biology and Ecology, University of the Basque Country, Spain
Dr Alejandro Martínez García	Institute for Ecosystem Studies, Italian National Research Council, Italy
Dr Matthew McGlone	Manaaki Whenua -- Landcare Research, Lincoln, New Zealand
Dr Sarah E. McSweeney	Department of Geography, The University of Melbourne, Melbourne, Australia
Professor Robert J. Orth	Biological Sciences Department, Virginia Institute of Marine Science, Gloucester Point, USA
Dr Sean N. Porter	South African Association for Marine Biological Research, Durban, South Africa
Professor Imants G. Priede	Institute of Biological and Environmental Sciences, University of Aberdeen, Scotland
Professor Mark C. Rains	School of Geosciences, University of South Florida, USA
Dr Catherine A. Reidy Liermann	Huxley College of the Environment, Western Washington University, USA
Associate Professor Belinda J Robson	School of Environmental Science, Murdoch University, Perth, Australia
Associate Professor Sergio Rossi	Department of Biological and Environmental Sciences and Technologies, Università del Salento, Lecce, Italy
Professor Jeremy Russell-Smith	Bushfire and Natural Hazards CRC, Charles Darwin University, Darwin Australia
Dr Ryan R. Rykaczewski	Ecosystem Sciences Division, NOAA Pacific Islands Fisheries Science Center, Honolulu, USA
Dr Troy S. Sakihara	Department of Land and Natural Resources, Hawaii, USA
Dr Scott R. Santos	Department of Biological Sciences, Auburn University, Auburn, Alabama, USA
Dr Nadine Schubert	Centre of Marine Sciences (CCMAR), Universidade do Algarve, Faro, Portugal
Dena M. Sedar	Hawaii State Parks, Honolulu, USA

Barbara Seidel	The Nature Conservancy, Honolulu, USA
Professor Lynne Shannon	Department of Biological Sciences, University of Cape Town, South Africa
Dr Monica Sharma	Consultant to the United Nations, New Delhi, India
Professor Charles R.C. Sheppard	Department of Life Sciences, University of Warwick, Coventry, England
Professor Martin J. Siegert	Grantham Institute for Climate Change & Environment, Imperial College London, London, England
Professor Stephen E. Swearer	School of BioSciences, The University of Melbourne, Australia
Dr Richard Williams	CSIRO, Melbourne, Australia
Dr Ryan Woodland	Center for Environmental Science, University of Maryland, Solomons, USA
Professor Kenneth R. Young	Department of Geography and the Environment, University of Texas, Austin, USA
